# Histological and transcriptomic insights into the interaction between grapevine and *Colletotrichum viniferum*

**DOI:** 10.3389/fpls.2024.1446288

**Published:** 2024-08-16

**Authors:** Mengru Dou, Yuhang Li, Yu Hao, Kangzhuang Zhang, Xiao Yin, Zinuo Feng, Xi Xu, Qi Zhang, Wenwu Bao, Xi Chen, Guotian Liu, Yuejin Wang, Ling Tian, Yan Xu

**Affiliations:** ^1^ State Key Laboratory of Crop Stress Biology in Arid Areas, Northwest Agriculture & Forestry University, Yangling, Shaanxi, China; ^2^ College of Horticulture, Northwest A&F University, Yangling, Shaanxi, China; ^3^ Key Laboratory of Horticultural Plant Biology and Germplasm Innovation in Northwest China, Ministry of Agriculture, Yangling, Shaanxi, China; ^4^ School of Management, Shenzhen Polytechnic University, Shenzhen, Guangdong, China

**Keywords:** grape, *Colletotrichum viniferum*, genome annotation, fungal infection strategy, grapevine response

## Abstract

**Introduction:**

Grape is of high economic value. *Colletotrichum viniferum*, a pathogen causing grape ripe rot and leaf spot, threatens grape production and quality.

**Methods:**

This study investigates the interplay between *C. viniferum* by Cytological study and transcriptome sequencing.

**Results:**

Different grapevine germplasms, *V. vinifera* cv. Thompson Seedless (TS), *V. labrusca* accession Beaumont (B) and *V. piasezkii* Liuba-8 (LB-8) were classified as highly sensitive, moderate resistant and resistant to *C. viniferum*, respectively. Cytological study analysis reveals distinct differences between susceptible and resistant grapes post-inoculation, including faster pathogen development, longer germination tubes, normal appressoria of *C. viniferum* and absence of white secretions in the susceptible host grapevine. To understand the pathogenic mechanisms of *C. viniferum*, transcriptome sequencing was performed on the susceptible grapevine “TS” identifying 236 differentially expressed *C. viniferum* genes. These included 56 effectors, 36 carbohydrate genes, 5 P450 genes, and 10 genes involved in secondary metabolism. Fungal effectors are known as pivotal pathogenic factors that modulate plant immunity and affect disease development. *Agrobacterium*-mediated transient transformation in *Nicotiana benthamiana* screened 10 effectors (CvA13877, CvA01508, CvA05621, CvA00229, CvA07043, CvA05569, CvA12648, CvA02698, CvA14071 and CvA10999) that inhibited INF1 (infestans 1, *P. infestans* PAMP elicitor) induced cell death and 2 effectors (CvA02641 and CvA11478) that induced cell death. Additionally, transcriptome analysis of “TS” in response to *C. viniferum* identified differentially expressed grape genes related to plant hormone signaling (*TGA*, *PR1*, *ETR*, and *ERF1/2*), resveratrol biosynthesis genes (*STS*), phenylpropanoid biosynthesis genes (*PAL* and *COMT*), photosynthetic antenna proteins (*Lhca* and *Lhcb*), transcription factors (*WRKY*, *NAC*, *MYB*, *ERF*, *GATA*, *bHLH* and *SBP*), ROS (reactive oxygen species) clearance genes (*CAT*, *GSH*, *POD* and *SOD*), and disease-related genes (*LRR*, *RPS2* and *GST*).

**Discussion:**

This study highlights the potential functional diversity of *C. viniferum* effectors. Our findings lay a foundation for further research of infection mechanisms in *Colletotrichum* and identification of disease response targets in grape.

## 1 Introduction

Grape (*Vitis vinifera* L.), an ancient and economically significant, is consumed worldwide (Dong et al., 2023). However, grapes are susceptible to various pathogens, including *Colletotrichum viniferum*, particularly under high temperatures and humidity. This pathogen affects grape cultivars such as Pione, Kyoho, Ives, and Merlot (Echeverrigaray et al., 2020; Yokosawa et al., 2020), resulting in significant economic losses of approximately 67% in the Mid-Atlantic region of the United States and around 37% in Northeast China (Cosseboom and Hu, 2022; Ji et al., 2021). *C. viniferum* infects grapevine leaves, shoots, stems, and berries, causing grape ripe rot and leaf spot, with particularly severe damage to berries (Pan et al., 2016).

*Colletotrichum* species are identified and categorized in China and globally according to morphological and molecular features. Pathogenic species identified include *C. acutatum*, *C. aenigma*, *C. capsici*, *C. fruticola*, *C. gloeosporioides*, and *C. viniferum*, among others (Lei et al., 2016; Oo and Oh, 2017; Echeverrigaray et al., 2020). With the continuous development of sequencing techniques, high-throughput sequencing technology has been widely adopted. Several *Colletotrichum* species from various host plants have had their complete genomes sequenced, including *C. lindemuthianum* (Gutiérrez et al., 2016), *C. acutatum* (Han et al., 2016), *C. graminicola* (Buiate et al., 2017), *C. sublineola* (Buiate et al., 2017), *C. asianum* (Meng et al., 2019), *C. higginsianum* (Ayako et al., 2019), and *C. fioriniae* (Xu et al., 2024). Fungal genome sequencing has greatly enhanced our understanding of the interactions between hosts and pathogens. However, the genomic sequencing of *C. viniferum* CvYL2a on grape has only recently been published (Dou et al., 2022).

Different grape varieties exhibit varying resistance levels to *Colletotrichum*. Researchers have classified the disease resistance abilities of various grape cultivars and their hybrids (Shiraishi et al., 2007). A comprehensive study of grape ripe rot resistance using natural field assessment, field inoculation, and indoor fruit *in vitro* analysis highlighted the exceptional resistance of Chinese wild grapes as valuable disease-resistant germplasm resources (He and Ren, 1990). Subsequent research revealed that cultivars such as Emerald Seedless, Tano Red, and Rem46-77 (Aestivalis GVIT 0970) are susceptible to grape ripe rot, while Agawan, Huangguan, and Xiangfei are resistant (Kim and Oh, 2019). The diversity in resistance levels provides valuable germplasm resources for investigating grape ripe rot resistance mechanisms, crucial for breeding and evaluating new resistant grapevine cultivars, and developing innovative disease control methods. Several investigations have analyzed the infection processes of various *Colletotrichum* spp. isolates in host fruits, such as apple infected by *C. fructicola*, Guava infected by *C. gloeosporioides*, blueberry, strawberry, and almond affected by *C. acutatum* (Arroyo et al., 2005; Diéguez-Uribeondo et al., 2005; Wharton and Schilder, 2008; Moraes et al., 2013; Shang et al., 2020). However, limited research has focused on grape pathogenesis and the histological differences between susceptible and resistant varieties. Currently, control of *Colletotrichum* spp. mainly relies on fungicide use, which negatively impacts soil, food safety, and the environment. Therefore, it’s critical to develop more effective and environmentally friendly disease management strategies by studying potential plant-pathogen interactions with a more profound comprehensive approach.

Transcriptome sequencing is the most straightforward way to investigating gene expression levels, with numerous studies exploring transcriptome sequencing in host-pathogen interactions involving *Colletotrichum* species. Transcriptome analysis of the host included strawberry infected by *C. siamense*, sugarcane infected by *C. falcatum*, and Asian ginseng (*Panax ginseng*) infected by *C. panacicola* (Nandakumar et al., 2021; Zheng et al., 2022; Xia et al., 2023). Similarly, transcriptome analyses of *Colletotrichum* spp. have explored pathogenicity mechanisms in different host-pathogen interactions, such as *C. camelliae* on tea, *C. falcatum* on sugarcane, and *C. fructicola* on apple (Liang et al., 2018; Prasanth et al., 2022; He et al., 2023). However, real-time integration of transcriptomic data from both hosts and *Colletotrichum* spp. during infection remains limited. While several studies have investigated grapevine transcriptomes in response to *Colletotrichum* spp. infection, the transcriptomic dynamics of *Colletotrichum* itself are missing (Lei et al., 2022; Shen et al., 2022; Yu et al., 2023).

*Colletotrichum* spp. seriously threatens the yield and quality of grapes, especially affecting high-quality grape varieties with poor resistance. Fortunately, most wild grape germplasm resources in China are resistant to this fungus. Through histochemical staining and ultrastructural observation, *V. vinifera* cv. Thompson Seedless (TS), *V. labrusca* accession Beaumont (B) and *V. piasezkii* Liuba-8 (LB-8) were identified as highly sensitive, moderately resistant and resistant to *C. viniferum*, respectively. The transient transformation assays in *Nicotiana benthamiana* showed that *Colletotrichum* spp. effectors CoNIS1, Cte1, Cg2LysM and CgCFEM1 inhibited plant immune response, CtNUDIX and ChCEC3 induced cell death similar to HR response (Bhadauria et al., 2013; Tsushima et al., 2021; Zhao et al., 2023; Feng et al., 2024; Yuan et al., 2024). Effectors play different roles during infection. Currently, there are no studies about *C. viniferum* effector on grape, and the infection mechanism remains unclear. To uncover the infection processes of *C. viniferum*, particularly the roles of effectors during infection and the responses of grapevine to *C. viniferum*, we conducted a study focusing on pathogenesis, selecting the susceptible grapes “TS” for transcriptome analysis. Our aim was to screen *C. viniferum* pathogenic genes by transcriptome sequencing, leading to the identification of 85 effectors. These effectors were subsequently screened on *Nicotiana benthamiana* to examine their roles in the infection process. Simultaneously, we analyzed the grapevines’ responses to *C. viniferum*, providing insights into the interaction between *C. viniferum* and grapes and shedding light on its pathogenic mechanism.

## 2 Materials and methods

### 2.1 Fungal strain, plant materials, and inoculation

The *C. viniferum* strain CvYL2a, previously obtained and purified in our research facility, was cultured on potato dextrose agar (PDA) under dark conditions at 25°C. *C. viniferum* conidia were induced by transferring mycelia from PDA to a Carboxymethylcellulose (CMC) sodium fluid medium and incubating them at 150 rpm and 25°C for 3-5 days. Conidia were separated from mycelia by filtering with two-layer sterilization gauze and collected after centrifugation at 5000 rpm for 10 min. After two washes with sterile distilled water (SDW), the conidia were standardized to a 5 × 10^6^ conidia/mL concentration, as determined using a hemocytometer and a light microscope.

For conidia inoculation, undamaged fully expanded leaves were collected from the apex of “TS”, “B” and “LB-8” grown in the grapevine germplasm resource vineyard at Northwest A&F University in Yangling, Shaanxi, China. When inoculation was carried out in June 2020, average outdoor day/night temperature ranged between 32 and 15°C. The vineyard management is under regular management with fungicides and insecticides, and organic and compound fertilizers. After sterilizing the leaf surfaces spraying with 75% ethanol for 30 seconds and rinsing them three times with SDW, circular leaf discs with a diameter of 15 mm were prepared, and placed in sterile Petri dishes 90 mm in diameter with moist double-layer filter papers. These leaf discs were inoculated with 20 µL of the above concentration of conidia suspension, and incubated at 25°C with a relative humidity of 90% and a photoperiod of 16 h light/8 h dark. Leaves placed in trays were sprayed with the same concentration of conidial suspension under the same conditions. The experiment was conducted with nine leaf discs, the diameter of each leaf disc necrosis was measured three times. The experiment was repeated three times.

### 2.2 Staining and light microscope imaging

The leaf disks were immersed in a solution containing ethanol and trichloromethane (3:1, vol/vol) along with trichloroacetic acid (0.45% wt/vol) for 3 days, followed by clearing in chloral hydrate (250 g/100 mL water) for another 3 days.

For trypan blue staining, leaf discs underwent a 30-minute staining process using a solution comprising trypan blue (20 mg), lactic acid (20 mL), glycerol (20 mL), phenol (20 mL), and ddH_2_O (20 mL). For DAB (Diaminobenzidine) staining, leaf discs were immersed in 5 mL centrifuge tubes filled with DAB staining Zsolution (1 mg DAB/mL ddH_2_O, pH 3.8) for 20 min under vacuum in the dark, followed by incubation at 25°C for 8 h in tin foil bags, and examined with 200 × or 400 × magnification using a light microscope (BX-53, Olympus). The resolution of the photographs was 150 dpi.

### 2.3 Sample preparation and scanning electron microscopy

Grape leaves inoculated with *C. viniferum* CvYL2a were cut into 5 × 5 mm pieces and initially fixed in 4% (vol/vol) glutaraldehyde in 0.1 M phosphate buffer (pH 6.8) at 4°C overnight. After 4 × 10 min rises in the same buffer, the samples were subjected to dehydration in a series of ethanol solutions ranging from 30% to 90% (vol/vol), each step lasting for 15 min, followed by three cycles of absolute ethyl alcohol dehydration. Finally, these samples were dried using a CO_2_ critical point dryer before being coated with gold and observed with SEM (Nano SEM-450, America).

### 2.4 Sample preparation and transmission electron microscopy

Grape leaves inoculated with *C. viniferum* CvYL2a were cut into 1 × 1 mm pieces and fixed in 4% (vol/vol) glutaraldehyde in 0.1 M phosphate buffer (pH 6.8) at 4°C overnight. After 4 × 10 min washes in the same buffer, the samples underwent post-fixation using a solution containing 1% osmium tetroxide (OsO_4_) at 4°C for 2 h and were subjected to dehydration as described in Section 2.3. Next, these samples were infiltrated with London Resin Company Ltd (LR) White Resin (Basingstoke, UK) in a series of proportions, 3:1 (vol/vol) for 2 h, 1:1 (vol/vol) for 8 h, 1:3 (vol/vol) for 12 h, and pure resin for 48 h, with a change every 24 h. Finally, they were immersed in pure resin and polymerized at 55°C for 48 h.

For semi-thin sections of 1µm thickness, samples, cut using a glass knife, were treated with 0.3% aqueous toluidine blue in 1.89% sodium tetraborate and observed with bright-field microscope (Olympus BX-51, Japan). Ultra-thin sections (90 nm) were prepared using a diamond knife and treated with uranyl acetate and lead citrate before being examined via a transmission electron microscope (HT7700, Japan).

### 2.5 Measurement of reactive oxygen species production rate

Plants rapidly produce reactive oxygen species (ROS) that directly inhibit the growth of pathogens during pathogens infection (Li et al., 2021). The DAB staining method is commonly used to detect H_2_O_2_ production (Huckelhoven et al., 1999; Ding et al., 2019). H_2_O_2_ production in both resistant and sensitive leaves was observed after DAB staining using a light microscopy. The rate was determined as the percentage of appressoria inducing H_2_O_2_ production in leaf cells to the overall count of appressoria. A total of 100 appressoria were randomly selected for assessment from each leaf, and this analysis was conducted on three blades at each time point. The experiment was performed with three independent biological replicates.

### 2.6 Gene family and phylogenetic evolution analyses

The gene sets of 14 fungal species were filtered, and similarity relationships between protein sequences of all species were analyzed using DIAMOND (Buchfink et al., 2015), which were further clustered using OrthoFinder2 (Emms and Kelly, 2019). To identify common single copy orthologous genes, multi-sequence alignment of genes within each single-copy homologous gene family was conducted using MUSCLE (Edgar, 2004). The evolutionary tree was constructed using RAxML with the maximum likelihood model (Stamatakis, 2014). Divergence times were estimated by integrating the constructed evolutionary tree with data from the TimeTree website and analyzed using the MCMCTree program in the software r8s and PAML software packages (Sanderson, 2003; Hedges et al., 2006). Data from 14 genomes used in comparative genomics can be found in the National Center for Biotechnology Information (NCBI) data library. These links and fungal strain names were shown in **Supplementary Table 8**.

### 2.7 RNA-seq and data analysis

Frozen tissue samples were obtained from *C. viniferum* CvYL2a-infested “TS” leaves at five different time points (0, 6, 12, 24, and 48 hours post inoculation (hpi)). Total RNA from these samples was extracted by Novogene Corporation Inc using the TRIzol Reagent (Invitrogen, Cat no. 15596026) and confirmed for integrity by 1% agarose gel electrophoresis. RNA purity and concentration were assessed using Nanodrop (Thermo Scientific, USA) and Agilent 5400 (Agilent Technologies, USA), respectively. Sequencing libraries of 150 bp paired-end reads were prepared and sequenced on an Illumina Novaseq platform. Clean reads were filtered by trimming the adapter sequences and low-quality reads of raw reads. High-quality reads were aligned to the *C. viniferum* CvYL2a (Dou et al., 2022) and the grapevine (12 × PN40024) reference genomes using HISAT2 (Mortazavi et al., 2008). Gene expression levels were quantified by calculating the fragments per kilobase of transcript per million mapped readings (FPKM). Differential expression genes (DEGs) were identified by comparing the inoculated samples at each infection time point with the 0 h samples using DESeq2 software (Love et al., 2014), with criteria set at Log_2_|(fold change)| > 4 and *P*-value < 0.05 via Benjamini and Hochberg’s approach. The gene ontology (GO) terms were categorized using GOSeq (Joung et al., 2010), and KEGG (http://www.genome.jp/kegg/) was used to explore the metabolic pathways of DEGs. Visualization of the results was done using venn diagrams and heatmaps created using TBtools v0.664 software (Chen et al., 2018).

### 2.8 qRT-PCR analysis

45 total RNA from three different grape germplasms, five inoculation periods (0, 6, 12, 24, and 48 hpi) with *C. viniferum* infection, and three replicates per period were extracted following the manufacturer’s instruction (R6827-01, Omega Bio-tek, USA). TransScript^®^ One-Step gDNA Removal and cDNA Synthesis SuperMix (AT311-02, Trans gen, China) was used for the synthesis of first-strand cDNA. Primers are listed in **Supplementary Table 2**. Real-time PCR amplification was conducted using TransScript^®^ II Green One-Step qRT-PCR SuperMix (AQ311-01, Trans gen, China).

### 2.9 Determination of endogenous hormone concentrations

According to the description of the method of extracting grape leaves hormones (Li et al., 2021b), 100 mg of TS leaves infected *C. viniferum* per period were promptly crushed in liquid nitrogen, followed by extraction with 1 mL of solvent (glacial acetic acid: methanol: isopropanol = 1: 20: 79 (v/v/v)). The concentration of hormones including salicylic acid (SA), ethylene (ET) synthesis precursor ACC and jasmonates (JA) in the extract was quantified by liquid chromatography-quadrupole ion trap-mass spectrometry (LC-QTRAP-MS, USA). There were three biological replicates for each hormone.

### 2.10 Plasmid construction and *Agrobacterium* transformation

SignalP and TMHMM software were used to select genes with signal peptide domain and TMHM without transmembrane domain as candidate effectors. 85 putative *C. viniferum* effectors were amplified, using cDNA as templates, with their predicted N-terminal signal sequences removed. The source of the cDNA was RNA extracted from grape leaves inoculated with *C. viniferum*. Specific primers designed (**Supplementary Table 2**) for each effector can be found in Appendix T. Subsequently, these sequences were cloned into vector pCAMBIA2300-GFP (Green fluorescent protein). Recombinant plasmids were transformed into *Agrobacterium tumefaciens* strain GV3101 using the liquid nitrogen freezing-thawing method. *Agrobacteria* carrying effector-GFP constructs were cultured in LB liquid medium, collected after centrifugation at 5000 rpm for 3 min, and resuspended in infiltration buffer (10 mM MgCl_2_, 10 mM MES (pH 5.7), 200µM acetosyringone) to achieve an OD_600_ of 0.6. The suspension was incubated for 3 h at 28°C in dark before infiltrating the leaves. Healthy leaves of 4 to 5 week-old *N. benthamiana* were infiltrated using a 1-mL needless syringe. INF1 (infestans 1, *P. infestans* PAMP elicitor) and GFP served as positive and negative controls, respectively. For experiments designed to suppress cell death triggered by INF1-GFP, leaves were infiltrated with effectors 12 h before INF1-GFP infiltration. Finally, leaf symptoms of cell death were evaluated through photography.

## 3 Results

### 3.1 Evolution of gene family in *C.viniferum* CvYL2a reveals unique features

**Figure 1:**
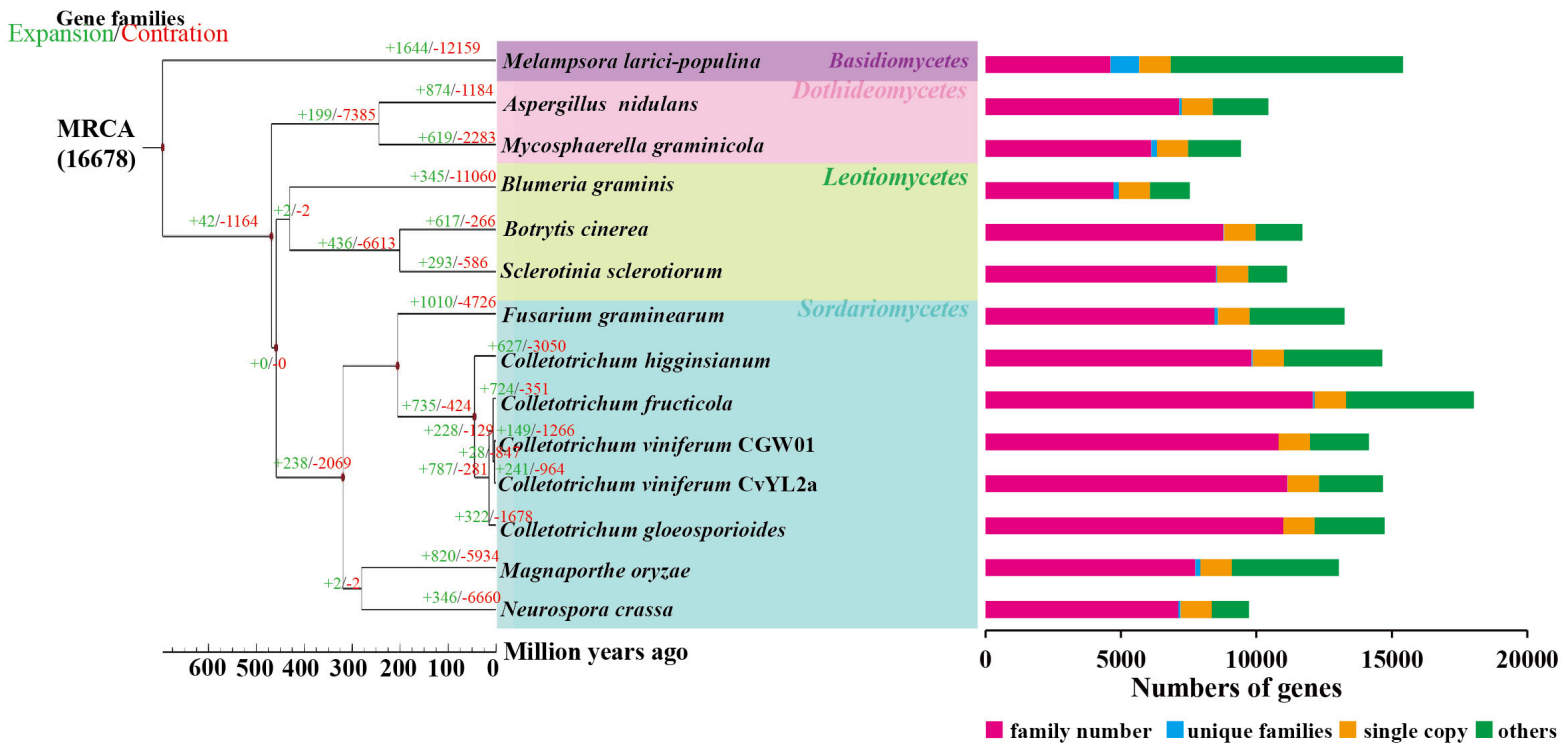
Phylogenetic relationship. The maximum likelihood phylogenetic tree was constructed using PhyML, based on the concatenated alignment of 1155 conserved single-copy orthologs from all species. Divergence time estimation for each species was performed utilizing the phylogenetic tree, software r8s, and the mcmctree program in the PAML software package. Time-correction points were obtained by integrating data from the TimeTree website.

To analyze the evolutionary relationships among *C. viniferum* CvYL2a and 13 other fungi, we clustered their proteins using OrthoFinder2. This process identified 16678 orthogroups containing 177914 proteins (**Supplementary 1**). From them, we selected 1155 single-copy orthologous families for phylogenetic analysis (**Supplementary 2**). Our findings indicate a close evolutionary relationship between *C. viniferum* CvYL2a and *C. viniferum* CGW01. CvYL2a appears to have diverged 3.2 million years ago within the genus *Colletotrichum*, which initially diverged approximately 45.2 million years ago (**Figure 1**). Additionally, we identified 33 specific genes in *C. viniferum* CvYL2a grouped into 13 categories. Notably, 20 of these genes (A08618, A10003, A09924, A08597, A01565, A09902, A09903, A09928, A11559, A09926, A09909, A01566, A10007, A09984, A09985, A10008, A14055, A10009, A11149 and A11147) lack significant homologous sequences in the NCBI database (**Supplementary Table 1**).

### 3.2 Symptoms of *C. viniferum* infection in three grape germplasms

**Figure 2:**
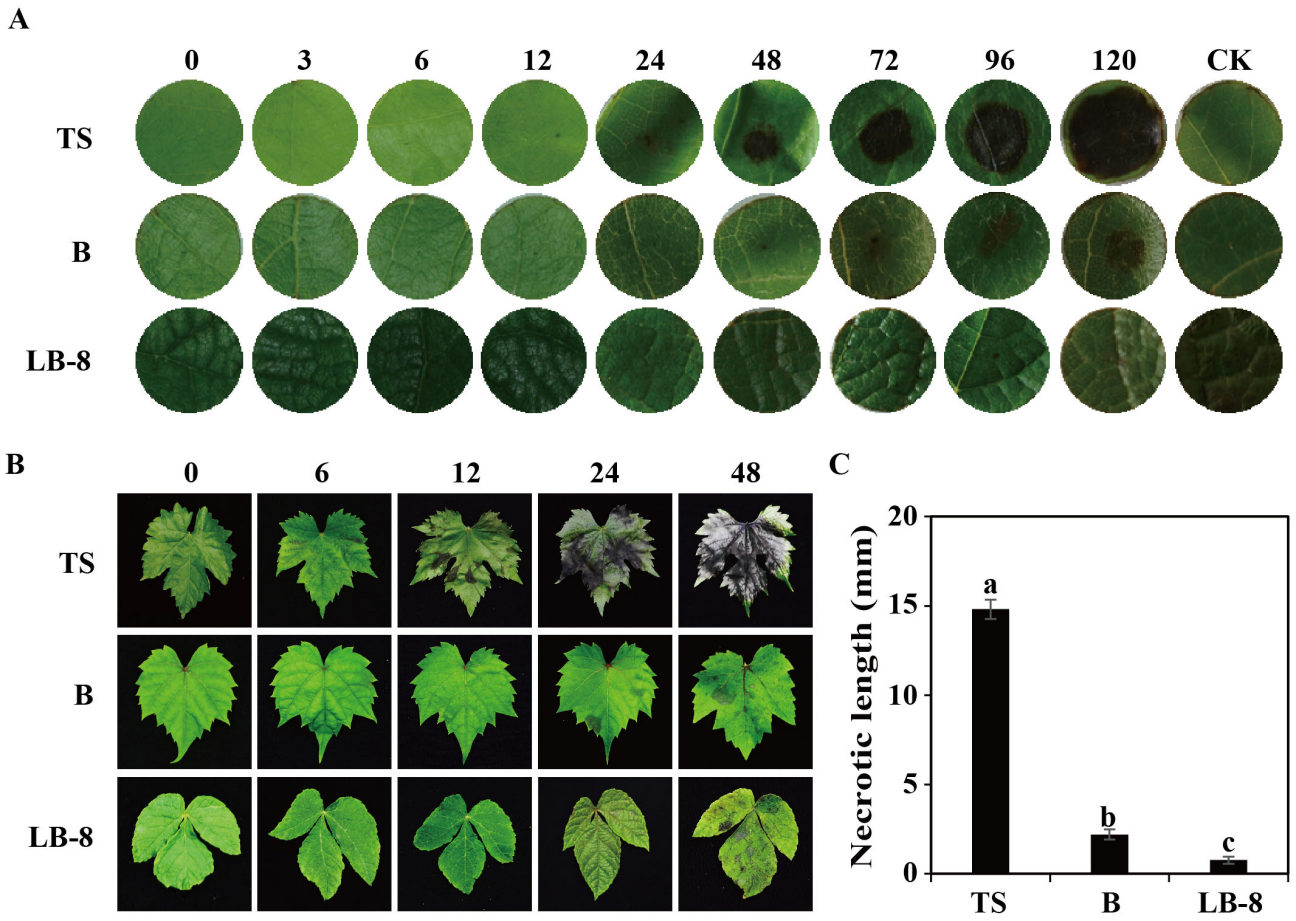
Ripe rot symptoms on three grapevine germplasms infected with *C. viniferum*. **(A)** Images captured at various time points [0, 3, 6, 12, 24, 48, 72, 96, and 120 hours post inoculation (hpi)] after applying 20 µl of fungi suspension (5 × 10^6^ conidia/ml) and incubating at 25°C. **(B)** Images were taken at 0, 6, 12, 24, and 48 hpi. Minimal Lesions were observed on “B” and “LB-8”, whereas necrosis rapidly developed on “TS” from 12 hpi. One representative leaf of three replicates is shown for each time point. **(C)** Symptoms evaluation through measurement of necrotic length at 120 hpi. Data represent the means ± SE of three independent experiments with three replicates taken from different plants, each containing ten replicates for each genotype. Distinct letters indicate significant differences (ANOVA test with Tukey’s comparisons, *P* < 0.05). TS, “*V. vinifera* cv. Thompson Seedless”; B, “*V. labrusca* accession Beaumont”; LB-8, “*V. piasezkii* Liuba-8”.

*C. viniferum* infection on grape leaves results in necrotic lesions, a typical symptom of grape ripe rot disease. To study the differences in resistance among the three grape germplasm resources to *C. viniferum*, we used leaf disc assays to observe necrotic lesion development. Small necrotic lesions appeared on “TS” leaves within 24 hpi, progressively expanding to a diameter of 14.81 cm by 120 hpi (**Figures 2A, C**). In contrast, “B” showed smaller and milder lesions from 72 to 120 hpi. Remarkably, “LB-8” exhibited minimal or no lesions at 120 hpi. When leaves were sprayed with the spore suspension, similar symptoms were observed. Brown grape leaf spots initially appeared at 12 hpi on “TS”, with spots enlarging and coalescing by 24 hpi, and large brown speckles became visible at 48 hpi. On the contrary, “B” and “LB-8” did not exhibit any disease necrotic spots before 24 hpi and small brown spots were observed only by 48 hpi (**Figure 2B**). Based on the grape ripe rot resistance grading standard (Gao et al., 2016), “TS”, “B” and “LB-8” were classified as high-sensitive (HS), moderate resistance (MR) and resistant (R) to *C. viniferum*, respectively.

**Figure 3:**
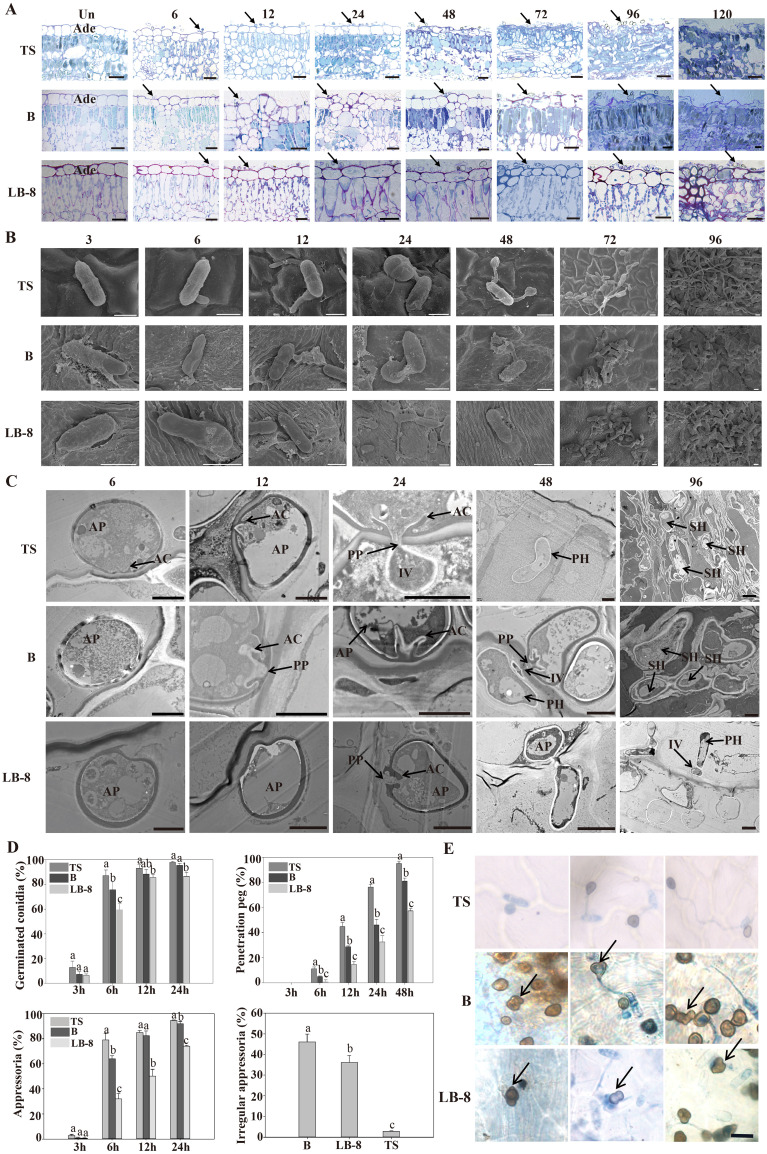
Histological and ultrastructural study of resistant and susceptible grapevine leaves infected by CvYL2a. **(A)** Cytological study of *C. viniferum* CvYL2a infected grape leaves. Un: Un-inoculated with *C. viniferum*; Ade, adaxial epidermis; scale bar, 20 μm. **(B)** SEM imaging of conidia development on leaves infected by *C. viniferum*. Scale bar = 5 μm **(C)** Ultrastructure of host-parasite interactions on “TS”, “B”, and “LB-8” leaves under TEM. AP, appressorium; AC, appressorial cone; Cu, cuticle; FCW, fungal cell wall; HCW, host cell wall; HPM, host plasma membrane; IV, infection vesicle; PP, penetration peg. Scale bar = 2 μm. **(D)** The percentages of *C. viniferum* germinated conidia, appressoria, and penetration peg on three grape germplasms at different time points post-inoculation, along with the presence of irregular appressoria on the three grape genotypes at 48 hpi, results are presented as mean ± SE, n=9. Different letters indicate significant differences (ANOVA test with Tukey’s comparisons, *P* < 0.05). **(E)** Occurrence of more mature appressorium deformities on “TS”, “B” and “LB-8” inoculated with *C. viniferum* CvYL2a. Deformed appressorium are marked by the arrow. Scal bar = 10 μm.

To study the differences in the infection process in three grape germplasms, we observed cytological changes under a light microscope after toluidine blue staining. From 6 to 24 hpi, conidia were distributed across the epidermal cells of “TS” leaves. By 48 hpi, epidermal cells began to collapse, reaching complete destruction at 72 hpi. At 96 hpi, the palisade mesophyll cells were also destroyed and leaf tissues had completely collapsed by 120 hpi. Compared to “TS”, “B” exhibited delayed symptom onset, with epidermal cells becoming concave at 72 hpi, and noticeable damage observed by 120 hpi. Remarkably, the epidermal cells of “LB-8” remained intact at 120 hpi (**Figure 3A**).

### 3.3 Developmental differences of *C. viniferum* on the leaves of three grape germplasm resources

To study developmental differences of *C. viniferum* on the leaves of three grape genotypes, we performed scanning and transmission electron microscopy observations. Initially, conidia formed a central septum at 3 hpi, dividing the conidia into two equal-sized parts on the leaves of all three grapevines. Additionally, the conidia on “B” and “LB-8” leaves were surrounded by abundant white secretions compared to “TS” (**Figure 3B**). Previous studies of downy mildew infection have shown that resistant grape varieties also produced white secretions (Yin et al., 2017). Subsequently, these conidia germinated and formed germination tubes from one or both ends of the conidia (**Figure 3B**). Although conidial germination in all three grapevines started at 3 hpi and significantly increased by 6 hpi, “TS” exhibited the highest germination rate at 24 hpi (**Figure 3D**). Interestingly, at 72 hpi, “B” and “LB-8” elongated germination tubes were shorter compared to “TS”, a difference that persisted even at 120 hpi (**Figure 3B**).

Next, appressoria developed from conidia, either directly or from the elongated germination tube, resulting in the production of several appressoria from a single conidium (**Figure 3B**). Appressorium formation became evident by 6 hpi on the leaves of all grape germplasms, with the highest appressorium formation rate occurring on “TS” by 24 hpi (**Figure 3D**). The shape of the appressoria is generally round or irregular in some cases (Velho et al., 2016). Irregular appressoria, such as downward concave hearts, gourds, and outward protruding convex morphology, were easily found with a proportion over 30% on “B” and “LB-8” (**Figures 3D, E**). This was possibly due to appressorium collapse caused by secretions from “B” and “LB-8” (**Figure 3B**) (Yin et al., 2017) or dense hair on grape leaves (**Figure 3C**; **Supplementary Figure 1**) (Kortekamp and Zyprian, 1999; Han et al., 2021). The paraxial surface of the B and LB-8 blades is covered with hairs, while the TS surface is hair-free.

The appressoria on “LB-8” appeared deflated and irregularly shaped compared to those on “TS”.

Transmission electron microscopy revealed that on “TS”, the appressoria formed penetration peg, infected vesicles and primary hyphae, and the primary hyphae further formed secondary hyphae (**Figure 3C**). The formation of penetration pegs started at 6 hpi on all grapevines. However, the formation ratio was significantly lower on “LB-8” compared to the other two germplasms, with nearly half of the appressoria on “LB-8” failing to produce penetration pegs. Despite a similar developmental trend across different germplasms, penetration pegs formed more rapidly on “TS” than on “B” and “LB-8” at each time point post-inoculation (**Figure 3D**). While “B” closely resembled “TS” in the penetration process, “LB-8” exhibited distinctive characteristics. “B” demonstrated a prolonged biotrophic stage compared to “TS”, with no observable secondary hyphae on “LB-8” (**Figure 3C**).

### 3.4 Expression of pathogenicity genes

**Figure 4:**
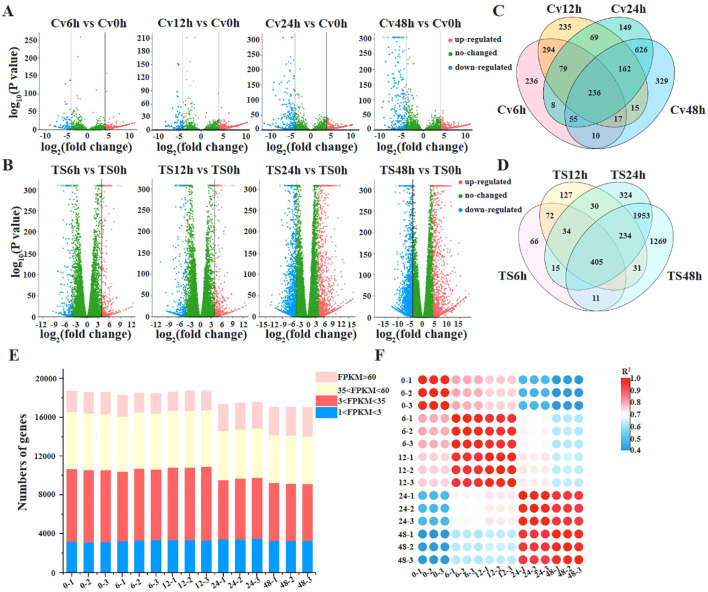
Transcriptome analyses of grapevines infected by *C. viniferum*. **(A, B)**Volcano plots displaying differentially expressed genes in *C. viniferum* and grapevine “TS”, respectively. Cv0h, Cv6h, Cv12h, Cv24h and Cv48h represent *C. viniferum* transcriptome, while TS0h, TS6h, TS12h, TS24h and TS48h represent “TS” transcriptome prepared from grape leaves post-inoculation with *C. viniferum* at 0, 6, 12, 24 and 48 hpi, respectively. **(C)**, Overlap of upregulated and downregulated DEGs in *C. viniferum*. **(D)**, Overlap of upregulated and downregulated DEGs in “TS”. **(E)**, Number of transcripts detected in 15 samples. Transcripts with an FPKM (fragments per kilobase of gene per million mapped reads) >1 are considered to be detected. Detected transcripts are further categorized as low (1<FPKM<3), moderated (3<FPKM<60), or high (FPKM>60) detection levels. **(F)**, Correlation of gene expression levels among grape leaves infected with *C. viniferum* at 0, 6, 12, 24, and 48 hpi.

To analyze the expression dynamics of *C. viniferum* pathogenic genes, we selected the susceptible grape “TS” characterized by rapid *C. viniferum* development for transcriptome analysis. A total of 95.29 Gb of sequence data was produced, resulting in approximately 42 million clean readings from each sample (**Figures 4E, F**). The clean reads were then mapped to the *C. viniferum* CvYL2a genome, with percentages ranging from 0.43% to 16.19% (**Supplementary Table 2**). The shared DEGs among the 6, 12, 24, and 48 hpi samples increased over infection period. Specifically, compared to 0 hpi samples, there were 852, 1029, 1115, and 1141 up-regulated DEGs (|log_2_ (fold change)| > 4, *P* < 0.05), and 83, 78, 269, and 309 down-regulated DEGs detected at 6, 12, 24, and 48 hpi, respectively (**Figure 4A**; **Supplementary Table 19–22**). In addition, there exhibited 236 CvYL2a DEGs consistent differential expression across all infection time points (**Figure 4C**).

**Figure 5:**
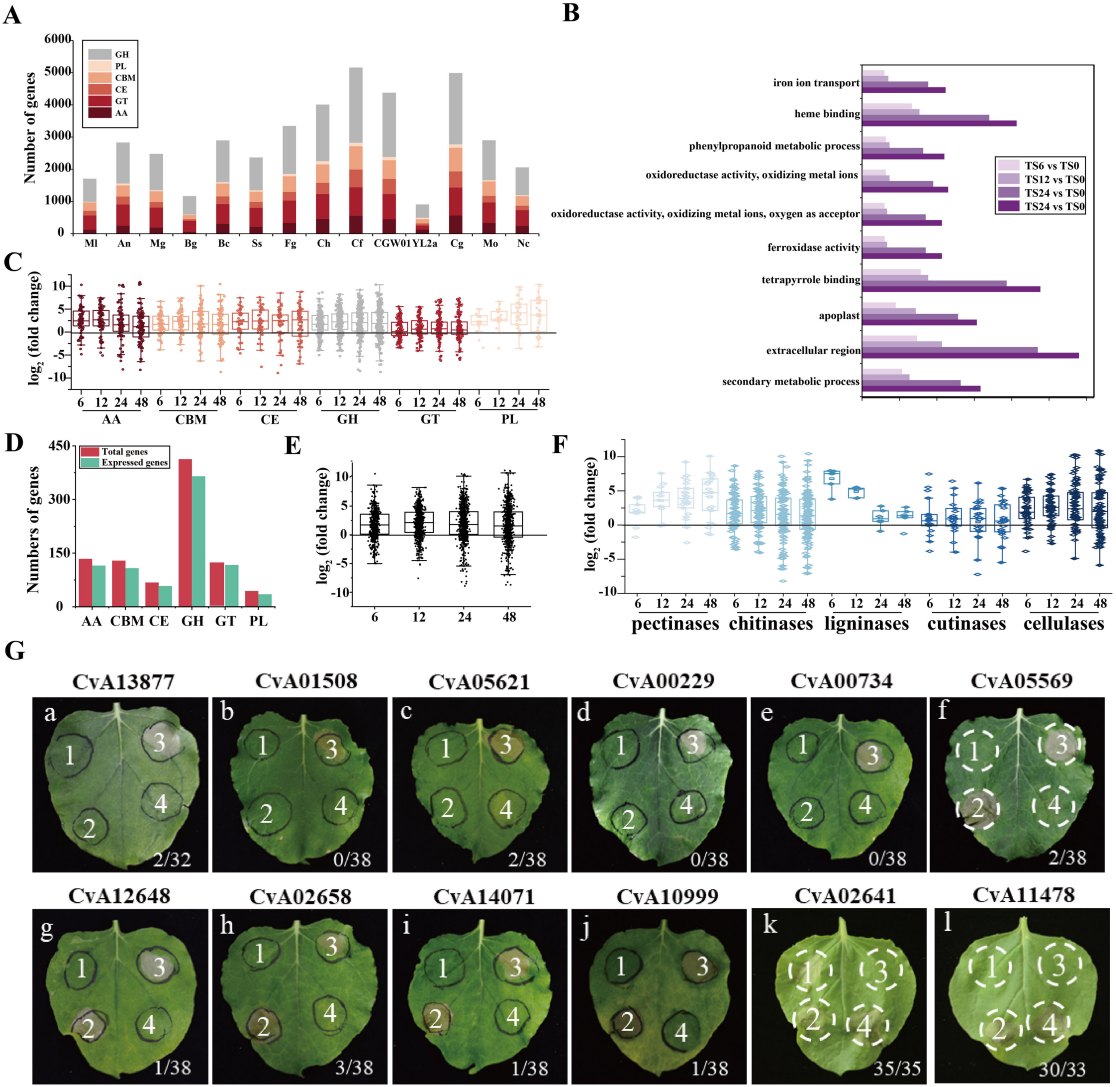
Gene Ontology and *C. viniferum* pathogenicity genes. **(A)** Repertoires of the carbohydrate-active enzymes (CAZymes) in *C. viniferum* and other 13 fungal genomes. GH, glycoside hydrolase; CE, carbohydrate esterase; AA, auxiliary activities; CBM, carbohydrate-binding module; PL, polysaccharide lyase. **(B)** Functional categorization of regulated differentially expressed genes (DEGs) in the *C. viniferum*-infected leaves of TS based on Gene Ontology (GO). *P*-value < 0.05. **(C)** Box plot of CAZymes gene expression of each family at 6, 12, 24, and 48 hpi. **(D)** CAZymes genes expressed post inoculation with *C. vinifera*. **(E)** Box plot displays expressed CAZymes (n = 530) in *C. viniferum* at 6, 12, 24, and 48 hpi. **(F)** Box plot of expressed CWDEs of *C. viniferum* in grape leaves at 6, 12, 24, and 48 hpi. **(G)** Cell death-suppression by INF1 and cell death-inducing activity of *C. viniferum* effectors on plant cell death induced. The label on the panel represents the name of the effector. a-j panels: the ability of 10 putative *C. viniferum* effectors to suppress cell death triggered by INF1. *A. tumefaciens* infiltration sites in *N. benthamiana* leaves expressing each of the 10 effectors were challenged with *A. tumefaciens* expressing the INF1 elicitin. *A. tumefaciens* strain carrying GFP was used as a control. Representative cell death symptoms were photographed at 7 days after INF1-GFP infiltration. k and l panels: 2 putative *C. viniferum* effectors CvA02641 and CvA11478 induced cell death. a-e panels received the following infiltration: 1, GFP; 2, effector-GFP; 3, GFP and INF1-GFP; 4, effector-GFP and INF1-GFP. f-j panels showed leaves infiltrated with 1, GFP; 2, INF1-GFP; 3, GFP and INF1-GFP; 4, effector-GFP and INF1-GFP. k and l panels were leaves infiltrated with 1, GFP; 2, effector-GFP; 3, GFP; 4, INF1-GFP. Ratios represent the proportion of infiltration sites displaying the cell death phenotype.

Transcriptome data was searched using SignalP, CAZy (Carbohydrate-Active Enzyme) database, AntiSMASH program, and the P450 database. All *C. viniferum* secondary metabolism gene clusters were shown in **Supplementary Table 10**. The transcriptome revealed significant differential expression of 56 effector genes, 36 Cazy genes, 5 P450 genes and 10 genes in secondary metabolism gene clusters at one or more infection stages (**Supplementary Table 5**). For further investigation into the functions of differentially expressed effectors, 20 cysteine-rich small effectors, 9 virulence-related effectors, and 28 effectors randomly chosen from the genome, along with 28 up-regulated effectors from both the genome and the transcriptome were selected (**Supplementary Table 6**) and assessed their abilities to induce or inhibit plant cell death. *Agrobacterium tumefaciens* carrying a GFP expression vector inserted with one of these 87 effectors was injected into *N. benthamiana* leaves. 12 h later, the hypersensitive response-inducing INF1 was injected at the same location. Among the effectors, ten inhibited INF1-induced cell death, while two induced cell death in *N. benthamiana* leaves. Subcellular localizations of 12 effectors in *N. benthamiana* were shown in **Supplementary Figure 2**. These findings suggest that these 12 effectors play active roles in pathogen-host interactions (**Figure 5G**).

We compared the CAZyme library across 14 fungal species and identified 909 CAZymes in the predicted proteome of *C. viniferum* CvYL2a. These CAZymes were distributed across six major CAZymes superfamilies, including 413 glycoside hydrolases (GHs), 121 glycosyl transferases (GTs), 44 polysaccharide lyases (PLs), 68 carbohydrate esterases (CEs), 134 auxiliary activities (AAs), and 129 carbohydrate binding modules (CBMs). Compared to other fungi, CvYL2a had fewer genes distributed in each superfamily. The expressed carbohydrate genes in CvYL2a during infection account for 87.79% of the total carbohydrate genes (**Figures 5C–E**). Within the CAZyme library, we identified 316 cell wall degrading enzymes (CWDEs), including 29 pectinases, 148 chitinases, 6 ligninases 26 cutinases, and 107 cellulases. Of these CWDEs, 273 (86.39%) genes were expressed during infection, with 103 being up-regulated and 21 down-regulated (**Figure 5F**; **Supplementary Table 9**).

### 3.5 Grapevine responses to *C.viniferum* infection

**Figure 6:**
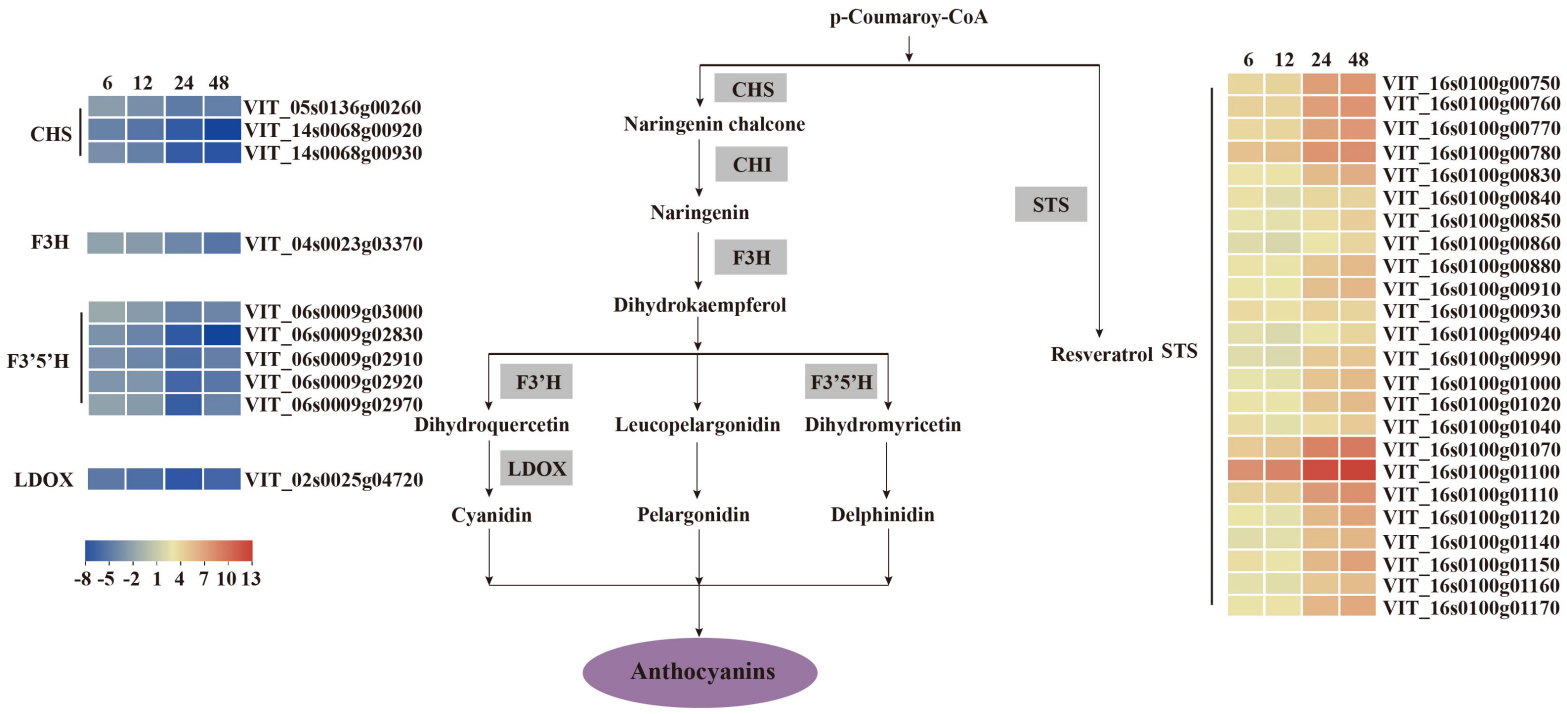
*V. vinifera* pathways and related gene expression for flavonoid biosynthesis in *C. viniferum*-infected leaves of *Vitis vinifera* cv. Thompson Seedless. Red indicates upregulation and blue indicates downregulation (*P* < 0.05) relative to the expression levels at 0 hpi. STS, stilbene synthase; CHS, chalcone synthase; CHI, chalcone isomerase; F3’H, flavonoid 3’ hydroxylase; F3’5’H, flavonoid 3’,10’-hydroxylase; F3H, flavanone 3-hydroxylase; LDOX, leucoanthocyanidin dioxygenase.

**Figure 7:**
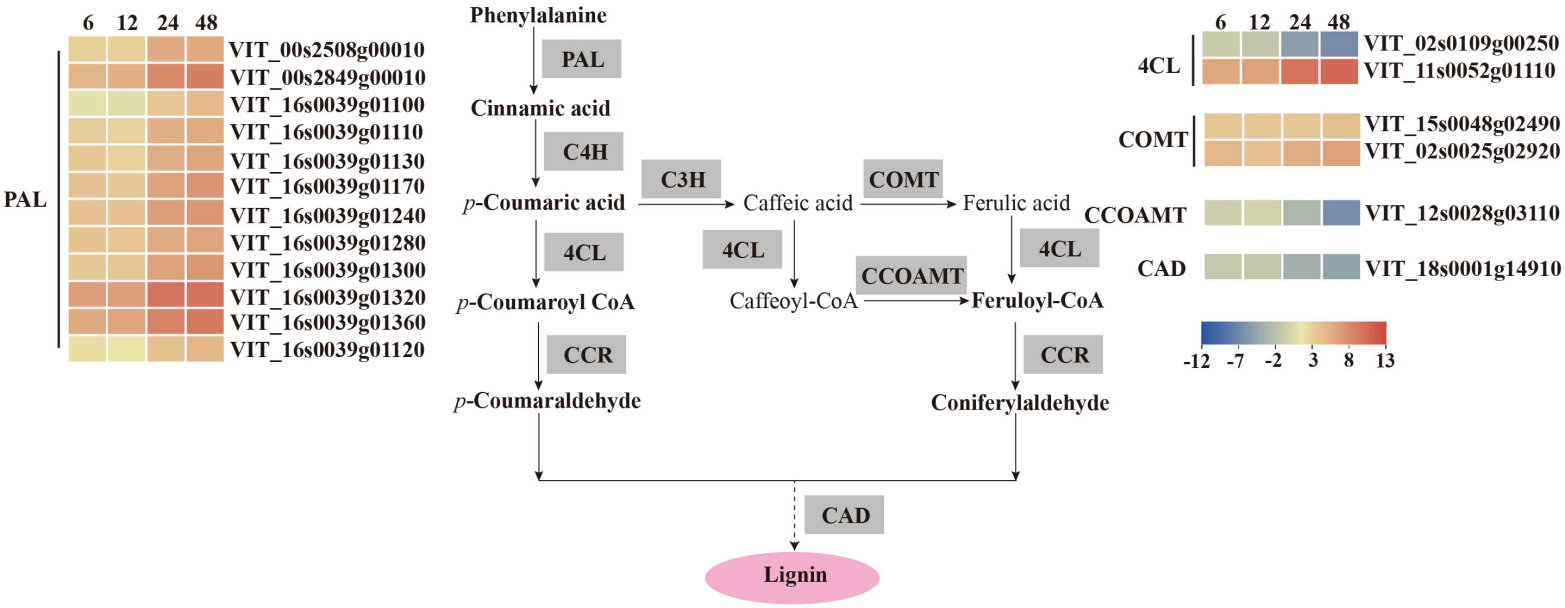
*V. vinifera* pathways and related gene expression for phenylpropanoid biosynthesis in *C. viniferum*-infected leaves of *Vitis vinifera* cv. Thompson Seedless. Red indicates upregulation and blue indicates downregulation, (*P* < 0.05) relative to the expression levels at 0 hpi. PAL, phenylalanine ammonia-lyase; C4H, cinnamate 4-hydroxylase; C3H, p-coumarate 3-hydroxylase; COMT, caffeic acid 3-O-methyltransferase; 4CL, 4 - coumarate -CoA ligase; CCOAMT, caffeoyl-CoA O-methyltransferase; CAD, cinnamyl alcohol dehydrogenase.

**Figure 8:**
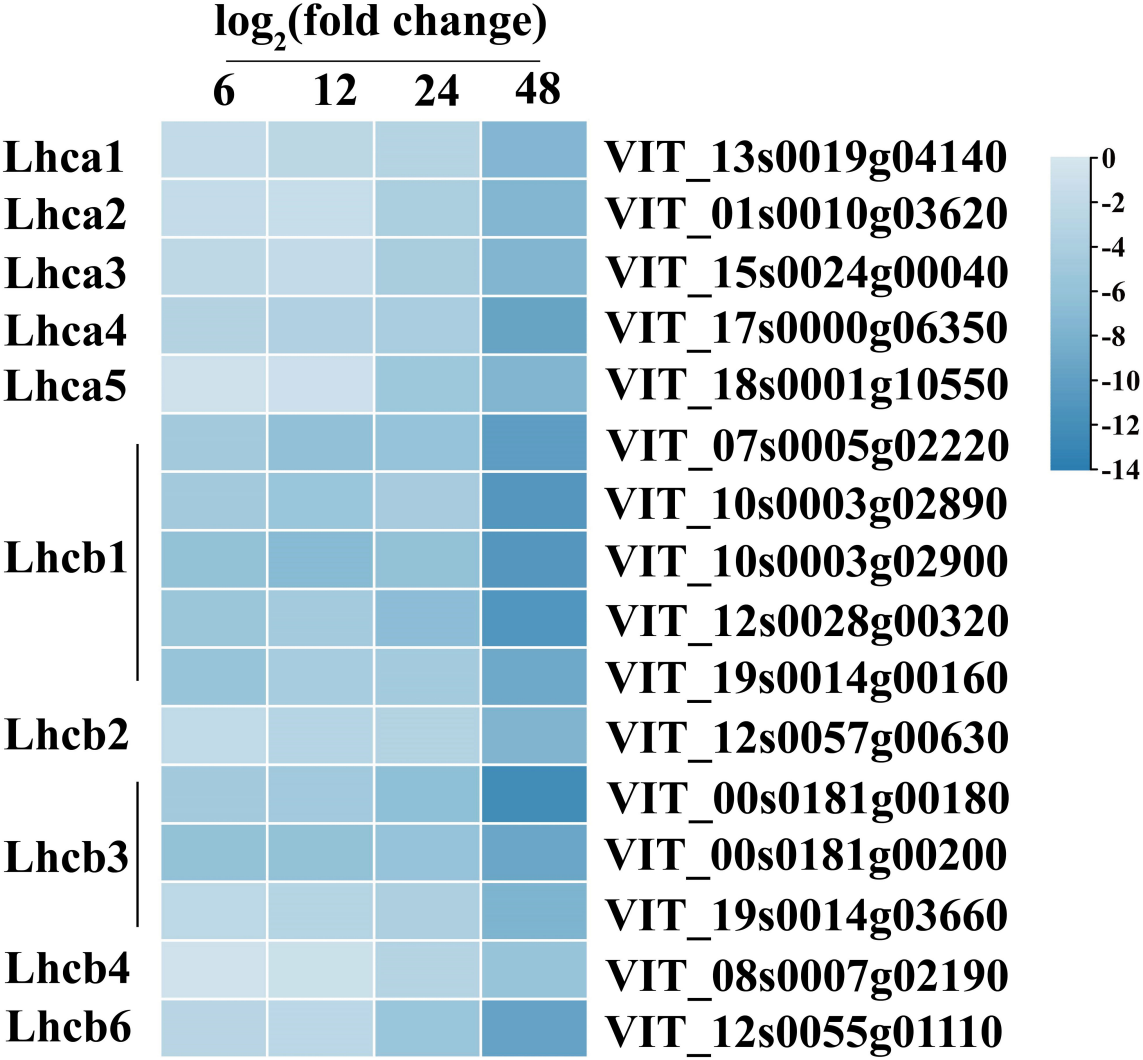
Gene expression of photosynthesis-antenna proteins in *C. viniferum*-infected leaves of *Vitis vinifera* cv. Thompson Seedless. Blue indicate downregulation, respectively; and unshaded represents no significant change (*P* < 0.05) relative to the expression levels at 0 hpi. Lhca, chlorophyll a binding protein of Light-harvesting chlorophyII; Lhcb, chlorophyll b binding protein of Light-harvesting chlorophyII.

To study the host response to *C. viniferum*, clean reads also were mapped to the *V. vinifera* PN40024 genome, with mapping percentages of 93.90 ± 0.06%, 93.82 ± 0.06%, 93.67 ± 0.12%, 91.59 ± 0.20%, and 77.90 ± 0.53%, respectively (**Supplementary Table 3**). In the *V. vinifera* transcriptome, we detected 422, 570, 1359, and 1503 up-regulated DEGs along with 261, 377, 1702, and 2480 down-regulated DEGs. Moreover, 405 common DEGs were identified throughout the infection process (**Figures 4B, D**). DEGs functional annotations with different time point were shown in **Supplementary Tables 11–14**. GO terms at different time point were analyzed (**Supplementary Tables 15–18**). Gene ontology analysis revealed their involvement in biological processes like iron ion transport, phenylpropane metabolism and secondary metabolism, cell components like apoplast and extracellular domain; and molecular functions including heme binding, tetrapyrrole binding and oxidoreductase activity (**Figure 5B**). Additionally, KEGG pathway enrichment analysis showed significant enrichment in phenylpropanoid-guided flavonoid and resveratrol biosynthesis (34 DEGs), phenylpropanoid biosynthesis (18 DEGs), photosynthesis antenna proteins (16 DEGs) (**Figures 6**–**8**).

Our analysis demonstrated distinct patterns of gene expression in several pathways, including phenylpropanoid-guided flavonoid and resveratrol biosynthesis, phenylpropanoid biosynthesis, and photosynthesis-antenna proteins. Notably, RNA-seq analysis showed downregulation of genes associated with anthocyanins synthesis, including flavonoid 3’,10’-hydroxylase (*F3’5’H*), flavanone 3-hydroxylase (*F3H*), chalcone synthase (*CHS*), and leucoanthocyanidin dioxygenase (*LDOX*). Conversely, the stilbene synthase (*STS*) gene associated with the synthesis of resveratrol was significantly upregulated (**Figure 6**). In the phenylpropanoid biosynthesis pathway, while caffeoyl-CoA O-methyltransferase (*CCOAMT*) and phenylalanine ammonia-lyase (*PAL*) were upregulated, cinnamyl alcohol dehydrogenase (*CAD*), a gene related to lignin synthesis, was downregulated (**Figure 7**). Grape genes involved in the photosynthesis-associated pathway encoded light-harvesting chlorophyll a/b-binding proteins (Lhc). The Lhc superfamily contains eight subfamilies: early light-induced protein (ELIP), ferrochelatase II (FCII), Lhca, Lhcb, one-helix protein (OHP), photosystem II subunit S (PsbS), photosystem II protein 33 (Psb33), and stress-enhanced protein (SEP) (Klimmek et al., 2006). All differentially expressed photosynthesis antenna genes including 5 *Lhca* and 11 *Lhcb* were downregulated in susceptible *V. vinifera* “TS” (**Figure 8**).

**Figure 9:**
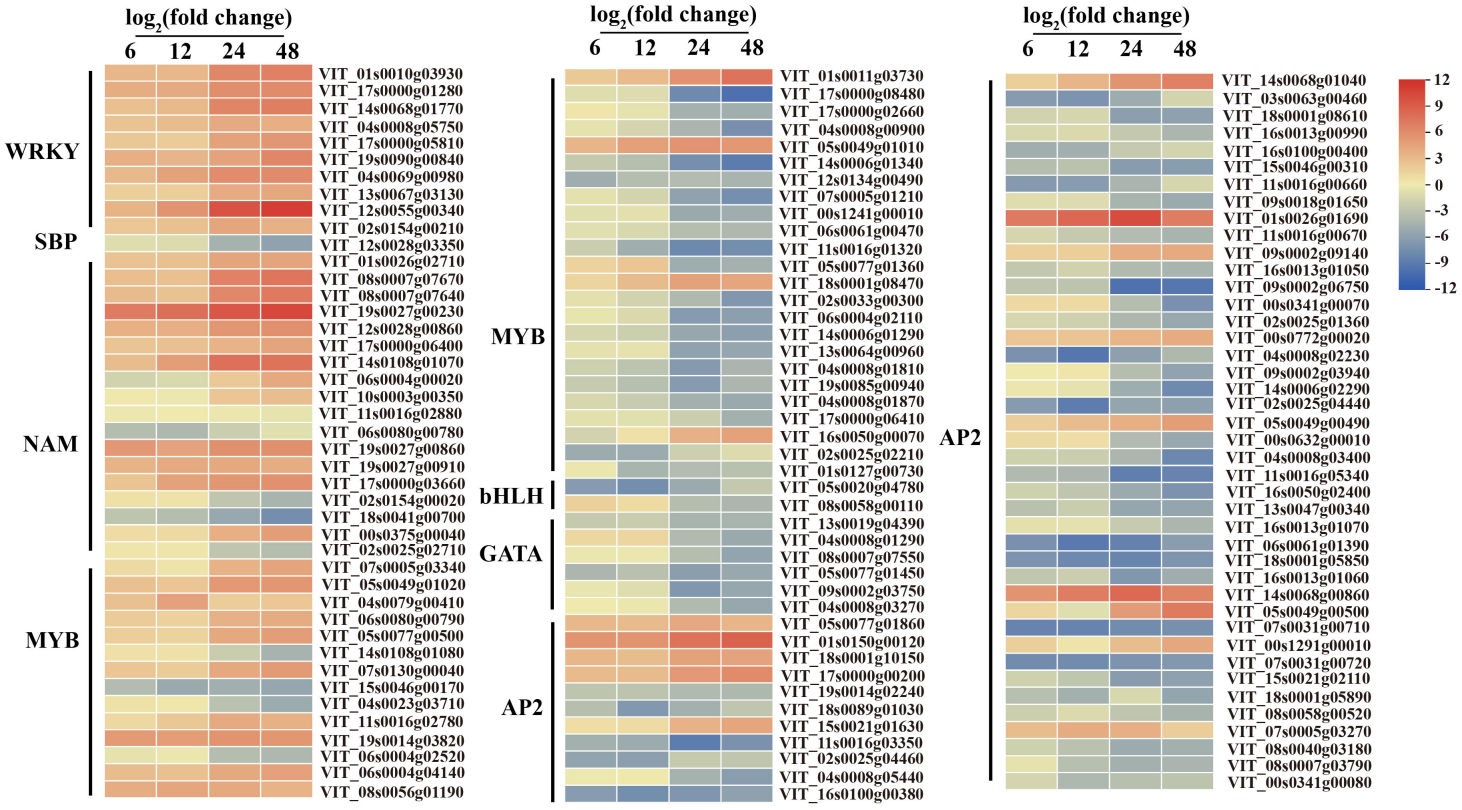
Expression patterns of differentially expressed transcription factors at 6, 12, 24, and 48 hpi in *C. viniferum*-infected *V. vinifera* leaves. WRKY, WRKY domain-containing transcription factors, SBP, quamosa promoter promoter binding proteins, NAM, No apical meristem, MYB, v-myb avian myeloblastosis viral oncogene homolog, bHLH, basic helix-loop-helix proteins, GATA, GATA-binding transcription factors, AP2, APETALA2 responsive factor. Red and blue indicate upregulation and downregulation, respectively, and yellow represents no significant change (*P* < 0.05) relative to the expression levels at 0 hpi.

**Figure 10:**
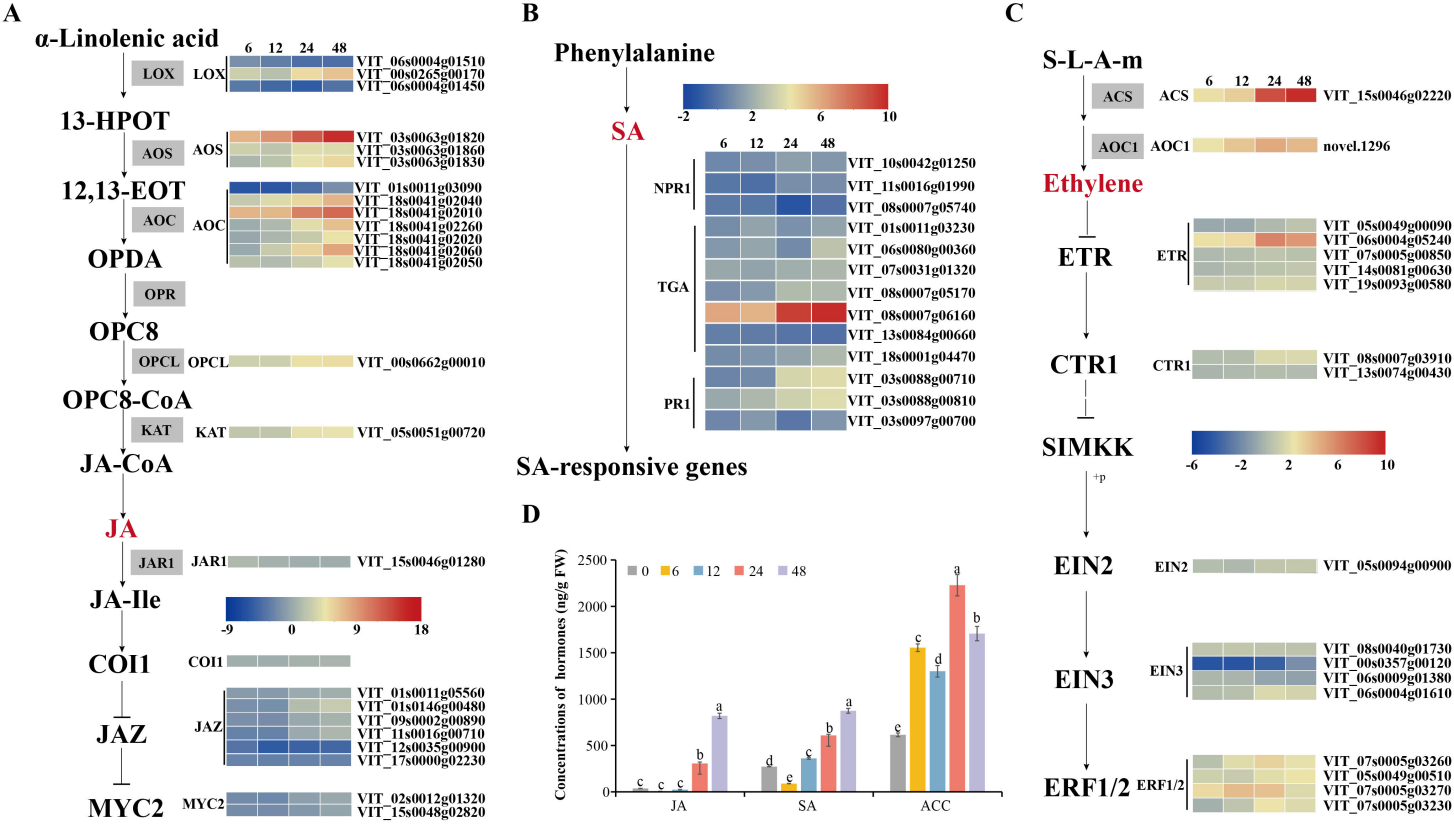
Hormonal content of grape leaves during infection and expression patterns of differentially expressed genes assigned to hormone signaling. **(A–C)** V. vinifera jasmonates (JA) biosynthesis, salicylic acid (SA) and ethylene (ET) pathways and related gene expression in C. viniferum-infected leaves at 6, 12, 24, and 48 hpi. Red and blue indicate upregulation and downregulation (P < 0.05), respectively, relative to the expression levels at 0 hpi. 12,13-EOT: 12,13(S)-epoxylinolenic acid; 13-HPOT: 13(S)-hydroperoxylinolenic acid; AOS: allene oxide synthase; LOX: lipoxygenase; AOC: allene oxide cyclase; OPR: 12-oxophytodienoate reductase; OPC:8, 3-oxo-2-(cis-29-pentenyl)-cyclopentane-1-octanoic acid; OPCL: 3-oxo-2-(cis-29-pentenyl)-cyclopentane-1-octanoic acid CoA Ligase; OPDA: 12-oxocis-10,15-phytodienoic acid; COI1, coronatine-insensitive protein 1; JAZ, jasmonate ZIM domain-containing protein; MYC2, transcription factor MYC2; ACS: 1-aminocyclopropane-1-carboxylate synthase 3; ACO1: 1-aminocyclopropane-1-carboxylate oxidase; ETR: ethylene receptor; CTR1: serine/threonine-protein kinase CTR1; EIN2: ethylene-insensitive protein 2; EIN3: ethylene-insensitive protein 3; EBF1/2: EIN3-binding F-box protein; ERF: ethylene-responsive transcription factor; NPR1: nonexpressor of pathogenesis-related genes 1; TGA: transcription factor TGA; PR1: pathogenesis-related protein 1. **(D)** Hormonal content of grape leaves in C. viniferum-infected leaves at 0, 6, 12, 24, and 48 hpi. Distinct letters indicate significant differences (ANOVA test with Tukey’s comparisons, P < 0.05). ACC: 1-aminocyclopropane-1-carboxylic acid, precursor of ethylene.

Plant disease resistance is significantly influenced by the involvement of transcription factors. Recent research has highlighted the involvement of various transcription factor families in regulating signal transduction in plant SA, ET and JA pathways responding to different pathogen infections, such as WRKY (WRKY domain-containing transcription factors) family, MYB (v-myb avian myeloblastosis viral oncogene homolog) family, and ERF (thylene responsive factors) family (Liu et al., 2021; Gao et al., 2022; Zhou et al., 2022). Our study found that numerous transcription factors, including 10 WRKYs, 18 NACs (NAM, ATAF1/2, and CUC2), 38 MYBs, 53 ERFs, 6 GATAs (GATA-binding transcription factors), 2 bHLHs (basic helix-loop-helix proteins), one SBP (quamosa promoter promoter binding proteins) were differentially expressed during *C.viniferum* infection at four stages (**Figure 9**). Gene expression associated with JA, SA, and ET signaling pathways significantly increased upon *C. viniferum* infection (**Figure 10**). Our results showed that the JA synthesis-related genes including *LOX* (VIT_00s0265g00170), AOS (VIT_03s0063g01820, VIT03s0063g01860 and VIT03s0063g01830), *AOC* (VIT_01s0011g03090, VIT_18s0041g02040, VIT_18s0041g02010, VIT_18s0041g02260, VIT_18s0041g02020, VIT_18s0041g02060, VIT_18s0041g02050), *OPCL* (VIT_00s0662g00010), and KAT (VIT_05s0051g00720), as well as the SA-synthesis-related genes *PAL* (VIT_16s0039g01170, VIT_00s2508g00010, VIT_16s0039g01280, VIT_00s2849g00010, VIT_16s0039g01100, VIT_16s0039g01300, VIT_16s0039g01110, VIT_16s0039g01130, VIT_16s0039g01240, VIT_16s0039g01320, VIT_16s0039g01120 and VIT_16s0039g01360) and *ACS* (VIT_15s0046g02220), a new transcript *AOC1* (novel.1296) for ethylene synthesis were upregulated during *C. viniferum* infection of grape leaves (**Figure 10**). We measured the hormones levels of *C. viniferum* infection and found that SA, JA, and ET synthesis precursor ACC significantly increased (**Figure 10**). The findings suggest the involvement of JA, SA, ET and ABA in regulating the response to *C. viniferum* during the late stage of infection.

**Figure 11:**
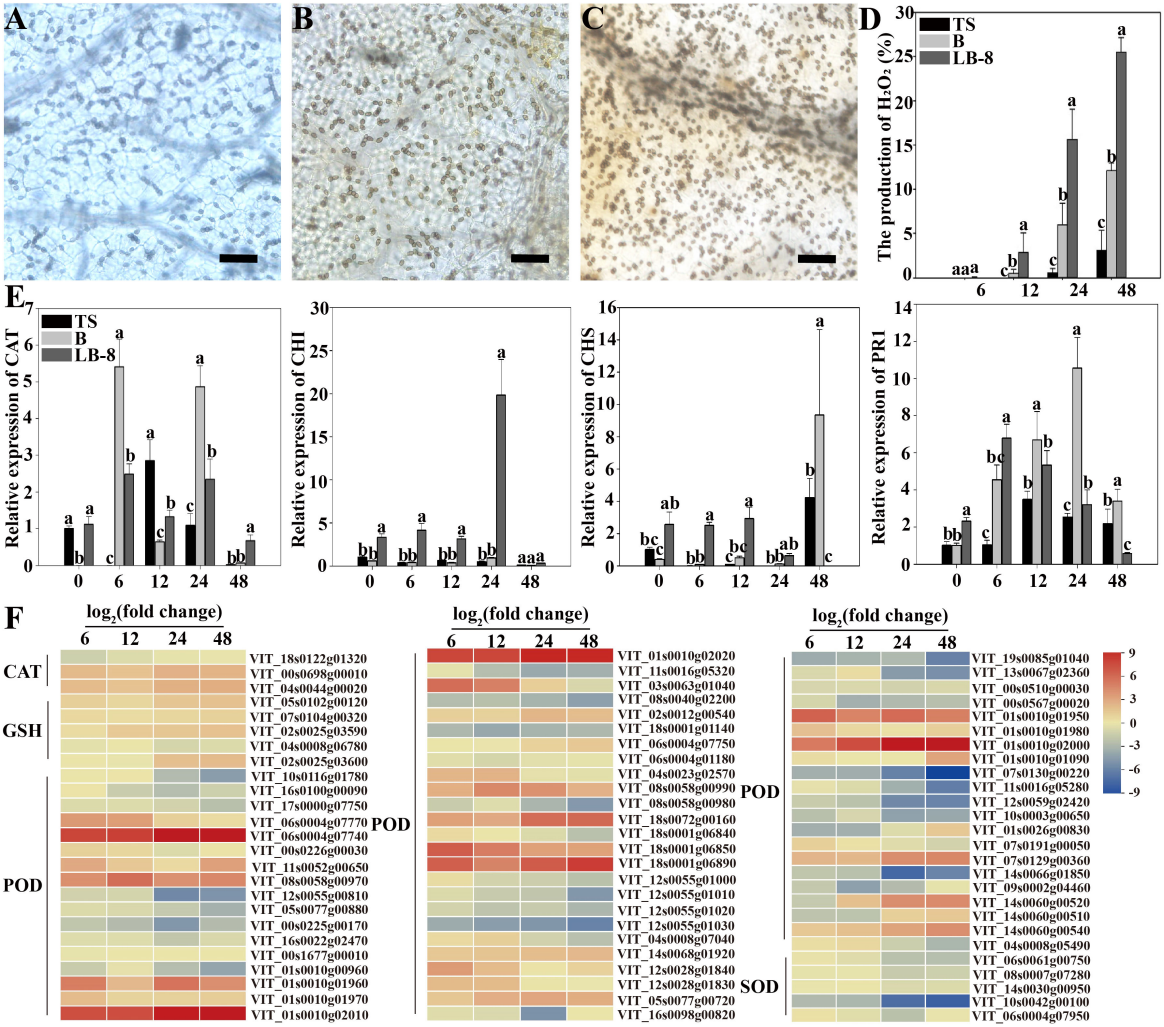
Responses of grapevines to *C viniferum* CvYL2a. **(A-C)** 2-day DAB staining of leaves from three grape varieties: **(A)** TS, **(B)** B, and **(C)** LB-8 inoculated with *C. viniferum* CvYL2a. Scal bar = 25 μm. **(D)** The production rate of H_2_O_2_ after inoculation of leaves from three grape varieties with *C. viniferum* CvYL2a, results are presented as mean ± SE, n = 9. Different letters indicate significant differences (ANOVA test with Tukey’s comparisons, *P* < 0.05). **(E)** Quantitative analysis of defense-related genes in “TS”, “B”, and “LB-8” plants after inoculation with *C. viniferum*. The gene expression levels were analyzed using quantitative real-time polymerase chain reaction (RT-qPCR). The natural logarithm transformed normalized-expression values were plotted for each gene at five time points (0, 6, 12, 24 and 48 hpi). Results are presented as mean ± SE, n = 3. Different letters indicate significant differences (ANOVA test with Tukey’s comparisons, *P* < 0.05). CAT, Catalase. CHI, Chalcone isomerase; CHS, Chalcone synthase; PR1, pathogenesis-related 1. **(F)** DEGs associated with reactive oxygen species (ROS) scavenging in *C viniferum*-infected “TS”. CAT, Catalase; GSH, Glutathione peroxidase; POD, Peroxidase; SOD, Superoxide dismutase.

To measure the production and accumulation of hydrogen peroxide (H_2_O_2_), we used diaminobenzidine (DAB) staining, which relies on the peroxidase-catalyzed oxidation of oxygen released by H_2_O_2_, resulting in tan deposits on the plant surface. Fewer cells produced H_2_O_2_ in “TS” leaves after *C. viniferum* inoculation. However, “B” and “LB-8” exhibited obvious browning due to DAB oxidation. “B” showed lighter staining and fewer stained cells after *C. viniferum* inoculation, while “LB-8” displayed the opposite pattern. At 6 hpi, none of the three grape leaves significantly produced H_2_O_2_. However, by 12 hpi, cells beneath the appressorium in “LB-8” exhibited tan staining, with a significant increase observed from 24 hpi to 48 hpi. In “B” leaves, the proportion increased from 6.00% to 12.12%, while “LB-8” reached 25.53%. “TS” leaves showed slight staining only at 48 hpi. These results suggest that *C. viniferum* invasion is more extensive during the initial 2 days after inoculation, with “TS” leaves exhibiting the lowest H_2_O_2_ production rate (**Figures 11A–D**). We identified 76 DEGs involved in antioxidation during *C. viniferum* infection in grapes. Among these, one-third (22) of the peroxidase (POD), and four-fifths of glutathione peroxidase (GSH) related DEGs were up-regulated. Furthermore, three catalase (CAT) DEGs showed increased expression, and one was down-regulated. These findings underscore the significant role of ROS scavenging by POD, CAT and GSH in the grapevine responses to *C. viniferum* (**Figure 11F**).

One notable transcriptomic characteristic observed in grape leaves following *C. viniferum* infection is the up-regulation of defense-related genes. To further substantiate our transcriptome sequencing results, we performed qRT-PCR analysis of four defense-related genes at different time points post inoculation with *C. viniferum*. Two genes, CAT (Catalase) and PR1 (pathogenesis-related 1), showed higher expression levels at 6 hpi in “B” grape leaves. In “TS”, the expression levels of defense-related genes increased slowly. In contrast, two other genes CHI (Chalcone isomerase) and CHS (Chalcone synthase) displayed rapid upregulation during infection in “LB-8” compared to “B” and “TS” (**Figure 11E**). Additionally, among 72 identified LRR (Leucine-rich repeat receptor) proteins in infected grape leaves, 23 were up-regulated. Two disease-resistant proteins (DRP) were detected, RPS2 (VIT_11s0016g01860) was upregulated and DRP (VIT_01s0026g01420) was down-regulated. Among two glutathione S-transferase (GST) genes, VIT_12s0028g00920 was up-regulated, whereas VIT_04s0079g00690 was down-regulated (**Supplementary Table 8**). These genes collectively play an important role in disease resistance responses.

## 4 Discussion

*Colletotrichum* spp. are highly destructive fungal pathogens to various plants, such as grapes, peppers, and lentils (Lei et al., 2016; Bhadauria et al., 2019; Huo et al., 2021). They can invade various parts of plants, including leaves, stems, shoots, tendrils, and berries Wang et al., 2021). Among these pathogens, *C. viniferum* poses a particularly serious risk to grape production. Previous research has demonstrated varying degrees of grapevine resistance to *C. viniferum*, indicating that different plant materials exhibit distinct reactions towards the same pathogen (Baroncelli et al., 2014; Echeverrigaray et al., 2020).

Histological observation is an effective method for studying the differences in cytology and ultrastructure of grapes infected with different germplasm resources by *C. viniferum*. Transcriptome analysis has become a common approach to studying pathogenic infection hosts. In this study, these two methods were used to reveal the interaction between grape and *C. viniferum*, providing a basis for the selection of candidate fungal pathogenic genes including CAZymes, effectors, P450 and grapevine response genes including plant hormone signaling, metabolite synthesis, expression of transcription factors, ROS clear genes and disease-associated genes.

### 4.1 *C. viniferum* developed rapidly on susceptible grapes

Current studies on *Colletotrichum* spp. in grapes have mainly focused on evolutionary and taxonomic aspects, with limited histological examination of the infection process of *Colletotrichum* spp., especially *C. viniferum* (Whitelaw-Weckert et al., 2010; Yan et al., 2015). Observations of *Colletotrichum* spp. on grape morphology, including conidia, appressorium, and hyphae, remain relatively scarce (Melksham et al., 2002; Peng et al., 2013; Oo and Oh, 2017). To evaluate the variance in *C. viniferum* infection on the differences of grapevines, the histopathology of the leaves of high-susceptible grape germplasm “TS”, medium-resistant grape germplasm “B”, and resistant grape germplasm “LB-8” were observed by transmission electron microscopy and scanning electron microscopy. By comparing the resistance levels of these three germplasm grapes to *C. viniferum*, the differences between susceptible grapes and resistant grapevine germplasm were studied. The results of this study can provide a theoretical basis for the later study of the pathogenesis of *C. viniferum*.

Our study revealed significant differences in the response of susceptible and resistant grapevines to *C. viniferum* infection. One notable distinction was the fast disintegration of upper epidermal cells in highly susceptible grapes compared to medium-resistant and resistant grapes. Additionally, visible damage on the leaf surface formed earlier in the susceptible grapevine (**Figures 2**, **3A**). After *C. viniferum* infection, white secretions appeared on the leaves of “B” and “LB-8”, while “TS” did not (**Figure 3B**), presumably a response, similar to previous studies on downy mildew infection of resistant grapes, which showed that “Langao-5” and “Liuba-8” resistant grape germplasms rapidly produced white secretion to limit downy mildew in the early stage of inoculation (Yin et al., 2017). We observed variations in the formation rate of appressoria and penetration pegs among different grapes. “TS” exhibited faster formation of appressoria and penetration pegs compared to “B” and “LB-8”. This pattern is consistent with findings in other pathosystems, such as *C. acutatum*-almond pathosystem (Diéguez-Uribeondo et al., 2005). Interestingly, “B” and “LB-8” displayed more than 30% malformed appressoria, which we attribute to two factors. Firstly, the secretion from resistant grapes “B” and “LB-8” may interfere with appressorium formation (**Figure 3B**), a response observed in grapevines against downy mildew (Yu et al., 2012; Yin et al., 2017). Secondly, the dense hair on grapevine leaves may have compressed and deformed appressoria (**Figure 3C**), acting as a primary barrier against fungal infection (Kortekamp and Zyprian, 1999).

*Colletotrichum* spp. infections typically undergo a biotrophic phase in the early stage, followed by a necrotrophic stage after the emergence of secondary hyphae (Shang et al., 2020; Li et al., 2021a). The period of the brief biotrophic stage of *Colletotrichum* spp. infection varied by host and other factors. For instance, when *C. fructicola* infects apple leaves, its short biotrophic stage continues for at least 36 hpi, and then it transforms into the necrotrophic stage at 48 hpi (Shang et al., 2020). Similarly, our observations indicate variations in the duration of the biotrophic phase among grape germplasm resources. For “TS”, the brief biotrophic phase lasted 24 h before transitioning to the necrotrophic phase by the emergence of secondary hyphae and the disintegration of epidermal cells. However, “B” exhibited a longer biotrophic stage than “TS” before progressing to the necrotrophic stage. Notably, secondary hyphae were not seen on “LB-8”, indicating that *C. viniferum* infection on the resistant grape “LB-8” maintains in the biotrophic stage (**Figure 3C**).

### 4.2 *C. viniferum* pathogenicity genes may play an important role in infection

Transcriptome sequencing offers a direct way to explore gene expression levels, and numerous studies have investigated transcriptome sequencing of *Colletotrichum* spp.-infected hosts, encompassing both fungal and host transcriptomes. Transcriptome analyses of *Colletotrichum* spp. includes *C. fructicola* on apples (Liang et al., 2018), *C. falcatum* on sugarcane (Prasanth et al., 2022), and *C. camelliae* on tea (He et al., 2023). However, the transcriptome of *C. viniferum* on grapes has not been studied. To study the pathogenic mechanism of *C. viniferum*, we conducted transcriptome analysis using the susceptible grape “TS”. Through transcriptome analysis, we identified a range of differentially expressed pathogenic factors, including 35 CAZymes, 56 effectors, and 5 P450 genes. Secondary metabolites are related to pathogenicity of fungi during infection. We also identified 67 secondary metabolic gene clusters, and only 10 genes (A01092, A01212, A01323, A07559, A08496, A11116, A13125, A13129, A13635, A13736) distributed in 9 secondary metabolite clusters (Cluster2, 21, 25, 26, 32, 33, 35, 57 and 65) had significant differences in expression levels during infection, these 10 genes may play core roles, and the remaining other genes on these 9 clusters may play accessory roles in fungal pathogenicity. (**Supplementary Tables 4**, **10**).

The numbers of major CAZyme superfamilies and CAZymes in CvYL2a were lower than those in other fungi (**Figure 5A**). Some of these enzymes were responsible for plant cell wall degradation, such as pectinase and cellulase. During *C. viniferum* infection of “TS”, the expression levels of these genes significantly increased, indicating their important roles in grape infection (**Figure 5F**). Furthermore, fungal and oomycete effectors are known as pivotal pathogenic factors that modulate plant immunity and affect disease development (Nie et al., 2019; Chen et al., 2021; Yin et al., 2022). In this study, two effectors induced HR responses in plants, potentially linked to plant resistance R genes in plants. Moreover, ten effectors were found to inhibit INF1-induced cell death, indicating their ability to evade immune recognition by grapevines and promote pathogen infection (**Figure 5G**).

### 4.3 Grape responses to *C. viniferum* include plant hormone signaling, metabolite synthesis, expression of transcription factors, ROS clear genes, and disease-associated genes

To explore grapevine responses to *C. viniferum*, we analyzed differentially expressed grapes genes involved in plant hormone signaling and metabolite synthesis, transcription factors, ROS clearance genes, and disease-associated genes. Plant hormones are important in plant responses to pathogens (Zhang et al., 2017; Li et al., 2019a). In this study, the level of SA increased at 6-12 hpi, JA increased at 24-48 hpi, and ACC, synthetic precursor of ET, increased at 12-24 hpi (**Figure 10D**). Our experiment indicated that the early stage of infection corresponds to the biotrophic stage, while the later stage corresponds to the necrotrophic stage. This is consistent with previous findings that SA-dependent responses are associated with biotrophic organisms, while ET and JA-dependent responses are associated with necrotic organisms (Glazebrook, 2005). The expression levels of hormone synthesis-related genes, downstream transcription factors (*MYC*, *ERF*, and *TGA*), and disease-related genes (*PR1*) were upregulated in the transcriptome. This finding suggests that these hormones may activate downstream transcription factors and disease-resistant genes. Hormonal networks that regulate downstream gene expression during plant-pathogen interactions have long been discovered. For example, SA activates TGA and PR1 (Shimizu et al., 2022), JA activates MYC (Schmiesing et al., 2016), and ethylene activates ERF (Broekaert et al., 2006). Additionally, other differentially expressed transcription factors (WRKY, SBP, NAM, MYB, and GATA) may also play critical roles in plant disease resistance and warrant further experimental studies (**Figure 9**). We hypothesize that the induction of JA, ET, and SA signaling pathways, along with the activation of transcription factors, are essential components of the grape response to *C. viniferum*.

Phenylpropanoid-guided flavonoid and resveratrol biosynthesis pathways, phenylpropanoid biosynthesis pathways, and photosynthetic antenna proteins have been implicated in plant resistance to pathogens (Jantasuriyarat et al., 2005; Manickavelu et al., 2010; Nabavi et al., 2020; Huang et al., 2021). Transcriptome analysis revealed upregulation of resveratrol synthesis genes during *C. viniferum* infection (**Figure 6**). Resveratrol is important in the fight against pathogen infections (Vestergaard and Ingmer, 2019; Zhou et al., 2024). This suggests a coordinated response wherein plants synthesize more resveratrol to resist *C. viniferum* infection. In phenylpropanoid biosynthesis, the downregulation of CAD genes, a key enzyme in lignin synthesis, was observed. CAD7, a negative regulator of plant immunity targeted by Avr3a effectors, promotes pathogen infection by inhibiting plant PAMP-triggered immunity (Li et al., 2019b). This raises the possibility that CAD could serve as a target protein for fungal effectors, leading to the loss of lignin synthesis activity and further promoting pathogen infection (**Figure 7**). Conversely, all differentially expressed photosynthetic antenna proteins were downregulated. Lhca/b-binding protein in the photosynthetic pathway is one of the most abundant proteins in plant chloroplasts. Genes encoding the Lhca/b protein have been identified in interaction between susceptible plants and pathogens (Jantasuriyarat et al., 2005; Manickavelu et al., 2010). The process of inducing plant response systems requires a lot of energy, which increases the need for photosynthesis in plants (Swarbrick et al., 2006). However, this study showed down regulation of Lhca and Lhcb during infection (**Figure 8**). Similarly, previous experiments have shown that tomato seedlings inoculated with DC3000 were also downregulated the expression of photosynthesis-related genes (Ishiga et al., 2009). This suggests that plants may limit the carbon sources available to pathogens or protect plant cells from oxidative damage by downregulating photosynthesis.

Earlier research has highlighted the diverse roles of plant response genes, such as chalcone synthase, pathogenesis-related proteins, catalase, and chitinase, in conferring resistance or tolerance to fungal pathogens (Nandeeshkumar et al., 2008; Jaulneau et al., 2010; Feng et al., 2011). In this study, we observed an earlier response in plants “LB-8” and “B” compared to “TS”, characterized by higher expression levels of response genes (**Figure 11E**). Another notable difference is the accumulation of reactive oxygen species. Hydrogen peroxide increases significantly during host-pathogen interactions (Wang et al., 2010). Our results suggest that plant ROS clearance genes are upregulated during “TS” infection (**Figure 11F**), consistent with previous findings on pathogenic infections (Segal and Wilson, 2018). Peroxidase activity in grapes during *C. viniferum* infection may contribute to lignin breakdown and ROS scavenging (**Figure 7**). The most studied function of LRR protein in plants is to play an important role in disease resistance. LRR-encoding genes act as pattern recognition receptors (PRRs) to sense pathogen-associated molecular patterns (PAMPs) and as R genes involved in the immune responses of plants (Kunze et al., 2004; Meyers et al., 2005). Additionally, genes associated with grape response, including LRR, disease resistance genes, and glutathione S-transferase, were upregulated (**Supplementary Table 6**). Despite the elevated expression levels of these genes, susceptible grapevines remain infected with *C. viniferum* and develop lesions. This phenomenon parallels observations in susceptible grapevines *V. vinifera* cv. Red globe infected with *Elsinoë ampelina* (Li et al., 2021c).

**Figure 12:**
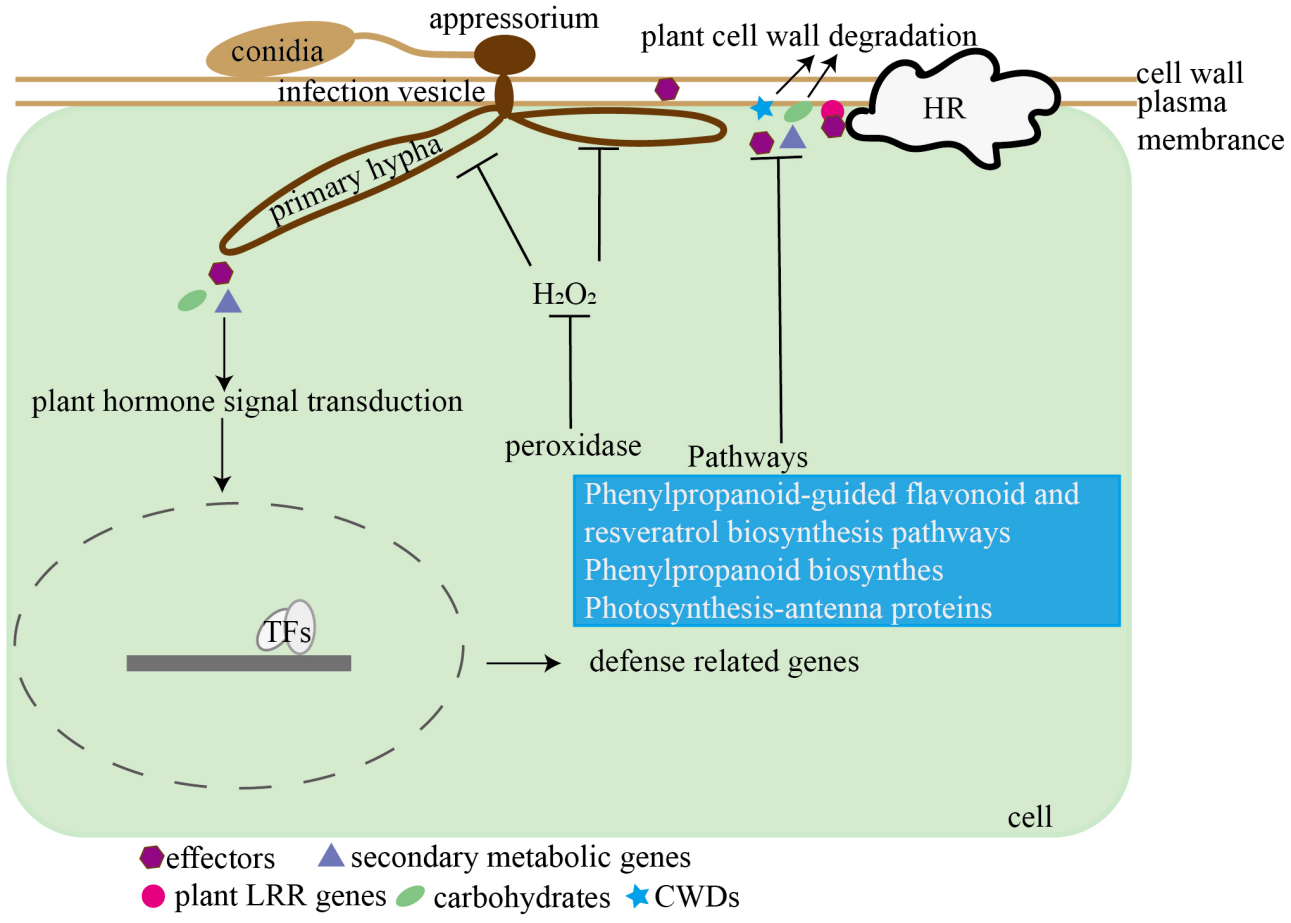
Hypothetical model of the interaction between *C. viniferum* and *V. vinifera*. *C. viniferum* infects grapevines through pathogenic genes including effectors, carbohydrates, secondary metabolites, and plant cell wall degradation enzymes, while grapes inhibit *C. viniferum* infection by LRRs recognizing pathogenic fungi and activating ROS accumulation.

Currently, only three grape transcriptome datasets related to *Colletotrichum* spp. infection are available (Lei et al., 2022; Shen et al., 2022; Yu et al., 2023). However, these three studies exclusively analyzed grape transcripts, leaving the changes in both *Colletotrichum* and grape transcriptomes during the infection process remain unexplored. Therefore, our research employed simultaneous transcriptome analyses to examine fungal genes associated with pathogenesis and grape genes associated with defense responses. We proposed a model depicting the interaction between grape and *C. viniferum* (**Figure 12**), providing a basis for further research on the pathogenesis of *C. viniferum* and the interaction between grapevines and *C. viniferum*.

## 5 Conclusion

In summary, our comparative analysis of susceptible and resistant grapevines through light and electron microscopy revealed distinct differences in infection dynamics. Notably, the diameter of *C. viniferum* lesions was the largest on “TS”, followed by “B”, and smallest on “LB-8”. Susceptible grapes exhibited no white secretion, normal appressoria shape, and the formation of secondary hyphae, facilitating *C. viniferum* infection on grapes. We propose a model illustrating the interaction between grapes and *C. viniferum* (**Figure 12**). *C. viniferum* employs a variety of virulence strategies, including effectors, CWDEs, and secondary metabolites to target various cellular processes for nutrient acquisition. Conversely, grapevine responses to *C. viniferum* involves hormone signaling, disease-related genes expression, metabolic pathways activation, ROS accumulation, and transcription factors regulation. Our findings hold significance for the selection of candidate fungal pathogenic genes and plant disease response genes. Furthermore, they laid the groundwork for further investigation into the pathogenic mechanisms of *C. viniferum* and the intricacies of the grape and *C. viniferum* interaction.

## Data Availability

All raw RNA-Seq read data in this study can be found in the National Center for Biotechnology Information (NCBI) Sequence Read Archive (SRA) with the accession numbers PRJNA1022593 and PRJNA1022588. The genomic datasets in this study can be found in online repositories. The names of the repository/repositories and accession number(s) can be found in the article/**Supplementary Material**.
