# Supplementary material for: Histological and transcriptomic insights into the interaction between grapevine and Colletotrichum viniferum
Source: Front Plant Sci. 2024 Aug 16;15:1446288. doi: 10.3389/fpls.2024.1446288 (PMC11362058; doi:10.3389/fpls.2024.1446288)
Supplement: Supplementary file 1 [file DataSheet1.docx]

***Supplementary Material***

**Supplementary 1** 16678 orthogroups of 14 fungi genome using OrthoFinder2

>Orthogroup0: ANID|CBF73551.1 ANID|CBF80718.1 ANID|CBF81947.1 ANID|CBF84602.1 ANID|CBF86301.1 BCIN|XP_001547359.1 BCIN|XP_024548692.1 BCIN|XP_024549059.1 BCIN|XP_024551029.1 BCIN|XP_024553094.1 BCIN|XP_024553126.1 BGRA|VDB87784.1 CFRU|XP_031876271.1 CFRU|XP_031878093.1 CFRU|XP_031878348.1 CFRU|XP_031878535.1 CFRU|XP_031878753.1 CFRU|XP_031879024.1 CFRU|XP_031879080.1 CFRU|XP_031880178.1 CFRU|XP_031881538.1 CFRU|XP_031882119.1 CFRU|XP_031882161.1 CFRU|XP_031882619.1 CFRU|XP_031883646.1 CFRU|XP_031883656.1 CFRU|XP_031883663.1 CFRU|XP_031884256.1 CFRU|XP_031884872.1 CFRU|XP_031885484.1 CFRU|XP_031886563.1 CFRU|XP_031887844.1 CFRU|XP_031887930.1 CFRU|XP_031888063.1 CFRU|XP_031888852.1 CFRU|XP_031891208.1 CFRU|XP_031891255.1 CFRU|XP_031891453.1 CFRU|XP_031891511.1 CFRU|XP_031892266.1 CFRU|XP_031892500.1 CGLO|KAF3797041.1 CGLO|KAF3797242.1 CGLO|KAF3797559.1 CGLO|KAF3798097.1 CGLO|KAF3798178.1 CGLO|KAF3798444.1 CGLO|KAF3798683.1 CGLO|KAF3798770.1 CGLO|KAF3798890.1 CGLO|KAF3799332.1 CGLO|KAF3799463.1 CGLO|KAF3799991.1 CGLO|KAF3800194.1 CGLO|KAF3800383.1 CGLO|KAF3802024.1 CGLO|KAF3802182.1 CGLO|KAF3802223.1 CGLO|KAF3802281.1 CGLO|KAF3802358.1 CGLO|KAF3803333.1 CGLO|KAF3803901.1 CGLO|KAF3804072.1 CGLO|KAF3804169.1 CGLO|KAF3804479.1 CGLO|KAF3804655.1 CGLO|KAF3804763.1 CGLO|KAF3805349.1 CGLO|KAF3805661.1 CGLO|KAF3805671.1 CGLO|KAF3805989.1 CGLO|KAF3806104.1 CGLO|KAF3806242.1 CGLO|KAF3806607.1 CGLO|KAF3806677.1 CGLO|KAF3806833.1 CGLO|KAF3808708.1 CGLO|KAF3809912.1 CGLO|KAF3811882.1 CGLO|KAF3812170.1 CHIG|XP_018150708.1 CHIG|XP_018151040.1 CHIG|XP_018151117.1 CHIG|XP_018151242.1 CHIG|XP_018151248.1 CHIG|XP_018151252.1 CHIG|XP_018151693.1 CHIG|XP_018152464.1 CHIG|XP_018152465.1 CHIG|XP_018152583.1 CHIG|XP_018153224.1 CHIG|XP_018153395.1 CHIG|XP_018153600.1 CHIG|XP_018153601.1 CHIG|XP_018154310.1 CHIG|XP_018154321.1 CHIG|XP_018154446.1 CHIG|XP_018154949.1 CHIG|XP_018156768.1 CHIG|XP_018157473.1 CHIG|XP_018159093.1 CHIG|XP_018160016.1 CHIG|XP_018161236.1 CHIG|XP_018161502.1 CHIG|XP_018161503.1 CHIG|XP_018161601.1 CHIG|XP_018163422.1 CHIG|XP_018164048.1 CHIG|XP_018164077.1 CHIG|XP_018164084.1 CHIG|XP_018164172.1 CHIG|XP_018164173.1 CHIG|XP_018164174.1 CVIN|KAF4896999.1 CVIN|KAF4901583.1 CVIN|KAF4901609.1 CVIN|KAF4902333.1 CVIN|KAF4905032.1 CVIN|KAF4905033.1 CVIN|KAF4909289.1 CVIN|KAF4909294.1 CVIN|KAF4910498.1 CVIN|KAF4912766.1 CVIN|KAF4913622.1 CVIN|KAF4913898.1 CVIN|KAF4914840.1 CVIN|KAF4915273.1 CVIN|KAF4915276.1 CVIN|KAF4916855.1 CVIN|KAF4917687.1 CVIN|KAF4918365.1 CVIN|KAF4918492.1 CVIN|KAF4918741.1 CVIN|KAF4919131.1 CVIN|KAF4919287.1 CVIN|KAF4919288.1 CVIN|KAF4919290.1 CVIN|KAF4919539.1 CVIN|KAF4920042.1 CVIN|KAF4920563.1 CVIN|KAF4920564.1 CVIN|KAF4920565.1 CVIN|KAF4921798.1 CVIN|KAF4921928.1 CVIN|KAF4922757.1 CVIN|KAF4923543.1 CVIN|KAF4923589.1 CVIN|KAF4923654.1 CVIN|KAF4923700.1 CVIN|KAF4924412.1 CVIN|KAF4924525.1 CVIN|KAF4924527.1 CVIN|KAF4924528.1 CVIN|KAF4924806.1 CVIN|KAF4924871.1 CVIN|KAF4925411.1 CVIN|KAF4925539.1 CVIN|KAF4925677.1 CVIN|KAF4925679.1 CVIN|KAF4925747.1 CVIN|KAF4925748.1 CVIN|KAF4926873.1 CVIN|KAF4927984.1 CVIN|KAF4929353.1 CVIN|KAF4929630.1 CVIN|KAF4929745.1 CVIN|KAF4929746.1 CVIN|KAF4930731.1 CVIN|KAF4930985.1 CVIN|KAF4931532.1 CVIN|KAF4931800.1 CVYL|A00063 CVYL|A00220 CVYL|A00970 CVYL|A02480 CVYL|A02481 CVYL|A03177 CVYL|A03209 CVYL|A03364 CVYL|A03771 CVYL|A03773 CVYL|A03775 CVYL|A03840 CVYL|A04238 CVYL|A04239 CVYL|A04308 CVYL|A04942 CVYL|A05063 CVYL|A05230 CVYL|A06093 CVYL|A06172 CVYL|A06173 CVYL|A06174 CVYL|A07034 CVYL|A07408 CVYL|A07527 CVYL|A07536 CVYL|A07537 CVYL|A07538 CVYL|A07831 CVYL|A08263 CVYL|A08908 CVYL|A08987 CVYL|A09138 CVYL|A09140 CVYL|A09152 CVYL|A09420 CVYL|A10206 CVYL|A10255 CVYL|A10452 CVYL|A10454 CVYL|A10664 CVYL|A10665 CVYL|A10991 CVYL|A11510 CVYL|A11831 CVYL|A12316 CVYL|A12843 CVYL|A12857 CVYL|A13325 CVYL|A14230 FGRM|XP_011318340.1 FGRM|XP_011321863.1 FGRM|XP_011322065.1 FGRM|XP_011322140.1 FGRM|XP_011322184.1 FGRM|XP_011322475.1 FGRM|XP_011322725.1 FGRM|XP_011322871.1 FGRM|XP_011323063.1 FGRM|XP_011323291.1 FGRM|XP_011325736.1 FGRM|XP_011325913.1 FGRM|XP_011327451.1 FGRM|XP_011327710.1 MGRA|XP_003849984.1 MGRA|XP_003857312.1 MGRA|XP_003857724.1 MORY|QBZ56363.1 MORY|QBZ57005.1 MORY|QBZ58693.1 MORY|QBZ60011.1 MORY|QBZ60766.1 MORY|QBZ65623.1 SSCL|APA09101.1 SSCL|APA09916.1 SSCL|APA11946.1 SSCL|APA15836.1 SSCL|APA16307.1

>Orthogroup1: ANID|CBF74673.1 BCIN|XP_001546099.2 BCIN|XP_001546730.2 BCIN|XP_001549783.1 BCIN|XP_001555131.1 BCIN|XP_001557334.1 BCIN|XP_024546647.1 BCIN|XP_024546913.1 BCIN|XP_024547046.1 BCIN|XP_024547118.1 BCIN|XP_024547167.1 BCIN|XP_024547444.1 BCIN|XP_024548303.1 BCIN|XP_024548304.1 BCIN|XP_024548916.1 BCIN|XP_024549116.1 BCIN|XP_024549149.1 BCIN|XP_024549500.1 BCIN|XP_024549626.1 BCIN|XP_024549950.1 BCIN|XP_024550155.1 BCIN|XP_024550855.1 BCIN|XP_024550905.1 BCIN|XP_024551845.1 BCIN|XP_024551892.1 BCIN|XP_024552101.1 BCIN|XP_024552419.1 BCIN|XP_024552568.1 BCIN|XP_024552590.1 BCIN|XP_024552627.1 CFRU|XP_031877458.1 CFRU|XP_031877510.1 CFRU|XP_031878446.1 CFRU|XP_031878458.1 CFRU|XP_031878927.1 CFRU|XP_031878936.1 CFRU|XP_031879032.1 CFRU|XP_031879281.1 CFRU|XP_031879761.1 CFRU|XP_031880073.1 CFRU|XP_031880281.1 CFRU|XP_031880284.1 CFRU|XP_031881610.1 CFRU|XP_031882338.1 CFRU|XP_031883186.1 CFRU|XP_031883610.1 CFRU|XP_031883634.1 CFRU|XP_031884112.1 CFRU|XP_031885276.1 CFRU|XP_031887322.1 CFRU|XP_031887662.1 CFRU|XP_031888810.1 CFRU|XP_031888839.1 CFRU|XP_031889213.1 CFRU|XP_031889380.1 CFRU|XP_031889719.1 CFRU|XP_031889869.1 CFRU|XP_031890726.1 CFRU|XP_031890914.1 CFRU|XP_031891004.1 CFRU|XP_031891078.1 CFRU|XP_031892273.1 CFRU|XP_031893177.1 CGLO|KAF3797186.1 CGLO|KAF3797535.1 CGLO|KAF3798141.1 CGLO|KAF3798210.1 CGLO|KAF3798440.1 CGLO|KAF3798591.1 CGLO|KAF3798774.1 CGLO|KAF3799791.1 CGLO|KAF3799980.1 CGLO|KAF3800238.1 CGLO|KAF3801441.1 CGLO|KAF3801583.1 CGLO|KAF3802584.1 CGLO|KAF3803342.1 CGLO|KAF3803851.1 CGLO|KAF3803994.1 CGLO|KAF3804238.1 CGLO|KAF3804246.1 CGLO|KAF3804247.1 CGLO|KAF3804316.1 CGLO|KAF3805624.1 CGLO|KAF3805626.1 CGLO|KAF3805938.1 CGLO|KAF3807193.1 CGLO|KAF3807400.1 CGLO|KAF3807776.1 CGLO|KAF3807902.1 CGLO|KAF3808107.1 CGLO|KAF3808665.1 CGLO|KAF3808754.1 CGLO|KAF3808948.1 CGLO|KAF3810386.1 CGLO|KAF3810527.1 CGLO|KAF3811006.1 CGLO|KAF3811707.1 CGLO|KAF3811805.1 CHIG|XP_018151091.1 CHIG|XP_018151562.1 CHIG|XP_018151715.1 CHIG|XP_018152503.1 CHIG|XP_018152929.1 CHIG|XP_018153052.1 CHIG|XP_018153125.1 CHIG|XP_018153276.1 CHIG|XP_018154219.1 CHIG|XP_018154227.1 CHIG|XP_018155071.1 CHIG|XP_018158052.1 CHIG|XP_018158999.1 CHIG|XP_018159448.1 CHIG|XP_018159706.1 CHIG|XP_018160010.1 CHIG|XP_018161623.1 CHIG|XP_018161840.1 CHIG|XP_018161855.1 CHIG|XP_018164833.1 CVIN|KAF4905046.1 CVIN|KAF4906973.1 CVIN|KAF4906975.1 CVIN|KAF4912404.1 CVIN|KAF4912761.1 CVIN|KAF4912762.1 CVIN|KAF4914564.1 CVIN|KAF4918730.1 CVIN|KAF4920045.1 CVIN|KAF4920210.1 CVIN|KAF4920521.1 CVIN|KAF4921651.1 CVIN|KAF4924880.1 CVIN|KAF4925691.1 CVIN|KAF4926167.1 CVIN|KAF4929655.1 CVYL|A00826 CVYL|A01984 CVYL|A03783 CVYL|A03905 CVYL|A03919 CVYL|A04003 CVYL|A04802 CVYL|A07573 CVYL|A09148 CVYL|A09589 CVYL|A10892 CVYL|A11197 CVYL|A11755 CVYL|A12412 CVYL|A12581 CVYL|A12809 CVYL|A13775 FGRM|XP_011315732.1 FGRM|XP_011317912.1 FGRM|XP_011318020.1 FGRM|XP_011318033.1 FGRM|XP_011318066.1 FGRM|XP_011319556.1 FGRM|XP_011319666.1 FGRM|XP_011319719.1 FGRM|XP_011320674.1 FGRM|XP_011320699.1 FGRM|XP_011320724.1 FGRM|XP_011320910.1 FGRM|XP_011320940.1 FGRM|XP_011321644.1 FGRM|XP_011321842.1 FGRM|XP_011322028.1 FGRM|XP_011322130.1 FGRM|XP_011322351.1 FGRM|XP_011322786.1 FGRM|XP_011323083.1 FGRM|XP_011323240.1 FGRM|XP_011323255.1 FGRM|XP_011325959.1 FGRM|XP_011327437.1 FGRM|XP_011327505.1 FGRM|XP_011327990.1 MORY|QBZ54818.1 MORY|QBZ54917.1 MORY|QBZ55091.1 MORY|QBZ55874.1 MORY|QBZ57073.1 MORY|QBZ60060.1 MORY|QBZ60769.1 MORY|QBZ62402.1 MORY|QBZ63358.1 MORY|QBZ64533.1 MORY|QBZ65589.1 MORY|QBZ66438.1 NCRA|XP_011393060.1 NCRA|XP_011393342.1 NCRA|XP_011393714.1 NCRA|XP_011393716.1 NCRA|XP_011394351.1 NCRA|XP_011394425.1 NCRA|XP_011394928.1 NCRA|XP_011395076.1 NCRA|XP_011395083.1 NCRA|XP_011395397.1 NCRA|XP_955771.1 NCRA|XP_955881.1 NCRA|XP_955884.1 NCRA|XP_955911.2 NCRA|XP_956313.1 NCRA|XP_957983.1 NCRA|XP_958501.2 NCRA|XP_958565.2 NCRA|XP_960100.3 NCRA|XP_960281.3 NCRA|XP_961943.3 NCRA|XP_962493.2 NCRA|XP_962689.2 NCRA|XP_962764.1 NCRA|XP_963393.3 NCRA|XP_963582.1 NCRA|XP_963598.2 NCRA|XP_963647.3 NCRA|XP_964033.2 NCRA|XP_964635.3 NCRA|XP_965049.2 SSCL|APA05395.1 SSCL|APA05697.1 SSCL|APA07154.1 SSCL|APA07448.1 SSCL|APA08676.1 SSCL|APA08799.1 SSCL|APA09141.1 SSCL|APA11782.1 SSCL|APA11880.1 SSCL|APA12555.1 SSCL|APA14017.1 SSCL|APA14021.1 SSCL|APA15740.1 SSCL|APA15896.1 SSCL|APA15988.1

>Orthogroup2: ANID|CBF69492.1 ANID|CBF73467.1 ANID|CBF75223.1 ANID|CBF76006.1 ANID|CBF83171.1 ANID|CBF85202.1 ANID|CBF88276.1 BCIN|XP_001548419.1 BCIN|XP_001553240.1 BCIN|XP_001553245.1 BCIN|XP_001558907.1 BCIN|XP_024547809.1 BCIN|XP_024549083.1 BCIN|XP_024549501.1 BCIN|XP_024551869.1 BCIN|XP_024553353.1 CFRU|XP_031876398.1 CFRU|XP_031876402.1 CFRU|XP_031876647.1 CFRU|XP_031878507.1 CFRU|XP_031878798.1 CFRU|XP_031879120.1 CFRU|XP_031879487.1 CFRU|XP_031879666.1 CFRU|XP_031879895.1 CFRU|XP_031880286.1 CFRU|XP_031880962.1 CFRU|XP_031881028.1 CFRU|XP_031881499.1 CFRU|XP_031881797.1 CFRU|XP_031883012.1 CFRU|XP_031883600.1 CFRU|XP_031884290.1 CFRU|XP_031886097.1 CFRU|XP_031888959.1 CFRU|XP_031889098.1 CFRU|XP_031889325.1 CFRU|XP_031889554.1 CFRU|XP_031889935.1 CFRU|XP_031890941.1 CFRU|XP_031891583.1 CFRU|XP_031892192.1 CFRU|XP_031892288.1 CFRU|XP_031892342.1 CGLO|KAF3797499.1 CGLO|KAF3797631.1 CGLO|KAF3797786.1 CGLO|KAF3798312.1 CGLO|KAF3798564.1 CGLO|KAF3798958.1 CGLO|KAF3799257.1 CGLO|KAF3799807.1 CGLO|KAF3800207.1 CGLO|KAF3800398.1 CGLO|KAF3800410.1 CGLO|KAF3800669.1 CGLO|KAF3800741.1 CGLO|KAF3801278.1 CGLO|KAF3801563.1 CGLO|KAF3801905.1 CGLO|KAF3802442.1 CGLO|KAF3803094.1 CGLO|KAF3805504.1 CGLO|KAF3805788.1 CGLO|KAF3808891.1 CGLO|KAF3808954.1 CGLO|KAF3809067.1 CGLO|KAF3809816.1 CGLO|KAF3810414.1 CGLO|KAF3810445.1 CHIG|XP_018151986.1 CHIG|XP_018152855.1 CHIG|XP_018153080.1 CHIG|XP_018153791.1 CHIG|XP_018153968.1 CHIG|XP_018154739.1 CHIG|XP_018155120.1 CHIG|XP_018155356.1 CHIG|XP_018155419.1 CHIG|XP_018155591.1 CHIG|XP_018155797.1 CHIG|XP_018155904.1 CHIG|XP_018156457.1 CHIG|XP_018156459.1 CHIG|XP_018156612.1 CHIG|XP_018158322.1 CHIG|XP_018158406.1 CHIG|XP_018159173.1 CHIG|XP_018159546.1 CHIG|XP_018159900.1 CHIG|XP_018161274.1 CHIG|XP_018163291.1 CHIG|XP_018163683.1 CHIG|XP_018164857.1 CVIN|KAF4895290.1 CVIN|KAF4908464.1 CVIN|KAF4908839.1 CVIN|KAF4913916.1 CVIN|KAF4918300.1 CVIN|KAF4918714.1 CVIN|KAF4919337.1 CVIN|KAF4919646.1 CVIN|KAF4919753.1 CVIN|KAF4920405.1 CVIN|KAF4920731.1 CVIN|KAF4921090.1 CVIN|KAF4921593.1 CVIN|KAF4921623.1 CVIN|KAF4922050.1 CVIN|KAF4923134.1 CVIN|KAF4923161.1 CVIN|KAF4923460.1 CVIN|KAF4924623.1 CVIN|KAF4926622.1 CVIN|KAF4926931.1 CVIN|KAF4929611.1 CVIN|KAF4930120.1 CVIN|KAF4930601.1 CVIN|KAF4930684.1 CVYL|A00463 CVYL|A00986 CVYL|A01181 CVYL|A01645 CVYL|A01793 CVYL|A01805 CVYL|A01948 CVYL|A02034 CVYL|A03010 CVYL|A03011 CVYL|A04876 CVYL|A05628 CVYL|A06032 CVYL|A06079 CVYL|A06734 CVYL|A07809 CVYL|A07875 CVYL|A10150 CVYL|A10186 CVYL|A11388 CVYL|A12571 CVYL|A13048 FGRM|XP_011318094.1 FGRM|XP_011318522.1 FGRM|XP_011320820.1 FGRM|XP_011320885.1 FGRM|XP_011321694.1 FGRM|XP_011322000.1 FGRM|XP_011322068.1 FGRM|XP_011323218.1 FGRM|XP_011324734.1 FGRM|XP_011325060.1 FGRM|XP_011327747.1 MGRA|XP_003855239.1 MGRA|XP_003856492.1 MLAR|XP_007408111.1 MLAR|XP_007410836.1 MLAR|XP_007413420.1 MLAR|XP_007413456.1 MORY|QBZ53475.1 MORY|QBZ53590.1 MORY|QBZ53712.1 MORY|QBZ53901.1 MORY|QBZ54874.1 MORY|QBZ56836.1 MORY|QBZ56889.1 MORY|QBZ57435.1 MORY|QBZ57550.1 MORY|QBZ57806.1 MORY|QBZ58154.1 MORY|QBZ58678.1 MORY|QBZ60923.1 MORY|QBZ61147.1 MORY|QBZ61617.1 MORY|QBZ61923.1 MORY|QBZ63550.1 MORY|QBZ64672.1 MORY|QBZ65034.1 MORY|QBZ66348.1 NCRA|XP_955892.1 NCRA|XP_956109.2 NCRA|XP_958254.1 NCRA|XP_958368.1 NCRA|XP_958583.1 NCRA|XP_959466.1 NCRA|XP_959499.1 NCRA|XP_961662.1 NCRA|XP_962414.2 NCRA|XP_963702.1 NCRA|XP_965386.2 NCRA|XP_965498.1 NCRA|XP_965598.1 SSCL|APA05399.1 SSCL|APA05793.1 SSCL|APA09140.1 SSCL|APA09281.1 SSCL|APA12064.1 SSCL|APA12066.1 SSCL|APA13672.1 SSCL|APA15869.1

>Orthogroup3: ANID|CBF69487.1 ANID|CBF71467.1 ANID|CBF75778.1 ANID|CBF75781.1 ANID|CBF79115.1 ANID|CBF80211.1 ANID|CBF82304.1 ANID|CBF83036.1 ANID|CBF83055.1 ANID|CBF84476.1 ANID|CBF84683.1 ANID|CBF86052.1 ANID|CBF87072.1 ANID|CBF88289.1 BCIN|XP_001557060.1 BCIN|XP_001558952.2 BCIN|XP_024545901.1 BCIN|XP_024548143.1 BCIN|XP_024549041.1 BCIN|XP_024549743.1 BCIN|XP_024550296.1 BCIN|XP_024552769.1 BCIN|XP_024552825.1 CFRU|XP_031876071.1 CFRU|XP_031876596.1 CFRU|XP_031876819.1 CFRU|XP_031878257.1 CFRU|XP_031878678.1 CFRU|XP_031879045.1 CFRU|XP_031879097.1 CFRU|XP_031879385.1 CFRU|XP_031879492.1 CFRU|XP_031881669.1 CFRU|XP_031882578.1 CFRU|XP_031884130.1 CFRU|XP_031885247.1 CFRU|XP_031885929.1 CFRU|XP_031887190.1 CFRU|XP_031888900.1 CFRU|XP_031889624.1 CFRU|XP_031889874.1 CFRU|XP_031890065.1 CFRU|XP_031890318.1 CFRU|XP_031890697.1 CFRU|XP_031892623.1 CFRU|XP_031892898.1 CFRU|XP_031893225.1 CGLO|KAF3796919.1 CGLO|KAF3797175.1 CGLO|KAF3798077.1 CGLO|KAF3798758.1 CGLO|KAF3798813.1 CGLO|KAF3798816.1 CGLO|KAF3799176.1 CGLO|KAF3799978.1 CGLO|KAF3801976.1 CGLO|KAF3802434.1 CGLO|KAF3803840.1 CGLO|KAF3803938.1 CGLO|KAF3804584.1 CGLO|KAF3805983.1 CGLO|KAF3805984.1 CGLO|KAF3806755.1 CGLO|KAF3806818.1 CGLO|KAF3807757.1 CGLO|KAF3808561.1 CGLO|KAF3809386.1 CGLO|KAF3809521.1 CGLO|KAF3809602.1 CGLO|KAF3810645.1 CHIG|XP_018150468.1 CHIG|XP_018150503.1 CHIG|XP_018150745.1 CHIG|XP_018151082.1 CHIG|XP_018151207.1 CHIG|XP_018151210.1 CHIG|XP_018151211.1 CHIG|XP_018151295.1 CHIG|XP_018151609.1 CHIG|XP_018151641.1 CHIG|XP_018152642.1 CHIG|XP_018153301.1 CHIG|XP_018154735.1 CHIG|XP_018155047.1 CHIG|XP_018156227.1 CHIG|XP_018156836.1 CHIG|XP_018157330.1 CHIG|XP_018159141.1 CHIG|XP_018159360.1 CHIG|XP_018159780.1 CHIG|XP_018160179.1 CHIG|XP_018161573.1 CHIG|XP_018161970.1 CHIG|XP_018161971.1 CHIG|XP_018162284.1 CHIG|XP_018163318.1 CHIG|XP_018163485.1 CHIG|XP_018164900.1 CVIN|KAF4890320.1 CVIN|KAF4903545.1 CVIN|KAF4904103.1 CVIN|KAF4904635.1 CVIN|KAF4906493.1 CVIN|KAF4906649.1 CVIN|KAF4907266.1 CVIN|KAF4912772.1 CVIN|KAF4914903.1 CVIN|KAF4917782.1 CVIN|KAF4917799.1 CVIN|KAF4918533.1 CVIN|KAF4918834.1 CVIN|KAF4919586.1 CVIN|KAF4919588.1 CVIN|KAF4919769.1 CVIN|KAF4919805.1 CVIN|KAF4920518.1 CVIN|KAF4923060.1 CVIN|KAF4930370.1 CVYL|A00842 CVYL|A02000 CVYL|A02411 CVYL|A02412 CVYL|A02875 CVYL|A05295 CVYL|A05488 CVYL|A08441 CVYL|A08498 CVYL|A08865 CVYL|A08960 CVYL|A09114 CVYL|A09117 CVYL|A09162 CVYL|A09220 CVYL|A10521 CVYL|A10642 CVYL|A10978 CVYL|A11584 CVYL|A12447 CVYL|A13577 CVYL|A13730 CVYL|A13732 CVYL|A13737 CVYL|A13738 FGRM|XP_011317630.1 FGRM|XP_011318310.1 FGRM|XP_011319546.1 FGRM|XP_011319925.1 FGRM|XP_011320599.1 FGRM|XP_011322392.1 FGRM|XP_011322985.1 FGRM|XP_011323126.1 FGRM|XP_011324386.1 MGRA|XP_003847731.1 MGRA|XP_003848974.1 MGRA|XP_003849645.1 MGRA|XP_003850944.1 MGRA|XP_003852001.1 MGRA|XP_003852783.1 MGRA|XP_003855801.1 MORY|QBZ57772.1 MORY|QBZ58317.1 MORY|QBZ61628.1 MORY|QBZ61696.1 MORY|QBZ62417.1 MORY|QBZ63120.1 MORY|QBZ63697.1 MORY|QBZ63711.1 MORY|QBZ64004.1 MORY|QBZ64367.1 MORY|QBZ65009.1 MORY|QBZ66081.1 MORY|QBZ66472.1 MORY|QBZ66480.1 NCRA|XP_955938.1 NCRA|XP_958135.3 NCRA|XP_958169.1 NCRA|XP_959122.2 NCRA|XP_965600.1 SSCL|APA05675.1 SSCL|APA05821.1 SSCL|APA05830.1 SSCL|APA08511.1 SSCL|APA08629.1 SSCL|APA10415.1 SSCL|APA10781.1 SSCL|APA11194.1 SSCL|APA14871.1 SSCL|APA15730.1 SSCL|APA15878.1 SSCL|APA16269.1

>Orthogroup4: ANID|CBF69412.1 ANID|CBF69480.1 ANID|CBF79557.1 ANID|CBF80160.1 ANID|CBF80171.1 ANID|CBF83038.1 ANID|CBF85469.1 BCIN|XP_001545401.1 BCIN|XP_024548814.1 BCIN|XP_024548872.1 BCIN|XP_024549090.1 BCIN|XP_024550073.1 BCIN|XP_024550390.1 BCIN|XP_024550907.1 BCIN|XP_024551956.1 BCIN|XP_024552381.1 BCIN|XP_024553446.1 BCIN|XP_024554041.1 BGRA|VCU39358.1 BGRA|VCU39359.1 CFRU|XP_031875742.1 CFRU|XP_031876469.1 CFRU|XP_031876867.1 CFRU|XP_031877859.1 CFRU|XP_031877972.1 CFRU|XP_031878301.1 CFRU|XP_031878436.1 CFRU|XP_031879030.1 CFRU|XP_031879486.1 CFRU|XP_031880461.1 CFRU|XP_031880905.1 CFRU|XP_031882855.1 CFRU|XP_031883473.1 CFRU|XP_031883812.1 CFRU|XP_031884263.1 CFRU|XP_031884430.1 CFRU|XP_031884584.1 CFRU|XP_031884746.1 CFRU|XP_031885127.1 CFRU|XP_031886825.1 CFRU|XP_031889628.1 CFRU|XP_031889672.1 CFRU|XP_031890058.1 CFRU|XP_031890123.1 CFRU|XP_031890263.1 CFRU|XP_031890956.1 CFRU|XP_031891059.1 CFRU|XP_031892806.1 CFRU|XP_031892876.1 CFRU|XP_031892929.1 CGLO|KAF3797061.1 CGLO|KAF3797755.1 CGLO|KAF3798016.1 CGLO|KAF3798241.1 CGLO|KAF3798757.1 CGLO|KAF3798866.1 CGLO|KAF3798976.1 CGLO|KAF3799753.1 CGLO|KAF3799846.1 CGLO|KAF3801534.1 CGLO|KAF3803795.1 CGLO|KAF3805973.1 CGLO|KAF3806609.1 CGLO|KAF3806661.1 CGLO|KAF3806991.1 CGLO|KAF3807276.1 CGLO|KAF3807505.1 CGLO|KAF3807781.1 CGLO|KAF3809443.1 CGLO|KAF3810517.1 CGLO|KAF3810803.1 CGLO|KAF3810887.1 CGLO|KAF3811679.1 CHIG|XP_018151547.1 CHIG|XP_018151887.1 CHIG|XP_018152621.1 CHIG|XP_018152713.1 CHIG|XP_018153113.1 CHIG|XP_018153116.1 CHIG|XP_018153124.1 CHIG|XP_018154067.1 CHIG|XP_018154918.1 CHIG|XP_018155056.1 CHIG|XP_018156551.1 CHIG|XP_018156952.1 CHIG|XP_018157035.1 CHIG|XP_018157389.1 CHIG|XP_018158001.1 CHIG|XP_018158057.1 CHIG|XP_018158726.1 CHIG|XP_018158985.1 CHIG|XP_018159359.1 CHIG|XP_018159597.1 CHIG|XP_018159675.1 CHIG|XP_018163011.1 CHIG|XP_018164246.1 CVIN|KAF4894650.1 CVIN|KAF4900884.1 CVIN|KAF4908447.1 CVIN|KAF4909118.1 CVIN|KAF4912447.1 CVIN|KAF4912986.1 CVIN|KAF4914570.1 CVIN|KAF4915624.1 CVIN|KAF4915955.1 CVIN|KAF4916373.1 CVIN|KAF4917504.1 CVIN|KAF4917808.1 CVIN|KAF4917899.1 CVIN|KAF4918233.1 CVIN|KAF4918782.1 CVIN|KAF4918819.1 CVIN|KAF4918939.1 CVIN|KAF4918970.1 CVIN|KAF4920306.1 CVIN|KAF4921128.1 CVIN|KAF4921491.1 CVIN|KAF4921696.1 CVIN|KAF4921780.1 CVIN|KAF4923459.1 CVIN|KAF4928178.1 CVIN|KAF4929826.1 CVYL|A00820 CVYL|A01133 CVYL|A02257 CVYL|A02970 CVYL|A04817 CVYL|A05027 CVYL|A05737 CVYL|A05905 CVYL|A06285 CVYL|A07895 CVYL|A08225 CVYL|A08271 CVYL|A08341 CVYL|A09070 CVYL|A09224 CVYL|A09669 CVYL|A10643 CVYL|A10759 CVYL|A11343 CVYL|A12318 CVYL|A12602 CVYL|A12701 CVYL|A13575 CVYL|A13597 CVYL|A13748 CVYL|A14166 CVYL|A14200 FGRM|XP_011317575.1 FGRM|XP_011319705.1 FGRM|XP_011320806.1 FGRM|XP_011322169.1 FGRM|XP_011322569.1 FGRM|XP_011327580.1 MGRA|XP_003848976.1 MGRA|XP_003849026.1 MGRA|XP_003849271.1 MGRA|XP_003851188.1 MGRA|XP_003853723.1 MGRA|XP_003857256.1 MGRA|XP_003857438.1 MORY|QBZ54718.1 MORY|QBZ54916.1 MORY|QBZ58072.1 MORY|QBZ58124.1 MORY|QBZ58279.1 MORY|QBZ60030.1 MORY|QBZ60031.1 MORY|QBZ60214.1 MORY|QBZ60710.1 MORY|QBZ61237.1 MORY|QBZ61699.1 MORY|QBZ61995.1 MORY|QBZ62610.1 MORY|QBZ63371.1 MORY|QBZ64944.1 MORY|QBZ65719.1 MORY|QBZ66482.1 MORY|QBZ66580.1 NCRA|XP_959671.3 NCRA|XP_964385.2 SSCL|APA07495.1 SSCL|APA09629.1 SSCL|APA11875.1 SSCL|APA13793.1 SSCL|APA13827.1 SSCL|APA13853.1 SSCL|APA15884.1

>Orthogroup5: ANID|CBF69573.1 ANID|CBF71817.1 ANID|CBF73717.1 ANID|CBF73917.1 ANID|CBF75176.1 ANID|CBF78331.1 ANID|CBF78540.1 ANID|CBF80303.1 ANID|CBF80389.1 ANID|CBF80439.1 ANID|CBF80744.1 ANID|CBF80809.1 ANID|CBF81003.1 ANID|CBF81142.1 ANID|CBF83035.1 ANID|CBF83450.1 ANID|CBF84480.1 ANID|CBF85153.1 ANID|CBF85596.1 ANID|CBF87762.1 ANID|CBF88083.1 ANID|CBF88209.1 ANID|CBF88770.1 BCIN|XP_001555324.1 BCIN|XP_024545988.1 BCIN|XP_024546819.1 BCIN|XP_024548113.1 BCIN|XP_024548124.1 BCIN|XP_024548427.1 BCIN|XP_024550158.1 BCIN|XP_024552742.1 CFRU|XP_031875316.1 CFRU|XP_031875319.1 CFRU|XP_031875396.1 CFRU|XP_031875512.1 CFRU|XP_031875850.1 CFRU|XP_031876481.1 CFRU|XP_031877003.1 CFRU|XP_031877014.1 CFRU|XP_031877735.1 CFRU|XP_031877750.1 CFRU|XP_031878896.1 CFRU|XP_031879701.1 CFRU|XP_031880527.1 CFRU|XP_031881524.1 CFRU|XP_031882141.1 CFRU|XP_031883985.1 CFRU|XP_031884356.1 CFRU|XP_031884377.1 CFRU|XP_031885625.1 CFRU|XP_031885778.1 CFRU|XP_031886805.1 CFRU|XP_031887187.1 CFRU|XP_031887188.1 CFRU|XP_031888020.1 CFRU|XP_031888067.1 CFRU|XP_031889307.1 CFRU|XP_031889416.1 CFRU|XP_031889741.1 CFRU|XP_031890144.1 CFRU|XP_031890284.1 CFRU|XP_031890878.1 CFRU|XP_031891114.1 CFRU|XP_031893009.1 CFRU|XP_031893147.1 CFRU|XP_031893149.1 CFRU|XP_031893167.1 CFRU|XP_031893238.1 CFRU|XP_031893512.1 CGLO|KAF3797336.1 CGLO|KAF3797994.1 CGLO|KAF3797995.1 CGLO|KAF3798753.1 CGLO|KAF3799041.1 CGLO|KAF3799940.1 CGLO|KAF3801466.1 CGLO|KAF3802097.1 CGLO|KAF3802186.1 CGLO|KAF3804322.1 CGLO|KAF3804703.1 CGLO|KAF3805619.1 CGLO|KAF3805672.1 CGLO|KAF3805713.1 CGLO|KAF3806091.1 CGLO|KAF3806406.1 CGLO|KAF3807483.1 CGLO|KAF3807678.1 CGLO|KAF3808118.1 CGLO|KAF3808906.1 CGLO|KAF3808907.1 CGLO|KAF3809380.1 CGLO|KAF3809591.1 CGLO|KAF3809659.1 CGLO|KAF3810141.1 CGLO|KAF3810750.1 CHIG|XP_018150469.1 CHIG|XP_018150624.1 CHIG|XP_018151064.1 CHIG|XP_018152010.1 CHIG|XP_018152537.1 CHIG|XP_018152599.1 CHIG|XP_018154148.1 CHIG|XP_018154168.1 CHIG|XP_018156598.1 CHIG|XP_018163084.1 CHIG|XP_018163273.1 CHIG|XP_018163505.1 CHIG|XP_018163588.1 CHIG|XP_018164581.1 CVIN|KAF4891225.1 CVIN|KAF4893181.1 CVIN|KAF4895288.1 CVIN|KAF4896776.1 CVIN|KAF4903089.1 CVIN|KAF4903210.1 CVIN|KAF4903211.1 CVIN|KAF4903834.1 CVIN|KAF4907611.1 CVIN|KAF4912142.1 CVIN|KAF4917665.1 CVIN|KAF4917995.1 CVIN|KAF4920129.1 CVIN|KAF4920627.1 CVIN|KAF4920667.1 CVIN|KAF4922628.1 CVIN|KAF4922750.1 CVIN|KAF4923568.1 CVIN|KAF4927774.1 CVYL|A02654 CVYL|A02730 CVYL|A03081 CVYL|A04014 CVYL|A08116 CVYL|A08361 CVYL|A08391 CVYL|A08444 CVYL|A09008 CVYL|A09832 CVYL|A09833 CVYL|A10356 CVYL|A10357 CVYL|A11380 FGRM|XP_011315568.1 FGRM|XP_011318227.1 FGRM|XP_011319573.1 FGRM|XP_011320520.1 FGRM|XP_011320658.1 FGRM|XP_011320711.1 FGRM|XP_011321061.1 FGRM|XP_011321087.1 FGRM|XP_011321680.1 FGRM|XP_011321876.1 FGRM|XP_011323289.1 FGRM|XP_011323862.1 FGRM|XP_011325800.1 FGRM|XP_011328610.1 FGRM|XP_011328611.1 MGRA|XP_003849341.1 MGRA|XP_003852530.1 MGRA|XP_003852537.1 MGRA|XP_003852882.1 MGRA|XP_003856715.1 MORY|QBZ53271.1 MORY|QBZ54828.1 MORY|QBZ56805.1 MORY|QBZ57636.1 MORY|QBZ57641.1 MORY|QBZ57642.1 MORY|QBZ58396.1 MORY|QBZ58446.1 MORY|QBZ58497.1 MORY|QBZ58570.1 MORY|QBZ59220.1 MORY|QBZ59472.1 MORY|QBZ63396.1 MORY|QBZ65461.1 MORY|QBZ66588.1 NCRA|XP_961929.3 SSCL|APA06065.1 SSCL|APA07437.1 SSCL|APA12249.1 SSCL|APA13127.1

>Orthogroup6: BCIN|XP_001557605.1 BCIN|XP_001558640.2 BCIN|XP_024546781.1 BCIN|XP_024548618.1 BCIN|XP_024553838.1 CFRU|XP_031875507.1 CFRU|XP_031876575.1 CFRU|XP_031877887.1 CFRU|XP_031878055.1 CFRU|XP_031878090.1 CFRU|XP_031879133.1 CFRU|XP_031879932.1 CFRU|XP_031880292.1 CFRU|XP_031880304.1 CFRU|XP_031880462.1 CFRU|XP_031880740.1 CFRU|XP_031881125.1 CFRU|XP_031881559.1 CFRU|XP_031881563.1 CFRU|XP_031882118.1 CFRU|XP_031883050.1 CFRU|XP_031883123.1 CFRU|XP_031883611.1 CFRU|XP_031884003.1 CFRU|XP_031884950.1 CFRU|XP_031885099.1 CFRU|XP_031885183.1 CFRU|XP_031888335.1 CFRU|XP_031889152.1 CFRU|XP_031889631.1 CFRU|XP_031890068.1 CFRU|XP_031890095.1 CFRU|XP_031890246.1 CFRU|XP_031891684.1 CFRU|XP_031892038.1 CFRU|XP_031892904.1 CFRU|XP_031893248.1 CGLO|KAF3798155.1 CGLO|KAF3798315.1 CGLO|KAF3799001.1 CGLO|KAF3800248.1 CGLO|KAF3800719.1 CGLO|KAF3800861.1 CGLO|KAF3800887.1 CGLO|KAF3801046.1 CGLO|KAF3801424.1 CGLO|KAF3801853.1 CGLO|KAF3802012.1 CGLO|KAF3802878.1 CGLO|KAF3803721.1 CGLO|KAF3805017.1 CGLO|KAF3805634.1 CGLO|KAF3805656.1 CGLO|KAF3806073.1 CGLO|KAF3806633.1 CGLO|KAF3807255.1 CGLO|KAF3807647.1 CGLO|KAF3807722.1 CGLO|KAF3808208.1 CGLO|KAF3808802.1 CGLO|KAF3808929.1 CGLO|KAF3809792.1 CGLO|KAF3810378.1 CGLO|KAF3810379.1 CGLO|KAF3810537.1 CGLO|KAF3810962.1 CGLO|KAF3811171.1 CGLO|KAF3812188.1 CHIG|XP_018150796.1 CHIG|XP_018150840.1 CHIG|XP_018152514.1 CHIG|XP_018155060.1 CHIG|XP_018155727.1 CHIG|XP_018156670.1 CHIG|XP_018157953.1 CHIG|XP_018158104.1 CHIG|XP_018158478.1 CHIG|XP_018159077.1 CHIG|XP_018160411.1 CHIG|XP_018163673.1 CHIG|XP_018163723.1 CHIG|XP_018164750.1 CVIN|KAF4901934.1 CVIN|KAF4902334.1 CVIN|KAF4906499.1 CVIN|KAF4906820.1 CVIN|KAF4907243.1 CVIN|KAF4909110.1 CVIN|KAF4909111.1 CVIN|KAF4911850.1 CVIN|KAF4913566.1 CVIN|KAF4914549.1 CVIN|KAF4915188.1 CVIN|KAF4915581.1 CVIN|KAF4917632.1 CVIN|KAF4918005.1 CVIN|KAF4918525.1 CVIN|KAF4918913.1 CVIN|KAF4919140.1 CVIN|KAF4919638.1 CVIN|KAF4920754.1 CVIN|KAF4920974.1 CVIN|KAF4920975.1 CVIN|KAF4922375.1 CVIN|KAF4922902.1 CVIN|KAF4923443.1 CVIN|KAF4923847.1 CVIN|KAF4924303.1 CVIN|KAF4927088.1 CVIN|KAF4927809.1 CVIN|KAF4928191.1 CVIN|KAF4928493.1 CVIN|KAF4931234.1 CVYL|A00202 CVYL|A00337 CVYL|A00485 CVYL|A00526 CVYL|A00862 CVYL|A01158 CVYL|A01468 CVYL|A02440 CVYL|A04333 CVYL|A05135 CVYL|A05173 CVYL|A05733 CVYL|A06609 CVYL|A06714 CVYL|A07660 CVYL|A07920 CVYL|A09057 CVYL|A10735 CVYL|A10944 CVYL|A10947 CVYL|A11100 CVYL|A11127 CVYL|A11591 CVYL|A11604 CVYL|A12346 CVYL|A12388 CVYL|A12854 CVYL|A12951 CVYL|A13615 CVYL|A14657 FGRM|XP_011315823.1 FGRM|XP_011316265.1 FGRM|XP_011319298.1 FGRM|XP_011320355.1 FGRM|XP_011322458.1 FGRM|XP_011323256.1 FGRM|XP_011325182.1 FGRM|XP_011325351.1 FGRM|XP_011325632.1 FGRM|XP_011325772.1 FGRM|XP_011325875.1 FGRM|XP_011325947.1 FGRM|XP_011327408.1 FGRM|XP_011327483.1 MGRA|XP_003847413.1 MGRA|XP_003849925.1 MGRA|XP_003852060.1 MGRA|XP_003853682.1 MGRA|XP_003855526.1 MORY|QBZ54443.1 MORY|QBZ54501.1 MORY|QBZ59490.1 MORY|QBZ61226.1 MORY|QBZ63744.1 MORY|QBZ64382.1 NCRA|XP_001728062.2 NCRA|XP_001728071.2 NCRA|XP_958118.2 NCRA|XP_958230.3 NCRA|XP_959360.1 NCRA|XP_962350.1 NCRA|XP_962566.3 SSCL|APA07431.1 SSCL|APA12600.1 SSCL|APA14823.1 SSCL|APA15329.1

>Orthogroup7: ANID|CBF73873.1 ANID|CBF76046.1 ANID|CBF78879.1 ANID|CBF80597.1 ANID|CBF82022.1 ANID|CBF83093.1 ANID|CBF84291.1 ANID|CBF87265.1 ANID|CBF88300.1 BCIN|XP_001551296.1 BCIN|XP_001553250.1 BCIN|XP_001557507.1 BCIN|XP_001560552.2 BCIN|XP_024546022.1 BCIN|XP_024546639.1 BCIN|XP_024546923.1 BCIN|XP_024547483.1 BCIN|XP_024547765.1 BCIN|XP_024548200.1 BCIN|XP_024549607.1 BCIN|XP_024549948.1 BCIN|XP_024550950.1 BCIN|XP_024551770.1 BCIN|XP_024551829.1 CFRU|XP_031876562.1 CFRU|XP_031876728.1 CFRU|XP_031877915.1 CFRU|XP_031879013.1 CFRU|XP_031879098.1 CFRU|XP_031880004.1 CFRU|XP_031880356.1 CFRU|XP_031881079.1 CFRU|XP_031881998.1 CFRU|XP_031882199.1 CFRU|XP_031883819.1 CFRU|XP_031885632.1 CFRU|XP_031885826.1 CFRU|XP_031887758.1 CFRU|XP_031887836.1 CFRU|XP_031891784.1 CFRU|XP_031891825.1 CFRU|XP_031892352.1 CFRU|XP_031892878.1 CFRU|XP_031893375.1 CGLO|KAF3797165.1 CGLO|KAF3798679.1 CGLO|KAF3798815.1 CGLO|KAF3798856.1 CGLO|KAF3798993.1 CGLO|KAF3799180.1 CGLO|KAF3800070.1 CGLO|KAF3800443.1 CGLO|KAF3800881.1 CGLO|KAF3801337.1 CGLO|KAF3802278.1 CGLO|KAF3803276.1 CGLO|KAF3804409.1 CGLO|KAF3805689.1 CGLO|KAF3806822.1 CGLO|KAF3807239.1 CGLO|KAF3808048.1 CGLO|KAF3810066.1 CGLO|KAF3810128.1 CGLO|KAF3810882.1 CHIG|XP_018150809.1 CHIG|XP_018151209.1 CHIG|XP_018152749.1 CHIG|XP_018152904.1 CHIG|XP_018154906.1 CHIG|XP_018155052.1 CHIG|XP_018155417.1 CHIG|XP_018156527.1 CHIG|XP_018156536.1 CHIG|XP_018156683.1 CHIG|XP_018157477.1 CHIG|XP_018157960.1 CHIG|XP_018159356.1 CHIG|XP_018160233.1 CHIG|XP_018161084.1 CHIG|XP_018161119.1 CHIG|XP_018161383.1 CHIG|XP_018164605.1 CHIG|XP_018165015.1 CHIG|XP_018165016.1 CVIN|KAF4902809.1 CVIN|KAF4910742.1 CVIN|KAF4911858.1 CVIN|KAF4912144.1 CVIN|KAF4912758.1 CVIN|KAF4916360.1 CVIN|KAF4918496.1 CVIN|KAF4918848.1 CVIN|KAF4918854.1 CVIN|KAF4919334.1 CVIN|KAF4919589.1 CVIN|KAF4920327.1 CVIN|KAF4920674.1 CVIN|KAF4922824.1 CVIN|KAF4923496.1 CVIN|KAF4928173.1 CVIN|KAF4928643.1 CVIN|KAF4930960.1 CVYL|A00325 CVYL|A01833 CVYL|A02262 CVYL|A02662 CVYL|A02719 CVYL|A03711 CVYL|A04109 CVYL|A06768 CVYL|A07747 CVYL|A07912 CVYL|A08184 CVYL|A09079 CVYL|A09115 CVYL|A09417 CVYL|A09860 CVYL|A10407 CVYL|A11119 FGRM|XP_011318231.1 FGRM|XP_011319978.1 FGRM|XP_011321440.1 FGRM|XP_011321815.1 FGRM|XP_011322136.1 FGRM|XP_011323237.1 FGRM|XP_011324179.1 FGRM|XP_011324331.1 FGRM|XP_011325265.1 FGRM|XP_011325843.1 FGRM|XP_011328115.1 MGRA|XP_003850560.1 MGRA|XP_003854164.1 MGRA|XP_003856898.1 MGRA|XP_003856911.1 MLAR|XP_007409999.1 MLAR|XP_007412745.1 MLAR|XP_007412778.1 MORY|QBZ53487.1 MORY|QBZ53541.1 MORY|QBZ56068.1 MORY|QBZ56621.1 MORY|QBZ63928.1 MORY|QBZ65339.1 MORY|QBZ66432.1 MORY|QBZ66433.1 NCRA|XP_011395299.1 NCRA|XP_957468.3 NCRA|XP_958171.3 NCRA|XP_960508.2 SSCL|APA05349.1 SSCL|APA06474.1 SSCL|APA07571.1 SSCL|APA08026.1 SSCL|APA10737.1 SSCL|APA12062.1 SSCL|APA14669.1 SSCL|APA14869.1

>Orthogroup8: ANID|CBF76241.1 ANID|CBF76690.1 ANID|CBF80682.1 ANID|CBF81700.1 ANID|CBF83918.1 ANID|CBF84486.1 ANID|CBF89418.1 ANID|CBF89972.1 BCIN|XP_001548851.1 BCIN|XP_024551001.1 BGRA|VCU40843.1 CFRU|XP_031876471.1 CFRU|XP_031877345.1 CFRU|XP_031879090.1 CFRU|XP_031879231.1 CFRU|XP_031879606.1 CFRU|XP_031879776.1 CFRU|XP_031880261.1 CFRU|XP_031880957.1 CFRU|XP_031881683.1 CFRU|XP_031884055.1 CFRU|XP_031884466.1 CFRU|XP_031885954.1 CFRU|XP_031886106.1 CFRU|XP_031886196.1 CFRU|XP_031887005.1 CFRU|XP_031888035.1 CFRU|XP_031888753.1 CFRU|XP_031889734.1 CFRU|XP_031890104.1 CFRU|XP_031890217.1 CFRU|XP_031891149.1 CFRU|XP_031891296.1 CFRU|XP_031891660.1 CFRU|XP_031891661.1 CFRU|XP_031891853.1 CFRU|XP_031892101.1 CFRU|XP_031892239.1 CFRU|XP_031892924.1 CGLO|KAF3797622.1 CGLO|KAF3798266.1 CGLO|KAF3798372.1 CGLO|KAF3798569.1 CGLO|KAF3798772.1 CGLO|KAF3799904.1 CGLO|KAF3801355.1 CGLO|KAF3801374.1 CGLO|KAF3802764.1 CGLO|KAF3804152.1 CGLO|KAF3804616.1 CGLO|KAF3805089.1 CGLO|KAF3805516.1 CGLO|KAF3807448.1 CGLO|KAF3809030.1 CGLO|KAF3809714.1 CGLO|KAF3809736.1 CGLO|KAF3810323.1 CGLO|KAF3810407.1 CGLO|KAF3810496.1 CGLO|KAF3811231.1 CGLO|KAF3811986.1 CHIG|XP_018152739.1 CHIG|XP_018153775.1 CHIG|XP_018154024.1 CHIG|XP_018154172.1 CHIG|XP_018155256.1 CHIG|XP_018155383.1 CHIG|XP_018155865.1 CHIG|XP_018156781.1 CHIG|XP_018158346.1 CHIG|XP_018159288.1 CHIG|XP_018159678.1 CHIG|XP_018160909.1 CHIG|XP_018161223.1 CHIG|XP_018164795.1 CHIG|XP_018164815.1 CHIG|XP_018164924.1 CVIN|KAF4895118.1 CVIN|KAF4897309.1 CVIN|KAF4903854.1 CVIN|KAF4903980.1 CVIN|KAF4907817.1 CVIN|KAF4910282.1 CVIN|KAF4910493.1 CVIN|KAF4912205.1 CVIN|KAF4912763.1 CVIN|KAF4913312.1 CVIN|KAF4915622.1 CVIN|KAF4916912.1 CVIN|KAF4918283.1 CVIN|KAF4918314.1 CVIN|KAF4918528.1 CVIN|KAF4921913.1 CVIN|KAF4921918.1 CVIN|KAF4922841.1 CVIN|KAF4923765.1 CVIN|KAF4924674.1 CVIN|KAF4927442.1 CVIN|KAF4929423.1 CVIN|KAF4930168.1 CVIN|KAF4930427.1 CVIN|KAF4931280.1 CVYL|A01609 CVYL|A03346 CVYL|A03774 CVYL|A04872 CVYL|A05521 CVYL|A05639 CVYL|A05657 CVYL|A05777 CVYL|A06301 CVYL|A06469 CVYL|A06637 CVYL|A06660 CVYL|A07707 CVYL|A07726 CVYL|A08091 CVYL|A08198 CVYL|A09150 CVYL|A09248 CVYL|A09617 CVYL|A09796 CVYL|A10956 CVYL|A11078 CVYL|A14100 FGRM|XP_011318293.1 FGRM|XP_011319690.1 FGRM|XP_011322798.1 FGRM|XP_011323108.1 FGRM|XP_011323278.1 FGRM|XP_011324312.1 FGRM|XP_011325270.1 FGRM|XP_011325727.1 FGRM|XP_011326068.1 MGRA|XP_003848636.1 MGRA|XP_003851735.1 MLAR|XP_007404301.1 MLAR|XP_007404302.1 MORY|QBZ53941.1 MORY|QBZ54943.1 MORY|QBZ56738.1 MORY|QBZ57869.1 MORY|QBZ58221.1 MORY|QBZ58470.1 MORY|QBZ60061.1 MORY|QBZ60745.1 MORY|QBZ63541.1 MORY|QBZ64320.1 NCRA|XP_958667.3 NCRA|XP_962567.2 NCRA|XP_963398.2 SSCL|APA11635.1 SSCL|APA15972.1

>Orthogroup9: BCIN|XP_001550524.2 BCIN|XP_001557416.2 CFRU|XP_031877571.1 CFRU|XP_031878482.1 CFRU|XP_031882428.1 FGRM|XP_011327644.1 MORY|QBZ57624.1 MORY|QBZ60185.1 SSCL|APA05390.1 SSCL|APA05507.1 SSCL|APA05595.1 SSCL|APA05653.1 SSCL|APA05702.1 SSCL|APA05703.1 SSCL|APA05842.1 SSCL|APA06035.1 SSCL|APA06036.1 SSCL|APA06070.1 SSCL|APA06113.1 SSCL|APA06228.1 SSCL|APA06283.1 SSCL|APA06391.1 SSCL|APA06567.1 SSCL|APA07162.1 SSCL|APA07164.1 SSCL|APA07191.1 SSCL|APA07240.1 SSCL|APA07321.1 SSCL|APA07421.1 SSCL|APA07461.1 SSCL|APA07500.1 SSCL|APA07544.1 SSCL|APA07643.1 SSCL|APA07644.1 SSCL|APA07651.1 SSCL|APA07664.1 SSCL|APA07679.1 SSCL|APA07723.1 SSCL|APA07899.1 SSCL|APA07999.1 SSCL|APA08028.1 SSCL|APA08178.1 SSCL|APA08490.1 SSCL|APA08524.1 SSCL|APA08686.1 SSCL|APA08690.1 SSCL|APA08691.1 SSCL|APA08874.1 SSCL|APA08899.1 SSCL|APA09133.1 SSCL|APA09250.1 SSCL|APA09260.1 SSCL|APA09261.1 SSCL|APA09265.1 SSCL|APA09324.1 SSCL|APA09329.1 SSCL|APA09356.1 SSCL|APA09357.1 SSCL|APA09367.1 SSCL|APA09475.1 SSCL|APA09565.1 SSCL|APA09566.1 SSCL|APA09579.1 SSCL|APA09827.1 SSCL|APA09829.1 SSCL|APA09832.1 SSCL|APA10059.1 SSCL|APA10084.1 SSCL|APA10130.1 SSCL|APA10190.1 SSCL|APA10207.1 SSCL|APA10233.1 SSCL|APA10234.1 SSCL|APA10279.1 SSCL|APA10280.1 SSCL|APA10377.1 SSCL|APA10378.1 SSCL|APA10425.1 SSCL|APA10432.1 SSCL|APA10615.1 SSCL|APA10729.1 SSCL|APA10741.1 SSCL|APA10744.1 SSCL|APA10753.1 SSCL|APA10756.1 SSCL|APA10757.1 SSCL|APA10916.1 SSCL|APA10917.1 SSCL|APA11244.1 SSCL|APA11330.1 SSCL|APA11339.1 SSCL|APA11352.1 SSCL|APA11411.1 SSCL|APA11414.1 SSCL|APA11491.1 SSCL|APA11595.1 SSCL|APA11745.1 SSCL|APA11912.1 SSCL|APA11929.1 SSCL|APA12031.1 SSCL|APA12582.1 SSCL|APA12900.1 SSCL|APA13175.1 SSCL|APA13179.1 SSCL|APA13267.1 SSCL|APA13448.1 SSCL|APA13454.1 SSCL|APA13623.1 SSCL|APA13657.1 SSCL|APA13781.1 SSCL|APA13988.1 SSCL|APA13989.1 SSCL|APA14001.1 SSCL|APA14076.1 SSCL|APA14077.1 SSCL|APA14078.1 SSCL|APA14091.1 SSCL|APA14100.1 SSCL|APA14250.1 SSCL|APA14283.1 SSCL|APA14436.1 SSCL|APA14487.1 SSCL|APA14492.1 SSCL|APA14493.1 SSCL|APA14522.1 SSCL|APA14546.1 SSCL|APA14556.1 SSCL|APA14877.1 SSCL|APA14933.1 SSCL|APA14950.1 SSCL|APA14951.1 SSCL|APA14978.1 SSCL|APA14985.1 SSCL|APA15105.1 SSCL|APA15109.1 SSCL|APA15330.1 SSCL|APA15478.1 SSCL|APA15479.1 SSCL|APA15585.1 SSCL|APA15586.1 SSCL|APA15587.1 SSCL|APA15656.1 SSCL|APA15697.1 SSCL|APA15698.1 SSCL|APA15753.1 SSCL|APA15760.1 SSCL|APA15761.1 SSCL|APA15815.1 SSCL|APA16027.1

>Orthogroup10: ANID|CBF73963.1 ANID|CBF73967.1 ANID|CBF80737.1 ANID|CBF87243.1 ANID|CBF87249.1 ANID|CBF87253.1 BGRA|VDB93572.1 CFRU|XP_031875597.1 CFRU|XP_031875802.1 CFRU|XP_031876413.1 CFRU|XP_031878198.1 CFRU|XP_031878326.1 CFRU|XP_031878685.1 CFRU|XP_031878866.1 CFRU|XP_031879077.1 CFRU|XP_031879339.1 CFRU|XP_031882997.1 CFRU|XP_031884468.1 CFRU|XP_031884571.1 CFRU|XP_031885904.1 CFRU|XP_031887329.1 CFRU|XP_031889552.1 CFRU|XP_031890893.1 CFRU|XP_031890904.1 CFRU|XP_031891703.1 CFRU|XP_031891970.1 CFRU|XP_031892078.1 CFRU|XP_031892161.1 CFRU|XP_031893577.1 CGLO|KAF3798054.1 CGLO|KAF3798806.1 CGLO|KAF3801401.1 CGLO|KAF3801429.1 CGLO|KAF3801508.1 CGLO|KAF3801782.1 CGLO|KAF3802598.1 CGLO|KAF3803842.1 CGLO|KAF3804758.1 CGLO|KAF3805487.1 CGLO|KAF3807951.1 CGLO|KAF3808533.1 CGLO|KAF3809391.1 CGLO|KAF3809508.1 CGLO|KAF3809789.1 CGLO|KAF3810329.1 CGLO|KAF3810343.1 CGLO|KAF3810644.1 CGLO|KAF3811758.1 CHIG|XP_018151549.1 CHIG|XP_018151886.1 CHIG|XP_018152619.1 CHIG|XP_018152652.1 CHIG|XP_018153109.1 CHIG|XP_018153374.1 CHIG|XP_018155045.1 CHIG|XP_018155790.1 CHIG|XP_018156715.1 CHIG|XP_018158102.1 CHIG|XP_018158103.1 CHIG|XP_018159286.1 CHIG|XP_018161301.1 CHIG|XP_018161564.1 CHIG|XP_018162664.1 CHIG|XP_018163103.1 CHIG|XP_018164957.1 CVIN|KAF4893027.1 CVIN|KAF4896242.1 CVIN|KAF4896245.1 CVIN|KAF4899862.1 CVIN|KAF4906646.1 CVIN|KAF4912213.1 CVIN|KAF4913926.1 CVIN|KAF4915190.1 CVIN|KAF4916381.1 CVIN|KAF4916999.1 CVIN|KAF4917628.1 CVIN|KAF4917806.1 CVIN|KAF4918560.1 CVIN|KAF4919574.1 CVIN|KAF4919593.1 CVIN|KAF4921934.1 CVIN|KAF4922366.1 CVIN|KAF4924062.1 CVIN|KAF4926165.1 CVIN|KAF4926177.1 CVIN|KAF4928016.1 CVYL|A00675 CVYL|A01885 CVYL|A02382 CVYL|A02919 CVYL|A05615 CVYL|A05763 CVYL|A06711 CVYL|A07656 CVYL|A07681 CVYL|A08089 CVYL|A08302 CVYL|A08958 CVYL|A09124 CVYL|A11609 CVYL|A12372 CVYL|A12374 CVYL|A12623 CVYL|A12926 CVYL|A13728 FGRM|XP_011315667.1 FGRM|XP_011318623.1 FGRM|XP_011320625.1 FGRM|XP_011323155.1 FGRM|XP_011325368.1 MGRA|XP_003849190.1 MGRA|XP_003849583.1 MGRA|XP_003849820.1 MGRA|XP_003850543.1 MGRA|XP_003850661.1 MLAR|XP_007418154.1 MORY|QBZ53330.1 MORY|QBZ53401.1 MORY|QBZ53622.1 MORY|QBZ54796.1 MORY|QBZ56538.1 MORY|QBZ57598.1 MORY|QBZ57776.1 MORY|QBZ57777.1 MORY|QBZ57856.1 MORY|QBZ61960.1 MORY|QBZ63043.1 MORY|QBZ63370.1 MORY|QBZ64418.1 MORY|QBZ64942.1 MORY|QBZ65324.1 MORY|QBZ65326.1 MORY|QBZ65333.1 MORY|QBZ65505.1 MORY|QBZ65518.1 MORY|QBZ66584.1 MORY|QBZ66640.1 NCRA|XP_963189.1

>Orthogroup11: ANID|CBF70149.1 ANID|CBF70628.1 ANID|CBF74267.1 ANID|CBF77069.1 ANID|CBF77962.1 ANID|CBF82389.1 ANID|CBF82870.1 ANID|CBF85157.1 ANID|CBF85456.1 ANID|CBF86126.1 BCIN|XP_001547953.2 BCIN|XP_001552845.1 BCIN|XP_001554294.1 BCIN|XP_001557266.1 BCIN|XP_001557558.1 BCIN|XP_024546813.1 BCIN|XP_024548023.1 BCIN|XP_024550238.1 BCIN|XP_024550535.1 BGRA|VDB92912.1 CFRU|XP_031876258.1 CFRU|XP_031877238.1 CFRU|XP_031878679.1 CFRU|XP_031878747.1 CFRU|XP_031879679.1 CFRU|XP_031879743.1 CFRU|XP_031880242.1 CFRU|XP_031880686.1 CFRU|XP_031881094.1 CFRU|XP_031882596.1 CFRU|XP_031883416.1 CFRU|XP_031884230.1 CFRU|XP_031891117.1 CFRU|XP_031891405.1 CFRU|XP_031891419.1 CFRU|XP_031893123.1 CGLO|KAF3798165.1 CGLO|KAF3798176.1 CGLO|KAF3798177.1 CGLO|KAF3798365.1 CGLO|KAF3800427.1 CGLO|KAF3801743.1 CGLO|KAF3803838.1 CGLO|KAF3803919.1 CGLO|KAF3804610.1 CGLO|KAF3804751.1 CGLO|KAF3805662.1 CGLO|KAF3806847.1 CGLO|KAF3808085.1 CGLO|KAF3808959.1 CGLO|KAF3809924.1 CGLO|KAF3811054.1 CGLO|KAF3811770.1 CHIG|XP_018151027.1 CHIG|XP_018152788.1 CHIG|XP_018159820.1 CHIG|XP_018161507.1 CHIG|XP_018161681.1 CHIG|XP_018161848.1 CHIG|XP_018164669.1 CVIN|KAF4896547.1 CVIN|KAF4901302.1 CVIN|KAF4906642.1 CVIN|KAF4907632.1 CVIN|KAF4918141.1 CVIN|KAF4919076.1 CVIN|KAF4919116.1 CVIN|KAF4919133.1 CVIN|KAF4920622.1 CVIN|KAF4922932.1 CVIN|KAF4923573.1 CVIN|KAF4925436.1 CVIN|KAF4925749.1 CVIN|KAF4930673.1 CVYL|A01701 CVYL|A01819 CVYL|A02495 CVYL|A04943 CVYL|A04952 CVYL|A05271 CVYL|A07535 CVYL|A08147 CVYL|A08883 CVYL|A08962 CVYL|A09002 CVYL|A09738 CVYL|A11766 CVYL|A12777 CVYL|A12958 FGRM|XP_011317501.1 FGRM|XP_011321598.1 FGRM|XP_011322318.1 FGRM|XP_011325760.1 FGRM|XP_011326001.1 FGRM|XP_011326118.1 MGRA|XP_003847822.1 MGRA|XP_003848777.1 MGRA|XP_003851793.1 MGRA|XP_003852644.1 MGRA|XP_003853200.1 MGRA|XP_003853839.1 MGRA|XP_003854048.1 MGRA|XP_003856233.1 MLAR|XP_007406619.1 MLAR|XP_007410229.1 MLAR|XP_007410231.1 MLAR|XP_007411484.1 MLAR|XP_007411669.1 MLAR|XP_007413214.1 MLAR|XP_007413393.1 MLAR|XP_007413394.1 MLAR|XP_007418332.1 MLAR|XP_007418647.1 MORY|QBZ56963.1 MORY|QBZ58057.1 MORY|QBZ58258.1 MORY|QBZ59476.1 MORY|QBZ60277.1 MORY|QBZ61942.1 MORY|QBZ64479.1 MORY|QBZ65829.1 NCRA|XP_001728277.1 NCRA|XP_957037.2 NCRA|XP_960312.2 NCRA|XP_961872.3 NCRA|XP_964555.1 SSCL|APA06077.1 SSCL|APA07618.1 SSCL|APA08310.1 SSCL|APA10911.1 SSCL|APA11319.1 SSCL|APA12340.1 SSCL|APA13599.1 SSCL|APA14383.1

>Orthogroup12: ANID|CBF70661.1 ANID|CBF71384.1 ANID|CBF75894.1 ANID|CBF76097.1 BCIN|XP_001549402.1 BCIN|XP_024549664.1 BCIN|XP_024551246.1 CFRU|XP_031878451.1 CFRU|XP_031880240.1 CFRU|XP_031880564.1 CFRU|XP_031880565.1 CFRU|XP_031880898.1 CFRU|XP_031881790.1 CFRU|XP_031883121.1 CFRU|XP_031885119.1 CFRU|XP_031885121.1 CFRU|XP_031885122.1 CFRU|XP_031887884.1 CFRU|XP_031889438.1 CFRU|XP_031892104.1 CFRU|XP_031892157.1 CFRU|XP_031892838.1 CFRU|XP_031892916.1 CGLO|KAF3798615.1 CGLO|KAF3800697.1 CGLO|KAF3801368.1 CGLO|KAF3802018.1 CGLO|KAF3806975.1 CGLO|KAF3807763.1 CGLO|KAF3808203.1 CGLO|KAF3808922.1 CGLO|KAF3808956.1 CGLO|KAF3809112.1 CGLO|KAF3809554.1 CGLO|KAF3809731.1 CGLO|KAF3811556.1 CHIG|XP_018151077.1 CHIG|XP_018154152.1 CHIG|XP_018155770.1 CHIG|XP_018155932.1 CHIG|XP_018160817.1 CHIG|XP_018161954.1 CHIG|XP_018162507.1 CHIG|XP_018162953.1 CHIG|XP_018163027.1 CHIG|XP_018163028.1 CHIG|XP_018163210.1 CVIN|KAF4900987.1 CVIN|KAF4903859.1 CVIN|KAF4913307.1 CVIN|KAF4914251.1 CVIN|KAF4918775.1 CVIN|KAF4920547.1 CVIN|KAF4920549.1 CVIN|KAF4920550.1 CVIN|KAF4920729.1 CVIN|KAF4920752.1 CVIN|KAF4922102.1 CVIN|KAF4924282.1 CVIN|KAF4925182.1 CVIN|KAF4930108.1 CVYL|A00552 CVYL|A00832 CVYL|A00833 CVYL|A00834 CVYL|A01140 CVYL|A01163 CVYL|A01296 CVYL|A03370 CVYL|A04769 CVYL|A04830 CVYL|A06268 CVYL|A06655 CVYL|A07713 CVYL|A13022 FGRM|XP_011317433.1 FGRM|XP_011317579.1 FGRM|XP_011319138.1 FGRM|XP_011321382.1 FGRM|XP_011321849.1 FGRM|XP_011321900.1 FGRM|XP_011321917.1 FGRM|XP_011322572.1 FGRM|XP_011322778.1 FGRM|XP_011322809.1 FGRM|XP_011323287.1 FGRM|XP_011324413.1 FGRM|XP_011324430.1 FGRM|XP_011324841.1 FGRM|XP_011325160.1 FGRM|XP_011325246.1 FGRM|XP_011325247.1 FGRM|XP_011325284.1 FGRM|XP_011325854.1 FGRM|XP_011327407.1 FGRM|XP_011327453.1 FGRM|XP_011327494.1 FGRM|XP_011327517.1 FGRM|XP_011328703.1 MGRA|XP_003852284.1 MGRA|XP_003855735.1 MGRA|XP_003855995.1 MORY|QBZ57248.1 MORY|QBZ57364.1 NCRA|XP_001728518.2 NCRA|XP_011392801.1 NCRA|XP_011395209.1 NCRA|XP_956770.1 NCRA|XP_957118.2 NCRA|XP_957418.1 NCRA|XP_957775.1 NCRA|XP_957841.1 NCRA|XP_958236.3 NCRA|XP_959016.1 NCRA|XP_959353.3 NCRA|XP_959354.2 NCRA|XP_959355.2 NCRA|XP_959357.3 NCRA|XP_959358.1 NCRA|XP_959365.1 NCRA|XP_960091.1 NCRA|XP_960101.1 NCRA|XP_960102.1 NCRA|XP_960268.3 NCRA|XP_960315.2 NCRA|XP_961055.3 NCRA|XP_962936.1 SSCL|APA06180.1 SSCL|APA10092.1 SSCL|APA11712.1

>Orthogroup13: BGRA|VCU39838.1 BGRA|VCU39839.1 BGRA|VCU39852.1 BGRA|VCU39853.1 BGRA|VCU39854.1 BGRA|VCU39855.1 BGRA|VCU39856.1 BGRA|VCU39874.1 BGRA|VCU39892.1 BGRA|VCU40211.1 BGRA|VCU40221.1 BGRA|VCU40222.1 BGRA|VCU40223.1 BGRA|VCU40227.1 BGRA|VCU40235.1 BGRA|VCU40254.1 BGRA|VCU40260.1 BGRA|VCU40268.1 BGRA|VCU40272.1 BGRA|VCU40278.1 BGRA|VCU40282.1 BGRA|VCU40284.1 BGRA|VCU40285.1 BGRA|VCU40288.1 BGRA|VCU40290.1 BGRA|VCU40294.1 BGRA|VCU40295.1 BGRA|VCU40296.1 BGRA|VCU40297.1 BGRA|VCU40302.1 BGRA|VCU40306.1 BGRA|VCU40315.1 BGRA|VCU40318.1 BGRA|VCU40320.1 BGRA|VCU40322.1 BGRA|VCU40326.1 BGRA|VCU40330.1 BGRA|VCU40348.1 BGRA|VCU40372.1 BGRA|VCU40376.1 BGRA|VCU40387.1 BGRA|VCU40388.1 BGRA|VCU40400.1 BGRA|VCU40401.1 BGRA|VCU40438.1 BGRA|VCU40878.1 BGRA|VCU40880.1 BGRA|VCU40881.1 BGRA|VCU41054.1 BGRA|VCU41105.1 BGRA|VCU41106.1 BGRA|VCU41109.1 BGRA|VCU41110.1 BGRA|VDB83485.1 BGRA|VDB83486.1 BGRA|VDB83488.1 BGRA|VDB83489.1 BGRA|VDB83492.1 BGRA|VDB83499.1 BGRA|VDB83500.1 BGRA|VDB83508.1 BGRA|VDB83510.1 BGRA|VDB83514.1 BGRA|VDB83523.1 BGRA|VDB83524.1 BGRA|VDB83538.1 BGRA|VDB83544.1 BGRA|VDB83549.1 BGRA|VDB83550.1 BGRA|VDB83553.1 BGRA|VDB83554.1 BGRA|VDB83570.1 BGRA|VDB83571.1 BGRA|VDB83572.1 BGRA|VDB83583.1 BGRA|VDB83584.1 BGRA|VDB83586.1 BGRA|VDB83589.1 BGRA|VDB83597.1 BGRA|VDB83600.1 BGRA|VDB83601.1 BGRA|VDB83614.1 BGRA|VDB83615.1 BGRA|VDB83616.1 BGRA|VDB83617.1 BGRA|VDB83802.1 BGRA|VDB83804.1 BGRA|VDB83826.1 BGRA|VDB83829.1 BGRA|VDB85789.1 BGRA|VDB86285.1 BGRA|VDB88693.1 BGRA|VDB88710.1 BGRA|VDB88712.1 BGRA|VDB88716.1 BGRA|VDB88721.1 BGRA|VDB88723.1 BGRA|VDB89062.1 BGRA|VDB89063.1 BGRA|VDB89841.1 BGRA|VDB89842.1 BGRA|VDB89845.1 BGRA|VDB89856.1 BGRA|VDB89864.1 BGRA|VDB89871.1 BGRA|VDB89878.1 BGRA|VDB89906.1 BGRA|VDB89908.1 BGRA|VDB89918.1 BGRA|VDB89924.1 BGRA|VDB89926.1 BGRA|VDB89942.1 BGRA|VDB89948.1 BGRA|VDB89951.1 BGRA|VDB89954.1 BGRA|VDB90401.1 BGRA|VDB90535.1 BGRA|VDB90537.1 BGRA|VDB93975.1 BGRA|VDB95227.1 BGRA|VDB95347.1 BGRA|VDB95349.1 BGRA|VDB95350.1 BGRA|VDB96498.1 BGRA|VDB96503.1 BGRA|VDB96504.1 BGRA|VDB96505.1 BGRA|VDB96509.1 BGRA|VDB96511.1 BGRA|VDB96512.1

>Orthogroup14: BGRA|VCU38865.1 BGRA|VCU38878.1 BGRA|VCU38895.1 BGRA|VCU38978.1 BGRA|VCU38999.1 BGRA|VCU39010.1 BGRA|VCU39172.1 BGRA|VCU39181.1 BGRA|VCU39247.1 BGRA|VCU39322.1 BGRA|VCU39386.1 BGRA|VCU39431.1 BGRA|VCU39438.1 BGRA|VCU39597.1 BGRA|VCU39827.1 BGRA|VCU39963.1 BGRA|VCU39992.1 BGRA|VCU40005.1 BGRA|VCU40035.1 BGRA|VCU40267.1 BGRA|VCU40325.1 BGRA|VCU40568.1 BGRA|VCU40605.1 BGRA|VCU40692.1 BGRA|VCU40694.1 BGRA|VCU40710.1 BGRA|VCU40771.1 BGRA|VCU40777.1 BGRA|VCU40797.1 BGRA|VCU40898.1 BGRA|VCU40989.1 BGRA|VCU41014.1 BGRA|VCU41170.1 BGRA|VCU41188.1 BGRA|VCU41310.1 BGRA|VCU41403.1 BGRA|VDB83585.1 BGRA|VDB83644.1 BGRA|VDB83857.1 BGRA|VDB83859.1 BGRA|VDB83902.1 BGRA|VDB83988.1 BGRA|VDB84061.1 BGRA|VDB84139.1 BGRA|VDB84173.1 BGRA|VDB84223.1 BGRA|VDB84348.1 BGRA|VDB84384.1 BGRA|VDB85745.1 BGRA|VDB85780.1 BGRA|VDB85928.1 BGRA|VDB85986.1 BGRA|VDB85991.1 BGRA|VDB85997.1 BGRA|VDB86009.1 BGRA|VDB86216.1 BGRA|VDB86288.1 BGRA|VDB86307.1 BGRA|VDB87610.1 BGRA|VDB87635.1 BGRA|VDB87693.1 BGRA|VDB87741.1 BGRA|VDB87759.1 BGRA|VDB87844.1 BGRA|VDB87846.1 BGRA|VDB87856.1 BGRA|VDB88067.1 BGRA|VDB88119.1 BGRA|VDB88169.1 BGRA|VDB88386.1 BGRA|VDB88757.1 BGRA|VDB88990.1 BGRA|VDB89118.1 BGRA|VDB89495.1 BGRA|VDB89686.1 BGRA|VDB89857.1 BGRA|VDB89859.1 BGRA|VDB89863.1 BGRA|VDB89866.1 BGRA|VDB89867.1 BGRA|VDB89870.1 BGRA|VDB89873.1 BGRA|VDB89876.1 BGRA|VDB90335.1 BGRA|VDB90441.1 BGRA|VDB90469.1 BGRA|VDB90492.1 BGRA|VDB90504.1 BGRA|VDB91113.1 BGRA|VDB91200.1 BGRA|VDB91223.1 BGRA|VDB91293.1 BGRA|VDB92686.1 BGRA|VDB92750.1 BGRA|VDB92859.1 BGRA|VDB92901.1 BGRA|VDB93090.1 BGRA|VDB93130.1 BGRA|VDB93167.1 BGRA|VDB93240.1 BGRA|VDB93260.1 BGRA|VDB93285.1 BGRA|VDB93296.1 BGRA|VDB93297.1 BGRA|VDB93603.1 BGRA|VDB93684.1 BGRA|VDB93717.1 BGRA|VDB93758.1 BGRA|VDB93979.1 BGRA|VDB93986.1 BGRA|VDB94467.1 BGRA|VDB94619.1 BGRA|VDB94704.1 BGRA|VDB94744.1 BGRA|VDB94933.1 BGRA|VDB94935.1 BGRA|VDB94973.1 BGRA|VDB95008.1 BGRA|VDB95033.1 BGRA|VDB95095.1 BGRA|VDB95146.1 BGRA|VDB96292.1 BGRA|VDB96300.1 BGRA|VDB96384.1 BGRA|VDB96515.1 BGRA|VDB96517.1

>Orthogroup15: ANID|CBF69517.1 ANID|CBF78469.1 ANID|CBF81501.1 ANID|CBF82793.1 ANID|CBF84417.1 ANID|CBF85199.1 ANID|CBF87153.1 BCIN|XP_024548684.1 BCIN|XP_024553313.1 CFRU|XP_031877232.1 CFRU|XP_031877965.1 CFRU|XP_031880937.1 CFRU|XP_031881311.1 CFRU|XP_031881400.1 CFRU|XP_031881418.1 CFRU|XP_031882223.1 CFRU|XP_031884403.1 CFRU|XP_031885245.1 CFRU|XP_031887443.1 CFRU|XP_031887531.1 CFRU|XP_031888803.1 CFRU|XP_031889113.1 CFRU|XP_031889231.1 CFRU|XP_031889973.1 CFRU|XP_031890942.1 CFRU|XP_031891410.1 CFRU|XP_031891647.1 CFRU|XP_031892667.1 CGLO|KAF3799267.1 CGLO|KAF3799698.1 CGLO|KAF3801571.1 CGLO|KAF3802796.1 CGLO|KAF3802797.1 CGLO|KAF3802852.1 CGLO|KAF3804352.1 CGLO|KAF3804726.1 CGLO|KAF3804745.1 CGLO|KAF3805741.1 CGLO|KAF3806791.1 CGLO|KAF3806854.1 CGLO|KAF3807743.1 CGLO|KAF3809169.1 CGLO|KAF3809190.1 CGLO|KAF3810963.1 CGLO|KAF3810988.1 CHIG|XP_018151602.1 CHIG|XP_018151624.1 CHIG|XP_018151975.1 CHIG|XP_018152973.1 CHIG|XP_018153265.1 CHIG|XP_018155992.1 CHIG|XP_018158448.1 CHIG|XP_018159893.1 CHIG|XP_018160122.1 CHIG|XP_018161150.1 CHIG|XP_018161802.1 CHIG|XP_018163035.1 CHIG|XP_018163633.1 CVIN|KAF4903554.1 CVIN|KAF4904500.1 CVIN|KAF4907242.1 CVIN|KAF4910471.1 CVIN|KAF4918362.1 CVIN|KAF4919818.1 CVIN|KAF4920542.1 CVIN|KAF4921094.1 CVIN|KAF4921821.1 CVIN|KAF4923349.1 CVIN|KAF4925305.1 CVIN|KAF4926580.1 CVIN|KAF4926594.1 CVIN|KAF4928297.1 CVIN|KAF4928690.1 CVYL|A01049 CVYL|A01356 CVYL|A02166 CVYL|A02186 CVYL|A02551 CVYL|A03738 CVYL|A04044 CVYL|A05161 CVYL|A05375 CVYL|A05394 CVYL|A06596 CVYL|A09824 CVYL|A09889 CVYL|A10158 CVYL|A12566 FGRM|XP_011318036.1 FGRM|XP_011318285.1 FGRM|XP_011321058.1 FGRM|XP_011321771.1 FGRM|XP_011321987.1 FGRM|XP_011324414.1 FGRM|XP_011327664.1 MGRA|XP_003847603.1 MGRA|XP_003848315.1 MGRA|XP_003848452.1 MGRA|XP_003848461.1 MGRA|XP_003850037.1 MGRA|XP_003853284.1 MGRA|XP_003853948.1 MORY|QBZ54067.1 MORY|QBZ55172.1 MORY|QBZ55215.1 MORY|QBZ56728.1 MORY|QBZ58128.1 MORY|QBZ58141.1 MORY|QBZ59990.1 MORY|QBZ61736.1 MORY|QBZ65018.1 MORY|QBZ65233.1 MORY|QBZ65781.1 MORY|QBZ66353.1 MORY|QBZ66528.1 NCRA|XP_011394798.1 NCRA|XP_956434.2 NCRA|XP_961803.1 SSCL|APA08632.1 SSCL|APA12577.1 SSCL|APA13624.1

>Orthogroup16: ANID|CBF70118.1 ANID|CBF76209.1 ANID|CBF80376.1 ANID|CBF82292.1 ANID|CBF82795.1 ANID|CBF84139.1 ANID|CBF85201.1 BCIN|XP_024547861.1 BCIN|XP_024550123.1 CFRU|XP_031877979.1 CFRU|XP_031878218.1 CFRU|XP_031878628.1 CFRU|XP_031881398.1 CFRU|XP_031881410.1 CFRU|XP_031881451.1 CFRU|XP_031881515.1 CFRU|XP_031881516.1 CFRU|XP_031883717.1 CFRU|XP_031884536.1 CFRU|XP_031884544.1 CFRU|XP_031885057.1 CFRU|XP_031885249.1 CFRU|XP_031887451.1 CFRU|XP_031888131.1 CFRU|XP_031888812.1 CFRU|XP_031889273.1 CFRU|XP_031889319.1 CFRU|XP_031889384.1 CFRU|XP_031891191.1 CFRU|XP_031893329.1 CFRU|XP_031893485.1 CGLO|KAF3798394.1 CGLO|KAF3798475.1 CGLO|KAF3799095.1 CGLO|KAF3799756.1 CGLO|KAF3800936.1 CGLO|KAF3803096.1 CGLO|KAF3804221.1 CGLO|KAF3804583.1 CGLO|KAF3805778.1 CGLO|KAF3807133.1 CGLO|KAF3807705.1 CGLO|KAF3807755.1 CGLO|KAF3808053.1 CGLO|KAF3808512.1 CGLO|KAF3808868.1 CGLO|KAF3810961.1 CHIG|XP_018153917.1 CHIG|XP_018154890.1 CHIG|XP_018158316.1 CHIG|XP_018159382.1 CHIG|XP_018159425.1 CHIG|XP_018160169.1 CHIG|XP_018160941.1 CHIG|XP_018161964.1 CHIG|XP_018162932.1 CHIG|XP_018163119.1 CHIG|XP_018163321.1 CVIN|KAF4896682.1 CVIN|KAF4907244.1 CVIN|KAF4916349.1 CVIN|KAF4917709.1 CVIN|KAF4918860.1 CVIN|KAF4920179.1 CVIN|KAF4920901.1 CVIN|KAF4922828.1 CVIN|KAF4922830.1 CVIN|KAF4923061.1 CVIN|KAF4926578.1 CVIN|KAF4926598.1 CVIN|KAF4926626.1 CVIN|KAF4929612.1 CVIN|KAF4930092.1 CVYL|A00988 CVYL|A01213 CVYL|A02187 CVYL|A02267 CVYL|A03531 CVYL|A03895 CVYL|A05297 CVYL|A05968 CVYL|A06042 CVYL|A06047 CVYL|A07164 CVYL|A08179 CVYL|A08210 CVYL|A09803 CVYL|A11340 CVYL|A11626 FGRM|XP_011315586.1 FGRM|XP_011319569.1 FGRM|XP_011321994.1 FGRM|XP_011322970.1 FGRM|XP_011323609.1 FGRM|XP_011325845.1 FGRM|XP_011328706.1 MGRA|XP_003851433.1 MLAR|XP_007403805.1 MLAR|XP_007405397.1 MLAR|XP_007406696.1 MLAR|XP_007408539.1 MLAR|XP_007415554.1 MORY|QBZ53625.1 MORY|QBZ58062.1 MORY|QBZ61224.1 MORY|QBZ61631.1 MORY|QBZ62032.1 MORY|QBZ65100.1 NCRA|XP_001728158.1 NCRA|XP_961041.2 NCRA|XP_964142.1 SSCL|APA08183.1 SSCL|APA10588.1

>Orthogroup17: ANID|CBF71241.1 ANID|CBF78087.1 ANID|CBF78809.1 ANID|CBF79162.1 ANID|CBF80142.1 ANID|CBF80752.1 ANID|CBF81971.1 ANID|CBF82224.1 ANID|CBF84010.1 ANID|CBF84011.1 ANID|CBF85739.1 ANID|CBF87325.1 ANID|CBF90352.1 BCIN|XP_024550751.1 BCIN|XP_024551271.1 BCIN|XP_024551593.1 BGRA|VDB89307.1 CFRU|XP_031877300.1 CFRU|XP_031878965.1 CFRU|XP_031879945.1 CFRU|XP_031881058.1 CFRU|XP_031882945.1 CFRU|XP_031882984.1 CFRU|XP_031883653.1 CFRU|XP_031886587.1 CFRU|XP_031888740.1 CFRU|XP_031889019.1 CFRU|XP_031889072.1 CFRU|XP_031889836.1 CFRU|XP_031889908.1 CFRU|XP_031890650.1 CFRU|XP_031891758.1 CFRU|XP_031892208.1 CGLO|KAF3799251.1 CGLO|KAF3799947.1 CGLO|KAF3800106.1 CGLO|KAF3800171.1 CGLO|KAF3800761.1 CGLO|KAF3803376.1 CGLO|KAF3804096.1 CGLO|KAF3804185.1 CGLO|KAF3804193.1 CGLO|KAF3804916.1 CGLO|KAF3805190.1 CGLO|KAF3806840.1 CGLO|KAF3809856.1 CGLO|KAF3810431.1 CGLO|KAF3810437.1 CGLO|KAF3811919.1 CHIG|XP_018159183.1 CHIG|XP_018162658.1 CHIG|XP_018163083.1 CHIG|XP_018163120.1 CHIG|XP_018163568.1 CHIG|XP_018163953.1 CVIN|KAF4894845.1 CVIN|KAF4895621.1 CVIN|KAF4895627.1 CVIN|KAF4905163.1 CVIN|KAF4908846.1 CVIN|KAF4909297.1 CVIN|KAF4915991.1 CVIN|KAF4918388.1 CVIN|KAF4918406.1 CVIN|KAF4919422.1 CVIN|KAF4919661.1 CVIN|KAF4922596.1 CVIN|KAF4924655.1 CVIN|KAF4924672.1 CVIN|KAF4925799.1 CVIN|KAF4925849.1 CVYL|A00443 CVYL|A00724 CVYL|A02487 CVYL|A03861 CVYL|A03868 CVYL|A06144 CVYL|A07059 CVYL|A07963 CVYL|A10014 CVYL|A10144 CVYL|A12141 CVYL|A13745 CVYL|A14303 FGRM|XP_011318280.1 FGRM|XP_011322603.1 FGRM|XP_011323076.1 FGRM|XP_011323156.1 FGRM|XP_011325977.1 FGRM|XP_011328667.1 MGRA|XP_003850557.1 MGRA|XP_003851211.1 MGRA|XP_003851331.1 MGRA|XP_003853008.1 MGRA|XP_003853838.1 MGRA|XP_003854079.1 MGRA|XP_003856451.1 MGRA|XP_003856758.1 MORY|QBZ53336.1 MORY|QBZ55549.1 MORY|QBZ59934.1 MORY|QBZ61196.1 MORY|QBZ63343.1 NCRA|XP_960015.3 NCRA|XP_963608.1 NCRA|XP_963895.3 NCRA|XP_965148.2 SSCL|APA10034.1 SSCL|APA10273.1 SSCL|APA10274.1 SSCL|APA10779.1 SSCL|APA11665.1 SSCL|APA14853.1

>Orthogroup18: ANID|CBF73441.1 ANID|CBF73982.1 ANID|CBF80212.1 ANID|CBF87870.1 ANID|CBF87872.1 ANID|CBF90374.1 BCIN|XP_001549486.1 BCIN|XP_024545910.1 BCIN|XP_024547198.1 BCIN|XP_024547539.1 BCIN|XP_024547565.1 BCIN|XP_024549433.1 BCIN|XP_024550314.1 BCIN|XP_024553850.1 CFRU|XP_031877250.1 CFRU|XP_031878233.1 CFRU|XP_031878946.1 CFRU|XP_031880440.1 CFRU|XP_031881651.1 CFRU|XP_031882657.1 CFRU|XP_031883517.1 CFRU|XP_031884191.1 CFRU|XP_031884203.1 CFRU|XP_031884419.1 CFRU|XP_031885312.1 CFRU|XP_031885809.1 CFRU|XP_031885906.1 CFRU|XP_031885977.1 CFRU|XP_031886234.1 CFRU|XP_031889920.1 CFRU|XP_031892809.1 CGLO|KAF3798626.1 CGLO|KAF3802548.1 CGLO|KAF3803885.1 CGLO|KAF3805355.1 CGLO|KAF3806390.1 CGLO|KAF3806828.1 CGLO|KAF3807197.1 CGLO|KAF3807302.1 CGLO|KAF3807888.1 CGLO|KAF3808490.1 CGLO|KAF3810366.1 CGLO|KAF3810805.1 CGLO|KAF3811013.1 CGLO|KAF3811027.1 CHIG|XP_018151518.1 CHIG|XP_018152576.1 CHIG|XP_018152988.1 CHIG|XP_018155223.1 CHIG|XP_018156596.1 CHIG|XP_018156608.1 CHIG|XP_018156756.1 CHIG|XP_018157574.1 CHIG|XP_018157883.1 CHIG|XP_018157884.1 CHIG|XP_018158937.1 CHIG|XP_018159434.1 CHIG|XP_018159522.1 CHIG|XP_018162262.1 CVIN|KAF4909114.1 CVIN|KAF4909279.1 CVIN|KAF4911883.1 CVIN|KAF4917738.1 CVIN|KAF4918101.1 CVIN|KAF4918158.1 CVIN|KAF4921315.1 CVIN|KAF4921761.1 CVIN|KAF4922595.1 CVIN|KAF4923238.1 CVIN|KAF4925460.1 CVIN|KAF4926898.1 CVIN|KAF4928148.1 CVIN|KAF4929096.1 CVYL|A00725 CVYL|A01132 CVYL|A02473 CVYL|A02832 CVYL|A04821 CVYL|A05747 CVYL|A08916 CVYL|A09334 CVYL|A09759 CVYL|A09774 CVYL|A10676 CVYL|A10788 CVYL|A11004 CVYL|A11650 CVYL|A11980 CVYL|A13769 FGRM|XP_011315635.1 FGRM|XP_011318019.1 FGRM|XP_011318225.1 FGRM|XP_011319545.1 FGRM|XP_011320483.1 FGRM|XP_011324771.1 FGRM|XP_011325375.1 FGRM|XP_011327997.1 MGRA|XP_003850387.1 MGRA|XP_003851594.1 MGRA|XP_003853384.1 MGRA|XP_003854925.1 MGRA|XP_003855812.1 MGRA|XP_003855817.1 MORY|QBZ59332.1 MORY|QBZ59369.1 NCRA|XP_961122.3 NCRA|XP_964673.2 SSCL|APA07325.1 SSCL|APA10978.1 SSCL|APA11830.1 SSCL|APA14126.1

>Orthogroup19: MLAR|XP_007403835.1 MLAR|XP_007403836.1 MLAR|XP_007404047.1 MLAR|XP_007404269.1 MLAR|XP_007404513.1 MLAR|XP_007404617.1 MLAR|XP_007404618.1 MLAR|XP_007404621.1 MLAR|XP_007404662.1 MLAR|XP_007404674.1 MLAR|XP_007404762.1 MLAR|XP_007404806.1 MLAR|XP_007404922.1 MLAR|XP_007405069.1 MLAR|XP_007405284.1 MLAR|XP_007405441.1 MLAR|XP_007405599.1 MLAR|XP_007405615.1 MLAR|XP_007406057.1 MLAR|XP_007406252.1 MLAR|XP_007406938.1 MLAR|XP_007406953.1 MLAR|XP_007407157.1 MLAR|XP_007407179.1 MLAR|XP_007407181.1 MLAR|XP_007407201.1 MLAR|XP_007407233.1 MLAR|XP_007407339.1 MLAR|XP_007407763.1 MLAR|XP_007407861.1 MLAR|XP_007408058.1 MLAR|XP_007408109.1 MLAR|XP_007408382.1 MLAR|XP_007408415.1 MLAR|XP_007408499.1 MLAR|XP_007408582.1 MLAR|XP_007408583.1 MLAR|XP_007408584.1 MLAR|XP_007408618.1 MLAR|XP_007408785.1 MLAR|XP_007408921.1 MLAR|XP_007408977.1 MLAR|XP_007409040.1 MLAR|XP_007409635.1 MLAR|XP_007409708.1 MLAR|XP_007409808.1 MLAR|XP_007409861.1 MLAR|XP_007409906.1 MLAR|XP_007409971.1 MLAR|XP_007409987.1 MLAR|XP_007410881.1 MLAR|XP_007410882.1 MLAR|XP_007411076.1 MLAR|XP_007411144.1 MLAR|XP_007411145.1 MLAR|XP_007411585.1 MLAR|XP_007411662.1 MLAR|XP_007411663.1 MLAR|XP_007411778.1 MLAR|XP_007411951.1 MLAR|XP_007412305.1 MLAR|XP_007412306.1 MLAR|XP_007412496.1 MLAR|XP_007412640.1 MLAR|XP_007412850.1 MLAR|XP_007413113.1 MLAR|XP_007413115.1 MLAR|XP_007413351.1 MLAR|XP_007413602.1 MLAR|XP_007413611.1 MLAR|XP_007413736.1 MLAR|XP_007413768.1 MLAR|XP_007413769.1 MLAR|XP_007413793.1 MLAR|XP_007414180.1 MLAR|XP_007414280.1 MLAR|XP_007414478.1 MLAR|XP_007414507.1 MLAR|XP_007414528.1 MLAR|XP_007414529.1 MLAR|XP_007414695.1 MLAR|XP_007414815.1 MLAR|XP_007415018.1 MLAR|XP_007415111.1 MLAR|XP_007415143.1 MLAR|XP_007415173.1 MLAR|XP_007415209.1 MLAR|XP_007415488.1 MLAR|XP_007415927.1 MLAR|XP_007416736.1 MLAR|XP_007416844.1 MLAR|XP_007416914.1 MLAR|XP_007416915.1 MLAR|XP_007417377.1 MLAR|XP_007417526.1 MLAR|XP_007417988.1 MLAR|XP_007418012.1 MLAR|XP_007418066.1 MLAR|XP_007418246.1 MLAR|XP_007418458.1 MLAR|XP_007418707.1 MLAR|XP_007419134.1 MLAR|XP_007419135.1 MLAR|XP_007419145.1 MLAR|XP_007419192.1 MLAR|XP_007419390.1 MLAR|XP_007419407.1 MLAR|XP_007419408.1 MLAR|XP_007419418.1 MLAR|XP_007419666.1

>Orthogroup20: BGRA|VCU38747.1 BGRA|VCU38748.1 BGRA|VCU38749.1 BGRA|VCU39388.1 BGRA|VCU39389.1 BGRA|VCU39390.1 BGRA|VCU39391.1 BGRA|VCU39395.1 BGRA|VCU39396.1 BGRA|VCU39399.1 BGRA|VCU39402.1 BGRA|VCU39403.1 BGRA|VCU39544.1 BGRA|VCU39555.1 BGRA|VCU39619.1 BGRA|VCU39620.1 BGRA|VCU40003.1 BGRA|VCU40006.1 BGRA|VCU40062.1 BGRA|VCU40063.1 BGRA|VCU40066.1 BGRA|VCU40068.1 BGRA|VCU40069.1 BGRA|VCU40070.1 BGRA|VCU40090.1 BGRA|VCU40092.1 BGRA|VCU40095.1 BGRA|VCU40097.1 BGRA|VCU40119.1 BGRA|VCU40393.1 BGRA|VCU40395.1 BGRA|VCU40398.1 BGRA|VCU40427.1 BGRA|VCU40430.1 BGRA|VCU40592.1 BGRA|VCU40802.1 BGRA|VCU40803.1 BGRA|VCU40804.1 BGRA|VCU40805.1 BGRA|VCU40806.1 BGRA|VCU40810.1 BGRA|VCU40811.1 BGRA|VCU41259.1 BGRA|VCU41262.1 BGRA|VDB83494.1 BGRA|VDB83497.1 BGRA|VDB83503.1 BGRA|VDB83504.1 BGRA|VDB83505.1 BGRA|VDB83506.1 BGRA|VDB83507.1 BGRA|VDB83517.1 BGRA|VDB83518.1 BGRA|VDB83527.1 BGRA|VDB83528.1 BGRA|VDB83529.1 BGRA|VDB83530.1 BGRA|VDB83534.1 BGRA|VDB83535.1 BGRA|VDB83536.1 BGRA|VDB83537.1 BGRA|VDB83560.1 BGRA|VDB83592.1 BGRA|VDB83593.1 BGRA|VDB83594.1 BGRA|VDB83604.1 BGRA|VDB83605.1 BGRA|VDB83621.1 BGRA|VDB83788.1 BGRA|VDB83794.1 BGRA|VDB83956.1 BGRA|VDB84081.1 BGRA|VDB84163.1 BGRA|VDB88296.1 BGRA|VDB88318.1 BGRA|VDB89110.1 BGRA|VDB89112.1 BGRA|VDB90578.1 BGRA|VDB90744.1 BGRA|VDB90782.1 BGRA|VDB90783.1 BGRA|VDB90789.1 BGRA|VDB90791.1 BGRA|VDB90792.1 BGRA|VDB90793.1 BGRA|VDB92611.1 BGRA|VDB92615.1 BGRA|VDB92758.1 BGRA|VDB92760.1 BGRA|VDB93855.1 BGRA|VDB93856.1 BGRA|VDB94587.1 BGRA|VDB94589.1 BGRA|VDB95243.1 BGRA|VDB95245.1 BGRA|VDB95265.1 BGRA|VDB95267.1 BGRA|VDB95271.1 BGRA|VDB95273.1 BGRA|VDB95275.1 BGRA|VDB95295.1 BGRA|VDB95297.1 BGRA|VDB95299.1 BGRA|VDB95303.1

>Orthogroup21: ANID|CBF71642.1 ANID|CBF79136.1 ANID|CBF82888.1 ANID|CBF82892.1 ANID|CBF83438.1 ANID|CBF84557.1 ANID|CBF85560.1 ANID|CBF86739.1 ANID|CBF87127.1 ANID|CBF87706.1 BCIN|XP_001548762.1 BCIN|XP_001548877.1 BCIN|XP_024547792.1 BCIN|XP_024549508.1 BCIN|XP_024549523.1 BCIN|XP_024551212.1 BCIN|XP_024552550.1 CFRU|XP_031877448.1 CFRU|XP_031878662.1 CFRU|XP_031878794.1 CFRU|XP_031883213.1 CFRU|XP_031883720.1 CFRU|XP_031883762.1 CFRU|XP_031884195.1 CFRU|XP_031884270.1 CFRU|XP_031887174.1 CFRU|XP_031888821.1 CFRU|XP_031889490.1 CFRU|XP_031889921.1 CFRU|XP_031889930.1 CFRU|XP_031892844.1 CGLO|KAF3797473.1 CGLO|KAF3797692.1 CGLO|KAF3798283.1 CGLO|KAF3798321.1 CGLO|KAF3799958.1 CGLO|KAF3802590.1 CGLO|KAF3804184.1 CGLO|KAF3807217.1 CGLO|KAF3808276.1 CGLO|KAF3808468.1 CGLO|KAF3809051.1 CGLO|KAF3809399.1 CGLO|KAF3810617.1 CGLO|KAF3810880.1 CGLO|KAF3811024.1 CHIG|XP_018152991.1 CHIG|XP_018153886.1 CHIG|XP_018154057.1 CHIG|XP_018155477.1 CHIG|XP_018155493.1 CHIG|XP_018156515.1 CHIG|XP_018157023.1 CHIG|XP_018159069.1 CHIG|XP_018163138.1 CVIN|KAF4908284.1 CVIN|KAF4908290.1 CVIN|KAF4915223.1 CVIN|KAF4916413.1 CVIN|KAF4918157.1 CVIN|KAF4918392.1 CVIN|KAF4920338.1 CVIN|KAF4926907.1 CVIN|KAF4926933.1 CVIN|KAF4928120.1 CVIN|KAF4928325.1 CVYL|A01076 CVYL|A02265 CVYL|A03008 CVYL|A03858 CVYL|A09047 CVYL|A09757 CVYL|A09788 CVYL|A09899 CVYL|A10172 CVYL|A10700 CVYL|A11671 CVYL|A13699 FGRM|XP_011315700.1 FGRM|XP_011317905.1 FGRM|XP_011319613.1 FGRM|XP_011319616.1 FGRM|XP_011320367.1 FGRM|XP_011323172.1 FGRM|XP_011327452.1 MGRA|XP_003851423.1 MGRA|XP_003854174.1 MGRA|XP_003854258.1 MORY|QBZ54339.1 MORY|QBZ57142.1 MORY|QBZ57630.1 MORY|QBZ62897.1 MORY|QBZ64212.1 MORY|QBZ64328.1 MORY|QBZ66502.1 MORY|QBZ66512.1 NCRA|XP_958492.1 NCRA|XP_961030.1 SSCL|APA06370.1 SSCL|APA09132.1 SSCL|APA09478.1 SSCL|APA09580.1 SSCL|APA15117.1

>Orthogroup22: ANID|CBF73484.1 ANID|CBF80392.1 ANID|CBF82705.1 ANID|CBF84100.1 ANID|CBF84497.1 ANID|CBF84623.1 ANID|CBF89303.1 BCIN|XP_001557793.1 BCIN|XP_024547132.1 BCIN|XP_024547627.1 BCIN|XP_024548242.1 BCIN|XP_024548755.1 BCIN|XP_024549048.1 BCIN|XP_024551039.1 CFRU|XP_031876825.1 CFRU|XP_031877314.1 CFRU|XP_031879338.1 CFRU|XP_031883587.1 CFRU|XP_031884217.1 CFRU|XP_031884232.1 CFRU|XP_031887063.1 CFRU|XP_031887969.1 CFRU|XP_031889896.1 CFRU|XP_031891001.1 CFRU|XP_031891597.1 CFRU|XP_031892081.1 CFRU|XP_031892218.1 CFRU|XP_031893480.1 CGLO|KAF3797404.1 CGLO|KAF3797611.1 CGLO|KAF3799925.1 CGLO|KAF3800211.1 CGLO|KAF3801404.1 CGLO|KAF3805613.1 CGLO|KAF3805809.1 CGLO|KAF3808655.1 CGLO|KAF3809606.1 CGLO|KAF3810483.1 CGLO|KAF3811044.1 CGLO|KAF3811061.1 CGLO|KAF3811428.1 CGLO|KAF3811702.1 CHIG|XP_018150800.1 CHIG|XP_018151885.1 CHIG|XP_018153119.1 CHIG|XP_018153761.1 CHIG|XP_018154035.1 CHIG|XP_018154113.1 CHIG|XP_018155126.1 CHIG|XP_018156511.1 CHIG|XP_018157364.1 CHIG|XP_018158415.1 CHIG|XP_018158450.1 CHIG|XP_018159128.1 CHIG|XP_018160669.1 CVIN|KAF4903415.1 CVIN|KAF4903416.1 CVIN|KAF4906965.1 CVIN|KAF4914256.1 CVIN|KAF4914934.1 CVIN|KAF4915258.1 CVIN|KAF4915945.1 CVIN|KAF4917881.1 CVIN|KAF4919212.1 CVIN|KAF4920409.1 CVIN|KAF4922360.1 CVIN|KAF4924628.1 CVIN|KAF4926124.1 CVIN|KAF4929399.1 CVYL|A03382 CVYL|A04626 CVYL|A06075 CVYL|A07583 CVYL|A08435 CVYL|A10273 CVYL|A11246 CVYL|A14086 FGRM|XP_011315815.1 FGRM|XP_011318619.1 FGRM|XP_011320578.1 FGRM|XP_011320738.1 FGRM|XP_011321005.1 FGRM|XP_011326844.1 FGRM|XP_011327778.1 MGRA|XP_003847590.1 MGRA|XP_003847825.1 MGRA|XP_003849213.1 MGRA|XP_003851864.1 MGRA|XP_003852827.1 MGRA|XP_003856169.1 MORY|QBZ56387.1 MORY|QBZ57136.1 MORY|QBZ57833.1 MORY|QBZ59926.1 MORY|QBZ60671.1 MORY|QBZ65203.1 NCRA|XP_956353.3 NCRA|XP_957075.1 SSCL|APA12719.1 SSCL|APA14032.1

>Orthogroup23: ANID|CBF69531.1 ANID|CBF71459.1 ANID|CBF73549.1 ANID|CBF75785.1 ANID|CBF77085.1 ANID|CBF78322.1 ANID|CBF79119.1 ANID|CBF80279.1 ANID|CBF82038.1 ANID|CBF85575.1 ANID|CBF86063.1 ANID|CBF87384.1 BCIN|XP_001548885.2 BCIN|XP_024550102.1 BCIN|XP_024551883.1 BCIN|XP_024551952.1 BCIN|XP_024552384.1 BCIN|XP_024552493.1 BCIN|XP_024552713.1 BCIN|XP_024553095.1 BCIN|XP_024553194.1 BCIN|XP_024553702.1 BCIN|XP_024553710.1 BCIN|XP_024553812.1 CFRU|XP_031876017.1 CFRU|XP_031876068.1 CFRU|XP_031876601.1 CFRU|XP_031877285.1 CFRU|XP_031877295.1 CFRU|XP_031877597.1 CFRU|XP_031878261.1 CFRU|XP_031881064.1 CFRU|XP_031882543.1 CFRU|XP_031883119.1 CFRU|XP_031883791.1 CFRU|XP_031885947.1 CFRU|XP_031891361.1 CFRU|XP_031891388.1 CGLO|KAF3797920.1 CGLO|KAF3798074.1 CGLO|KAF3800897.1 CGLO|KAF3801981.1 CGLO|KAF3804593.1 CGLO|KAF3806789.1 CGLO|KAF3806802.1 CGLO|KAF3806813.1 CGLO|KAF3808202.1 CGLO|KAF3808507.1 CGLO|KAF3809906.1 CHIG|XP_018153112.1 CHIG|XP_018153303.1 CHIG|XP_018157123.1 CHIG|XP_018159450.1 CHIG|XP_018160034.1 CHIG|XP_018161599.1 CHIG|XP_018161674.1 CHIG|XP_018163211.1 CVIN|KAF4907255.1 CVIN|KAF4907839.1 CVIN|KAF4908874.1 CVIN|KAF4914236.1 CVIN|KAF4924283.1 CVIN|KAF4928004.1 CVIN|KAF4930382.1 CVIN|KAF4930652.1 CVYL|A00551 CVYL|A02451 CVYL|A02459 CVYL|A05285 CVYL|A05355 CVYL|A05566 CVYL|A08284 CVYL|A12434 FGRM|XP_011317574.1 FGRM|XP_011318253.1 FGRM|XP_011319911.1 FGRM|XP_011320626.1 FGRM|XP_011322174.1 FGRM|XP_011325322.1 MGRA|XP_003850939.1 MGRA|XP_003854793.1 MGRA|XP_003855638.1 MGRA|XP_003855815.1 MGRA|XP_003856124.1 MORY|QBZ61145.1 MORY|QBZ61185.1 MORY|QBZ64223.1 MORY|QBZ64948.1 MORY|QBZ66582.1 NCRA|XP_001728000.2 NCRA|XP_963577.3 NCRA|XP_964920.2 SSCL|APA06217.1 SSCL|APA10778.1 SSCL|APA15095.1 SSCL|APA15993.1 SSCL|APA16308.1

>Orthogroup24: ANID|CBF69707.1 ANID|CBF70273.1 ANID|CBF75096.1 ANID|CBF76609.1 ANID|CBF79013.1 ANID|CBF87513.1 BCIN|XP_001548471.2 BCIN|XP_001550556.2 BCIN|XP_001552081.1 BCIN|XP_001554305.2 BCIN|XP_001554811.1 BCIN|XP_001555473.1 BCIN|XP_001560806.2 BCIN|XP_024546823.1 BCIN|XP_024546945.1 BCIN|XP_024547569.1 BCIN|XP_024548702.1 BCIN|XP_024548768.1 BCIN|XP_024549192.1 BCIN|XP_024549318.1 BCIN|XP_024549886.1 BCIN|XP_024550498.1 BCIN|XP_024550763.1 BCIN|XP_024552364.1 BCIN|XP_024553087.1 BCIN|XP_024553170.1 BGRA|VDB89081.1 CFRU|XP_031879475.1 CFRU|XP_031880405.1 CFRU|XP_031883953.1 CFRU|XP_031885338.1 CFRU|XP_031888385.1 CFRU|XP_031891983.1 CGLO|KAF3799475.1 CGLO|KAF3801268.1 CGLO|KAF3802432.1 CGLO|KAF3803634.1 CGLO|KAF3803940.1 CGLO|KAF3805078.1 CGLO|KAF3810626.1 CHIG|XP_018151610.1 CHIG|XP_018154460.1 CHIG|XP_018154733.1 CHIG|XP_018156613.1 CHIG|XP_018156924.1 CHIG|XP_018163435.1 CHIG|XP_018163825.1 CHIG|XP_018164863.1 CVIN|KAF4910016.1 CVIN|KAF4913947.1 CVIN|KAF4918230.1 CVIN|KAF4921557.1 CVIN|KAF4927435.1 CVIN|KAF4928146.1 CVIN|KAF4931788.1 CVYL|A00319 CVYL|A06481 CVYL|A07822 CVYL|A08863 CVYL|A13337 CVYL|A13574 CVYL|A14569 FGRM|XP_011317065.1 FGRM|XP_011320470.1 MGRA|XP_003848221.1 MGRA|XP_003848297.1 MGRA|XP_003851807.1 MGRA|XP_003852888.1 MGRA|XP_003855019.1 MORY|QBZ53546.1 MORY|QBZ54375.1 MORY|QBZ54690.1 MORY|QBZ55838.1 MORY|QBZ58601.1 MORY|QBZ60209.1 MORY|QBZ61698.1 MORY|QBZ64957.1 MORY|QBZ65954.1 NCRA|XP_958030.2 NCRA|XP_964653.3 SSCL|APA05562.1 SSCL|APA06311.1 SSCL|APA06371.1 SSCL|APA06372.1 SSCL|APA06373.1 SSCL|APA08185.1 SSCL|APA09715.1 SSCL|APA09811.1 SSCL|APA09969.1 SSCL|APA10335.1 SSCL|APA11216.1 SSCL|APA13818.1 SSCL|APA14179.1 SSCL|APA14392.1 SSCL|APA15520.1

>Orthogroup25: BGRA|VCU38763.1 BGRA|VCU39275.1 BGRA|VCU39716.1 BGRA|VCU40292.1 BGRA|VCU40464.1 BGRA|VCU40477.1 BGRA|VCU40603.1 BGRA|VCU40612.1 BGRA|VCU40671.1 BGRA|VCU40672.1 BGRA|VCU40732.1 BGRA|VCU40735.1 BGRA|VCU40742.1 BGRA|VCU40743.1 BGRA|VCU40744.1 BGRA|VCU40745.1 BGRA|VCU40746.1 BGRA|VCU40747.1 BGRA|VCU40963.1 BGRA|VCU40964.1 BGRA|VCU40968.1 BGRA|VCU40971.1 BGRA|VCU41012.1 BGRA|VCU41037.1 BGRA|VCU41056.1 BGRA|VCU41058.1 BGRA|VCU41060.1 BGRA|VDB84102.1 BGRA|VDB85756.1 BGRA|VDB85839.1 BGRA|VDB85841.1 BGRA|VDB85858.1 BGRA|VDB85860.1 BGRA|VDB85862.1 BGRA|VDB85878.1 BGRA|VDB85880.1 BGRA|VDB85882.1 BGRA|VDB85890.1 BGRA|VDB85894.1 BGRA|VDB85916.1 BGRA|VDB85918.1 BGRA|VDB85919.1 BGRA|VDB85922.1 BGRA|VDB85971.1 BGRA|VDB85972.1 BGRA|VDB85978.1 BGRA|VDB85987.1 BGRA|VDB85996.1 BGRA|VDB86004.1 BGRA|VDB86005.1 BGRA|VDB86291.1 BGRA|VDB86397.1 BGRA|VDB87650.1 BGRA|VDB87818.1 BGRA|VDB88269.1 BGRA|VDB88280.1 BGRA|VDB88797.1 BGRA|VDB88839.1 BGRA|VDB88888.1 BGRA|VDB88890.1 BGRA|VDB89321.1 BGRA|VDB89327.1 BGRA|VDB89329.1 BGRA|VDB89333.1 BGRA|VDB89335.1 BGRA|VDB89349.1 BGRA|VDB89368.1 BGRA|VDB89398.1 BGRA|VDB90591.1 BGRA|VDB91012.1 BGRA|VDB91014.1 BGRA|VDB91037.1 BGRA|VDB91049.1 BGRA|VDB91051.1 BGRA|VDB91052.1 BGRA|VDB91166.1 BGRA|VDB92759.1 BGRA|VDB92995.1 BGRA|VDB93112.1 BGRA|VDB93117.1 BGRA|VDB93189.1 BGRA|VDB93305.1 BGRA|VDB93409.1 BGRA|VDB93411.1 BGRA|VDB93625.1 BGRA|VDB93639.1 BGRA|VDB93645.1 BGRA|VDB93654.1 BGRA|VDB94633.1 BGRA|VDB94636.1 BGRA|VDB94640.1 BGRA|VDB94827.1 BGRA|VDB96318.1 BGRA|VDB96372.1 BGRA|VDB96394.1

>Orthogroup26: BGRA|VCU38746.1 BGRA|VCU38800.1 BGRA|VCU38802.1 BGRA|VCU39002.1 BGRA|VCU39364.1 BGRA|VCU39369.1 BGRA|VCU39371.1 BGRA|VCU39377.1 BGRA|VCU39381.1 BGRA|VCU39382.1 BGRA|VCU39387.1 BGRA|VCU40160.1 BGRA|VCU40192.1 BGRA|VCU40236.1 BGRA|VCU40263.1 BGRA|VCU40264.1 BGRA|VCU40275.1 BGRA|VCU40276.1 BGRA|VCU40333.1 BGRA|VCU40335.1 BGRA|VCU40336.1 BGRA|VCU40337.1 BGRA|VCU40339.1 BGRA|VCU40340.1 BGRA|VCU40343.1 BGRA|VCU40344.1 BGRA|VCU40346.1 BGRA|VCU40350.1 BGRA|VCU40420.1 BGRA|VCU40448.1 BGRA|VCU40452.1 BGRA|VCU40784.1 BGRA|VCU40787.1 BGRA|VCU40788.1 BGRA|VCU40959.1 BGRA|VCU41146.1 BGRA|VCU41397.1 BGRA|VCU41400.1 BGRA|VCU41406.1 BGRA|VCU41407.1 BGRA|VCU41408.1 BGRA|VCU41409.1 BGRA|VDB83513.1 BGRA|VDB83520.1 BGRA|VDB83555.1 BGRA|VDB84215.1 BGRA|VDB84217.1 BGRA|VDB85773.1 BGRA|VDB85774.1 BGRA|VDB88107.1 BGRA|VDB88112.1 BGRA|VDB88127.1 BGRA|VDB88258.1 BGRA|VDB88259.1 BGRA|VDB88262.1 BGRA|VDB88265.1 BGRA|VDB88266.1 BGRA|VDB88267.1 BGRA|VDB88272.1 BGRA|VDB88273.1 BGRA|VDB88274.1 BGRA|VDB88278.1 BGRA|VDB88294.1 BGRA|VDB88308.1 BGRA|VDB88337.1 BGRA|VDB88339.1 BGRA|VDB88341.1 BGRA|VDB88369.1 BGRA|VDB88371.1 BGRA|VDB88372.1 BGRA|VDB88374.1 BGRA|VDB88377.1 BGRA|VDB88384.1 BGRA|VDB88388.1 BGRA|VDB89185.1 BGRA|VDB89341.1 BGRA|VDB89343.1 BGRA|VDB89345.1 BGRA|VDB89535.1 BGRA|VDB89536.1 BGRA|VDB89537.1 BGRA|VDB90918.1 BGRA|VDB91241.1 BGRA|VDB91257.1 BGRA|VDB91397.1 BGRA|VDB92405.1 BGRA|VDB92407.1 BGRA|VDB93310.1 BGRA|VDB93311.1 BGRA|VDB93998.1 BGRA|VDB94747.1 BGRA|VDB94748.1 BGRA|VDB95102.1

>Orthogroup27: BGRA|VCU39173.1 BGRA|VCU39174.1 BGRA|VCU39352.1 BGRA|VCU39452.1 BGRA|VCU39459.1 BGRA|VCU39518.1 BGRA|VCU39529.1 BGRA|VCU39532.1 BGRA|VCU39638.1 BGRA|VCU39654.1 BGRA|VCU39746.1 BGRA|VCU39788.1 BGRA|VCU39807.1 BGRA|VCU39878.1 BGRA|VCU39884.1 BGRA|VCU40202.1 BGRA|VCU40210.1 BGRA|VCU40241.1 BGRA|VCU40300.1 BGRA|VCU40576.1 BGRA|VCU40588.1 BGRA|VCU40623.1 BGRA|VCU40652.1 BGRA|VCU40849.1 BGRA|VCU40932.1 BGRA|VCU40956.1 BGRA|VCU40980.1 BGRA|VCU41062.1 BGRA|VCU41144.1 BGRA|VCU41176.1 BGRA|VCU41387.1 BGRA|VDB83543.1 BGRA|VDB83613.1 BGRA|VDB83849.1 BGRA|VDB83850.1 BGRA|VDB84364.1 BGRA|VDB85664.1 BGRA|VDB85688.1 BGRA|VDB85744.1 BGRA|VDB85957.1 BGRA|VDB86129.1 BGRA|VDB86376.1 BGRA|VDB87854.1 BGRA|VDB87934.1 BGRA|VDB87977.1 BGRA|VDB88047.1 BGRA|VDB88147.1 BGRA|VDB88148.1 BGRA|VDB88165.1 BGRA|VDB88224.1 BGRA|VDB88364.1 BGRA|VDB89020.1 BGRA|VDB89058.1 BGRA|VDB89059.1 BGRA|VDB89436.1 BGRA|VDB89448.1 BGRA|VDB89461.1 BGRA|VDB89481.1 BGRA|VDB89681.1 BGRA|VDB89869.1 BGRA|VDB89872.1 BGRA|VDB89879.1 BGRA|VDB89950.1 BGRA|VDB90371.1 BGRA|VDB90391.1 BGRA|VDB90624.1 BGRA|VDB90632.1 BGRA|VDB90691.1 BGRA|VDB90725.1 BGRA|VDB90886.1 BGRA|VDB91389.1 BGRA|VDB92695.1 BGRA|VDB92730.1 BGRA|VDB92987.1 BGRA|VDB93182.1 BGRA|VDB93196.1 BGRA|VDB93624.1 BGRA|VDB93961.1 BGRA|VDB93988.1 BGRA|VDB93993.1 BGRA|VDB94012.1 BGRA|VDB94458.1 BGRA|VDB94472.1 BGRA|VDB94480.1 BGRA|VDB94608.1 BGRA|VDB94609.1 BGRA|VDB94693.1 BGRA|VDB94922.1 BGRA|VDB94937.1 BGRA|VDB96371.1 BGRA|VDB96432.1 FGRM|XP_011319866.1

>Orthogroup28: ANID|CBF69359.1 ANID|CBF73509.1 ANID|CBF82122.1 ANID|CBF87520.1 ANID|CBF87727.1 ANID|CBF89919.1 BCIN|XP_024546944.1 BCIN|XP_024547466.1 BCIN|XP_024553131.1 BCIN|XP_024553708.1 CFRU|XP_031875912.1 CFRU|XP_031878418.1 CFRU|XP_031879994.1 CFRU|XP_031881135.1 CFRU|XP_031882765.1 CFRU|XP_031882937.1 CFRU|XP_031882963.1 CFRU|XP_031887795.1 CFRU|XP_031890932.1 CFRU|XP_031891596.1 CFRU|XP_031891945.1 CGLO|KAF3797403.1 CGLO|KAF3800456.1 CGLO|KAF3802102.1 CGLO|KAF3803861.1 CGLO|KAF3805183.1 CGLO|KAF3806644.1 CGLO|KAF3806653.1 CGLO|KAF3807818.1 CGLO|KAF3808210.1 CGLO|KAF3809691.1 CGLO|KAF3810422.1 CGLO|KAF3810761.1 CGLO|KAF3811741.1 CHIG|XP_018151058.1 CHIG|XP_018151777.1 CHIG|XP_018157362.1 CHIG|XP_018158427.1 CHIG|XP_018161369.1 CHIG|XP_018162558.1 CHIG|XP_018162984.1 CHIG|XP_018163194.1 CHIG|XP_018164216.1 CHIG|XP_018164222.1 CVIN|KAF4902813.1 CVIN|KAF4908252.1 CVIN|KAF4917959.1 CVIN|KAF4918908.1 CVIN|KAF4921532.1 CVIN|KAF4921942.1 CVIN|KAF4922994.1 CVIN|KAF4924315.1 CVIN|KAF4925447.1 CVIN|KAF4929400.1 CVIN|KAF4932140.1 CVYL|A00558 CVYL|A00785 CVYL|A01841 CVYL|A03289 CVYL|A06610 CVYL|A07193 CVYL|A08939 CVYL|A10275 CVYL|A11383 CVYL|A12358 CVYL|A12368 CVYL|A12756 FGRM|XP_011317852.1 FGRM|XP_011321886.1 FGRM|XP_011322384.1 FGRM|XP_011323098.1 FGRM|XP_011325769.1 MGRA|XP_003847727.1 MGRA|XP_003847773.1 MGRA|XP_003855468.1 MLAR|XP_007417973.1 MORY|QBZ53910.1 MORY|QBZ55923.1 MORY|QBZ57961.1 MORY|QBZ58288.1 MORY|QBZ58607.1 MORY|QBZ58916.1 MORY|QBZ60162.1 MORY|QBZ65305.1 MORY|QBZ66435.1 NCRA|XP_001728070.2 NCRA|XP_001728263.1 NCRA|XP_011393252.1 SSCL|APA15101.1

>Orthogroup29: ANID|CBF69887.1 ANID|CBF73453.1 ANID|CBF76036.1 ANID|CBF76038.1 ANID|CBF77087.1 ANID|CBF80539.1 ANID|CBF84349.1 ANID|CBF87069.1 ANID|CBF87235.1 ANID|CBF87236.1 ANID|CBF87869.1 ANID|CBF90372.1 BCIN|XP_001550898.2 BCIN|XP_001552859.2 BCIN|XP_024546178.1 BCIN|XP_024546952.1 BCIN|XP_024551708.1 CFRU|XP_031876560.1 CFRU|XP_031876603.1 CFRU|XP_031881358.1 CFRU|XP_031883329.1 CFRU|XP_031883834.1 CFRU|XP_031884131.1 CFRU|XP_031885633.1 CFRU|XP_031887830.1 CFRU|XP_031893218.1 CGLO|KAF3799977.1 CGLO|KAF3800591.1 CGLO|KAF3800880.1 CGLO|KAF3802065.1 CGLO|KAF3805101.1 CGLO|KAF3809504.1 CGLO|KAF3810065.1 CHIG|XP_018150810.1 CHIG|XP_018150828.1 CHIG|XP_018151538.1 CHIG|XP_018151732.1 CHIG|XP_018152088.1 CHIG|XP_018152658.1 CHIG|XP_018154907.1 CHIG|XP_018156534.1 CHIG|XP_018156835.1 CHIG|XP_018162263.1 CHIG|XP_018163337.1 CVIN|KAF4907268.1 CVIN|KAF4911862.1 CVIN|KAF4911872.1 CVIN|KAF4912753.1 CVIN|KAF4917971.1 CVIN|KAF4927053.1 CVYL|A02661 CVYL|A06456 CVYL|A10072 CVYL|A10196 CVYL|A11118 CVYL|A12445 CVYL|A12446 CVYL|A13125 FGRM|XP_011315630.1 FGRM|XP_011315631.1 FGRM|XP_011318224.1 FGRM|XP_011318306.1 FGRM|XP_011319542.1 FGRM|XP_011319713.1 FGRM|XP_011319727.1 FGRM|XP_011320598.1 FGRM|XP_011321944.1 FGRM|XP_011325380.1 FGRM|XP_011325382.1 FGRM|XP_011325925.1 MGRA|XP_003847857.1 MGRA|XP_003850202.1 MGRA|XP_003851777.1 MGRA|XP_003851968.1 MGRA|XP_003855676.1 MLAR|XP_007414336.1 MORY|QBZ53722.1 MORY|QBZ55850.1 MORY|QBZ59901.1 MORY|QBZ63590.1 MORY|QBZ64288.1 MORY|QBZ66643.1 NCRA|XP_963411.3 SSCL|APA06242.1 SSCL|APA06243.1 SSCL|APA07050.1 SSCL|APA12397.1

>Orthogroup30: MLAR|XP_007403646.1 MLAR|XP_007403817.1 MLAR|XP_007404277.1 MLAR|XP_007404470.1 MLAR|XP_007404539.1 MLAR|XP_007405196.1 MLAR|XP_007405324.1 MLAR|XP_007405325.1 MLAR|XP_007405469.1 MLAR|XP_007405477.1 MLAR|XP_007405478.1 MLAR|XP_007405482.1 MLAR|XP_007405701.1 MLAR|XP_007405867.1 MLAR|XP_007406350.1 MLAR|XP_007406351.1 MLAR|XP_007406352.1 MLAR|XP_007406611.1 MLAR|XP_007407177.1 MLAR|XP_007407364.1 MLAR|XP_007407677.1 MLAR|XP_007408102.1 MLAR|XP_007408240.1 MLAR|XP_007408370.1 MLAR|XP_007408566.1 MLAR|XP_007408621.1 MLAR|XP_007409435.1 MLAR|XP_007409515.1 MLAR|XP_007410146.1 MLAR|XP_007410894.1 MLAR|XP_007411083.1 MLAR|XP_007411086.1 MLAR|XP_007411182.1 MLAR|XP_007411692.1 MLAR|XP_007411791.1 MLAR|XP_007412057.1 MLAR|XP_007412401.1 MLAR|XP_007412564.1 MLAR|XP_007412565.1 MLAR|XP_007412683.1 MLAR|XP_007412786.1 MLAR|XP_007412873.1 MLAR|XP_007414045.1 MLAR|XP_007414169.1 MLAR|XP_007414183.1 MLAR|XP_007414246.1 MLAR|XP_007414254.1 MLAR|XP_007414279.1 MLAR|XP_007414404.1 MLAR|XP_007414565.1 MLAR|XP_007415086.1 MLAR|XP_007415135.1 MLAR|XP_007415694.1 MLAR|XP_007416106.1 MLAR|XP_007416134.1 MLAR|XP_007416252.1 MLAR|XP_007416626.1 MLAR|XP_007416631.1 MLAR|XP_007416632.1 MLAR|XP_007416921.1 MLAR|XP_007417360.1 MLAR|XP_007417748.1 MLAR|XP_007417749.1 MLAR|XP_007417913.1 MLAR|XP_007417945.1 MLAR|XP_007418109.1 MLAR|XP_007418450.1 MLAR|XP_007418451.1 MLAR|XP_007418452.1 MLAR|XP_007418524.1 MLAR|XP_007418525.1 MLAR|XP_007418537.1 MLAR|XP_007418729.1 MLAR|XP_007418840.1 MLAR|XP_007418973.1 MLAR|XP_007419056.1 MLAR|XP_007419103.1 MLAR|XP_007419206.1 MLAR|XP_007419316.1 MLAR|XP_007419361.1 MLAR|XP_007419396.1 MLAR|XP_007419429.1 MLAR|XP_007419511.1 MLAR|XP_007419728.1 MLAR|XP_007419772.1

>Orthogroup31: ANID|CBF87351.1 BCIN|XP_024549696.1 BCIN|XP_024550391.1 CFRU|XP_031875865.1 CFRU|XP_031876731.1 CFRU|XP_031877101.1 CFRU|XP_031877586.1 CFRU|XP_031878750.1 CFRU|XP_031880129.1 CFRU|XP_031880573.1 CFRU|XP_031883091.1 CFRU|XP_031883710.1 CFRU|XP_031883713.1 CFRU|XP_031884452.1 CFRU|XP_031887143.1 CFRU|XP_031888952.1 CFRU|XP_031892120.1 CFRU|XP_031892875.1 CGLO|KAF3797540.1 CGLO|KAF3798682.1 CGLO|KAF3799767.1 CGLO|KAF3803997.1 CGLO|KAF3804170.1 CGLO|KAF3806752.1 CGLO|KAF3807906.1 CGLO|KAF3808225.1 CGLO|KAF3809205.1 CGLO|KAF3809754.1 CGLO|KAF3810865.1 CGLO|KAF3811842.1 CHIG|XP_018150932.1 CHIG|XP_018151116.1 CHIG|XP_018151692.1 CHIG|XP_018154959.1 CHIG|XP_018154960.1 CHIG|XP_018155423.1 CHIG|XP_018156033.1 CHIG|XP_018157474.1 CHIG|XP_018158002.1 CHIG|XP_018158056.1 CHIG|XP_018158239.1 CHIG|XP_018160031.1 CHIG|XP_018163186.1 CVIN|KAF4897554.1 CVIN|KAF4914268.1 CVIN|KAF4918364.1 CVIN|KAF4918493.1 CVIN|KAF4918981.1 CVIN|KAF4919515.1 CVIN|KAF4923576.1 CVIN|KAF4924870.1 CVIN|KAF4925166.1 CVIN|KAF4925317.1 CVYL|A00566 CVYL|A01394 CVYL|A02281 CVYL|A03841 CVYL|A06673 CVYL|A08804 CVYL|A08998 CVYL|A11328 CVYL|A12844 CVYL|A14062 FGRM|XP_011317604.1 FGRM|XP_011318339.1 FGRM|XP_011322872.1 FGRM|XP_011325714.1 FGRM|XP_011326011.1 FGRM|XP_011326012.1 MGRA|XP_003850440.1 MGRA|XP_003851732.1 MGRA|XP_003854009.1 MGRA|XP_003857003.1 MORY|QBZ56361.1 MORY|QBZ58234.1 MORY|QBZ60032.1 MORY|QBZ60216.1 MORY|QBZ60768.1 MORY|QBZ63457.1 NCRA|XP_959095.1 NCRA|XP_959839.1 NCRA|XP_963842.1 NCRA|XP_963897.2 SSCL|APA05629.1

>Orthogroup32: ANID|CBF74652.1 ANID|CBF74799.1 ANID|CBF81915.1 ANID|CBF84421.1 ANID|CBF85372.1 ANID|CBF89245.1 ANID|CBF90197.1 BCIN|XP_001554714.1 BCIN|XP_001559415.2 BGRA|VDB84374.1 BGRA|VDB88126.1 CFRU|XP_031876331.1 CFRU|XP_031877054.1 CFRU|XP_031877246.1 CFRU|XP_031879498.1 CFRU|XP_031879590.1 CFRU|XP_031880285.1 CFRU|XP_031883261.1 CFRU|XP_031885199.1 CFRU|XP_031889856.1 CFRU|XP_031891541.1 CFRU|XP_031892437.1 CGLO|KAF3797073.1 CGLO|KAF3801745.1 CGLO|KAF3802412.1 CGLO|KAF3802779.1 CGLO|KAF3804698.1 CGLO|KAF3806797.1 CGLO|KAF3807667.1 CGLO|KAF3808949.1 CGLO|KAF3810434.1 CGLO|KAF3811870.1 CHIG|XP_018150762.1 CHIG|XP_018152968.1 CHIG|XP_018154725.1 CHIG|XP_018155183.1 CHIG|XP_018155807.1 CVIN|KAF4899039.1 CVIN|KAF4911077.1 CVIN|KAF4912218.1 CVIN|KAF4918209.1 CVIN|KAF4920555.1 CVIN|KAF4920761.1 CVIN|KAF4922934.1 CVIN|KAF4923384.1 CVIN|KAF4924642.1 CVIN|KAF4927821.1 CVYL|A01146 CVYL|A05425 CVYL|A06190 CVYL|A06786 CVYL|A07985 CVYL|A07992 CVYL|A10636 CVYL|A12957 CVYL|A13556 CVYL|A13778 FGRM|XP_011315742.1 FGRM|XP_011316725.1 FGRM|XP_011317595.1 FGRM|XP_011318042.1 FGRM|XP_011318179.1 FGRM|XP_011318205.1 FGRM|XP_011318304.1 FGRM|XP_011321434.1 FGRM|XP_011324427.1 FGRM|XP_011325327.1 FGRM|XP_011325731.1 MGRA|XP_003849270.1 MGRA|XP_003849597.1 MGRA|XP_003854072.1 MLAR|XP_007404292.1 MLAR|XP_007404797.1 MLAR|XP_007406345.1 MLAR|XP_007407030.1 MORY|QBZ59331.1 MORY|QBZ61230.1 MORY|QBZ64859.1 NCRA|XP_956862.1 NCRA|XP_957264.2 NCRA|XP_960135.1 SSCL|APA07671.1 SSCL|APA15456.1

>Orthogroup33: ANID|CBF80512.1 ANID|CBF83502.1 ANID|CBF83933.1 ANID|CBF89574.1 ANID|CBF89592.1 BCIN|XP_001550175.1 BCIN|XP_001554597.1 BCIN|XP_001556145.1 BCIN|XP_024547318.1 BCIN|XP_024550636.1 BCIN|XP_024552613.1 BCIN|XP_024553530.1 BGRA|VCU41163.1 CFRU|XP_031877509.1 CFRU|XP_031879259.1 CFRU|XP_031880404.1 CFRU|XP_031884116.1 CFRU|XP_031885451.1 CFRU|XP_031887641.1 CFRU|XP_031889476.1 CFRU|XP_031891498.1 CGLO|KAF3797534.1 CGLO|KAF3803301.1 CGLO|KAF3806531.1 CGLO|KAF3807028.1 CGLO|KAF3807251.1 CGLO|KAF3808848.1 CGLO|KAF3811010.1 CHIG|XP_018153027.1 CHIG|XP_018155749.1 CHIG|XP_018156925.1 CHIG|XP_018157569.1 CHIG|XP_018159713.1 CHIG|XP_018160397.1 CHIG|XP_018161264.1 CHIG|XP_018163717.1 CVIN|KAF4909975.1 CVIN|KAF4920285.1 CVIN|KAF4921358.1 CVIN|KAF4921650.1 CVIN|KAF4924491.1 CVIN|KAF4926897.1 CVIN|KAF4928147.1 CVIN|KAF4928634.1 CVYL|A01232 CVYL|A01983 CVYL|A03735 CVYL|A04258 CVYL|A06323 CVYL|A09339 CVYL|A09777 CVYL|A10732 FGRM|XP_011319357.1 FGRM|XP_011320219.1 FGRM|XP_011320999.1 FGRM|XP_011321979.1 FGRM|XP_011326703.1 FGRM|XP_011328059.1 MGRA|XP_003856039.1 MGRA|XP_003857459.1 MLAR|XP_007404609.1 MLAR|XP_007410834.1 MLAR|XP_007410919.1 MORY|QBZ57188.1 MORY|QBZ58167.1 MORY|QBZ62236.1 MORY|QBZ63706.1 MORY|QBZ64721.1 MORY|QBZ65953.1 NCRA|XP_957931.2 NCRA|XP_959431.1 NCRA|XP_959670.1 NCRA|XP_961116.1 NCRA|XP_961253.2 NCRA|XP_962581.3 NCRA|XP_964654.3 SSCL|APA08737.1 SSCL|APA08869.1 SSCL|APA11333.1 SSCL|APA11453.1 SSCL|APA11655.1 SSCL|APA13767.1 SSCL|APA14299.1

>Orthogroup34: ANID|CBF70181.1 ANID|CBF73895.1 ANID|CBF76010.1 ANID|CBF76012.1 ANID|CBF82527.1 ANID|CBF83992.1 ANID|CBF86648.1 ANID|CBF86676.1 BCIN|XP_001545295.2 BCIN|XP_001545827.2 BCIN|XP_001548264.1 BCIN|XP_001560174.1 BCIN|XP_001561189.2 BCIN|XP_024552470.1 CFRU|XP_031877937.1 CFRU|XP_031878411.1 CFRU|XP_031880074.1 CFRU|XP_031883981.1 CFRU|XP_031884526.1 CFRU|XP_031884678.1 CFRU|XP_031884916.1 CFRU|XP_031885109.1 CFRU|XP_031887981.1 CFRU|XP_031889431.1 CFRU|XP_031890262.1 CFRU|XP_031892741.1 CGLO|KAF3797290.1 CGLO|KAF3797686.1 CGLO|KAF3799666.1 CGLO|KAF3802090.1 CGLO|KAF3802810.1 CGLO|KAF3805007.1 CGLO|KAF3807752.1 CGLO|KAF3810744.1 CGLO|KAF3810900.1 CGLO|KAF3812180.1 CHIG|XP_018151067.1 CHIG|XP_018151069.1 CHIG|XP_018151232.1 CHIG|XP_018152636.1 CHIG|XP_018153403.1 CHIG|XP_018154107.1 CHIG|XP_018154933.1 CHIG|XP_018156271.1 CHIG|XP_018158419.1 CHIG|XP_018159056.1 CHIG|XP_018163512.1 CVIN|KAF4901000.1 CVIN|KAF4908249.1 CVIN|KAF4916342.1 CVIN|KAF4920535.1 CVIN|KAF4920955.1 CVIN|KAF4920956.1 CVIN|KAF4922755.1 CVIN|KAF4927139.1 CVIN|KAF4928277.1 CVIN|KAF4932015.1 CVYL|A00207 CVYL|A00846 CVYL|A01085 CVYL|A02245 CVYL|A06542 CVYL|A07207 FGRM|XP_011315835.1 FGRM|XP_011317959.1 FGRM|XP_011321037.1 FGRM|XP_011321929.1 FGRM|XP_011323137.1 FGRM|XP_011323138.1 FGRM|XP_011323315.1 FGRM|XP_011323851.1 FGRM|XP_011326004.1 FGRM|XP_011326006.1 FGRM|XP_011326039.1 MGRA|XP_003847842.1 MGRA|XP_003851843.1 MORY|QBZ53626.1 MORY|QBZ57892.1 NCRA|XP_960210.1 NCRA|XP_962208.1 SSCL|APA08540.1 SSCL|APA09927.1

>Orthogroup35: MLAR|XP_007403643.1 MLAR|XP_007403773.1 MLAR|XP_007404326.1 MLAR|XP_007404327.1 MLAR|XP_007404391.1 MLAR|XP_007404417.1 MLAR|XP_007404681.1 MLAR|XP_007404842.1 MLAR|XP_007405372.1 MLAR|XP_007405423.1 MLAR|XP_007405520.1 MLAR|XP_007405796.1 MLAR|XP_007406306.1 MLAR|XP_007406639.1 MLAR|XP_007406971.1 MLAR|XP_007407074.1 MLAR|XP_007407169.1 MLAR|XP_007407325.1 MLAR|XP_007407683.1 MLAR|XP_007408198.1 MLAR|XP_007408209.1 MLAR|XP_007408359.1 MLAR|XP_007408652.1 MLAR|XP_007408799.1 MLAR|XP_007408931.1 MLAR|XP_007409026.1 MLAR|XP_007409158.1 MLAR|XP_007409210.1 MLAR|XP_007409260.1 MLAR|XP_007409320.1 MLAR|XP_007409335.1 MLAR|XP_007409486.1 MLAR|XP_007409487.1 MLAR|XP_007409503.1 MLAR|XP_007409586.1 MLAR|XP_007409916.1 MLAR|XP_007409995.1 MLAR|XP_007410107.1 MLAR|XP_007411102.1 MLAR|XP_007411136.1 MLAR|XP_007411156.1 MLAR|XP_007411157.1 MLAR|XP_007411232.1 MLAR|XP_007411567.1 MLAR|XP_007411577.1 MLAR|XP_007411712.1 MLAR|XP_007411910.1 MLAR|XP_007412868.1 MLAR|XP_007412919.1 MLAR|XP_007413135.1 MLAR|XP_007413284.1 MLAR|XP_007413504.1 MLAR|XP_007413586.1 MLAR|XP_007413838.1 MLAR|XP_007413887.1 MLAR|XP_007414312.1 MLAR|XP_007414327.1 MLAR|XP_007414894.1 MLAR|XP_007414902.1 MLAR|XP_007415105.1 MLAR|XP_007415110.1 MLAR|XP_007415264.1 MLAR|XP_007415290.1 MLAR|XP_007415477.1 MLAR|XP_007415618.1 MLAR|XP_007415848.1 MLAR|XP_007416041.1 MLAR|XP_007416228.1 MLAR|XP_007416386.1 MLAR|XP_007416517.1 MLAR|XP_007416724.1 MLAR|XP_007417471.1 MLAR|XP_007418007.1 MLAR|XP_007418020.1 MLAR|XP_007418307.1 MLAR|XP_007418414.1 MLAR|XP_007418666.1 MLAR|XP_007418704.1 MLAR|XP_007419254.1 MLAR|XP_007419310.1 MLAR|XP_007419492.1 MLAR|XP_007419696.1

>Orthogroup36: ANID|CBF81783.1 BCIN|XP_001546796.1 BCIN|XP_001550787.2 BCIN|XP_001553843.1 BCIN|XP_001560446.2 BCIN|XP_024545915.1 BCIN|XP_024547843.1 BCIN|XP_024551122.1 BCIN|XP_024552916.1 BCIN|XP_024553623.1 BCIN|XP_024553964.1 BGRA|VDB89895.1 CFRU|XP_031878225.1 CFRU|XP_031879117.1 CFRU|XP_031882221.1 CFRU|XP_031885161.1 CFRU|XP_031885597.1 CFRU|XP_031889164.1 CFRU|XP_031889287.1 CFRU|XP_031893087.1 CFRU|XP_031893164.1 CGLO|KAF3798824.1 CGLO|KAF3803145.1 CGLO|KAF3803306.1 CGLO|KAF3804348.1 CGLO|KAF3804702.1 CGLO|KAF3808548.1 CGLO|KAF3809562.1 CGLO|KAF3810050.1 CGLO|KAF3810290.1 CGLO|KAF3810334.1 CHIG|XP_018152609.1 CHIG|XP_018153023.1 CHIG|XP_018154881.1 CHIG|XP_018155624.1 CHIG|XP_018155654.1 CHIG|XP_018158247.1 CHIG|XP_018158452.1 CHIG|XP_018158976.1 CHIG|XP_018160126.1 CHIG|XP_018163188.1 CHIG|XP_018164249.1 CVIN|KAF4918321.1 CVIN|KAF4927572.1 CVIN|KAF4928337.1 CVIN|KAF4930101.1 CVYL|A01031 CVYL|A03742 CVYL|A04039 FGRM|XP_011317677.1 FGRM|XP_011318344.1 FGRM|XP_011319605.1 FGRM|XP_011320698.1 FGRM|XP_011320873.1 FGRM|XP_011321020.1 FGRM|XP_011322652.1 FGRM|XP_011322981.1 FGRM|XP_011323249.1 FGRM|XP_011325981.1 FGRM|XP_011327107.1 MGRA|XP_003851367.1 MGRA|XP_003855939.1 MGRA|XP_003856917.1 MORY|QBZ58484.1 MORY|QBZ61972.1 NCRA|XP_011393667.1 NCRA|XP_011394847.1 NCRA|XP_955809.1 NCRA|XP_956503.2 NCRA|XP_956504.2 NCRA|XP_956812.3 NCRA|XP_958290.2 NCRA|XP_958984.1 NCRA|XP_958992.3 NCRA|XP_960276.1 NCRA|XP_962721.1 SSCL|APA07331.1 SSCL|APA07332.1 SSCL|APA07458.1 SSCL|APA08917.1 SSCL|APA16225.1

>Orthogroup37: MLAR|XP_007404367.1 MLAR|XP_007404421.1 MLAR|XP_007404422.1 MLAR|XP_007404423.1 MLAR|XP_007404700.1 MLAR|XP_007404702.1 MLAR|XP_007405189.1 MLAR|XP_007405333.1 MLAR|XP_007405872.1 MLAR|XP_007405873.1 MLAR|XP_007405950.1 MLAR|XP_007406039.1 MLAR|XP_007406754.1 MLAR|XP_007407750.1 MLAR|XP_007407929.1 MLAR|XP_007407930.1 MLAR|XP_007408092.1 MLAR|XP_007408093.1 MLAR|XP_007408095.1 MLAR|XP_007408233.1 MLAR|XP_007408375.1 MLAR|XP_007408454.1 MLAR|XP_007408554.1 MLAR|XP_007408645.1 MLAR|XP_007408737.1 MLAR|XP_007409161.1 MLAR|XP_007409436.1 MLAR|XP_007409528.1 MLAR|XP_007409529.1 MLAR|XP_007409698.1 MLAR|XP_007409699.1 MLAR|XP_007410024.1 MLAR|XP_007410217.1 MLAR|XP_007411055.1 MLAR|XP_007411056.1 MLAR|XP_007411251.1 MLAR|XP_007411654.1 MLAR|XP_007411655.1 MLAR|XP_007411656.1 MLAR|XP_007411914.1 MLAR|XP_007411933.1 MLAR|XP_007411934.1 MLAR|XP_007412013.1 MLAR|XP_007412432.1 MLAR|XP_007412433.1 MLAR|XP_007412800.1 MLAR|XP_007413148.1 MLAR|XP_007413149.1 MLAR|XP_007413150.1 MLAR|XP_007413621.1 MLAR|XP_007413622.1 MLAR|XP_007414170.1 MLAR|XP_007414444.1 MLAR|XP_007414445.1 MLAR|XP_007414485.1 MLAR|XP_007414488.1 MLAR|XP_007415450.1 MLAR|XP_007415711.1 MLAR|XP_007416057.1 MLAR|XP_007416120.1 MLAR|XP_007416700.1 MLAR|XP_007416702.1 MLAR|XP_007417014.1 MLAR|XP_007417015.1 MLAR|XP_007417018.1 MLAR|XP_007417105.1 MLAR|XP_007417143.1 MLAR|XP_007417161.1 MLAR|XP_007417195.1 MLAR|XP_007417658.1 MLAR|XP_007417751.1 MLAR|XP_007417793.1 MLAR|XP_007418161.1 MLAR|XP_007418677.1 MLAR|XP_007419034.1 MLAR|XP_007419259.1 MLAR|XP_007419260.1 MLAR|XP_007419327.1 MLAR|XP_007419328.1 MLAR|XP_007419412.1 MLAR|XP_007419413.1

>Orthogroup38: ANID|CBF69720.1 ANID|CBF73447.1 ANID|CBF78390.1 ANID|CBF81471.1 BCIN|XP_001552301.1 BCIN|XP_001553165.1 BCIN|XP_001555629.1 BCIN|XP_001559665.1 BCIN|XP_024548317.1 BCIN|XP_024548513.1 BCIN|XP_024551063.1 CFRU|XP_031876343.1 CFRU|XP_031877912.1 CFRU|XP_031877966.1 CFRU|XP_031880102.1 CFRU|XP_031881649.1 CFRU|XP_031883538.1 CFRU|XP_031883802.1 CFRU|XP_031883962.1 CFRU|XP_031886704.1 CFRU|XP_031891218.1 CGLO|KAF3799815.1 CGLO|KAF3804048.1 CGLO|KAF3804510.1 CGLO|KAF3804975.1 CGLO|KAF3805360.1 CGLO|KAF3805692.1 CGLO|KAF3809050.1 CGLO|KAF3810854.1 CGLO|KAF3810966.1 CGLO|KAF3811808.1 CGLO|KAF3811858.1 CHIG|XP_018151727.1 CHIG|XP_018154876.1 CHIG|XP_018156752.1 CHIG|XP_018159478.1 CHIG|XP_018160175.1 CHIG|XP_018163595.1 CVIN|KAF4907230.1 CVIN|KAF4907231.1 CVIN|KAF4907245.1 CVIN|KAF4907990.1 CVIN|KAF4909527.1 CVIN|KAF4911103.1 CVIN|KAF4920573.1 CVIN|KAF4920677.1 CVIN|KAF4924519.1 CVIN|KAF4924878.1 CVIN|KAF4927074.1 CVIN|KAF4927606.1 CVYL|A02183 CVYL|A02210 CVYL|A02292 CVYL|A04226 CVYL|A06198 CVYL|A07008 CVYL|A09694 CVYL|A09799 CVYL|A09855 CVYL|A11009 CVYL|A12811 FGRM|XP_011315669.1 FGRM|XP_011319514.1 FGRM|XP_011319612.1 FGRM|XP_011321394.1 FGRM|XP_011324229.1 FGRM|XP_011325223.1 FGRM|XP_011325309.1 FGRM|XP_011325381.1 FGRM|XP_011325743.1 FGRM|XP_011327154.1 MGRA|XP_003848548.1 MGRA|XP_003857361.1 MORY|QBZ53989.1 MORY|QBZ62969.1 MORY|QBZ64776.1 NCRA|XP_963278.1 SSCL|APA11140.1 SSCL|APA12566.1

>Orthogroup39: ANID|CBF70575.1 ANID|CBF71554.1 ANID|CBF75994.1 ANID|CBF78577.1 ANID|CBF79044.1 BCIN|XP_024546919.1 BCIN|XP_024548506.1 BGRA|VDB89533.1 CFRU|XP_031875635.1 CFRU|XP_031877059.1 CFRU|XP_031877099.1 CFRU|XP_031878533.1 CFRU|XP_031880667.1 CFRU|XP_031881142.1 CFRU|XP_031881339.1 CFRU|XP_031889199.1 CFRU|XP_031891145.1 CFRU|XP_031891295.1 CGLO|KAF3797623.1 CGLO|KAF3798610.1 CGLO|KAF3803166.1 CGLO|KAF3804012.1 CGLO|KAF3804619.1 CGLO|KAF3805749.1 CGLO|KAF3809879.1 CGLO|KAF3809951.1 CGLO|KAF3811088.1 CHIG|XP_018152977.1 CHIG|XP_018153206.1 CHIG|XP_018153923.1 CHIG|XP_018155509.1 CHIG|XP_018157415.1 CHIG|XP_018161436.1 CHIG|XP_018161531.1 CHIG|XP_018162124.1 CHIG|XP_018163599.1 CVIN|KAF4911082.1 CVIN|KAF4919098.1 CVIN|KAF4926612.1 CVIN|KAF4928352.1 CVIN|KAF4929429.1 CVIN|KAF4930103.1 CVIN|KAF4930623.1 CVIN|KAF4930721.1 CVYL|A01053 CVYL|A01672 CVYL|A01737 CVYL|A04836 CVYL|A05262 CVYL|A09707 CVYL|A09834 CVYL|A11077 FGRM|XP_011315666.1 FGRM|XP_011318200.1 FGRM|XP_011318268.1 FGRM|XP_011318518.1 FGRM|XP_011319669.1 FGRM|XP_011319921.1 FGRM|XP_011321043.1 FGRM|XP_011322459.1 FGRM|XP_011322975.1 FGRM|XP_011325199.1 FGRM|XP_011325660.1 FGRM|XP_011328712.1 MGRA|XP_003849000.1 MGRA|XP_003851043.1 MGRA|XP_003852322.1 MGRA|XP_003853319.1 MGRA|XP_003856140.1 MGRA|XP_003856665.1 MORY|QBZ55491.1 MORY|QBZ59123.1 NCRA|XP_960988.3 NCRA|XP_961779.1 NCRA|XP_963122.1 NCRA|XP_965499.1 SSCL|APA05824.1 SSCL|APA12136.1

>Orthogroup40: ANID|CBF69477.1 ANID|CBF74724.1 ANID|CBF75787.1 ANID|CBF86562.1 ANID|CBF86663.1 ANID|CBF87439.1 BCIN|XP_024546468.1 BCIN|XP_024546848.1 BCIN|XP_024549741.1 BCIN|XP_024551284.1 BCIN|XP_024552365.1 CFRU|XP_031877076.1 CFRU|XP_031879333.1 CFRU|XP_031881439.1 CFRU|XP_031883941.1 CFRU|XP_031885191.1 CFRU|XP_031887184.1 CFRU|XP_031887673.1 CFRU|XP_031888590.1 CFRU|XP_031890900.1 CFRU|XP_031891472.1 CFRU|XP_031892770.1 CGLO|KAF3797077.1 CGLO|KAF3800333.1 CGLO|KAF3803626.1 CGLO|KAF3804503.1 CGLO|KAF3807158.1 CGLO|KAF3808564.1 CGLO|KAF3809384.1 CGLO|KAF3811759.1 CHIG|XP_018156231.1 CHIG|XP_018157072.1 CHIG|XP_018159811.1 CHIG|XP_018160329.1 CHIG|XP_018160989.1 CHIG|XP_018161718.1 CHIG|XP_018162819.1 CHIG|XP_018163836.1 CVIN|KAF4899033.1 CVIN|KAF4900277.1 CVIN|KAF4906492.1 CVIN|KAF4913943.1 CVIN|KAF4917563.1 CVIN|KAF4918881.1 CVIN|KAF4924512.1 CVYL|A03125 CVYL|A03561 CVYL|A04218 CVYL|A07309 CVYL|A10632 CVYL|A11581 CVYL|A14559 FGRM|XP_011317512.1 FGRM|XP_011319891.1 FGRM|XP_011322415.1 FGRM|XP_011323027.1 FGRM|XP_011323724.1 FGRM|XP_011326420.1 FGRM|XP_011326541.1 MGRA|XP_003854389.1 MORY|QBZ53719.1 MORY|QBZ54702.1 MORY|QBZ56168.1 MORY|QBZ61823.1 MORY|QBZ63115.1 MORY|QBZ63231.1 MORY|QBZ63709.1 MORY|QBZ63995.1 MORY|QBZ66587.1 NCRA|XP_011393292.1 NCRA|XP_011393387.1 NCRA|XP_958819.2 NCRA|XP_959059.2 SSCL|APA05673.1 SSCL|APA06665.1 SSCL|APA10265.1 SSCL|APA13289.1

>Orthogroup41: ANID|CBF85186.1 ANID|CBF87102.1 ANID|CBF87366.1 BCIN|XP_001549315.1 BCIN|XP_024548713.1 BGRA|VDB94001.1 CFRU|XP_031877452.1 CFRU|XP_031877998.1 CFRU|XP_031879318.1 CFRU|XP_031880017.1 CFRU|XP_031880410.1 CFRU|XP_031880900.1 CFRU|XP_031884300.1 CFRU|XP_031892304.1 CGLO|KAF3797131.1 CGLO|KAF3798977.1 CGLO|KAF3799604.1 CGLO|KAF3806987.1 CGLO|KAF3807229.1 CGLO|KAF3808652.1 CHIG|XP_018151678.1 CHIG|XP_018152700.1 CHIG|XP_018152701.1 CHIG|XP_018152841.1 CHIG|XP_018153111.1 CHIG|XP_018154599.1 CHIG|XP_018156530.1 CHIG|XP_018159061.1 CHIG|XP_018159610.1 CHIG|XP_018160720.1 CHIG|XP_018160868.1 CHIG|XP_018161247.1 CHIG|XP_018161856.1 CHIG|XP_018164609.1 CVIN|KAF4896912.1 CVIN|KAF4909735.1 CVIN|KAF4918784.1 CVIN|KAF4920029.1 CVIN|KAF4923458.1 CVIN|KAF4926193.1 CVIN|KAF4926495.1 CVIN|KAF4926886.1 CVYL|A01018 CVYL|A06281 CVYL|A07896 CVYL|A08105 CVYL|A10180 CVYL|A10571 CVYL|A11210 CVYL|A11889 CVYL|A13466 FGRM|XP_011316543.1 FGRM|XP_011319294.1 FGRM|XP_011321913.1 FGRM|XP_011323260.1 FGRM|XP_011325345.1 MGRA|XP_003847740.1 MGRA|XP_003848303.1 MGRA|XP_003855184.1 MGRA|XP_003857544.1 MLAR|XP_007405002.1 MLAR|XP_007405033.1 MLAR|XP_007406915.1 MLAR|XP_007409998.1 MLAR|XP_007410394.1 MLAR|XP_007410570.1 MLAR|XP_007411475.1 MORY|QBZ53428.1 MORY|QBZ61009.1 MORY|QBZ64152.1 MORY|QBZ65452.1 NCRA|XP_956509.3 NCRA|XP_958259.1 NCRA|XP_963967.2 NCRA|XP_964148.3 SSCL|APA09984.1 SSCL|APA12116.1

>Orthogroup42: ANID|CBF85688.1 ANID|CBF86904.1 ANID|CBF87506.1 BCIN|XP_001554986.1 BCIN|XP_001560867.1 BCIN|XP_024551837.1 BGRA|VDB90458.1 CFRU|XP_031876518.1 CFRU|XP_031877316.1 CFRU|XP_031878871.1 CFRU|XP_031884928.1 CFRU|XP_031885153.1 CFRU|XP_031891222.1 CFRU|XP_031891227.1 CFRU|XP_031891645.1 CFRU|XP_031892338.1 CGLO|KAF3797014.1 CGLO|KAF3797152.1 CGLO|KAF3797885.1 CGLO|KAF3797887.1 CGLO|KAF3799909.1 CGLO|KAF3802619.1 CGLO|KAF3804727.1 CGLO|KAF3807660.1 CGLO|KAF3812181.1 CHIG|XP_018151904.1 CHIG|XP_018153413.1 CHIG|XP_018153670.1 CHIG|XP_018153780.1 CHIG|XP_018156777.1 CHIG|XP_018157093.1 CHIG|XP_018161634.1 CHIG|XP_018161637.1 CHIG|XP_018161804.1 CHIG|XP_018162883.1 CVIN|KAF4892828.1 CVIN|KAF4895818.1 CVIN|KAF4903974.1 CVIN|KAF4905512.1 CVIN|KAF4920958.1 CVIN|KAF4923096.1 CVIN|KAF4923348.1 CVIN|KAF4927779.1 CVYL|A00206 CVYL|A00379 CVYL|A01915 CVYL|A05322 CVYL|A05324 CVYL|A05391 CVYL|A06720 CVYL|A09612 CVYL|A10547 FGRM|XP_011321484.1 FGRM|XP_011322165.1 FGRM|XP_011324230.1 FGRM|XP_011324448.1 FGRM|XP_011326051.1 FGRM|XP_011326160.1 MGRA|XP_003847665.1 MLAR|XP_007405287.1 MLAR|XP_007413158.1 MLAR|XP_007413819.1 MLAR|XP_007413821.1 MLAR|XP_007417306.1 MORY|QBZ56729.1 MORY|QBZ59347.1 MORY|QBZ59453.1 MORY|QBZ60674.1 MORY|QBZ64097.1 MORY|QBZ64293.1 MORY|QBZ65515.1 NCRA|XP_001728217.2 NCRA|XP_958437.3 NCRA|XP_959693.2 SSCL|APA07769.1 SSCL|APA08080.1 SSCL|APA14741.1

>Orthogroup43: ANID|CBF69379.1 ANID|CBF73623.1 ANID|CBF78333.1 ANID|CBF84231.1 ANID|CBF87203.1 ANID|CBF88879.1 BCIN|XP_001548505.1 BCIN|XP_001552101.2 BCIN|XP_024549711.1 BCIN|XP_024550026.1 BCIN|XP_024551237.1 CFRU|XP_031876679.1 CFRU|XP_031879010.1 CFRU|XP_031880076.1 CFRU|XP_031881977.1 CFRU|XP_031882357.1 CFRU|XP_031883699.1 CFRU|XP_031886202.1 CFRU|XP_031886283.1 CFRU|XP_031889787.1 CFRU|XP_031891772.1 CFRU|XP_031891968.1 CFRU|XP_031893580.1 CGLO|KAF3798865.1 CGLO|KAF3800150.1 CGLO|KAF3800208.1 CGLO|KAF3801253.1 CGLO|KAF3801919.1 CGLO|KAF3806041.1 CGLO|KAF3806384.1 CGLO|KAF3806582.1 CGLO|KAF3809416.1 CGLO|KAF3809512.1 CHIG|XP_018153373.1 CHIG|XP_018155112.1 CHIG|XP_018157621.1 CHIG|XP_018163208.1 CHIG|XP_018163209.1 CHIG|XP_018164785.1 CVIN|KAF4916324.1 CVIN|KAF4918578.1 CVIN|KAF4918697.1 CVIN|KAF4920335.1 CVIN|KAF4920404.1 CVIN|KAF4920463.1 CVIN|KAF4921936.1 CVIN|KAF4923230.1 CVIN|KAF4924645.1 CVIN|KAF4925862.1 CVYL|A03080 CVYL|A06078 CVYL|A06844 CVYL|A07838 CVYL|A08505 CVYL|A09071 CVYL|A09286 CVYL|A10210 CVYL|A13945 FGRM|XP_011321298.1 FGRM|XP_011322937.1 FGRM|XP_011327305.1 MGRA|XP_003851356.1 MGRA|XP_003851421.1 MGRA|XP_003856963.1 MORY|QBZ56881.1 MORY|QBZ58276.1 MORY|QBZ64301.1 NCRA|XP_959425.2 NCRA|XP_960542.1 SSCL|APA05290.1 SSCL|APA05366.1 SSCL|APA05466.1 SSCL|APA08094.1 SSCL|APA09233.1 SSCL|APA10314.1 SSCL|APA12131.1

>Orthogroup44: MLAR|XP_007403736.1 MLAR|XP_007403737.1 MLAR|XP_007404170.1 MLAR|XP_007404258.1 MLAR|XP_007405339.1 MLAR|XP_007405471.1 MLAR|XP_007405503.1 MLAR|XP_007405567.1 MLAR|XP_007405568.1 MLAR|XP_007406322.1 MLAR|XP_007406472.1 MLAR|XP_007406473.1 MLAR|XP_007406712.1 MLAR|XP_007406713.1 MLAR|XP_007406815.1 MLAR|XP_007407156.1 MLAR|XP_007407234.1 MLAR|XP_007407304.1 MLAR|XP_007407706.1 MLAR|XP_007407715.1 MLAR|XP_007407716.1 MLAR|XP_007407718.1 MLAR|XP_007408391.1 MLAR|XP_007408392.1 MLAR|XP_007408430.1 MLAR|XP_007408749.1 MLAR|XP_007408750.1 MLAR|XP_007408751.1 MLAR|XP_007408928.1 MLAR|XP_007409357.1 MLAR|XP_007409828.1 MLAR|XP_007410182.1 MLAR|XP_007410253.1 MLAR|XP_007410732.1 MLAR|XP_007410757.1 MLAR|XP_007410897.1 MLAR|XP_007410963.1 MLAR|XP_007411227.1 MLAR|XP_007411311.1 MLAR|XP_007411367.1 MLAR|XP_007411911.1 MLAR|XP_007412174.1 MLAR|XP_007412795.1 MLAR|XP_007413271.1 MLAR|XP_007413581.1 MLAR|XP_007413764.1 MLAR|XP_007413949.1 MLAR|XP_007413950.1 MLAR|XP_007414330.1 MLAR|XP_007414707.1 MLAR|XP_007415212.1 MLAR|XP_007416759.1 MLAR|XP_007417102.1 MLAR|XP_007417187.1 MLAR|XP_007417315.1 MLAR|XP_007417328.1 MLAR|XP_007417338.1 MLAR|XP_007417449.1 MLAR|XP_007417568.1 MLAR|XP_007417589.1 MLAR|XP_007417773.1 MLAR|XP_007418014.1 MLAR|XP_007418016.1 MLAR|XP_007418017.1 MLAR|XP_007418365.1 MLAR|XP_007418593.1 MLAR|XP_007418594.1 MLAR|XP_007418663.1 MLAR|XP_007418664.1 MLAR|XP_007418930.1 MLAR|XP_007419238.1 MLAR|XP_007419287.1 MLAR|XP_007419391.1 MLAR|XP_007419519.1 MLAR|XP_007419621.1 MLAR|XP_007419833.1

>Orthogroup45: ANID|CBF75531.1 ANID|CBF77420.1 ANID|CBF80699.1 ANID|CBF83899.1 ANID|CBF87420.1 BCIN|XP_001552246.1 BCIN|XP_024546165.1 BCIN|XP_024546993.1 BCIN|XP_024547567.1 BCIN|XP_024549373.1 CFRU|XP_031876270.1 CFRU|XP_031876461.1 CFRU|XP_031880803.1 CFRU|XP_031883164.1 CFRU|XP_031885570.1 CFRU|XP_031885908.1 CFRU|XP_031888186.1 CFRU|XP_031890952.1 CFRU|XP_031892114.1 CFRU|XP_031892380.1 CFRU|XP_031893012.1 CGLO|KAF3797277.1 CGLO|KAF3798013.1 CGLO|KAF3800062.1 CGLO|KAF3802206.1 CGLO|KAF3805992.1 CGLO|KAF3806983.1 CGLO|KAF3808281.1 CGLO|KAF3809597.1 CGLO|KAF3810027.1 CGLO|KAF3811989.1 CHIG|XP_018150561.1 CHIG|XP_018150562.1 CHIG|XP_018151101.1 CHIG|XP_018152662.1 CHIG|XP_018154322.1 CHIG|XP_018157274.1 CHIG|XP_018158075.1 CHIG|XP_018159297.1 CHIG|XP_018159611.1 CHIG|XP_018165011.1 CVIN|KAF4889564.1 CVIN|KAF4915598.1 CVIN|KAF4917926.1 CVIN|KAF4918765.1 CVIN|KAF4919343.1 CVIN|KAF4921122.1 CVIN|KAF4924090.1 CVIN|KAF4925712.1 CVIN|KAF4930434.1 CVYL|A00618 CVYL|A02628 CVYL|A03192 CVYL|A05518 CVYL|A06277 CVYL|A06761 CVYL|A07524 CVYL|A08344 CVYL|A10413 CVYL|A12585 FGRM|XP_011315611.1 FGRM|XP_011317903.1 FGRM|XP_011318307.1 FGRM|XP_011319498.1 FGRM|XP_011319918.1 FGRM|XP_011322957.1 FGRM|XP_011324993.1 MORY|QBZ60744.1 MORY|QBZ64249.1 MORY|QBZ66039.1 NCRA|XP_011393225.1 NCRA|XP_960029.1 SSCL|APA05941.1 SSCL|APA06439.1 SSCL|APA12901.1

>Orthogroup46: ANID|CBF71797.1 ANID|CBF71811.1 ANID|CBF74895.1 ANID|CBF79149.1 ANID|CBF80818.1 ANID|CBF82175.1 ANID|CBF82267.1 ANID|CBF84567.1 ANID|CBF86762.1 ANID|CBF87196.1 ANID|CBF88837.1 BCIN|XP_001547022.2 BCIN|XP_001547188.1 BCIN|XP_001556101.1 BCIN|XP_001559904.1 BCIN|XP_024547382.1 BCIN|XP_024547578.1 BCIN|XP_024548833.1 BCIN|XP_024549323.1 BCIN|XP_024550039.1 BGRA|VCU39564.1 CFRU|XP_031877084.1 CFRU|XP_031877258.1 CFRU|XP_031878987.1 CFRU|XP_031880779.1 CFRU|XP_031887285.1 CFRU|XP_031887529.1 CFRU|XP_031890175.1 CGLO|KAF3802832.1 CGLO|KAF3803305.1 CGLO|KAF3806788.1 CGLO|KAF3806874.1 CGLO|KAF3807842.1 CGLO|KAF3809322.1 CGLO|KAF3811107.1 CHIG|XP_018151666.1 CHIG|XP_018152961.1 CHIG|XP_018156160.1 CHIG|XP_018156539.1 CHIG|XP_018156877.1 CHIG|XP_018158299.1 CHIG|XP_018159452.1 CHIG|XP_018159588.1 CHIG|XP_018162972.1 CHIG|XP_018162973.1 CHIG|XP_018163043.1 CVIN|KAF4895981.1 CVIN|KAF4905802.1 CVIN|KAF4911089.1 CVIN|KAF4914235.1 CVIN|KAF4922589.1 CVIN|KAF4924173.1 CVIN|KAF4928692.1 CVYL|A00769 CVYL|A01522 CVYL|A02450 CVYL|A02520 CVYL|A03741 CVYL|A05179 CVYL|A07977 FGRM|XP_011318561.1 FGRM|XP_011319293.1 FGRM|XP_011320984.1 FGRM|XP_011322964.1 FGRM|XP_011324384.1 MORY|QBZ64964.1 NCRA|XP_959754.2 NCRA|XP_959846.2 NCRA|XP_962191.1 SSCL|APA05649.1 SSCL|APA07730.1 SSCL|APA07731.1 SSCL|APA09746.1 SSCL|APA12866.1

>Orthogroup47: ANID|CBF78915.1 ANID|CBF79598.1 ANID|CBF82125.1 ANID|CBF83913.1 BCIN|XP_001553590.2 BCIN|XP_001554721.1 BCIN|XP_001556430.1 BCIN|XP_001558272.1 BCIN|XP_024549627.1 BGRA|VCU39288.1 BGRA|VDB83697.1 CFRU|XP_031879691.1 CFRU|XP_031880955.1 CFRU|XP_031881788.1 CFRU|XP_031884010.1 CFRU|XP_031884938.1 CFRU|XP_031885123.1 CFRU|XP_031885436.1 CFRU|XP_031886446.1 CFRU|XP_031891108.1 CGLO|KAF3797829.1 CGLO|KAF3798189.1 CGLO|KAF3798655.1 CGLO|KAF3800450.1 CGLO|KAF3801031.1 CGLO|KAF3801483.1 CGLO|KAF3805028.1 CGLO|KAF3807766.1 CGLO|KAF3811883.1 CHIG|XP_018151908.1 CHIG|XP_018155167.1 CHIG|XP_018157505.1 CHIG|XP_018160176.1 CHIG|XP_018162965.1 CHIG|XP_018163497.1 CVIN|KAF4900988.1 CVIN|KAF4902817.1 CVIN|KAF4908690.1 CVIN|KAF4916394.1 CVIN|KAF4919126.1 CVIN|KAF4919457.1 CVIN|KAF4920525.1 CVIN|KAF4920562.1 CVIN|KAF4927141.1 CVYL|A00352 CVYL|A00830 CVYL|A01835 CVYL|A04934 CVYL|A06176 CVYL|A06524 CVYL|A09393 CVYL|A12646 CVYL|A13012 FGRM|XP_011317383.1 FGRM|XP_011318251.1 FGRM|XP_011319640.1 FGRM|XP_011322258.1 FGRM|XP_011322914.1 MGRA|XP_003847891.1 MGRA|XP_003855507.1 MGRA|XP_003857137.1 MORY|QBZ53617.1 MORY|QBZ53765.1 MORY|QBZ57866.1 MORY|QBZ58348.1 MORY|QBZ62508.1 MORY|QBZ64210.1 MORY|QBZ64428.1 MORY|QBZ64978.1 SSCL|APA05756.1 SSCL|APA06425.1 SSCL|APA06574.1 SSCL|APA13668.1 SSCL|APA15462.1

>Orthogroup48: ANID|CBF71839.1 ANID|CBF79242.1 ANID|CBF80487.1 ANID|CBF85555.1 BCIN|XP_024545898.1 BCIN|XP_024546765.1 BCIN|XP_024547721.1 BCIN|XP_024551155.1 BCIN|XP_024551712.1 CFRU|XP_031875985.1 CFRU|XP_031878464.1 CFRU|XP_031878830.1 CFRU|XP_031879403.1 CFRU|XP_031880113.1 CFRU|XP_031880367.1 CFRU|XP_031884606.1 CFRU|XP_031888898.1 CFRU|XP_031891768.1 CFRU|XP_031892880.1 CGLO|KAF3799137.1 CGLO|KAF3799174.1 CGLO|KAF3799748.1 CGLO|KAF3799810.1 CGLO|KAF3807230.1 CGLO|KAF3808608.1 CGLO|KAF3811794.1 CHIG|XP_018151323.1 CHIG|XP_018151545.1 CHIG|XP_018151575.1 CHIG|XP_018151759.1 CHIG|XP_018154111.1 CHIG|XP_018155044.1 CHIG|XP_018156684.1 CHIG|XP_018161565.1 CHIG|XP_018163173.1 CHIG|XP_018163481.1 CHIG|XP_018163482.1 CHIG|XP_018164608.1 CVIN|KAF4907999.1 CVIN|KAF4911610.1 CVIN|KAF4914906.1 CVIN|KAF4924923.1 CVIN|KAF4926162.1 CVYL|A01900 CVYL|A09698 CVYL|A09699 CVYL|A09700 CVYL|A11142 CVYL|A11347 CVYL|A12371 CVYL|A12800 FGRM|XP_011319443.1 FGRM|XP_011327617.1 MGRA|XP_003856913.1 MORY|QBZ57568.1 MORY|QBZ57655.1 MORY|QBZ57672.1 MORY|QBZ58412.1 MORY|QBZ60730.1 MORY|QBZ63157.1 MORY|QBZ64454.1 MORY|QBZ64455.1 MORY|QBZ64960.1 MORY|QBZ64967.1 MORY|QBZ66514.1 NCRA|XP_011395280.1 SSCL|APA06275.1 SSCL|APA07155.1 SSCL|APA07157.1 SSCL|APA15016.1 SSCL|APA15164.1 SSCL|APA15392.1 SSCL|APA15881.1

>Orthogroup49: ANID|CBF76870.1 ANID|CBF79672.1 ANID|CBF80350.1 ANID|CBF84638.1 ANID|CBF87442.1 ANID|CBF87998.1 ANID|CBF88817.1 BCIN|XP_001555249.2 BCIN|XP_024546005.1 BCIN|XP_024549700.1 BCIN|XP_024551902.1 BCIN|XP_024552672.1 CFRU|XP_031875698.1 CFRU|XP_031876103.1 CFRU|XP_031877969.1 CFRU|XP_031878188.1 CFRU|XP_031879160.1 CFRU|XP_031884954.1 CFRU|XP_031886674.1 CFRU|XP_031891005.1 CGLO|KAF3798124.1 CGLO|KAF3807011.1 CGLO|KAF3807378.1 CGLO|KAF3807490.1 CGLO|KAF3808510.1 CGLO|KAF3810885.1 CGLO|KAF3811706.1 CGLO|KAF3812190.1 CHIG|XP_018151580.1 CHIG|XP_018151829.1 CHIG|XP_018153420.1 CHIG|XP_018153944.1 CHIG|XP_018154915.1 CHIG|XP_018156277.1 CHIG|XP_018156861.1 CHIG|XP_018158744.1 CHIG|XP_018159677.1 CHIG|XP_018161966.1 CVIN|KAF4887324.1 CVIN|KAF4905042.1 CVIN|KAF4907617.1 CVIN|KAF4916359.1 CVIN|KAF4917720.1 CVIN|KAF4920973.1 CVIN|KAF4927952.1 CVYL|A00198 CVYL|A02259 CVYL|A05012 CVYL|A06304 CVYL|A08235 CVYL|A10641 CVYL|A11629 CVYL|A12729 FGRM|XP_011320479.1 FGRM|XP_011321004.1 FGRM|XP_011321788.1 FGRM|XP_011321957.1 FGRM|XP_011322960.1 FGRM|XP_011325975.1 FGRM|XP_011326015.1 FGRM|XP_011328426.1 MGRA|XP_003848153.1 MGRA|XP_003848348.1 MGRA|XP_003849789.1 MORY|QBZ57908.1 MORY|QBZ59886.1 MORY|QBZ61807.1 NCRA|XP_011394124.1 NCRA|XP_960553.1 SSCL|APA05634.1 SSCL|APA08596.1 SSCL|APA08670.1 SSCL|APA15543.1

>Orthogroup50: ANID|CBF73911.1 ANID|CBF74938.1 ANID|CBF79108.1 ANID|CBF84724.1 ANID|CBF87204.1 CFRU|XP_031877317.1 CFRU|XP_031877449.1 CFRU|XP_031878954.1 CFRU|XP_031882714.1 CFRU|XP_031883079.1 CFRU|XP_031883795.1 CFRU|XP_031883831.1 CFRU|XP_031888826.1 CFRU|XP_031890713.1 CFRU|XP_031891653.1 CFRU|XP_031891987.1 CGLO|KAF3799915.1 CGLO|KAF3800709.1 CGLO|KAF3801285.1 CGLO|KAF3801577.1 CGLO|KAF3804275.1 CGLO|KAF3804600.1 CGLO|KAF3805679.1 CGLO|KAF3806634.1 CGLO|KAF3807893.1 CHIG|XP_018153788.1 CHIG|XP_018160083.1 CHIG|XP_018161990.1 CHIG|XP_018163077.1 CHIG|XP_018163199.1 CHIG|XP_018163264.1 CHIG|XP_018163341.1 CHIG|XP_018163562.1 CHIG|XP_018163572.1 CHIG|XP_018164228.1 CHIG|XP_018164843.1 CVIN|KAF4891283.1 CVIN|KAF4903973.1 CVIN|KAF4909058.1 CVIN|KAF4915220.1 CVIN|KAF4918914.1 CVIN|KAF4920672.1 CVIN|KAF4921113.1 CVIN|KAF4921586.1 CVIN|KAF4922616.1 CVYL|A00496 CVYL|A00720 CVYL|A03958 CVYL|A05280 CVYL|A07803 CVYL|A09607 CVYL|A09871 CVYL|A11675 CVYL|A12347 CVYL|A12556 FGRM|XP_011315570.1 FGRM|XP_011315663.1 FGRM|XP_011315684.1 FGRM|XP_011322094.1 FGRM|XP_011322436.1 FGRM|XP_011322627.1 FGRM|XP_011323293.1 FGRM|XP_011325163.1 FGRM|XP_011325302.1 FGRM|XP_011325717.1 FGRM|XP_011327111.1 FGRM|XP_011327401.1 MGRA|XP_003847854.1 MGRA|XP_003856916.1 MORY|QBZ54779.1 MORY|QBZ59187.1 MORY|QBZ66484.1

>Orthogroup51: ANID|CBF78968.1 ANID|CBF80368.1 ANID|CBF82374.1 ANID|CBF84544.1 BCIN|XP_001550950.1 BCIN|XP_001552369.2 BCIN|XP_001552463.1 BCIN|XP_001559473.1 BCIN|XP_024551932.1 CFRU|XP_031877244.1 CFRU|XP_031878584.1 CFRU|XP_031883223.1 CFRU|XP_031883954.1 CFRU|XP_031884233.1 CFRU|XP_031885739.1 CFRU|XP_031887006.1 CFRU|XP_031887846.1 CFRU|XP_031889937.1 CFRU|XP_031890287.1 CFRU|XP_031892890.1 CGLO|KAF3798381.1 CGLO|KAF3798749.1 CGLO|KAF3802193.1 CGLO|KAF3805077.1 CGLO|KAF3806799.1 CGLO|KAF3808174.1 CGLO|KAF3810042.1 CGLO|KAF3810411.1 CGLO|KAF3811062.1 CGLO|KAF3811232.1 CHIG|XP_018151683.1 CHIG|XP_018153118.1 CHIG|XP_018156489.1 CHIG|XP_018157761.1 CHIG|XP_018160455.1 CHIG|XP_018162668.1 CHIG|XP_018162921.1 CHIG|XP_018163238.1 CHIG|XP_018163436.1 CVIN|KAF4908470.1 CVIN|KAF4915263.1 CVIN|KAF4917941.1 CVIN|KAF4922753.1 CVIN|KAF4922812.1 CVIN|KAF4924263.1 CVIN|KAF4927434.1 CVIN|KAF4931188.1 CVYL|A00528 CVYL|A02638 CVYL|A03200 CVYL|A04434 CVYL|A06482 CVYL|A08201 CVYL|A09168 CVYL|A11390 FGRM|XP_011319409.1 FGRM|XP_011320621.1 FGRM|XP_011321721.1 FGRM|XP_011322264.1 FGRM|XP_011322742.1 FGRM|XP_011327386.1 FGRM|XP_011327709.1 MORY|QBZ56311.1 MORY|QBZ60796.1 MORY|QBZ61242.1 NCRA|XP_958051.1 SSCL|APA07141.1 SSCL|APA07637.1 SSCL|APA07638.1 SSCL|APA11187.1 SSCL|APA14440.1

>Orthogroup52: ANID|CBF70143.1 BCIN|XP_001546526.1 BCIN|XP_024546846.1 BCIN|XP_024550517.1 BCIN|XP_024552595.1 BGRA|VDB93027.1 BGRA|VDB96336.1 CFRU|XP_031877100.1 CFRU|XP_031878999.1 CFRU|XP_031882117.1 CFRU|XP_031886402.1 CFRU|XP_031887243.1 CFRU|XP_031888807.1 CGLO|KAF3799275.1 CGLO|KAF3801496.1 CGLO|KAF3801568.1 CGLO|KAF3803996.1 CGLO|KAF3806105.1 CGLO|KAF3806296.1 CGLO|KAF3807903.1 CGLO|KAF3812017.1 CHIG|XP_018150930.1 CHIG|XP_018151993.1 CHIG|XP_018161427.1 CHIG|XP_018162249.1 CHIG|XP_018164224.1 CHIG|XP_018164562.1 CVIN|KAF4902335.1 CVIN|KAF4923953.1 CVIN|KAF4924817.1 CVIN|KAF4925165.1 CVYL|A07407 CVYL|A08805 CVYL|A10165 CVYL|A11888 CVYL|A12563 FGRM|XP_011324337.1 FGRM|XP_011325980.1 MGRA|XP_003853182.1 MGRA|XP_003856737.1 MLAR|XP_007410399.1 MLAR|XP_007412271.1 MLAR|XP_007414363.1 MLAR|XP_007414367.1 MLAR|XP_007414368.1 MLAR|XP_007414598.1 MLAR|XP_007414733.1 MLAR|XP_007414838.1 MLAR|XP_007417412.1 MLAR|XP_007417414.1 MLAR|XP_007417415.1 MLAR|XP_007417459.1 MLAR|XP_007417470.1 MORY|QBZ53443.1 MORY|QBZ54120.1 MORY|QBZ54446.1 MORY|QBZ55507.1 MORY|QBZ59909.1 MORY|QBZ60765.1 MORY|QBZ62992.1 MORY|QBZ63442.1 MORY|QBZ64172.1 MORY|QBZ65090.1 MORY|QBZ65769.1 NCRA|XP_956233.2 NCRA|XP_957767.1 NCRA|XP_957968.2 NCRA|XP_963161.1 SSCL|APA11262.1 SSCL|APA15126.1

>Orthogroup53: MLAR|XP_007403521.1 MLAR|XP_007403576.1 MLAR|XP_007403964.1 MLAR|XP_007403981.1 MLAR|XP_007404227.1 MLAR|XP_007404267.1 MLAR|XP_007404488.1 MLAR|XP_007404816.1 MLAR|XP_007405108.1 MLAR|XP_007405404.1 MLAR|XP_007405480.1 MLAR|XP_007405866.1 MLAR|XP_007406544.1 MLAR|XP_007407363.1 MLAR|XP_007407509.1 MLAR|XP_007407748.1 MLAR|XP_007407779.1 MLAR|XP_007407804.1 MLAR|XP_007407932.1 MLAR|XP_007408258.1 MLAR|XP_007408376.1 MLAR|XP_007408426.1 MLAR|XP_007408932.1 MLAR|XP_007409094.1 MLAR|XP_007409461.1 MLAR|XP_007409709.1 MLAR|XP_007410143.1 MLAR|XP_007410423.1 MLAR|XP_007410539.1 MLAR|XP_007410637.1 MLAR|XP_007410913.1 MLAR|XP_007411079.1 MLAR|XP_007411214.1 MLAR|XP_007411578.1 MLAR|XP_007411690.1 MLAR|XP_007412378.1 MLAR|XP_007412452.1 MLAR|XP_007412459.1 MLAR|XP_007412574.1 MLAR|XP_007412716.1 MLAR|XP_007412758.1 MLAR|XP_007413061.1 MLAR|XP_007413854.1 MLAR|XP_007413953.1 MLAR|XP_007414060.1 MLAR|XP_007414078.1 MLAR|XP_007414194.1 MLAR|XP_007414257.1 MLAR|XP_007414270.1 MLAR|XP_007414690.1 MLAR|XP_007414965.1 MLAR|XP_007415172.1 MLAR|XP_007415562.1 MLAR|XP_007415636.1 MLAR|XP_007415843.1 MLAR|XP_007415949.1 MLAR|XP_007416189.1 MLAR|XP_007416689.1 MLAR|XP_007416755.1 MLAR|XP_007417885.1 MLAR|XP_007418605.1 MLAR|XP_007418832.1 MLAR|XP_007419201.1 MLAR|XP_007419435.1 MLAR|XP_007419443.1 MLAR|XP_007419597.1 MLAR|XP_007419792.1 MLAR|XP_007419796.1

>Orthogroup54: ANID|CBF69576.1 ANID|CBF74212.1 ANID|CBF85206.1 BCIN|XP_024546135.1 BCIN|XP_024548090.1 BCIN|XP_024549543.1 BCIN|XP_024553253.1 BCIN|XP_024553654.1 BCIN|XP_024553655.1 CFRU|XP_031875476.1 CFRU|XP_031877889.1 CFRU|XP_031882830.1 CFRU|XP_031883559.1 CFRU|XP_031884081.1 CFRU|XP_031884307.1 CFRU|XP_031886183.1 CFRU|XP_031887649.1 CFRU|XP_031891787.1 CGLO|KAF3800012.1 CGLO|KAF3800013.1 CGLO|KAF3801124.1 CGLO|KAF3801340.1 CGLO|KAF3803344.1 CGLO|KAF3807739.1 CGLO|KAF3810938.1 CHIG|XP_018153041.1 CHIG|XP_018153208.1 CHIG|XP_018154899.1 CHIG|XP_018155238.1 CHIG|XP_018155409.1 CHIG|XP_018156549.1 CHIG|XP_018158354.1 CHIG|XP_018158970.1 CHIG|XP_018159776.1 CHIG|XP_018163507.1 CVIN|KAF4909510.1 CVIN|KAF4910741.1 CVIN|KAF4920199.1 CVIN|KAF4920200.1 CVIN|KAF4926866.1 CVIN|KAF4930388.1 CVYL|A02209 CVYL|A03617 CVYL|A05720 CVYL|A07744 CVYL|A09792 CVYL|A10227 CVYL|A10228 CVYL|A12386 FGRM|XP_011327114.1 MGRA|XP_003847983.1 MLAR|XP_007405465.1 MLAR|XP_007407851.1 MLAR|XP_007408255.1 MLAR|XP_007408995.1 MORY|QBZ66334.1 NCRA|XP_001728006.1 NCRA|XP_011395087.1 NCRA|XP_958335.2 NCRA|XP_960129.3 NCRA|XP_963151.3 SSCL|APA07147.1 SSCL|APA07876.1 SSCL|APA08621.1 SSCL|APA08622.1 SSCL|APA10045.1 SSCL|APA15443.1

>Orthogroup55: ANID|CBF83314.1 ANID|CBF84412.1 ANID|CBF84606.1 ANID|CBF85105.1 ANID|CBF89359.1 BCIN|XP_001548382.2 BCIN|XP_024546642.1 BCIN|XP_024547053.1 BCIN|XP_024549037.1 BCIN|XP_024549225.1 BCIN|XP_024550645.1 BCIN|XP_024552083.1 BGRA|VCU40519.1 BGRA|VDB85750.1 CFRU|XP_031875478.1 CFRU|XP_031875610.1 CFRU|XP_031877398.1 CFRU|XP_031878950.1 CFRU|XP_031884693.1 CFRU|XP_031885144.1 CFRU|XP_031887068.1 CFRU|XP_031888353.1 CFRU|XP_031890699.1 CGLO|KAF3799078.1 CGLO|KAF3803742.1 CGLO|KAF3804857.1 CGLO|KAF3807796.1 CGLO|KAF3807898.1 CGLO|KAF3811430.1 CHIG|XP_018150692.1 CHIG|XP_018153171.1 CHIG|XP_018158181.1 CHIG|XP_018160671.1 CHIG|XP_018160672.1 CHIG|XP_018163072.1 CHIG|XP_018164389.1 CVIN|KAF4890057.1 CVIN|KAF4917883.1 CVIN|KAF4918265.1 CVIN|KAF4921145.1 CVIN|KAF4921525.1 CVIN|KAF4922613.1 CVIN|KAF4931502.1 CVYL|A00716 CVYL|A04628 CVYL|A11451 CVYL|A11703 CVYL|A12216 FGRM|XP_011317675.1 FGRM|XP_011318560.1 FGRM|XP_011323190.1 FGRM|XP_011325897.1 FGRM|XP_011326846.1 FGRM|XP_011327571.1 FGRM|XP_011328375.1 MGRA|XP_003854900.1 MORY|QBZ58824.1 MORY|QBZ59242.1 MORY|QBZ65261.1 NCRA|XP_959387.2 NCRA|XP_962732.1 SSCL|APA06964.1 SSCL|APA07626.1 SSCL|APA09274.1 SSCL|APA11463.1 SSCL|APA12867.1 SSCL|APA14512.1

>Orthogroup56: ANID|CBF74901.1 ANID|CBF80318.1 ANID|CBF87450.1 BCIN|XP_001548196.2 BCIN|XP_001552368.1 BCIN|XP_024550954.1 BCIN|XP_024551225.1 CFRU|XP_031876119.1 CFRU|XP_031876860.1 CFRU|XP_031877945.1 CFRU|XP_031881795.1 CFRU|XP_031884629.1 CFRU|XP_031885681.1 CFRU|XP_031891412.1 CFRU|XP_031892306.1 CFRU|XP_031893188.1 CFRU|XP_031893386.1 CGLO|KAF3796961.1 CGLO|KAF3797828.1 CGLO|KAF3797987.1 CGLO|KAF3800841.1 CGLO|KAF3804746.1 CGLO|KAF3807361.1 CGLO|KAF3809523.1 CGLO|KAF3810254.1 CGLO|KAF3810923.1 CHIG|XP_018150853.1 CHIG|XP_018152667.1 CHIG|XP_018154912.1 CHIG|XP_018156865.1 CHIG|XP_018157944.1 CHIG|XP_018158325.1 CHIG|XP_018159306.1 CHIG|XP_018161831.1 CVIN|KAF4893184.1 CVIN|KAF4898617.1 CVIN|KAF4900989.1 CVIN|KAF4903555.1 CVIN|KAF4903964.1 CVIN|KAF4909515.1 CVIN|KAF4910803.1 CVIN|KAF4915586.1 CVIN|KAF4918553.1 CVIN|KAF4920057.1 CVYL|A02222 CVYL|A02856 CVYL|A05374 CVYL|A05819 CVYL|A08366 CVYL|A08496 CVYL|A11082 CVYL|A13013 FGRM|XP_011321952.1 FGRM|XP_011322074.1 FGRM|XP_011322238.1 FGRM|XP_011325385.1 MGRA|XP_003852501.1 MGRA|XP_003853421.1 MLAR|XP_007403528.1 MLAR|XP_007404054.1 MLAR|XP_007404127.1 MLAR|XP_007412301.1 MORY|QBZ66380.1 NCRA|XP_960108.2 SSCL|APA05817.1 SSCL|APA10116.1

>Orthogroup57: ANID|CBF69883.1 ANID|CBF75444.1 ANID|CBF80391.1 ANID|CBF80759.1 ANID|CBF83286.1 ANID|CBF89614.1 BCIN|XP_001550120.1 BCIN|XP_024546754.1 BCIN|XP_024548743.1 BCIN|XP_024550668.1 BGRA|VCU39421.1 CFRU|XP_031879144.1 CFRU|XP_031880817.1 CFRU|XP_031881027.1 CFRU|XP_031884325.1 CFRU|XP_031884681.1 CFRU|XP_031884858.1 CFRU|XP_031888972.1 CFRU|XP_031893363.1 CGLO|KAF3799217.1 CGLO|KAF3799668.1 CGLO|KAF3799999.1 CGLO|KAF3801017.1 CGLO|KAF3807069.1 CGLO|KAF3809686.1 CGLO|KAF3809830.1 CHIG|XP_018152092.1 CHIG|XP_018152501.1 CHIG|XP_018153050.1 CHIG|XP_018153333.1 CHIG|XP_018157908.1 CHIG|XP_018158312.1 CHIG|XP_018158887.1 CHIG|XP_018159729.1 CVIN|KAF4895373.1 CVIN|KAF4900576.1 CVIN|KAF4909314.1 CVIN|KAF4912984.1 CVIN|KAF4920244.1 CVIN|KAF4925028.1 CVIN|KAF4925029.1 CVIN|KAF4926917.1 CVIN|KAF4930696.1 CVYL|A01782 CVYL|A02511 CVYL|A05831 CVYL|A05892 CVYL|A06362 CVYL|A09584 CVYL|A10112 CVYL|A10215 FGRM|XP_011316218.1 FGRM|XP_011321947.1 FGRM|XP_011321955.1 FGRM|XP_011325971.1 FGRM|XP_011327456.1 FGRM|XP_011328004.1 MGRA|XP_003849072.1 MGRA|XP_003849571.1 MORY|QBZ56753.1 MORY|QBZ64219.1 NCRA|XP_959994.1 SSCL|APA06394.1 SSCL|APA09757.1 SSCL|APA11375.1

>Orthogroup58: ANID|CBF74606.1 ANID|CBF76247.1 ANID|CBF82403.1 ANID|CBF84541.1 ANID|CBF85644.1 ANID|CBF86932.1 ANID|CBF87147.1 BCIN|XP_001549481.1 BCIN|XP_001550319.2 BCIN|XP_001551816.1 BCIN|XP_001560561.1 BCIN|XP_024549445.1 BCIN|XP_024550095.1 BGRA|VCU39568.1 BGRA|VCU40050.1 CFRU|XP_031879882.1 CFRU|XP_031880000.1 CFRU|XP_031882586.1 CFRU|XP_031887070.1 CFRU|XP_031893598.1 CGLO|KAF3798924.1 CGLO|KAF3800864.1 CGLO|KAF3811390.1 CHIG|XP_018150835.1 CHIG|XP_018151032.1 CHIG|XP_018152883.1 CHIG|XP_018152958.1 CHIG|XP_018164869.1 CVIN|KAF4894129.1 CVIN|KAF4908222.1 CVIN|KAF4915572.1 CVIN|KAF4919707.1 CVIN|KAF4925434.1 CVYL|A04582 CVYL|A07844 CVYL|A07946 CVYL|A08887 CVYL|A11103 FGRM|XP_011317293.1 FGRM|XP_011321585.1 FGRM|XP_011324437.1 FGRM|XP_011326064.1 FGRM|XP_011327369.1 MGRA|XP_003849364.1 MGRA|XP_003850562.1 MGRA|XP_003850624.1 MGRA|XP_003851142.1 MGRA|XP_003852793.1 MGRA|XP_003854119.1 MGRA|XP_003857615.1 MGRA|XP_003857633.1 MLAR|XP_007411601.1 MLAR|XP_007417809.1 MLAR|XP_007417818.1 MORY|QBZ54097.1 MORY|QBZ61957.1 MORY|QBZ63714.1 MORY|QBZ65245.1 NCRA|XP_958937.1 NCRA|XP_961039.2 NCRA|XP_964364.3 SSCL|APA09218.1 SSCL|APA10724.1 SSCL|APA12809.1 SSCL|APA16223.1

>Orthogroup59: ANID|CBF81670.1 BCIN|XP_024551302.1 BGRA|VDB87573.1 BGRA|VDB93269.1 CFRU|XP_031875834.1 CFRU|XP_031881870.1 CFRU|XP_031886960.1 CFRU|XP_031889614.1 CFRU|XP_031889888.1 CFRU|XP_031889962.1 CFRU|XP_031891757.1 CFRU|XP_031891940.1 CGLO|KAF3800107.1 CGLO|KAF3805556.1 CGLO|KAF3805964.1 CGLO|KAF3810010.1 CGLO|KAF3810417.1 CGLO|KAF3810557.1 CGLO|KAF3811329.1 CHIG|XP_018157722.1 CHIG|XP_018158409.1 CHIG|XP_018158993.1 CHIG|XP_018159043.1 CHIG|XP_018162653.1 CHIG|XP_018164202.1 CHIG|XP_018165043.1 CVIN|KAF4899749.1 CVIN|KAF4908457.1 CVIN|KAF4913009.1 CVIN|KAF4917809.1 CVIN|KAF4925854.1 CVIN|KAF4927246.1 CVYL|A02609 CVYL|A04512 CVYL|A11385 CVYL|A13635 CVYL|A13758 FGRM|XP_011316519.1 FGRM|XP_011319519.1 FGRM|XP_011319597.1 FGRM|XP_011319725.1 FGRM|XP_011320303.1 FGRM|XP_011320766.1 FGRM|XP_011320819.1 FGRM|XP_011322405.1 FGRM|XP_011322424.1 FGRM|XP_011322817.1 FGRM|XP_011322839.1 FGRM|XP_011322840.1 FGRM|XP_011325824.1 FGRM|XP_011328363.1 MGRA|XP_003852424.1 MGRA|XP_003852996.1 MGRA|XP_003853885.1 MGRA|XP_003856006.1 MGRA|XP_003857144.1 MORY|QBZ54532.1 MORY|QBZ55187.1 MORY|QBZ55847.1 MORY|QBZ60012.1 MORY|QBZ65875.1 NCRA|XP_957384.1 NCRA|XP_958219.1 NCRA|XP_959818.1 SSCL|APA10586.1

>Orthogroup60: ANID|CBF71484.1 ANID|CBF71848.1 ANID|CBF71850.1 ANID|CBF71852.1 ANID|CBF74006.1 ANID|CBF76028.1 ANID|CBF76085.1 ANID|CBF76161.1 ANID|CBF76163.1 ANID|CBF76165.1 ANID|CBF79347.1 ANID|CBF80194.1 ANID|CBF80196.1 ANID|CBF80218.1 ANID|CBF80219.1 ANID|CBF80226.1 ANID|CBF80228.1 ANID|CBF80230.1 ANID|CBF80252.1 ANID|CBF80253.1 ANID|CBF80272.1 ANID|CBF80836.1 ANID|CBF80843.1 ANID|CBF80845.1 ANID|CBF80847.1 ANID|CBF81153.1 ANID|CBF81155.1 ANID|CBF81157.1 ANID|CBF81164.1 ANID|CBF81844.1 ANID|CBF81848.1 ANID|CBF81850.1 ANID|CBF81852.1 ANID|CBF81853.1 ANID|CBF82237.1 ANID|CBF82246.1 ANID|CBF84162.1 ANID|CBF84163.1 ANID|CBF84238.1 ANID|CBF86037.1 ANID|CBF87221.1 ANID|CBF87223.1 ANID|CBF89285.1 ANID|CBF89286.1 ANID|CBF89288.1 ANID|CBF89334.1 ANID|CBF89606.1 CFRU|XP_031875306.1 CFRU|XP_031879236.1 CFRU|XP_031881202.1 CGLO|KAF3803874.1 CHIG|XP_018151903.1 CHIG|XP_018155492.1 MGRA|XP_003855599.1 NCRA|XP_011394023.1 NCRA|XP_958002.1 SSCL|APA07562.1 SSCL|APA10029.1 SSCL|APA10429.1 SSCL|APA10430.1 SSCL|APA11524.1 SSCL|APA11926.1 SSCL|APA12632.1 SSCL|APA13902.1

>Orthogroup61: CFRU|XP_031876143.1 CFRU|XP_031877260.1 CFRU|XP_031878865.1 CFRU|XP_031880891.1 CFRU|XP_031881330.1 CFRU|XP_031884810.1 CFRU|XP_031884867.1 CFRU|XP_031885761.1 CFRU|XP_031889216.1 CFRU|XP_031891724.1 CFRU|XP_031893185.1 CGLO|KAF3800091.1 CGLO|KAF3800934.1 CGLO|KAF3802571.1 CGLO|KAF3806841.1 CGLO|KAF3808736.1 CGLO|KAF3809639.1 CGLO|KAF3810076.1 CGLO|KAF3810286.1 CGLO|KAF3812158.1 CGLO|KAF3812177.1 CHIG|XP_018152569.1 CHIG|XP_018153389.1 CHIG|XP_018153401.1 CHIG|XP_018155639.1 CHIG|XP_018157919.1 CHIG|XP_018159520.1 CHIG|XP_018159614.1 CHIG|XP_018159839.1 CHIG|XP_018161348.1 CHIG|XP_018165032.1 CVIN|KAF4892844.1 CVIN|KAF4896990.1 CVIN|KAF4896992.1 CVIN|KAF4909296.1 CVIN|KAF4912746.1 CVIN|KAF4918614.1 CVIN|KAF4918749.1 CVIN|KAF4918777.1 CVIN|KAF4920917.1 CVIN|KAF4920959.1 CVIN|KAF4929589.1 CVYL|A00210 CVYL|A00211 CVYL|A00236 CVYL|A00926 CVYL|A01859 CVYL|A02488 CVYL|A02673 CVYL|A06266 CVYL|A06785 CVYL|A08411 CVYL|A08412 CVYL|A11263 FGRM|XP_011324989.1 MORY|QBZ60133.1 MORY|QBZ60140.1 MORY|QBZ60226.1 MORY|QBZ61810.1 MORY|QBZ63683.1 MORY|QBZ65138.1 MORY|QBZ65283.1 MORY|QBZ66143.1 MORY|QBZ66300.1

>Orthogroup62: MLAR|XP_007403518.1 MLAR|XP_007403738.1 MLAR|XP_007403962.1 MLAR|XP_007404144.1 MLAR|XP_007404154.1 MLAR|XP_007404468.1 MLAR|XP_007404656.1 MLAR|XP_007404936.1 MLAR|XP_007405613.1 MLAR|XP_007405748.1 MLAR|XP_007405865.1 MLAR|XP_007406185.1 MLAR|XP_007406476.1 MLAR|XP_007406811.1 MLAR|XP_007407044.1 MLAR|XP_007408053.1 MLAR|XP_007408077.1 MLAR|XP_007408434.1 MLAR|XP_007409030.1 MLAR|XP_007409463.1 MLAR|XP_007409869.1 MLAR|XP_007410022.1 MLAR|XP_007410194.1 MLAR|XP_007410425.1 MLAR|XP_007410472.1 MLAR|XP_007410641.1 MLAR|XP_007411226.1 MLAR|XP_007411229.1 MLAR|XP_007411535.1 MLAR|XP_007411780.1 MLAR|XP_007411810.1 MLAR|XP_007412082.1 MLAR|XP_007412121.1 MLAR|XP_007412460.1 MLAR|XP_007412653.1 MLAR|XP_007413062.1 MLAR|XP_007413694.1 MLAR|XP_007414040.1 MLAR|XP_007414290.1 MLAR|XP_007414326.1 MLAR|XP_007415266.1 MLAR|XP_007415355.1 MLAR|XP_007415391.1 MLAR|XP_007415563.1 MLAR|XP_007415891.1 MLAR|XP_007416336.1 MLAR|XP_007416619.1 MLAR|XP_007416643.1 MLAR|XP_007416746.1 MLAR|XP_007416924.1 MLAR|XP_007417169.1 MLAR|XP_007417320.1 MLAR|XP_007417640.1 MLAR|XP_007417680.1 MLAR|XP_007418295.1 MLAR|XP_007418733.1 MLAR|XP_007418743.1 MLAR|XP_007418942.1 MLAR|XP_007419106.1 MLAR|XP_007419152.1 MLAR|XP_007419319.1 MLAR|XP_007419483.1 MLAR|XP_007419503.1 MLAR|XP_007419598.1

>Orthogroup63: ANID|CBF82768.1 ANID|CBF84674.1 ANID|CBF84697.1 ANID|CBF88205.1 BCIN|XP_001555698.2 BCIN|XP_024552402.1 BCIN|XP_024552815.1 BGRA|VCU40508.1 CFRU|XP_031875708.1 CFRU|XP_031876683.1 CFRU|XP_031876892.1 CFRU|XP_031884609.1 CFRU|XP_031885896.1 CFRU|XP_031888893.1 CFRU|XP_031891278.1 CGLO|KAF3798144.1 CGLO|KAF3799279.1 CGLO|KAF3799774.1 CGLO|KAF3799835.1 CGLO|KAF3801913.1 CGLO|KAF3811130.1 CHIG|XP_018151621.1 CHIG|XP_018154033.1 CHIG|XP_018158225.1 CHIG|XP_018159370.1 CHIG|XP_018159427.1 CHIG|XP_018159884.1 CHIG|XP_018160372.1 CHIG|XP_018161771.1 CHIG|XP_018163105.1 CHIG|XP_018163569.1 CVIN|KAF4898720.1 CVIN|KAF4918709.1 CVIN|KAF4918974.1 CVIN|KAF4924507.1 CVIN|KAF4924814.1 CVYL|A02351 CVYL|A04287 CVYL|A04970 CVYL|A05611 CVYL|A09679 CVYL|A10168 CVYL|A11321 FGRM|XP_011320868.1 FGRM|XP_011322385.1 FGRM|XP_011322968.1 FGRM|XP_011326936.1 MGRA|XP_003850482.1 MGRA|XP_003851519.1 MGRA|XP_003851984.1 MGRA|XP_003854601.1 MGRA|XP_003855050.1 MLAR|XP_007406515.1 MORY|QBZ53367.1 MORY|QBZ57801.1 MORY|QBZ59241.1 MORY|QBZ63434.1 NCRA|XP_956068.2 NCRA|XP_963599.1 SSCL|APA08595.1 SSCL|APA10098.1 SSCL|APA13283.1 SSCL|APA16014.1

>Orthogroup64: ANID|CBF70130.1 ANID|CBF73403.1 ANID|CBF78490.1 ANID|CBF83982.1 ANID|CBF83984.1 ANID|CBF84386.1 ANID|CBF85232.1 CFRU|XP_031877326.1 CFRU|XP_031877513.1 CFRU|XP_031879146.1 CFRU|XP_031881520.1 CFRU|XP_031889518.1 CGLO|KAF3797530.1 CGLO|KAF3799885.1 CGLO|KAF3799897.1 CGLO|KAF3805719.1 CGLO|KAF3805911.1 CGLO|KAF3807061.1 CHIG|XP_018151972.1 CHIG|XP_018155055.1 CHIG|XP_018156746.1 CHIG|XP_018156747.1 CHIG|XP_018159717.1 CHIG|XP_018162011.1 CHIG|XP_018164739.1 CHIG|XP_018164936.1 CVIN|KAF4898935.1 CVIN|KAF4907227.1 CVIN|KAF4907240.1 CVIN|KAF4920250.1 CVIN|KAF4921646.1 CVIN|KAF4921721.1 CVIN|KAF4926638.1 CVYL|A01979 CVYL|A06355 CVYL|A09623 CVYL|A09826 CVYL|A11005 CVYL|A11008 CVYL|A13798 FGRM|XP_011318060.1 FGRM|XP_011320950.1 FGRM|XP_011328654.1 MLAR|XP_007404958.1 MLAR|XP_007407615.1 MLAR|XP_007410813.1 MLAR|XP_007413322.1 MORY|QBZ53466.1 MORY|QBZ53467.1 MORY|QBZ57369.1 MORY|QBZ57670.1 MORY|QBZ63366.1 MORY|QBZ63546.1 MORY|QBZ63800.1 NCRA|XP_958702.1 NCRA|XP_958707.3 NCRA|XP_961748.1 NCRA|XP_961749.1 NCRA|XP_963069.2 NCRA|XP_963297.1 NCRA|XP_963743.2 NCRA|XP_963913.1

>Orthogroup65: ANID|CBF71329.1 ANID|CBF82101.1 ANID|CBF83180.1 ANID|CBF83840.1 BCIN|XP_001550227.1 BCIN|XP_001553166.1 BCIN|XP_024552814.1 BGRA|VCU39622.1 CFRU|XP_031877988.1 CFRU|XP_031879142.1 CFRU|XP_031879755.1 CFRU|XP_031881607.1 CFRU|XP_031883160.1 CFRU|XP_031885382.1 CFRU|XP_031889592.1 CFRU|XP_031891670.1 CFRU|XP_031893467.1 CGLO|KAF3798194.1 CGLO|KAF3799609.1 CGLO|KAF3800729.1 CGLO|KAF3805397.1 CGLO|KAF3809586.1 CGLO|KAF3810464.1 CHIG|XP_018154473.1 CHIG|XP_018154604.1 CHIG|XP_018156714.1 CHIG|XP_018156844.1 CHIG|XP_018159154.1 CHIG|XP_018159394.1 CHIG|XP_018163281.1 CHIG|XP_018164718.1 CVIN|KAF4916797.1 CVIN|KAF4917771.1 CVIN|KAF4919620.1 CVIN|KAF4924614.1 CVIN|KAF4926449.1 CVYL|A00475 CVYL|A04925 CVYL|A07600 CVYL|A11043 CVYL|A13470 CVYL|A14065 FGRM|XP_011316546.1 FGRM|XP_011319454.1 FGRM|XP_011321133.1 FGRM|XP_011325448.1 FGRM|XP_011327760.1 MGRA|XP_003847776.1 MGRA|XP_003850294.1 MGRA|XP_003852883.1 MLAR|XP_007410240.1 MLAR|XP_007410328.1 MORY|QBZ54872.1 MORY|QBZ57255.1 MORY|QBZ60830.1 MORY|QBZ64095.1 NCRA|XP_955927.2 NCRA|XP_958139.1 SSCL|APA05856.1 SSCL|APA07525.1 SSCL|APA16016.1 SSCL|APA16339.1

>Orthogroup66: ANID|CBF73646.1 ANID|CBF79014.1 ANID|CBF83114.1 ANID|CBF83706.1 ANID|CBF87084.1 BCIN|XP_001551034.1 BCIN|XP_001553893.2 BCIN|XP_001559121.1 BCIN|XP_024547202.1 BCIN|XP_024548059.1 BCIN|XP_024548311.1 CFRU|XP_031882816.1 CFRU|XP_031883583.1 CFRU|XP_031883700.1 CFRU|XP_031884110.1 CFRU|XP_031886392.1 CFRU|XP_031892931.1 CGLO|KAF3806269.1 CGLO|KAF3809442.1 CGLO|KAF3811008.1 CGLO|KAF3811913.1 CGLO|KAF3812052.1 CHIG|XP_018152475.1 CHIG|XP_018152718.1 CHIG|XP_018153025.1 CHIG|XP_018153273.1 CHIG|XP_018155124.1 CHIG|XP_018155150.1 CHIG|XP_018162096.1 CHIG|XP_018164163.1 CVIN|KAF4908452.1 CVIN|KAF4911843.1 CVIN|KAF4920413.1 CVIN|KAF4923706.1 CVIN|KAF4923964.1 CVIN|KAF4926901.1 CVYL|A06071 CVYL|A06145 CVYL|A08223 CVYL|A09779 CVYL|A11859 CVYL|A14243 FGRM|XP_011319019.1 FGRM|XP_011322041.1 FGRM|XP_011322244.1 FGRM|XP_011322465.1 FGRM|XP_011323079.1 FGRM|XP_011324391.1 FGRM|XP_011325255.1 MGRA|XP_003848877.1 MGRA|XP_003851919.1 MGRA|XP_003852847.1 MGRA|XP_003855923.1 MORY|QBZ55044.1 MORY|QBZ59889.1 MORY|QBZ63455.1 NCRA|XP_958043.1 SSCL|APA05829.1 SSCL|APA07499.1 SSCL|APA10417.1 SSCL|APA13054.1 SSCL|APA14130.1

>Orthogroup67: ANID|CBF75173.1 ANID|CBF86736.1 ANID|CBF89889.1 BCIN|XP_001547685.1 BCIN|XP_001551617.1 BCIN|XP_001553752.1 BCIN|XP_024547273.1 BCIN|XP_024549809.1 BCIN|XP_024549892.1 BCIN|XP_024550971.1 BCIN|XP_024553954.1 BGRA|VCU39990.1 CFRU|XP_031880483.1 CFRU|XP_031882572.1 CFRU|XP_031887015.1 CFRU|XP_031888359.1 CFRU|XP_031892573.1 CGLO|KAF3803611.1 CGLO|KAF3803889.1 CGLO|KAF3809157.1 CGLO|KAF3811319.1 CGLO|KAF3811577.1 CHIG|XP_018151048.1 CHIG|XP_018155977.1 CHIG|XP_018160545.1 CHIG|XP_018160842.1 CHIG|XP_018160843.1 CHIG|XP_018163851.1 CVIN|KAF4914893.1 CVIN|KAF4919809.1 CVIN|KAF4920366.1 CVIN|KAF4925472.1 CVIN|KAF4927243.1 CVYL|A01343 CVYL|A03428 CVYL|A04500 CVYL|A08896 CVYL|A14545 FGRM|XP_011323292.1 FGRM|XP_011324743.1 FGRM|XP_011326553.1 FGRM|XP_011327587.1 FGRM|XP_011327936.1 MGRA|XP_003852187.1 MLAR|XP_007404650.1 MLAR|XP_007407666.1 MLAR|XP_007409660.1 MLAR|XP_007410275.1 MLAR|XP_007416145.1 MLAR|XP_007416855.1 MORY|QBZ57443.1 MORY|QBZ58043.1 MORY|QBZ60865.1 MORY|QBZ63202.1 MORY|QBZ66221.1 NCRA|XP_961439.1 SSCL|APA05572.1 SSCL|APA06129.1 SSCL|APA13309.1 SSCL|APA14232.1 SSCL|APA14482.1 SSCL|APA15437.1

>Orthogroup68: ANID|CBF76107.1 ANID|CBF76113.1 ANID|CBF76478.1 ANID|CBF80461.1 ANID|CBF83927.1 ANID|CBF87969.1 BCIN|XP_001558536.2 BGRA|VDB95075.1 CFRU|XP_031876680.1 CFRU|XP_031877046.1 CFRU|XP_031878291.1 CFRU|XP_031881162.1 CFRU|XP_031887509.1 CFRU|XP_031887510.1 CFRU|XP_031889698.1 CFRU|XP_031890603.1 CFRU|XP_031890843.1 CGLO|KAF3798108.1 CGLO|KAF3801616.1 CGLO|KAF3801904.1 CGLO|KAF3802523.1 CGLO|KAF3804802.1 CGLO|KAF3805953.1 CGLO|KAF3807183.1 CGLO|KAF3811077.1 CHIG|XP_018151378.1 CHIG|XP_018153093.1 CHIG|XP_018154839.1 CHIG|XP_018158130.1 CHIG|XP_018159433.1 CHIG|XP_018159855.1 CHIG|XP_018164296.1 CVIN|KAF4895030.1 CVIN|KAF4918715.1 CVIN|KAF4918998.1 CVIN|KAF4920235.1 CVIN|KAF4920236.1 CVIN|KAF4922706.1 CVIN|KAF4927988.1 CVIN|KAF4929755.1 CVYL|A02115 CVYL|A02345 CVYL|A03589 CVYL|A08251 CVYL|A09719 CVYL|A12270 CVYL|A12520 FGRM|XP_011316969.1 FGRM|XP_011318315.1 FGRM|XP_011322557.1 FGRM|XP_011327294.1 FGRM|XP_011328203.1 MGRA|XP_003848956.1 MGRA|XP_003856736.1 MLAR|XP_007411716.1 MORY|QBZ53880.1 MORY|QBZ59190.1 MORY|QBZ60019.1 MORY|QBZ66228.1 NCRA|XP_956652.1 NCRA|XP_960371.2 SSCL|APA14675.1

>Orthogroup69: BGRA|VDB85982.1 CFRU|XP_031875298.1 CFRU|XP_031876132.1 CFRU|XP_031876737.1 CFRU|XP_031877124.1 CFRU|XP_031877294.1 CFRU|XP_031877340.1 CFRU|XP_031878686.1 CFRU|XP_031880080.1 CFRU|XP_031882174.1 CFRU|XP_031882334.1 CFRU|XP_031888596.1 CFRU|XP_031889568.1 CFRU|XP_031889964.1 CFRU|XP_031891521.1 CFRU|XP_031893502.1 CGLO|KAF3798684.1 CGLO|KAF3802011.1 CGLO|KAF3803841.1 CGLO|KAF3803983.1 CGLO|KAF3804364.1 CGLO|KAF3804371.1 CGLO|KAF3808078.1 CGLO|KAF3810291.1 CGLO|KAF3810410.1 CGLO|KAF3811800.1 CHIG|XP_018151083.1 CHIG|XP_018151084.1 CHIG|XP_018151754.1 CHIG|XP_018157380.1 CHIG|XP_018157472.1 CHIG|XP_018159605.1 CHIG|XP_018159845.1 CVIN|KAF4906648.1 CVIN|KAF4906957.1 CVIN|KAF4908469.1 CVIN|KAF4918329.1 CVIN|KAF4918334.1 CVIN|KAF4918480.1 CVIN|KAF4919774.1 CVIN|KAF4920769.1 CVIN|KAF4923183.1 CVIN|KAF4924876.1 CVIN|KAF4929351.1 CVYL|A02435 CVYL|A03039 CVYL|A04051 CVYL|A08155 CVYL|A08959 CVYL|A09421 CVYL|A10257 CVYL|A11391 CVYL|A13839 FGRM|XP_011316695.1 FGRM|XP_011319987.1 FGRM|XP_011321848.1 FGRM|XP_011324258.1 FGRM|XP_011328117.1 MORY|QBZ56364.1 MORY|QBZ62574.1 NCRA|XP_011394336.1 NCRA|XP_965421.2

>Orthogroup70: MLAR|XP_007403583.1 MLAR|XP_007403947.1 MLAR|XP_007404474.1 MLAR|XP_007405162.1 MLAR|XP_007405523.1 MLAR|XP_007405535.1 MLAR|XP_007405875.1 MLAR|XP_007405876.1 MLAR|XP_007406373.1 MLAR|XP_007406427.1 MLAR|XP_007406757.1 MLAR|XP_007406845.1 MLAR|XP_007406847.1 MLAR|XP_007407391.1 MLAR|XP_007407392.1 MLAR|XP_007407658.1 MLAR|XP_007407980.1 MLAR|XP_007408282.1 MLAR|XP_007408283.1 MLAR|XP_007408396.1 MLAR|XP_007408489.1 MLAR|XP_007408490.1 MLAR|XP_007409575.1 MLAR|XP_007409726.1 MLAR|XP_007410117.1 MLAR|XP_007410506.1 MLAR|XP_007410515.1 MLAR|XP_007410516.1 MLAR|XP_007410885.1 MLAR|XP_007412423.1 MLAR|XP_007412860.1 MLAR|XP_007412863.1 MLAR|XP_007413267.1 MLAR|XP_007413290.1 MLAR|XP_007414033.1 MLAR|XP_007414195.1 MLAR|XP_007414196.1 MLAR|XP_007414210.1 MLAR|XP_007414429.1 MLAR|XP_007414586.1 MLAR|XP_007415340.1 MLAR|XP_007415434.1 MLAR|XP_007416030.1 MLAR|XP_007416198.1 MLAR|XP_007416199.1 MLAR|XP_007416208.1 MLAR|XP_007417284.1 MLAR|XP_007417285.1 MLAR|XP_007418059.1 MLAR|XP_007418090.1 MLAR|XP_007418102.1 MLAR|XP_007418285.1 MLAR|XP_007418286.1 MLAR|XP_007418422.1 MLAR|XP_007418682.1 MLAR|XP_007419004.1 MLAR|XP_007419130.1 MLAR|XP_007419160.1 MLAR|XP_007419525.1 MLAR|XP_007419526.1 MLAR|XP_007419584.1 MLAR|XP_007419737.1

>Orthogroup71: MLAR|XP_007404420.1 MLAR|XP_007404696.1 MLAR|XP_007405055.1 MLAR|XP_007405060.1 MLAR|XP_007405183.1 MLAR|XP_007405334.1 MLAR|XP_007405870.1 MLAR|XP_007405948.1 MLAR|XP_007406038.1 MLAR|XP_007406050.1 MLAR|XP_007406752.1 MLAR|XP_007406753.1 MLAR|XP_007407046.1 MLAR|XP_007407744.1 MLAR|XP_007407828.1 MLAR|XP_007407931.1 MLAR|XP_007407963.1 MLAR|XP_007408373.1 MLAR|XP_007408374.1 MLAR|XP_007408425.1 MLAR|XP_007408448.1 MLAR|XP_007408450.1 MLAR|XP_007408451.1 MLAR|XP_007408452.1 MLAR|XP_007409162.1 MLAR|XP_007409437.1 MLAR|XP_007409530.1 MLAR|XP_007409696.1 MLAR|XP_007409697.1 MLAR|XP_007410023.1 MLAR|XP_007410214.1 MLAR|XP_007411054.1 MLAR|XP_007411653.1 MLAR|XP_007411932.1 MLAR|XP_007412016.1 MLAR|XP_007412118.1 MLAR|XP_007412120.1 MLAR|XP_007412431.1 MLAR|XP_007412793.1 MLAR|XP_007412798.1 MLAR|XP_007412799.1 MLAR|XP_007413147.1 MLAR|XP_007414443.1 MLAR|XP_007414486.1 MLAR|XP_007414489.1 MLAR|XP_007415610.1 MLAR|XP_007415710.1 MLAR|XP_007415951.1 MLAR|XP_007416121.1 MLAR|XP_007416473.1 MLAR|XP_007416698.1 MLAR|XP_007417019.1 MLAR|XP_007417081.1 MLAR|XP_007417144.1 MLAR|XP_007417637.1 MLAR|XP_007418945.1 MLAR|XP_007419032.1 MLAR|XP_007419033.1 MLAR|XP_007419257.1 MLAR|XP_007419258.1 MLAR|XP_007419322.1 MLAR|XP_007419414.1

>Orthogroup72: ANID|CBF74509.1 ANID|CBF75012.1 ANID|CBF78041.1 ANID|CBF80268.1 ANID|CBF83822.1 ANID|CBF83922.1 ANID|CBF84256.1 ANID|CBF85015.1 BCIN|XP_001546066.2 BCIN|XP_001546878.2 BCIN|XP_001554888.1 BCIN|XP_001555270.1 BCIN|XP_001560291.1 BCIN|XP_024549375.1 BCIN|XP_024551959.1 BCIN|XP_024552273.1 CFRU|XP_031880028.1 CFRU|XP_031884155.1 CFRU|XP_031886057.1 CFRU|XP_031889413.1 CFRU|XP_031889504.1 CGLO|KAF3801168.1 CGLO|KAF3803107.1 CGLO|KAF3805927.1 CGLO|KAF3811845.1 CHIG|XP_018150750.1 CHIG|XP_018151690.1 CHIG|XP_018156691.1 CHIG|XP_018158927.1 CVIN|KAF4911056.1 CVIN|KAF4911057.1 CVIN|KAF4911879.1 CVIN|KAF4917919.1 CVIN|KAF4924869.1 CVYL|A09797 CVYL|A12845 CVYL|A13785 CVYL|A14171 FGRM|XP_011319278.1 FGRM|XP_011320744.1 FGRM|XP_011321699.1 FGRM|XP_011322320.1 FGRM|XP_011327397.1 MGRA|XP_003847424.1 MGRA|XP_003847771.1 MGRA|XP_003850019.1 MGRA|XP_003851157.1 MGRA|XP_003851224.1 MGRA|XP_003852994.1 MGRA|XP_003854008.1 MORY|QBZ57507.1 MORY|QBZ60644.1 MORY|QBZ60673.1 NCRA|XP_961990.2 SSCL|APA08335.1 SSCL|APA08584.1 SSCL|APA08655.1 SSCL|APA08836.1 SSCL|APA08949.1 SSCL|APA13135.1 SSCL|APA14992.1

>Orthogroup73: ANID|CBF82898.1 ANID|CBF82901.1 ANID|CBF82995.1 ANID|CBF85398.1 ANID|CBF86068.1 BCIN|XP_024548728.1 BCIN|XP_024548958.1 BCIN|XP_024550061.1 BCIN|XP_024550870.1 BCIN|XP_024551485.1 BCIN|XP_024551667.1 BCIN|XP_024552011.1 BCIN|XP_024553003.1 BCIN|XP_024553468.1 BGRA|VDB93363.1 CFRU|XP_031881672.1 CFRU|XP_031881742.1 CFRU|XP_031885604.1 CFRU|XP_031893196.1 CGLO|KAF3800621.1 CGLO|KAF3804021.1 CGLO|KAF3807451.1 CGLO|KAF3810047.1 CHIG|XP_018150924.1 CHIG|XP_018157766.1 CHIG|XP_018159664.1 CVIN|KAF4917924.1 CVIN|KAF4918197.1 CVIN|KAF4918507.1 CVIN|KAF4925178.1 CVYL|A02643 CVYL|A08783 CVYL|A10960 CVYL|A13096 FGRM|XP_011315718.1 FGRM|XP_011317666.1 FGRM|XP_011317854.1 FGRM|XP_011318185.1 FGRM|XP_011320437.1 FGRM|XP_011320587.1 FGRM|XP_011320713.1 FGRM|XP_011322225.1 FGRM|XP_011323345.1 FGRM|XP_011325922.1 FGRM|XP_011327461.1 MGRA|XP_003848061.1 MGRA|XP_003849284.1 MGRA|XP_003851422.1 MGRA|XP_003852938.1 MGRA|XP_003853141.1 MGRA|XP_003853198.1 MGRA|XP_003855010.1 MGRA|XP_003856544.1 SSCL|APA06522.1 SSCL|APA09500.1 SSCL|APA10394.1 SSCL|APA11795.1 SSCL|APA12771.1 SSCL|APA13820.1 SSCL|APA14452.1 SSCL|APA14805.1

>Orthogroup74: BGRA|VCU38969.1 BGRA|VCU38970.1 BGRA|VCU38971.1 BGRA|VCU38995.1 BGRA|VCU38997.1 BGRA|VCU39004.1 BGRA|VCU39005.1 BGRA|VCU39180.1 BGRA|VCU39191.1 BGRA|VCU39192.1 BGRA|VCU39193.1 BGRA|VCU39195.1 BGRA|VCU39196.1 BGRA|VCU39197.1 BGRA|VCU39198.1 BGRA|VCU39200.1 BGRA|VCU39213.1 BGRA|VDB85698.1 BGRA|VDB85782.1 BGRA|VDB85783.1 BGRA|VDB85845.1 BGRA|VDB85847.1 BGRA|VDB88389.1 BGRA|VDB88395.1 BGRA|VDB88680.1 BGRA|VDB89056.1 BGRA|VDB89106.1 BGRA|VDB90488.1 BGRA|VDB90490.1 BGRA|VDB91285.1 BGRA|VDB91395.1 BGRA|VDB94460.1 BGRA|VDB96284.1 BGRA|VDB96414.1 BGRA|VDB96429.1 BGRA|VDB96430.1 BGRA|VDB96434.1 BGRA|VDB96435.1 BGRA|VDB96436.1 BGRA|VDB96437.1 BGRA|VDB96438.1 BGRA|VDB96439.1 BGRA|VDB96440.1 BGRA|VDB96442.1 BGRA|VDB96443.1 BGRA|VDB96445.1 BGRA|VDB96446.1 BGRA|VDB96447.1 BGRA|VDB96448.1 BGRA|VDB96451.1 BGRA|VDB96453.1 BGRA|VDB96454.1 BGRA|VDB96455.1 BGRA|VDB96456.1 BGRA|VDB96457.1 BGRA|VDB96459.1 BGRA|VDB96461.1 BGRA|VDB96462.1 BGRA|VDB96463.1 BGRA|VDB96464.1 BGRA|VDB96465.1

>Orthogroup75: MLAR|XP_007403762.1 MLAR|XP_007403804.1 MLAR|XP_007403853.1 MLAR|XP_007404013.1 MLAR|XP_007404159.1 MLAR|XP_007405193.1 MLAR|XP_007405348.1 MLAR|XP_007405507.1 MLAR|XP_007405739.1 MLAR|XP_007405879.1 MLAR|XP_007407281.1 MLAR|XP_007407557.1 MLAR|XP_007407598.1 MLAR|XP_007408443.1 MLAR|XP_007408711.1 MLAR|XP_007408876.1 MLAR|XP_007409187.1 MLAR|XP_007409445.1 MLAR|XP_007409885.1 MLAR|XP_007410002.1 MLAR|XP_007410690.1 MLAR|XP_007411899.1 MLAR|XP_007412080.1 MLAR|XP_007412168.1 MLAR|XP_007413383.1 MLAR|XP_007413798.1 MLAR|XP_007413977.1 MLAR|XP_007414119.1 MLAR|XP_007414211.1 MLAR|XP_007415192.1 MLAR|XP_007415661.1 MLAR|XP_007415720.1 MLAR|XP_007415721.1 MLAR|XP_007416425.1 MLAR|XP_007416437.1 MLAR|XP_007416621.1 MLAR|XP_007416649.1 MLAR|XP_007417086.1 MLAR|XP_007417197.1 MLAR|XP_007417299.1 MLAR|XP_007417301.1 MLAR|XP_007417373.1 MLAR|XP_007417934.1 MLAR|XP_007418095.1 MLAR|XP_007418110.1 MLAR|XP_007418204.1 MLAR|XP_007418329.1 MLAR|XP_007418440.1 MLAR|XP_007418476.1 MLAR|XP_007418559.1 MLAR|XP_007418579.1 MLAR|XP_007418767.1 MLAR|XP_007418892.1 MLAR|XP_007418982.1 MLAR|XP_007419208.1 MLAR|XP_007419433.1 MLAR|XP_007419481.1 MLAR|XP_007419588.1 MLAR|XP_007419602.1 MLAR|XP_007419654.1 MLAR|XP_007419828.1

>Orthogroup76: ANID|CBF69434.1 ANID|CBF73532.1 ANID|CBF80180.1 ANID|CBF80452.1 ANID|CBF87135.1 BCIN|XP_001556989.1 BCIN|XP_001558557.1 CFRU|XP_031877328.1 CFRU|XP_031881619.1 CFRU|XP_031881654.1 CFRU|XP_031881896.1 CFRU|XP_031886319.1 CFRU|XP_031888642.1 CFRU|XP_031888902.1 CFRU|XP_031890748.1 CGLO|KAF3797833.1 CGLO|KAF3799181.1 CGLO|KAF3799241.1 CGLO|KAF3799896.1 CGLO|KAF3805358.1 CGLO|KAF3806286.1 CGLO|KAF3811731.1 CHIG|XP_018151057.1 CHIG|XP_018151796.1 CHIG|XP_018153335.1 CHIG|XP_018156808.1 CHIG|XP_018157671.1 CHIG|XP_018158114.1 CHIG|XP_018159789.1 CHIG|XP_018161828.1 CVIN|KAF4906633.1 CVIN|KAF4908834.1 CVIN|KAF4914902.1 CVIN|KAF4921722.1 CVIN|KAF4922998.1 CVIN|KAF4923926.1 CVYL|A09624 CVYL|A10077 CVYL|A10134 CVYL|A10922 CVYL|A11879 CVYL|A12747 CVYL|A13009 FGRM|XP_011318250.1 FGRM|XP_011322283.1 MGRA|XP_003851067.1 MGRA|XP_003857066.1 MLAR|XP_007406338.1 MLAR|XP_007410400.1 MLAR|XP_007410420.1 MLAR|XP_007413777.1 MLAR|XP_007417085.1 MORY|QBZ53678.1 MORY|QBZ54113.1 MORY|QBZ58130.1 MORY|QBZ58153.1 MORY|QBZ59343.1 MORY|QBZ59914.1 NCRA|XP_011394793.1 SSCL|APA10583.1

>Orthogroup77: ANID|CBF71491.1 ANID|CBF82958.1 ANID|CBF86026.1 BCIN|XP_001548818.1 BCIN|XP_024547074.1 BCIN|XP_024548115.1 BCIN|XP_024548131.1 CFRU|XP_031875651.1 CFRU|XP_031875863.1 CFRU|XP_031876192.1 CFRU|XP_031878639.1 CFRU|XP_031878989.1 CFRU|XP_031878991.1 CFRU|XP_031883722.1 CFRU|XP_031884351.1 CFRU|XP_031887224.1 CFRU|XP_031888920.1 CFRU|XP_031893213.1 CGLO|KAF3799944.1 CGLO|KAF3802625.1 CGLO|KAF3804294.1 CGLO|KAF3807865.1 CHIG|XP_018150662.1 CHIG|XP_018150701.1 CHIG|XP_018158395.1 CVIN|KAF4903087.1 CVIN|KAF4905507.1 CVIN|KAF4917479.1 CVYL|A00747 CVYL|A01921 CVYL|A02266 CVYL|A03817 CVYL|A14189 FGRM|XP_011315826.1 FGRM|XP_011317908.1 FGRM|XP_011318495.1 FGRM|XP_011319604.1 FGRM|XP_011319621.1 FGRM|XP_011320669.1 FGRM|XP_011323324.1 FGRM|XP_011325292.1 MORY|QBZ63952.1 MORY|QBZ66320.1 SSCL|APA07343.1 SSCL|APA08683.1 SSCL|APA10359.1 SSCL|APA10375.1 SSCL|APA11275.1 SSCL|APA11428.1 SSCL|APA11429.1 SSCL|APA11430.1 SSCL|APA11507.1 SSCL|APA11939.1 SSCL|APA11969.1 SSCL|APA13409.1 SSCL|APA13412.1 SSCL|APA14649.1 SSCL|APA15267.1 SSCL|APA15333.1 SSCL|APA16011.1

>Orthogroup78: ANID|CBF75874.1 ANID|CBF86071.1 BCIN|XP_024549325.1 BCIN|XP_024549432.1 BCIN|XP_024549940.1 BCIN|XP_024553962.1 BGRA|VDB96332.1 CFRU|XP_031878881.1 CFRU|XP_031879496.1 CFRU|XP_031887261.1 CFRU|XP_031890555.1 CFRU|XP_031891744.1 CFRU|XP_031892263.1 CGLO|KAF3798133.1 CGLO|KAF3800165.1 CGLO|KAF3801260.1 CGLO|KAF3802628.1 CGLO|KAF3809271.1 CHIG|XP_018154736.1 CHIG|XP_018156107.1 CHIG|XP_018158638.1 CHIG|XP_018160907.1 CHIG|XP_018161296.1 CHIG|XP_018165103.1 CVIN|KAF4913910.1 CVIN|KAF4921066.1 CVIN|KAF4921634.1 CVIN|KAF4921927.1 CVIN|KAF4925797.1 CVIN|KAF4925980.1 CVYL|A01464 CVYL|A01923 CVYL|A06862 CVYL|A07829 CVYL|A13581 CVYL|A13973 FGRM|XP_011316352.1 FGRM|XP_011317741.1 FGRM|XP_011318838.1 FGRM|XP_011323401.1 FGRM|XP_011324379.1 MGRA|XP_003848640.1 MGRA|XP_003849143.1 MGRA|XP_003850613.1 MGRA|XP_003853697.1 MLAR|XP_007410920.1 MLAR|XP_007413219.1 MLAR|XP_007415099.1 MLAR|XP_007415222.1 MORY|QBZ56083.1 MORY|QBZ58187.1 MORY|QBZ63017.1 NCRA|XP_958975.2 NCRA|XP_960096.2 NCRA|XP_961671.1 NCRA|XP_964183.3 SSCL|APA05278.1 SSCL|APA09239.1 SSCL|APA15427.1 SSCL|APA15806.1

>Orthogroup79: ANID|CBF76494.1 ANID|CBF82056.1 ANID|CBF84243.1 ANID|CBF84276.1 ANID|CBF87299.1 BCIN|XP_001548739.1 BCIN|XP_001553435.2 BCIN|XP_001560679.1 BCIN|XP_024553120.1 CFRU|XP_031875691.1 CFRU|XP_031876573.1 CFRU|XP_031877727.1 CFRU|XP_031885160.1 CFRU|XP_031892248.1 CFRU|XP_031892268.1 CFRU|XP_031892336.1 CFRU|XP_031892883.1 CGLO|KAF3797150.1 CGLO|KAF3797317.1 CGLO|KAF3798116.1 CGLO|KAF3800892.1 CGLO|KAF3801257.1 CGLO|KAF3801371.1 CGLO|KAF3807676.1 CHIG|XP_018150791.1 CHIG|XP_018153307.1 CHIG|XP_018157094.1 CHIG|XP_018157191.1 CHIG|XP_018162897.1 CHIG|XP_018164861.1 CVIN|KAF4911870.1 CVIN|KAF4917655.1 CVIN|KAF4921923.1 CVIN|KAF4927823.1 CVIN|KAF4927995.1 CVYL|A06853 CVYL|A07710 CVYL|A07833 CVYL|A08243 CVYL|A10378 CVYL|A11132 FGRM|XP_011317995.1 FGRM|XP_011320180.1 FGRM|XP_011322739.1 FGRM|XP_011323191.1 FGRM|XP_011323796.1 FGRM|XP_011325456.1 FGRM|XP_011326284.1 MGRA|XP_003851997.1 MGRA|XP_003852467.1 MGRA|XP_003852918.1 MGRA|XP_003853049.1 MGRA|XP_003855183.1 MGRA|XP_003857060.1 MORY|QBZ61459.1 NCRA|XP_956057.1 NCRA|XP_960842.1 SSCL|APA10838.1 SSCL|APA10908.1 SSCL|APA16347.1

>Orthogroup80: MLAR|XP_007403673.1 MLAR|XP_007403758.1 MLAR|XP_007403795.1 MLAR|XP_007403979.1 MLAR|XP_007403995.1 MLAR|XP_007404082.1 MLAR|XP_007404230.1 MLAR|XP_007404388.1 MLAR|XP_007404406.1 MLAR|XP_007404407.1 MLAR|XP_007404408.1 MLAR|XP_007404409.1 MLAR|XP_007404526.1 MLAR|XP_007404603.1 MLAR|XP_007405135.1 MLAR|XP_007405265.1 MLAR|XP_007405266.1 MLAR|XP_007405608.1 MLAR|XP_007405609.1 MLAR|XP_007405622.1 MLAR|XP_007405927.1 MLAR|XP_007406364.1 MLAR|XP_007406370.1 MLAR|XP_007407214.1 MLAR|XP_007408290.1 MLAR|XP_007408684.1 MLAR|XP_007408759.1 MLAR|XP_007408772.1 MLAR|XP_007408800.1 MLAR|XP_007409042.1 MLAR|XP_007409219.1 MLAR|XP_007409817.1 MLAR|XP_007410017.1 MLAR|XP_007410018.1 MLAR|XP_007410019.1 MLAR|XP_007410438.1 MLAR|XP_007410477.1 MLAR|XP_007410520.1 MLAR|XP_007410675.1 MLAR|XP_007411304.1 MLAR|XP_007411336.1 MLAR|XP_007411636.1 MLAR|XP_007411957.1 MLAR|XP_007412820.1 MLAR|XP_007413828.1 MLAR|XP_007414697.1 MLAR|XP_007415030.1 MLAR|XP_007415147.1 MLAR|XP_007415758.1 MLAR|XP_007415782.1 MLAR|XP_007417473.1 MLAR|XP_007417852.1 MLAR|XP_007417853.1 MLAR|XP_007417888.1 MLAR|XP_007418075.1 MLAR|XP_007418087.1 MLAR|XP_007419082.1 MLAR|XP_007419085.1 MLAR|XP_007419086.1 MLAR|XP_007419087.1

>Orthogroup81: BGRA|VCU39097.1 BGRA|VCU39098.1 BGRA|VCU39100.1 BGRA|VCU39113.1 BGRA|VCU39114.1 BGRA|VCU39116.1 BGRA|VCU39117.1 BGRA|VCU39118.1 BGRA|VCU39128.1 BGRA|VCU39129.1 BGRA|VCU39136.1 BGRA|VCU39138.1 BGRA|VCU39139.1 BGRA|VCU39140.1 BGRA|VCU39141.1 BGRA|VCU39142.1 BGRA|VCU39143.1 BGRA|VCU39144.1 BGRA|VCU39145.1 BGRA|VCU39150.1 BGRA|VCU39608.1 BGRA|VCU39609.1 BGRA|VCU40431.1 BGRA|VCU41191.1 BGRA|VCU41194.1 BGRA|VCU41195.1 BGRA|VCU41196.1 BGRA|VCU41198.1 BGRA|VCU41199.1 BGRA|VCU41200.1 BGRA|VCU41201.1 BGRA|VCU41207.1 BGRA|VCU41209.1 BGRA|VCU41210.1 BGRA|VCU41213.1 BGRA|VCU41221.1 BGRA|VCU41367.1 BGRA|VCU41368.1 BGRA|VCU41389.1 BGRA|VCU41390.1 BGRA|VCU41396.1 BGRA|VDB83954.1 BGRA|VDB84162.1 BGRA|VDB85708.1 BGRA|VDB85730.1 BGRA|VDB85746.1 BGRA|VDB85787.1 BGRA|VDB85788.1 BGRA|VDB85790.1 BGRA|VDB85791.1 BGRA|VDB85807.1 BGRA|VDB85808.1 BGRA|VDB85813.1 BGRA|VDB85815.1 BGRA|VDB86010.1 BGRA|VDB86011.1 BGRA|VDB87910.1 BGRA|VDB87912.1 BGRA|VDB96381.1

>Orthogroup82: MLAR|XP_007404545.1 MLAR|XP_007404834.1 MLAR|XP_007405139.1 MLAR|XP_007405245.1 MLAR|XP_007405719.1 MLAR|XP_007405850.1 MLAR|XP_007406225.1 MLAR|XP_007406961.1 MLAR|XP_007407205.1 MLAR|XP_007407613.1 MLAR|XP_007408037.1 MLAR|XP_007408204.1 MLAR|XP_007408424.1 MLAR|XP_007408849.1 MLAR|XP_007408927.1 MLAR|XP_007409827.1 MLAR|XP_007409954.1 MLAR|XP_007410317.1 MLAR|XP_007410508.1 MLAR|XP_007410638.1 MLAR|XP_007410761.1 MLAR|XP_007411320.1 MLAR|XP_007411321.1 MLAR|XP_007412353.1 MLAR|XP_007412916.1 MLAR|XP_007413269.1 MLAR|XP_007413479.1 MLAR|XP_007413496.1 MLAR|XP_007413544.1 MLAR|XP_007413718.1 MLAR|XP_007413865.1 MLAR|XP_007413930.1 MLAR|XP_007414039.1 MLAR|XP_007414614.1 MLAR|XP_007414615.1 MLAR|XP_007414752.1 MLAR|XP_007414810.1 MLAR|XP_007414948.1 MLAR|XP_007415102.1 MLAR|XP_007415332.1 MLAR|XP_007416301.1 MLAR|XP_007416337.1 MLAR|XP_007416465.1 MLAR|XP_007416730.1 MLAR|XP_007416810.1 MLAR|XP_007417106.1 MLAR|XP_007417295.1 MLAR|XP_007417398.1 MLAR|XP_007417717.1 MLAR|XP_007417786.1 MLAR|XP_007418228.1 MLAR|XP_007418791.1 MLAR|XP_007419122.1 MLAR|XP_007419132.1 MLAR|XP_007419184.1 MLAR|XP_007419304.1 MLAR|XP_007419369.1 MLAR|XP_007419638.1

>Orthogroup83: BCIN|XP_001552814.1 BCIN|XP_001555115.1 BCIN|XP_001555963.1 BCIN|XP_001556379.1 BCIN|XP_001556804.1 BCIN|XP_024547424.1 CFRU|XP_031875685.1 CFRU|XP_031877361.1 CFRU|XP_031877364.1 CFRU|XP_031878178.1 CFRU|XP_031879509.1 CFRU|XP_031880996.1 CFRU|XP_031884092.1 CFRU|XP_031891876.1 CFRU|XP_031892080.1 CGLO|KAF3799916.1 CGLO|KAF3799920.1 CGLO|KAF3799965.1 CGLO|KAF3801303.1 CGLO|KAF3801403.1 CGLO|KAF3802389.1 CGLO|KAF3802567.1 CGLO|KAF3807824.1 CGLO|KAF3808480.1 CHIG|XP_018152522.1 CHIG|XP_018154687.1 CHIG|XP_018157643.1 CHIG|XP_018159060.1 CHIG|XP_018159088.1 CHIG|XP_018159646.1 CHIG|XP_018161400.1 CHIG|XP_018161981.1 CVIN|KAF4903972.1 CVIN|KAF4906958.1 CVIN|KAF4917606.1 CVIN|KAF4917729.1 CVIN|KAF4919357.1 CVIN|KAF4919359.1 CVIN|KAF4921502.1 CVIN|KAF4921601.1 CVIN|KAF4922179.1 CVIN|KAF4922359.1 CVIN|KAF4926887.1 CVYL|A01856 CVYL|A07679 CVYL|A07783 CVYL|A09603 CVYL|A09606 CVYL|A10181 CVYL|A11659 CVYL|A13532 FGRM|XP_011321867.1 FGRM|XP_011322553.1 MGRA|XP_003856047.1 MORY|QBZ54341.1 MORY|QBZ62895.1 SSCL|APA08968.1

>Orthogroup84: MLAR|XP_007403811.1 MLAR|XP_007404641.1 MLAR|XP_007405080.1 MLAR|XP_007405207.1 MLAR|XP_007405354.1 MLAR|XP_007405554.1 MLAR|XP_007405556.1 MLAR|XP_007405566.1 MLAR|XP_007405790.1 MLAR|XP_007406198.1 MLAR|XP_007407110.1 MLAR|XP_007407185.1 MLAR|XP_007407404.1 MLAR|XP_007407583.1 MLAR|XP_007407619.1 MLAR|XP_007407687.1 MLAR|XP_007408166.1 MLAR|XP_007408318.1 MLAR|XP_007408721.1 MLAR|XP_007408966.1 MLAR|XP_007409356.1 MLAR|XP_007409375.1 MLAR|XP_007409453.1 MLAR|XP_007409741.1 MLAR|XP_007410290.1 MLAR|XP_007410454.1 MLAR|XP_007410911.1 MLAR|XP_007410914.1 MLAR|XP_007411193.1 MLAR|XP_007411356.1 MLAR|XP_007411834.1 MLAR|XP_007411835.1 MLAR|XP_007411891.1 MLAR|XP_007411941.1 MLAR|XP_007413597.1 MLAR|XP_007413803.1 MLAR|XP_007413859.1 MLAR|XP_007414267.1 MLAR|XP_007414570.1 MLAR|XP_007414621.1 MLAR|XP_007415506.1 MLAR|XP_007415754.1 MLAR|XP_007415864.1 MLAR|XP_007416083.1 MLAR|XP_007416771.1 MLAR|XP_007416988.1 MLAR|XP_007417011.1 MLAR|XP_007417013.1 MLAR|XP_007417163.1 MLAR|XP_007417218.1 MLAR|XP_007417251.1 MLAR|XP_007417305.1 MLAR|XP_007417416.1 MLAR|XP_007417577.1 MLAR|XP_007419126.1 MLAR|XP_007419533.1 MLAR|XP_007419646.1

>Orthogroup85: BCIN|XP_024546082.1 CFRU|XP_031875939.1 CFRU|XP_031879331.1 CFRU|XP_031881868.1 CFRU|XP_031881869.1 CFRU|XP_031881871.1 CFRU|XP_031881874.1 CFRU|XP_031881875.1 CFRU|XP_031884005.1 CFRU|XP_031884045.1 CFRU|XP_031884046.1 CFRU|XP_031884050.1 CFRU|XP_031884768.1 CFRU|XP_031889123.1 CGLO|KAF3803110.1 CGLO|KAF3804981.1 CGLO|KAF3804983.1 CGLO|KAF3805708.1 CGLO|KAF3808654.1 CGLO|KAF3810011.1 CGLO|KAF3812109.1 CHIG|XP_018155604.1 CHIG|XP_018157683.1 CHIG|XP_018157684.1 CHIG|XP_018163534.1 CHIG|XP_018163616.1 CVIN|KAF4907631.1 CVIN|KAF4913001.1 CVIN|KAF4913002.1 CVIN|KAF4913015.1 CVIN|KAF4913016.1 CVIN|KAF4918115.1 CVIN|KAF4927102.1 CVIN|KAF4927103.1 CVIN|KAF4927105.1 CVIN|KAF4927106.1 CVIN|KAF4927107.1 CVIN|KAF4929558.1 CVYL|A00287 CVYL|A02610 CVYL|A02611 CVYL|A06567 CVYL|A06568 CVYL|A06569 CVYL|A06572 CVYL|A09802 CVYL|A11866 FGRM|XP_011316914.1 MGRA|XP_003854774.1 MGRA|XP_003854775.1 MORY|QBZ56863.1 MORY|QBZ60683.1 MORY|QBZ61131.1 NCRA|XP_011394344.1 NCRA|XP_957513.2 NCRA|XP_957514.2

>Orthogroup86: MLAR|XP_007403860.1 MLAR|XP_007404575.1 MLAR|XP_007404811.1 MLAR|XP_007405483.1 MLAR|XP_007405636.1 MLAR|XP_007406425.1 MLAR|XP_007407285.1 MLAR|XP_007407359.1 MLAR|XP_007407410.1 MLAR|XP_007407712.1 MLAR|XP_007407952.1 MLAR|XP_007408089.1 MLAR|XP_007408353.1 MLAR|XP_007409168.1 MLAR|XP_007409189.1 MLAR|XP_007409190.1 MLAR|XP_007409319.1 MLAR|XP_007410646.1 MLAR|XP_007411176.1 MLAR|XP_007411178.1 MLAR|XP_007411792.1 MLAR|XP_007412425.1 MLAR|XP_007412450.1 MLAR|XP_007412707.1 MLAR|XP_007412801.1 MLAR|XP_007412972.1 MLAR|XP_007413678.1 MLAR|XP_007414148.1 MLAR|XP_007414173.1 MLAR|XP_007414314.1 MLAR|XP_007414701.1 MLAR|XP_007415095.1 MLAR|XP_007415414.1 MLAR|XP_007415950.1 MLAR|XP_007416054.1 MLAR|XP_007416066.1 MLAR|XP_007416071.1 MLAR|XP_007416319.1 MLAR|XP_007416703.1 MLAR|XP_007417176.1 MLAR|XP_007417361.1 MLAR|XP_007417391.1 MLAR|XP_007417771.1 MLAR|XP_007417936.1 MLAR|XP_007417947.1 MLAR|XP_007418470.1 MLAR|XP_007418535.1 MLAR|XP_007418738.1 MLAR|XP_007418838.1 MLAR|XP_007419037.1 MLAR|XP_007419091.1 MLAR|XP_007419313.1 MLAR|XP_007419392.1 MLAR|XP_007419449.1 MLAR|XP_007419464.1 MLAR|XP_007419509.1

>Orthogroup87: ANID|CBF73518.1 ANID|CBF80789.1 ANID|CBF86428.1 ANID|CBF86988.1 BCIN|XP_001546186.1 BCIN|XP_001553433.1 BCIN|XP_024548025.1 BGRA|VDB86400.1 CFRU|XP_031878821.1 CFRU|XP_031878837.1 CFRU|XP_031883315.1 CFRU|XP_031884599.1 CFRU|XP_031886119.1 CFRU|XP_031886144.1 CFRU|XP_031886338.1 CFRU|XP_031887917.1 CFRU|XP_031892133.1 CFRU|XP_031892383.1 CGLO|KAF3797492.1 CGLO|KAF3799044.1 CGLO|KAF3801766.1 CGLO|KAF3809139.1 CGLO|KAF3811980.1 CHIG|XP_018153850.1 CHIG|XP_018155956.1 CHIG|XP_018158210.1 CHIG|XP_018159262.1 CVIN|KAF4918280.1 CVIN|KAF4921628.1 CVIN|KAF4921656.1 CVIN|KAF4922107.1 CVIN|KAF4922928.1 CVIN|KAF4923249.1 CVIN|KAF4930329.1 CVYL|A01323 CVYL|A01940 CVYL|A01942 CVYL|A05528 CVYL|A11486 CVYL|A11999 CVYL|A12941 FGRM|XP_011319177.1 FGRM|XP_011328641.1 MGRA|XP_003847668.1 MGRA|XP_003853120.1 MGRA|XP_003854094.1 MGRA|XP_003855445.1 MORY|QBZ61653.1 MORY|QBZ62882.1 MORY|QBZ62912.1 MORY|QBZ64239.1 NCRA|XP_011394946.1 NCRA|XP_959184.1 SSCL|APA10910.1 SSCL|APA11935.1

>Orthogroup88: ANID|CBF78900.1 ANID|CBF79711.1 BCIN|XP_001547017.1 BCIN|XP_024547951.1 BCIN|XP_024550347.1 BCIN|XP_024552095.1 BCIN|XP_024552422.1 BGRA|VDB85743.1 CFRU|XP_031883639.1 CFRU|XP_031883667.1 CFRU|XP_031883701.1 CFRU|XP_031884324.1 CFRU|XP_031884608.1 CFRU|XP_031886659.1 CFRU|XP_031891308.1 CGLO|KAF3796958.1 CGLO|KAF3797385.1 CGLO|KAF3800000.1 CGLO|KAF3800197.1 CGLO|KAF3800201.1 CGLO|KAF3800237.1 CGLO|KAF3801196.1 CHIG|XP_018153049.1 CHIG|XP_018155054.1 CHIG|XP_018155108.1 CHIG|XP_018155119.1 CHIG|XP_018157126.1 CHIG|XP_018158039.1 CHIG|XP_018162502.1 CVIN|KAF4903968.1 CVIN|KAF4920430.1 CVIN|KAF4920435.1 CVIN|KAF4921776.1 CVIN|KAF4926919.1 CVIN|KAF4929387.1 CVIN|KAF4932099.1 CVYL|A05823 CVYL|A06086 CVYL|A06090 CVYL|A07138 CVYL|A10216 CVYL|A10299 CVYL|A12411 FGRM|XP_011320072.1 FGRM|XP_011326958.1 MGRA|XP_003848345.1 MLAR|XP_007407816.1 MORY|QBZ55682.1 MORY|QBZ59317.1 NCRA|XP_001728488.2 NCRA|XP_964577.1 SSCL|APA05651.1 SSCL|APA08401.1 SSCL|APA11028.1 SSCL|APA12885.1

>Orthogroup89: ANID|CBF71803.1 ANID|CBF75103.1 ANID|CBF83495.1 ANID|CBF88498.1 BCIN|XP_001558268.1 BCIN|XP_001561324.1 BCIN|XP_024549890.1 BGRA|VDB86125.1 BGRA|VDB90905.1 BGRA|VDB95028.1 CFRU|XP_031881604.1 CFRU|XP_031884943.1 CFRU|XP_031888387.1 CFRU|XP_031891040.1 CFRU|XP_031891782.1 CGLO|KAF3801027.1 CGLO|KAF3801393.1 CGLO|KAF3803632.1 CGLO|KAF3805390.1 CHIG|XP_018151391.1 CHIG|XP_018153321.1 CHIG|XP_018156720.1 CHIG|XP_018163827.1 CHIG|XP_018164777.1 CVIN|KAF4907814.1 CVIN|KAF4908679.1 CVIN|KAF4913939.1 CVIN|KAF4917768.1 CVIN|KAF4922697.1 CVYL|A00356 CVYL|A07691 CVYL|A11036 CVYL|A12510 CVYL|A14567 FGRM|XP_011315786.1 FGRM|XP_011322771.1 FGRM|XP_011324344.1 FGRM|XP_011326531.1 FGRM|XP_011328475.1 MGRA|XP_003848500.1 MGRA|XP_003851836.1 MGRA|XP_003856324.1 MLAR|XP_007409625.1 MORY|QBZ54687.1 MORY|QBZ62692.1 MORY|QBZ64343.1 MORY|QBZ66202.1 NCRA|XP_958032.1 NCRA|XP_958627.1 NCRA|XP_960762.1 NCRA|XP_965203.1 SSCL|APA05567.1 SSCL|APA06865.1 SSCL|APA13667.1

>Orthogroup90: ANID|CBF85532.1 BCIN|XP_001548454.2 BCIN|XP_001560611.1 BCIN|XP_024549496.1 BCIN|XP_024551042.1 CFRU|XP_031876384.1 CFRU|XP_031877508.1 CFRU|XP_031882594.1 CFRU|XP_031882806.1 CFRU|XP_031885770.1 CFRU|XP_031889679.1 CFRU|XP_031891861.1 CGLO|KAF3798415.1 CGLO|KAF3806638.1 CGLO|KAF3809810.1 CGLO|KAF3810061.1 CGLO|KAF3810614.1 CHIG|XP_018151078.1 CHIG|XP_018158955.1 CHIG|XP_018160105.1 CHIG|XP_018164946.1 CVIN|KAF4908270.1 CVIN|KAF4912754.1 CVIN|KAF4918919.1 CVIN|KAF4919322.1 CVIN|KAF4920024.1 CVIN|KAF4925454.1 CVYL|A02658 CVYL|A03006 CVYL|A06729 CVYL|A08943 CVYL|A11220 CVYL|A12351 CVYL|A13696 FGRM|XP_011318013.1 FGRM|XP_011322847.1 MGRA|XP_003847726.1 MGRA|XP_003850170.1 MGRA|XP_003852884.1 MGRA|XP_003856522.1 MLAR|XP_007407802.1 MLAR|XP_007407805.1 MLAR|XP_007411200.1 MLAR|XP_007414824.1 MLAR|XP_007414825.1 MORY|QBZ53593.1 MORY|QBZ54876.1 MORY|QBZ60634.1 MORY|QBZ64001.1 MORY|QBZ64137.1 MORY|QBZ64991.1 MORY|QBZ65072.1 MORY|QBZ66573.1 NCRA|XP_958973.1

>Orthogroup91: ANID|CBF73457.1 ANID|CBF76583.1 ANID|CBF81882.1 BCIN|XP_024549556.1 BCIN|XP_024550614.1 BGRA|VCU40204.1 BGRA|VDB88025.1 CFRU|XP_031875320.1 CFRU|XP_031875645.1 CFRU|XP_031875725.1 CFRU|XP_031876990.1 CFRU|XP_031877000.1 CFRU|XP_031877307.1 CFRU|XP_031881858.1 CFRU|XP_031882715.1 CFRU|XP_031883520.1 CGLO|KAF3799872.1 CGLO|KAF3803399.1 CGLO|KAF3809993.1 CGLO|KAF3810842.1 CHIG|XP_018150623.1 CHIG|XP_018153821.1 CHIG|XP_018154978.1 CHIG|XP_018157736.1 CHIG|XP_018163933.1 CVIN|KAF4905025.1 CVIN|KAF4911112.1 CVIN|KAF4918411.1 CVIN|KAF4921725.1 CVYL|A02298 CVYL|A02593 CVYL|A09646 CVYL|A14329 FGRM|XP_011316878.1 FGRM|XP_011316879.1 FGRM|XP_011322114.1 FGRM|XP_011325436.1 FGRM|XP_011325681.1 MGRA|XP_003848284.1 MGRA|XP_003848353.1 MGRA|XP_003850551.1 MGRA|XP_003851941.1 MLAR|XP_007404738.1 MORY|QBZ55077.1 MORY|QBZ56002.1 MORY|QBZ59972.1 MORY|QBZ63297.1 MORY|QBZ66748.1 NCRA|XP_955945.2 NCRA|XP_957924.2 NCRA|XP_965309.3 SSCL|APA11431.1 SSCL|APA13916.1

>Orthogroup92: ANID|CBF74829.1 ANID|CBF78470.1 ANID|CBF78847.1 ANID|CBF87282.1 ANID|CBF87284.1 BCIN|XP_001555592.1 BCIN|XP_001560161.1 CFRU|XP_031875917.1 CFRU|XP_031878167.1 CFRU|XP_031880831.1 CFRU|XP_031881907.1 CFRU|XP_031882651.1 CFRU|XP_031884098.1 CFRU|XP_031884633.1 CFRU|XP_031885108.1 CFRU|XP_031885110.1 CGLO|KAF3799738.1 CGLO|KAF3801824.1 CGLO|KAF3803882.1 CGLO|KAF3806920.1 CGLO|KAF3807751.1 CGLO|KAF3807753.1 CGLO|KAF3810006.1 CGLO|KAF3811073.1 CHIG|XP_018150802.1 CHIG|XP_018151651.1 CHIG|XP_018152941.1 CHIG|XP_018153100.1 CHIG|XP_018155390.1 CHIG|XP_018162649.1 CHIG|XP_018163021.1 CVIN|KAF4913006.1 CVIN|KAF4915267.1 CVIN|KAF4919157.1 CVIN|KAF4920504.1 CVIN|KAF4920505.1 CVIN|KAF4921510.1 CVIN|KAF4925458.1 CVYL|A00795 CVYL|A00845 CVYL|A00847 CVYL|A02563 CVYL|A08918 CVYL|A09725 CVYL|A11354 CVYL|A12883 FGRM|XP_011323064.1 FGRM|XP_011325853.1 FGRM|XP_011325880.1 MGRA|XP_003848450.1 MGRA|XP_003851933.1 MORY|QBZ66444.1 SSCL|APA15859.1

>Orthogroup93: ANID|CBF76291.1 ANID|CBF77164.1 ANID|CBF80671.1 ANID|CBF85310.1 BCIN|XP_001547344.1 BCIN|XP_024546307.1 BCIN|XP_024552871.1 CFRU|XP_031877918.1 CFRU|XP_031880698.1 CFRU|XP_031883415.1 CFRU|XP_031883650.1 CFRU|XP_031884266.1 CFRU|XP_031887778.1 CFRU|XP_031891120.1 CGLO|KAF3798584.1 CGLO|KAF3801744.1 CGLO|KAF3803799.1 CGLO|KAF3807149.1 CGLO|KAF3810946.1 CGLO|KAF3811774.1 CGLO|KAF3811924.1 CHIG|XP_018150768.1 CHIG|XP_018154900.1 CHIG|XP_018156694.1 CHIG|XP_018160980.1 CHIG|XP_018161549.1 CVIN|KAF4895624.1 CVIN|KAF4907650.1 CVIN|KAF4914194.1 CVIN|KAF4917920.1 CVIN|KAF4920188.1 CVIN|KAF4922933.1 CVIN|KAF4928565.1 CVIN|KAF4930079.1 CVYL|A01656 CVYL|A02202 CVYL|A03550 CVYL|A04860 CVYL|A06140 CVYL|A12779 CVYL|A14170 FGRM|XP_011316501.1 FGRM|XP_011324421.1 MGRA|XP_003852525.1 MGRA|XP_003854062.1 MGRA|XP_003856054.1 MLAR|XP_007404110.1 MORY|QBZ53932.1 MORY|QBZ58204.1 MORY|QBZ61947.1 NCRA|XP_961684.1 SSCL|APA09112.1 SSCL|APA16116.1

>Orthogroup94: BGRA|VCU39163.1 BGRA|VCU39175.1 BGRA|VCU39212.1 BGRA|VCU39519.1 BGRA|VCU39527.1 BGRA|VCU39653.1 BGRA|VCU39701.1 BGRA|VCU39718.1 BGRA|VCU39747.1 BGRA|VCU39791.1 BGRA|VCU40242.1 BGRA|VCU40299.1 BGRA|VCU40497.1 BGRA|VCU40654.1 BGRA|VCU40841.1 BGRA|VCU41177.1 BGRA|VCU41264.1 BGRA|VCU41274.1 BGRA|VCU41322.1 BGRA|VCU41326.1 BGRA|VDB83491.1 BGRA|VDB83531.1 BGRA|VDB84141.1 BGRA|VDB85684.1 BGRA|VDB85686.1 BGRA|VDB85958.1 BGRA|VDB86325.1 BGRA|VDB86339.1 BGRA|VDB87570.1 BGRA|VDB87936.1 BGRA|VDB88117.1 BGRA|VDB88120.1 BGRA|VDB88222.1 BGRA|VDB88260.1 BGRA|VDB88989.1 BGRA|VDB88994.1 BGRA|VDB89057.1 BGRA|VDB89680.1 BGRA|VDB89722.1 BGRA|VDB89860.1 BGRA|VDB89865.1 BGRA|VDB90369.1 BGRA|VDB90724.1 BGRA|VDB92439.1 BGRA|VDB92990.1 BGRA|VDB93149.1 BGRA|VDB94459.1 BGRA|VDB94610.1 BGRA|VDB94824.1 BGRA|VDB94826.1 BGRA|VDB94830.1 BGRA|VDB94923.1 BGRA|VDB95182.1

>Orthogroup95: MLAR|XP_007404158.1 MLAR|XP_007404310.1 MLAR|XP_007404456.1 MLAR|XP_007404712.1 MLAR|XP_007404767.1 MLAR|XP_007404768.1 MLAR|XP_007404769.1 MLAR|XP_007404770.1 MLAR|XP_007404771.1 MLAR|XP_007404948.1 MLAR|XP_007405623.1 MLAR|XP_007405982.1 MLAR|XP_007406058.1 MLAR|XP_007406588.1 MLAR|XP_007407120.1 MLAR|XP_007407121.1 MLAR|XP_007407136.1 MLAR|XP_007407326.1 MLAR|XP_007407328.1 MLAR|XP_007407333.1 MLAR|XP_007407760.1 MLAR|XP_007407922.1 MLAR|XP_007408267.1 MLAR|XP_007409315.1 MLAR|XP_007409917.1 MLAR|XP_007410061.1 MLAR|XP_007410263.1 MLAR|XP_007411125.1 MLAR|XP_007411190.1 MLAR|XP_007411488.1 MLAR|XP_007411705.1 MLAR|XP_007412456.1 MLAR|XP_007412686.1 MLAR|XP_007412763.1 MLAR|XP_007413014.1 MLAR|XP_007413360.1 MLAR|XP_007413510.1 MLAR|XP_007413973.1 MLAR|XP_007414130.1 MLAR|XP_007414167.1 MLAR|XP_007414583.1 MLAR|XP_007414878.1 MLAR|XP_007415073.1 MLAR|XP_007415161.1 MLAR|XP_007415473.1 MLAR|XP_007416717.1 MLAR|XP_007416718.1 MLAR|XP_007416719.1 MLAR|XP_007417022.1 MLAR|XP_007418351.1 MLAR|XP_007418577.1 MLAR|XP_007418588.1 MLAR|XP_007419224.1

>Orthogroup96: MLAR|XP_007404252.1 MLAR|XP_007404637.1 MLAR|XP_007404638.1 MLAR|XP_007404750.1 MLAR|XP_007405687.1 MLAR|XP_007405895.1 MLAR|XP_007406719.1 MLAR|XP_007406851.1 MLAR|XP_007407138.1 MLAR|XP_007407401.1 MLAR|XP_007407869.1 MLAR|XP_007408567.1 MLAR|XP_007408682.1 MLAR|XP_007408906.1 MLAR|XP_007409129.1 MLAR|XP_007409288.1 MLAR|XP_007409303.1 MLAR|XP_007409361.1 MLAR|XP_007409409.1 MLAR|XP_007409566.1 MLAR|XP_007410082.1 MLAR|XP_007411107.1 MLAR|XP_007411108.1 MLAR|XP_007411114.1 MLAR|XP_007411516.1 MLAR|XP_007412131.1 MLAR|XP_007412517.1 MLAR|XP_007412649.1 MLAR|XP_007413698.1 MLAR|XP_007413964.1 MLAR|XP_007414232.1 MLAR|XP_007414256.1 MLAR|XP_007414294.1 MLAR|XP_007414382.1 MLAR|XP_007414405.1 MLAR|XP_007414738.1 MLAR|XP_007414954.1 MLAR|XP_007415728.1 MLAR|XP_007416147.1 MLAR|XP_007416739.1 MLAR|XP_007417177.1 MLAR|XP_007417417.1 MLAR|XP_007417995.1 MLAR|XP_007418193.1 MLAR|XP_007418660.1 MLAR|XP_007418665.1 MLAR|XP_007418736.1 MLAR|XP_007418761.1 MLAR|XP_007419008.1 MLAR|XP_007419303.1 MLAR|XP_007419494.1 MLAR|XP_007419798.1 MLAR|XP_007419808.1

>Orthogroup97: ANID|CBF71205.1 ANID|CBF71846.1 ANID|CBF74966.1 ANID|CBF76141.1 ANID|CBF80232.1 ANID|CBF89272.1 ANID|CBF89325.1 ANID|CBF89338.1 BCIN|XP_001552723.1 CFRU|XP_031875794.1 CFRU|XP_031875978.1 CFRU|XP_031875998.1 CFRU|XP_031878211.1 CFRU|XP_031883146.1 CFRU|XP_031883946.1 CFRU|XP_031890993.1 CFRU|XP_031893612.1 CGLO|KAF3800816.1 CGLO|KAF3801513.1 CGLO|KAF3801514.1 CGLO|KAF3803256.1 CGLO|KAF3803805.1 CGLO|KAF3805016.1 CGLO|KAF3808488.1 CHIG|XP_018150856.1 CHIG|XP_018151625.1 CHIG|XP_018152826.1 CHIG|XP_018158990.1 CHIG|XP_018160296.1 CVIN|KAF4919633.1 CVIN|KAF4922469.1 CVIN|KAF4927089.1 CVYL|A00430 CVYL|A05486 CVYL|A08733 CVYL|A09107 CVYL|A09998 FGRM|XP_011318155.1 FGRM|XP_011319561.1 FGRM|XP_011325238.1 FGRM|XP_011326027.1 FGRM|XP_011328702.1 MGRA|XP_003849964.1 MORY|QBZ57958.1 MORY|QBZ58148.1 MORY|QBZ65056.1 NCRA|XP_011394221.1 NCRA|XP_961797.3 NCRA|XP_961932.2 NCRA|XP_965033.1 SSCL|APA10644.1 SSCL|APA14063.1

>Orthogroup98: ANID|CBF71547.1 ANID|CBF81976.1 ANID|CBF82396.1 BCIN|XP_001547254.1 BCIN|XP_001550254.1 BCIN|XP_001551072.1 BCIN|XP_001553186.1 BCIN|XP_024546494.1 BCIN|XP_024552410.1 BGRA|VDB93655.1 CFRU|XP_031880255.1 CFRU|XP_031881015.1 CFRU|XP_031884481.1 CFRU|XP_031887179.1 CFRU|XP_031888194.1 CGLO|KAF3799744.1 CGLO|KAF3802086.1 CGLO|KAF3809031.1 CGLO|KAF3809401.1 CGLO|KAF3809850.1 CHIG|XP_018154236.1 CHIG|XP_018155864.1 CHIG|XP_018156244.1 CHIG|XP_018161431.1 CVIN|KAF4916409.1 CVIN|KAF4917963.1 CVIN|KAF4918972.1 CVIN|KAF4923767.1 CVIN|KAF4930702.1 CVYL|A01607 CVYL|A01764 CVYL|A03041 CVYL|A03301 FGRM|XP_011319608.1 FGRM|XP_011328061.1 MGRA|XP_003852363.1 MGRA|XP_003854016.1 MORY|QBZ56961.1 MORY|QBZ58298.1 MORY|QBZ58341.1 MORY|QBZ63059.1 MORY|QBZ65521.1 NCRA|XP_956939.1 NCRA|XP_957099.1 NCRA|XP_958498.2 NCRA|XP_958970.1 NCRA|XP_959595.3 NCRA|XP_960566.3 NCRA|XP_962436.2 SSCL|APA07387.1 SSCL|APA07533.1 SSCL|APA14269.1

>Orthogroup99: ANID|CBF74058.1 ANID|CBF83089.1 ANID|CBF83094.1 ANID|CBF85188.1 BCIN|XP_024546269.1 BCIN|XP_024550375.1 BCIN|XP_024550377.1 CFRU|XP_031877374.1 CFRU|XP_031879163.1 CFRU|XP_031879277.1 CFRU|XP_031884073.1 CFRU|XP_031885764.1 CFRU|XP_031887508.1 CFRU|XP_031893522.1 CGLO|KAF3799917.1 CGLO|KAF3805005.1 CGLO|KAF3807008.1 CGLO|KAF3807184.1 CGLO|KAF3808348.1 CGLO|KAF3808603.1 CGLO|KAF3810072.1 CHIG|XP_018151537.1 CHIG|XP_018151661.1 CHIG|XP_018152405.1 CHIG|XP_018157780.1 CHIG|XP_018159361.1 CHIG|XP_018159859.1 CHIG|XP_018161557.1 CHIG|XP_018162665.1 CHIG|XP_018163511.1 CVIN|KAF4895117.1 CVIN|KAF4912740.1 CVIN|KAF4918635.1 CVIN|KAF4920237.1 CVIN|KAF4926204.1 CVIN|KAF4927138.1 CVYL|A02668 CVYL|A03590 CVYL|A06543 CVYL|A09507 CVYL|A12379 FGRM|XP_011317717.1 FGRM|XP_011323091.1 MORY|QBZ53334.1 MORY|QBZ53644.1 MORY|QBZ54470.1 MORY|QBZ56181.1 MORY|QBZ60171.1 MORY|QBZ63117.1 NCRA|XP_011394932.1 NCRA|XP_958493.1 SSCL|APA15883.1

>Orthogroup100: CFRU|XP_031875503.1 CFRU|XP_031875894.1 CFRU|XP_031876845.1 CFRU|XP_031877837.1 CFRU|XP_031881014.1 CFRU|XP_031882331.1 CFRU|XP_031885060.1 CFRU|XP_031887534.1 CFRU|XP_031892903.1 CGLO|KAF3797975.1 CGLO|KAF3802659.1 CGLO|KAF3803303.1 CGLO|KAF3804366.1 CGLO|KAF3806620.1 CGLO|KAF3806815.1 CGLO|KAF3807769.1 CGLO|KAF3808658.1 CGLO|KAF3808688.1 CGLO|KAF3809047.1 CGLO|KAF3809599.1 CGLO|KAF3809849.1 CHIG|XP_018151551.1 CHIG|XP_018151614.1 CHIG|XP_018151615.1 CHIG|XP_018153829.1 CHIG|XP_018153920.1 CHIG|XP_018160153.1 CHIG|XP_018160180.1 CHIG|XP_018161149.1 CHIG|XP_018161866.1 CVIN|KAF4895166.1 CVIN|KAF4918357.1 CVIN|KAF4918929.1 CVIN|KAF4920523.1 CVIN|KAF4926116.1 CVIN|KAF4926117.1 CVIN|KAF4928665.1 CVIN|KAF4930703.1 CVYL|A01765 CVYL|A03737 CVYL|A04050 CVYL|A11844 CVYL|A13656 FGRM|XP_011319614.1 FGRM|XP_011319635.1 FGRM|XP_011321817.1 FGRM|XP_011322167.1 MLAR|XP_007404944.1 MORY|QBZ56128.1 MORY|QBZ58125.1 MORY|QBZ64966.1 NCRA|XP_958167.1

>Orthogroup101: MLAR|XP_007403645.1 MLAR|XP_007403815.1 MLAR|XP_007405323.1 MLAR|XP_007405479.1 MLAR|XP_007405699.1 MLAR|XP_007406349.1 MLAR|XP_007407101.1 MLAR|XP_007407176.1 MLAR|XP_007407676.1 MLAR|XP_007407953.1 MLAR|XP_007408101.1 MLAR|XP_007408238.1 MLAR|XP_007408564.1 MLAR|XP_007408620.1 MLAR|XP_007409306.1 MLAR|XP_007409439.1 MLAR|XP_007409513.1 MLAR|XP_007410741.1 MLAR|XP_007410742.1 MLAR|XP_007411081.1 MLAR|XP_007411084.1 MLAR|XP_007412718.1 MLAR|XP_007412785.1 MLAR|XP_007412811.1 MLAR|XP_007414168.1 MLAR|XP_007414184.1 MLAR|XP_007414253.1 MLAR|XP_007414276.1 MLAR|XP_007414563.1 MLAR|XP_007414703.1 MLAR|XP_007414983.1 MLAR|XP_007415085.1 MLAR|XP_007415403.1 MLAR|XP_007415856.1 MLAR|XP_007416113.1 MLAR|XP_007416133.1 MLAR|XP_007416624.1 MLAR|XP_007417362.1 MLAR|XP_007417772.1 MLAR|XP_007417915.1 MLAR|XP_007417946.1 MLAR|XP_007418093.1 MLAR|XP_007418356.1 MLAR|XP_007418544.1 MLAR|XP_007418730.1 MLAR|XP_007418847.1 MLAR|XP_007419054.1 MLAR|XP_007419203.1 MLAR|XP_007419395.1 MLAR|XP_007419461.1 MLAR|XP_007419512.1 MLAR|XP_007419581.1

>Orthogroup102: MLAR|XP_007403654.1 MLAR|XP_007403786.1 MLAR|XP_007404317.1 MLAR|XP_007404390.1 MLAR|XP_007404742.1 MLAR|XP_007404766.1 MLAR|XP_007405364.1 MLAR|XP_007405399.1 MLAR|XP_007405494.1 MLAR|XP_007406004.1 MLAR|XP_007406206.1 MLAR|XP_007406602.1 MLAR|XP_007406898.1 MLAR|XP_007406899.1 MLAR|XP_007406918.1 MLAR|XP_007407045.1 MLAR|XP_007407207.1 MLAR|XP_007407451.1 MLAR|XP_007407675.1 MLAR|XP_007407991.1 MLAR|XP_007408033.1 MLAR|XP_007408815.1 MLAR|XP_007408970.1 MLAR|XP_007409502.1 MLAR|XP_007409632.1 MLAR|XP_007410460.1 MLAR|XP_007410804.1 MLAR|XP_007410869.1 MLAR|XP_007411284.1 MLAR|XP_007411688.1 MLAR|XP_007411849.1 MLAR|XP_007413812.1 MLAR|XP_007415326.1 MLAR|XP_007415624.1 MLAR|XP_007415689.1 MLAR|XP_007415706.1 MLAR|XP_007415737.1 MLAR|XP_007415974.1 MLAR|XP_007416683.1 MLAR|XP_007416715.1 MLAR|XP_007417318.1 MLAR|XP_007417581.1 MLAR|XP_007418065.1 MLAR|XP_007418352.1 MLAR|XP_007418374.1 MLAR|XP_007418386.1 MLAR|XP_007418542.1 MLAR|XP_007419173.1 MLAR|XP_007419505.1 MLAR|XP_007419618.1 MLAR|XP_007419782.1 MLAR|XP_007419841.1

>Orthogroup103: MLAR|XP_007403681.1 MLAR|XP_007403748.1 MLAR|XP_007403833.1 MLAR|XP_007404879.1 MLAR|XP_007405753.1 MLAR|XP_007405931.1 MLAR|XP_007406413.1 MLAR|XP_007407251.1 MLAR|XP_007407707.1 MLAR|XP_007407709.1 MLAR|XP_007407876.1 MLAR|XP_007408176.1 MLAR|XP_007408293.1 MLAR|XP_007408523.1 MLAR|XP_007408680.1 MLAR|XP_007408890.1 MLAR|XP_007408942.1 MLAR|XP_007410080.1 MLAR|XP_007410081.1 MLAR|XP_007410624.1 MLAR|XP_007410625.1 MLAR|XP_007411317.1 MLAR|XP_007411346.1 MLAR|XP_007411837.1 MLAR|XP_007412151.1 MLAR|XP_007412581.1 MLAR|XP_007412796.1 MLAR|XP_007412982.1 MLAR|XP_007413038.1 MLAR|XP_007413905.1 MLAR|XP_007414102.1 MLAR|XP_007414995.1 MLAR|XP_007415119.1 MLAR|XP_007415943.1 MLAR|XP_007416435.1 MLAR|XP_007417096.1 MLAR|XP_007417114.1 MLAR|XP_007417191.1 MLAR|XP_007417544.1 MLAR|XP_007417840.1 MLAR|XP_007418114.1 MLAR|XP_007418340.1 MLAR|XP_007418341.1 MLAR|XP_007418349.1 MLAR|XP_007418363.1 MLAR|XP_007418795.1 MLAR|XP_007418860.1 MLAR|XP_007419366.1 MLAR|XP_007419557.1 MLAR|XP_007419576.1 MLAR|XP_007419717.1 MLAR|XP_007419786.1

>Orthogroup104: MLAR|XP_007404268.1 MLAR|XP_007404272.1 MLAR|XP_007404476.1 MLAR|XP_007404564.1 MLAR|XP_007405214.1 MLAR|XP_007405571.1 MLAR|XP_007405795.1 MLAR|XP_007405949.1 MLAR|XP_007406320.1 MLAR|XP_007406471.1 MLAR|XP_007406548.1 MLAR|XP_007407055.1 MLAR|XP_007407057.1 MLAR|XP_007407221.1 MLAR|XP_007407324.1 MLAR|XP_007407365.1 MLAR|XP_007407479.1 MLAR|XP_007407560.1 MLAR|XP_007408896.1 MLAR|XP_007409273.1 MLAR|XP_007409959.1 MLAR|XP_007410093.1 MLAR|XP_007410541.1 MLAR|XP_007410655.1 MLAR|XP_007411697.1 MLAR|XP_007412014.1 MLAR|XP_007412081.1 MLAR|XP_007412180.1 MLAR|XP_007412265.1 MLAR|XP_007412571.1 MLAR|XP_007414044.1 MLAR|XP_007415712.1 MLAR|XP_007415841.1 MLAR|XP_007415846.1 MLAR|XP_007416178.1 MLAR|XP_007416249.1 MLAR|XP_007416334.1 MLAR|XP_007416721.1 MLAR|XP_007417198.1 MLAR|XP_007417213.1 MLAR|XP_007417634.1 MLAR|XP_007418279.1 MLAR|XP_007418487.1 MLAR|XP_007418825.1 MLAR|XP_007419182.1 MLAR|XP_007419218.1 MLAR|XP_007419346.1 MLAR|XP_007419386.1 MLAR|XP_007419400.1 MLAR|XP_007419441.1 MLAR|XP_007419565.1 MLAR|XP_007419725.1

>Orthogroup105: MLAR|XP_007404477.1 MLAR|XP_007404814.1 MLAR|XP_007404823.1 MLAR|XP_007404828.1 MLAR|XP_007405144.1 MLAR|XP_007405201.1 MLAR|XP_007405547.1 MLAR|XP_007405774.1 MLAR|XP_007405882.1 MLAR|XP_007406155.1 MLAR|XP_007406493.1 MLAR|XP_007406579.1 MLAR|XP_007406736.1 MLAR|XP_007406740.1 MLAR|XP_007407099.1 MLAR|XP_007407143.1 MLAR|XP_007407220.1 MLAR|XP_007407556.1 MLAR|XP_007409125.1 MLAR|XP_007409175.1 MLAR|XP_007409713.1 MLAR|XP_007410096.1 MLAR|XP_007410193.1 MLAR|XP_007410358.1 MLAR|XP_007410447.1 MLAR|XP_007410726.1 MLAR|XP_007410748.1 MLAR|XP_007412340.1 MLAR|XP_007412608.1 MLAR|XP_007412880.1 MLAR|XP_007413350.1 MLAR|XP_007414075.1 MLAR|XP_007415622.1 MLAR|XP_007415732.1 MLAR|XP_007416130.1 MLAR|XP_007416131.1 MLAR|XP_007416706.1 MLAR|XP_007416760.1 MLAR|XP_007416761.1 MLAR|XP_007417283.1 MLAR|XP_007417635.1 MLAR|XP_007417865.1 MLAR|XP_007418263.1 MLAR|XP_007418303.1 MLAR|XP_007418326.1 MLAR|XP_007418499.1 MLAR|XP_007418562.1 MLAR|XP_007419220.1 MLAR|XP_007419399.1 MLAR|XP_007419444.1 MLAR|XP_007419445.1 MLAR|XP_007419567.1

>Orthogroup106: MLAR|XP_007404960.1 MLAR|XP_007405062.1 MLAR|XP_007405509.1 MLAR|XP_007407184.1 MLAR|XP_007407968.1 MLAR|XP_007408257.1 MLAR|XP_007408263.1 MLAR|XP_007408364.1 MLAR|XP_007408465.1 MLAR|XP_007408466.1 MLAR|XP_007408713.1 MLAR|XP_007409031.1 MLAR|XP_007409887.1 MLAR|XP_007410003.1 MLAR|XP_007410004.1 MLAR|XP_007410279.1 MLAR|XP_007410280.1 MLAR|XP_007411168.1 MLAR|XP_007411901.1 MLAR|XP_007412122.1 MLAR|XP_007412777.1 MLAR|XP_007413738.1 MLAR|XP_007413739.1 MLAR|XP_007414121.1 MLAR|XP_007414122.1 MLAR|XP_007414213.1 MLAR|XP_007414214.1 MLAR|XP_007414251.1 MLAR|XP_007415194.1 MLAR|XP_007415612.1 MLAR|XP_007415613.1 MLAR|XP_007416482.1 MLAR|XP_007416623.1 MLAR|XP_007416681.1 MLAR|XP_007417300.1 MLAR|XP_007417302.1 MLAR|XP_007417384.1 MLAR|XP_007418104.1 MLAR|XP_007418206.1 MLAR|XP_007418411.1 MLAR|XP_007418581.1 MLAR|XP_007418833.1 MLAR|XP_007418845.1 MLAR|XP_007418890.1 MLAR|XP_007418891.1 MLAR|XP_007418986.1 MLAR|XP_007419196.1 MLAR|XP_007419212.1 MLAR|XP_007419213.1 MLAR|XP_007419436.1 MLAR|XP_007419590.1 MLAR|XP_007419600.1

>Orthogroup107: ANID|CBF71823.1 ANID|CBF76561.1 ANID|CBF86622.1 ANID|CBF87116.1 ANID|CBF88117.1 BCIN|XP_001547212.2 BCIN|XP_001548857.1 BCIN|XP_001549476.1 BCIN|XP_001549864.1 BCIN|XP_001553967.1 CFRU|XP_031877235.1 CFRU|XP_031878098.1 CFRU|XP_031880214.1 CFRU|XP_031880958.1 CFRU|XP_031885910.1 CFRU|XP_031885955.1 CFRU|XP_031888820.1 CFRU|XP_031890894.1 CGLO|KAF3799228.1 CGLO|KAF3801846.1 CGLO|KAF3805517.1 CGLO|KAF3806837.1 CGLO|KAF3808940.1 CGLO|KAF3811987.1 CHIG|XP_018155255.1 CHIG|XP_018155382.1 CHIG|XP_018159334.1 CHIG|XP_018159606.1 CHIG|XP_018161109.1 CHIG|XP_018161473.1 CVIN|KAF4901587.1 CVIN|KAF4909292.1 CVIN|KAF4918312.1 CVIN|KAF4930428.1 CVIN|KAF4930724.1 CVYL|A01731 CVYL|A02484 CVYL|A05520 CVYL|A05640 CVYL|A12859 FGRM|XP_011317430.1 FGRM|XP_011322230.1 FGRM|XP_011322648.1 FGRM|XP_011325131.1 MLAR|XP_007411862.1 MLAR|XP_007411863.1 MLAR|XP_007412538.1 MLAR|XP_007416654.1 SSCL|APA08244.1 SSCL|APA13447.1 SSCL|APA15980.1

>Orthogroup108: ANID|CBF73428.1 ANID|CBF78859.1 ANID|CBF84390.1 ANID|CBF87297.1 BCIN|XP_024545967.1 BCIN|XP_024546481.1 BCIN|XP_024547117.1 BCIN|XP_024547262.1 BCIN|XP_024548150.1 BCIN|XP_024548433.1 BCIN|XP_024549233.1 BCIN|XP_024549692.1 BCIN|XP_024550508.1 BCIN|XP_024551391.1 BCIN|XP_024551750.1 BCIN|XP_024552963.1 BCIN|XP_024553328.1 BGRA|VDB93287.1 CFRU|XP_031881722.1 CFRU|XP_031886959.1 CFRU|XP_031889309.1 CFRU|XP_031892663.1 CGLO|KAF3797289.1 CGLO|KAF3797847.1 CGLO|KAF3808909.1 CGLO|KAF3811328.1 CHIG|XP_018152696.1 CHIG|XP_018160554.1 CVIN|KAF4908869.1 CVIN|KAF4920734.1 CVIN|KAF4927247.1 CVYL|A01177 CVYL|A04511 CVYL|A11529 FGRM|XP_011325445.1 FGRM|XP_011325625.1 FGRM|XP_011326899.1 FGRM|XP_011327735.1 MGRA|XP_003856617.1 MORY|QBZ56622.1 MORY|QBZ57017.1 MORY|QBZ63284.1 NCRA|XP_961504.3 SSCL|APA05624.1 SSCL|APA07411.1 SSCL|APA10623.1 SSCL|APA11266.1 SSCL|APA14016.1 SSCL|APA14218.1 SSCL|APA14219.1 SSCL|APA16248.1

>Orthogroup109: ANID|CBF78938.1 BCIN|XP_001547129.1 BCIN|XP_001549298.1 BCIN|XP_001555183.1 BCIN|XP_001555528.1 BCIN|XP_024545947.1 BCIN|XP_024547003.1 BGRA|VDB92812.1 BGRA|VDB93135.1 CFRU|XP_031882613.1 CFRU|XP_031882959.1 CFRU|XP_031883549.1 CFRU|XP_031884955.1 CGLO|KAF3803867.1 CGLO|KAF3808307.1 CGLO|KAF3810870.1 CHIG|XP_018151061.1 CHIG|XP_018151587.1 CHIG|XP_018153870.1 CHIG|XP_018154968.1 CVIN|KAF4913276.1 CVIN|KAF4916347.1 CVIN|KAF4924077.1 CVIN|KAF4925450.1 CVYL|A00643 CVYL|A02274 CVYL|A05931 CVYL|A08933 FGRM|XP_011316524.1 FGRM|XP_011322016.1 FGRM|XP_011325579.1 MGRA|XP_003847578.1 MGRA|XP_003848252.1 MGRA|XP_003854919.1 MGRA|XP_003856923.1 MLAR|XP_007408333.1 MLAR|XP_007408595.1 MLAR|XP_007408596.1 MLAR|XP_007408597.1 MLAR|XP_007411542.1 MLAR|XP_007414535.1 MLAR|XP_007416912.1 MORY|QBZ57853.1 MORY|QBZ64551.1 MORY|QBZ64854.1 NCRA|XP_965183.2 SSCL|APA05877.1 SSCL|APA07378.1 SSCL|APA09175.1 SSCL|APA10749.1 SSCL|APA12109.1

>Orthogroup110: ANID|CBF79793.1 ANID|CBF82339.1 BCIN|XP_024551665.1 BCIN|XP_024553807.1 BGRA|VCU38949.1 CFRU|XP_031876968.1 CFRU|XP_031876970.1 CFRU|XP_031876980.1 CFRU|XP_031877010.1 CFRU|XP_031877313.1 CFRU|XP_031877371.1 CGLO|KAF3799924.1 CHIG|XP_018153779.1 CHIG|XP_018156498.1 CVIN|KAF4903978.1 CVIN|KAF4903979.1 CVIN|KAF4906961.1 CVYL|A09613 FGRM|XP_011315645.1 FGRM|XP_011325979.1 FGRM|XP_011327776.1 FGRM|XP_011327777.1 MGRA|XP_003847416.1 MGRA|XP_003851300.1 MGRA|XP_003853553.1 MORY|QBZ53311.1 MORY|QBZ53312.1 MORY|QBZ53313.1 MORY|QBZ53314.1 MORY|QBZ53493.1 MORY|QBZ53494.1 MORY|QBZ53495.1 MORY|QBZ53496.1 MORY|QBZ53497.1 MORY|QBZ53498.1 MORY|QBZ53499.1 MORY|QBZ53500.1 MORY|QBZ53501.1 MORY|QBZ53510.1 MORY|QBZ53515.1 MORY|QBZ53601.1 MORY|QBZ55013.1 MORY|QBZ56801.1 MORY|QBZ58562.1 MORY|QBZ58563.1 MORY|QBZ66632.1 MORY|QBZ66648.1 MORY|QBZ66698.1 NCRA|XP_962556.1 SSCL|APA14802.1 SSCL|APA15260.1

>Orthogroup111: ANID|CBF83064.1 BCIN|XP_001559635.1 BCIN|XP_024549103.1 CFRU|XP_031876480.1 CFRU|XP_031880299.1 CFRU|XP_031882571.1 CFRU|XP_031884514.1 CFRU|XP_031884671.1 CFRU|XP_031893275.1 CGLO|KAF3799678.1 CGLO|KAF3799717.1 CGLO|KAF3808927.1 CGLO|KAF3809542.1 CHIG|XP_018158064.1 CHIG|XP_018158260.1 CHIG|XP_018158270.1 CHIG|XP_018163089.1 CHIG|XP_018163090.1 CVIN|KAF4905643.1 CVIN|KAF4918568.1 CVIN|KAF4919945.1 CVIN|KAF4919954.1 CVIN|KAF4920720.1 CVIN|KAF4925474.1 CVYL|A01159 CVYL|A05842 CVYL|A05877 CVYL|A08349 CVYL|A08480 CVYL|A08899 FGRM|XP_011321151.1 FGRM|XP_011325372.1 FGRM|XP_011326167.1 MLAR|XP_007405853.1 MLAR|XP_007413051.1 MLAR|XP_007413053.1 MLAR|XP_007413057.1 MLAR|XP_007413058.1 MLAR|XP_007413059.1 MLAR|XP_007413127.1 MLAR|XP_007413134.1 MLAR|XP_007419538.1 MLAR|XP_007419539.1 MLAR|XP_007419540.1 MLAR|XP_007419812.1 MORY|QBZ54840.1 MORY|QBZ57793.1 MORY|QBZ61916.1 NCRA|XP_958513.3 NCRA|XP_959289.2 SSCL|APA09402.1

>Orthogroup112: BCIN|XP_001549558.2 BCIN|XP_024550620.1 BCIN|XP_024552582.1 BCIN|XP_024553288.1 CFRU|XP_031878843.1 CFRU|XP_031880095.1 CFRU|XP_031880257.1 CFRU|XP_031881980.1 CFRU|XP_031881983.1 CFRU|XP_031882964.1 CFRU|XP_031888620.1 CGLO|KAF3798776.1 CGLO|KAF3798823.1 CGLO|KAF3799341.1 CGLO|KAF3799342.1 CGLO|KAF3799343.1 CGLO|KAF3802634.1 CGLO|KAF3806058.1 CGLO|KAF3808211.1 CGLO|KAF3809037.1 CHIG|XP_018161298.1 CHIG|XP_018162977.1 CVIN|KAF4912778.1 CVIN|KAF4913555.1 CVIN|KAF4921661.1 CVIN|KAF4923770.1 CVIN|KAF4924290.1 CVIN|KAF4924771.1 CVYL|A00559 CVYL|A02342 CVYL|A03925 CVYL|A04397 CVYL|A04408 CVYL|A07456 CVYL|A09585 CVYL|A12798 CVYL|A12799 CVYL|A13616 FGRM|XP_011318326.1 FGRM|XP_011320956.1 FGRM|XP_011321663.1 FGRM|XP_011323363.1 FGRM|XP_011325097.1 FGRM|XP_011328737.1 MGRA|XP_003849600.1 MGRA|XP_003852729.1 NCRA|XP_011393626.1 NCRA|XP_962827.3 SSCL|APA15386.1 SSCL|APA15643.1 SSCL|APA15644.1

>Orthogroup113: MLAR|XP_007404155.1 MLAR|XP_007404228.1 MLAR|XP_007404463.1 MLAR|XP_007405533.1 MLAR|XP_007405575.1 MLAR|XP_007405614.1 MLAR|XP_007405749.1 MLAR|XP_007405802.1 MLAR|XP_007406186.1 MLAR|XP_007406202.1 MLAR|XP_007407000.1 MLAR|XP_007407189.1 MLAR|XP_007407933.1 MLAR|XP_007408078.1 MLAR|XP_007408553.1 MLAR|XP_007409180.1 MLAR|XP_007409751.1 MLAR|XP_007410048.1 MLAR|XP_007410181.1 MLAR|XP_007410640.1 MLAR|XP_007410642.1 MLAR|XP_007410658.1 MLAR|XP_007411228.1 MLAR|XP_007411921.1 MLAR|XP_007411967.1 MLAR|XP_007412440.1 MLAR|XP_007413695.1 MLAR|XP_007413787.1 MLAR|XP_007414041.1 MLAR|XP_007414627.1 MLAR|XP_007414739.1 MLAR|XP_007414970.1 MLAR|XP_007415726.1 MLAR|XP_007416003.1 MLAR|XP_007416373.1 MLAR|XP_007416381.1 MLAR|XP_007416620.1 MLAR|XP_007416922.1 MLAR|XP_007417171.1 MLAR|XP_007417376.1 MLAR|XP_007417709.1 MLAR|XP_007417779.1 MLAR|XP_007418103.1 MLAR|XP_007418294.1 MLAR|XP_007418495.1 MLAR|XP_007418759.1 MLAR|XP_007419153.1 MLAR|XP_007419205.1 MLAR|XP_007419320.1 MLAR|XP_007419404.1 MLAR|XP_007419484.1

>Orthogroup114: MLAR|XP_007403761.1 MLAR|XP_007403977.1 MLAR|XP_007404123.1 MLAR|XP_007404131.1 MLAR|XP_007404465.1 MLAR|XP_007404577.1 MLAR|XP_007404588.1 MLAR|XP_007405079.1 MLAR|XP_007405125.1 MLAR|XP_007406145.1 MLAR|XP_007406536.1 MLAR|XP_007406538.1 MLAR|XP_007406802.1 MLAR|XP_007407118.1 MLAR|XP_007407561.1 MLAR|XP_007407765.1 MLAR|XP_007407947.1 MLAR|XP_007407949.1 MLAR|XP_007408000.1 MLAR|XP_007408481.1 MLAR|XP_007408609.1 MLAR|XP_007408844.1 MLAR|XP_007410005.1 MLAR|XP_007410050.1 MLAR|XP_007410247.1 MLAR|XP_007410643.1 MLAR|XP_007411811.1 MLAR|XP_007411939.1 MLAR|XP_007412720.1 MLAR|XP_007413897.1 MLAR|XP_007414456.1 MLAR|XP_007414709.1 MLAR|XP_007415029.1 MLAR|XP_007415120.1 MLAR|XP_007415314.1 MLAR|XP_007415549.1 MLAR|XP_007416723.1 MLAR|XP_007416998.1 MLAR|XP_007417493.1 MLAR|XP_007417583.1 MLAR|XP_007417832.1 MLAR|XP_007417948.1 MLAR|XP_007418097.1 MLAR|XP_007418106.1 MLAR|XP_007418108.1 MLAR|XP_007418453.1 MLAR|XP_007418667.1 MLAR|XP_007418871.1 MLAR|XP_007418879.1 MLAR|XP_007419128.1 MLAR|XP_007419174.1

>Orthogroup115: ANID|CBF69557.1 ANID|CBF71560.1 ANID|CBF80434.1 ANID|CBF82747.1 BCIN|XP_001558118.2 BCIN|XP_024553690.1 BGRA|VDB96341.1 CFRU|XP_031878387.1 CFRU|XP_031879049.1 CFRU|XP_031882232.1 CFRU|XP_031883870.1 CFRU|XP_031889285.1 CFRU|XP_031890113.1 CGLO|KAF3798832.1 CGLO|KAF3801284.1 CGLO|KAF3802348.1 CGLO|KAF3804363.1 CGLO|KAF3805047.1 CGLO|KAF3808901.1 CGLO|KAF3810753.1 CHIG|XP_018151225.1 CHIG|XP_018152804.1 CHIG|XP_018161585.1 CHIG|XP_018162550.1 CVIN|KAF4889312.1 CVIN|KAF4905800.1 CVIN|KAF4914511.1 CVIN|KAF4918335.1 CVIN|KAF4919604.1 CVIN|KAF4921587.1 CVIN|KAF4932021.1 CVYL|A04052 CVYL|A05180 CVYL|A06509 CVYL|A07202 CVYL|A09102 FGRM|XP_011317593.1 FGRM|XP_011318330.1 FGRM|XP_011327651.1 MGRA|XP_003847419.1 MGRA|XP_003851960.1 MGRA|XP_003854549.1 MLAR|XP_007406598.1 MORY|QBZ53426.1 MORY|QBZ59718.1 MORY|QBZ59719.1 NCRA|XP_956651.1 NCRA|XP_958502.1 SSCL|APA12966.1 SSCL|APA15089.1

>Orthogroup116: ANID|CBF70633.1 ANID|CBF71591.1 ANID|CBF76678.1 ANID|CBF80144.1 ANID|CBF85228.1 ANID|CBF85230.1 ANID|CBF87258.1 BCIN|XP_001549344.1 BCIN|XP_001552245.1 CFRU|XP_031877369.1 CFRU|XP_031878919.1 CFRU|XP_031880947.1 CFRU|XP_031883533.1 CFRU|XP_031884945.1 CFRU|XP_031887512.1 CGLO|KAF3806855.1 CGLO|KAF3807874.1 CGLO|KAF3807875.1 CGLO|KAF3809110.1 CGLO|KAF3810852.1 CGLO|KAF3811602.1 CGLO|KAF3812193.1 CHIG|XP_018153416.1 CHIG|XP_018153826.1 CHIG|XP_018159542.1 CHIG|XP_018160874.1 CVIN|KAF4893128.1 CVIN|KAF4911105.1 CVIN|KAF4920979.1 CVIN|KAF4921476.1 CVIN|KAF4921705.1 CVIN|KAF4922578.1 CVYL|A00192 CVYL|A00737 CVYL|A00738 CVYL|A02290 CVYL|A02502 CVYL|A03460 CVYL|A09651 FGRM|XP_011315643.1 FGRM|XP_011324862.1 MGRA|XP_003848913.1 MGRA|XP_003849223.1 MGRA|XP_003851317.1 MLAR|XP_007410174.1 MLAR|XP_007417217.1 MORY|QBZ56553.1 NCRA|XP_961973.2 SSCL|APA06405.1 SSCL|APA12902.1

>Orthogroup117: ANID|CBF73421.1 ANID|CBF76627.1 ANID|CBF76692.1 ANID|CBF88800.1 BCIN|XP_001560201.2 BCIN|XP_024549732.1 BCIN|XP_024551175.1 BCIN|XP_024551627.1 BGRA|VCU41059.1 CFRU|XP_031880278.1 CFRU|XP_031881557.1 CFRU|XP_031882710.1 CFRU|XP_031883201.1 CGLO|KAF3804809.1 CGLO|KAF3806640.1 CGLO|KAF3807445.1 CGLO|KAF3809089.1 CHIG|XP_018151543.1 CHIG|XP_018155892.1 CHIG|XP_018156785.1 CHIG|XP_018163253.1 CVIN|KAF4918529.1 CVIN|KAF4918931.1 CVIN|KAF4918934.1 CVIN|KAF4923805.1 CVIN|KAF4924304.1 CVYL|A00504 CVYL|A01623 CVYL|A10953 CVYL|A12353 FGRM|XP_011325364.1 FGRM|XP_011326235.1 FGRM|XP_011328287.1 MGRA|XP_003854441.1 MORY|QBZ55169.1 MORY|QBZ61798.1 MORY|QBZ62602.1 MORY|QBZ62738.1 MORY|QBZ63087.1 NCRA|XP_957091.2 NCRA|XP_959036.3 NCRA|XP_959395.2 NCRA|XP_960189.1 NCRA|XP_960409.2 NCRA|XP_965228.1 SSCL|APA05433.1 SSCL|APA06271.1 SSCL|APA10182.1 SSCL|APA15899.1

>Orthogroup118: ANID|CBF85212.1 ANID|CBF89390.1 BCIN|XP_024548637.1 BCIN|XP_024549082.1 BCIN|XP_024550081.1 BCIN|XP_024550804.1 BCIN|XP_024553571.1 CFRU|XP_031877163.1 CFRU|XP_031883980.1 CFRU|XP_031885890.1 CFRU|XP_031890576.1 CFRU|XP_031892251.1 CGLO|KAF3803992.1 CGLO|KAF3805008.1 CGLO|KAF3805449.1 CGLO|KAF3806730.1 CGLO|KAF3809690.1 CHIG|XP_018150944.1 CHIG|XP_018151070.1 CHIG|XP_018155305.1 CHIG|XP_018158708.1 CHIG|XP_018163513.1 CHIG|XP_018164901.1 CVIN|KAF4919491.1 CVIN|KAF4921953.1 CVIN|KAF4925213.1 CVIN|KAF4927136.1 CVIN|KAF4930394.1 CVYL|A05579 CVYL|A06541 CVYL|A06607 CVYL|A08808 CVYL|A14041 FGRM|XP_011317340.1 FGRM|XP_011317679.1 FGRM|XP_011323042.1 FGRM|XP_011323066.1 FGRM|XP_011323122.1 FGRM|XP_011323321.1 FGRM|XP_011325155.1 FGRM|XP_011328870.1 MGRA|XP_003854493.1 MORY|QBZ55835.1 MORY|QBZ60461.1 NCRA|XP_011392810.1 NCRA|XP_963752.1 SSCL|APA09867.1 SSCL|APA11503.1 SSCL|APA15868.1

>Orthogroup119: ANID|CBF69705.1 ANID|CBF69936.1 ANID|CBF88558.1 ANID|CBF90396.1 BCIN|XP_001560008.1 BCIN|XP_024546758.1 BCIN|XP_024547345.1 BCIN|XP_024547370.1 BCIN|XP_024547964.1 BCIN|XP_024549286.1 BCIN|XP_024551250.1 CFRU|XP_031875845.1 CFRU|XP_031881956.1 CFRU|XP_031884100.1 CFRU|XP_031890585.1 CFRU|XP_031890688.1 CGLO|KAF3805546.1 CGLO|KAF3806159.1 CGLO|KAF3806713.1 CGLO|KAF3806746.1 CGLO|KAF3811069.1 CHIG|XP_018153095.1 CHIG|XP_018155398.1 CHIG|XP_018158689.1 CHIG|XP_018161098.1 CHIG|XP_018164502.1 CVIN|KAF4890970.1 CVIN|KAF4891766.1 CVIN|KAF4915261.1 CVIN|KAF4919474.1 CVIN|KAF4919488.1 CVYL|A05669 CVYL|A07348 CVYL|A09728 CVYL|A14020 CVYL|A14058 FGRM|XP_011315867.1 FGRM|XP_011323192.1 FGRM|XP_011325044.1 FGRM|XP_011325121.1 MORY|QBZ59010.1 NCRA|XP_959706.3 NCRA|XP_963379.3 SSCL|APA08380.1 SSCL|APA08908.1 SSCL|APA09980.1 SSCL|APA14324.1 SSCL|APA14373.1

>Orthogroup120: ANID|CBF73386.1 ANID|CBF81948.1 ANID|CBF87736.1 BCIN|XP_001546858.1 BCIN|XP_001555779.2 BCIN|XP_024548804.1 BCIN|XP_024551018.1 BCIN|XP_024553355.1 BCIN|XP_024553760.1 BGRA|VDB85983.1 BGRA|VDB86329.1 CFRU|XP_031884272.1 CFRU|XP_031891342.1 CFRU|XP_031892901.1 CGLO|KAF3797419.1 CGLO|KAF3799956.1 CGLO|KAF3804587.1 CGLO|KAF3806817.1 CGLO|KAF3809601.1 CHIG|XP_018151526.1 CHIG|XP_018151647.1 CHIG|XP_018153108.1 CHIG|XP_018156533.1 CHIG|XP_018157042.1 CHIG|XP_018157379.1 CHIG|XP_018159071.1 CVIN|KAF4929350.1 CVYL|A05292 CVYL|A10170 CVYL|A10258 FGRM|XP_011318235.1 FGRM|XP_011319986.1 FGRM|XP_011328596.1 MGRA|XP_003855956.1 MORY|QBZ54348.1 MORY|QBZ62888.1 MORY|QBZ63119.1 MORY|QBZ66578.1 NCRA|XP_011394320.1 NCRA|XP_957867.2 NCRA|XP_962939.1 SSCL|APA07270.1 SSCL|APA07271.1 SSCL|APA10036.1 SSCL|APA10270.1 SSCL|APA11169.1 SSCL|APA16356.1 SSCL|APA16357.1

>Orthogroup121: ANID|CBF74692.1 ANID|CBF74694.1 ANID|CBF83471.1 ANID|CBF83667.1 ANID|CBF86363.1 BCIN|XP_024546205.1 CFRU|XP_031878846.1 CFRU|XP_031879494.1 CFRU|XP_031882696.1 CFRU|XP_031883296.1 CFRU|XP_031884612.1 CFRU|XP_031886056.1 CFRU|XP_031893058.1 CGLO|KAF3799079.1 CGLO|KAF3801169.1 CGLO|KAF3801751.1 CGLO|KAF3802436.1 CGLO|KAF3802623.1 CGLO|KAF3803869.1 CGLO|KAF3808390.1 CHIG|XP_018151086.1 CHIG|XP_018152457.1 CHIG|XP_018155034.1 CHIG|XP_018155205.1 CHIG|XP_018158180.1 CHIG|XP_018159275.1 CVIN|KAF4890318.1 CVIN|KAF4905504.1 CVIN|KAF4911052.1 CVIN|KAF4916860.1 CVIN|KAF4918266.1 CVIN|KAF4922936.1 CVIN|KAF4925428.1 CVYL|A01919 CVYL|A08930 CVYL|A09549 CVYL|A11450 CVYL|A12954 CVYL|A13579 FGRM|XP_011317487.1 FGRM|XP_011327525.1 MGRA|XP_003847855.1 MGRA|XP_003852478.1 MORY|QBZ65538.1 NCRA|XP_958530.1 NCRA|XP_958986.2 NCRA|XP_962843.2 SSCL|APA07020.1

>Orthogroup122: ANID|CBF74968.1 ANID|CBF86789.1 BCIN|XP_024545894.1 CFRU|XP_031878912.1 CFRU|XP_031879738.1 CFRU|XP_031879952.1 CFRU|XP_031881574.1 CFRU|XP_031883072.1 CFRU|XP_031883165.1 CFRU|XP_031890322.1 CFRU|XP_031891343.1 CFRU|XP_031891907.1 CFRU|XP_031892219.1 CGLO|KAF3798162.1 CGLO|KAF3798391.1 CGLO|KAF3798932.1 CGLO|KAF3805614.1 CGLO|KAF3807396.1 CGLO|KAF3808269.1 CGLO|KAF3808270.1 CGLO|KAF3808282.1 CGLO|KAF3809724.1 CHIG|XP_018151955.1 CHIG|XP_018152728.1 CHIG|XP_018152880.1 CHIG|XP_018163037.1 CHIG|XP_018164822.1 CHIG|XP_018165010.1 CVIN|KAF4898137.1 CVIN|KAF4921161.1 CVIN|KAF4921964.1 CVIN|KAF4922817.1 CVIN|KAF4924091.1 CVIN|KAF4929357.1 CVYL|A00619 CVYL|A04955 CVYL|A06646 CVYL|A07582 CVYL|A07852 CVYL|A08207 CVYL|A10259 CVYL|A12195 FGRM|XP_011322461.1 FGRM|XP_011323048.1 MGRA|XP_003855448.1 MORY|QBZ59441.1 MORY|QBZ65649.1 SSCL|APA09487.1

>Orthogroup123: ANID|CBF78393.1 BCIN|XP_001554946.1 CFRU|XP_031876097.1 CFRU|XP_031877657.1 CFRU|XP_031880158.1 CFRU|XP_031883075.1 CFRU|XP_031884326.1 CFRU|XP_031886145.1 CFRU|XP_031889308.1 CFRU|XP_031891690.1 CFRU|XP_031891729.1 CGLO|KAF3799998.1 CGLO|KAF3800087.1 CGLO|KAF3801107.1 CGLO|KAF3805650.1 CGLO|KAF3807358.1 CGLO|KAF3808267.1 CGLO|KAF3808888.1 CGLO|KAF3811823.1 CGLO|KAF3811978.1 CHIG|XP_018151742.1 CHIG|XP_018153051.1 CHIG|XP_018153759.1 CHIG|XP_018156186.1 CHIG|XP_018163320.1 CHIG|XP_018164679.1 CHIG|XP_018165008.1 CVIN|KAF4895289.1 CVIN|KAF4909551.1 CVIN|KAF4920053.1 CVIN|KAF4924891.1 CVIN|KAF4925686.1 CVIN|KAF4926914.1 CVIN|KAF4930331.1 CVYL|A01179 CVYL|A05530 CVYL|A07548 CVYL|A10214 CVYL|A10852 CVYL|A10853 CVYL|A11288 CVYL|A12827 FGRM|XP_011325122.1 FGRM|XP_011325735.1 MGRA|XP_003855000.1 NCRA|XP_957420.1 NCRA|XP_964953.1 SSCL|APA08370.1

>Orthogroup124: ANID|CBF80198.1 BCIN|XP_001547385.1 BCIN|XP_001560071.2 CFRU|XP_031878082.1 CFRU|XP_031878501.1 CFRU|XP_031881223.1 CFRU|XP_031881417.1 CFRU|XP_031885145.1 CFRU|XP_031889058.1 CFRU|XP_031889225.1 CFRU|XP_031890086.1 CGLO|KAF3799266.1 CGLO|KAF3799803.1 CGLO|KAF3801870.1 CGLO|KAF3805721.1 CGLO|KAF3807797.1 CGLO|KAF3808917.1 CGLO|KAF3810371.1 CGLO|KAF3810989.1 CHIG|XP_018151604.1 CHIG|XP_018155776.1 CHIG|XP_018156582.1 CHIG|XP_018159643.1 CHIG|XP_018162994.1 CHIG|XP_018163631.1 CVIN|KAF4892665.1 CVIN|KAF4920749.1 CVIN|KAF4921536.1 CVIN|KAF4921822.1 CVIN|KAF4926589.1 CVYL|A00805 CVYL|A01168 CVYL|A02165 CVYL|A04790 CVYL|A05741 CVYL|A09822 CVYL|A10157 FGRM|XP_011320513.1 FGRM|XP_011322751.1 FGRM|XP_011325361.1 MGRA|XP_003853300.1 MGRA|XP_003854015.1 MGRA|XP_003856157.1 MORY|QBZ53739.1 MORY|QBZ62451.1 NCRA|XP_958971.1 NCRA|XP_959386.1 SSCL|APA14225.1

>Orthogroup125: BCIN|XP_024553752.1 BGRA|VDB92876.1 CFRU|XP_031876915.1 CFRU|XP_031878728.1 CFRU|XP_031882197.1 CFRU|XP_031888045.1 CFRU|XP_031889543.1 CFRU|XP_031893430.1 CGLO|KAF3798332.1 CGLO|KAF3799122.1 CGLO|KAF3799850.1 CGLO|KAF3802058.1 CGLO|KAF3804407.1 CGLO|KAF3809445.1 CGLO|KAF3810482.1 CHIG|XP_018151568.1 CHIG|XP_018151657.1 CHIG|XP_018154189.1 CHIG|XP_018156505.1 CHIG|XP_018156883.1 CHIG|XP_018157047.1 CHIG|XP_018157055.1 CHIG|XP_018158106.1 CHIG|XP_018159130.1 CHIG|XP_018160218.1 CVIN|KAF4901925.1 CVIN|KAF4908441.1 CVIN|KAF4920296.1 CVIN|KAF4921741.1 CVIN|KAF4924627.1 CVIN|KAF4930879.1 CVYL|A03329 CVYL|A03424 CVYL|A04106 CVYL|A08228 CVYL|A09037 CVYL|A09665 CVYL|A14085 MGRA|XP_003853387.1 MGRA|XP_003856163.1 MORY|QBZ58122.1 MORY|QBZ60725.1 MORY|QBZ63077.1 MORY|QBZ64218.1 MORY|QBZ65380.1 NCRA|XP_011395387.1 NCRA|XP_959741.2 SSCL|APA08443.1

>Orthogroup126: MLAR|XP_007404295.1 MLAR|XP_007405413.1 MLAR|XP_007405522.1 MLAR|XP_007405816.1 MLAR|XP_007406359.1 MLAR|XP_007406428.1 MLAR|XP_007406521.1 MLAR|XP_007406592.1 MLAR|XP_007406714.1 MLAR|XP_007406946.1 MLAR|XP_007407021.1 MLAR|XP_007407238.1 MLAR|XP_007407261.1 MLAR|XP_007407275.1 MLAR|XP_007407582.1 MLAR|XP_007407913.1 MLAR|XP_007407921.1 MLAR|XP_007408207.1 MLAR|XP_007408208.1 MLAR|XP_007408743.1 MLAR|XP_007409807.1 MLAR|XP_007410596.1 MLAR|XP_007410695.1 MLAR|XP_007411718.1 MLAR|XP_007412109.1 MLAR|XP_007412322.1 MLAR|XP_007412417.1 MLAR|XP_007412418.1 MLAR|XP_007412738.1 MLAR|XP_007413132.1 MLAR|XP_007413146.1 MLAR|XP_007413197.1 MLAR|XP_007413648.1 MLAR|XP_007413686.1 MLAR|XP_007413867.1 MLAR|XP_007414255.1 MLAR|XP_007414371.1 MLAR|XP_007415609.1 MLAR|XP_007416048.1 MLAR|XP_007416242.1 MLAR|XP_007417280.1 MLAR|XP_007417409.1 MLAR|XP_007417582.1 MLAR|XP_007418748.1 MLAR|XP_007419058.1 MLAR|XP_007419592.1 MLAR|XP_007419687.1 MLAR|XP_007419711.1

>Orthogroup127: ANID|CBF69509.1 ANID|CBF71799.1 ANID|CBF80820.1 ANID|CBF84141.1 ANID|CBF84280.1 ANID|CBF84517.1 ANID|CBF84542.1 BCIN|XP_001559905.2 BCIN|XP_024547380.1 CFRU|XP_031877278.1 CFRU|XP_031878366.1 CFRU|XP_031878749.1 CFRU|XP_031878888.1 CFRU|XP_031878969.1 CFRU|XP_031887532.1 CGLO|KAF3798370.1 CGLO|KAF3806787.1 CGLO|KAF3807843.1 CGLO|KAF3807856.1 CGLO|KAF3810785.1 CHIG|XP_018159454.1 CHIG|XP_018161418.1 CHIG|XP_018162971.1 CHIG|XP_018163044.1 CVIN|KAF4914243.1 CVIN|KAF4922590.1 CVIN|KAF4922605.1 CVIN|KAF4923575.1 CVIN|KAF4928691.1 CVIN|KAF4932151.1 CVYL|A00755 CVYL|A00768 CVYL|A02449 CVYL|A03739 CVYL|A07165 CVYL|A08999 MGRA|XP_003856837.1 MORY|QBZ53554.1 MORY|QBZ56765.1 MORY|QBZ57127.1 MORY|QBZ61770.1 MORY|QBZ62395.1 MORY|QBZ64242.1 MORY|QBZ65439.1 NCRA|XP_011395139.1 NCRA|XP_011395212.1 SSCL|APA10039.1

>Orthogroup128: ANID|CBF73621.1 ANID|CBF76664.1 ANID|CBF80955.1 ANID|CBF85031.1 ANID|CBF87087.1 BCIN|XP_024553305.1 BCIN|XP_024553339.1 BCIN|XP_024553882.1 CFRU|XP_031880650.1 CFRU|XP_031882390.1 CFRU|XP_031882925.1 CFRU|XP_031884070.1 CFRU|XP_031885263.1 CGLO|KAF3797472.1 CGLO|KAF3803400.1 CGLO|KAF3807605.1 CGLO|KAF3807744.1 CGLO|KAF3811132.1 CHIG|XP_018156330.1 CHIG|XP_018160370.1 CHIG|XP_018163016.1 CHIG|XP_018163932.1 CVIN|KAF4888952.1 CVIN|KAF4918415.1 CVIN|KAF4920541.1 CVIN|KAF4924549.1 CVIN|KAF4928945.1 CVYL|A00854 CVYL|A02877 CVYL|A04289 CVYL|A09898 CVYL|A14330 FGRM|XP_011317104.1 FGRM|XP_011319002.1 FGRM|XP_011321904.1 FGRM|XP_011321935.1 FGRM|XP_011325308.1 FGRM|XP_011326924.1 MGRA|XP_003857134.1 MORY|QBZ56921.1 MORY|QBZ61469.1 NCRA|XP_956425.1 NCRA|XP_958839.2 NCRA|XP_964064.3 SSCL|APA10382.1 SSCL|APA13656.1 SSCL|APA15202.1

>Orthogroup129: ANID|CBF74534.1 ANID|CBF78350.1 ANID|CBF83961.1 BCIN|XP_001545339.1 BCIN|XP_024548532.1 BCIN|XP_024549185.1 BGRA|VCU40368.1 CFRU|XP_031879015.1 CFRU|XP_031880819.1 CFRU|XP_031881728.1 CFRU|XP_031885224.1 CFRU|XP_031888776.1 CFRU|XP_031893461.1 CGLO|KAF3797846.1 CGLO|KAF3806942.1 CGLO|KAF3809584.1 CHIG|XP_018151553.1 CHIG|XP_018152602.1 CHIG|XP_018159653.1 CHIG|XP_018160785.1 CHIG|XP_018161398.1 CHIG|XP_018161878.1 CHIG|XP_018162922.1 CVIN|KAF4908865.1 CVIN|KAF4919155.1 CVIN|KAF4919201.1 CVIN|KAF4920300.1 CVIN|KAF4927783.1 CVYL|A06233 CVYL|A06892 CVYL|A09066 CVYL|A11530 FGRM|XP_011315837.1 FGRM|XP_011322441.1 FGRM|XP_011323199.1 FGRM|XP_011327680.1 MGRA|XP_003851101.1 MGRA|XP_003857356.1 MLAR|XP_007414013.1 MORY|QBZ57689.1 MORY|QBZ59832.1 MORY|QBZ60134.1 MORY|QBZ61250.1 MORY|QBZ61959.1 NCRA|XP_958497.1 NCRA|XP_959690.1 SSCL|APA12095.1

>Orthogroup130: ANID|CBF78959.1 ANID|CBF83553.1 BCIN|XP_001548479.1 BCIN|XP_001558280.1 BCIN|XP_001560194.2 BCIN|XP_024549070.1 BCIN|XP_024550704.1 BGRA|VDB91387.1 CFRU|XP_031878402.1 CFRU|XP_031880642.1 CFRU|XP_031880796.1 CFRU|XP_031889671.1 CGLO|KAF3805974.1 CGLO|KAF3806956.1 CGLO|KAF3809950.1 CGLO|KAF3810734.1 CHIG|XP_018156675.1 CHIG|XP_018156677.1 CHIG|XP_018159639.1 CHIG|XP_018162594.1 CHIG|XP_018163118.1 CVIN|KAF4917807.1 CVIN|KAF4919175.1 CVIN|KAF4930743.1 CVIN|KAF4932091.1 CVYL|A01673 CVYL|A06245 CVYL|A07218 CVYL|A13747 FGRM|XP_011318390.1 FGRM|XP_011319307.1 FGRM|XP_011322707.1 MGRA|XP_003847935.1 MGRA|XP_003849611.1 MGRA|XP_003856600.1 MGRA|XP_003856949.1 MLAR|XP_007415574.1 MORY|QBZ58134.1 MORY|QBZ62251.1 MORY|QBZ65171.1 NCRA|XP_011393608.1 NCRA|XP_958412.2 SSCL|APA11538.1 SSCL|APA13673.1 SSCL|APA13826.1 SSCL|APA15858.1 SSCL|APA15893.1

>Orthogroup131: ANID|CBF81936.1 ANID|CBF84391.1 BCIN|XP_001547670.1 BCIN|XP_001550329.2 BCIN|XP_024550944.1 BCIN|XP_024551312.1 CFRU|XP_031878155.1 CFRU|XP_031878560.1 CFRU|XP_031878606.1 CFRU|XP_031879434.1 CFRU|XP_031880106.1 CFRU|XP_031883871.1 CFRU|XP_031884857.1 CFRU|XP_031890083.1 CGLO|KAF3798448.1 CGLO|KAF3798480.1 CGLO|KAF3798744.1 CGLO|KAF3799654.1 CGLO|KAF3801858.1 CGLO|KAF3805049.1 CGLO|KAF3811802.1 CGLO|KAF3812174.1 CHIG|XP_018151751.1 CHIG|XP_018153396.1 CHIG|XP_018159374.1 CVIN|KAF4901938.1 CVIN|KAF4917466.1 CVIN|KAF4918755.1 CVIN|KAF4922194.1 CVIN|KAF4924874.1 CVYL|A00216 CVYL|A06508 CVYL|A12806 CVYL|A12849 CVYL|A13517 FGRM|XP_011322931.1 FGRM|XP_011324282.1 FGRM|XP_011327501.1 MGRA|XP_003852830.1 MGRA|XP_003854300.1 MORY|QBZ53456.1 MORY|QBZ54441.1 NCRA|XP_958745.1 NCRA|XP_963311.2 SSCL|APA05876.1 SSCL|APA10010.1 SSCL|APA11716.1

>Orthogroup132: ANID|CBF88914.1 BCIN|XP_001546453.1 CFRU|XP_031875896.1 CFRU|XP_031879493.1 CFRU|XP_031879916.1 CFRU|XP_031881489.1 CFRU|XP_031883484.1 CFRU|XP_031888697.1 CFRU|XP_031890895.1 CFRU|XP_031892535.1 CGLO|KAF3797056.1 CGLO|KAF3799009.1 CGLO|KAF3802435.1 CGLO|KAF3804300.1 CGLO|KAF3805801.1 CGLO|KAF3807822.1 CGLO|KAF3811764.1 CGLO|KAF3811937.1 CHIG|XP_018152915.1 CHIG|XP_018154156.1 CHIG|XP_018159599.1 CHIG|XP_018162989.1 CVIN|KAF4890319.1 CVIN|KAF4908006.1 CVIN|KAF4908648.1 CVIN|KAF4920111.1 CVIN|KAF4920935.1 CVIN|KAF4921533.1 CVYL|A00790 CVYL|A03982 CVYL|A06021 CVYL|A06129 CVYL|A10648 CVYL|A13578 FGRM|XP_011321589.1 FGRM|XP_011321837.1 FGRM|XP_011322423.1 FGRM|XP_011323339.1 FGRM|XP_011323391.1 FGRM|XP_011323530.1 MGRA|XP_003855288.1 MGRA|XP_003856173.1 MORY|QBZ59333.1 MORY|QBZ61716.1 NCRA|XP_001727984.2 SSCL|APA08278.1 SSCL|APA13744.1

>Orthogroup133: BCIN|XP_024552368.1 BGRA|VCU40389.1 CFRU|XP_031876676.1 CFRU|XP_031880641.1 CFRU|XP_031884173.1 CFRU|XP_031890925.1 CFRU|XP_031891105.1 CFRU|XP_031893499.1 CGLO|KAF3800857.1 CGLO|KAF3801482.1 CGLO|KAF3801916.1 CGLO|KAF3803762.1 CGLO|KAF3809949.1 CGLO|KAF3811743.1 CHIG|XP_018150837.1 CHIG|XP_018151807.1 CHIG|XP_018151907.1 CHIG|XP_018153158.1 CHIG|XP_018159421.1 CHIG|XP_018161530.1 CVIN|KAF4915562.1 CVIN|KAF4917892.1 CVIN|KAF4918694.1 CVIN|KAF4922992.1 CVIN|KAF4930742.1 CVYL|A01674 CVYL|A02354 CVYL|A11096 CVYL|A12647 CVYL|A12758 CVYL|A14142 FGRM|XP_011319797.1 MLAR|XP_007405821.1 MLAR|XP_007406343.1 MLAR|XP_007406344.1 MLAR|XP_007407076.1 MLAR|XP_007412523.1 MLAR|XP_007413889.1 MLAR|XP_007413890.1 MLAR|XP_007413891.1 MLAR|XP_007416261.1 MORY|QBZ59804.1 MORY|QBZ60282.1 MORY|QBZ64201.1 MORY|QBZ65172.1 NCRA|XP_965647.2 SSCL|APA13230.1

>Orthogroup134: ANID|CBF70352.1 ANID|CBF73965.1 ANID|CBF80419.1 ANID|CBF82272.1 ANID|CBF85559.1 ANID|CBF87267.1 BCIN|XP_024545900.1 BCIN|XP_024546641.1 CFRU|XP_031877274.1 CFRU|XP_031877359.1 CFRU|XP_031883509.1 CFRU|XP_031888838.1 CFRU|XP_031889158.1 CFRU|XP_031890072.1 CGLO|KAF3799864.1 CGLO|KAF3802821.1 CGLO|KAF3804226.1 CGLO|KAF3811929.1 CHIG|XP_018153797.1 CHIG|XP_018156538.1 CHIG|XP_018158005.1 CHIG|XP_018158164.1 CHIG|XP_018158165.1 CHIG|XP_018158300.1 CHIG|XP_018159138.1 CHIG|XP_018160074.1 CHIG|XP_018161953.1 CHIG|XP_018164821.1 CVIN|KAF4894123.1 CVIN|KAF4908659.1 CVIN|KAF4921703.1 CVIN|KAF4930179.1 CVYL|A00952 CVYL|A02442 CVYL|A02443 CVYL|A03918 CVYL|A06136 CVYL|A09653 CVYL|A12424 FGRM|XP_011315648.1 FGRM|XP_011317659.1 FGRM|XP_011320985.1 FGRM|XP_011325124.1 MORY|QBZ63365.1 MORY|QBZ64313.1 SSCL|APA15879.1

>Orthogroup135: ANID|CBF71525.1 ANID|CBF74681.1 ANID|CBF76715.1 ANID|CBF83169.1 ANID|CBF84393.1 ANID|CBF86693.1 ANID|CBF87142.1 BCIN|XP_024547453.1 BCIN|XP_024549614.1 BCIN|XP_024551888.1 BGRA|VCU39914.1 BGRA|VDB90341.1 CFRU|XP_031878897.1 CFRU|XP_031883671.1 CFRU|XP_031890749.1 CFRU|XP_031893516.1 CGLO|KAF3800231.1 CGLO|KAF3807899.1 CGLO|KAF3809593.1 CGLO|KAF3811724.1 CHIG|XP_018152597.1 CHIG|XP_018155075.1 CHIG|XP_018159769.1 CHIG|XP_018160786.1 CHIG|XP_018163068.1 CVIN|KAF4915248.1 CVIN|KAF4919205.1 CVIN|KAF4921799.1 CVIN|KAF4922977.1 CVYL|A00715 CVYL|A08442 CVYL|A12406 CVYL|A12741 FGRM|XP_011318370.1 FGRM|XP_011320794.1 FGRM|XP_011320946.1 FGRM|XP_011321156.1 FGRM|XP_011323697.1 FGRM|XP_011327268.1 MGRA|XP_003849935.1 MGRA|XP_003855503.1 MORY|QBZ65639.1 NCRA|XP_958515.2 SSCL|APA06225.1 SSCL|APA13991.1 SSCL|APA15995.1

>Orthogroup136: ANID|CBF73650.1 ANID|CBF75136.1 ANID|CBF77551.1 ANID|CBF87158.1 BCIN|XP_024545925.1 BCIN|XP_024547656.1 BGRA|VCU38850.1 CFRU|XP_031880487.1 CFRU|XP_031881676.1 CFRU|XP_031883599.1 CFRU|XP_031887095.1 CFRU|XP_031888822.1 CGLO|KAF3807449.1 CGLO|KAF3810819.1 CGLO|KAF3811340.1 CGLO|KAF3811581.1 CHIG|XP_018154974.1 CHIG|XP_018156780.1 CHIG|XP_018160598.1 CHIG|XP_018160846.1 CVIN|KAF4899853.1 CVIN|KAF4914888.1 CVIN|KAF4918393.1 CVIN|KAF4918531.1 CVIN|KAF4927211.1 CVYL|A02007 CVYL|A03432 CVYL|A03859 CVYL|A04522 CVYL|A10957 FGRM|XP_011317652.1 FGRM|XP_011321985.1 FGRM|XP_011323466.1 FGRM|XP_011323846.1 MGRA|XP_003847750.1 MGRA|XP_003848175.1 MGRA|XP_003854788.1 MGRA|XP_003855943.1 MLAR|XP_007407154.1 MLAR|XP_007412606.1 MORY|QBZ58222.1 MORY|QBZ63441.1 NCRA|XP_001728310.2 NCRA|XP_961900.1 SSCL|APA07339.1 SSCL|APA07741.1

>Orthogroup137: ANID|CBF74597.1 ANID|CBF75574.1 ANID|CBF87107.1 ANID|CBF88868.1 BCIN|XP_001545487.1 BCIN|XP_024548132.1 BCIN|XP_024550246.1 BCIN|XP_024551762.1 BCIN|XP_024552440.1 BCIN|XP_024554067.1 CFRU|XP_031875807.1 CFRU|XP_031880008.1 CFRU|XP_031887059.1 CFRU|XP_031888991.1 CFRU|XP_031891430.1 CFRU|XP_031891505.1 CFRU|XP_031892027.1 CGLO|KAF3799288.1 CGLO|KAF3801439.1 CGLO|KAF3801783.1 CGLO|KAF3804647.1 CGLO|KAF3804734.1 CGLO|KAF3806741.1 CGLO|KAF3808045.1 CGLO|KAF3811211.1 CHIG|XP_018151314.1 CHIG|XP_018159287.1 CHIG|XP_018159874.1 CHIG|XP_018161720.1 CVIN|KAF4913900.1 CVIN|KAF4916997.1 CVIN|KAF4922341.1 CVIN|KAF4923366.1 CVIN|KAF4924787.1 CVYL|A03529 CVYL|A05238 CVYL|A07645 CVYL|A12925 FGRM|XP_011323690.1 FGRM|XP_011327389.1 FGRM|XP_011328278.1 MGRA|XP_003854718.1 MLAR|XP_007404451.1 MORY|QBZ59771.1 SSCL|APA05325.1 SSCL|APA15000.1

>Orthogroup138: ANID|CBF81021.1 ANID|CBF89371.1 BCIN|XP_001550707.1 BCIN|XP_001555330.1 BCIN|XP_024550696.1 CFRU|XP_031884060.1 CFRU|XP_031884252.1 CFRU|XP_031885726.1 CFRU|XP_031889510.1 CFRU|XP_031892088.1 CGLO|KAF3799997.1 CGLO|KAF3800119.1 CGLO|KAF3804994.1 CGLO|KAF3805898.1 CGLO|KAF3810033.1 CHIG|XP_018153042.1 CHIG|XP_018157740.1 CHIG|XP_018158912.1 CHIG|XP_018163524.1 CHIG|XP_018165056.1 CVIN|KAF4917949.1 CVIN|KAF4920797.1 CVIN|KAF4925806.1 CVIN|KAF4926915.1 CVIN|KAF4927127.1 CVYL|A02622 CVYL|A06556 CVYL|A06807 CVYL|A10213 FGRM|XP_011316255.1 MGRA|XP_003852232.1 MLAR|XP_007415254.1 MLAR|XP_007415255.1 MLAR|XP_007415325.1 MLAR|XP_007416070.1 MLAR|XP_007419683.1 MORY|QBZ54144.1 MORY|QBZ59851.1 MORY|QBZ65195.1 NCRA|XP_956431.1 NCRA|XP_957090.1 NCRA|XP_957642.2 NCRA|XP_962498.1 SSCL|APA08612.1 SSCL|APA11530.1 SSCL|APA15366.1

>Orthogroup139: ANID|CBF84467.1 ANID|CBF84576.1 ANID|CBF89610.1 BCIN|XP_024548393.1 BCIN|XP_024549349.1 BCIN|XP_024549382.1 BCIN|XP_024553443.1 CFRU|XP_031877041.1 CFRU|XP_031877931.1 CFRU|XP_031880116.1 CFRU|XP_031884103.1 CFRU|XP_031889591.1 CGLO|KAF3799994.1 CGLO|KAF3810463.1 CGLO|KAF3810955.1 CGLO|KAF3811118.1 CGLO|KAF3811792.1 CHIG|XP_018152932.1 CHIG|XP_018153035.1 CHIG|XP_018154884.1 CHIG|XP_018159193.1 CVIN|KAF4905162.1 CVIN|KAF4907252.1 CVIN|KAF4924613.1 CVIN|KAF4924921.1 CVIN|KAF4926913.1 CVYL|A02193 CVYL|A07970 CVYL|A10209 CVYL|A12797 CVYL|A14064 FGRM|XP_011317849.1 FGRM|XP_011321171.1 FGRM|XP_011321779.1 FGRM|XP_011322655.1 FGRM|XP_011323749.1 MGRA|XP_003849497.1 MGRA|XP_003854012.1 MORY|QBZ53446.1 MORY|QBZ63151.1 MORY|QBZ64116.1 NCRA|XP_960645.1 NCRA|XP_964938.1 SSCL|APA09034.1 SSCL|APA13856.1 SSCL|APA15783.1

>Orthogroup140: ANID|CBF87091.1 BCIN|XP_001554560.1 BCIN|XP_001560195.1 BCIN|XP_024547563.1 BCIN|XP_024549183.1 BGRA|VCU40901.1 CFRU|XP_031878928.1 CFRU|XP_031880911.1 CFRU|XP_031889575.1 CFRU|XP_031890061.1 CFRU|XP_031891182.1 CFRU|XP_031893235.1 CGLO|KAF3798762.1 CGLO|KAF3800808.1 CGLO|KAF3804741.1 CGLO|KAF3806935.1 CGLO|KAF3807854.1 CGLO|KAF3810576.1 CHIG|XP_018151254.1 CHIG|XP_018159029.1 CHIG|XP_018163046.1 CVIN|KAF4903558.1 CVIN|KAF4912776.1 CVIN|KAF4914230.1 CVIN|KAF4919182.1 CVIN|KAF4922503.1 CVIN|KAF4922603.1 CVYL|A00757 CVYL|A05379 CVYL|A06227 CVYL|A08743 CVYL|A09158 CVYL|A13655 FGRM|XP_011321041.1 FGRM|XP_011324389.1 MGRA|XP_003850136.1 MGRA|XP_003851389.1 MGRA|XP_003852366.1 MGRA|XP_003855991.1 MLAR|XP_007410664.1 MLAR|XP_007416103.1 MORY|QBZ59034.1 NCRA|XP_960900.3 SSCL|APA11911.1 SSCL|APA15023.1 SSCL|APA15709.1

>Orthogroup141: BCIN|XP_001547159.1 BCIN|XP_001547732.2 BCIN|XP_001553490.1 BCIN|XP_024549497.1 BCIN|XP_024553142.1 CFRU|XP_031878516.1 CFRU|XP_031879001.1 CFRU|XP_031884008.1 CFRU|XP_031884553.1 CFRU|XP_031885712.1 CGLO|KAF3799802.1 CGLO|KAF3805020.1 CGLO|KAF3810150.1 CGLO|KAF3810318.1 CHIG|XP_018150948.1 CHIG|XP_018157982.1 CHIG|XP_018158341.1 CHIG|XP_018162978.1 CHIG|XP_018163500.1 CVIN|KAF4891715.1 CVIN|KAF4892663.1 CVIN|KAF4894262.1 CVIN|KAF4927095.1 CVIN|KAF4929100.1 CVYL|A00775 CVYL|A02740 CVYL|A04791 CVYL|A05785 CVYL|A06531 FGRM|XP_011316494.1 FGRM|XP_011316495.1 FGRM|XP_011319679.1 FGRM|XP_011321566.1 MGRA|XP_003851544.1 MGRA|XP_003855273.1 MORY|QBZ55158.1 MORY|QBZ55171.1 MORY|QBZ60699.1 MORY|QBZ62023.1 MORY|QBZ65259.1 NCRA|XP_956657.1 NCRA|XP_958729.1 NCRA|XP_958730.1 SSCL|APA05380.1 SSCL|APA08929.1 SSCL|APA13322.1

>Orthogroup142: ANID|CBF71114.1 ANID|CBF90341.1 BCIN|XP_001553032.1 CFRU|XP_031879332.1 CFRU|XP_031882405.1 CFRU|XP_031884753.1 CFRU|XP_031888011.1 CFRU|XP_031888162.1 CFRU|XP_031890934.1 CGLO|KAF3801007.1 CGLO|KAF3801562.1 CGLO|KAF3802101.1 CGLO|KAF3807528.1 CGLO|KAF3808653.1 CHIG|XP_018151985.1 CHIG|XP_018154123.1 CHIG|XP_018156409.1 CHIG|XP_018158154.1 CHIG|XP_018161857.1 CVIN|KAF4912989.1 CVIN|KAF4914255.1 CVIN|KAF4917991.1 CVIN|KAF4921093.1 CVIN|KAF4926194.1 CVIN|KAF4928876.1 CVYL|A02957 CVYL|A03290 CVYL|A03376 CVYL|A05899 CVYL|A11878 CVYL|A12572 FGRM|XP_011318602.1 FGRM|XP_011321641.1 FGRM|XP_011321857.1 FGRM|XP_011323212.1 MGRA|XP_003849920.1 MORY|QBZ55217.1 MORY|QBZ61307.1 MORY|QBZ61904.1 MORY|QBZ63657.1 MORY|QBZ65235.1 NCRA|XP_957508.1 NCRA|XP_959407.1 NCRA|XP_960049.1 SSCL|APA11270.1

>Orthogroup143: ANID|CBF71426.1 ANID|CBF78842.1 BCIN|XP_001561195.1 BCIN|XP_024549498.1 BCIN|XP_024552380.1 CFRU|XP_031876840.1 CFRU|XP_031877454.1 CFRU|XP_031879547.1 CFRU|XP_031879643.1 CFRU|XP_031880895.1 CFRU|XP_031884824.1 CFRU|XP_031890160.1 CGLO|KAF3797974.1 CGLO|KAF3798445.1 CGLO|KAF3801048.1 CGLO|KAF3802728.1 CGLO|KAF3802748.1 CGLO|KAF3802849.1 CHIG|XP_018159617.1 CHIG|XP_018159787.1 CVIN|KAF4890511.1 CVIN|KAF4910476.1 CVIN|KAF4910779.1 CVIN|KAF4914944.1 CVIN|KAF4918771.1 CVIN|KAF4920022.1 CVYL|A00333 CVYL|A05164 CVYL|A06263 CVYL|A08051 CVYL|A08072 CVYL|A08073 CVYL|A08380 CVYL|A11194 FGRM|XP_011317883.1 FGRM|XP_011322122.1 FGRM|XP_011322678.1 FGRM|XP_011322777.1 MGRA|XP_003850422.1 MGRA|XP_003852307.1 MORY|QBZ54763.1 MORY|QBZ57542.1 MORY|QBZ60181.1 NCRA|XP_961743.3 SSCL|APA13318.1

>Orthogroup144: ANID|CBF80149.1 ANID|CBF81244.1 ANID|CBF82319.1 BCIN|XP_001556044.2 BCIN|XP_001559546.1 BCIN|XP_024546007.1 BCIN|XP_024546161.1 BCIN|XP_024551903.1 BCIN|XP_024552396.1 CFRU|XP_031876811.1 CFRU|XP_031881990.1 CFRU|XP_031890689.1 CFRU|XP_031891801.1 CFRU|XP_031892269.1 CFRU|XP_031892613.1 CGLO|KAF3797125.1 CGLO|KAF3797963.1 CGLO|KAF3801254.1 CGLO|KAF3806110.1 CGLO|KAF3806747.1 CGLO|KAF3809762.1 CHIG|XP_018153375.1 CHIG|XP_018157090.1 CHIG|XP_018158723.1 CHIG|XP_018160187.1 CHIG|XP_018164549.1 CVIN|KAF4896764.1 CVIN|KAF4896909.1 CVIN|KAF4902339.1 CVIN|KAF4914951.1 CVIN|KAF4919511.1 CVIN|KAF4921933.1 CVYL|A06683 CVYL|A07403 CVYL|A07836 CVYL|A10577 CVYL|A11236 CVYL|A14059 FGRM|XP_011318277.1 FGRM|XP_011321371.1 MGRA|XP_003856968.1 MORY|QBZ61780.1 SSCL|APA07322.1 SSCL|APA13302.1 SSCL|APA13955.1

>Orthogroup145: MLAR|XP_007403520.1 MLAR|XP_007403730.1 MLAR|XP_007403952.1 MLAR|XP_007404153.1 MLAR|XP_007405438.1 MLAR|XP_007405803.1 MLAR|XP_007405804.1 MLAR|XP_007406576.1 MLAR|XP_007406756.1 MLAR|XP_007406780.1 MLAR|XP_007406995.1 MLAR|XP_007409015.1 MLAR|XP_007409016.1 MLAR|XP_007409710.1 MLAR|XP_007409830.1 MLAR|XP_007409915.1 MLAR|XP_007409988.1 MLAR|XP_007410371.1 MLAR|XP_007411248.1 MLAR|XP_007411428.1 MLAR|XP_007411561.1 MLAR|XP_007411665.1 MLAR|XP_007411908.1 MLAR|XP_007412098.1 MLAR|XP_007412739.1 MLAR|XP_007413175.1 MLAR|XP_007414384.1 MLAR|XP_007414432.1 MLAR|XP_007415089.1 MLAR|XP_007415437.1 MLAR|XP_007415438.1 MLAR|XP_007416518.1 MLAR|XP_007416708.1 MLAR|XP_007416923.1 MLAR|XP_007417694.1 MLAR|XP_007417775.1 MLAR|XP_007418142.1 MLAR|XP_007418739.1 MLAR|XP_007418943.1 MLAR|XP_007419011.1 MLAR|XP_007419012.1 MLAR|XP_007419183.1 MLAR|XP_007419312.1 MLAR|XP_007419410.1 MLAR|XP_007419587.1

>Orthogroup146: ANID|CBF73562.1 BCIN|XP_001560457.2 BCIN|XP_024551397.1 CFRU|XP_031876821.1 CFRU|XP_031878397.1 CFRU|XP_031884068.1 CFRU|XP_031884546.1 CFRU|XP_031886117.1 CFRU|XP_031890297.1 CGLO|KAF3797976.1 CGLO|KAF3802865.1 CGLO|KAF3805685.1 CGLO|KAF3810362.1 CGLO|KAF3810779.1 CGLO|KAF3810835.1 CHIG|XP_018155251.1 CHIG|XP_018155362.1 CHIG|XP_018158019.1 CHIG|XP_018158361.1 CHIG|XP_018162534.1 CVIN|KAF4911113.1 CVIN|KAF4914945.1 CVIN|KAF4923838.1 CVIN|KAF4932076.1 CVYL|A05148 CVYL|A05619 CVYL|A05750 CVYL|A07171 CVYL|A08379 FGRM|XP_011316893.1 FGRM|XP_011317571.1 FGRM|XP_011320949.1 FGRM|XP_011322414.1 FGRM|XP_011322608.1 FGRM|XP_011327046.1 MGRA|XP_003849035.1 MGRA|XP_003849857.1 MLAR|XP_007404007.1 MLAR|XP_007410685.1 MLAR|XP_007416034.1 MORY|QBZ58097.1 NCRA|XP_963056.1 SSCL|APA10631.1 SSCL|APA15707.1

>Orthogroup147: ANID|CBF73576.1 ANID|CBF74489.1 ANID|CBF78955.1 ANID|CBF81975.1 ANID|CBF82093.1 ANID|CBF87292.1 ANID|CBF87758.1 BCIN|XP_001550224.1 BCIN|XP_024546779.1 BCIN|XP_024550621.1 BCIN|XP_024552616.1 BCIN|XP_024553581.1 BCIN|XP_024553966.1 CFRU|XP_031879459.1 CFRU|XP_031880312.1 CFRU|XP_031882917.1 CFRU|XP_031884264.1 CFRU|XP_031892111.1 CGLO|KAF3800058.1 CGLO|KAF3802398.1 CGLO|KAF3803793.1 CGLO|KAF3808993.1 CHIG|XP_018153123.1 CHIG|XP_018154689.1 CHIG|XP_018155254.1 CHIG|XP_018165006.1 CVIN|KAF4917902.1 CVIN|KAF4918025.1 CVIN|KAF4919321.1 CVIN|KAF4922201.1 CVIN|KAF4923694.1 CVYL|A01413 CVYL|A06757 CVYL|A13541 CVYL|A14165 CVYL|A14228 FGRM|XP_011317845.1 FGRM|XP_011320787.1 FGRM|XP_011320881.1 MORY|QBZ58158.1 MORY|QBZ60135.1 NCRA|XP_963883.1 SSCL|APA15424.1 SSCL|APA16337.1

>Orthogroup148: ANID|CBF75291.1 ANID|CBF78632.1 ANID|CBF90243.1 BCIN|XP_001560329.2 BGRA|VCU40597.1 CFRU|XP_031875929.1 CFRU|XP_031876129.1 CFRU|XP_031880097.1 CFRU|XP_031882991.1 CFRU|XP_031883855.1 CFRU|XP_031885916.1 CFRU|XP_031889948.1 CGLO|KAF3797723.1 CGLO|KAF3800762.1 CGLO|KAF3802902.1 CGLO|KAF3805038.1 CGLO|KAF3805709.1 CGLO|KAF3810298.1 CGLO|KAF3811983.1 CHIG|XP_018155257.1 CHIG|XP_018158471.1 CHIG|XP_018163468.1 CHIG|XP_018163617.1 CVIN|KAF4907429.1 CVIN|KAF4907629.1 CVIN|KAF4917559.1 CVIN|KAF4919662.1 CVIN|KAF4927144.1 CVIN|KAF4930145.1 CVIN|KAF4930429.1 CVYL|A00442 CVYL|A05111 CVYL|A05525 CVYL|A06513 CVYL|A09838 CVYL|A11428 FGRM|XP_011316578.1 FGRM|XP_011321433.1 FGRM|XP_011326396.1 MGRA|XP_003856309.1 MLAR|XP_007412991.1 MORY|QBZ55982.1 NCRA|XP_011392863.1 SSCL|APA08817.1

>Orthogroup149: ANID|CBF75478.1 ANID|CBF78525.1 ANID|CBF87080.1 BCIN|XP_024553433.1 CFRU|XP_031878455.1 CFRU|XP_031878456.1 CFRU|XP_031878457.1 CGLO|KAF3798587.1 CGLO|KAF3798588.1 CGLO|KAF3798589.1 CGLO|KAF3798590.1 CGLO|KAF3801032.1 CHIG|XP_018151833.1 CHIG|XP_018155491.1 CHIG|XP_018156769.1 CHIG|XP_018157750.1 CHIG|XP_018163400.1 CHIG|XP_018163494.1 CVIN|KAF4930080.1 CVIN|KAF4930112.1 CVYL|A04854 CVYL|A04855 CVYL|A04857 FGRM|XP_011318416.1 FGRM|XP_011325188.1 MGRA|XP_003847510.1 MGRA|XP_003847714.1 MGRA|XP_003848376.1 MGRA|XP_003848658.1 MGRA|XP_003852169.1 MGRA|XP_003852417.1 MGRA|XP_003853491.1 MORY|QBZ57549.1 MORY|QBZ63464.1 MORY|QBZ65556.1 NCRA|XP_958119.1 NCRA|XP_959099.1 NCRA|XP_959568.1 NCRA|XP_962743.1 NCRA|XP_962746.1 SSCL|APA07397.1 SSCL|APA12528.1 SSCL|APA12741.1 SSCL|APA13865.1

>Orthogroup150: ANID|CBF76225.1 ANID|CBF82947.1 ANID|CBF83182.1 BCIN|XP_024549926.1 CFRU|XP_031879996.1 CFRU|XP_031882376.1 CFRU|XP_031882672.1 CFRU|XP_031882673.1 CFRU|XP_031884727.1 CFRU|XP_031884728.1 CFRU|XP_031887756.1 CFRU|XP_031891406.1 CFRU|XP_031891906.1 CFRU|XP_031892295.1 CGLO|KAF3797185.1 CGLO|KAF3800449.1 CGLO|KAF3804615.1 CGLO|KAF3805184.1 CGLO|KAF3805185.1 CGLO|KAF3807564.1 CGLO|KAF3809723.1 CGLO|KAF3811632.1 CHIG|XP_018154880.1 CHIG|XP_018156373.1 CHIG|XP_018157770.1 CVIN|KAF4889562.1 CVIN|KAF4905174.1 CVIN|KAF4911889.1 CVIN|KAF4919075.1 CVIN|KAF4928905.1 CVYL|A02918 CVYL|A05264 FGRM|XP_011318351.1 FGRM|XP_011318932.1 FGRM|XP_011319589.1 FGRM|XP_011323081.1 FGRM|XP_011325768.1 MGRA|XP_003854383.1 MORY|QBZ57115.1 MORY|QBZ59754.1 NCRA|XP_959857.2 NCRA|XP_961087.3 NCRA|XP_961522.3 SSCL|APA05262.1

>Orthogroup151: ANID|CBF78208.1 ANID|CBF82171.1 ANID|CBF83124.1 BCIN|XP_024550736.1 BCIN|XP_024550764.1 BGRA|VDB91158.1 CFRU|XP_031875943.1 CFRU|XP_031884673.1 CFRU|XP_031888207.1 CFRU|XP_031888835.1 CFRU|XP_031891795.1 CFRU|XP_031892293.1 CGLO|KAF3797188.1 CGLO|KAF3799358.1 CGLO|KAF3799758.1 CGLO|KAF3800054.1 CGLO|KAF3802261.1 CHIG|XP_018154374.1 CHIG|XP_018159943.1 CHIG|XP_018159944.1 CHIG|XP_018164999.1 CVIN|KAF4910729.1 CVIN|KAF4918954.1 CVIN|KAF4919329.1 CVIN|KAF4920806.1 CVIN|KAF4924399.1 CVYL|A04586 CVYL|A06751 CVYL|A10509 CVYL|A11006 CVYL|A11333 FGRM|XP_011318252.1 FGRM|XP_011322168.1 MGRA|XP_003847787.1 MGRA|XP_003848157.1 MGRA|XP_003849331.1 MGRA|XP_003850817.1 MGRA|XP_003851234.1 MGRA|XP_003852310.1 MGRA|XP_003854939.1 MGRA|XP_003857473.1 NCRA|XP_964645.3 SSCL|APA11640.1 SSCL|APA11685.1

>Orthogroup152: ANID|CBF78913.1 CFRU|XP_031876118.1 CFRU|XP_031877144.1 CFRU|XP_031882319.1 CFRU|XP_031883864.1 CFRU|XP_031892110.1 CGLO|KAF3800057.1 CGLO|KAF3804018.1 CGLO|KAF3804384.1 CGLO|KAF3805174.1 CGLO|KAF3807367.1 CHIG|XP_018150929.1 CHIG|XP_018156848.1 CHIG|XP_018157666.1 CHIG|XP_018160164.1 CHIG|XP_018165007.1 CVIN|KAF4894251.1 CVIN|KAF4919341.1 CVIN|KAF4920082.1 CVIN|KAF4925179.1 CVIN|KAF4927469.1 CVYL|A04080 CVYL|A06577 CVYL|A06756 CVYL|A08786 CVYL|A10863 FGRM|XP_011320776.1 FGRM|XP_011326069.1 MGRA|XP_003847816.1 MGRA|XP_003848313.1 MGRA|XP_003851195.1 MGRA|XP_003851558.1 MORY|QBZ58156.1 MORY|QBZ59117.1 MORY|QBZ59825.1 MORY|QBZ59965.1 MORY|QBZ61968.1 MORY|QBZ64360.1 MORY|QBZ64974.1 MORY|QBZ66329.1 MORY|QBZ66335.1 NCRA|XP_955868.1 NCRA|XP_956654.2 NCRA|XP_964777.3

>Orthogroup153: ANID|CBF80914.1 ANID|CBF84784.1 ANID|CBF84786.1 ANID|CBF86102.1 BCIN|XP_001557140.1 BCIN|XP_024547856.1 BCIN|XP_024548553.1 BGRA|VCU40213.1 BGRA|VCU40809.1 BGRA|VDB94616.1 CFRU|XP_031877985.1 CFRU|XP_031885608.1 CFRU|XP_031890547.1 CGLO|KAF3799612.1 CGLO|KAF3806696.1 CGLO|KAF3810086.1 CHIG|XP_018153414.1 CHIG|XP_018154607.1 CHIG|XP_018157905.1 CHIG|XP_018158669.1 CVIN|KAF4912751.1 CVIN|KAF4925961.1 CVIN|KAF4926452.1 CVYL|A02680 CVYL|A13474 CVYL|A14001 FGRM|XP_011316555.1 FGRM|XP_011316658.1 FGRM|XP_011328252.1 MGRA|XP_003848822.1 MGRA|XP_003852514.1 MGRA|XP_003857319.1 MLAR|XP_007410255.1 MLAR|XP_007411134.1 MLAR|XP_007416987.1 MORY|QBZ53903.1 MORY|QBZ54302.1 MORY|QBZ62570.1 NCRA|XP_956567.1 NCRA|XP_963366.1 NCRA|XP_964602.3 SSCL|APA08175.1 SSCL|APA08557.1 SSCL|APA10065.1

>Orthogroup154: ANID|CBF71834.1 BCIN|XP_024550746.1 CFRU|XP_031877484.1 CFRU|XP_031878307.1 CFRU|XP_031882255.1 CFRU|XP_031887326.1 CFRU|XP_031888841.1 CFRU|XP_031889722.1 CFRU|XP_031890166.1 CFRU|XP_031890249.1 CFRU|XP_031890258.1 CGLO|KAF3798095.1 CGLO|KAF3798442.1 CGLO|KAF3802826.1 CGLO|KAF3808109.1 CHIG|XP_018154221.1 CHIG|XP_018154230.1 CHIG|XP_018155763.1 CHIG|XP_018158143.1 CHIG|XP_018160012.1 CHIG|XP_018164884.1 CHIG|XP_018164887.1 CVIN|KAF4894121.1 CVIN|KAF4905809.1 CVIN|KAF4905811.1 CVIN|KAF4920046.1 CVIN|KAF4927981.1 CVYL|A04004 CVYL|A04006 CVYL|A05187 CVYL|A08125 CVYL|A08265 CVYL|A12431 CVYL|A13774 FGRM|XP_011318034.1 FGRM|XP_011327553.1 FGRM|XP_011327556.1 FGRM|XP_011328738.1 MORY|QBZ55884.1 MORY|QBZ55887.1 NCRA|XP_001728179.1 NCRA|XP_961942.2 SSCL|APA11670.1

>Orthogroup155: ANID|CBF80543.1 BCIN|XP_001560963.1 BCIN|XP_024547943.1 BCIN|XP_024551676.1 BCIN|XP_024553508.1 BGRA|VDB89490.1 BGRA|VDB91171.1 BGRA|VDB91172.1 CFRU|XP_031881204.1 CFRU|XP_031890464.1 CFRU|XP_031891043.1 CGLO|KAF3801624.1 CGLO|KAF3804929.1 CGLO|KAF3810053.1 CHIG|XP_018150509.1 CHIG|XP_018151177.1 CHIG|XP_018151386.1 CHIG|XP_018151648.1 CHIG|XP_018155224.1 CHIG|XP_018163311.1 CHIG|XP_018164430.1 CVIN|KAF4916875.1 CVIN|KAF4919409.1 CVIN|KAF4922711.1 CVYL|A02066 CVYL|A02646 CVYL|A12125 CVYL|A12512 FGRM|XP_011324191.1 FGRM|XP_011328195.1 MGRA|XP_003848151.1 MGRA|XP_003850617.1 MGRA|XP_003853246.1 MORY|QBZ53477.1 MORY|QBZ56391.1 MORY|QBZ62674.1 NCRA|XP_958631.3 NCRA|XP_964823.2 NCRA|XP_964932.3 SSCL|APA06008.1 SSCL|APA06690.1 SSCL|APA08416.1 SSCL|APA13785.1

>Orthogroup156: MLAR|XP_007403606.1 MLAR|XP_007404576.1 MLAR|XP_007405303.1 MLAR|XP_007406148.1 MLAR|XP_007406519.1 MLAR|XP_007406520.1 MLAR|XP_007406554.1 MLAR|XP_007407068.1 MLAR|XP_007407680.1 MLAR|XP_007407702.1 MLAR|XP_007407767.1 MLAR|XP_007407859.1 MLAR|XP_007407860.1 MLAR|XP_007408054.1 MLAR|XP_007409164.1 MLAR|XP_007409441.1 MLAR|XP_007410410.1 MLAR|XP_007410488.1 MLAR|XP_007410502.1 MLAR|XP_007411427.1 MLAR|XP_007411771.1 MLAR|XP_007411865.1 MLAR|XP_007412093.1 MLAR|XP_007412413.1 MLAR|XP_007412414.1 MLAR|XP_007412721.1 MLAR|XP_007413677.1 MLAR|XP_007414042.1 MLAR|XP_007414822.1 MLAR|XP_007414827.1 MLAR|XP_007415312.1 MLAR|XP_007416384.1 MLAR|XP_007416644.1 MLAR|XP_007416872.1 MLAR|XP_007417170.1 MLAR|XP_007417778.1 MLAR|XP_007418496.1 MLAR|XP_007418708.1 MLAR|XP_007418726.1 MLAR|XP_007418758.1 MLAR|XP_007418837.1 MLAR|XP_007419363.1 MLAR|XP_007419652.1

>Orthogroup157: ANID|CBF70180.1 ANID|CBF78855.1 ANID|CBF84194.1 ANID|CBF86646.1 ANID|CBF86674.1 BCIN|XP_024548665.1 BCIN|XP_024548887.1 CFRU|XP_031875841.1 CFRU|XP_031879017.1 CFRU|XP_031884114.1 CFRU|XP_031886357.1 CFRU|XP_031888790.1 CGLO|KAF3799664.1 CGLO|KAF3804183.1 CGLO|KAF3805540.1 CGLO|KAF3806255.1 CGLO|KAF3811012.1 CHIG|XP_018151072.1 CHIG|XP_018151233.1 CHIG|XP_018153030.1 CHIG|XP_018155392.1 CHIG|XP_018158223.1 CHIG|XP_018159419.1 CHIG|XP_018160017.1 CHIG|XP_018162085.1 CVIN|KAF4889029.1 CVIN|KAF4918391.1 CVIN|KAF4926899.1 CVIN|KAF4931455.1 CVYL|A05663 CVYL|A09775 FGRM|XP_011323065.1 FGRM|XP_011323139.1 FGRM|XP_011323314.1 FGRM|XP_011324979.1 MGRA|XP_003848251.1 MORY|QBZ57979.1 MORY|QBZ58220.1 NCRA|XP_001728446.2 NCRA|XP_962207.2 SSCL|APA09404.1 SSCL|APA09928.1

>Orthogroup158: ANID|CBF74975.1 ANID|CBF78035.1 ANID|CBF81473.1 BCIN|XP_001554380.1 BGRA|VDB93335.1 CFRU|XP_031878091.1 CFRU|XP_031879411.1 CFRU|XP_031885599.1 CFRU|XP_031885872.1 CFRU|XP_031890882.1 CFRU|XP_031892238.1 CFRU|XP_031892487.1 CGLO|KAF3801160.1 CGLO|KAF3801329.1 CGLO|KAF3801467.1 CGLO|KAF3801851.1 CGLO|KAF3802426.1 CGLO|KAF3809061.1 CHIG|XP_018151898.1 CHIG|XP_018154729.1 CHIG|XP_018155880.1 CHIG|XP_018159338.1 CVIN|KAF4903832.1 CVIN|KAF4910470.1 CVIN|KAF4910760.1 CVIN|KAF4911064.1 CVIN|KAF4918225.1 CVYL|A01270 CVYL|A02650 CVYL|A05686 CVYL|A07758 CVYL|A12656 CVYL|A12855 CVYL|A13570 FGRM|XP_011319150.1 FGRM|XP_011323371.1 MGRA|XP_003851847.1 MLAR|XP_007406976.1 MLAR|XP_007407853.1 MORY|QBZ62463.1 NCRA|XP_011394658.1 SSCL|APA12371.1

>Orthogroup159: ANID|CBF75892.1 ANID|CBF75912.1 ANID|CBF80305.1 ANID|CBF80593.1 ANID|CBF80765.1 ANID|CBF80770.1 ANID|CBF84668.1 CFRU|XP_031875947.1 CFRU|XP_031878854.1 CFRU|XP_031879109.1 CFRU|XP_031882274.1 CFRU|XP_031884557.1 CFRU|XP_031887362.1 CGLO|KAF3797110.1 CGLO|KAF3798801.1 CGLO|KAF3802624.1 CGLO|KAF3803769.1 CGLO|KAF3804471.1 CGLO|KAF3805703.1 CGLO|KAF3809311.1 CHIG|XP_018150655.1 CHIG|XP_018153895.1 CHIG|XP_018158396.1 CHIG|XP_018159693.1 CHIG|XP_018160158.1 CHIG|XP_018163007.1 CHIG|XP_018163150.1 CVIN|KAF4905506.1 CVIN|KAF4917914.1 CVIN|KAF4930991.1 CVYL|A01510 CVYL|A01920 CVYL|A04185 FGRM|XP_011320641.1 FGRM|XP_011328857.1 MGRA|XP_003849340.1 MORY|QBZ55880.1 MORY|QBZ57372.1 MORY|QBZ59469.1 MORY|QBZ62307.1 NCRA|XP_956367.1 NCRA|XP_963915.3

>Orthogroup160: ANID|CBF78453.1 BCIN|XP_001559622.1 CFRU|XP_031878970.1 CFRU|XP_031881249.1 CFRU|XP_031881522.1 CFRU|XP_031891502.1 CFRU|XP_031891891.1 CGLO|KAF3801323.1 CGLO|KAF3802491.1 CGLO|KAF3804651.1 CGLO|KAF3807921.1 CHIG|XP_018154797.1 CHIG|XP_018155859.1 CHIG|XP_018161708.1 CHIG|XP_018163058.1 CHIG|XP_018163636.1 CVIN|KAF4910754.1 CVIN|KAF4913904.1 CVIN|KAF4915242.1 CVIN|KAF4918985.1 CVIN|KAF4926636.1 CVYL|A00698 CVYL|A02083 CVYL|A05234 CVYL|A07763 CVYL|A09828 FGRM|XP_011319385.1 FGRM|XP_011326043.1 MGRA|XP_003850951.1 MGRA|XP_003850952.1 MLAR|XP_007406952.1 MLAR|XP_007414401.1 MLAR|XP_007415271.1 MLAR|XP_007415280.1 MLAR|XP_007415306.1 MORY|QBZ55005.1 MORY|QBZ59911.1 MORY|QBZ65318.1 NCRA|XP_955817.3 NCRA|XP_959279.1 NCRA|XP_960313.1 SSCL|APA09395.1

>Orthogroup161: ANID|CBF84864.1 BCIN|XP_001558848.1 BGRA|VDB93053.1 CFRU|XP_031877343.1 CFRU|XP_031879367.1 CFRU|XP_031880734.1 CFRU|XP_031884369.1 CFRU|XP_031891065.1 CFRU|XP_031891693.1 CGLO|KAF3799082.1 CGLO|KAF3799907.1 CGLO|KAF3805649.1 CGLO|KAF3808596.1 CGLO|KAF3809008.1 CGLO|KAF3811163.1 CGLO|KAF3811661.1 CHIG|XP_018151862.1 CHIG|XP_018153793.1 CHIG|XP_018159483.1 CHIG|XP_018161895.1 CVIN|KAF4892836.1 CVIN|KAF4903982.1 CVIN|KAF4918263.1 CVIN|KAF4925687.1 CVIN|KAF4926201.1 CVIN|KAF4931267.1 CVYL|A04324 CVYL|A07549 CVYL|A09614 CVYL|A11446 CVYL|A11547 CVYL|A12690 FGRM|XP_011322091.1 FGRM|XP_011325629.1 FGRM|XP_011327406.1 FGRM|XP_011327598.1 FGRM|XP_011328028.1 MGRA|XP_003850500.1 MORY|QBZ61927.1 NCRA|XP_958746.1 NCRA|XP_960250.1 SSCL|APA05996.1

>Orthogroup162: ANID|CBF86829.1 ANID|CBF88885.1 BCIN|XP_001548661.1 BCIN|XP_001552206.1 BCIN|XP_001557149.1 BGRA|VDB92799.1 CFRU|XP_031882560.1 CFRU|XP_031886304.1 CFRU|XP_031887678.1 CGLO|KAF3800474.1 CGLO|KAF3806226.1 CGLO|KAF3807176.1 CHIG|XP_018152221.1 CHIG|XP_018161002.1 CHIG|XP_018162061.1 CVIN|KAF4906665.1 CVIN|KAF4917573.1 CVIN|KAF4931528.1 CVYL|A03581 CVYL|A11816 CVYL|A13237 FGRM|XP_011321328.1 FGRM|XP_011323563.1 FGRM|XP_011324036.1 MGRA|XP_003849394.1 MGRA|XP_003851879.1 MLAR|XP_007403633.1 MLAR|XP_007404030.1 MLAR|XP_007407826.1 MLAR|XP_007412644.1 MLAR|XP_007414140.1 MLAR|XP_007417077.1 MLAR|XP_007418703.1 MORY|QBZ58822.1 MORY|QBZ61544.1 MORY|QBZ62008.1 NCRA|XP_956002.2 NCRA|XP_956319.3 NCRA|XP_956597.1 SSCL|APA13001.1 SSCL|APA15031.1 SSCL|APA15588.1

>Orthogroup163: BCIN|XP_024550820.1 BCIN|XP_024550994.1 BCIN|XP_024551123.1 BCIN|XP_024551346.1 BCIN|XP_024553406.1 CFRU|XP_031882147.1 CFRU|XP_031883785.1 CFRU|XP_031886437.1 CFRU|XP_031889507.1 CFRU|XP_031890477.1 CFRU|XP_031891402.1 CFRU|XP_031891613.1 CGLO|KAF3798725.1 CGLO|KAF3805923.1 CGLO|KAF3805925.1 CGLO|KAF3806345.1 CGLO|KAF3811753.1 CVIN|KAF4899317.1 CVIN|KAF4919079.1 CVYL|A05268 CVYL|A11934 CVYL|A12190 CVYL|A13787 FGRM|XP_011323333.1 FGRM|XP_011326458.1 MORY|QBZ57859.1 NCRA|XP_001728118.2 NCRA|XP_001728563.1 NCRA|XP_011393544.1 NCRA|XP_011393658.1 NCRA|XP_011394444.1 NCRA|XP_011394775.1 NCRA|XP_955901.3 NCRA|XP_957101.2 NCRA|XP_958309.2 NCRA|XP_958461.1 NCRA|XP_958571.1 NCRA|XP_960369.1 SSCL|APA11729.1 SSCL|APA11805.1 SSCL|APA13169.1 SSCL|APA14776.1

>Orthogroup164: BGRA|VCU39289.1 BGRA|VCU39307.1 BGRA|VCU39309.1 BGRA|VCU39315.1 BGRA|VCU39332.1 BGRA|VCU39335.1 BGRA|VCU39344.1 BGRA|VCU39347.1 BGRA|VCU39355.1 BGRA|VCU39356.1 BGRA|VCU40921.1 BGRA|VDB86379.1 BGRA|VDB88080.1 BGRA|VDB88084.1 BGRA|VDB88085.1 BGRA|VDB88087.1 BGRA|VDB88088.1 BGRA|VDB88089.1 BGRA|VDB88091.1 BGRA|VDB88093.1 BGRA|VDB88095.1 BGRA|VDB88096.1 BGRA|VDB88097.1 BGRA|VDB88115.1 BGRA|VDB88941.1 BGRA|VDB91050.1 BGRA|VDB91062.1 BGRA|VDB92928.1 BGRA|VDB92937.1 BGRA|VDB93703.1 BGRA|VDB93705.1 BGRA|VDB93706.1 BGRA|VDB93707.1 BGRA|VDB93708.1 BGRA|VDB93724.1 BGRA|VDB93728.1 BGRA|VDB93730.1 BGRA|VDB93732.1 BGRA|VDB93733.1 BGRA|VDB93734.1 BGRA|VDB93735.1 BGRA|VDB93736.1

>Orthogroup165: MLAR|XP_007403593.1 MLAR|XP_007403800.1 MLAR|XP_007404023.1 MLAR|XP_007406041.1 MLAR|XP_007407409.1 MLAR|XP_007407505.1 MLAR|XP_007407854.1 MLAR|XP_007407886.1 MLAR|XP_007408336.1 MLAR|XP_007408671.1 MLAR|XP_007408758.1 MLAR|XP_007409671.1 MLAR|XP_007409904.1 MLAR|XP_007410405.1 MLAR|XP_007410752.1 MLAR|XP_007411199.1 MLAR|XP_007411823.1 MLAR|XP_007412448.1 MLAR|XP_007412583.1 MLAR|XP_007413708.1 MLAR|XP_007413899.1 MLAR|XP_007413900.1 MLAR|XP_007414980.1 MLAR|XP_007415104.1 MLAR|XP_007415368.1 MLAR|XP_007415590.1 MLAR|XP_007415755.1 MLAR|XP_007416123.1 MLAR|XP_007416132.1 MLAR|XP_007416847.1 MLAR|XP_007417243.1 MLAR|XP_007417371.1 MLAR|XP_007417584.1 MLAR|XP_007417641.1 MLAR|XP_007417878.1 MLAR|XP_007417882.1 MLAR|XP_007418459.1 MLAR|XP_007418566.1 MLAR|XP_007419165.1 MLAR|XP_007419166.1 MLAR|XP_007419423.1 MLAR|XP_007419840.1

>Orthogroup166: SSCL|APA05486.1 SSCL|APA05488.1 SSCL|APA05500.1 SSCL|APA05658.1 SSCL|APA05659.1 SSCL|APA06009.1 SSCL|APA06010.1 SSCL|APA06561.1 SSCL|APA06562.1 SSCL|APA07909.1 SSCL|APA07910.1 SSCL|APA08387.1 SSCL|APA08388.1 SSCL|APA08427.1 SSCL|APA08606.1 SSCL|APA08975.1 SSCL|APA09152.1 SSCL|APA09153.1 SSCL|APA09253.1 SSCL|APA09259.1 SSCL|APA09396.1 SSCL|APA10434.1 SSCL|APA10645.1 SSCL|APA10732.1 SSCL|APA10760.1 SSCL|APA10975.1 SSCL|APA11508.1 SSCL|APA11758.1 SSCL|APA12295.1 SSCL|APA12583.1 SSCL|APA13181.1 SSCL|APA13274.1 SSCL|APA13275.1 SSCL|APA14237.1 SSCL|APA14851.1 SSCL|APA14878.1 SSCL|APA14879.1 SSCL|APA15327.1 SSCL|APA15885.1 SSCL|APA15894.1 SSCL|APA16038.1 SSCL|APA16239.1

>Orthogroup167: ANID|CBF70432.1 BCIN|XP_001547305.1 BCIN|XP_001559346.1 BCIN|XP_024546983.1 BGRA|VCU39434.1 CFRU|XP_031879456.1 CFRU|XP_031883570.1 CFRU|XP_031884402.1 CFRU|XP_031887827.1 CFRU|XP_031887855.1 CGLO|KAF3799699.1 CGLO|KAF3802071.1 CGLO|KAF3802384.1 CGLO|KAF3811906.1 CHIG|XP_018154298.1 CHIG|XP_018154699.1 CHIG|XP_018155151.1 CHIG|XP_018157910.1 CHIG|XP_018161179.1 CVIN|KAF4911835.1 CVIN|KAF4917976.1 CVIN|KAF4919986.1 CVIN|KAF4922171.1 CVYL|A03315 CVYL|A05860 CVYL|A06152 CVYL|A13527 FGRM|XP_011317285.1 FGRM|XP_011323158.1 MGRA|XP_003847833.1 MGRA|XP_003855798.1 MLAR|XP_007418810.1 MLAR|XP_007418811.1 MLAR|XP_007418818.1 MLAR|XP_007418821.1 MLAR|XP_007418827.1 MORY|QBZ64846.1 NCRA|XP_001728130.2 NCRA|XP_956467.2 SSCL|APA07879.1 SSCL|APA12536.1

>Orthogroup168: ANID|CBF71440.1 ANID|CBF75862.1 ANID|CBF78998.1 ANID|CBF82052.1 ANID|CBF84899.1 ANID|CBF87348.1 BCIN|XP_024546852.1 CFRU|XP_031882081.1 CFRU|XP_031884634.1 CFRU|XP_031889643.1 CFRU|XP_031893537.1 CGLO|KAF3799739.1 CGLO|KAF3806024.1 CGLO|KAF3808111.1 CGLO|KAF3810512.1 CHIG|XP_018152810.1 CHIG|XP_018159104.1 CVIN|KAF4899563.1 CVIN|KAF4914574.1 CVIN|KAF4917844.1 CVIN|KAF4920608.1 CVYL|A07488 CVYL|A08123 CVYL|A11353 CVYL|A13593 FGRM|XP_011317945.1 FGRM|XP_011321801.1 MGRA|XP_003847810.1 MGRA|XP_003847832.1 MGRA|XP_003848199.1 MGRA|XP_003848735.1 MGRA|XP_003852037.1 MGRA|XP_003853252.1 MGRA|XP_003856605.1 MGRA|XP_003857146.1 MGRA|XP_003857565.1 MORY|QBZ54835.1 MORY|QBZ60888.1 NCRA|XP_011392832.1 NCRA|XP_959497.1 SSCL|APA06034.1

>Orthogroup169: ANID|CBF75107.1 ANID|CBF76688.1 ANID|CBF85354.1 ANID|CBF86773.1 BCIN|XP_001548852.1 BCIN|XP_001559353.1 BCIN|XP_024552293.1 CFRU|XP_031876056.1 CFRU|XP_031879778.1 CFRU|XP_031881011.1 CFRU|XP_031889274.1 CGLO|KAF3798568.1 CGLO|KAF3802000.1 CGLO|KAF3808870.1 CGLO|KAF3809875.1 CHIG|XP_018154138.1 CHIG|XP_018159443.1 CHIG|XP_018161440.1 CVIN|KAF4896683.1 CVIN|KAF4919773.1 CVIN|KAF4930171.1 CVIN|KAF4930719.1 CVYL|A01212 CVYL|A01742 CVYL|A01743 CVYL|A02432 CVYL|A04873 FGRM|XP_011318149.1 FGRM|XP_011319438.1 FGRM|XP_011322494.1 MGRA|XP_003848683.1 MGRA|XP_003848741.1 MGRA|XP_003852623.1 MLAR|XP_007407051.1 MLAR|XP_007414153.1 MLAR|XP_007418576.1 MORY|QBZ59988.1 MORY|QBZ63377.1 SSCL|APA07715.1 SSCL|APA13070.1 SSCL|APA15973.1

>Orthogroup170: ANID|CBF80804.1 BCIN|XP_001547933.1 BCIN|XP_024548774.1 BCIN|XP_024551419.1 CFRU|XP_031879739.1 CFRU|XP_031880727.1 CFRU|XP_031883134.1 CFRU|XP_031888135.1 CFRU|XP_031888619.1 CFRU|XP_031889906.1 CGLO|KAF3798163.1 CGLO|KAF3804192.1 CGLO|KAF3810439.1 CHIG|XP_018159178.1 CHIG|XP_018159482.1 CHIG|XP_018160178.1 CHIG|XP_018163297.1 CVIN|KAF4919114.1 CVIN|KAF4919626.1 CVIN|KAF4924622.1 CVIN|KAF4931266.1 CVYL|A00455 CVYL|A02472 CVYL|A04323 CVYL|A04954 CVYL|A13834 FGRM|XP_011318171.1 FGRM|XP_011325989.1 FGRM|XP_011327277.1 MGRA|XP_003847512.1 MGRA|XP_003852416.1 MGRA|XP_003852611.1 MGRA|XP_003855459.1 MGRA|XP_003857103.1 MGRA|XP_003857653.1 MORY|QBZ64691.1 NCRA|XP_965602.3 SSCL|APA06763.1 SSCL|APA09700.1 SSCL|APA11191.1 SSCL|APA15245.1

>Orthogroup171: ANID|CBF83990.1 BCIN|XP_001560168.1 CFRU|XP_031878756.1 CFRU|XP_031879145.1 CFRU|XP_031880341.1 CFRU|XP_031888919.1 CFRU|XP_031889891.1 CFRU|XP_031890105.1 CFRU|XP_031891466.1 CFRU|XP_031892899.1 CGLO|KAF3797575.1 CGLO|KAF3804292.1 CGLO|KAF3804668.1 CGLO|KAF3805959.1 CGLO|KAF3807325.1 CGLO|KAF3809005.1 CGLO|KAF3809603.1 CGLO|KAF3810408.1 CHIG|XP_018155619.1 CHIG|XP_018161597.1 CVIN|KAF4893136.1 CVIN|KAF4911882.1 CVIN|KAF4913894.1 CVIN|KAF4919210.1 CVIN|KAF4920136.1 CVIN|KAF4920247.1 CVIN|KAF4923584.1 CVYL|A03977 CVYL|A05215 CVYL|A06358 CVYL|A08439 CVYL|A08969 CVYL|A10811 CVYL|A13762 MGRA|XP_003853950.1 MLAR|XP_007417241.1 MLAR|XP_007417242.1 MORY|QBZ58466.1 MORY|QBZ60130.1 MORY|QBZ63454.1 MORY|QBZ66537.1

>Orthogroup172: ANID|CBF85628.1 BCIN|XP_001557038.1 BCIN|XP_024546790.1 BGRA|VDB88312.1 CFRU|XP_031878585.1 CFRU|XP_031879222.1 CFRU|XP_031880099.1 CFRU|XP_031882859.1 CFRU|XP_031887289.1 CFRU|XP_031893371.1 CGLO|KAF3797719.1 CGLO|KAF3803340.1 CGLO|KAF3806656.1 CGLO|KAF3807072.1 CGLO|KAF3809326.1 CGLO|KAF3809683.1 CHIG|XP_018159742.1 CVIN|KAF4892841.1 CVIN|KAF4917541.1 CVIN|KAF4919027.1 CVIN|KAF4919263.1 CVIN|KAF4923684.1 CVIN|KAF4924192.1 CVIN|KAF4925001.1 CVYL|A01528 CVYL|A03781 CVYL|A06366 CVYL|A09587 CVYL|A11433 CVYL|A12686 CVYL|A14206 FGRM|XP_011321860.1 FGRM|XP_011322359.1 FGRM|XP_011328416.1 MGRA|XP_003848599.1 MGRA|XP_003851948.1 MORY|QBZ60242.1 MORY|QBZ61444.1 MORY|QBZ65197.1 SSCL|APA06100.1 SSCL|APA08497.1

>Orthogroup173: CFRU|XP_031876903.1 CFRU|XP_031878172.1 CFRU|XP_031878868.1 CFRU|XP_031881758.1 CFRU|XP_031881787.1 CFRU|XP_031883195.1 CFRU|XP_031884569.1 CFRU|XP_031887813.1 CFRU|XP_031891833.1 CFRU|XP_031893484.1 CGLO|KAF3799821.1 CGLO|KAF3800702.1 CGLO|KAF3801819.1 CGLO|KAF3802047.1 CGLO|KAF3802591.1 CGLO|KAF3808261.1 CGLO|KAF3809781.1 CGLO|KAF3810345.1 CHIG|XP_018152942.1 CHIG|XP_018153799.1 CHIG|XP_018153836.1 CHIG|XP_018153837.1 CHIG|XP_018157632.1 CHIG|XP_018158790.1 CVIN|KAF4899864.1 CVIN|KAF4900283.1 CVIN|KAF4900998.1 CVIN|KAF4901928.1 CVIN|KAF4907995.1 CVIN|KAF4915175.1 CVIN|KAF4916987.1 CVYL|A00597 CVYL|A03341 CVYL|A06702 CVYL|A11988 CVYL|A12099 CVYL|A12888 CVYL|A13017 FGRM|XP_011325733.1 FGRM|XP_011325968.1 FGRM|XP_011326072.1

>Orthogroup174: MLAR|XP_007405215.1 MLAR|XP_007406192.1 MLAR|XP_007406429.1 MLAR|XP_007407191.1 MLAR|XP_007407271.1 MLAR|XP_007407309.1 MLAR|XP_007407601.1 MLAR|XP_007407606.1 MLAR|XP_007407682.1 MLAR|XP_007408018.1 MLAR|XP_007408346.1 MLAR|XP_007408577.1 MLAR|XP_007408873.1 MLAR|XP_007409197.1 MLAR|XP_007409292.1 MLAR|XP_007409295.1 MLAR|XP_007409332.1 MLAR|XP_007409395.1 MLAR|XP_007409490.1 MLAR|XP_007409679.1 MLAR|XP_007410167.1 MLAR|XP_007410238.1 MLAR|XP_007410730.1 MLAR|XP_007412051.1 MLAR|XP_007412190.1 MLAR|XP_007412244.1 MLAR|XP_007412251.1 MLAR|XP_007414029.1 MLAR|XP_007414663.1 MLAR|XP_007414935.1 MLAR|XP_007415399.1 MLAR|XP_007415596.1 MLAR|XP_007415751.1 MLAR|XP_007415865.1 MLAR|XP_007416247.1 MLAR|XP_007416602.1 MLAR|XP_007416879.1 MLAR|XP_007417447.1 MLAR|XP_007417517.1 MLAR|XP_007417714.1 MLAR|XP_007419756.1

>Orthogroup175: ANID|CBF69410.1 ANID|CBF78585.1 ANID|CBF83147.1 BCIN|XP_024550293.1 BCIN|XP_024552754.1 CFRU|XP_031876065.1 CFRU|XP_031877315.1 CFRU|XP_031878892.1 CFRU|XP_031880010.1 CFRU|XP_031884605.1 CFRU|XP_031887338.1 CFRU|XP_031887955.1 CGLO|KAF3798952.1 CGLO|KAF3799749.1 CGLO|KAF3799908.1 CGLO|KAF3801980.1 CGLO|KAF3802112.1 CGLO|KAF3807825.1 CGLO|KAF3809377.1 CHIG|XP_018151718.1 CHIG|XP_018152862.1 CHIG|XP_018153090.1 CHIG|XP_018161977.1 CVIN|KAF4896773.1 CVIN|KAF4903975.1 CVIN|KAF4912434.1 CVIN|KAF4921503.1 CVIN|KAF4923501.1 CVYL|A00792 CVYL|A02985 CVYL|A03281 CVYL|A07868 FGRM|XP_011318274.1 FGRM|XP_011321154.1 FGRM|XP_011324361.1 FGRM|XP_011327532.1 MGRA|XP_003853396.1 MGRA|XP_003856912.1 MORY|QBZ58638.1 SSCL|APA07496.1

>Orthogroup176: ANID|CBF71034.1 ANID|CBF75022.1 ANID|CBF77968.1 ANID|CBF84719.1 BCIN|XP_001558483.1 BCIN|XP_001561340.1 BGRA|VDB89294.1 CFRU|XP_031878128.1 CFRU|XP_031880696.1 CFRU|XP_031881474.1 CGLO|KAF3801823.1 CGLO|KAF3805821.1 CGLO|KAF3809967.1 CHIG|XP_018159330.1 CHIG|XP_018161550.1 CHIG|XP_018163707.1 CVIN|KAF4890772.1 CVIN|KAF4914189.1 CVIN|KAF4920896.1 CVYL|A01655 CVYL|A06002 CVYL|A12884 FGRM|XP_011319717.1 FGRM|XP_011320482.1 FGRM|XP_011322936.1 FGRM|XP_011327148.1 MGRA|XP_003850101.1 MGRA|XP_003850938.1 MGRA|XP_003857636.1 MLAR|XP_007413687.1 MLAR|XP_007415502.1 MLAR|XP_007415576.1 MLAR|XP_007417814.1 MLAR|XP_007417816.1 MORY|QBZ62863.1 MORY|QBZ65416.1 NCRA|XP_958070.1 NCRA|XP_965187.1 SSCL|APA06873.1 SSCL|APA14709.1

>Orthogroup177: ANID|CBF71247.1 ANID|CBF71666.1 ANID|CBF86285.1 BCIN|XP_001553961.1 BCIN|XP_001557997.1 BGRA|VDB92720.1 CFRU|XP_031880623.1 CFRU|XP_031884478.1 CFRU|XP_031889589.1 CGLO|KAF3799057.1 CGLO|KAF3805216.1 CGLO|KAF3809939.1 CHIG|XP_018158197.1 CHIG|XP_018158852.1 CHIG|XP_018161518.1 CVIN|KAF4907183.1 CVIN|KAF4918275.1 CVIN|KAF4930658.1 CVYL|A01684 CVYL|A11471 CVYL|A13863 FGRM|XP_011322287.1 FGRM|XP_011325577.1 FGRM|XP_011326110.1 FGRM|XP_011327592.1 MGRA|XP_003849461.1 MGRA|XP_003854918.1 MGRA|XP_003855909.1 MORY|QBZ53946.1 MORY|QBZ55090.1 MORY|QBZ65139.1 MORY|QBZ65830.1 NCRA|XP_959046.3 NCRA|XP_959401.1 NCRA|XP_963208.2 NCRA|XP_963600.1 NCRA|XP_963904.2 NCRA|XP_964015.1 SSCL|APA11084.1 SSCL|APA12701.1

>Orthogroup178: ANID|CBF71556.1 BCIN|XP_001546018.1 BCIN|XP_024546674.1 BCIN|XP_024550613.1 CFRU|XP_031879285.1 CFRU|XP_031881139.1 CFRU|XP_031881193.1 CFRU|XP_031882598.1 CFRU|XP_031888869.1 CFRU|XP_031889460.1 CGLO|KAF3803926.1 CGLO|KAF3808566.1 CGLO|KAF3809869.1 CGLO|KAF3811000.1 CHIG|XP_018151024.1 CHIG|XP_018151612.1 CHIG|XP_018151613.1 CHIG|XP_018154868.1 CHIG|XP_018161451.1 CVIN|KAF4906487.1 CVIN|KAF4921845.1 CVIN|KAF4924779.1 CVIN|KAF4925476.1 CVIN|KAF4928324.1 CVIN|KAF4930707.1 CVYL|A01749 CVYL|A02154 CVYL|A04131 CVYL|A08876 CVYL|A11579 FGRM|XP_011317834.1 FGRM|XP_011327379.1 MGRA|XP_003850751.1 MGRA|XP_003854032.1 MORY|QBZ54925.1 MORY|QBZ59998.1 MORY|QBZ64222.1 MORY|QBZ64254.1 NCRA|XP_961848.1 SSCL|APA11427.1

>Orthogroup179: ANID|CBF73574.1 BCIN|XP_001550282.2 BCIN|XP_024545917.1 BCIN|XP_024552263.1 BCIN|XP_024553284.1 BCIN|XP_024553456.1 BGRA|VCU39182.1 BGRA|VDB83880.1 CFRU|XP_031890172.1 CFRU|XP_031892756.1 CGLO|KAF3797198.1 CGLO|KAF3802891.1 CHIG|XP_018157319.1 CHIG|XP_018158502.1 CVIN|KAF4913033.1 CVIN|KAF4920828.1 CVYL|A05122 CVYL|A10500 FGRM|XP_011316580.1 FGRM|XP_011319942.1 FGRM|XP_011322233.1 MGRA|XP_003847663.1 MGRA|XP_003848541.1 MGRA|XP_003849136.1 MGRA|XP_003849296.1 MGRA|XP_003852302.1 MGRA|XP_003857657.1 MLAR|XP_007404555.1 MLAR|XP_007406412.1 MORY|QBZ55979.1 MORY|QBZ59918.1 MORY|QBZ66114.1 NCRA|XP_964570.2 NCRA|XP_964765.1 SSCL|APA07336.1 SSCL|APA09653.1 SSCL|APA12845.1 SSCL|APA13114.1 SSCL|APA13550.1 SSCL|APA13835.1

>Orthogroup180: ANID|CBF73652.1 BCIN|XP_001550809.2 BCIN|XP_001558524.1 BCIN|XP_024552552.1 BGRA|VCU39339.1 CFRU|XP_031879039.1 CFRU|XP_031886254.1 CFRU|XP_031889848.1 CFRU|XP_031893488.1 CGLO|KAF3798797.1 CGLO|KAF3799068.1 CGLO|KAF3806212.1 CGLO|KAF3808238.1 CGLO|KAF3808333.1 CHIG|XP_018153970.1 CHIG|XP_018154252.1 CHIG|XP_018159002.1 CHIG|XP_018162204.1 CVIN|KAF4909556.1 CVIN|KAF4919596.1 CVIN|KAF4931569.1 CVYL|A00577 CVYL|A08654 CVYL|A09129 CVYL|A11799 FGRM|XP_011316221.1 FGRM|XP_011319591.1 FGRM|XP_011323213.1 FGRM|XP_011324142.1 FGRM|XP_011326025.1 FGRM|XP_011327642.1 MGRA|XP_003850975.1 MGRA|XP_003857799.1 MORY|QBZ63152.1 MORY|QBZ63773.1 NCRA|XP_011394994.1 NCRA|XP_957314.2 NCRA|XP_957758.1 SSCL|APA08649.1 SSCL|APA14680.1

>Orthogroup181: ANID|CBF74017.1 ANID|CBF89994.1 BCIN|XP_024551825.1 CFRU|XP_031882815.1 CFRU|XP_031891096.1 CFRU|XP_031891473.1 CFRU|XP_031891575.1 CFRU|XP_031892765.1 CFRU|XP_031893369.1 CGLO|KAF3797078.1 CGLO|KAF3797628.1 CGLO|KAF3804502.1 CGLO|KAF3808268.1 CGLO|KAF3809682.1 CGLO|KAF3811756.1 CGLO|KAF3812051.1 CHIG|XP_018150878.1 CHIG|XP_018152502.1 CHIG|XP_018153798.1 CHIG|XP_018157073.1 CHIG|XP_018157074.1 CHIG|XP_018157420.1 CHIG|XP_018159809.1 CHIG|XP_018160330.1 CHIG|XP_018165009.1 CVIN|KAF4890323.1 CVIN|KAF4911377.1 CVIN|KAF4924513.1 CVIN|KAF4925003.1 CVYL|A04217 CVYL|A09588 CVYL|A10631 CVYL|A11072 FGRM|XP_011323026.1 FGRM|XP_011323723.1 FGRM|XP_011325123.1 FGRM|XP_011325976.1 MORY|QBZ61822.1 MORY|QBZ66344.1 NCRA|XP_011393289.1

>Orthogroup182: ANID|CBF74873.1 ANID|CBF84828.1 ANID|CBF88582.1 BCIN|XP_001550696.1 BCIN|XP_024547860.1 BCIN|XP_024549393.1 BCIN|XP_024550023.1 BGRA|VDB92815.1 BGRA|VDB93307.1 BGRA|VDB93308.1 BGRA|VDB94018.1 CFRU|XP_031886174.1 CFRU|XP_031887740.1 CFRU|XP_031890425.1 CGLO|KAF3805274.1 CGLO|KAF3806565.1 CGLO|KAF3807128.1 CHIG|XP_018157605.1 CHIG|XP_018158798.1 CHIG|XP_018160946.1 CVIN|KAF4901296.1 CVIN|KAF4920176.1 CVIN|KAF4920457.1 CVYL|A09304 CVYL|A13925 FGRM|XP_011316665.1 FGRM|XP_011323617.1 FGRM|XP_011327884.1 MGRA|XP_003855499.1 MGRA|XP_003857796.1 MORY|QBZ56296.1 MORY|QBZ58912.1 MORY|QBZ60401.1 NCRA|XP_011394173.1 NCRA|XP_960558.2 NCRA|XP_962310.3 NCRA|XP_964966.1 SSCL|APA05364.1 SSCL|APA09056.1 SSCL|APA09313.1

>Orthogroup183: ANID|CBF76544.1 ANID|CBF76546.1 ANID|CBF80456.1 BCIN|XP_001550574.1 BCIN|XP_024547230.1 BGRA|VDB89054.1 CFRU|XP_031881937.1 CFRU|XP_031881940.1 CFRU|XP_031882315.1 CFRU|XP_031884162.1 CFRU|XP_031884163.1 CFRU|XP_031888562.1 CGLO|KAF3797814.1 CGLO|KAF3797870.1 CGLO|KAF3804399.1 CHIG|XP_018160207.1 CHIG|XP_018164482.1 CHIG|XP_018164483.1 CVIN|KAF4911593.1 CVIN|KAF4911594.1 CVIN|KAF4923175.1 CVIN|KAF4930953.1 CVYL|A03044 CVYL|A04095 CVYL|A07332 FGRM|XP_011322768.1 FGRM|XP_011325750.1 FGRM|XP_011327849.1 FGRM|XP_011327850.1 MGRA|XP_003853362.1 MLAR|XP_007405684.1 MLAR|XP_007409270.1 MLAR|XP_007409798.1 MLAR|XP_007409799.1 MORY|QBZ56757.1 MORY|QBZ59002.1 NCRA|XP_959705.1 NCRA|XP_959717.3 SSCL|APA14162.1 SSCL|APA14163.1

>Orthogroup184: ANID|CBF78813.1 ANID|CBF79656.1 ANID|CBF80359.1 ANID|CBF87163.1 BCIN|XP_024549506.1 BCIN|XP_024550553.1 CFRU|XP_031880816.1 CFRU|XP_031883758.1 CFRU|XP_031884568.1 CFRU|XP_031886164.1 CFRU|XP_031892179.1 CFRU|XP_031893162.1 CGLO|KAF3797468.1 CGLO|KAF3809467.1 CGLO|KAF3810347.1 CGLO|KAF3812004.1 CHIG|XP_018152706.1 CHIG|XP_018152707.1 CHIG|XP_018155589.1 CHIG|XP_018156644.1 CHIG|XP_018159674.1 CVIN|KAF4899865.1 CVIN|KAF4918787.1 CVIN|KAF4921286.1 CVIN|KAF4930440.1 CVYL|A05504 CVYL|A05761 CVYL|A06288 CVYL|A10035 FGRM|XP_011327415.1 MGRA|XP_003848905.1 MGRA|XP_003851328.1 MGRA|XP_003854702.1 MORY|QBZ61774.1 MORY|QBZ61977.1 MORY|QBZ64091.1 NCRA|XP_958188.1 NCRA|XP_960204.1 NCRA|XP_965181.1 SSCL|APA09134.1

>Orthogroup185: ANID|CBF79949.1 ANID|CBF83986.1 ANID|CBF87008.1 BCIN|XP_001547805.1 BCIN|XP_024551448.1 BCIN|XP_024552641.1 BGRA|VCU39966.1 CFRU|XP_031877793.1 CFRU|XP_031883224.1 CFRU|XP_031886194.1 CFRU|XP_031891363.1 CGLO|KAF3797918.1 CGLO|KAF3805536.1 CGLO|KAF3808172.1 CHIG|XP_018155386.1 CHIG|XP_018161602.1 CHIG|XP_018162443.1 CHIG|XP_018163243.1 CVIN|KAF4907836.1 CVIN|KAF4916963.1 CVIN|KAF4918305.1 CVIN|KAF4924302.1 CVYL|A00525 CVYL|A05353 CVYL|A05659 CVYL|A07276 FGRM|XP_011318657.1 FGRM|XP_011321933.1 FGRM|XP_011322495.1 FGRM|XP_011327017.1 MGRA|XP_003848195.1 MGRA|XP_003851171.1 MGRA|XP_003857750.1 MLAR|XP_007406400.1 MORY|QBZ64058.1 NCRA|XP_958514.3 NCRA|XP_961599.1 SSCL|APA10713.1 SSCL|APA15439.1 SSCL|APA15593.1

>Orthogroup186: ANID|CBF83739.1 ANID|CBF86106.1 BCIN|XP_001547269.1 BCIN|XP_001550617.2 BCIN|XP_001553665.1 BCIN|XP_001556605.2 BCIN|XP_001558652.1 BCIN|XP_001559355.1 BCIN|XP_024549197.1 BCIN|XP_024550754.1 BCIN|XP_024551995.1 BCIN|XP_024552301.1 BCIN|XP_024552535.1 BCIN|XP_024553171.1 BCIN|XP_024553338.1 BCIN|XP_024554070.1 CFRU|XP_031881138.1 CFRU|XP_031891334.1 CGLO|KAF3797398.1 CGLO|KAF3809870.1 CHIG|XP_018151643.1 CHIG|XP_018156609.1 CHIG|XP_018157358.1 CHIG|XP_018161450.1 CVIN|KAF4929412.1 CVIN|KAF4930706.1 CVYL|A01748 CVYL|A10281 FGRM|XP_011319622.1 MGRA|XP_003848603.1 MORY|QBZ55035.1 MORY|QBZ62935.1 NCRA|XP_955820.1 SSCL|APA05654.1 SSCL|APA06277.1 SSCL|APA07424.1 SSCL|APA07713.1 SSCL|APA08871.1 SSCL|APA11657.1 SSCL|APA15728.1

>Orthogroup187: ANID|CBF86738.1 ANID|CBF87130.1 BCIN|XP_024546597.1 BCIN|XP_024547447.1 CFRU|XP_031878864.1 CFRU|XP_031879296.1 CFRU|XP_031880047.1 CFRU|XP_031884194.1 CFRU|XP_031892841.1 CGLO|KAF3802572.1 CGLO|KAF3807216.1 CGLO|KAF3808577.1 CGLO|KAF3811025.1 CGLO|KAF3811831.1 CHIG|XP_018151521.1 CHIG|XP_018152989.1 CHIG|XP_018153885.1 CHIG|XP_018157022.1 CHIG|XP_018161914.1 CVIN|KAF4905055.1 CVIN|KAF4917608.1 CVIN|KAF4924906.1 CVIN|KAF4928161.1 CVYL|A01860 CVYL|A10699 CVYL|A12832 FGRM|XP_011315701.1 FGRM|XP_011320368.1 FGRM|XP_011322251.1 FGRM|XP_011322780.1 FGRM|XP_011323171.1 FGRM|XP_011327264.1 MORY|QBZ57625.1 MORY|QBZ58611.1 MORY|QBZ59584.1 MORY|QBZ64214.1 MORY|QBZ64330.1 MORY|QBZ66600.1 MORY|QBZ66686.1 SSCL|APA06424.1

>Orthogroup188: ANID|CBF87159.1 BCIN|XP_024547716.1 BCIN|XP_024548382.1 BCIN|XP_024552567.1 CFRU|XP_031883033.1 CFRU|XP_031885774.1 CFRU|XP_031886380.1 CFRU|XP_031889396.1 CFRU|XP_031892109.1 CGLO|KAF3800060.1 CGLO|KAF3806368.1 CGLO|KAF3808181.1 CGLO|KAF3810060.1 CHIG|XP_018152565.1 CHIG|XP_018162224.1 CHIG|XP_018163228.1 CVIN|KAF4898728.1 CVIN|KAF4914519.1 CVIN|KAF4919342.1 CVIN|KAF4923243.1 CVIN|KAF4924296.1 CVYL|A00535 CVYL|A02651 CVYL|A06759 CVYL|A11954 CVYL|A11956 FGRM|XP_011315813.1 FGRM|XP_011321410.1 FGRM|XP_011322250.1 FGRM|XP_011328567.1 MGRA|XP_003850153.1 MLAR|XP_007408480.1 MLAR|XP_007408625.1 MLAR|XP_007408626.1 MLAR|XP_007409647.1 MLAR|XP_007409676.1 MLAR|XP_007410310.1 MLAR|XP_007415308.1 NCRA|XP_957769.1 SSCL|APA08673.1

>Orthogroup189: MLAR|XP_007403522.1 MLAR|XP_007404253.1 MLAR|XP_007404328.1 MLAR|XP_007405688.1 MLAR|XP_007405896.1 MLAR|XP_007407139.1 MLAR|XP_007407232.1 MLAR|XP_007407402.1 MLAR|XP_007407771.1 MLAR|XP_007407870.1 MLAR|XP_007407948.1 MLAR|XP_007408199.1 MLAR|XP_007408683.1 MLAR|XP_007408848.1 MLAR|XP_007408907.1 MLAR|XP_007409131.1 MLAR|XP_007409289.1 MLAR|XP_007409304.1 MLAR|XP_007409587.1 MLAR|XP_007411115.1 MLAR|XP_007411518.1 MLAR|XP_007412040.1 MLAR|XP_007412199.1 MLAR|XP_007412539.1 MLAR|XP_007413176.1 MLAR|XP_007413839.1 MLAR|XP_007413965.1 MLAR|XP_007414295.1 MLAR|XP_007414383.1 MLAR|XP_007415947.1 MLAR|XP_007416148.1 MLAR|XP_007416567.1 MLAR|XP_007416725.1 MLAR|XP_007418182.1 MLAR|XP_007418299.1 MLAR|XP_007418654.1 MLAR|XP_007418656.1 MLAR|XP_007418764.1 MLAR|XP_007419311.1 MLAR|XP_007419495.1

>Orthogroup190: MLAR|XP_007403582.1 MLAR|XP_007403775.1 MLAR|XP_007403776.1 MLAR|XP_007404540.1 MLAR|XP_007404825.1 MLAR|XP_007404965.1 MLAR|XP_007405057.1 MLAR|XP_007405200.1 MLAR|XP_007405371.1 MLAR|XP_007405648.1 MLAR|XP_007405868.1 MLAR|XP_007407366.1 MLAR|XP_007407503.1 MLAR|XP_007407589.1 MLAR|XP_007407901.1 MLAR|XP_007408079.1 MLAR|XP_007408352.1 MLAR|XP_007410147.1 MLAR|XP_007410780.1 MLAR|XP_007411009.1 MLAR|XP_007411091.1 MLAR|XP_007411184.1 MLAR|XP_007412475.1 MLAR|XP_007412567.1 MLAR|XP_007413673.1 MLAR|XP_007413927.1 MLAR|XP_007414147.1 MLAR|XP_007414244.1 MLAR|XP_007414250.1 MLAR|XP_007414571.1 MLAR|XP_007414977.1 MLAR|XP_007415690.1 MLAR|XP_007415695.1 MLAR|XP_007416004.1 MLAR|XP_007417111.1 MLAR|XP_007418461.1 MLAR|XP_007418462.1 MLAR|XP_007418529.1 MLAR|XP_007419057.1 MLAR|XP_007419084.1

>Orthogroup191: ANID|CBF69701.1 ANID|CBF77369.1 ANID|CBF82779.1 ANID|CBF82808.1 ANID|CBF82987.1 ANID|CBF86021.1 BCIN|XP_001553411.1 BCIN|XP_001554815.1 BCIN|XP_001560882.2 BCIN|XP_024546897.1 BCIN|XP_024548497.1 CFRU|XP_031875920.1 CFRU|XP_031882923.1 CFRU|XP_031885766.1 CGLO|KAF3803401.1 CGLO|KAF3807805.1 CGLO|KAF3810080.1 CHIG|XP_018157917.1 CHIG|XP_018158226.1 CHIG|XP_018163931.1 CVIN|KAF4912743.1 CVIN|KAF4918417.1 CVIN|KAF4921523.1 CVYL|A00799 CVYL|A02676 CVYL|A14331 FGRM|XP_011321841.1 MGRA|XP_003851257.1 MGRA|XP_003854395.1 MGRA|XP_003857587.1 MORY|QBZ65490.1 MORY|QBZ66476.1 NCRA|XP_959674.1 NCRA|XP_964065.2 SSCL|APA05843.1 SSCL|APA08091.1 SSCL|APA10927.1 SSCL|APA12148.1 SSCL|APA15521.1

>Orthogroup192: ANID|CBF70198.1 BCIN|XP_024553208.1 CFRU|XP_031880974.1 CFRU|XP_031883449.1 CFRU|XP_031890292.1 CFRU|XP_031890892.1 CFRU|XP_031891854.1 CGLO|KAF3801491.1 CGLO|KAF3801739.1 CGLO|KAF3802862.1 CGLO|KAF3809808.1 CGLO|KAF3809901.1 CHIG|XP_018151923.1 CHIG|XP_018158455.1 CHIG|XP_018159253.1 CHIG|XP_018161488.1 CHIG|XP_018164948.1 CVIN|KAF4899967.1 CVIN|KAF4916385.1 CVIN|KAF4919313.1 CVIN|KAF4922929.1 CVIN|KAF4930645.1 CVYL|A01717 CVYL|A05156 CVYL|A06727 CVYL|A12638 CVYL|A12963 FGRM|XP_011321808.1 FGRM|XP_011325310.1 FGRM|XP_011325796.1 MGRA|XP_003848300.1 MORY|QBZ57428.1 MORY|QBZ63751.1 MORY|QBZ64986.1 NCRA|XP_957063.1 NCRA|XP_959127.1 NCRA|XP_963215.1 NCRA|XP_964544.2 SSCL|APA13474.1

>Orthogroup193: ANID|CBF70334.1 ANID|CBF85455.1 ANID|CBF87436.1 BCIN|XP_001548443.2 BCIN|XP_024551991.1 BGRA|VCU39945.1 CFRU|XP_031877152.1 CFRU|XP_031877977.1 CFRU|XP_031880639.1 CFRU|XP_031884505.1 CFRU|XP_031886504.1 CFRU|XP_031892063.1 CGLO|KAF3799063.1 CGLO|KAF3801295.1 CGLO|KAF3803971.1 CGLO|KAF3806329.1 CGLO|KAF3811195.1 CHIG|XP_018150971.1 CHIG|XP_018154927.1 CHIG|XP_018158192.1 CHIG|XP_018164850.1 CVIN|KAF4916370.1 CVIN|KAF4918269.1 CVIN|KAF4921561.1 CVIN|KAF4923933.1 CVIN|KAF4925141.1 CVIN|KAF4931251.1 CVYL|A04359 CVYL|A07792 CVYL|A08830 CVYL|A11464 CVYL|A11917 FGRM|XP_011318215.1 FGRM|XP_011322703.1 FGRM|XP_011323353.1 MORY|QBZ64446.1 NCRA|XP_965618.1 SSCL|APA09292.1 SSCL|APA12665.1

>Orthogroup194: ANID|CBF71463.1 ANID|CBF73486.1 ANID|CBF82421.1 ANID|CBF86045.1 ANID|CBF88298.1 ANID|CBF89301.1 BCIN|XP_001560980.1 BCIN|XP_024547561.1 BGRA|VDB92936.1 CFRU|XP_031878852.1 CFRU|XP_031883564.1 CGLO|KAF3802577.1 CGLO|KAF3806756.1 CGLO|KAF3809741.1 CGLO|KAF3810795.1 CHIG|XP_018151570.1 CHIG|XP_018155051.1 CHIG|XP_018155077.1 CHIG|XP_018156681.1 CHIG|XP_018161333.1 CVIN|KAF4917597.1 CVIN|KAF4919514.1 CVIN|KAF4921785.1 CVYL|A01866 CVYL|A02025 FGRM|XP_011317531.1 FGRM|XP_011319442.1 FGRM|XP_011327621.1 MGRA|XP_003848567.1 MORY|QBZ55113.1 MORY|QBZ57676.1 MORY|QBZ57677.1 MORY|QBZ60723.1 MORY|QBZ60762.1 MORY|QBZ65385.1 NCRA|XP_959323.1 SSCL|APA06702.1 SSCL|APA09954.1 SSCL|APA15018.1

>Orthogroup195: ANID|CBF73605.1 ANID|CBF75008.1 ANID|CBF80285.1 ANID|CBF82799.1 BCIN|XP_024552378.1 CFRU|XP_031879429.1 CFRU|XP_031879883.1 CFRU|XP_031883187.1 CFRU|XP_031884869.1 CFRU|XP_031888039.1 CFRU|XP_031889622.1 CGLO|KAF3798940.1 CGLO|KAF3800713.1 CGLO|KAF3802046.1 CGLO|KAF3802439.1 CGLO|KAF3810551.1 CGLO|KAF3812173.1 CHIG|XP_018152866.1 CHIG|XP_018153397.1 CHIG|XP_018154738.1 CHIG|XP_018163266.1 CVIN|KAF4898132.1 CVIN|KAF4899754.1 CVIN|KAF4910495.1 CVIN|KAF4913909.1 CVIN|KAF4918754.1 CVIN|KAF4919729.1 CVYL|A00217 CVYL|A00490 CVYL|A03342 CVYL|A07857 CVYL|A13582 CVYL|A13627 FGRM|XP_011320504.1 MGRA|XP_003854453.1 MORY|QBZ65657.1 MORY|QBZ66386.1 NCRA|XP_956060.1 SSCL|APA13211.1

>Orthogroup196: ANID|CBF74060.1 ANID|CBF76157.1 ANID|CBF88175.1 BCIN|XP_024548187.1 BCIN|XP_024553148.1 CFRU|XP_031876080.1 CFRU|XP_031880730.1 CFRU|XP_031881133.1 CFRU|XP_031882711.1 CFRU|XP_031889006.1 CFRU|XP_031891618.1 CGLO|KAF3797932.1 CGLO|KAF3801999.1 CGLO|KAF3803350.1 CGLO|KAF3809898.1 CGLO|KAF3811160.1 CHIG|XP_018154199.1 CHIG|XP_018159439.1 CHIG|XP_018159440.1 CHIG|XP_018160349.1 CHIG|XP_018160350.1 CVIN|KAF4907831.1 CVIN|KAF4915967.1 CVIN|KAF4919762.1 CVIN|KAF4930643.1 CVIN|KAF4931271.1 CVYL|A01719 CVYL|A04320 CVYL|A05366 CVYL|A14276 FGRM|XP_011320552.1 FGRM|XP_011321734.1 FGRM|XP_011323269.1 MGRA|XP_003853603.1 MGRA|XP_003853911.1 MORY|QBZ58019.1 MORY|QBZ60389.1 NCRA|XP_961194.1 SSCL|APA13327.1

>Orthogroup197: ANID|CBF74189.1 ANID|CBF79204.1 ANID|CBF82591.1 BCIN|XP_001548641.1 BCIN|XP_024546939.1 BCIN|XP_024548867.1 BGRA|VDB84107.1 CFRU|XP_031880932.1 CFRU|XP_031882036.1 CFRU|XP_031883503.1 CFRU|XP_031890419.1 CGLO|KAF3804800.1 CGLO|KAF3806090.1 CGLO|KAF3806913.1 CGLO|KAF3811920.1 CHIG|XP_018151874.1 CHIG|XP_018159595.1 CHIG|XP_018164294.1 CHIG|XP_018164582.1 CVIN|KAF4890282.1 CVIN|KAF4895625.1 CVIN|KAF4909962.1 CVIN|KAF4929756.1 CVYL|A02556 CVYL|A07422 CVYL|A12271 FGRM|XP_011316299.1 FGRM|XP_011319532.1 FGRM|XP_011325421.1 FGRM|XP_011325761.1 MGRA|XP_003854552.1 MLAR|XP_007415377.1 MORY|QBZ55174.1 MORY|QBZ55351.1 NCRA|XP_959923.2 NCRA|XP_963925.2 SSCL|APA05801.1 SSCL|APA09432.1 SSCL|APA09635.1

>Orthogroup198: ANID|CBF75140.1 ANID|CBF83972.1 BCIN|XP_001552812.2 BCIN|XP_024546743.1 BCIN|XP_024547146.1 BCIN|XP_024547425.1 BCIN|XP_024549186.1 BCIN|XP_024550038.1 BCIN|XP_024553033.1 BCIN|XP_024553593.1 CFRU|XP_031877339.1 CFRU|XP_031878259.1 CFRU|XP_031879999.1 CFRU|XP_031880851.1 CFRU|XP_031884200.1 CFRU|XP_031888644.1 CGLO|KAF3798071.1 CGLO|KAF3798921.1 CGLO|KAF3800006.1 CGLO|KAF3805491.1 CGLO|KAF3806897.1 CHIG|XP_018152523.1 CHIG|XP_018153044.1 CHIG|XP_018155350.1 CVIN|KAF4904494.1 CVIN|KAF4913934.1 CVIN|KAF4924808.1 CVIN|KAF4926861.1 CVIN|KAF4928001.1 CVYL|A02542 CVYL|A07841 CVYL|A11275 FGRM|XP_011317516.1 FGRM|XP_011320511.1 FGRM|XP_011325341.1 MORY|QBZ63723.1 SSCL|APA06209.1 SSCL|APA06404.1 SSCL|APA08971.1

>Orthogroup199: ANID|CBF75442.1 ANID|CBF81984.1 ANID|CBF87954.1 BCIN|XP_001560564.1 BCIN|XP_024549086.1 BCIN|XP_024549825.1 BGRA|VDB88103.1 CFRU|XP_031883525.1 CFRU|XP_031890703.1 CFRU|XP_031890849.1 CFRU|XP_031892615.1 CGLO|KAF3797127.1 CGLO|KAF3801622.1 CGLO|KAF3810837.1 CHIG|XP_018151355.1 CHIG|XP_018155031.1 CVIN|KAF4896915.1 CVIN|KAF4911114.1 CVIN|KAF4922682.1 CVIN|KAF4922699.1 CVYL|A02302 CVYL|A10573 CVYL|A12514 CVYL|A12541 MGRA|XP_003847614.1 MGRA|XP_003848166.1 MGRA|XP_003849056.1 MGRA|XP_003849733.1 MGRA|XP_003850025.1 MLAR|XP_007417344.1 MORY|QBZ57787.1 MORY|QBZ62551.1 MORY|QBZ63047.1 MORY|QBZ63291.1 NCRA|XP_960424.1 NCRA|XP_962123.2 SSCL|APA05652.1 SSCL|APA07886.1 SSCL|APA15889.1

>Orthogroup200: ANID|CBF81902.1 BCIN|XP_001557447.1 BCIN|XP_024550096.1 BGRA|VCU39970.1 BGRA|VCU41025.1 CFRU|XP_031876876.1 CFRU|XP_031877170.1 CFRU|XP_031881597.1 CGLO|KAF3799844.1 CGLO|KAF3801780.1 CGLO|KAF3806411.1 CGLO|KAF3807408.1 CHIG|XP_018151316.1 CHIG|XP_018153828.1 CHIG|XP_018156813.1 CHIG|XP_018162294.1 CVIN|KAF4921697.1 CVIN|KAF4923259.1 CVIN|KAF4929596.1 CVYL|A09671 CVYL|A10903 CVYL|A11611 CVYL|A12004 FGRM|XP_011321060.1 FGRM|XP_011321541.1 FGRM|XP_011321918.1 FGRM|XP_011326560.1 MGRA|XP_003854848.1 MLAR|XP_007409103.1 MLAR|XP_007411030.1 MLAR|XP_007412555.1 MLAR|XP_007412558.1 MLAR|XP_007413969.1 MLAR|XP_007414777.1 MLAR|XP_007416564.1 MORY|QBZ63910.1 NCRA|XP_956796.1 SSCL|APA10657.1 SSCL|APA10723.1

>Orthogroup201: ANID|CBF84133.1 ANID|CBF85356.1 ANID|CBF89127.1 BGRA|VCU40842.1 CFRU|XP_031876870.1 CFRU|XP_031879754.1 CFRU|XP_031881032.1 CFRU|XP_031881146.1 CGLO|KAF3797933.1 CGLO|KAF3797934.1 CGLO|KAF3798193.1 CGLO|KAF3799843.1 CGLO|KAF3809878.1 CGLO|KAF3809904.1 CHIG|XP_018154198.1 CHIG|XP_018158061.1 CHIG|XP_018161437.1 CHIG|XP_018161491.1 CVIN|KAF4908471.1 CVIN|KAF4916791.1 CVIN|KAF4916792.1 CVIN|KAF4917843.1 CVIN|KAF4921698.1 CVIN|KAF4930647.1 CVIN|KAF4930667.1 CVIN|KAF4930713.1 CVYL|A01714 CVYL|A01738 CVYL|A04926 CVYL|A09673 FGRM|XP_011317654.1 FGRM|XP_011320899.1 FGRM|XP_011322054.1 FGRM|XP_011322605.1 FGRM|XP_011322632.1 MGRA|XP_003854323.1 MGRA|XP_003855418.1 MORY|QBZ53693.1 MORY|QBZ60388.1

>Orthogroup202: ANID|CBF90011.1 BCIN|XP_001555001.1 BCIN|XP_024547432.1 BCIN|XP_024551083.1 CFRU|XP_031875949.1 CFRU|XP_031880018.1 CFRU|XP_031881097.1 CFRU|XP_031883790.1 CFRU|XP_031884216.1 CFRU|XP_031884357.1 CFRU|XP_031890514.1 CGLO|KAF3798975.1 CGLO|KAF3800426.1 CGLO|KAF3806757.1 CGLO|KAF3811042.1 CGLO|KAF3811043.1 CHIG|XP_018150746.1 CHIG|XP_018151677.1 CHIG|XP_018151883.1 CHIG|XP_018152840.1 CHIG|XP_018154159.1 CHIG|XP_018161377.1 CVIN|KAF4901298.1 CVIN|KAF4923476.1 CVYL|A01818 CVYL|A04971 CVYL|A07894 FGRM|XP_011318210.1 FGRM|XP_011320741.1 MGRA|XP_003849938.1 MGRA|XP_003850732.1 MGRA|XP_003854426.1 MLAR|XP_007409612.1 MLAR|XP_007416307.1 MORY|QBZ59489.1 SSCL|APA07627.1 SSCL|APA08149.1 SSCL|APA13431.1 SSCL|APA15966.1

>Orthogroup203: BCIN|XP_001552864.1 BCIN|XP_024545969.1 BCIN|XP_024552823.1 CFRU|XP_031879431.1 CFRU|XP_031885918.1 CFRU|XP_031886100.1 CFRU|XP_031890739.1 CFRU|XP_031890970.1 CGLO|KAF3799656.1 CGLO|KAF3801179.1 CGLO|KAF3801543.1 CGLO|KAF3811720.1 CGLO|KAF3811994.1 CHIG|XP_018151548.1 CHIG|XP_018154133.1 CHIG|XP_018158127.1 CHIG|XP_018163495.1 CHIG|XP_018164741.1 CVIN|KAF4911041.1 CVIN|KAF4921107.1 CVIN|KAF4922163.1 CVIN|KAF4922972.1 CVIN|KAF4930358.1 CVYL|A05513 CVYL|A06212 CVYL|A08087 CVYL|A12593 CVYL|A12737 CVYL|A13519 FGRM|XP_011317399.1 FGRM|XP_011320600.1 FGRM|XP_011320620.1 FGRM|XP_011321891.1 FGRM|XP_011322934.1 MORY|QBZ55151.1 MORY|QBZ56726.1 MORY|QBZ60635.1 NCRA|XP_959490.1 SSCL|APA07414.1

>Orthogroup204: BCIN|XP_001555583.2 BCIN|XP_001560857.1 BCIN|XP_024548681.1 BCIN|XP_024552521.1 BGRA|VCU40923.1 CFRU|XP_031885541.1 CFRU|XP_031887585.1 CFRU|XP_031887586.1 CGLO|KAF3798666.1 CGLO|KAF3803267.1 CHIG|XP_018156620.1 CHIG|XP_018157498.1 CHIG|XP_018161130.1 CVIN|KAF4919450.1 CVIN|KAF4928699.1 CVIN|KAF4928700.1 CVYL|A03702 CVYL|A09405 FGRM|XP_011322710.1 FGRM|XP_011323557.1 FGRM|XP_011328406.1 FGRM|XP_011328575.1 FGRM|XP_011328576.1 FGRM|XP_011328577.1 MGRA|XP_003852107.1 MGRA|XP_003852713.1 MGRA|XP_003857537.1 MORY|QBZ56186.1 MORY|QBZ58452.1 MORY|QBZ65493.1 MORY|QBZ66629.1 MORY|QBZ66654.1 NCRA|XP_011394383.1 NCRA|XP_959389.2 NCRA|XP_963277.2 SSCL|APA07445.1 SSCL|APA08078.1 SSCL|APA09906.1 SSCL|APA15475.1

>Orthogroup205: BCIN|XP_001545511.1 BCIN|XP_001547970.1 BCIN|XP_024549544.1 BCIN|XP_024552322.1 CFRU|XP_031880231.1 CFRU|XP_031880413.1 CFRU|XP_031882996.1 CFRU|XP_031887385.1 CFRU|XP_031889936.1 CFRU|XP_031890551.1 CGLO|KAF3804879.1 CGLO|KAF3807268.1 CGLO|KAF3807949.1 CGLO|KAF3808979.1 CGLO|KAF3809501.1 CGLO|KAF3810409.1 CHIG|XP_018150838.1 CHIG|XP_018154118.1 CHIG|XP_018156492.1 CHIG|XP_018157690.1 CHIG|XP_018163620.1 CVIN|KAF4908455.1 CVIN|KAF4920847.1 CVIN|KAF4921160.1 CVIN|KAF4921256.1 CVIN|KAF4924064.1 CVIN|KAF4928185.1 CVYL|A00677 CVYL|A00678 CVYL|A10070 CVYL|A10750 CVYL|A11392 CVYL|A12183 FGRM|XP_011323044.1 FGRM|XP_011324883.1 MGRA|XP_003849644.1 MGRA|XP_003851457.1 MGRA|XP_003857564.1 SSCL|APA08623.1

>Orthogroup206: MLAR|XP_007403532.1 MLAR|XP_007403781.1 MLAR|XP_007403983.1 MLAR|XP_007404057.1 MLAR|XP_007404586.1 MLAR|XP_007404805.1 MLAR|XP_007405900.1 MLAR|XP_007406617.1 MLAR|XP_007406927.1 MLAR|XP_007407841.1 MLAR|XP_007407996.1 MLAR|XP_007408059.1 MLAR|XP_007408088.1 MLAR|XP_007409221.1 MLAR|XP_007409759.1 MLAR|XP_007410326.1 MLAR|XP_007412540.1 MLAR|XP_007412871.1 MLAR|XP_007412941.1 MLAR|XP_007413052.1 MLAR|XP_007413985.1 MLAR|XP_007414291.1 MLAR|XP_007414893.1 MLAR|XP_007415236.1 MLAR|XP_007415410.1 MLAR|XP_007415451.1 MLAR|XP_007416062.1 MLAR|XP_007416661.1 MLAR|XP_007416769.1 MLAR|XP_007417186.1 MLAR|XP_007417451.1 MLAR|XP_007417460.1 MLAR|XP_007418469.1 MLAR|XP_007418539.1 MLAR|XP_007418540.1 MLAR|XP_007419271.1 MLAR|XP_007419274.1 MLAR|XP_007419521.1 MLAR|XP_007419773.1

>Orthogroup207: MLAR|XP_007404259.1 MLAR|XP_007404498.1 MLAR|XP_007405217.1 MLAR|XP_007405493.1 MLAR|XP_007405978.1 MLAR|XP_007406860.1 MLAR|XP_007407260.1 MLAR|XP_007407482.1 MLAR|XP_007407833.1 MLAR|XP_007408118.1 MLAR|XP_007408639.1 MLAR|XP_007409870.1 MLAR|XP_007410254.1 MLAR|XP_007410592.1 MLAR|XP_007411339.1 MLAR|XP_007412507.1 MLAR|XP_007413261.1 MLAR|XP_007413822.1 MLAR|XP_007413979.1 MLAR|XP_007414081.1 MLAR|XP_007414650.1 MLAR|XP_007414753.1 MLAR|XP_007414883.1 MLAR|XP_007415251.1 MLAR|XP_007416274.1 MLAR|XP_007416463.1 MLAR|XP_007417124.1 MLAR|XP_007417330.1 MLAR|XP_007417340.1 MLAR|XP_007417378.1 MLAR|XP_007417725.1 MLAR|XP_007417746.1 MLAR|XP_007418434.1 MLAR|XP_007418790.1 MLAR|XP_007419121.1 MLAR|XP_007419306.1 MLAR|XP_007419635.1 MLAR|XP_007419697.1 MLAR|XP_007419842.1

>Orthogroup208: ANID|CBF69504.1 ANID|CBF85195.1 BCIN|XP_001556114.2 BCIN|XP_001557265.2 BCIN|XP_024546559.1 BCIN|XP_024549460.1 BCIN|XP_024550148.1 CFRU|XP_031877290.1 CFRU|XP_031878697.1 CFRU|XP_031880029.1 CFRU|XP_031880085.1 CFRU|XP_031884646.1 CGLO|KAF3798363.1 CGLO|KAF3799690.1 CGLO|KAF3806810.1 CGLO|KAF3809036.1 CGLO|KAF3811780.1 CHIG|XP_018151760.1 CHIG|XP_018158284.1 CHIG|XP_018159528.1 CVIN|KAF4907637.1 CVIN|KAF4908881.1 CVIN|KAF4919967.1 CVYL|A02467 CVYL|A05854 CVYL|A09000 CVYL|A12785 FGRM|XP_011318265.1 FGRM|XP_011322090.1 FGRM|XP_011322837.1 FGRM|XP_011327627.1 MGRA|XP_003848965.1 MORY|QBZ63135.1 NCRA|XP_958156.1 SSCL|APA06788.1 SSCL|APA08891.1 SSCL|APA09198.1 SSCL|APA10766.1

>Orthogroup209: ANID|CBF69709.1 ANID|CBF76249.1 ANID|CBF85912.1 BCIN|XP_024546994.1 BCIN|XP_024548417.1 BGRA|VCU40101.1 BGRA|VCU40618.1 CFRU|XP_031887942.1 CFRU|XP_031888710.1 CFRU|XP_031888720.1 CGLO|KAF3797586.1 CGLO|KAF3799197.1 CGLO|KAF3804175.1 CHIG|XP_018154109.1 CHIG|XP_018159817.1 CHIG|XP_018164954.1 CVIN|KAF4912178.1 CVIN|KAF4914911.1 CVIN|KAF4918370.1 CVYL|A03402 CVYL|A03849 CVYL|A10092 FGRM|XP_011318618.1 FGRM|XP_011325412.1 MGRA|XP_003848681.1 MGRA|XP_003851942.1 MGRA|XP_003853000.1 MGRA|XP_003856606.1 MLAR|XP_007406056.1 MLAR|XP_007410553.1 MLAR|XP_007410565.1 MLAR|XP_007410566.1 MLAR|XP_007419732.1 MORY|QBZ58685.1 MORY|QBZ60052.1 NCRA|XP_959849.1 SSCL|APA05940.1 SSCL|APA12283.1

>Orthogroup210: ANID|CBF73488.1 ANID|CBF74013.1 ANID|CBF78846.1 ANID|CBF78849.1 ANID|CBF86059.1 BCIN|XP_024550652.1 CFRU|XP_031878324.1 CFRU|XP_031879939.1 CFRU|XP_031882744.1 CFRU|XP_031892196.1 CGLO|KAF3798056.1 CGLO|KAF3798989.1 CGLO|KAF3806664.1 CGLO|KAF3809812.1 CHIG|XP_018152901.1 CHIG|XP_018153316.1 CHIG|XP_018156990.1 CHIG|XP_018158307.1 CHIG|XP_018164210.1 CVIN|KAF4918903.1 CVIN|KAF4919306.1 CVIN|KAF4923493.1 CVIN|KAF4927977.1 CVYL|A06732 CVYL|A07908 CVYL|A08300 CVYL|A14198 FGRM|XP_011320905.1 FGRM|XP_011323239.1 FGRM|XP_011325103.1 MGRA|XP_003849768.1 MGRA|XP_003851986.1 MGRA|XP_003854687.1 MORY|QBZ53621.1 MORY|QBZ61862.1 MORY|QBZ63118.1 NCRA|XP_955888.3 SSCL|APA11478.1

>Orthogroup211: ANID|CBF73492.1 ANID|CBF78493.1 ANID|CBF82819.1 ANID|CBF83145.1 ANID|CBF86048.1 ANID|CBF86206.1 ANID|CBF88297.1 ANID|CBF89299.1 BCIN|XP_001545450.2 BCIN|XP_001548423.1 BCIN|XP_001559066.2 CFRU|XP_031877237.1 CFRU|XP_031878617.1 CFRU|XP_031883582.1 CFRU|XP_031889922.1 CGLO|KAF3798489.1 CGLO|KAF3800214.1 CGLO|KAF3806836.1 CGLO|KAF3810618.1 CHIG|XP_018154036.1 CHIG|XP_018155125.1 CHIG|XP_018158974.1 CVIN|KAF4908289.1 CVIN|KAF4920410.1 CVYL|A02483 CVYL|A06072 CVYL|A13702 FGRM|XP_011315691.1 FGRM|XP_011319292.1 FGRM|XP_011322042.1 MGRA|XP_003849631.1 MGRA|XP_003853623.1 MORY|QBZ57764.1 MORY|QBZ61776.1 NCRA|XP_001728129.1 NCRA|XP_963232.1 SSCL|APA09077.1 SSCL|APA09283.1

>Orthogroup212: ANID|CBF75171.1 ANID|CBF79679.1 BCIN|XP_024546232.1 BCIN|XP_024547320.1 BCIN|XP_024548250.1 BCIN|XP_024552762.1 BCIN|XP_024553560.1 BGRA|VDB96388.1 CFRU|XP_031876695.1 CFRU|XP_031876839.1 CFRU|XP_031877373.1 CFRU|XP_031892618.1 CGLO|KAF3797176.1 CGLO|KAF3797973.1 CGLO|KAF3799903.1 CHIG|XP_018150813.1 CHIG|XP_018150814.1 CHIG|XP_018153308.1 CHIG|XP_018153309.1 CHIG|XP_018157329.1 CHIG|XP_018157461.1 CVIN|KAF4918499.1 CVIN|KAF4918835.1 CVIN|KAF4921693.1 CVYL|A09433 CVYL|A09618 CVYL|A10520 FGRM|XP_011319926.1 FGRM|XP_011321353.1 MGRA|XP_003851332.1 MORY|QBZ54377.1 MORY|QBZ66079.1 NCRA|XP_958096.3 NCRA|XP_965428.2 SSCL|APA06320.1 SSCL|APA08630.1 SSCL|APA10780.1 SSCL|APA10892.1

>Orthogroup213: ANID|CBF78921.1 BCIN|XP_024549034.1 BCIN|XP_024553555.1 BCIN|XP_024553843.1 CFRU|XP_031876138.1 CFRU|XP_031877512.1 CFRU|XP_031881340.1 CFRU|XP_031884985.1 CFRU|XP_031887014.1 CFRU|XP_031888950.1 CGLO|KAF3797536.1 CGLO|KAF3801016.1 CGLO|KAF3804168.1 CGLO|KAF3805750.1 CGLO|KAF3811317.1 CHIG|XP_018159993.1 CHIG|XP_018160543.1 CHIG|XP_018163667.1 CVIN|KAF4912980.1 CVIN|KAF4918377.1 CVIN|KAF4918597.1 CVIN|KAF4921652.1 CVIN|KAF4926611.1 CVIN|KAF4927199.1 CVYL|A01985 CVYL|A03839 CVYL|A04498 CVYL|A05893 CVYL|A09823 CVYL|A11268 FGRM|XP_011322434.1 FGRM|XP_011327629.1 MORY|QBZ53733.1 MORY|QBZ61908.1 MORY|QBZ65588.1 MORY|QBZ66141.1 NCRA|XP_962853.1 SSCL|APA13895.1

>Orthogroup214: ANID|CBF79122.1 ANID|CBF81578.1 ANID|CBF84282.1 BCIN|XP_001555842.2 BCIN|XP_024546800.1 BCIN|XP_024553579.1 CFRU|XP_031880784.1 CFRU|XP_031881397.1 CFRU|XP_031884187.1 CFRU|XP_031885686.1 CFRU|XP_031885948.1 CGLO|KAF3803772.1 CGLO|KAF3805437.1 CGLO|KAF3805740.1 CGLO|KAF3806872.1 CGLO|KAF3810249.1 CHIG|XP_018153145.1 CHIG|XP_018153264.1 CHIG|XP_018155296.1 CHIG|XP_018157939.1 CHIG|XP_018159550.1 CVIN|KAF4895978.1 CVIN|KAF4910792.1 CVIN|KAF4917912.1 CVIN|KAF4926595.1 CVIN|KAF4930383.1 CVYL|A02518 CVYL|A02851 CVYL|A05567 CVYL|A09794 CVYL|A14150 FGRM|XP_011318312.1 FGRM|XP_011325149.1 FGRM|XP_011327663.1 MGRA|XP_003850660.1 NCRA|XP_961264.1 SSCL|APA08436.1 SSCL|APA13619.1

>Orthogroup215: ANID|CBF79809.1 ANID|CBF88875.1 BCIN|XP_001560884.1 BCIN|XP_024552848.1 CFRU|XP_031877056.1 CFRU|XP_031877256.1 CFRU|XP_031883550.1 CFRU|XP_031889329.1 CFRU|XP_031893508.1 CGLO|KAF3797699.1 CGLO|KAF3806824.1 CGLO|KAF3808076.1 CGLO|KAF3810871.1 CGLO|KAF3811082.1 CHIG|XP_018152778.1 CHIG|XP_018153074.1 CHIG|XP_018154969.1 CHIG|XP_018155485.1 CHIG|XP_018159535.1 CHIG|XP_018163153.1 CVIN|KAF4895029.1 CVIN|KAF4908883.1 CVIN|KAF4916346.1 CVIN|KAF4922834.1 CVIN|KAF4928330.1 CVYL|A01071 CVYL|A02273 CVYL|A02469 CVYL|A08157 CVYL|A09713 FGRM|XP_011321172.1 FGRM|XP_011321760.1 FGRM|XP_011325184.1 FGRM|XP_011328473.1 MGRA|XP_003854107.1 MORY|QBZ64356.1 NCRA|XP_011394166.1 SSCL|APA08100.1

>Orthogroup216: ANID|CBF80127.1 ANID|CBF84928.1 BCIN|XP_001545928.1 BCIN|XP_001560321.1 BCIN|XP_024548229.1 BGRA|VDB89176.1 CFRU|XP_031877997.1 CFRU|XP_031878310.1 CFRU|XP_031882283.1 CGLO|KAF3798086.1 CGLO|KAF3804349.1 CHIG|XP_018158150.1 CHIG|XP_018160125.1 CVIN|KAF4918359.1 CVIN|KAF4926497.1 CVIN|KAF4927962.1 CVYL|A04040 CVYL|A08275 CVYL|A13463 FGRM|XP_011317625.1 FGRM|XP_011318189.1 FGRM|XP_011319247.1 FGRM|XP_011319433.1 FGRM|XP_011322057.1 FGRM|XP_011322762.1 FGRM|XP_011323714.1 MGRA|XP_003851781.1 MGRA|XP_003855776.1 MLAR|XP_007407681.1 MLAR|XP_007408463.1 MORY|QBZ53766.1 MORY|QBZ56065.1 MORY|QBZ57845.1 NCRA|XP_955960.3 NCRA|XP_958365.2 NCRA|XP_958967.1 SSCL|APA08822.1 SSCL|APA12481.1

>Orthogroup217: ANID|CBF88238.1 ANID|CBF89648.1 BCIN|XP_001553556.1 BCIN|XP_024550312.1 BCIN|XP_024551834.1 CFRU|XP_031881615.1 CFRU|XP_031882211.1 CGLO|KAF3804376.1 CGLO|KAF3807404.1 CHIG|XP_018156816.1 CHIG|XP_018160185.1 CVIN|KAF4918322.1 CVIN|KAF4929653.1 CVYL|A04069 CVYL|A10908 FGRM|XP_011318709.1 FGRM|XP_011328167.1 MGRA|XP_003847473.1 MGRA|XP_003851988.1 MGRA|XP_003855099.1 MLAR|XP_007405157.1 MLAR|XP_007405160.1 MLAR|XP_007407077.1 MLAR|XP_007408422.1 MLAR|XP_007408509.1 MLAR|XP_007410702.1 MLAR|XP_007410703.1 MLAR|XP_007410704.1 MLAR|XP_007410705.1 MLAR|XP_007417557.1 MORY|QBZ54089.1 MORY|QBZ59853.1 MORY|QBZ65585.1 NCRA|XP_957040.2 NCRA|XP_960114.1 NCRA|XP_965064.1 SSCL|APA10977.1 SSCL|APA14733.1

>Orthogroup218: CFRU|XP_031884771.1 CFRU|XP_031884772.1 CFRU|XP_031884775.1 CFRU|XP_031884776.1 CFRU|XP_031884878.1 CFRU|XP_031884879.1 CFRU|XP_031884956.1 CFRU|XP_031884957.1 CFRU|XP_031885879.1 CGLO|KAF3800970.1 CGLO|KAF3800971.1 CGLO|KAF3800973.1 CGLO|KAF3800974.1 CGLO|KAF3800978.1 CGLO|KAF3800980.1 CGLO|KAF3801155.1 CHIG|XP_018152423.1 CHIG|XP_018153230.1 CHIG|XP_018153231.1 CHIG|XP_018156592.1 CVIN|KAF4911050.1 CVIN|KAF4913260.1 CVIN|KAF4913268.1 CVIN|KAF4913269.1 CVIN|KAF4913275.1 CVIN|KAF4913277.1 CVIN|KAF4913278.1 CVYL|A05691 CVYL|A05927 CVYL|A05930 CVYL|A05932 CVYL|A05933 CVYL|A05934 CVYL|A05935 FGRM|XP_011317551.1 FGRM|XP_011323491.1 MORY|QBZ64856.1 NCRA|XP_011394158.1

>Orthogroup219: MLAR|XP_007403882.1 MLAR|XP_007404058.1 MLAR|XP_007404246.1 MLAR|XP_007404723.1 MLAR|XP_007405128.1 MLAR|XP_007405717.1 MLAR|XP_007406240.1 MLAR|XP_007406948.1 MLAR|XP_007407090.1 MLAR|XP_007407114.1 MLAR|XP_007407135.1 MLAR|XP_007407353.1 MLAR|XP_007408179.1 MLAR|XP_007409573.1 MLAR|XP_007409574.1 MLAR|XP_007410787.1 MLAR|XP_007411207.1 MLAR|XP_007411294.1 MLAR|XP_007411361.1 MLAR|XP_007413190.1 MLAR|XP_007413766.1 MLAR|XP_007414403.1 MLAR|XP_007415012.1 MLAR|XP_007416587.1 MLAR|XP_007416607.1 MLAR|XP_007416753.1 MLAR|XP_007417316.1 MLAR|XP_007417686.1 MLAR|XP_007417992.1 MLAR|XP_007418004.1 MLAR|XP_007418091.1 MLAR|XP_007418194.1 MLAR|XP_007418867.1 MLAR|XP_007419100.1 MLAR|XP_007419117.1 MLAR|XP_007419222.1 MLAR|XP_007419488.1 MLAR|XP_007419770.1

>Orthogroup220: MLAR|XP_007404939.1 MLAR|XP_007406220.1 MLAR|XP_007406259.1 MLAR|XP_007407062.1 MLAR|XP_007407641.1 MLAR|XP_007407742.1 MLAR|XP_007409610.1 MLAR|XP_007409728.1 MLAR|XP_007409729.1 MLAR|XP_007409932.1 MLAR|XP_007411351.1 MLAR|XP_007411737.1 MLAR|XP_007412687.1 MLAR|XP_007412900.1 MLAR|XP_007413017.1 MLAR|XP_007413119.1 MLAR|XP_007413424.1 MLAR|XP_007414506.1 MLAR|XP_007414634.1 MLAR|XP_007414734.1 MLAR|XP_007414880.1 MLAR|XP_007415008.1 MLAR|XP_007415267.1 MLAR|XP_007415363.1 MLAR|XP_007416029.1 MLAR|XP_007416055.1 MLAR|XP_007416630.1 MLAR|XP_007416977.1 MLAR|XP_007416983.1 MLAR|XP_007417020.1 MLAR|XP_007417037.1 MLAR|XP_007417043.1 MLAR|XP_007417834.1 MLAR|XP_007418296.1 MLAR|XP_007418948.1 MLAR|XP_007419050.1 MLAR|XP_007419701.1 MLAR|XP_007419800.1

>Orthogroup221: MLAR|XP_007405721.1 MLAR|XP_007406432.1 MLAR|XP_007406435.1 MLAR|XP_007406458.1 MLAR|XP_007407300.1 MLAR|XP_007407495.1 MLAR|XP_007407610.1 MLAR|XP_007408402.1 MLAR|XP_007408470.1 MLAR|XP_007408471.1 MLAR|XP_007408708.1 MLAR|XP_007408850.1 MLAR|XP_007408869.1 MLAR|XP_007410322.1 MLAR|XP_007410770.1 MLAR|XP_007410820.1 MLAR|XP_007410972.1 MLAR|XP_007411048.1 MLAR|XP_007412512.1 MLAR|XP_007412628.1 MLAR|XP_007412935.1 MLAR|XP_007412936.1 MLAR|XP_007416267.1 MLAR|XP_007416268.1 MLAR|XP_007416269.1 MLAR|XP_007416270.1 MLAR|XP_007416280.1 MLAR|XP_007416302.1 MLAR|XP_007416868.1 MLAR|XP_007417383.1 MLAR|XP_007418027.1 MLAR|XP_007418162.1 MLAR|XP_007418163.1 MLAR|XP_007418390.1 MLAR|XP_007418869.1 MLAR|XP_007419347.1 MLAR|XP_007419348.1 MLAR|XP_007419541.1

>Orthogroup222: ANID|CBF69657.1 ANID|CBF73449.1 BCIN|XP_001551870.1 BCIN|XP_024550686.1 BCIN|XP_024550816.1 BCIN|XP_024552366.1 BCIN|XP_024552984.1 CFRU|XP_031885885.1 CFRU|XP_031889892.1 CFRU|XP_031892585.1 CFRU|XP_031892816.1 CGLO|KAF3807200.1 CGLO|KAF3810489.1 CGLO|KAF3811975.1 CHIG|XP_018152580.1 CHIG|XP_018156547.1 CHIG|XP_018157085.1 CHIG|XP_018159123.1 CVIN|KAF4924637.1 CVIN|KAF4928195.1 CVIN|KAF4930332.1 CVYL|A01136 CVYL|A10590 CVYL|A10679 CVYL|A14092 FGRM|XP_011315799.1 FGRM|XP_011318216.1 FGRM|XP_011318622.1 FGRM|XP_011318971.1 MORY|QBZ59368.1 MORY|QBZ65567.1 NCRA|XP_001728404.1 NCRA|XP_958753.1 NCRA|XP_964708.3 SSCL|APA11510.1 SSCL|APA11829.1 SSCL|APA15019.1

>Orthogroup223: ANID|CBF75970.1 ANID|CBF78463.1 ANID|CBF79016.1 ANID|CBF82943.1 ANID|CBF89281.1 ANID|CBF89401.1 BCIN|XP_024548119.1 CFRU|XP_031877376.1 CFRU|XP_031877575.1 CFRU|XP_031878823.1 CFRU|XP_031889038.1 CFRU|XP_031892409.1 CFRU|XP_031893321.1 CGLO|KAF3797102.1 CGLO|KAF3797487.1 CGLO|KAF3804215.1 CGLO|KAF3806261.1 CGLO|KAF3806263.1 CGLO|KAF3806264.1 CGLO|KAF3806940.1 CGLO|KAF3808316.1 CGLO|KAF3809650.1 CVIN|KAF4921660.1 CVIN|KAF4922408.1 CVIN|KAF4925019.1 CVYL|A01934 CVYL|A08398 CVYL|A10604 CVYL|A11850 FGRM|XP_011315704.1 FGRM|XP_011317552.1 FGRM|XP_011319655.1 FGRM|XP_011321804.1 FGRM|XP_011323367.1 FGRM|XP_011327578.1 FGRM|XP_011327579.1 MORY|QBZ62917.1

>Orthogroup224: ANID|CBF76992.1 ANID|CBF77471.1 ANID|CBF80060.1 ANID|CBF82202.1 BCIN|XP_001558985.1 BCIN|XP_024546644.1 BCIN|XP_024550489.1 BGRA|VCU38785.1 BGRA|VDB90571.1 CFRU|XP_031881534.1 CFRU|XP_031884779.1 CFRU|XP_031884780.1 CFRU|XP_031888538.1 CGLO|KAF3803754.1 CGLO|KAF3812118.1 CHIG|XP_018153174.1 CHIG|XP_018156712.1 CHIG|XP_018158720.1 CVIN|KAF4912464.1 CVIN|KAF4917749.1 CVYL|A11055 CVYL|A14134 FGRM|XP_011320050.1 FGRM|XP_011326775.1 MGRA|XP_003851313.1 MGRA|XP_003851772.1 MGRA|XP_003854123.1 MLAR|XP_007408897.1 MLAR|XP_007409509.1 MLAR|XP_007418331.1 MORY|QBZ59793.1 MORY|QBZ65360.1 NCRA|XP_011392792.1 NCRA|XP_965636.2 SSCL|APA05838.1 SSCL|APA06477.1 SSCL|APA11207.1

>Orthogroup225: ANID|CBF79787.1 ANID|CBF80344.1 ANID|CBF84693.1 BCIN|XP_001547281.1 CFRU|XP_031875558.1 CFRU|XP_031876586.1 CFRU|XP_031879610.1 CFRU|XP_031883527.1 CFRU|XP_031890125.1 CFRU|XP_031890799.1 CGLO|KAF3798243.1 CGLO|KAF3800902.1 CGLO|KAF3802746.1 CGLO|KAF3808550.1 CGLO|KAF3810839.1 CGLO|KAF3811691.1 CGLO|KAF3811692.1 CHIG|XP_018151297.1 CHIG|XP_018155033.1 CVIN|KAF4900883.1 CVIN|KAF4904372.1 CVIN|KAF4907265.1 CVIN|KAF4911111.1 CVIN|KAF4915942.1 CVYL|A02300 CVYL|A08069 CVYL|A09226 CVYL|A12717 FGRM|XP_011321131.1 FGRM|XP_011327524.1 FGRM|XP_011327561.1 MGRA|XP_003848638.1 MGRA|XP_003853188.1 MORY|QBZ55085.1 MORY|QBZ59962.1 NCRA|XP_959804.3 SSCL|APA06985.1

>Orthogroup226: ANID|CBF80110.1 ANID|CBF80628.1 BCIN|XP_024552248.1 CFRU|XP_031880920.1 CFRU|XP_031881867.1 CFRU|XP_031883967.1 CFRU|XP_031884066.1 CFRU|XP_031884280.1 CFRU|XP_031887989.1 CFRU|XP_031890813.1 CFRU|XP_031890815.1 CFRU|XP_031891026.1 CFRU|XP_031892171.1 CGLO|KAF3797470.1 CGLO|KAF3799986.1 CGLO|KAF3801319.1 CGLO|KAF3802253.1 CGLO|KAF3804973.1 CGLO|KAF3806957.1 CGLO|KAF3810014.1 CHIG|XP_018164786.1 CVIN|KAF4888678.1 CVIN|KAF4910744.1 CVIN|KAF4919172.1 CVIN|KAF4924427.1 CVIN|KAF4924429.1 CVIN|KAF4926875.1 CVYL|A02613 CVYL|A03844 CVYL|A06246 CVYL|A10229 FGRM|XP_011320931.1 FGRM|XP_011321962.1 FGRM|XP_011323429.1 MGRA|XP_003852006.1 MORY|QBZ58846.1 SSCL|APA08970.1

>Orthogroup227: ANID|CBF85553.1 BCIN|XP_001557831.1 BCIN|XP_024548778.1 BCIN|XP_024551324.1 BCIN|XP_024551681.1 CFRU|XP_031875902.1 CFRU|XP_031875928.1 CFRU|XP_031884143.1 CFRU|XP_031888717.1 CFRU|XP_031893549.1 CGLO|KAF3799199.1 CGLO|KAF3800015.1 CGLO|KAF3807808.1 CGLO|KAF3809624.1 CHIG|XP_018153019.1 CHIG|XP_018159815.1 CVIN|KAF4894526.1 CVIN|KAF4907628.1 CVIN|KAF4921522.1 CVIN|KAF4926903.1 CVYL|A00796 CVYL|A08419 CVYL|A09790 CVYL|A09836 CVYL|A09837 CVYL|A10094 FGRM|XP_011317402.1 FGRM|XP_011323409.1 FGRM|XP_011324349.1 MGRA|XP_003849999.1 MORY|QBZ57554.1 MORY|QBZ58065.1 MORY|QBZ61228.1 MORY|QBZ63756.1 NCRA|XP_959656.1 NCRA|XP_964437.2 SSCL|APA09693.1

>Orthogroup228: ANID|CBF86006.1 ANID|CBF87105.1 BCIN|XP_001554112.1 CFRU|XP_031878303.1 CFRU|XP_031879138.1 CFRU|XP_031886405.1 CFRU|XP_031891192.1 CFRU|XP_031891676.1 CGLO|KAF3798093.1 CGLO|KAF3800127.1 CGLO|KAF3802620.1 CGLO|KAF3806290.1 CGLO|KAF3807084.1 CHIG|XP_018151616.1 CHIG|XP_018156680.1 CHIG|XP_018158144.1 CHIG|XP_018161322.1 CHIG|XP_018165060.1 CVIN|KAF4905516.1 CVIN|KAF4919043.1 CVIN|KAF4919044.1 CVIN|KAF4923951.1 CVIN|KAF4925869.1 CVIN|KAF4927978.1 CVYL|A01916 CVYL|A05296 CVYL|A06378 CVYL|A06814 CVYL|A08268 FGRM|XP_011321265.1 MORY|QBZ53337.1 MORY|QBZ53684.1 MORY|QBZ55146.1 MORY|QBZ58379.1 MORY|QBZ64151.1 NCRA|XP_964833.1 SSCL|APA05541.1

>Orthogroup229: BGRA|VDB85692.1 BGRA|VDB85696.1 BGRA|VDB87940.1 BGRA|VDB87942.1 BGRA|VDB87953.1 BGRA|VDB87971.1 BGRA|VDB87985.1 BGRA|VDB87993.1 BGRA|VDB87995.1 BGRA|VDB87997.1 BGRA|VDB88001.1 BGRA|VDB88004.1 BGRA|VDB88005.1 BGRA|VDB88007.1 BGRA|VDB88008.1 BGRA|VDB88009.1 BGRA|VDB88010.1 BGRA|VDB88011.1 BGRA|VDB88012.1 BGRA|VDB88013.1 BGRA|VDB88014.1 BGRA|VDB88016.1 BGRA|VDB88017.1 BGRA|VDB88018.1 BGRA|VDB89037.1 BGRA|VDB89040.1 BGRA|VDB89043.1 BGRA|VDB89044.1 BGRA|VDB89047.1 BGRA|VDB89048.1 BGRA|VDB89051.1 BGRA|VDB91207.1 BGRA|VDB91239.1 BGRA|VDB91243.1 BGRA|VDB91249.1 BGRA|VDB91253.1 BGRA|VDB91255.1

>Orthogroup230: CFRU|XP_031878342.1 CFRU|XP_031881057.1 CFRU|XP_031884365.1 CFRU|XP_031887750.1 CFRU|XP_031889923.1 CFRU|XP_031890067.1 CGLO|KAF3798090.1 CGLO|KAF3802148.1 CGLO|KAF3802837.1 CGLO|KAF3809916.1 CGLO|KAF3811021.1 CGLO|KAF3811625.1 CHIG|XP_018156516.1 CHIG|XP_018157245.1 CHIG|XP_018158147.1 CHIG|XP_018160897.1 CVIN|KAF4908277.1 CVIN|KAF4908278.1 CVIN|KAF4910466.1 CVIN|KAF4918155.1 CVIN|KAF4921429.1 CVIN|KAF4927958.1 CVIN|KAF4930733.1 CVYL|A01705 CVYL|A03484 CVYL|A05175 CVYL|A08272 CVYL|A09755 CVYL|A13703 FGRM|XP_011315753.1 FGRM|XP_011324394.1 FGRM|XP_011326052.1 FGRM|XP_011327478.1 MGRA|XP_003853636.1 MORY|QBZ54815.1 MORY|QBZ58476.1 MORY|QBZ63074.1

>Orthogroup231: MLAR|XP_007403690.1 MLAR|XP_007403697.1 MLAR|XP_007404178.1 MLAR|XP_007404179.1 MLAR|XP_007404181.1 MLAR|XP_007404182.1 MLAR|XP_007404194.1 MLAR|XP_007405707.1 MLAR|XP_007406076.1 MLAR|XP_007406600.1 MLAR|XP_007406694.1 MLAR|XP_007407448.1 MLAR|XP_007407745.1 MLAR|XP_007407746.1 MLAR|XP_007408201.1 MLAR|XP_007408754.1 MLAR|XP_007409884.1 MLAR|XP_007410518.1 MLAR|XP_007410807.1 MLAR|XP_007410916.1 MLAR|XP_007411564.1 MLAR|XP_007412314.1 MLAR|XP_007412530.1 MLAR|XP_007412532.1 MLAR|XP_007413125.1 MLAR|XP_007413525.1 MLAR|XP_007413928.1 MLAR|XP_007414763.1 MLAR|XP_007415117.1 MLAR|XP_007416292.1 MLAR|XP_007416569.1 MLAR|XP_007417880.1 MLAR|XP_007418536.1 MLAR|XP_007418700.1 MLAR|XP_007418709.1 MLAR|XP_007419360.1 MLAR|XP_007419428.1

>Orthogroup232: ANID|CBF69421.1 ANID|CBF73633.1 BCIN|XP_001556138.1 BCIN|XP_001560261.2 CFRU|XP_031878362.1 CFRU|XP_031888696.1 CGLO|KAF3802640.1 CHIG|XP_018159103.1 CHIG|XP_018161466.1 CHIG|XP_018164203.1 CVIN|KAF4932147.1 CVYL|A07156 FGRM|XP_011315861.1 FGRM|XP_011319671.1 FGRM|XP_011321145.1 FGRM|XP_011322216.1 FGRM|XP_011322344.1 FGRM|XP_011322552.1 FGRM|XP_011323704.1 FGRM|XP_011327551.1 FGRM|XP_011328682.1 MGRA|XP_003847622.1 MGRA|XP_003848398.1 MGRA|XP_003852073.1 MGRA|XP_003852397.1 MGRA|XP_003854104.1 MGRA|XP_003854252.1 MORY|QBZ54460.1 MORY|QBZ62670.1 MORY|QBZ66553.1 SSCL|APA08180.1 SSCL|APA08872.1 SSCL|APA10161.1 SSCL|APA11250.1 SSCL|APA15955.1 SSCL|APA16184.1

>Orthogroup233: ANID|CBF69436.1 ANID|CBF71217.1 ANID|CBF89173.1 BCIN|XP_024547695.1 BCIN|XP_024550743.1 BCIN|XP_024551103.1 BCIN|XP_024551453.1 CFRU|XP_031877899.1 CFRU|XP_031877900.1 CFRU|XP_031889311.1 CFRU|XP_031890008.1 CGLO|KAF3802856.1 CGLO|KAF3810895.1 CGLO|KAF3810896.1 CHIG|XP_018154943.1 CHIG|XP_018159778.1 CVIN|KAF4916350.1 CVIN|KAF4916351.1 CVYL|A01175 CVYL|A02248 FGRM|XP_011319557.1 MGRA|XP_003849657.1 MGRA|XP_003849848.1 MGRA|XP_003850810.1 MGRA|XP_003853255.1 MGRA|XP_003854404.1 MGRA|XP_003854412.1 MORY|QBZ60872.1 MORY|QBZ60893.1 MORY|QBZ66147.1 MORY|QBZ66203.1 NCRA|XP_957515.2 NCRA|XP_964661.1 SSCL|APA07900.1 SSCL|APA11672.1 SSCL|APA12051.1

>Orthogroup234: ANID|CBF69458.1 ANID|CBF74471.1 ANID|CBF75006.1 ANID|CBF78527.1 ANID|CBF79175.1 ANID|CBF80156.1 ANID|CBF83141.1 ANID|CBF84180.1 BCIN|XP_001559357.1 BCIN|XP_001559633.1 CFRU|XP_031881606.1 CFRU|XP_031882567.1 CFRU|XP_031892166.1 CGLO|KAF3803905.1 CGLO|KAF3805398.1 CGLO|KAF3809797.1 CHIG|XP_018151668.1 CHIG|XP_018163741.1 CHIG|XP_018164944.1 CVIN|KAF4917745.1 CVIN|KAF4919317.1 CVIN|KAF4925464.1 CVYL|A06716 CVYL|A08912 CVYL|A11044 CVYL|A11045 FGRM|XP_011325107.1 MGRA|XP_003850935.1 MGRA|XP_003852293.1 MGRA|XP_003853082.1 MGRA|XP_003856702.1 MORY|QBZ59383.1 MORY|QBZ66676.1 SSCL|APA07712.1 SSCL|APA09400.1 SSCL|APA10037.1

>Orthogroup235: ANID|CBF71339.1 ANID|CBF73976.1 ANID|CBF74500.1 ANID|CBF82020.1 ANID|CBF83219.1 ANID|CBF87118.1 ANID|CBF87441.1 ANID|CBF89587.1 CFRU|XP_031876415.1 CFRU|XP_031878334.1 CFRU|XP_031883535.1 CFRU|XP_031885738.1 CGLO|KAF3798023.1 CGLO|KAF3801957.1 CGLO|KAF3810043.1 CGLO|KAF3810850.1 CHIG|XP_018154985.1 CHIG|XP_018157764.1 CHIG|XP_018158081.1 CVIN|KAF4911107.1 CVIN|KAF4915607.1 CVIN|KAF4917942.1 CVIN|KAF4919786.1 CVYL|A02392 CVYL|A02639 CVYL|A08333 FGRM|XP_011320237.1 FGRM|XP_011321166.1 FGRM|XP_011321868.1 FGRM|XP_011322850.1 FGRM|XP_011324267.1 FGRM|XP_011327308.1 FGRM|XP_011327643.1 MORY|QBZ55865.1 MORY|QBZ65151.1 SSCL|APA09612.1

>Orthogroup236: ANID|CBF71392.1 ANID|CBF85749.1 ANID|CBF89709.1 BCIN|XP_001554780.1 BCIN|XP_024546268.1 BCIN|XP_024546935.1 BCIN|XP_024548221.1 CFRU|XP_031877819.1 CFRU|XP_031878680.1 CFRU|XP_031881203.1 CFRU|XP_031891819.1 CGLO|KAF3800075.1 CGLO|KAF3802474.1 CGLO|KAF3803837.1 CGLO|KAF3806610.1 CHIG|XP_018151092.1 CHIG|XP_018154209.1 CHIG|XP_018154775.1 CHIG|XP_018164245.1 CVIN|KAF4891447.1 CVIN|KAF4916877.1 CVIN|KAF4929743.1 CVYL|A02065 CVYL|A08963 CVYL|A12319 FGRM|XP_011315670.1 FGRM|XP_011317021.1 FGRM|XP_011320745.1 FGRM|XP_011326139.1 FGRM|XP_011328021.1 MGRA|XP_003849629.1 MGRA|XP_003853525.1 MORY|QBZ55435.1 NCRA|XP_964639.1 SSCL|APA12474.1 SSCL|APA15507.1

>Orthogroup237: ANID|CBF71489.1 BCIN|XP_001554418.2 BCIN|XP_001557419.2 BCIN|XP_024548106.1 CFRU|XP_031875736.1 CFRU|XP_031876254.1 CFRU|XP_031878485.1 CFRU|XP_031886346.1 CFRU|XP_031889829.1 CGLO|KAF3798633.1 CGLO|KAF3802007.1 CGLO|KAF3802336.1 CGLO|KAF3807588.1 CGLO|KAF3808440.1 CGLO|KAF3810559.1 CHIG|XP_018151588.1 CHIG|XP_018153280.1 CHIG|XP_018156346.1 CVIN|KAF4892078.1 CVIN|KAF4928950.1 CVYL|A06171 CVYL|A08672 CVYL|A11699 CVYL|A13637 FGRM|XP_011320706.1 FGRM|XP_011322020.1 FGRM|XP_011323209.1 FGRM|XP_011327513.1 FGRM|XP_011328858.1 MORY|QBZ60839.1 MORY|QBZ62021.1 NCRA|XP_964375.1 SSCL|APA08685.1 SSCL|APA10360.1 SSCL|APA15662.1 SSCL|APA16013.1

>Orthogroup238: ANID|CBF73471.1 ANID|CBF82386.1 ANID|CBF84347.1 ANID|CBF89307.1 BCIN|XP_001551859.1 BCIN|XP_001556004.2 BCIN|XP_024547560.1 CFRU|XP_031877401.1 CFRU|XP_031879806.1 CFRU|XP_031883189.1 CFRU|XP_031888636.1 CFRU|XP_031889014.1 CGLO|KAF3799226.1 CGLO|KAF3803044.1 CGLO|KAF3804269.1 CGLO|KAF3808436.1 CHIG|XP_018153591.1 CHIG|XP_018154034.1 CHIG|XP_018159846.1 CHIG|XP_018163267.1 CVIN|KAF4909054.1 CVIN|KAF4909302.1 CVIN|KAF4929953.1 CVIN|KAF4931497.1 CVYL|A00056 CVYL|A03951 CVYL|A10120 CVYL|A11707 FGRM|XP_011325783.1 MGRA|XP_003856322.1 MLAR|XP_007406183.1 MORY|QBZ60004.1 MORY|QBZ62666.1 NCRA|XP_956346.1 SSCL|APA13932.1 SSCL|APA15228.1

>Orthogroup239: ANID|CBF73556.1 ANID|CBF78942.1 ANID|CBF79702.1 ANID|CBF81521.1 BCIN|XP_001552855.1 BCIN|XP_001555710.2 BCIN|XP_024552333.1 CFRU|XP_031878289.1 CFRU|XP_031880622.1 CFRU|XP_031887544.1 CGLO|KAF3803290.1 CGLO|KAF3809934.1 CHIG|XP_018155451.1 CHIG|XP_018158136.1 CHIG|XP_018161140.1 CHIG|XP_018161512.1 CVIN|KAF4927935.1 CVIN|KAF4928683.1 CVIN|KAF4930705.1 CVYL|A01690 CVYL|A03725 CVYL|A08259 FGRM|XP_011318381.1 FGRM|XP_011319578.1 FGRM|XP_011323245.1 FGRM|XP_011327965.1 MGRA|XP_003852246.1 MGRA|XP_003855679.1 MORY|QBZ58074.1 MORY|QBZ63391.1 MORY|QBZ64561.1 NCRA|XP_957951.1 NCRA|XP_962206.3 NCRA|XP_962454.1 NCRA|XP_964791.2 SSCL|APA13187.1

>Orthogroup240: ANID|CBF73577.1 ANID|CBF73579.1 ANID|CBF76273.1 ANID|CBF79191.1 ANID|CBF82104.1 BCIN|XP_024546795.1 BCIN|XP_024550941.1 CFRU|XP_031879832.1 CFRU|XP_031889842.1 CFRU|XP_031890316.1 CFRU|XP_031891262.1 CGLO|KAF3797433.1 CGLO|KAF3803000.1 CGLO|KAF3805233.1 CGLO|KAF3808335.1 CHIG|XP_018153609.1 CHIG|XP_018155495.1 CHIG|XP_018157392.1 CHIG|XP_018160336.1 CVIN|KAF4904109.1 CVIN|KAF4918676.1 CVIN|KAF4929414.1 CVIN|KAF4929950.1 CVYL|A00011 CVYL|A09217 CVYL|A10243 CVYL|A13883 FGRM|XP_011319726.1 FGRM|XP_011321233.1 FGRM|XP_011322009.1 FGRM|XP_011322508.1 FGRM|XP_011328599.1 MGRA|XP_003854154.1 MLAR|XP_007414191.1 MORY|QBZ53459.1 SSCL|APA06094.1

>Orthogroup241: ANID|CBF73701.1 ANID|CBF74378.1 ANID|CBF80796.1 ANID|CBF81860.1 ANID|CBF85715.1 ANID|CBF86345.1 ANID|CBF88912.1 ANID|CBF89974.1 BCIN|XP_001549274.2 BCIN|XP_001559423.2 BCIN|XP_024548039.1 CFRU|XP_031875798.1 CFRU|XP_031878311.1 CFRU|XP_031880262.1 CGLO|KAF3797583.1 CGLO|KAF3798089.1 CGLO|KAF3809014.1 CHIG|XP_018154154.1 CHIG|XP_018155643.1 CHIG|XP_018155851.1 CHIG|XP_018164773.1 CVIN|KAF4912163.1 CVIN|KAF4918017.1 CVIN|KAF4927959.1 CVYL|A01583 CVYL|A03405 CVYL|A08273 FGRM|XP_011321034.1 FGRM|XP_011325658.1 FGRM|XP_011325659.1 MGRA|XP_003847666.1 MGRA|XP_003855040.1 MGRA|XP_003855544.1 MGRA|XP_003857061.1 MGRA|XP_003857479.1 SSCL|APA08279.1

>Orthogroup242: ANID|CBF75260.1 ANID|CBF75265.1 BCIN|XP_001548987.2 BCIN|XP_001548988.2 BCIN|XP_024546027.1 BCIN|XP_024553000.1 BGRA|VDB92878.1 CFRU|XP_031889972.1 CFRU|XP_031889977.1 CFRU|XP_031889978.1 CGLO|KAF3799672.1 CGLO|KAF3802860.1 CHIG|XP_018158457.1 CHIG|XP_018158458.1 CVIN|KAF4895364.1 CVIN|KAF4899964.1 CVIN|KAF4899971.1 CVYL|A05152 CVYL|A05153 CVYL|A05835 FGRM|XP_011316383.1 FGRM|XP_011316384.1 FGRM|XP_011316385.1 MGRA|XP_003856255.1 MGRA|XP_003856450.1 MGRA|XP_003857363.1 MLAR|XP_007405714.1 MLAR|XP_007415321.1 MORY|QBZ55915.1 MORY|QBZ55942.1 NCRA|XP_011392847.1 NCRA|XP_964809.2 NCRA|XP_964810.1 SSCL|APA07016.1 SSCL|APA07017.1 SSCL|APA07301.1

>Orthogroup243: ANID|CBF75857.1 ANID|CBF82342.1 ANID|CBF83194.1 ANID|CBF88877.1 BCIN|XP_024547052.1 CFRU|XP_031883506.1 CFRU|XP_031883830.1 CFRU|XP_031884723.1 CFRU|XP_031884784.1 CFRU|XP_031888758.1 CGLO|KAF3805683.1 CGLO|KAF3811905.1 CGLO|KAF3812099.1 CGLO|KAF3812121.1 CHIG|XP_018153258.1 CHIG|XP_018155152.1 CHIG|XP_018158721.1 CVIN|KAF4911824.1 CVIN|KAF4918120.1 CVIN|KAF4918394.1 CVIN|KAF4920655.1 CVYL|A00277 CVYL|A00305 CVYL|A03860 CVYL|A06153 CVYL|A09866 FGRM|XP_011318113.1 FGRM|XP_011318266.1 FGRM|XP_011323085.1 FGRM|XP_011327623.1 MGRA|XP_003849811.1 MGRA|XP_003855984.1 MORY|QBZ60945.1 NCRA|XP_956457.1 NCRA|XP_958714.3 SSCL|APA14511.1

>Orthogroup244: ANID|CBF75992.1 ANID|CBF76652.1 BCIN|XP_001552718.2 BGRA|VDB83721.1 BGRA|VDB89193.1 CFRU|XP_031881501.1 CFRU|XP_031886257.1 CFRU|XP_031888216.1 CFRU|XP_031889200.1 CGLO|KAF3803165.1 CGLO|KAF3803659.1 CGLO|KAF3806214.1 CHIG|XP_018155510.1 CHIG|XP_018162050.1 CHIG|XP_018163799.1 CVIN|KAF4926645.1 CVIN|KAF4928353.1 CVIN|KAF4928501.1 CVIN|KAF4931469.1 CVYL|A01052 CVYL|A09815 CVYL|A11803 CVYL|A14592 FGRM|XP_011317010.1 FGRM|XP_011319670.1 FGRM|XP_011321778.1 FGRM|XP_011321991.1 FGRM|XP_011328711.1 MGRA|XP_003847730.1 MGRA|XP_003849481.1 MGRA|XP_003853705.1 MGRA|XP_003857127.1 MORY|QBZ58121.1 NCRA|XP_961795.1 NCRA|XP_963121.2 SSCL|APA14071.1

>Orthogroup245: ANID|CBF77345.1 BCIN|XP_024550380.1 BCIN|XP_024551705.1 BGRA|VDB94878.1 CFRU|XP_031878285.1 CFRU|XP_031884705.1 CFRU|XP_031886469.1 CFRU|XP_031892180.1 CGLO|KAF3798072.1 CGLO|KAF3801024.1 CGLO|KAF3801317.1 CHIG|XP_018152719.1 CHIG|XP_018153324.1 CHIG|XP_018161987.1 CHIG|XP_018162177.1 CVIN|KAF4889708.1 CVIN|KAF4921578.1 CVIN|KAF4927998.1 CVIN|KAF4931486.1 CVYL|A00360 CVYL|A07768 CVYL|A08286 CVYL|A11779 FGRM|XP_011318715.1 FGRM|XP_011321881.1 FGRM|XP_011322753.1 FGRM|XP_011324074.1 MGRA|XP_003853908.1 MGRA|XP_003857723.1 MLAR|XP_007409050.1 MORY|QBZ55244.1 MORY|QBZ59554.1 NCRA|XP_956997.3 NCRA|XP_965328.3 SSCL|APA11070.1 SSCL|APA14540.1

>Orthogroup246: ANID|CBF78065.1 ANID|CBF78981.1 ANID|CBF87301.1 BCIN|XP_024552431.1 CFRU|XP_031883271.1 CFRU|XP_031886092.1 CFRU|XP_031891569.1 CFRU|XP_031892059.1 CFRU|XP_031893509.1 CGLO|KAF3801292.1 CGLO|KAF3802347.1 CGLO|KAF3804685.1 CGLO|KAF3805502.1 CGLO|KAF3808077.1 CHIG|XP_018152210.1 CHIG|XP_018152779.1 CHIG|XP_018161586.1 CHIG|XP_018161755.1 CVIN|KAF4898351.1 CVIN|KAF4918319.1 CVIN|KAF4919227.1 CVIN|KAF4921564.1 CVIN|KAF4922832.1 CVYL|A05198 CVYL|A05626 CVYL|A07795 CVYL|A08156 CVYL|A13210 FGRM|XP_011318323.1 FGRM|XP_011318639.1 FGRM|XP_011321310.1 FGRM|XP_011322101.1 MGRA|XP_003849590.1 MORY|QBZ57664.1 NCRA|XP_963482.3 SSCL|APA14990.1

>Orthogroup247: ANID|CBF79090.1 ANID|CBF85580.1 BCIN|XP_001551724.1 BCIN|XP_024548731.1 BCIN|XP_024548825.1 CFRU|XP_031886626.1 CFRU|XP_031888464.1 CGLO|KAF3797799.1 CGLO|KAF3807515.1 CHIG|XP_018156460.1 CHIG|XP_018158593.1 CVIN|KAF4917522.1 CVIN|KAF4923128.1 CVYL|A03027 CVYL|A05037 FGRM|XP_011318591.1 FGRM|XP_011322198.1 FGRM|XP_011328407.1 MGRA|XP_003849998.1 MGRA|XP_003852295.1 MGRA|XP_003855311.1 MLAR|XP_007413872.1 MLAR|XP_007417482.1 MLAR|XP_007417483.1 MLAR|XP_007417485.1 MLAR|XP_007417486.1 MLAR|XP_007417491.1 MORY|QBZ58959.1 MORY|QBZ60486.1 NCRA|XP_959411.2 NCRA|XP_959573.2 NCRA|XP_965713.1 SSCL|APA08983.1 SSCL|APA09617.1 SSCL|APA09772.1 SSCL|APA11917.1

>Orthogroup248: ANID|CBF80630.1 ANID|CBF88272.1 BCIN|XP_024550080.1 CFRU|XP_031877975.1 CFRU|XP_031879713.1 CFRU|XP_031883126.1 CFRU|XP_031883407.1 CFRU|XP_031889901.1 CFRU|XP_031890691.1 CFRU|XP_031891256.1 CFRU|XP_031893004.1 CGLO|KAF3798578.1 CGLO|KAF3801708.1 CGLO|KAF3804661.1 CGLO|KAF3806749.1 CGLO|KAF3807959.1 CGLO|KAF3809588.1 CGLO|KAF3810633.1 CGLO|KAF3810888.1 CHIG|XP_018152601.1 CHIG|XP_018154929.1 CHIG|XP_018159223.1 CHIG|XP_018159224.1 CVIN|KAF4901599.1 CVIN|KAF4913896.1 CVIN|KAF4916372.1 CVIN|KAF4919204.1 CVYL|A02256 CVYL|A04865 CVYL|A05224 CVYL|A08447 CVYL|A13718 FGRM|XP_011325718.1 FGRM|XP_011327426.1 MGRA|XP_003856570.1 MORY|QBZ55017.1

>Orthogroup249: ANID|CBF83860.1 ANID|CBF84998.1 BCIN|XP_001559117.1 BCIN|XP_001561246.2 BCIN|XP_024547527.1 CFRU|XP_031879984.1 CFRU|XP_031881085.1 CFRU|XP_031884088.1 CFRU|XP_031887029.1 CFRU|XP_031891042.1 CGLO|KAF3800469.1 CGLO|KAF3801625.1 CGLO|KAF3811057.1 CGLO|KAF3811286.1 CHIG|XP_018151387.1 CHIG|XP_018153131.1 CHIG|XP_018160511.1 CHIG|XP_018161356.1 CVIN|KAF4917613.1 CVIN|KAF4918148.1 CVIN|KAF4922703.1 CVIN|KAF4927225.1 CVYL|A01852 CVYL|A04446 CVYL|A09740 CVYL|A12511 FGRM|XP_011323531.1 FGRM|XP_011323626.1 FGRM|XP_011328197.1 MORY|QBZ58030.1 MORY|QBZ59981.1 NCRA|XP_957838.1 NCRA|XP_958630.1 SSCL|APA07216.1 SSCL|APA07706.1 SSCL|APA09044.1

>Orthogroup250: ANID|CBF85629.1 BCIN|XP_001553969.2 BCIN|XP_001554050.2 BCIN|XP_001555756.1 BCIN|XP_024549291.1 CFRU|XP_031877273.1 CFRU|XP_031879674.1 CFRU|XP_031885125.1 CFRU|XP_031885274.1 CFRU|XP_031891800.1 CFRU|XP_031893340.1 CFRU|XP_031893542.1 CGLO|KAF3800055.1 CGLO|KAF3802013.1 CGLO|KAF3807768.1 CGLO|KAF3808116.1 CHIG|XP_018152361.1 CHIG|XP_018152816.1 CHIG|XP_018158058.1 CHIG|XP_018158079.1 CHIG|XP_018165000.1 CVIN|KAF4909500.1 CVIN|KAF4914233.1 CVIN|KAF4919332.1 CVIN|KAF4920526.1 CVIN|KAF4920644.1 CVYL|A02441 CVYL|A06754 CVYL|A09463 FGRM|XP_011317754.1 FGRM|XP_011325986.1 MGRA|XP_003847947.1 MGRA|XP_003853416.1 MGRA|XP_003856550.1 MORY|QBZ58090.1 SSCL|APA10117.1

>Orthogroup251: ANID|CBF87387.1 BCIN|XP_001548259.1 BCIN|XP_001558960.2 BCIN|XP_024549993.1 BGRA|VDB94452.1 CFRU|XP_031881063.1 CFRU|XP_031883098.1 CFRU|XP_031885344.1 CFRU|XP_031886865.1 CGLO|KAF3806517.1 CGLO|KAF3808318.1 CGLO|KAF3809907.1 CGLO|KAF3811218.1 CHIG|XP_018157554.1 CHIG|XP_018160427.1 CHIG|XP_018161493.1 CHIG|XP_018163116.1 CVIN|KAF4921333.1 CVIN|KAF4924066.1 CVIN|KAF4930653.1 CVIN|KAF4931207.1 CVYL|A00649 CVYL|A01712 CVYL|A04385 CVYL|A09353 FGRM|XP_011318646.1 FGRM|XP_011327155.1 FGRM|XP_011327982.1 MGRA|XP_003856857.1 MLAR|XP_007418384.1 MORY|QBZ57058.1 MORY|QBZ58928.1 NCRA|XP_958599.1 SSCL|APA05318.1 SSCL|APA05827.1 SSCL|APA09932.1

>Orthogroup252: BCIN|XP_001552831.1 BCIN|XP_001553224.2 BGRA|VCU41352.1 CFRU|XP_031875844.1 CFRU|XP_031877061.1 CFRU|XP_031879335.1 CGLO|KAF3805545.1 CGLO|KAF3808673.1 CGLO|KAF3811093.1 CHIG|XP_018151578.1 CHIG|XP_018152981.1 CHIG|XP_018161839.1 CVIN|KAF4890966.1 CVIN|KAF4911085.1 CVIN|KAF4926126.1 CVYL|A05668 CVYL|A09701 CVYL|A11656 FGRM|XP_011317424.1 MGRA|XP_003852433.1 MLAR|XP_007409787.1 MLAR|XP_007409851.1 MLAR|XP_007410141.1 MLAR|XP_007412205.1 MLAR|XP_007412206.1 MLAR|XP_007413164.1 MLAR|XP_007413506.1 MLAR|XP_007414207.1 MLAR|XP_007416104.1 MLAR|XP_007417651.1 MLAR|XP_007417652.1 MLAR|XP_007417653.1 MORY|QBZ63025.1 NCRA|XP_959686.1 SSCL|APA07608.1 SSCL|APA12072.1

>Orthogroup253: BGRA|VDB91097.1 SSCL|APA05251.1 SSCL|APA05858.1 SSCL|APA06422.1 SSCL|APA06458.1 SSCL|APA06564.1 SSCL|APA07399.1 SSCL|APA07400.1 SSCL|APA07555.1 SSCL|APA07573.1 SSCL|APA07728.1 SSCL|APA09119.1 SSCL|APA09241.1 SSCL|APA09315.1 SSCL|APA09316.1 SSCL|APA09338.1 SSCL|APA09345.1 SSCL|APA10088.1 SSCL|APA10722.1 SSCL|APA11526.1 SSCL|APA11527.1 SSCL|APA11746.1 SSCL|APA13150.1 SSCL|APA13800.1 SSCL|APA13893.1 SSCL|APA13904.1 SSCL|APA14053.1 SSCL|APA14054.1 SSCL|APA14084.1 SSCL|APA14085.1 SSCL|APA14521.1 SSCL|APA15575.1 SSCL|APA15659.1 SSCL|APA15803.1 SSCL|APA16120.1 SSCL|APA16354.1

>Orthogroup254: MLAR|XP_007403692.1 MLAR|XP_007403787.1 MLAR|XP_007403930.1 MLAR|XP_007404048.1 MLAR|XP_007404573.1 MLAR|XP_007406160.1 MLAR|XP_007406479.1 MLAR|XP_007406545.1 MLAR|XP_007406547.1 MLAR|XP_007407354.1 MLAR|XP_007407406.1 MLAR|XP_007407407.1 MLAR|XP_007407570.1 MLAR|XP_007407766.1 MLAR|XP_007408247.1 MLAR|XP_007409945.1 MLAR|XP_007410171.1 MLAR|XP_007410562.1 MLAR|XP_007410736.1 MLAR|XP_007411364.1 MLAR|XP_007413541.1 MLAR|XP_007414534.1 MLAR|XP_007415132.1 MLAR|XP_007415358.1 MLAR|XP_007415948.1 MLAR|XP_007416052.1 MLAR|XP_007416260.1 MLAR|XP_007416663.1 MLAR|XP_007416666.1 MLAR|XP_007417892.1 MLAR|XP_007418198.1 MLAR|XP_007418472.1 MLAR|XP_007418888.1 MLAR|XP_007418999.1 MLAR|XP_007419294.1 MLAR|XP_007419730.1

>Orthogroup255: MLAR|XP_007404914.1 MLAR|XP_007405884.1 MLAR|XP_007406086.1 MLAR|XP_007406296.1 MLAR|XP_007406297.1 MLAR|XP_007406298.1 MLAR|XP_007406835.1 MLAR|XP_007406985.1 MLAR|XP_007407498.1 MLAR|XP_007409602.1 MLAR|XP_007410772.1 MLAR|XP_007410774.1 MLAR|XP_007410848.1 MLAR|XP_007411034.1 MLAR|XP_007411035.1 MLAR|XP_007411121.1 MLAR|XP_007411286.1 MLAR|XP_007411381.1 MLAR|XP_007411393.1 MLAR|XP_007413010.1 MLAR|XP_007413011.1 MLAR|XP_007414908.1 MLAR|XP_007415616.1 MLAR|XP_007416419.1 MLAR|XP_007416420.1 MLAR|XP_007416436.1 MLAR|XP_007416439.1 MLAR|XP_007416860.1 MLAR|XP_007417504.1 MLAR|XP_007417505.1 MLAR|XP_007417535.1 MLAR|XP_007417953.1 MLAR|XP_007418788.1 MLAR|XP_007418800.1 MLAR|XP_007419604.1 MLAR|XP_007419605.1

>Orthogroup256: ANID|CBF73612.1 ANID|CBF82804.1 BCIN|XP_001550078.1 BCIN|XP_001560589.1 BCIN|XP_024546734.1 BCIN|XP_024550333.1 CFRU|XP_031879652.1 CFRU|XP_031884760.1 CFRU|XP_031887194.1 CFRU|XP_031889312.1 CGLO|KAF3798581.1 CGLO|KAF3801059.1 CGLO|KAF3808910.1 CGLO|KAF3809367.1 CHIG|XP_018153266.1 CHIG|XP_018153957.1 CHIG|XP_018155784.1 CHIG|XP_018156222.1 CVIN|KAF4902040.1 CVIN|KAF4918121.1 CVIN|KAF4920732.1 CVIN|KAF4930163.1 CVYL|A00307 CVYL|A01176 CVYL|A01576 CVYL|A04862 FGRM|XP_011322317.1 MGRA|XP_003857130.1 MLAR|XP_007403618.1 MORY|QBZ62655.1 NCRA|XP_011395022.1 SSCL|APA06823.1 SSCL|APA07916.1 SSCL|APA11010.1 SSCL|APA16046.1

>Orthogroup257: ANID|CBF74610.1 ANID|CBF81125.1 ANID|CBF82381.1 ANID|CBF84219.1 ANID|CBF87444.1 BCIN|XP_001556041.2 BCIN|XP_001558622.1 BCIN|XP_024554084.1 CFRU|XP_031888639.1 CFRU|XP_031889645.1 CFRU|XP_031891656.1 CGLO|KAF3804262.1 CGLO|KAF3809718.1 CGLO|KAF3810641.1 CHIG|XP_018152733.1 CHIG|XP_018155540.1 CHIG|XP_018158972.1 CHIG|XP_018164920.1 CVIN|KAF4909072.1 CVIN|KAF4917783.1 CVIN|KAF4921967.1 CVYL|A03944 CVYL|A06641 CVYL|A13725 FGRM|XP_011315661.1 FGRM|XP_011322489.1 FGRM|XP_011327667.1 FGRM|XP_011327874.1 MGRA|XP_003848998.1 MGRA|XP_003855430.1 MGRA|XP_003856408.1 MORY|QBZ57758.1 MORY|QBZ66410.1 NCRA|XP_955910.1 SSCL|APA05679.1

>Orthogroup258: ANID|CBF75109.1 ANID|CBF76686.1 ANID|CBF84137.1 ANID|CBF85351.1 ANID|CBF89119.1 BCIN|XP_024547438.1 CFRU|XP_031875382.1 CFRU|XP_031876081.1 CFRU|XP_031881009.1 CFRU|XP_031881089.1 CFRU|XP_031882962.1 CGLO|KAF3801785.1 CGLO|KAF3807964.1 CGLO|KAF3809877.1 CGLO|KAF3809902.1 CHIG|XP_018159441.1 CHIG|XP_018161438.1 CHIG|XP_018161489.1 CVIN|KAF4916995.1 CVIN|KAF4919781.1 CVIN|KAF4924037.1 CVIN|KAF4930644.1 CVIN|KAF4930712.1 CVYL|A00664 CVYL|A01716 CVYL|A01739 CVYL|A01740 CVYL|A02430 FGRM|XP_011322051.1 FGRM|XP_011322607.1 MGRA|XP_003857434.1 MORY|QBZ53692.1 MORY|QBZ59986.1 NCRA|XP_963196.1 SSCL|APA15974.1

>Orthogroup259: ANID|CBF75152.1 ANID|CBF81741.1 ANID|CBF82143.1 BCIN|XP_024547186.1 BCIN|XP_024548371.1 BGRA|VCU39581.1 CFRU|XP_031879805.1 CFRU|XP_031884947.1 CFRU|XP_031887400.1 CFRU|XP_031889924.1 CGLO|KAF3803020.1 CGLO|KAF3809280.1 CGLO|KAF3810620.1 CGLO|KAF3812189.1 CHIG|XP_018153727.1 CHIG|XP_018156117.1 CHIG|XP_018158977.1 CVIN|KAF4920972.1 CVIN|KAF4921055.1 CVIN|KAF4929970.1 CVYL|A00033 CVYL|A01474 FGRM|XP_011319145.1 FGRM|XP_011325095.1 FGRM|XP_011327798.1 MGRA|XP_003847873.1 MGRA|XP_003848718.1 MLAR|XP_007403483.1 MLAR|XP_007409284.1 MORY|QBZ60329.1 MORY|QBZ60613.1 NCRA|XP_959787.1 NCRA|XP_961070.1 SSCL|APA12338.1 SSCL|APA14112.1

>Orthogroup260: ANID|CBF77966.1 ANID|CBF88738.1 BCIN|XP_024550637.1 CFRU|XP_031875553.1 CFRU|XP_031876061.1 CFRU|XP_031876077.1 CFRU|XP_031877884.1 CFRU|XP_031888080.1 CFRU|XP_031891871.1 CGLO|KAF3801990.1 CGLO|KAF3802036.1 CGLO|KAF3804764.1 CGLO|KAF3808555.1 CHIG|XP_018150749.1 CHIG|XP_018154132.1 CHIG|XP_018161925.1 CHIG|XP_018164262.1 CVIN|KAF4906498.1 CVIN|KAF4910487.1 CVIN|KAF4919761.1 CVIN|KAF4919764.1 CVIN|KAF4929822.1 CVYL|A02421 CVYL|A03352 CVYL|A11590 FGRM|XP_011315843.1 FGRM|XP_011316001.1 FGRM|XP_011321822.1 MGRA|XP_003850078.1 MGRA|XP_003852191.1 MGRA|XP_003854050.1 MLAR|XP_007404452.1 MLAR|XP_007409857.1 MORY|QBZ63382.1 MORY|QBZ64625.1

>Orthogroup261: ANID|CBF79303.1 ANID|CBF82890.1 ANID|CBF83048.1 ANID|CBF89958.1 BCIN|XP_001549005.2 BCIN|XP_001550723.1 BCIN|XP_001558116.1 BCIN|XP_024551251.1 CFRU|XP_031878209.1 CFRU|XP_031885378.1 CFRU|XP_031889039.1 CGLO|KAF3799184.1 CGLO|KAF3799493.1 CGLO|KAF3808522.1 CHIG|XP_018153899.1 CHIG|XP_018159792.1 CHIG|XP_018160177.1 CHIG|XP_018161258.1 CVIN|KAF4914901.1 CVIN|KAF4917640.1 CVIN|KAF4931882.1 CVYL|A10080 CVYL|A11618 CVYL|A13353 FGRM|XP_011318145.1 FGRM|XP_011322321.1 MGRA|XP_003856978.1 MORY|QBZ54151.1 MORY|QBZ57825.1 MORY|QBZ58307.1 MORY|QBZ62729.1 NCRA|XP_962420.1 NCRA|XP_965604.1 SSCL|APA07002.1 SSCL|APA12965.1

>Orthogroup262: ANID|CBF80637.1 ANID|CBF87524.1 ANID|CBF89256.1 ANID|CBF89258.1 BCIN|XP_024547651.1 CFRU|XP_031876342.1 CFRU|XP_031880755.1 CFRU|XP_031886128.1 CFRU|XP_031892927.1 CGLO|KAF3798409.1 CGLO|KAF3805426.1 CGLO|KAF3807414.1 CGLO|KAF3809928.1 CHIG|XP_018156240.1 CHIG|XP_018160293.1 CVIN|KAF4918722.1 CVIN|KAF4930405.1 CVIN|KAF4930408.1 CVIN|KAF4930676.1 CVYL|A01696 CVYL|A05556 CVYL|A08220 CVYL|A10894 CVYL|A11227 CVYL|A12769 FGRM|XP_011323194.1 FGRM|XP_011325237.1 FGRM|XP_011328670.1 MORY|QBZ55420.1 MORY|QBZ56106.1 MORY|QBZ60813.1 NCRA|XP_962174.3 SSCL|APA07677.1 SSCL|APA07746.1 SSCL|APA10647.1

>Orthogroup263: ANID|CBF81419.1 BCIN|XP_024545909.1 BCIN|XP_024546366.1 BCIN|XP_024548711.1 BCIN|XP_024553061.1 BGRA|VCU38751.1 BGRA|VCU41385.1 BGRA|VDB89298.1 CFRU|XP_031877135.1 CFRU|XP_031877198.1 CFRU|XP_031890533.1 CGLO|KAF3803972.1 CGLO|KAF3804860.1 CHIG|XP_018162313.1 CHIG|XP_018164392.1 CVIN|KAF4918052.1 CVIN|KAF4921150.1 CVIN|KAF4925148.1 CVYL|A08829 CVYL|A12023 CVYL|A12213 FGRM|XP_011316304.1 FGRM|XP_011321701.1 FGRM|XP_011324126.1 FGRM|XP_011328742.1 MGRA|XP_003851279.1 MGRA|XP_003852097.1 MORY|QBZ55293.1 MORY|QBZ55740.1 NCRA|XP_960090.1 NCRA|XP_964278.3 SSCL|APA06852.1 SSCL|APA07324.1 SSCL|APA09978.1 SSCL|APA16256.1

>Orthogroup264: ANID|CBF81562.1 BCIN|XP_001555114.2 BCIN|XP_001555446.1 BCIN|XP_001561080.1 CFRU|XP_031877062.1 CFRU|XP_031881657.1 CFRU|XP_031883469.1 CFRU|XP_031884748.1 CFRU|XP_031888645.1 CGLO|KAF3799331.1 CGLO|KAF3800999.1 CGLO|KAF3810818.1 CGLO|KAF3811094.1 CHIG|XP_018152982.1 CHIG|XP_018154065.1 CHIG|XP_018154311.1 CHIG|XP_018154976.1 CHIG|XP_018156753.1 CHIG|XP_018163029.1 CVIN|KAF4899855.1 CVIN|KAF4911074.1 CVIN|KAF4912977.1 CVIN|KAF4913623.1 CVIN|KAF4924807.1 CVYL|A02009 CVYL|A04297 CVYL|A05907 CVYL|A10998 FGRM|XP_011317649.1 FGRM|XP_011320758.1 MGRA|XP_003856555.1 MORY|QBZ63435.1 NCRA|XP_958817.1 SSCL|APA06772.1 SSCL|APA08165.1

>Orthogroup265: ANID|CBF84502.1 ANID|CBF89996.1 BCIN|XP_001546355.1 BCIN|XP_001546360.1 BCIN|XP_001548592.2 BCIN|XP_001549079.1 BCIN|XP_024552099.1 CFRU|XP_031876965.1 CFRU|XP_031880608.1 CFRU|XP_031889872.1 CFRU|XP_031889878.1 CGLO|KAF3799142.1 CGLO|KAF3805979.1 CHIG|XP_018154998.1 CHIG|XP_018158950.1 CVIN|KAF4917778.1 CVYL|A13741 FGRM|XP_011315892.1 FGRM|XP_011315893.1 FGRM|XP_011315894.1 FGRM|XP_011315896.1 FGRM|XP_011315897.1 FGRM|XP_011318153.1 FGRM|XP_011318271.1 FGRM|XP_011320832.1 FGRM|XP_011320964.1 FGRM|XP_011322611.1 FGRM|XP_011325906.1 FGRM|XP_011327567.1 MORY|QBZ53307.1 SSCL|APA05382.1 SSCL|APA05386.1 SSCL|APA05388.1 SSCL|APA11747.1 SSCL|APA12927.1

>Orthogroup266: ANID|CBF86203.1 BCIN|XP_001552241.2 BCIN|XP_024548046.1 BCIN|XP_024552664.1 BGRA|VCU39264.1 CFRU|XP_031877395.1 CFRU|XP_031885536.1 CFRU|XP_031889730.1 CFRU|XP_031893276.1 CGLO|KAF3798664.1 CGLO|KAF3808434.1 CGLO|KAF3809543.1 CGLO|KAF3810577.1 CHIG|XP_018157495.1 CHIG|XP_018159032.1 CHIG|XP_018162010.1 CVIN|KAF4914227.1 CVIN|KAF4918567.1 CVIN|KAF4919452.1 CVIN|KAF4931498.1 CVYL|A08479 CVYL|A09403 CVYL|A11709 CVYL|A13657 FGRM|XP_011323552.1 FGRM|XP_011325891.1 MGRA|XP_003847712.1 MLAR|XP_007410978.1 MLAR|XP_007412214.1 MORY|QBZ54841.1 MORY|QBZ56574.1 NCRA|XP_960326.1 NCRA|XP_962289.2 SSCL|APA08283.1 SSCL|APA12909.1

>Orthogroup267: CFRU|XP_031875996.1 CFRU|XP_031876155.1 CFRU|XP_031876966.1 CGLO|KAF3798284.1 CGLO|KAF3802356.1 CGLO|KAF3802951.1 CGLO|KAF3807984.1 CGLO|KAF3812026.1 CHIG|XP_018150487.1 CHIG|XP_018150534.1 CHIG|XP_018150626.1 CHIG|XP_018150632.1 CHIG|XP_018150654.1 CHIG|XP_018150681.1 CHIG|XP_018150715.1 CHIG|XP_018156573.1 CHIG|XP_018156671.1 CVIN|KAF4892660.1 CVIN|KAF4920867.1 CVIN|KAF4922554.1 CVYL|A05437 CVYL|A08515 CVYL|A08658 CVYL|A09922 CVYL|A09941 CVYL|A09978 CVYL|A09992 FGRM|XP_011325774.1 MGRA|XP_003847123.1 MGRA|XP_003847190.1 MORY|QBZ55037.1 MORY|QBZ57601.1 MORY|QBZ65468.1 MORY|QBZ66647.1 MORY|QBZ66704.1

>Orthogroup268: CFRU|XP_031879053.1 CFRU|XP_031883024.1 CFRU|XP_031888161.1 CFRU|XP_031889525.1 CFRU|XP_031889702.1 CGLO|KAF3797617.1 CGLO|KAF3798836.1 CGLO|KAF3805956.1 CGLO|KAF3808228.1 CHIG|XP_018151193.1 CHIG|XP_018153084.1 CHIG|XP_018154124.1 CHIG|XP_018157606.1 CHIG|XP_018158982.1 CHIG|XP_018163183.1 CVIN|KAF4911880.1 CVIN|KAF4914249.1 CVIN|KAF4914259.1 CVIN|KAF4919583.1 CVYL|A00569 CVYL|A03375 CVYL|A09098 CVYL|A13766 FGRM|XP_011325744.1 FGRM|XP_011326187.1 MLAR|XP_007409614.1 MLAR|XP_007412327.1 MLAR|XP_007412381.1 MLAR|XP_007413866.1 MLAR|XP_007413981.1 MORY|QBZ57544.1 MORY|QBZ57831.1 MORY|QBZ60863.1 MORY|QBZ64901.1 NCRA|XP_957734.1

>Orthogroup269: MLAR|XP_007403481.1 MLAR|XP_007404284.1 MLAR|XP_007405560.1 MLAR|XP_007405851.1 MLAR|XP_007406182.1 MLAR|XP_007406346.1 MLAR|XP_007406347.1 MLAR|XP_007409028.1 MLAR|XP_007409029.1 MLAR|XP_007409032.1 MLAR|XP_007409033.1 MLAR|XP_007409112.1 MLAR|XP_007409113.1 MLAR|XP_007409675.1 MLAR|XP_007410198.1 MLAR|XP_007410699.1 MLAR|XP_007410700.1 MLAR|XP_007410984.1 MLAR|XP_007411223.1 MLAR|XP_007411464.1 MLAR|XP_007411465.1 MLAR|XP_007411466.1 MLAR|XP_007411482.1 MLAR|XP_007411612.1 MLAR|XP_007411616.1 MLAR|XP_007412129.1 MLAR|XP_007413196.1 MLAR|XP_007413285.1 MLAR|XP_007413321.1 MLAR|XP_007414108.1 MLAR|XP_007414890.1 MLAR|XP_007415881.1 MLAR|XP_007415882.1 MLAR|XP_007416665.1 MLAR|XP_007418632.1

>Orthogroup270: MLAR|XP_007403492.1 MLAR|XP_007405047.1 MLAR|XP_007405453.1 MLAR|XP_007405651.1 MLAR|XP_007405652.1 MLAR|XP_007405862.1 MLAR|XP_007406248.1 MLAR|XP_007406397.1 MLAR|XP_007406651.1 MLAR|XP_007407047.1 MLAR|XP_007407048.1 MLAR|XP_007407597.1 MLAR|XP_007407617.1 MLAR|XP_007407957.1 MLAR|XP_007408557.1 MLAR|XP_007409984.1 MLAR|XP_007411169.1 MLAR|XP_007411185.1 MLAR|XP_007414855.1 MLAR|XP_007414859.1 MLAR|XP_007414915.1 MLAR|XP_007414972.1 MLAR|XP_007415844.1 MLAR|XP_007415860.1 MLAR|XP_007415861.1 MLAR|XP_007415952.1 MLAR|XP_007415953.1 MLAR|XP_007417332.1 MLAR|XP_007417343.1 MLAR|XP_007418731.1 MLAR|XP_007418807.1 MLAR|XP_007419291.1 MLAR|XP_007419382.1 MLAR|XP_007419383.1 MLAR|XP_007419437.1

>Orthogroup271: MLAR|XP_007405126.1 MLAR|XP_007405429.1 MLAR|XP_007405666.1 MLAR|XP_007405740.1 MLAR|XP_007406044.1 MLAR|XP_007406142.1 MLAR|XP_007406260.1 MLAR|XP_007407956.1 MLAR|XP_007409071.1 MLAR|XP_007409430.1 MLAR|XP_007409619.1 MLAR|XP_007409958.1 MLAR|XP_007410132.1 MLAR|XP_007410216.1 MLAR|XP_007411070.1 MLAR|XP_007411576.1 MLAR|XP_007411694.1 MLAR|XP_007411781.1 MLAR|XP_007411813.1 MLAR|XP_007412287.1 MLAR|XP_007412464.1 MLAR|XP_007413171.1 MLAR|XP_007413200.1 MLAR|XP_007414504.1 MLAR|XP_007414684.1 MLAR|XP_007414760.1 MLAR|XP_007415137.1 MLAR|XP_007415164.1 MLAR|XP_007415259.1 MLAR|XP_007415492.1 MLAR|XP_007416217.1 MLAR|XP_007418158.1 MLAR|XP_007418967.1 MLAR|XP_007419422.1 MLAR|XP_007419475.1

>Orthogroup272: ANID|CBF69357.1 ANID|CBF75938.1 ANID|CBF78377.1 ANID|CBF78415.1 ANID|CBF89352.1 BCIN|XP_001554952.1 CFRU|XP_031878978.1 CFRU|XP_031881917.1 CFRU|XP_031893226.1 CGLO|KAF3806082.1 CGLO|KAF3807886.1 CGLO|KAF3809637.1 CHIG|XP_018152585.1 CHIG|XP_018163086.1 CHIG|XP_018164592.1 CVIN|KAF4896982.1 CVIN|KAF4909971.1 CVIN|KAF4922601.1 CVYL|A00727 CVYL|A07430 CVYL|A08409 FGRM|XP_011322561.1 FGRM|XP_011325148.1 FGRM|XP_011328679.1 MGRA|XP_003852305.1 MLAR|XP_007410428.1 MLAR|XP_007413888.1 MLAR|XP_007413957.1 MLAR|XP_007418082.1 MORY|QBZ63627.1 NCRA|XP_961851.1 NCRA|XP_962432.2 NCRA|XP_963070.3 SSCL|APA08376.1

>Orthogroup273: ANID|CBF70684.1 ANID|CBF70985.1 ANID|CBF73878.1 ANID|CBF76072.1 ANID|CBF76571.1 ANID|CBF77622.1 ANID|CBF78018.1 ANID|CBF78361.1 ANID|CBF79546.1 ANID|CBF82083.1 ANID|CBF83505.1 ANID|CBF84126.1 ANID|CBF84396.1 ANID|CBF86544.1 ANID|CBF87422.1 ANID|CBF88413.1 ANID|CBF88708.1 ANID|CBF89022.1 ANID|CBF89295.1 CFRU|XP_031875509.1 CFRU|XP_031877570.1 CFRU|XP_031882751.1 CHIG|XP_018151194.1 CHIG|XP_018151721.1 CVYL|A09950 MORY|QBZ57609.1 MORY|QBZ57610.1 MORY|QBZ57629.1 MORY|QBZ58273.1 NCRA|XP_956808.1 NCRA|XP_965377.1 SSCL|APA09045.1 SSCL|APA15849.1 SSCL|APA16216.1

>Orthogroup274: ANID|CBF70858.1 ANID|CBF71313.1 ANID|CBF76243.1 BCIN|XP_001553849.1 BCIN|XP_024554015.1 BGRA|VDB92684.1 CFRU|XP_031877767.1 CFRU|XP_031880628.1 CFRU|XP_031892092.1 CGLO|KAF3800112.1 CGLO|KAF3809945.1 CGLO|KAF3810670.1 CHIG|XP_018161525.1 CHIG|XP_018162427.1 CHIG|XP_018165050.1 CVIN|KAF4916953.1 CVIN|KAF4925857.1 CVIN|KAF4930714.1 CVYL|A01678 CVYL|A06803 CVYL|A07291 FGRM|XP_011318385.1 FGRM|XP_011318437.1 MGRA|XP_003848892.1 MGRA|XP_003850344.1 MLAR|XP_007405911.1 MLAR|XP_007405912.1 MLAR|XP_007411343.1 MLAR|XP_007412140.1 MORY|QBZ55707.1 NCRA|XP_011393887.1 NCRA|XP_958408.1 SSCL|APA13271.1 SSCL|APA15334.1

>Orthogroup275: ANID|CBF71445.1 ANID|CBF80140.1 BCIN|XP_001555607.2 CFRU|XP_031878761.1 CFRU|XP_031880315.1 CFRU|XP_031883697.1 CFRU|XP_031884080.1 CFRU|XP_031890796.1 CFRU|XP_031893617.1 CGLO|KAF3797570.1 CGLO|KAF3800011.1 CGLO|KAF3800817.1 CGLO|KAF3808992.1 CGLO|KAF3810851.1 CGLO|KAF3811697.1 CHIG|XP_018155410.1 CHIG|XP_018156550.1 CHIG|XP_018161362.1 CHIG|XP_018161703.1 CHIG|XP_018163172.1 CVIN|KAF4911108.1 CVIN|KAF4920855.1 CVIN|KAF4922468.1 CVIN|KAF4923583.1 CVYL|A01402 CVYL|A02289 CVYL|A08732 CVYL|A08976 CVYL|A10226 FGRM|XP_011320920.1 MORY|QBZ61620.1 NCRA|XP_011393995.1 NCRA|XP_957374.3 NCRA|XP_964644.1

>Orthogroup276: ANID|CBF73993.1 ANID|CBF80176.1 ANID|CBF87761.1 BCIN|XP_001547222.1 BCIN|XP_001549009.1 BCIN|XP_001552683.1 BCIN|XP_001555313.1 BCIN|XP_001557064.1 BCIN|XP_001557135.1 CFRU|XP_031878256.1 CFRU|XP_031883430.1 CFRU|XP_031883801.1 CFRU|XP_031889108.1 CGLO|KAF3798075.1 CGLO|KAF3801770.1 CGLO|KAF3803088.1 CGLO|KAF3805099.1 CHIG|XP_018155581.1 CHIG|XP_018159509.1 CHIG|XP_018163421.1 CHIG|XP_018165005.1 CVIN|KAF4922926.1 CVIN|KAF4928005.1 CVIN|KAF4929600.1 CVYL|A00980 CVYL|A06459 CVYL|A08282 CVYL|A12937 FGRM|XP_011321825.1 MORY|QBZ58342.1 MORY|QBZ64385.1 MORY|QBZ65422.1 NCRA|XP_965605.1 SSCL|APA13194.1

>Orthogroup277: ANID|CBF74263.1 BCIN|XP_001554424.1 BGRA|VDB93722.1 CFRU|XP_031875932.1 CFRU|XP_031876686.1 CFRU|XP_031878618.1 CFRU|XP_031879973.1 CFRU|XP_031881830.1 CFRU|XP_031890079.1 CGLO|KAF3798490.1 CGLO|KAF3798739.1 CGLO|KAF3798973.1 CGLO|KAF3800631.1 CHIG|XP_018151680.1 CHIG|XP_018152053.1 CHIG|XP_018152843.1 CHIG|XP_018159409.1 CVIN|KAF4903200.1 CVIN|KAF4917487.1 CVIN|KAF4918184.1 CVIN|KAF4918702.1 CVIN|KAF4923470.1 CVIN|KAF4923472.1 CVYL|A02367 CVYL|A07891 CVYL|A09172 CVYL|A11171 CVYL|A13085 FGRM|XP_011317217.1 MGRA|XP_003855480.1 MGRA|XP_003856981.1 MORY|QBZ57302.1 NCRA|XP_956274.3 SSCL|APA12619.1

>Orthogroup278: ANID|CBF74979.1 ANID|CBF80191.1 ANID|CBF87511.1 BCIN|XP_001547202.1 CFRU|XP_031883673.1 CFRU|XP_031884682.1 CFRU|XP_031888742.1 CFRU|XP_031889625.1 CFRU|XP_031890114.1 CGLO|KAF3799289.1 CGLO|KAF3799669.1 CGLO|KAF3800229.1 CGLO|KAF3802831.1 CGLO|KAF3810643.1 CHIG|XP_018154745.1 CHIG|XP_018154901.1 CHIG|XP_018155073.1 CHIG|XP_018158981.1 CHIG|XP_018159873.1 CVIN|KAF4895372.1 CVIN|KAF4905806.1 CVIN|KAF4917795.1 CVIN|KAF4921795.1 CVIN|KAF4924788.1 CVYL|A03640 CVYL|A05181 CVYL|A05832 CVYL|A12404 CVYL|A13727 FGRM|XP_011322623.1 FGRM|XP_011322940.1 MORY|QBZ53569.1 MORY|QBZ64405.1 NCRA|XP_956437.1

>Orthogroup279: ANID|CBF75886.1 ANID|CBF80421.1 ANID|CBF80424.1 ANID|CBF87262.1 BCIN|XP_001547340.2 CFRU|XP_031882934.1 CFRU|XP_031884189.1 CFRU|XP_031884651.1 CFRU|XP_031890997.1 CFRU|XP_031892307.1 CGLO|KAF3801519.1 CGLO|KAF3806648.1 CGLO|KAF3807909.1 CGLO|KAF3811029.1 CHIG|XP_018152620.1 CHIG|XP_018153003.1 CHIG|XP_018158306.1 CHIG|XP_018159926.1 CVIN|KAF4898147.1 CVIN|KAF4898618.1 CVIN|KAF4913291.1 CVIN|KAF4918150.1 CVYL|A05800 CVYL|A09761 CVYL|A10566 CVYL|A12614 FGRM|XP_011327429.1 MGRA|XP_003853592.1 MGRA|XP_003854462.1 MORY|QBZ60709.1 MORY|QBZ62397.1 MORY|QBZ64598.1 MORY|QBZ65170.1 SSCL|APA09122.1

>Orthogroup280: ANID|CBF77312.1 ANID|CBF78417.1 ANID|CBF80813.1 ANID|CBF84491.1 BCIN|XP_001554951.1 BCIN|XP_001559251.1 CFRU|XP_031883456.1 CFRU|XP_031885806.1 CFRU|XP_031886656.1 CFRU|XP_031889464.1 CGLO|KAF3800218.1 CGLO|KAF3801195.1 CGLO|KAF3808876.1 CGLO|KAF3810231.1 CHIG|XP_018155121.1 CHIG|XP_018155199.1 CHIG|XP_018162503.1 CVIN|KAF4920416.1 CVIN|KAF4929094.1 CVIN|KAF4932098.1 CVYL|A01206 CVYL|A02830 CVYL|A06068 CVYL|A07139 FGRM|XP_011322030.1 FGRM|XP_011322383.1 MGRA|XP_003850471.1 MGRA|XP_003851936.1 MORY|QBZ55868.1 MORY|QBZ64989.1 NCRA|XP_955806.1 NCRA|XP_957687.1 SSCL|APA08375.1 SSCL|APA08940.1

>Orthogroup281: ANID|CBF78247.1 BCIN|XP_024547801.1 BCIN|XP_024550753.1 CFRU|XP_031879329.1 CFRU|XP_031882669.1 CFRU|XP_031883547.1 CFRU|XP_031885050.1 CFRU|XP_031888704.1 CFRU|XP_031889364.1 CFRU|XP_031892169.1 CGLO|KAF3799231.1 CGLO|KAF3803875.1 CGLO|KAF3807698.1 CGLO|KAF3808656.1 CGLO|KAF3809800.1 CGLO|KAF3810874.1 CHIG|XP_018156512.1 CHIG|XP_018162933.1 CVIN|KAF4909300.1 CVIN|KAF4916367.1 CVIN|KAF4918875.1 CVIN|KAF4919308.1 CVIN|KAF4925425.1 CVYL|A02271 CVYL|A06719 CVYL|A07253 CVYL|A08925 CVYL|A10123 FGRM|XP_011318123.1 FGRM|XP_011328751.1 MORY|QBZ59342.1 NCRA|XP_957711.3 SSCL|APA08076.1 SSCL|APA11663.1

>Orthogroup282: ANID|CBF78465.1 ANID|CBF89406.1 BCIN|XP_024551133.1 CFRU|XP_031878282.1 CFRU|XP_031885284.1 CFRU|XP_031890057.1 CGLO|KAF3798063.1 CGLO|KAF3798756.1 CGLO|KAF3807798.1 CHIG|XP_018158158.1 CVIN|KAF4903207.1 CVIN|KAF4917906.1 CVIN|KAF4921515.1 CVIN|KAF4927970.1 CVYL|A00804 CVYL|A08294 CVYL|A09163 FGRM|XP_011319620.1 FGRM|XP_011321819.1 FGRM|XP_011322339.1 MGRA|XP_003847905.1 MGRA|XP_003850684.1 MGRA|XP_003852313.1 MGRA|XP_003852808.1 MLAR|XP_007403866.1 MLAR|XP_007416084.1 MORY|QBZ53512.1 MORY|QBZ56745.1 MORY|QBZ60627.1 MORY|QBZ63990.1 MORY|QBZ64236.1 MORY|QBZ64500.1 NCRA|XP_960539.2 NCRA|XP_960658.1

>Orthogroup283: ANID|CBF79959.1 BCIN|XP_024548373.1 BCIN|XP_024549834.1 BCIN|XP_024550346.1 BGRA|VDB85994.1 CFRU|XP_031875617.1 CFRU|XP_031876209.1 CFRU|XP_031876210.1 CFRU|XP_031876324.1 CFRU|XP_031880417.1 CFRU|XP_031883729.1 CFRU|XP_031892475.1 CFRU|XP_031892476.1 CGLO|KAF3807311.1 CGLO|KAF3807652.1 CHIG|XP_018156986.1 CVIN|KAF4918092.1 CVIN|KAF4918095.1 CVIN|KAF4920689.1 CVYL|A10423 CVYL|A10798 FGRM|XP_011326170.1 FGRM|XP_011328329.1 MLAR|XP_007406083.1 MORY|QBZ57627.1 MORY|QBZ57628.1 MORY|QBZ58510.1 MORY|QBZ65869.1 NCRA|XP_965751.3 SSCL|APA09150.1 SSCL|APA11026.1 SSCL|APA11283.1 SSCL|APA13355.1 SSCL|APA13395.1

>Orthogroup284: ANID|CBF80410.1 CFRU|XP_031879917.1 CFRU|XP_031880105.1 CFRU|XP_031893528.1 CGLO|KAF3798948.1 CGLO|KAF3809475.1 CGLO|KAF3811806.1 CHIG|XP_018151726.1 CHIG|XP_018152697.1 CHIG|XP_018152876.1 CVIN|KAF4921271.1 CVIN|KAF4923507.1 CVIN|KAF4924879.1 CVYL|A07864 CVYL|A10042 CVYL|A12810 FGRM|XP_011319515.1 FGRM|XP_011322469.1 FGRM|XP_011326173.1 FGRM|XP_011328478.1 MLAR|XP_007404840.1 MLAR|XP_007404853.1 MLAR|XP_007406256.1 MLAR|XP_007407276.1 MLAR|XP_007411904.1 MLAR|XP_007417367.1 MLAR|XP_007418493.1 MLAR|XP_007418494.1 MLAR|XP_007418507.1 MLAR|XP_007418508.1 MORY|QBZ55116.1 MORY|QBZ66758.1 NCRA|XP_961783.2 NCRA|XP_963236.2

>Orthogroup285: ANID|CBF82047.1 ANID|CBF82525.1 ANID|CBF83535.1 BCIN|XP_001550900.1 BCIN|XP_001552823.2 BCIN|XP_024554064.1 CFRU|XP_031875888.1 CFRU|XP_031881525.1 CFRU|XP_031884159.1 CFRU|XP_031891039.1 CGLO|KAF3801628.1 CGLO|KAF3802663.1 CGLO|KAF3805718.1 CHIG|XP_018151392.1 CHIG|XP_018157718.1 CHIG|XP_018163635.1 CVIN|KAF4895168.1 CVIN|KAF4921202.1 CVIN|KAF4926637.1 CVYL|A09827 CVYL|A12509 CVYL|A13601 FGRM|XP_011315859.1 FGRM|XP_011317515.1 MGRA|XP_003852319.1 MGRA|XP_003854112.1 MGRA|XP_003855928.1 MGRA|XP_003857485.1 MLAR|XP_007417256.1 MORY|QBZ63106.1 NCRA|XP_963274.1 SSCL|APA07603.1 SSCL|APA12395.1 SSCL|APA15451.1

>Orthogroup286: ANID|CBF82817.1 ANID|CBF83072.1 ANID|CBF86619.1 BCIN|XP_001550345.1 BCIN|XP_001551253.2 BCIN|XP_001551761.1 BGRA|VDB89854.1 CFRU|XP_031875490.1 CFRU|XP_031875502.1 CFRU|XP_031875517.1 CFRU|XP_031875656.1 CFRU|XP_031877583.1 CFRU|XP_031882988.1 CFRU|XP_031888728.1 CFRU|XP_031890584.1 CGLO|KAF3800771.1 CGLO|KAF3804134.1 CGLO|KAF3806714.1 CHIG|XP_018158690.1 CHIG|XP_018159976.1 CVIN|KAF4910272.1 CVIN|KAF4919475.1 CVIN|KAF4919655.1 CVYL|A00433 CVYL|A03607 CVYL|A14021 FGRM|XP_011326724.1 FGRM|XP_011328265.1 MGRA|XP_003852461.1 MORY|QBZ60464.1 NCRA|XP_001728262.2 SSCL|APA10048.1 SSCL|APA11991.1 SSCL|APA13246.1

>Orthogroup287: ANID|CBF84834.1 ANID|CBF86439.1 BCIN|XP_001552353.1 BCIN|XP_024546369.1 BGRA|VDB86064.1 CFRU|XP_031877150.1 CFRU|XP_031881422.1 CFRU|XP_031885627.1 CFRU|XP_031888016.1 CGLO|KAF3802094.1 CGLO|KAF3803968.1 CGLO|KAF3805724.1 CGLO|KAF3810138.1 CHIG|XP_018154243.1 CHIG|XP_018157971.1 CHIG|XP_018163627.1 CVIN|KAF4912154.1 CVIN|KAF4917968.1 CVIN|KAF4925143.1 CVIN|KAF4926586.1 CVYL|A02729 CVYL|A03296 CVYL|A08833 CVYL|A09819 FGRM|XP_011320314.1 MGRA|XP_003849875.1 MGRA|XP_003852894.1 MGRA|XP_003855740.1 MORY|QBZ53606.1 MORY|QBZ59905.1 MORY|QBZ64744.1 NCRA|XP_958633.1 SSCL|APA06844.1 SSCL|APA11179.1

>Orthogroup288: BCIN|XP_001546261.1 BCIN|XP_001550232.1 BCIN|XP_001552498.1 BCIN|XP_001560376.1 CFRU|XP_031876021.1 CFRU|XP_031878804.1 CFRU|XP_031883982.1 CFRU|XP_031893046.1 CGLO|KAF3797305.1 CGLO|KAF3797497.1 CGLO|KAF3798374.1 CGLO|KAF3805108.1 CHIG|XP_018152735.1 CHIG|XP_018157204.1 CHIG|XP_018161276.1 CVIN|KAF4899571.1 CVIN|KAF4921625.1 CVIN|KAF4922847.1 CVIN|KAF4927393.1 CVYL|A01946 CVYL|A06450 CVYL|A08199 CVYL|A10390 FGRM|XP_011317952.1 FGRM|XP_011320199.1 FGRM|XP_011322106.1 MORY|QBZ65603.1 NCRA|XP_958697.1 NCRA|XP_963407.1 NCRA|XP_963408.2 SSCL|APA08792.1 SSCL|APA10258.1 SSCL|APA10765.1 SSCL|APA16345.1

>Orthogroup289: BGRA|VCU38836.1 BGRA|VCU38974.1 BGRA|VCU39152.1 BGRA|VCU39882.1 BGRA|VCU40269.1 BGRA|VCU40673.1 BGRA|VCU40984.1 BGRA|VCU41001.1 BGRA|VCU41192.1 BGRA|VDB83986.1 BGRA|VDB85892.1 BGRA|VDB87719.1 BGRA|VDB88045.1 BGRA|VDB88092.1 BGRA|VDB89039.1 BGRA|VDB89041.1 BGRA|VDB89042.1 BGRA|VDB89055.1 BGRA|VDB89091.1 BGRA|VDB89628.1 BGRA|VDB89755.1 BGRA|VDB89881.1 BGRA|VDB91367.1 BGRA|VDB92443.1 BGRA|VDB92635.1 BGRA|VDB92736.1 BGRA|VDB93077.1 BGRA|VDB94450.1 BGRA|VDB94465.1 BGRA|VDB94495.1 BGRA|VDB94508.1 BGRA|VDB94624.1 BGRA|VDB94676.1 BGRA|VDB94694.1

>Orthogroup290: BGRA|VCU39199.1 BGRA|VCU39310.1 BGRA|VCU39560.1 BGRA|VCU39567.1 BGRA|VCU39995.1 BGRA|VCU40047.1 BGRA|VCU40164.1 BGRA|VCU40739.1 BGRA|VCU40867.1 BGRA|VCU41375.1 BGRA|VDB83566.1 BGRA|VDB83591.1 BGRA|VDB83685.1 BGRA|VDB85768.1 BGRA|VDB85898.1 BGRA|VDB86140.1 BGRA|VDB86387.1 BGRA|VDB87975.1 BGRA|VDB88032.1 BGRA|VDB88152.1 BGRA|VDB88244.1 BGRA|VDB88759.1 BGRA|VDB88935.1 BGRA|VDB89205.1 BGRA|VDB89280.1 BGRA|VDB89781.1 BGRA|VDB89789.1 BGRA|VDB89807.1 BGRA|VDB92770.1 BGRA|VDB92816.1 BGRA|VDB92935.1 BGRA|VDB93611.1 BGRA|VDB94856.1 BGRA|VDB94979.1

>Orthogroup291: CVIN|KAF4924071.1 MLAR|XP_007403543.1 MLAR|XP_007403544.1 MLAR|XP_007403872.1 MLAR|XP_007405192.1 MLAR|XP_007407731.1 MLAR|XP_007407732.1 MLAR|XP_007408363.1 MLAR|XP_007408738.1 MLAR|XP_007408888.1 MLAR|XP_007408889.1 MLAR|XP_007411549.1 MLAR|XP_007412143.1 MLAR|XP_007412757.1 MLAR|XP_007412805.1 MLAR|XP_007413009.1 MLAR|XP_007414493.1 MLAR|XP_007414494.1 MLAR|XP_007417375.1 MLAR|XP_007417643.1 MLAR|XP_007417644.1 MLAR|XP_007418229.1 MLAR|XP_007418231.1 MLAR|XP_007418727.1 MLAR|XP_007418728.1 MLAR|XP_007418760.1 MLAR|XP_007418834.1 MLAR|XP_007418841.1 MLAR|XP_007419010.1 MLAR|XP_007419013.1 MLAR|XP_007419113.1 MLAR|XP_007419185.1 MLAR|XP_007419579.1 SSCL|APA16180.1

>Orthogroup292: MLAR|XP_007403613.1 MLAR|XP_007403698.1 MLAR|XP_007404147.1 MLAR|XP_007405291.1 MLAR|XP_007405430.1 MLAR|XP_007405667.1 MLAR|XP_007406045.1 MLAR|XP_007406091.1 MLAR|XP_007406162.1 MLAR|XP_007406261.1 MLAR|XP_007406500.1 MLAR|XP_007407747.1 MLAR|XP_007407917.1 MLAR|XP_007407960.1 MLAR|XP_007408453.1 MLAR|XP_007408561.1 MLAR|XP_007408644.1 MLAR|XP_007408895.1 MLAR|XP_007409109.1 MLAR|XP_007409318.1 MLAR|XP_007409415.1 MLAR|XP_007411782.1 MLAR|XP_007413990.1 MLAR|XP_007414623.1 MLAR|XP_007414761.1 MLAR|XP_007415260.1 MLAR|XP_007415552.1 MLAR|XP_007416218.1 MLAR|XP_007416731.1 MLAR|XP_007418131.1 MLAR|XP_007418274.1 MLAR|XP_007418854.1 MLAR|XP_007419359.1 MLAR|XP_007419499.1

>Orthogroup293: MLAR|XP_007404895.1 MLAR|XP_007404940.1 MLAR|XP_007405510.1 MLAR|XP_007406537.1 MLAR|XP_007406541.1 MLAR|XP_007407303.1 MLAR|XP_007408887.1 MLAR|XP_007408894.1 MLAR|XP_007411103.1 MLAR|XP_007412127.1 MLAR|XP_007413005.1 MLAR|XP_007413006.1 MLAR|XP_007413539.1 MLAR|XP_007413654.1 MLAR|XP_007413987.1 MLAR|XP_007414475.1 MLAR|XP_007414548.1 MLAR|XP_007415142.1 MLAR|XP_007415409.1 MLAR|XP_007415536.1 MLAR|XP_007415945.1 MLAR|XP_007416980.1 MLAR|XP_007417388.1 MLAR|XP_007418032.1 MLAR|XP_007418033.1 MLAR|XP_007418096.1 MLAR|XP_007418410.1 MLAR|XP_007418446.1 MLAR|XP_007418779.1 MLAR|XP_007419181.1 MLAR|XP_007419266.1 MLAR|XP_007419267.1 MLAR|XP_007419480.1 MLAR|XP_007419575.1

>Orthogroup294: ANID|CBF69451.1 ANID|CBF73494.1 ANID|CBF80428.1 ANID|CBF82812.1 ANID|CBF83139.1 ANID|CBF86046.1 ANID|CBF88295.1 ANID|CBF89312.1 BCIN|XP_024547379.1 BCIN|XP_024547562.1 BCIN|XP_024548101.1 BCIN|XP_024550097.1 BCIN|XP_024554044.1 CFRU|XP_031883769.1 CFRU|XP_031891921.1 CGLO|KAF3801387.1 CGLO|KAF3805179.1 CHIG|XP_018151663.1 CHIG|XP_018154037.1 CHIG|XP_018154091.1 CHIG|XP_018158294.1 CHIG|XP_018158298.1 CHIG|XP_018160085.1 CVIN|KAF4927413.1 CVYL|A00313 CVYL|A05680 CVYL|A06574 CVYL|A07696 FGRM|XP_011320993.1 MORY|QBZ53539.1 MORY|QBZ61777.1 SSCL|APA09955.1 SSCL|APA16268.1

>Orthogroup295: ANID|CBF69639.1 ANID|CBF73765.1 BCIN|XP_024546167.1 BCIN|XP_024549953.1 BCIN|XP_024553826.1 BGRA|VDB93431.1 BGRA|VDB93584.1 CFRU|XP_031876195.1 CFRU|XP_031876291.1 CFRU|XP_031877176.1 CFRU|XP_031886336.1 CGLO|KAF3806409.1 CHIG|XP_018162291.1 CHIG|XP_018162292.1 CVIN|KAF4923258.1 CVIN|KAF4923285.1 CVYL|A12002 FGRM|XP_011326577.1 FGRM|XP_011326578.1 MGRA|XP_003850312.1 MGRA|XP_003853893.1 MLAR|XP_007410531.1 MLAR|XP_007411410.1 MLAR|XP_007412666.1 MLAR|XP_007415412.1 MLAR|XP_007415416.1 MLAR|XP_007416452.1 MLAR|XP_007419684.1 MORY|QBZ63906.1 NCRA|XP_956165.3 NCRA|XP_956166.2 SSCL|APA05293.1 SSCL|APA15249.1

>Orthogroup296: ANID|CBF71186.1 ANID|CBF77661.1 ANID|CBF80314.1 BCIN|XP_001550077.1 BCIN|XP_001552698.1 BCIN|XP_024546447.1 BCIN|XP_024547538.1 BCIN|XP_024548409.1 BCIN|XP_024552770.1 CFRU|XP_031889324.1 CFRU|XP_031891393.1 CFRU|XP_031893460.1 CGLO|KAF3803093.1 CGLO|KAF3804596.1 CGLO|KAF3809583.1 CHIG|XP_018152603.1 CHIG|XP_018155590.1 CHIG|XP_018161671.1 CVIN|KAF4919202.1 CVIN|KAF4923083.1 CVIN|KAF4929554.1 CVYL|A00985 CVYL|A05282 CVYL|A08448 FGRM|XP_011322566.1 FGRM|XP_011325357.1 MORY|QBZ55820.1 NCRA|XP_959790.3 SSCL|APA07112.1 SSCL|APA07504.1 SSCL|APA12288.1 SSCL|APA14102.1 SSCL|APA16047.1

>Orthogroup297: ANID|CBF71681.1 ANID|CBF82188.1 BCIN|XP_024550302.1 BCIN|XP_024551222.1 CFRU|XP_031875982.1 CFRU|XP_031877438.1 CFRU|XP_031886463.1 CFRU|XP_031888915.1 CGLO|KAF3804289.1 CGLO|KAF3808449.1 CHIG|XP_018150614.1 CHIG|XP_018160167.1 CHIG|XP_018162171.1 CVIN|KAF4915204.1 CVIN|KAF4919513.1 CVIN|KAF4920141.1 CVIN|KAF4931483.1 CVYL|A03973 CVYL|A11692 CVYL|A11774 CVYL|A14061 FGRM|XP_011322246.1 FGRM|XP_011324069.1 MGRA|XP_003855426.1 MLAR|XP_007410430.1 MLAR|XP_007411020.1 MLAR|XP_007411815.1 MLAR|XP_007418300.1 MORY|QBZ59580.1 NCRA|XP_011393932.1 NCRA|XP_956692.2 SSCL|APA10120.1 SSCL|APA10965.1

>Orthogroup298: ANID|CBF73870.1 ANID|CBF78290.1 BCIN|XP_024547734.1 BCIN|XP_024550840.1 CFRU|XP_031878217.1 CFRU|XP_031880989.1 CFRU|XP_031885412.1 CGLO|KAF3799425.1 CGLO|KAF3800458.1 CGLO|KAF3808472.1 CHIG|XP_018150710.1 CHIG|XP_018152297.1 CHIG|XP_018155550.1 CHIG|XP_018161367.1 CHIG|XP_018161950.1 CVIN|KAF4915216.1 CVIN|KAF4931733.1 CVYL|A11669 CVYL|A13289 FGRM|XP_011322488.1 FGRM|XP_011323661.1 FGRM|XP_011325204.1 MGRA|XP_003847275.1 MGRA|XP_003847299.1 MGRA|XP_003847693.1 MGRA|XP_003854880.1 MORY|QBZ56915.1 MORY|QBZ64783.1 MORY|QBZ64784.1 MORY|QBZ65109.1 NCRA|XP_011393549.1 NCRA|XP_959312.2 SSCL|APA07984.1

>Orthogroup299: ANID|CBF74187.1 ANID|CBF80165.1 ANID|CBF81654.1 BCIN|XP_024547006.1 BCIN|XP_024547023.1 CFRU|XP_031876684.1 CFRU|XP_031880827.1 CFRU|XP_031883672.1 CFRU|XP_031889007.1 CGLO|KAF3800230.1 CGLO|KAF3801912.1 CGLO|KAF3804195.1 CGLO|KAF3806914.1 CHIG|XP_018155074.1 CHIG|XP_018159428.1 CHIG|XP_018159596.1 CVIN|KAF4890284.1 CVIN|KAF4918386.1 CVIN|KAF4921794.1 CVYL|A02557 CVYL|A03870 CVYL|A12405 FGRM|XP_011320181.1 FGRM|XP_011320913.1 FGRM|XP_011323160.1 MGRA|XP_003849409.1 MORY|QBZ55175.1 MORY|QBZ59163.1 MORY|QBZ59165.1 MORY|QBZ66193.1 NCRA|XP_958451.2 SSCL|APA05881.1 SSCL|APA08470.1

>Orthogroup300: ANID|CBF75954.1 ANID|CBF78736.1 BCIN|XP_024550245.1 BCIN|XP_024552715.1 CFRU|XP_031881290.1 CFRU|XP_031884205.1 CFRU|XP_031885041.1 CFRU|XP_031886490.1 CFRU|XP_031890794.1 CFRU|XP_031893001.1 CGLO|KAF3806243.1 CGLO|KAF3807693.1 CGLO|KAF3809671.1 CGLO|KAF3810971.1 CGLO|KAF3811017.1 CGLO|KAF3811689.1 CHIG|XP_018151832.1 CHIG|XP_018162080.1 CVIN|KAF4915943.1 CVIN|KAF4921837.1 CVIN|KAF4925038.1 CVIN|KAF4926895.1 CVIN|KAF4927834.1 CVIN|KAF4931447.1 CVYL|A02178 CVYL|A06886 CVYL|A09601 CVYL|A09771 CVYL|A11832 CVYL|A12714 FGRM|XP_011322355.1 MGRA|XP_003857809.1 SSCL|APA10650.1

>Orthogroup301: ANID|CBF76682.1 ANID|CBF78891.1 ANID|CBF85353.1 BCIN|XP_024547440.1 BCIN|XP_024548911.1 CFRU|XP_031876058.1 CFRU|XP_031878880.1 CFRU|XP_031880175.1 CFRU|XP_031881010.1 CFRU|XP_031891674.1 CGLO|KAF3800125.1 CGLO|KAF3801997.1 CGLO|KAF3802626.1 CGLO|KAF3809876.1 CHIG|XP_018159437.1 CHIG|XP_018161297.1 CHIG|XP_018161439.1 CVIN|KAF4905505.1 CVIN|KAF4907635.1 CVIN|KAF4919759.1 CVIN|KAF4925845.1 CVIN|KAF4930718.1 CVYL|A01741 CVYL|A01922 CVYL|A02428 CVYL|A06812 FGRM|XP_011321402.1 FGRM|XP_011322535.1 MLAR|XP_007403662.1 MLAR|XP_007419077.1 MORY|QBZ54105.1 MORY|QBZ59987.1 SSCL|APA15976.1

>Orthogroup302: ANID|CBF78974.1 ANID|CBF82132.1 ANID|CBF88938.1 CFRU|XP_031883199.1 CFRU|XP_031888655.1 CFRU|XP_031888757.1 CFRU|XP_031889095.1 CFRU|XP_031889883.1 CFRU|XP_031891962.1 CGLO|KAF3799185.1 CGLO|KAF3801245.1 CGLO|KAF3804161.1 CGLO|KAF3805962.1 CGLO|KAF3808159.1 CGLO|KAF3808897.1 CHIG|XP_018159793.1 CHIG|XP_018160000.1 CHIG|XP_018164879.1 CVIN|KAF4914904.1 CVIN|KAF4917794.1 CVIN|KAF4918400.1 CVIN|KAF4921955.1 CVIN|KAF4924307.1 CVYL|A00510 CVYL|A01188 CVYL|A03833 CVYL|A06602 CVYL|A10081 CVYL|A13759 FGRM|XP_011319696.1 FGRM|XP_011320865.1 FGRM|XP_011328720.1 MORY|QBZ53531.1

>Orthogroup303: ANID|CBF79705.1 ANID|CBF80673.1 ANID|CBF83908.1 BCIN|XP_024546889.1 BCIN|XP_024550679.1 CFRU|XP_031878667.1 CFRU|XP_031880246.1 CFRU|XP_031887282.1 CFRU|XP_031892763.1 CGLO|KAF3797080.1 CGLO|KAF3797545.1 CGLO|KAF3808963.1 CGLO|KAF3809200.1 CHIG|XP_018150533.1 CHIG|XP_018151196.1 CHIG|XP_018156040.1 CHIG|XP_018157028.1 CVIN|KAF4911394.1 CVIN|KAF4920718.1 CVIN|KAF4925311.1 CVYL|A01389 CVYL|A10628 FGRM|XP_011319118.1 FGRM|XP_011321423.1 MGRA|XP_003849228.1 MGRA|XP_003854237.1 MGRA|XP_003855916.1 MLAR|XP_007409531.1 MLAR|XP_007409767.1 MORY|QBZ57070.1 MORY|QBZ64031.1 NCRA|XP_960692.1 SSCL|APA05850.1

>Orthogroup304: ANID|CBF80264.1 ANID|CBF86030.1 ANID|CBF89124.1 BCIN|XP_001554419.2 BCIN|XP_024547570.1 BCIN|XP_024548107.1 BCIN|XP_024548640.1 BCIN|XP_024552828.1 CFRU|XP_031876252.1 CFRU|XP_031878481.1 CFRU|XP_031888068.1 CFRU|XP_031890675.1 CGLO|KAF3798632.1 CGLO|KAF3802337.1 CHIG|XP_018150645.1 CHIG|XP_018151589.1 CHIG|XP_018151775.1 CHIG|XP_018151776.1 CHIG|XP_018153281.1 CHIG|XP_018153282.1 CHIG|XP_018153894.1 CHIG|XP_018162654.1 CVYL|A06645 FGRM|XP_011321042.1 FGRM|XP_011322699.1 FGRM|XP_011325619.1 FGRM|XP_011327514.1 NCRA|XP_001728229.2 NCRA|XP_959887.1 SSCL|APA08684.1 SSCL|APA11509.1 SSCL|APA15266.1 SSCL|APA16012.1

>Orthogroup305: ANID|CBF80783.1 ANID|CBF84621.1 ANID|CBF88981.1 BCIN|XP_024547679.1 BCIN|XP_024552879.1 BGRA|VCU39610.1 CFRU|XP_031876064.1 CFRU|XP_031882985.1 CFRU|XP_031889473.1 CFRU|XP_031893604.1 CGLO|KAF3800760.1 CGLO|KAF3800866.1 CGLO|KAF3801994.1 CGLO|KAF3808836.1 CHIG|XP_018150833.1 CHIG|XP_018155739.1 CHIG|XP_018159513.1 CVIN|KAF4909985.1 CVIN|KAF4915570.1 CVIN|KAF4919664.1 CVIN|KAF4919756.1 CVYL|A00444 CVYL|A01245 CVYL|A02425 CVYL|A11105 FGRM|XP_011318919.1 FGRM|XP_011321581.1 MGRA|XP_003852346.1 MGRA|XP_003857442.1 MORY|QBZ53861.1 MORY|QBZ65241.1 NCRA|XP_961414.2 SSCL|APA16101.1

>Orthogroup306: ANID|CBF83201.1 ANID|CBF86907.1 BCIN|XP_001554309.1 CFRU|XP_031875601.1 CFRU|XP_031888872.1 CFRU|XP_031889341.1 CGLO|KAF3799215.1 CGLO|KAF3803113.1 CGLO|KAF3804762.1 CHIG|XP_018155608.1 CHIG|XP_018159834.1 CHIG|XP_018159848.1 CVIN|KAF4909313.1 CVIN|KAF4926127.1 CVIN|KAF4926130.1 CVIN|KAF4928322.1 CVYL|A01001 CVYL|A10111 CVYL|A11600 FGRM|XP_011315695.1 FGRM|XP_011322480.1 FGRM|XP_011327485.1 MGRA|XP_003847546.1 MGRA|XP_003852361.1 MGRA|XP_003855022.1 MORY|QBZ55844.1 MORY|QBZ58582.1 MORY|QBZ62704.1 NCRA|XP_958570.3 NCRA|XP_964353.2 SSCL|APA14396.1 SSCL|APA14397.1 SSCL|APA14398.1

>Orthogroup307: ANID|CBF85759.1 ANID|CBF86628.1 BCIN|XP_001548904.1 CFRU|XP_031878087.1 CFRU|XP_031885863.1 CFRU|XP_031889626.1 CFRU|XP_031889880.1 CFRU|XP_031892630.1 CFRU|XP_031893181.1 CGLO|KAF3801841.1 CGLO|KAF3805977.1 CGLO|KAF3806601.1 CGLO|KAF3809635.1 CGLO|KAF3810515.1 CHIG|XP_018158948.1 CHIG|XP_018159106.1 CVIN|KAF4896989.1 CVIN|KAF4901590.1 CVIN|KAF4914573.1 CVIN|KAF4916898.1 CVIN|KAF4917797.1 CVIN|KAF4928124.1 CVYL|A08407 CVYL|A09265 CVYL|A10708 CVYL|A13596 CVYL|A13743 FGRM|XP_011320780.1 MGRA|XP_003854277.1 MORY|QBZ59928.1 MORY|QBZ65573.1 NCRA|XP_957478.1 SSCL|APA14481.1

>Orthogroup308: ANID|CBF86658.1 BCIN|XP_024547715.1 CFRU|XP_031878754.1 CFRU|XP_031879095.1 CFRU|XP_031882545.1 CFRU|XP_031888906.1 CFRU|XP_031890832.1 CFRU|XP_031893011.1 CGLO|KAF3797542.1 CGLO|KAF3797544.1 CGLO|KAF3797547.1 CGLO|KAF3797548.1 CGLO|KAF3797549.1 CGLO|KAF3797550.1 CGLO|KAF3803871.1 CGLO|KAF3804206.1 CGLO|KAF3809485.1 CGLO|KAF3809486.1 CGLO|KAF3811737.1 CHIG|XP_018151800.1 CVIN|KAF4909769.1 CVIN|KAF4920318.1 CVIN|KAF4922990.1 CVIN|KAF4923545.1 CVIN|KAF4925420.1 CVYL|A03881 CVYL|A08928 CVYL|A08997 CVYL|A09053 CVYL|A10053 CVYL|A12753 MORY|QBZ59029.1 MORY|QBZ62723.1

>Orthogroup309: BCIN|XP_001549529.1 BCIN|XP_001559450.1 BCIN|XP_001561161.1 BGRA|VDB90357.1 CFRU|XP_031877943.1 CFRU|XP_031878836.1 CFRU|XP_031881258.1 CGLO|KAF3802528.1 CGLO|KAF3802579.1 CGLO|KAF3810910.1 CHIG|XP_018154834.1 CHIG|XP_018154930.1 CHIG|XP_018161331.1 CVIN|KAF4893533.1 CVIN|KAF4917595.1 CVIN|KAF4921851.1 CVYL|A01864 CVYL|A02130 CVYL|A02235 FGRM|XP_011324642.1 FGRM|XP_011327654.1 FGRM|XP_011327951.1 FGRM|XP_011328085.1 MLAR|XP_007407016.1 MORY|QBZ53952.1 MORY|QBZ57766.1 MORY|QBZ64265.1 NCRA|XP_957065.1 NCRA|XP_962190.2 NCRA|XP_963203.1 SSCL|APA07659.1 SSCL|APA08348.1 SSCL|APA13567.1

>Orthogroup310: CFRU|XP_031881385.1 CFRU|XP_031883309.1 CFRU|XP_031885175.1 CFRU|XP_031886889.1 CFRU|XP_031886896.1 CFRU|XP_031886965.1 CGLO|KAF3800369.1 CGLO|KAF3801759.1 CGLO|KAF3805793.1 CGLO|KAF3811302.1 CGLO|KAF3811326.1 CHIG|XP_018162865.1 CHIG|XP_018163690.1 CVIN|KAF4926614.1 CVIN|KAF4927249.1 CVIN|KAF4927266.1 CVIN|KAF4927799.1 CVYL|A04480 CVYL|A04507 CVYL|A04509 CVYL|A07742 MORY|QBZ53462.1 MORY|QBZ57556.1 MORY|QBZ57794.1 MORY|QBZ57826.1 MORY|QBZ58459.1 MORY|QBZ61156.1 MORY|QBZ63923.1 MORY|QBZ66233.1 NCRA|XP_958717.1 NCRA|XP_960284.1 NCRA|XP_961405.2 SSCL|APA12662.1

>Orthogroup311: MLAR|XP_007403816.1 MLAR|XP_007405198.1 MLAR|XP_007405405.1 MLAR|XP_007405637.1 MLAR|XP_007405645.1 MLAR|XP_007408248.1 MLAR|XP_007408354.1 MLAR|XP_007409191.1 MLAR|XP_007409514.1 MLAR|XP_007411013.1 MLAR|XP_007411085.1 MLAR|XP_007411698.1 MLAR|XP_007412065.1 MLAR|XP_007412402.1 MLAR|XP_007412429.1 MLAR|XP_007412473.1 MLAR|XP_007412736.1 MLAR|XP_007412784.1 MLAR|XP_007413679.1 MLAR|XP_007414247.1 MLAR|XP_007414258.1 MLAR|XP_007414278.1 MLAR|XP_007414564.1 MLAR|XP_007414566.1 MLAR|XP_007416105.1 MLAR|XP_007416114.1 MLAR|XP_007416253.1 MLAR|XP_007417095.1 MLAR|XP_007417959.1 MLAR|XP_007418449.1 MLAR|XP_007418574.1 MLAR|XP_007419430.1 MLAR|XP_007419513.1

>Orthogroup312: MLAR|XP_007403825.1 MLAR|XP_007404200.1 MLAR|XP_007404201.1 MLAR|XP_007404311.1 MLAR|XP_007404668.1 MLAR|XP_007404867.1 MLAR|XP_007404919.1 MLAR|XP_007405212.1 MLAR|XP_007406777.1 MLAR|XP_007407056.1 MLAR|XP_007407909.1 MLAR|XP_007408100.1 MLAR|XP_007408323.1 MLAR|XP_007409593.1 MLAR|XP_007409833.1 MLAR|XP_007410401.1 MLAR|XP_007411380.1 MLAR|XP_007411683.1 MLAR|XP_007411948.1 MLAR|XP_007412165.1 MLAR|XP_007413221.1 MLAR|XP_007413570.1 MLAR|XP_007415263.1 MLAR|XP_007416788.1 MLAR|XP_007416906.1 MLAR|XP_007417038.1 MLAR|XP_007417354.1 MLAR|XP_007418828.1 MLAR|XP_007419265.1 MLAR|XP_007419299.1 MLAR|XP_007419318.1 MLAR|XP_007419474.1 MLAR|XP_007419478.1

>Orthogroup313: ANID|CBF69443.1 BCIN|XP_001556153.2 BCIN|XP_024549057.1 BGRA|VCU39898.1 CFRU|XP_031875836.1 CFRU|XP_031876424.1 CFRU|XP_031883692.1 CGLO|KAF3801974.1 CGLO|KAF3805560.1 CGLO|KAF3810845.1 CHIG|XP_018154980.1 CHIG|XP_018155408.1 CVIN|KAF4893019.1 CVIN|KAF4911110.1 CVIN|KAF4919807.1 CVYL|A02296 CVYL|A02409 CVYL|A05678 FGRM|XP_011322255.1 FGRM|XP_011322292.1 MGRA|XP_003851615.1 MGRA|XP_003853648.1 MLAR|XP_007410064.1 MLAR|XP_007410789.1 MLAR|XP_007412335.1 MORY|QBZ57349.1 MORY|QBZ63038.1 MORY|QBZ65136.1 NCRA|XP_955763.2 NCRA|XP_958357.2 SSCL|APA08865.1 SSCL|APA15833.1

>Orthogroup314: ANID|CBF69518.1 ANID|CBF80182.1 BCIN|XP_024549931.1 CFRU|XP_031875918.1 CFRU|XP_031876681.1 CFRU|XP_031880171.1 CFRU|XP_031881103.1 CFRU|XP_031882595.1 CFRU|XP_031888147.1 CFRU|XP_031893026.1 CGLO|KAF3797725.1 CGLO|KAF3800421.1 CGLO|KAF3800838.1 CGLO|KAF3803918.1 CGLO|KAF3807810.1 CHIG|XP_018151028.1 CHIG|XP_018159094.1 CHIG|XP_018163036.1 CVIN|KAF4888830.1 CVIN|KAF4899216.1 CVIN|KAF4917558.1 CVIN|KAF4921507.1 CVIN|KAF4925435.1 CVIN|KAF4930723.1 CVYL|A01813 CVYL|A03396 CVYL|A08710 CVYL|A08884 CVYL|A11425 FGRM|XP_011324398.1 FGRM|XP_011325753.1 SSCL|APA05256.1

>Orthogroup315: ANID|CBF69550.1 ANID|CBF79612.1 BCIN|XP_001553710.1 BCIN|XP_024549952.1 CFRU|XP_031882267.1 CFRU|XP_031889093.1 CFRU|XP_031890986.1 CGLO|KAF3801666.1 CGLO|KAF3804449.1 CGLO|KAF3808895.1 CHIG|XP_018151441.1 CHIG|XP_018160275.1 CVIN|KAF4921208.1 CVIN|KAF4930918.1 CVYL|A01186 CVYL|A04158 CVYL|A12469 FGRM|XP_011324495.1 FGRM|XP_011327322.1 FGRM|XP_011328501.1 MGRA|XP_003850088.1 MORY|QBZ58385.1 MORY|QBZ58544.1 MORY|QBZ58561.1 MORY|QBZ62153.1 MORY|QBZ64718.1 MORY|QBZ66631.1 MORY|QBZ66658.1 NCRA|XP_957560.3 NCRA|XP_961113.3 SSCL|APA05292.1 SSCL|APA13504.1

>Orthogroup316: ANID|CBF73586.1 BCIN|XP_001555688.1 CFRU|XP_031879392.1 CFRU|XP_031881288.1 CFRU|XP_031883524.1 CFRU|XP_031890007.1 CFRU|XP_031891720.1 CGLO|KAF3801289.1 CGLO|KAF3802848.1 CGLO|KAF3808580.1 CGLO|KAF3810836.1 CGLO|KAF3810975.1 CHIG|XP_018154187.1 CHIG|XP_018158446.1 CHIG|XP_018164854.1 CVIN|KAF4905052.1 CVIN|KAF4910475.1 CVIN|KAF4911119.1 CVIN|KAF4921569.1 CVIN|KAF4921862.1 CVYL|A02173 CVYL|A02303 CVYL|A05165 CVYL|A07799 CVYL|A11565 FGRM|XP_011317568.1 FGRM|XP_011326680.1 MGRA|XP_003848492.1 MORY|QBZ53655.1 MORY|QBZ62522.1 NCRA|XP_964952.3 SSCL|APA10102.1

>Orthogroup317: ANID|CBF74969.1 ANID|CBF80663.1 ANID|CBF82352.1 ANID|CBF83801.1 BCIN|XP_001555405.1 BCIN|XP_001555729.1 BCIN|XP_024546733.1 BCIN|XP_024547314.1 BCIN|XP_024549391.1 BCIN|XP_024553180.1 CFRU|XP_031880732.1 CFRU|XP_031884225.1 CFRU|XP_031884565.1 CGLO|KAF3806819.1 CGLO|KAF3810350.1 CGLO|KAF3811052.1 CGLO|KAF3811154.1 CHIG|XP_018153137.1 CHIG|XP_018160341.1 CVIN|KAF4918162.1 CVIN|KAF4931198.1 CVYL|A04314 CVYL|A05758 CVYL|A09746 MGRA|XP_003855826.1 MORY|QBZ57488.1 SSCL|APA08148.1 SSCL|APA09041.1 SSCL|APA10311.1 SSCL|APA10416.1 SSCL|APA10736.1 SSCL|APA15573.1

>Orthogroup318: ANID|CBF75148.1 ANID|CBF76217.1 ANID|CBF81202.1 BCIN|XP_024546356.1 BCIN|XP_024552605.1 CFRU|XP_031884791.1 CFRU|XP_031888133.1 CFRU|XP_031888415.1 CFRU|XP_031889680.1 CGLO|KAF3800954.1 CGLO|KAF3803670.1 CGLO|KAF3810609.1 CHIG|XP_018153219.1 CHIG|XP_018158963.1 CHIG|XP_018163788.1 CVIN|KAF4908287.1 CVIN|KAF4912792.1 CVIN|KAF4928560.1 CVYL|A05950 CVYL|A13692 CVYL|A14601 FGRM|XP_011319647.1 FGRM|XP_011321726.1 FGRM|XP_011325166.1 FGRM|XP_011327067.1 MGRA|XP_003852261.1 MORY|QBZ58431.1 MORY|QBZ58608.1 NCRA|XP_011393181.1 NCRA|XP_011394289.1 SSCL|APA06868.1 SSCL|APA15690.1

>Orthogroup319: ANID|CBF75555.1 BCIN|XP_001547677.1 BGRA|VCU39170.1 CFRU|XP_031884241.1 CFRU|XP_031884258.1 CFRU|XP_031889182.1 CFRU|XP_031891144.1 CGLO|KAF3797659.1 CGLO|KAF3799992.1 CGLO|KAF3803777.1 CHIG|XP_018153033.1 CHIG|XP_018155432.1 CHIG|XP_018161692.1 CHIG|XP_018161693.1 CVIN|KAF4917915.1 CVIN|KAF4919083.1 CVIN|KAF4919379.1 CVIN|KAF4926921.1 CVYL|A01111 CVYL|A05257 CVYL|A10207 CVYL|A14155 FGRM|XP_011318126.1 FGRM|XP_011319889.1 FGRM|XP_011327428.1 MGRA|XP_003854503.1 MLAR|XP_007407153.1 MLAR|XP_007409428.1 MORY|QBZ57711.1 NCRA|XP_959311.2 NCRA|XP_960310.2 SSCL|APA13314.1

>Orthogroup320: ANID|CBF75665.1 ANID|CBF86626.1 BCIN|XP_001552398.1 BCIN|XP_024547393.1 BCIN|XP_024550992.1 BGRA|VDB89715.1 CFRU|XP_031876492.1 CFRU|XP_031879271.1 CFRU|XP_031885994.1 CGLO|KAF3796995.1 CGLO|KAF3796996.1 CGLO|KAF3808610.1 CHIG|XP_018153691.1 CHIG|XP_018161906.1 CVIN|KAF4894840.1 CVIN|KAF4926160.1 CVIN|KAF4930364.1 CVYL|A05492 CVYL|A12366 FGRM|XP_011320514.1 FGRM|XP_011321553.1 FGRM|XP_011328587.1 MGRA|XP_003849550.1 MGRA|XP_003856565.1 MGRA|XP_003856854.1 MLAR|XP_007405655.1 MLAR|XP_007406368.1 MORY|QBZ63966.1 NCRA|XP_965356.2 SSCL|APA11615.1 SSCL|APA14400.1 SSCL|APA14907.1

>Orthogroup321: ANID|CBF77647.1 BCIN|XP_001546517.1 BCIN|XP_001546962.1 BCIN|XP_001559784.1 CFRU|XP_031884065.1 CFRU|XP_031892384.1 CGLO|KAF3804992.1 CGLO|KAF3809138.1 CHIG|XP_018155957.1 CHIG|XP_018157729.1 CHIG|XP_018163527.1 CVIN|KAF4922106.1 CVIN|KAF4927125.1 CVYL|A01322 CVYL|A06558 FGRM|XP_011319184.1 FGRM|XP_011323364.1 MLAR|XP_007403893.1 MLAR|XP_007405448.1 MLAR|XP_007406924.1 MLAR|XP_007408224.1 MLAR|XP_007414134.1 MLAR|XP_007414667.1 MLAR|XP_007415286.1 MORY|QBZ56122.1 MORY|QBZ61177.1 MORY|QBZ64337.1 NCRA|XP_958846.1 NCRA|XP_959591.1 SSCL|APA09684.1 SSCL|APA12557.1 SSCL|APA15122.1

>Orthogroup322: ANID|CBF80524.1 BCIN|XP_024546782.1 BCIN|XP_024553043.1 CFRU|XP_031875750.1 CFRU|XP_031883742.1 CFRU|XP_031889868.1 CFRU|XP_031892447.1 CGLO|KAF3797208.1 CGLO|KAF3797758.1 CGLO|KAF3800042.1 CGLO|KAF3810523.1 CGLO|KAF3811950.1 CHIG|XP_018155136.1 CHIG|XP_018155646.1 CHIG|XP_018156426.1 CHIG|XP_018159086.1 CVIN|KAF4905815.1 CVIN|KAF4914555.1 CVIN|KAF4918814.1 CVIN|KAF4920814.1 CVYL|A02973 CVYL|A06114 CVYL|A10489 CVYL|A13602 FGRM|XP_011318790.1 FGRM|XP_011319694.1 FGRM|XP_011319886.1 FGRM|XP_011322505.1 MGRA|XP_003857695.1 MORY|QBZ57732.1 NCRA|XP_958980.2 SSCL|APA16163.1

>Orthogroup323: ANID|CBF83602.1 BCIN|XP_024549072.1 CFRU|XP_031876145.1 CFRU|XP_031876249.1 CFRU|XP_031877356.1 CFRU|XP_031880248.1 CFRU|XP_031882995.1 CFRU|XP_031890226.1 CGLO|KAF3798727.1 CGLO|KAF3799879.1 CGLO|KAF3802329.1 CGLO|KAF3802330.1 CGLO|KAF3804401.1 CGLO|KAF3807947.1 CGLO|KAF3808982.1 CGLO|KAF3808983.1 CGLO|KAF3810292.1 CHIG|XP_018158960.1 CHIG|XP_018159768.1 CVIN|KAF4899860.1 CVIN|KAF4915929.1 CVIN|KAF4918611.1 CVIN|KAF4920849.1 CVIN|KAF4924056.1 CVIN|KAF4930951.1 CVYL|A01302 CVYL|A08677 CVYL|A09186 CVYL|A11260 CVYL|A12712 FGRM|XP_011319537.1 FGRM|XP_011320976.1

>Orthogroup324: BCIN|XP_001555845.2 BCIN|XP_001557604.1 CFRU|XP_031881415.1 CFRU|XP_031885685.1 CFRU|XP_031885687.1 CFRU|XP_031885797.1 CFRU|XP_031887634.1 CGLO|KAF3803227.1 CGLO|KAF3805723.1 CGLO|KAF3810248.1 CGLO|KAF3810250.1 CGLO|KAF3810272.1 CHIG|XP_018152793.1 CHIG|XP_018157940.1 CHIG|XP_018163629.1 CVIN|KAF4910791.1 CVIN|KAF4910793.1 CVIN|KAF4918598.1 CVIN|KAF4926587.1 CVIN|KAF4928674.1 CVYL|A02850 CVYL|A02852 CVYL|A03658 CVYL|A09820 CVYL|A11274 FGRM|XP_011321989.1 MORY|QBZ56818.1 MORY|QBZ60565.1 NCRA|XP_957112.1 NCRA|XP_965402.2 SSCL|APA07625.1 SSCL|APA14563.1

>Orthogroup325: BCIN|XP_024548418.1 CFRU|XP_031878704.1 CFRU|XP_031880463.1 CFRU|XP_031881632.1 CFRU|XP_031893173.1 CGLO|KAF3797576.1 CGLO|KAF3805372.1 CGLO|KAF3807256.1 CGLO|KAF3808127.1 CHIG|XP_018152824.1 CHIG|XP_018155187.1 CHIG|XP_018156736.1 CHIG|XP_018156928.1 CVIN|KAF4907225.1 CVIN|KAF4909730.1 CVIN|KAF4923585.1 CVIN|KAF4928190.1 CVYL|A08108 CVYL|A08968 CVYL|A10736 FGRM|XP_011318003.1 FGRM|XP_011320223.1 FGRM|XP_011326208.1 MGRA|XP_003847933.1 MGRA|XP_003854715.1 MLAR|XP_007410905.1 MLAR|XP_007413427.1 MORY|QBZ55022.1 MORY|QBZ65847.1 NCRA|XP_959493.1 NCRA|XP_961661.2 SSCL|APA12281.1

>Orthogroup326: BGRA|VDB87786.1 CFRU|XP_031876257.1 CFRU|XP_031876259.1 CFRU|XP_031876264.1 CFRU|XP_031876265.1 CFRU|XP_031876708.1 CFRU|XP_031877267.1 CFRU|XP_031877268.1 CFRU|XP_031877883.1 CFRU|XP_031878772.1 CFRU|XP_031879745.1 CFRU|XP_031880324.1 CFRU|XP_031881081.1 CFRU|XP_031881842.1 CFRU|XP_031882813.1 CFRU|XP_031883986.1 CFRU|XP_031886343.1 CFRU|XP_031886345.1 CFRU|XP_031886350.1 CFRU|XP_031886440.1 CFRU|XP_031887457.1 CFRU|XP_031887569.1 CFRU|XP_031887659.1 CFRU|XP_031887878.1 CFRU|XP_031888953.1 CFRU|XP_031889294.1 CFRU|XP_031890273.1 CFRU|XP_031891454.1 CFRU|XP_031892359.1 CFRU|XP_031892503.1 CFRU|XP_031892524.1 CVIN|KAF4892474.1

>Orthogroup327: MLAR|XP_007403479.1 MLAR|XP_007403625.1 MLAR|XP_007405101.1 MLAR|XP_007405440.1 MLAR|XP_007408020.1 MLAR|XP_007408420.1 MLAR|XP_007409079.1 MLAR|XP_007409080.1 MLAR|XP_007409408.1 MLAR|XP_007410113.1 MLAR|XP_007410854.1 MLAR|XP_007410918.1 MLAR|XP_007411740.1 MLAR|XP_007411741.1 MLAR|XP_007412241.1 MLAR|XP_007412242.1 MLAR|XP_007412502.1 MLAR|XP_007412625.1 MLAR|XP_007412627.1 MLAR|XP_007412829.1 MLAR|XP_007413302.1 MLAR|XP_007414764.1 MLAR|XP_007414803.1 MLAR|XP_007415337.1 MLAR|XP_007415868.1 MLAR|XP_007416641.1 MLAR|XP_007417154.1 MLAR|XP_007417984.1 MLAR|XP_007418026.1 MLAR|XP_007418637.1 MLAR|XP_007418638.1 MLAR|XP_007419193.1

>Orthogroup328: MLAR|XP_007403918.1 MLAR|XP_007404256.1 MLAR|XP_007404257.1 MLAR|XP_007405759.1 MLAR|XP_007406358.1 MLAR|XP_007408818.1 MLAR|XP_007408819.1 MLAR|XP_007408821.1 MLAR|XP_007409232.1 MLAR|XP_007409746.1 MLAR|XP_007410822.1 MLAR|XP_007411374.1 MLAR|XP_007411375.1 MLAR|XP_007411376.1 MLAR|XP_007412331.1 MLAR|XP_007412663.1 MLAR|XP_007412858.1 MLAR|XP_007412866.1 MLAR|XP_007412945.1 MLAR|XP_007413557.1 MLAR|XP_007413558.1 MLAR|XP_007413559.1 MLAR|XP_007413610.1 MLAR|XP_007413724.1 MLAR|XP_007413790.1 MLAR|XP_007416097.1 MLAR|XP_007416098.1 MLAR|XP_007416099.1 MLAR|XP_007417986.1 MLAR|XP_007418549.1 MLAR|XP_007419075.1 MLAR|XP_007419076.1

>Orthogroup329: MLAR|XP_007404208.1 MLAR|XP_007404329.1 MLAR|XP_007405756.1 MLAR|XP_007407653.1 MLAR|XP_007408030.1 MLAR|XP_007408699.1 MLAR|XP_007408883.1 MLAR|XP_007408884.1 MLAR|XP_007408885.1 MLAR|XP_007410155.1 MLAR|XP_007410552.1 MLAR|XP_007410714.1 MLAR|XP_007411126.1 MLAR|XP_007411491.1 MLAR|XP_007411522.1 MLAR|XP_007412067.1 MLAR|XP_007413166.1 MLAR|XP_007413495.1 MLAR|XP_007414137.1 MLAR|XP_007414653.1 MLAR|XP_007416202.1 MLAR|XP_007416312.1 MLAR|XP_007417747.1 MLAR|XP_007417889.1 MLAR|XP_007417964.1 MLAR|XP_007418248.1 MLAR|XP_007418249.1 MLAR|XP_007418315.1 MLAR|XP_007418969.1 MLAR|XP_007419069.1 MLAR|XP_007419268.1 MLAR|XP_007419722.1

>Orthogroup330: ANID|CBF69494.1 ANID|CBF79796.1 ANID|CBF82875.1 ANID|CBF83007.1 ANID|CBF84170.1 ANID|CBF87312.1 BCIN|XP_001557897.2 BCIN|XP_024547526.1 CFRU|XP_031881495.1 CFRU|XP_031882321.1 CFRU|XP_031889754.1 CGLO|KAF3804386.1 CGLO|KAF3805787.1 CGLO|KAF3810468.1 CHIG|XP_018159187.1 CHIG|XP_018160210.1 CHIG|XP_018163685.1 CVIN|KAF4894252.1 CVIN|KAF4924663.1 CVIN|KAF4926571.1 CVYL|A04081 CVYL|A06033 CVYL|A14069 FGRM|XP_011320888.1 FGRM|XP_011325293.1 MLAR|XP_007403579.1 MORY|QBZ58420.1 MORY|QBZ65147.1 NCRA|XP_963284.2 SSCL|APA07701.1 SSCL|APA12777.1

>Orthogroup331: ANID|CBF70434.1 BCIN|XP_001554522.1 BCIN|XP_024551026.1 CFRU|XP_031875551.1 CFRU|XP_031876440.1 CFRU|XP_031878699.1 CFRU|XP_031881416.1 CGLO|KAF3798345.1 CGLO|KAF3801937.1 CGLO|KAF3805722.1 CGLO|KAF3808551.1 CGLO|KAF3811782.1 CHIG|XP_018151066.1 CHIG|XP_018151129.1 CHIG|XP_018154106.1 CHIG|XP_018163630.1 CVIN|KAF4918719.1 CVIN|KAF4923561.1 CVIN|KAF4926588.1 CVYL|A02374 CVYL|A02375 CVYL|A09019 CVYL|A09821 CVYL|A12787 FGRM|XP_011323067.1 FGRM|XP_011323424.1 MGRA|XP_003851635.1 MGRA|XP_003854271.1 MORY|QBZ58223.1 MORY|QBZ60328.1 SSCL|APA11856.1

>Orthogroup332: ANID|CBF71378.1 ANID|CBF78815.1 ANID|CBF82802.1 BCIN|XP_024546349.1 BCIN|XP_024552631.1 CFRU|XP_031884444.1 CFRU|XP_031888588.1 CFRU|XP_031891025.1 CFRU|XP_031892493.1 CGLO|KAF3797192.1 CGLO|KAF3803625.1 CGLO|KAF3810346.1 CHIG|XP_018155588.1 CHIG|XP_018157315.1 CHIG|XP_018157316.1 CHIG|XP_018163838.1 CVIN|KAF4920370.1 CVIN|KAF4920802.1 CVYL|A14557 FGRM|XP_011319929.1 FGRM|XP_011323295.1 FGRM|XP_011323296.1 FGRM|XP_011323449.1 FGRM|XP_011326543.1 MGRA|XP_003850353.1 MGRA|XP_003851324.1 MGRA|XP_003851326.1 MORY|QBZ54729.1 MORY|QBZ64086.1 NCRA|XP_963943.3 NCRA|XP_964243.3

>Orthogroup333: ANID|CBF71566.1 BCIN|XP_001550218.2 BCIN|XP_001553384.1 BCIN|XP_001557852.1 BCIN|XP_024546401.1 BCIN|XP_024546737.1 BCIN|XP_024550986.1 BCIN|XP_024551334.1 BCIN|XP_024552993.1 BGRA|VCU39924.1 BGRA|VDB92710.1 CFRU|XP_031876460.1 CFRU|XP_031876960.1 CGLO|KAF3798003.1 CGLO|KAF3810718.1 CHIG|XP_018162576.1 CVIN|KAF4908233.1 CVYL|A07235 FGRM|XP_011318154.1 FGRM|XP_011323979.1 FGRM|XP_011327439.1 MORY|QBZ54862.1 MORY|QBZ64610.1 NCRA|XP_961837.1 SSCL|APA08306.1 SSCL|APA11601.1 SSCL|APA12828.1 SSCL|APA13997.1 SSCL|APA14632.1 SSCL|APA15132.1 SSCL|APA16328.1

>Orthogroup334: ANID|CBF73426.1 ANID|CBF89386.1 BCIN|XP_001560992.1 CFRU|XP_031876685.1 CFRU|XP_031876689.1 CFRU|XP_031879925.1 CFRU|XP_031879926.1 CFRU|XP_031880033.1 CFRU|XP_031884856.1 CGLO|KAF3799022.1 CGLO|KAF3801005.1 CGLO|KAF3811787.1 CHIG|XP_018151712.1 CHIG|XP_018153115.1 CHIG|XP_018154072.1 CHIG|XP_018154073.1 CVIN|KAF4895110.1 CVIN|KAF4895112.1 CVIN|KAF4907648.1 CVIN|KAF4912995.1 CVIN|KAF4918704.1 CVIN|KAF4918707.1 CVYL|A02362 CVYL|A05901 CVYL|A07937 CVYL|A12792 FGRM|XP_011318643.1 MLAR|XP_007408973.1 MORY|QBZ66585.1 NCRA|XP_964440.1 SSCL|APA06693.1

>Orthogroup335: ANID|CBF73446.1 ANID|CBF84473.1 BCIN|XP_001555869.1 BCIN|XP_024546703.1 BCIN|XP_024546704.1 BCIN|XP_024550075.1 BCIN|XP_024551210.1 BCIN|XP_024551827.1 BCIN|XP_024553475.1 CFRU|XP_031878440.1 CFRU|XP_031884053.1 CFRU|XP_031884054.1 CGLO|KAF3798629.1 CGLO|KAF3805095.1 CHIG|XP_018153912.1 CHIG|XP_018154164.1 CHIG|XP_018156525.1 CHIG|XP_018161567.1 CVIN|KAF4912454.1 CVYL|A04819 FGRM|XP_011315623.1 FGRM|XP_011324691.1 MGRA|XP_003847610.1 MGRA|XP_003848839.1 MORY|QBZ60789.1 MORY|QBZ66636.1 NCRA|XP_959242.1 SSCL|APA06069.1 SSCL|APA08633.1 SSCL|APA10891.1 SSCL|APA11942.1

>Orthogroup336: ANID|CBF73475.1 ANID|CBF73513.1 ANID|CBF82913.1 ANID|CBF88302.1 BCIN|XP_001550230.1 BCIN|XP_024545918.1 BCIN|XP_024545919.1 BCIN|XP_024549266.1 BCIN|XP_024553032.1 CFRU|XP_031880790.1 CFRU|XP_031883478.1 CFRU|XP_031893437.1 CGLO|KAF3806926.1 CGLO|KAF3809567.1 CGLO|KAF3810802.1 CHIG|XP_018159609.1 CVIN|KAF4912196.1 CVIN|KAF4919159.1 CVIN|KAF4921789.1 CVYL|A02569 CVYL|A08459 CVYL|A08639 FGRM|XP_011321646.1 FGRM|XP_011326243.1 FGRM|XP_011327572.1 MGRA|XP_003848108.1 MGRA|XP_003848669.1 MGRA|XP_003856082.1 MORY|QBZ66486.1 SSCL|APA08882.1 SSCL|APA16344.1

>Orthogroup337: ANID|CBF73530.1 ANID|CBF76016.1 ANID|CBF80735.1 ANID|CBF82528.1 ANID|CBF87170.1 ANID|CBF89636.1 BCIN|XP_024548785.1 BCIN|XP_024550673.1 BCIN|XP_024550803.1 BCIN|XP_024552469.1 BGRA|VDB83921.1 CFRU|XP_031884257.1 CFRU|XP_031889430.1 CGLO|KAF3797685.1 CGLO|KAF3799993.1 CGLO|KAF3801372.1 CGLO|KAF3802089.1 CGLO|KAF3802199.1 CHIG|XP_018153034.1 CHIG|XP_018155461.1 CVIN|KAF4919371.1 CVIN|KAF4926920.1 CVYL|A01086 CVYL|A10208 FGRM|XP_011315864.1 FGRM|XP_011323090.1 FGRM|XP_011323338.1 MGRA|XP_003850462.1 NCRA|XP_001728451.2 SSCL|APA08541.1 SSCL|APA09678.1

>Orthogroup338: ANID|CBF73583.1 BCIN|XP_001552608.1 BGRA|VDB89162.1 BGRA|VDB89163.1 CFRU|XP_031887475.1 CFRU|XP_031888933.1 CFRU|XP_031891654.1 CGLO|KAF3804277.1 CGLO|KAF3804601.1 CGLO|KAF3809782.1 CHIG|XP_018151031.1 CHIG|XP_018160084.1 CHIG|XP_018160991.1 CHIG|XP_018161668.1 CVIN|KAF4891282.1 CVIN|KAF4909068.1 CVIN|KAF4917574.1 CVYL|A03559 CVYL|A05279 FGRM|XP_011323804.1 FGRM|XP_011328379.1 MGRA|XP_003851293.1 MGRA|XP_003857467.1 MLAR|XP_007403607.1 MORY|QBZ58737.1 MORY|QBZ63646.1 MORY|QBZ64310.1 MORY|QBZ66498.1 NCRA|XP_959085.1 NCRA|XP_962219.1 SSCL|APA11454.1

>Orthogroup339: ANID|CBF74601.1 ANID|CBF76052.1 ANID|CBF80119.1 ANID|CBF81938.1 BCIN|XP_001548314.1 BCIN|XP_001556763.1 CFRU|XP_031876368.1 CFRU|XP_031878156.1 CFRU|XP_031891143.1 CFRU|XP_031892232.1 CGLO|KAF3801859.1 CGLO|KAF3804626.1 CHIG|XP_018155191.1 CHIG|XP_018159373.1 CVIN|KAF4901937.1 CVIN|KAF4919107.1 CVIN|KAF4920585.1 CVIN|KAF4921976.1 CVYL|A02305 CVYL|A05258 CVYL|A06203 CVYL|A06623 FGRM|XP_011328676.1 MGRA|XP_003848290.1 MGRA|XP_003849564.1 MGRA|XP_003851889.1 MGRA|XP_003851906.1 MGRA|XP_003856678.1 MGRA|XP_003857327.1 MORY|QBZ53455.1 SSCL|APA06376.1

>Orthogroup340: ANID|CBF74849.1 ANID|CBF76810.1 ANID|CBF80370.1 ANID|CBF82140.1 ANID|CBF84436.1 ANID|CBF88655.1 BCIN|XP_001559983.2 BCIN|XP_024551017.1 CFRU|XP_031880802.1 CFRU|XP_031884398.1 CGLO|KAF3796973.1 CGLO|KAF3798479.1 CGLO|KAF3806980.1 CHIG|XP_018150736.1 CHIG|XP_018158317.1 CHIG|XP_018159623.1 CVIN|KAF4913292.1 CVIN|KAF4918766.1 CVYL|A05808 CVYL|A06273 CVYL|A08612 FGRM|XP_011316513.1 FGRM|XP_011318647.1 FGRM|XP_011319497.1 FGRM|XP_011322157.1 FGRM|XP_011323870.1 MORY|QBZ56730.1 NCRA|XP_965578.1 SSCL|APA08451.1 SSCL|APA09795.1 SSCL|APA10649.1

>Orthogroup341: ANID|CBF75326.1 ANID|CBF77459.1 ANID|CBF78346.1 BCIN|XP_001559491.1 BCIN|XP_024548912.1 BCIN|XP_024549591.1 BGRA|VCU39573.1 BGRA|VDB83882.1 CFRU|XP_031884405.1 CFRU|XP_031886973.1 CGLO|KAF3810340.1 CGLO|KAF3811374.1 CHIG|XP_018158356.1 CHIG|XP_018160610.1 CVIN|KAF4895361.1 CVIN|KAF4921034.1 CVYL|A04566 CVYL|A05766 FGRM|XP_011316488.1 FGRM|XP_011327211.1 MGRA|XP_003851185.1 MGRA|XP_003855881.1 MLAR|XP_007410442.1 MLAR|XP_007413047.1 MLAR|XP_007413157.1 MORY|QBZ55185.1 MORY|QBZ56905.1 NCRA|XP_011392822.1 NCRA|XP_011394481.1 SSCL|APA09570.1 SSCL|APA13980.1

>Orthogroup342: ANID|CBF75776.1 ANID|CBF87481.1 BCIN|XP_001558008.1 BCIN|XP_024547454.1 CFRU|XP_031879774.1 CFRU|XP_031885606.1 CFRU|XP_031892226.1 CGLO|KAF3798561.1 CGLO|KAF3809702.1 CGLO|KAF3810045.1 CHIG|XP_018153852.1 CHIG|XP_018157762.1 CHIG|XP_018165079.1 CVIN|KAF4917932.1 CVIN|KAF4921973.1 CVIN|KAF4930099.1 CVYL|A02641 CVYL|A04879 CVYL|A06625 FGRM|XP_011317842.1 FGRM|XP_011322077.1 FGRM|XP_011325371.1 MGRA|XP_003851797.1 MORY|QBZ57710.1 MORY|QBZ60001.1 MORY|QBZ61179.1 NCRA|XP_957450.3 NCRA|XP_964145.1 SSCL|APA12050.1 SSCL|APA12709.1 SSCL|APA15996.1

>Orthogroup343: ANID|CBF76125.1 ANID|CBF76133.1 ANID|CBF79648.1 ANID|CBF89739.1 BCIN|XP_001555925.1 BCIN|XP_024546928.1 BGRA|VDB93665.1 BGRA|VDB93851.1 CFRU|XP_031882639.1 CFRU|XP_031884833.1 CGLO|KAF3800492.1 CGLO|KAF3812220.1 CHIG|XP_018152269.1 CHIG|XP_018153442.1 CVIN|KAF4916331.1 CVIN|KAF4920984.1 CVYL|A00166 CVYL|A13221 FGRM|XP_011316059.1 FGRM|XP_011316331.1 MGRA|XP_003847074.1 MGRA|XP_003847311.1 MGRA|XP_003848766.1 MLAR|XP_007405540.1 MLAR|XP_007415976.1 MORY|QBZ53949.1 MORY|QBZ55286.1 NCRA|XP_958904.2 NCRA|XP_963223.1 SSCL|APA05812.1 SSCL|APA06029.1

>Orthogroup344: ANID|CBF76155.1 ANID|CBF84217.1 BCIN|XP_024546732.1 CFRU|XP_031876113.1 CFRU|XP_031880078.1 CFRU|XP_031881004.1 CFRU|XP_031882031.1 CGLO|KAF3800412.1 CGLO|KAF3806099.1 CGLO|KAF3807366.1 CGLO|KAF3811801.1 CHIG|XP_018154176.1 CHIG|XP_018156847.1 CHIG|XP_018161320.1 CHIG|XP_018161321.1 CHIG|XP_018163409.1 CHIG|XP_018164570.1 CVIN|KAF4902342.1 CVIN|KAF4920083.1 CVIN|KAF4924875.1 CVYL|A10861 CVYL|A12805 FGRM|XP_011318014.1 FGRM|XP_011321735.1 FGRM|XP_011327666.1 FGRM|XP_011328843.1 MORY|QBZ57525.1 MORY|QBZ65175.1 MORY|QBZ65410.1 MORY|QBZ66412.1 SSCL|APA06824.1

>Orthogroup345: ANID|CBF76271.1 ANID|CBF85349.1 ANID|CBF85646.1 ANID|CBF86322.1 BCIN|XP_001560558.1 BCIN|XP_001561213.1 CFRU|XP_031880121.1 CFRU|XP_031883894.1 CFRU|XP_031889752.1 CFRU|XP_031891056.1 CFRU|XP_031893603.1 CGLO|KAF3800867.1 CGLO|KAF3810466.1 CGLO|KAF3811681.1 CHIG|XP_018150832.1 CHIG|XP_018151852.1 CHIG|XP_018159189.1 CVIN|KAF4915583.1 CVIN|KAF4915938.1 CVIN|KAF4924616.1 CVYL|A11106 CVYL|A12704 CVYL|A14067 FGRM|XP_011321582.1 MGRA|XP_003852468.1 MGRA|XP_003855829.1 MLAR|XP_007405489.1 MORY|QBZ54098.1 MORY|QBZ60525.1 NCRA|XP_963989.1 SSCL|APA07174.1

>Orthogroup346: ANID|CBF77083.1 ANID|CBF78232.1 BCIN|XP_024547457.1 CFRU|XP_031886067.1 CFRU|XP_031886425.1 CFRU|XP_031891469.1 CFRU|XP_031893227.1 CGLO|KAF3804664.1 CGLO|KAF3805473.1 CGLO|KAF3809638.1 CGLO|KAF3811875.1 CHIG|XP_018152584.1 CHIG|XP_018155181.1 CHIG|XP_018155342.1 CHIG|XP_018161737.1 CVIN|KAF4896991.1 CVIN|KAF4897795.1 CVIN|KAF4913891.1 CVIN|KAF4920576.1 CVYL|A05221 CVYL|A05603 CVYL|A06186 CVYL|A08410 FGRM|XP_011315819.1 FGRM|XP_011318507.1 FGRM|XP_011322430.1 FGRM|XP_011322560.1 MORY|QBZ53730.1 NCRA|XP_011394131.1 NCRA|XP_962188.1 SSCL|APA15999.1

>Orthogroup347: ANID|CBF78511.1 ANID|CBF78933.1 ANID|CBF82401.1 ANID|CBF85655.1 BCIN|XP_001559371.1 BCIN|XP_024550709.1 CFRU|XP_031882215.1 CFRU|XP_031889310.1 CGLO|KAF3804372.1 CGLO|KAF3808908.1 CHIG|XP_018152057.1 CHIG|XP_018160166.1 CVIN|KAF4918347.1 CVYL|A01137 CVYL|A01178 CVYL|A04066 FGRM|XP_011322014.1 FGRM|XP_011324548.1 FGRM|XP_011324986.1 FGRM|XP_011325907.1 MGRA|XP_003848264.1 MGRA|XP_003852136.1 MGRA|XP_003856825.1 MORY|QBZ57851.1 MORY|QBZ60884.1 MORY|QBZ63981.1 MORY|QBZ65201.1 NCRA|XP_958313.1 NCRA|XP_964784.1 SSCL|APA07702.1 SSCL|APA12003.1

>Orthogroup348: ANID|CBF78548.1 ANID|CBF80076.1 ANID|CBF81681.1 BCIN|XP_001559006.1 CFRU|XP_031879037.1 CFRU|XP_031879342.1 CFRU|XP_031881640.1 CFRU|XP_031884465.1 CFRU|XP_031893427.1 CGLO|KAF3798859.1 CGLO|KAF3807412.1 CGLO|KAF3810326.1 CHIG|XP_018151172.1 CHIG|XP_018153043.1 CHIG|XP_018157328.1 CVIN|KAF4908443.1 CVIN|KAF4920332.1 CVIN|KAF4929590.1 CVYL|A09077 CVYL|A10898 FGRM|XP_011320824.1 FGRM|XP_011320863.1 FGRM|XP_011322491.1 MGRA|XP_003847974.1 MGRA|XP_003851105.1 MGRA|XP_003853925.1 MGRA|XP_003856158.1 MGRA|XP_003856661.1 MGRA|XP_003856836.1 MGRA|XP_003857746.1 SSCL|APA05859.1

>Orthogroup349: ANID|CBF78682.1 BCIN|XP_001557163.1 BGRA|VDB93474.1 CFRU|XP_031885035.1 CFRU|XP_031891920.1 CGLO|KAF3800358.1 CGLO|KAF3801384.1 CGLO|KAF3802947.1 CHIG|XP_018151244.1 CHIG|XP_018151245.1 CHIG|XP_018152330.1 CHIG|XP_018156602.1 CHIG|XP_018162847.1 CVIN|KAF4907807.1 CVIN|KAF4914546.1 CVYL|A05487 CVYL|A07608 CVYL|A07698 FGRM|XP_011326389.1 MGRA|XP_003850942.1 MGRA|XP_003854408.1 MLAR|XP_007404494.1 MLAR|XP_007404550.1 MLAR|XP_007407484.1 MLAR|XP_007416179.1 MORY|QBZ59534.1 MORY|QBZ59999.1 MORY|QBZ66529.1 NCRA|XP_957821.1 NCRA|XP_965606.1 SSCL|APA15005.1

>Orthogroup350: ANID|CBF78871.1 ANID|CBF88287.1 BCIN|XP_001547499.1 BCIN|XP_024546288.1 BCIN|XP_024546702.1 BCIN|XP_024547213.1 BGRA|VDB90862.1 BGRA|VDB93623.1 CFRU|XP_031879880.1 CFRU|XP_031890762.1 CGLO|KAF3798942.1 CGLO|KAF3801525.1 CHIG|XP_018151943.1 CHIG|XP_018152868.1 CVIN|KAF4898136.1 CVIN|KAF4898140.1 CVYL|A07859 CVYL|A12610 FGRM|XP_011324375.1 FGRM|XP_011327722.1 MGRA|XP_003849118.1 MGRA|XP_003854859.1 MLAR|XP_007403496.1 MLAR|XP_007413400.1 MLAR|XP_007415683.1 MORY|QBZ53365.1 MORY|QBZ60187.1 NCRA|XP_957857.1 NCRA|XP_959528.1 SSCL|APA06938.1 SSCL|APA14979.1

>Orthogroup351: ANID|CBF79053.1 ANID|CBF89377.1 BCIN|XP_024553811.1 CFRU|XP_031881069.1 CGLO|KAF3800406.1 CHIG|XP_018158431.1 CHIG|XP_018159451.1 CHIG|XP_018161399.1 CHIG|XP_018161885.1 CHIG|XP_018163064.1 CHIG|XP_018164801.1 CVIN|KAF4906996.1 CVIN|KAF4930603.1 CVYL|A01801 CVYL|A14180 FGRM|XP_011315734.1 FGRM|XP_011322620.1 MGRA|XP_003847946.1 MGRA|XP_003848434.1 MGRA|XP_003849336.1 MGRA|XP_003849918.1 MGRA|XP_003853938.1 MGRA|XP_003856669.1 MGRA|XP_003856808.1 MORY|QBZ54923.1 MORY|QBZ57536.1 MORY|QBZ65843.1 MORY|QBZ66306.1 SSCL|APA06207.1 SSCL|APA09704.1 SSCL|APA14593.1

>Orthogroup352: ANID|CBF80117.1 ANID|CBF85345.1 BCIN|XP_001561291.2 BCIN|XP_024552589.1 BGRA|VCU40873.1 CFRU|XP_031889821.1 CGLO|KAF3798803.1 CGLO|KAF3805225.1 CHIG|XP_018158842.1 CVIN|KAF4911356.1 CVYL|A13873 FGRM|XP_011317948.1 FGRM|XP_011319703.1 FGRM|XP_011321797.1 MGRA|XP_003856447.1 MLAR|XP_007405993.1 MLAR|XP_007406116.1 MLAR|XP_007406177.1 MLAR|XP_007407895.1 MLAR|XP_007407896.1 MLAR|XP_007409257.1 MLAR|XP_007409374.1 MLAR|XP_007409642.1 MLAR|XP_007409643.1 MLAR|XP_007409644.1 MLAR|XP_007411964.1 MLAR|XP_007417463.1 MLAR|XP_007419229.1 MORY|QBZ57419.1 NCRA|XP_964190.2 SSCL|APA06840.1

>Orthogroup353: ANID|CBF81685.1 ANID|CBF87743.1 BCIN|XP_024550884.1 BCIN|XP_024551277.1 CFRU|XP_031880583.1 CFRU|XP_031880839.1 CFRU|XP_031884996.1 CGLO|KAF3801552.1 CGLO|KAF3807860.1 CGLO|KAF3812197.1 CHIG|XP_018151576.1 CHIG|XP_018153422.1 CHIG|XP_018154244.1 CVIN|KAF4920945.1 CVIN|KAF4922624.1 CVYL|A00188 CVYL|A00751 FGRM|XP_011315820.1 MGRA|XP_003848201.1 MORY|QBZ53323.1 MORY|QBZ54754.1 MORY|QBZ58335.1 MORY|QBZ58501.1 MORY|QBZ61263.1 MORY|QBZ63732.1 MORY|QBZ66503.1 MORY|QBZ66504.1 MORY|QBZ66625.1 MORY|QBZ66644.1 NCRA|XP_959383.2 SSCL|APA11812.1

>Orthogroup354: ANID|CBF82238.1 CFRU|XP_031875299.1 CFRU|XP_031875364.1 CFRU|XP_031875650.1 CFRU|XP_031876227.1 CFRU|XP_031876972.1 CFRU|XP_031877070.1 CFRU|XP_031877525.1 CFRU|XP_031877552.1 CFRU|XP_031879325.1 CFRU|XP_031879383.1 CFRU|XP_031880015.1 CFRU|XP_031880427.1 CFRU|XP_031880857.1 CFRU|XP_031881101.1 CFRU|XP_031881132.1 CFRU|XP_031881496.1 CFRU|XP_031881877.1 CFRU|XP_031882061.1 CFRU|XP_031883897.1 CFRU|XP_031883952.1 CFRU|XP_031884437.1 CFRU|XP_031884620.1 CFRU|XP_031884953.1 CFRU|XP_031887570.1 CFRU|XP_031888913.1 CFRU|XP_031890022.1 CFRU|XP_031892147.1 CFRU|XP_031892739.1 CFRU|XP_031893016.1 CFRU|XP_031893197.1

>Orthogroup355: ANID|CBF83591.1 ANID|CBF85846.1 CFRU|XP_031875443.1 CFRU|XP_031876048.1 CFRU|XP_031883299.1 CFRU|XP_031884613.1 CFRU|XP_031889037.1 CFRU|XP_031893374.1 CGLO|KAF3801703.1 CGLO|KAF3804216.1 CGLO|KAF3805252.1 CGLO|KAF3808052.1 CHIG|XP_018151306.1 CHIG|XP_018152750.1 CHIG|XP_018158822.1 CHIG|XP_018159210.1 CVIN|KAF4915642.1 CVIN|KAF4918267.1 CVIN|KAF4918684.1 CVIN|KAF4922820.1 CVYL|A08180 CVYL|A11449 CVYL|A12995 CVYL|A13901 FGRM|XP_011327445.1 MGRA|XP_003851384.1 MGRA|XP_003857220.1 MORY|QBZ59854.1 MORY|QBZ60537.1 MORY|QBZ64398.1 NCRA|XP_011392819.1

>Orthogroup356: BCIN|XP_024553612.1 BGRA|VDB88180.1 CFRU|XP_031885093.1 CFRU|XP_031889535.1 CFRU|XP_031889635.1 CGLO|KAF3805889.1 CGLO|KAF3807721.1 CGLO|KAF3810529.1 CHIG|XP_018158911.1 CHIG|XP_018159097.1 CHIG|XP_018162954.1 CVIN|KAF4914577.1 CVIN|KAF4918888.1 CVIN|KAF4920773.1 CVYL|A07020 CVYL|A13607 CVYL|A13824 FGRM|XP_011318301.1 FGRM|XP_011318635.1 FGRM|XP_011321269.1 FGRM|XP_011322061.1 FGRM|XP_011322486.1 FGRM|XP_011324405.1 FGRM|XP_011325318.1 FGRM|XP_011325321.1 FGRM|XP_011326210.1 FGRM|XP_011327420.1 MLAR|XP_007413003.1 MORY|QBZ55883.1 NCRA|XP_957068.2 SSCL|APA08460.1

>Orthogroup357: CGLO|KAF3801360.1 CHIG|XP_018151025.1 CHIG|XP_018151786.1 CHIG|XP_018157087.1 CHIG|XP_018157696.1 CHIG|XP_018158904.1 CHIG|XP_018159930.1 CHIG|XP_018161279.1 CHIG|XP_018162951.1 CHIG|XP_018164775.1 CVIN|KAF4891595.1 CVIN|KAF4905024.1 CVIN|KAF4913308.1 CVIN|KAF4914943.1 CVIN|KAF4918237.1 CVIN|KAF4918340.1 CVIN|KAF4918363.1 CVIN|KAF4918876.1 CVIN|KAF4919585.1 CVIN|KAF4920144.1 CVIN|KAF4920551.1 CVIN|KAF4920552.1 CVIN|KAF4920594.1 CVIN|KAF4920595.1 CVIN|KAF4920596.1 CVIN|KAF4923235.1 CVIN|KAF4923236.1 CVIN|KAF4924675.1 CVIN|KAF4925692.1 CVIN|KAF4925693.1 CVIN|KAF4930149.1

>Orthogroup358: MLAR|XP_007403861.1 MLAR|XP_007404812.1 MLAR|XP_007405056.1 MLAR|XP_007405863.1 MLAR|XP_007406695.1 MLAR|XP_007407286.1 MLAR|XP_007408867.1 MLAR|XP_007410884.1 MLAR|XP_007411177.1 MLAR|XP_007412451.1 MLAR|XP_007412735.1 MLAR|XP_007412973.1 MLAR|XP_007414315.1 MLAR|XP_007415096.1 MLAR|XP_007415415.1 MLAR|XP_007416704.1 MLAR|XP_007417142.1 MLAR|XP_007417842.1 MLAR|XP_007417950.1 MLAR|XP_007417958.1 MLAR|XP_007418094.1 MLAR|XP_007418554.1 MLAR|XP_007418724.1 MLAR|XP_007418846.1 MLAR|XP_007418979.1 MLAR|XP_007419038.1 MLAR|XP_007419108.1 MLAR|XP_007419314.1 MLAR|XP_007419365.1 MLAR|XP_007419393.1 MLAR|XP_007419510.1

>Orthogroup359: ANID|CBF69585.1 ANID|CBF71775.1 ANID|CBF84269.1 BCIN|XP_001560369.1 CFRU|XP_031881449.1 CFRU|XP_031883573.1 CFRU|XP_031885136.1 CFRU|XP_031891184.1 CFRU|XP_031892128.1 CGLO|KAF3800083.1 CGLO|KAF3800938.1 CGLO|KAF3804743.1 CGLO|KAF3807777.1 CGLO|KAF3811908.1 CHIG|XP_018153202.1 CHIG|XP_018155143.1 CVIN|KAF4896540.1 CVIN|KAF4903560.1 CVIN|KAF4911827.1 CVIN|KAF4920899.1 CVIN|KAF4921500.1 CVYL|A00823 CVYL|A05377 CVYL|A05966 CVYL|A06150 FGRM|XP_011328687.1 MGRA|XP_003851141.1 MORY|QBZ57678.1 MORY|QBZ61740.1 SSCL|APA08797.1

>Orthogroup360: ANID|CBF69653.1 ANID|CBF74791.1 BCIN|XP_001551851.1 BCIN|XP_024553827.1 BGRA|VDB86357.1 CFRU|XP_031876648.1 CFRU|XP_031891489.1 CFRU|XP_031893013.1 CGLO|KAF3797895.1 CGLO|KAF3801907.1 CGLO|KAF3808416.1 CHIG|XP_018152494.1 CHIG|XP_018161627.1 CVIN|KAF4918712.1 CVIN|KAF4923065.1 CVIN|KAF4924995.1 CVYL|A02347 CVYL|A05332 CVYL|A09575 FGRM|XP_011317929.1 FGRM|XP_011318673.1 MGRA|XP_003854592.1 MGRA|XP_003855893.1 MLAR|XP_007417479.1 MORY|QBZ54851.1 MORY|QBZ62031.1 NCRA|XP_958280.1 NCRA|XP_963045.1 SSCL|APA15234.1 SSCL|APA15247.1

>Orthogroup361: ANID|CBF70183.1 ANID|CBF79154.1 ANID|CBF80338.1 ANID|CBF89142.1 BCIN|XP_024551498.1 BCIN|XP_024553290.1 CFRU|XP_031878508.1 CFRU|XP_031883948.1 CFRU|XP_031884903.1 CGLO|KAF3798643.1 CGLO|KAF3805035.1 CGLO|KAF3812097.1 CHIG|XP_018153255.1 CHIG|XP_018153893.1 CHIG|XP_018156518.1 CHIG|XP_018163475.1 CVIN|KAF4912450.1 CVIN|KAF4918118.1 CVYL|A00303 CVYL|A04804 CVYL|A06517 FGRM|XP_011322688.1 MGRA|XP_003848197.1 MGRA|XP_003850204.1 MGRA|XP_003850666.1 MLAR|XP_007404658.1 MLAR|XP_007404664.1 MORY|QBZ58432.1 SSCL|APA10353.1 SSCL|APA13544.1

>Orthogroup362: ANID|CBF70573.1 BCIN|XP_001552045.1 BCIN|XP_001559304.1 BCIN|XP_001560263.2 BCIN|XP_024548803.1 CFRU|XP_031880926.1 CFRU|XP_031886382.1 CGLO|KAF3806367.1 CGLO|KAF3806959.1 CHIG|XP_018151156.1 CHIG|XP_018162226.1 CVIN|KAF4919171.1 CVIN|KAF4923241.1 CVYL|A06249 CVYL|A09060 CVYL|A11953 FGRM|XP_011322924.1 FGRM|XP_011324270.1 MGRA|XP_003852421.1 MGRA|XP_003854255.1 MLAR|XP_007410601.1 MLAR|XP_007411581.1 MLAR|XP_007414526.1 MLAR|XP_007417813.1 MLAR|XP_007418270.1 NCRA|XP_961873.1 SSCL|APA07850.1 SSCL|APA09648.1 SSCL|APA10025.1 SSCL|APA15958.1

>Orthogroup363: ANID|CBF71116.1 ANID|CBF78449.1 BCIN|XP_001550516.1 BCIN|XP_024545961.1 CFRU|XP_031877926.1 CFRU|XP_031878160.1 CFRU|XP_031880884.1 CFRU|XP_031891751.1 CGLO|KAF3800109.1 CGLO|KAF3801803.1 CGLO|KAF3806883.1 CHIG|XP_018154950.1 CHIG|XP_018159585.1 CHIG|XP_018165046.1 CVIN|KAF4894668.1 CVIN|KAF4916984.1 CVIN|KAF4925852.1 CVYL|A02228 CVYL|A02528 CVYL|A06800 CVYL|A12903 FGRM|XP_011318160.1 FGRM|XP_011325889.1 FGRM|XP_011328358.1 FGRM|XP_011328590.1 MGRA|XP_003849919.1 MORY|QBZ54510.1 MORY|QBZ59118.1 NCRA|XP_011395368.1 SSCL|APA15263.1

>Orthogroup364: ANID|CBF71545.1 BCIN|XP_001549964.2 CFRU|XP_031876967.1 CFRU|XP_031878807.1 CFRU|XP_031878992.1 CFRU|XP_031879912.1 CGLO|KAF3799007.1 CGLO|KAF3799139.1 CGLO|KAF3799141.1 CGLO|KAF3802639.1 CGLO|KAF3807850.1 CHIG|XP_018152983.1 CHIG|XP_018162184.1 CHIG|XP_018163040.1 CVIN|KAF4921659.1 CVIN|KAF4922607.1 CVIN|KAF4923444.1 CVYL|A00761 CVYL|A01933 CVYL|A07924 FGRM|XP_011315627.1 FGRM|XP_011320919.1 FGRM|XP_011321851.1 MGRA|XP_003849546.1 MGRA|XP_003853901.1 MGRA|XP_003854368.1 MORY|QBZ55180.1 MORY|QBZ64198.1 NCRA|XP_964786.2 SSCL|APA06623.1

>Orthogroup365: ANID|CBF73413.1 ANID|CBF75131.1 ANID|CBF86422.1 ANID|CBF87201.1 BCIN|XP_024552752.1 CFRU|XP_031876123.1 CFRU|XP_031877067.1 CFRU|XP_031882352.1 CFRU|XP_031882708.1 CGLO|KAF3806635.1 CGLO|KAF3807537.1 CGLO|KAF3811103.1 CHIG|XP_018152964.1 CHIG|XP_018156400.1 CHIG|XP_018163339.1 CHIG|XP_018164227.1 CVIN|KAF4911070.1 CVIN|KAF4918609.1 CVIN|KAF4918912.1 CVIN|KAF4928921.1 CVYL|A02946 CVYL|A07981 CVYL|A11266 CVYL|A12348 FGRM|XP_011315685.1 FGRM|XP_011318580.1 MGRA|XP_003848049.1 MORY|QBZ56956.1 MORY|QBZ64267.1 SSCL|APA07494.1

>Orthogroup366: ANID|CBF73469.1 ANID|CBF74647.1 ANID|CBF75896.1 ANID|CBF81205.1 ANID|CBF87037.1 BCIN|XP_001553602.2 BGRA|VCU40403.1 BGRA|VCU40406.1 BGRA|VCU40412.1 BGRA|VCU40414.1 BGRA|VCU40436.1 BGRA|VCU40972.1 BGRA|VDB90807.1 BGRA|VDB95021.1 BGRA|VDB95339.1 CFRU|XP_031879783.1 CGLO|KAF3802987.1 CHIG|XP_018153626.1 CVIN|KAF4929961.1 CVYL|A00420 FGRM|XP_011316926.1 FGRM|XP_011322341.1 MGRA|XP_003851522.1 MLAR|XP_007410816.1 MORY|QBZ60845.1 MORY|QBZ63847.1 NCRA|XP_955886.2 NCRA|XP_958160.1 NCRA|XP_963774.1 SSCL|APA06434.1

>Orthogroup367: ANID|CBF73850.1 ANID|CBF74667.1 ANID|CBF74948.1 ANID|CBF77786.1 ANID|CBF80089.1 ANID|CBF82092.1 ANID|CBF87867.1 ANID|CBF88422.1 BCIN|XP_001551569.2 BCIN|XP_001554059.1 BCIN|XP_001558734.2 BCIN|XP_024551016.1 BCIN|XP_024552796.1 BGRA|VCU41393.1 CFRU|XP_031879473.1 CHIG|XP_018154653.1 CVIN|KAF4926475.1 CVYL|A13506 FGRM|XP_011321237.1 FGRM|XP_011322752.1 MGRA|XP_003848299.1 MGRA|XP_003848864.1 MGRA|XP_003856513.1 MLAR|XP_007407814.1 MORY|QBZ61019.1 NCRA|XP_959691.3 NCRA|XP_963321.1 SSCL|APA05630.1 SSCL|APA11811.1 SSCL|APA16039.1

>Orthogroup368: ANID|CBF73971.1 ANID|CBF85177.1 ANID|CBF89051.1 BCIN|XP_001559667.2 BCIN|XP_024552819.1 BGRA|VCU41161.1 CFRU|XP_031882460.1 CFRU|XP_031884463.1 CGLO|KAF3807634.1 CGLO|KAF3810331.1 CHIG|XP_018151889.1 CHIG|XP_018156294.1 CHIG|XP_018158305.1 CVIN|KAF4895359.1 CVYL|A03116 CVYL|A05773 FGRM|XP_011319033.1 FGRM|XP_011320990.1 MGRA|XP_003849189.1 MGRA|XP_003856214.1 MGRA|XP_003857246.1 MLAR|XP_007403670.1 MLAR|XP_007403779.1 MLAR|XP_007407350.1 MORY|QBZ62484.1 MORY|QBZ63369.1 MORY|QBZ65027.1 MORY|QBZ65082.1 NCRA|XP_960695.2 SSCL|APA16008.1

>Orthogroup369: ANID|CBF73989.1 BCIN|XP_024546880.1 CFRU|XP_031880022.1 CFRU|XP_031883708.1 CFRU|XP_031884587.1 CGLO|KAF3799762.1 CGLO|KAF3800257.1 CGLO|KAF3805197.1 CGLO|KAF3810863.1 CHIG|XP_018154956.1 CHIG|XP_018158241.1 CHIG|XP_018163615.1 CHIG|XP_018164867.1 CVIN|KAF4897555.1 CVIN|KAF4909083.1 CVIN|KAF4918962.1 CVIN|KAF4920458.1 CVIN|KAF4921773.1 CVYL|A02028 CVYL|A02283 CVYL|A07959 CVYL|A11337 FGRM|XP_011324550.1 MGRA|XP_003846907.1 MGRA|XP_003848021.1 MGRA|XP_003857778.1 MORY|QBZ59132.1 MORY|QBZ64644.1 MORY|QBZ65352.1 NCRA|XP_964849.1

>Orthogroup370: ANID|CBF73997.1 ANID|CBF78599.1 ANID|CBF78703.1 ANID|CBF89948.1 BCIN|XP_001546901.2 BCIN|XP_024547384.1 BCIN|XP_024552528.1 CFRU|XP_031876089.1 CHIG|XP_018151834.1 CHIG|XP_018154196.1 CVIN|KAF4929544.1 CVYL|A10868 FGRM|XP_011319842.1 MGRA|XP_003847218.1 MGRA|XP_003849307.1 MGRA|XP_003849311.1 MGRA|XP_003852326.1 MGRA|XP_003853790.1 MGRA|XP_003854590.1 MGRA|XP_003854914.1 MGRA|XP_003854938.1 MGRA|XP_003856145.1 MGRA|XP_003857554.1 NCRA|XP_961866.1 SSCL|APA06715.1 SSCL|APA11637.1 SSCL|APA11638.1 SSCL|APA14386.1 SSCL|APA15320.1 SSCL|APA16221.1

>Orthogroup371: ANID|CBF75189.1 ANID|CBF83027.1 ANID|CBF87321.1 BCIN|XP_024549436.1 CFRU|XP_031877247.1 CFRU|XP_031883135.1 CFRU|XP_031883461.1 CFRU|XP_031883763.1 CFRU|XP_031884607.1 CGLO|KAF3796952.1 CGLO|KAF3800222.1 CGLO|KAF3800758.1 CGLO|KAF3805040.1 CHIG|XP_018155066.1 CHIG|XP_018163324.1 CHIG|XP_018163465.1 CVIN|KAF4919627.1 CVIN|KAF4919724.1 CVIN|KAF4920420.1 CVYL|A00447 CVYL|A06065 CVYL|A06511 FGRM|XP_011318124.1 FGRM|XP_011321447.1 FGRM|XP_011328413.1 MGRA|XP_003847253.1 MGRA|XP_003856841.1 MGRA|XP_003856876.1 MORY|QBZ57860.1 SSCL|APA09210.1

>Orthogroup372: ANID|CBF77964.1 CFRU|XP_031876338.1 CFRU|XP_031876443.1 CFRU|XP_031878860.1 CFRU|XP_031884416.1 CFRU|XP_031887538.1 CFRU|XP_031890519.1 CGLO|KAF3796979.1 CGLO|KAF3801183.1 CGLO|KAF3801953.1 CGLO|KAF3803286.1 CHIG|XP_018151605.1 CHIG|XP_018155197.1 CHIG|XP_018156909.1 CHIG|XP_018161497.1 CVIN|KAF4890551.1 CVIN|KAF4905513.1 CVIN|KAF4913285.1 CVIN|KAF4919516.1 CVYL|A01914 CVYL|A02388 CVYL|A04974 CVYL|A05802 CVYL|A06209 FGRM|XP_011322895.1 FGRM|XP_011323189.1 FGRM|XP_011325630.1 MORY|QBZ57683.1 MORY|QBZ58286.1 MORY|QBZ63755.1

>Orthogroup373: ANID|CBF78234.1 ANID|CBF84329.1 BCIN|XP_024548876.1 BCIN|XP_024550476.1 CFRU|XP_031876895.1 CFRU|XP_031884183.1 CFRU|XP_031886055.1 CFRU|XP_031891463.1 CGLO|KAF3799839.1 CGLO|KAF3803775.1 CGLO|KAF3804665.1 CGLO|KAF3805474.1 CHIG|XP_018153833.1 CHIG|XP_018155341.1 CHIG|XP_018159140.1 CHIG|XP_018161738.1 CVIN|KAF4898727.1 CVIN|KAF4913892.1 CVIN|KAF4917911.1 CVYL|A05220 CVYL|A05604 CVYL|A09675 CVYL|A14153 FGRM|XP_011327423.1 FGRM|XP_011328741.1 MORY|QBZ53729.1 MORY|QBZ65091.1 NCRA|XP_962558.3 NCRA|XP_963610.2 SSCL|APA11189.1

>Orthogroup374: ANID|CBF78739.1 BCIN|XP_024547388.1 BCIN|XP_024547558.1 BCIN|XP_024552367.1 CFRU|XP_031889043.1 CFRU|XP_031890305.1 CGLO|KAF3799187.1 CGLO|KAF3810396.1 CHIG|XP_018151564.1 CHIG|XP_018151572.1 CHIG|XP_018151837.1 CHIG|XP_018153332.1 CHIG|XP_018156673.1 CHIG|XP_018156676.1 CHIG|XP_018159795.1 CHIG|XP_018164212.1 CVIN|KAF4908461.1 CVIN|KAF4914921.1 CVYL|A08914 CVYL|A10083 MGRA|XP_003852535.1 MGRA|XP_003854833.1 MORY|QBZ60715.1 MORY|QBZ60722.1 MORY|QBZ65099.1 NCRA|XP_956309.2 NCRA|XP_959376.3 SSCL|APA06525.1 SSCL|APA14390.1 SSCL|APA16244.1

>Orthogroup375: ANID|CBF78983.1 BCIN|XP_001550922.1 BCIN|XP_001553496.1 BGRA|VDB84235.1 CFRU|XP_031877066.1 CFRU|XP_031878957.1 CFRU|XP_031891580.1 CGLO|KAF3797636.1 CGLO|KAF3807904.1 CGLO|KAF3811087.1 CHIG|XP_018152978.1 CHIG|XP_018157428.1 CVIN|KAF4893854.1 CVIN|KAF4911068.1 CVIN|KAF4915239.1 CVYL|A00712 CVYL|A09708 CVYL|A11051 FGRM|XP_011323031.1 FGRM|XP_011324322.1 MGRA|XP_003853495.1 MGRA|XP_003853943.1 MGRA|XP_003856683.1 MLAR|XP_007404356.1 MLAR|XP_007404737.1 MORY|QBZ60054.1 MORY|QBZ64358.1 MORY|QBZ66427.1 NCRA|XP_965392.1 SSCL|APA10870.1

>Orthogroup376: ANID|CBF79428.1 ANID|CBF79432.1 BCIN|XP_024549713.1 BCIN|XP_024549714.1 BGRA|VDB91022.1 BGRA|VDB91028.1 CFRU|XP_031882788.1 CFRU|XP_031882864.1 CGLO|KAF3803354.1 CHIG|XP_018164105.1 CVIN|KAF4915964.1 CVIN|KAF4915966.1 CVYL|A14279 CVYL|A14280 FGRM|XP_011316181.1 FGRM|XP_011316182.1 MGRA|XP_003851516.1 MGRA|XP_003851991.1 MLAR|XP_007406386.1 MLAR|XP_007407673.1 MLAR|XP_007409882.1 MLAR|XP_007411546.1 MLAR|XP_007417633.1 MLAR|XP_007417704.1 MLAR|XP_007418423.1 MORY|QBZ54655.1 NCRA|XP_001728368.2 NCRA|XP_964218.1 SSCL|APA05461.1 SSCL|APA05462.1

>Orthogroup377: ANID|CBF80355.1 BCIN|XP_024546194.1 CFRU|XP_031878281.1 CFRU|XP_031888412.1 CFRU|XP_031889327.1 CFRU|XP_031893159.1 CGLO|KAF3798067.1 CGLO|KAF3803667.1 CGLO|KAF3809463.1 CHIG|XP_018152709.1 CHIG|XP_018155502.1 CHIG|XP_018163790.1 CVIN|KAF4927969.1 CVIN|KAF4928303.1 CVIN|KAF4928562.1 CVYL|A01062 CVYL|A08290 CVYL|A10030 CVYL|A14600 FGRM|XP_011322622.1 FGRM|XP_011323874.1 FGRM|XP_011327418.1 MGRA|XP_003851070.1 MORY|QBZ54627.1 MORY|QBZ58632.1 MORY|QBZ64462.1 MORY|QBZ65963.1 NCRA|XP_001728155.2 NCRA|XP_963873.1 SSCL|APA07036.1

>Orthogroup378: ANID|CBF81449.1 ANID|CBF86015.1 BCIN|XP_001556808.2 BCIN|XP_001561172.1 BCIN|XP_024548522.1 BGRA|VCU40367.1 BGRA|VCU40381.1 BGRA|VDB92625.1 CFRU|XP_031883568.1 CFRU|XP_031886175.1 CGLO|KAF3806562.1 CHIG|XP_018155080.1 CHIG|XP_018157602.1 CVIN|KAF4920471.1 CVIN|KAF4921783.1 CVYL|A02023 CVYL|A09307 FGRM|XP_011323503.1 FGRM|XP_011324357.1 MLAR|XP_007403826.1 MLAR|XP_007414766.1 MLAR|XP_007415353.1 MORY|QBZ53356.1 MORY|QBZ62388.1 NCRA|XP_959428.3 NCRA|XP_959869.2 SSCL|APA07106.1 SSCL|APA12112.1 SSCL|APA14585.1 SSCL|APA14586.1

>Orthogroup379: ANID|CBF82492.1 ANID|CBF90292.1 BCIN|XP_001546449.2 CFRU|XP_031877407.1 CFRU|XP_031880212.1 CFRU|XP_031886046.1 CGLO|KAF3801152.1 CGLO|KAF3808458.1 CGLO|KAF3808937.1 CHIG|XP_018155211.1 CHIG|XP_018155765.1 CHIG|XP_018161995.1 CVIN|KAF4904492.1 CVIN|KAF4915206.1 CVIN|KAF4920745.1 CVYL|A01153 CVYL|A05693 CVYL|A11683 FGRM|XP_011321988.1 FGRM|XP_011322506.1 FGRM|XP_011325950.1 FGRM|XP_011327255.1 FGRM|XP_011327499.1 MGRA|XP_003850272.1 MORY|QBZ56147.1 MORY|QBZ59261.1 MORY|QBZ64371.1 NCRA|XP_955885.1 NCRA|XP_963968.3 SSCL|APA08277.1

>Orthogroup380: ANID|CBF83000.1 ANID|CBF86704.1 BCIN|XP_024547550.1 BCIN|XP_024550309.1 BGRA|VDB85749.1 CFRU|XP_031878293.1 CFRU|XP_031881029.1 CFRU|XP_031889330.1 CFRU|XP_031889362.1 CGLO|KAF3797700.1 CGLO|KAF3798106.1 CGLO|KAF3809852.1 CHIG|XP_018155486.1 CVIN|KAF4927941.1 CVIN|KAF4928308.1 CVIN|KAF4928331.1 CVIN|KAF4930636.1 CVYL|A01023 CVYL|A01070 CVYL|A01762 CVYL|A08252 FGRM|XP_011321178.1 FGRM|XP_011327329.1 MGRA|XP_003849434.1 MGRA|XP_003856905.1 MORY|QBZ54964.1 MORY|QBZ57513.1 NCRA|XP_962891.1 SSCL|APA07859.1 SSCL|APA10974.1

>Orthogroup381: ANID|CBF83617.1 ANID|CBF84952.1 BCIN|XP_024548998.1 BCIN|XP_024549461.1 BGRA|VDB93284.1 CFRU|XP_031878288.1 CFRU|XP_031878669.1 CFRU|XP_031889465.1 CFRU|XP_031890198.1 CGLO|KAF3797543.1 CGLO|KAF3808830.1 CHIG|XP_018155747.1 CHIG|XP_018158456.1 CHIG|XP_018162835.1 CVIN|KAF4899966.1 CVIN|KAF4909982.1 CVIN|KAF4915600.1 CVIN|KAF4923542.1 CVYL|A01251 CVYL|A05155 CVYL|A08335 CVYL|A08996 FGRM|XP_011316416.1 FGRM|XP_011321967.1 MGRA|XP_003850426.1 MGRA|XP_003853150.1 MLAR|XP_007405232.1 MORY|QBZ55212.1 NCRA|XP_964545.2 SSCL|APA09339.1

>Orthogroup382: ANID|CBF85305.1 ANID|CBF88911.1 BCIN|XP_001551701.1 BCIN|XP_024547862.1 BCIN|XP_024548040.1 CFRU|XP_031887370.1 CFRU|XP_031890864.1 CFRU|XP_031893220.1 CGLO|KAF3801455.1 CGLO|KAF3809516.1 CHIG|XP_018151574.1 CHIG|XP_018151658.1 CHIG|XP_018152647.1 CHIG|XP_018154157.1 CHIG|XP_018156164.1 CHIG|XP_018157664.1 CHIG|XP_018162669.1 CVIN|KAF4912398.1 CVIN|KAF4918576.1 CVIN|KAF4924167.1 CVYL|A01543 CVYL|A08503 CVYL|A12670 MGRA|XP_003852880.1 MORY|QBZ56958.1 MORY|QBZ60719.1 NCRA|XP_011395057.1 SSCL|APA08184.1 SSCL|APA08280.1 SSCL|APA11612.1

>Orthogroup383: ANID|CBF87369.1 BCIN|XP_024552385.1 CFRU|XP_031881399.1 CFRU|XP_031889649.1 CFRU|XP_031892025.1 CFRU|XP_031892245.1 CGLO|KAF3801380.1 CGLO|KAF3801433.1 CGLO|KAF3805742.1 CGLO|KAF3807905.1 CGLO|KAF3810518.1 CHIG|XP_018151607.1 CHIG|XP_018161711.1 CHIG|XP_018164769.1 CVIN|KAF4896174.1 CVIN|KAF4907818.1 CVIN|KAF4914571.1 CVIN|KAF4922337.1 CVYL|A00324 CVYL|A07650 CVYL|A07702 CVYL|A09878 FGRM|XP_011321930.1 FGRM|XP_011323331.1 FGRM|XP_011324387.1 FGRM|XP_011325193.1 MORY|QBZ53726.1 MORY|QBZ55812.1 MORY|QBZ65095.1 MORY|QBZ65337.1

>Orthogroup384: ANID|CBF88592.1 BCIN|XP_024549467.1 CFRU|XP_031878318.1 CFRU|XP_031885419.1 CFRU|XP_031887286.1 CFRU|XP_031889419.1 CGLO|KAF3798037.1 CGLO|KAF3802723.1 CGLO|KAF3806494.1 CGLO|KAF3808728.1 CGLO|KAF3809324.1 CHIG|XP_018155640.1 CHIG|XP_018156162.1 CHIG|XP_018157530.1 CHIG|XP_018158083.1 CVIN|KAF4919443.1 CVIN|KAF4924198.1 CVIN|KAF4927944.1 CVIN|KAF4929640.1 CVYL|A01526 CVYL|A08322 CVYL|A09374 FGRM|XP_011325810.1 FGRM|XP_011327744.1 FGRM|XP_011328145.1 FGRM|XP_011328750.1 FGRM|XP_011328754.1 MORY|QBZ56571.1 NCRA|XP_961794.1 SSCL|APA09190.1

>Orthogroup385: ANID|CBF89457.1 BCIN|XP_001560566.1 BCIN|XP_024552540.1 CFRU|XP_031876904.1 CFRU|XP_031884983.1 CFRU|XP_031886444.1 CGLO|KAF3811891.1 CGLO|KAF3812114.1 CHIG|XP_018151709.1 CHIG|XP_018153351.1 CHIG|XP_018153858.1 CHIG|XP_018155177.1 CVIN|KAF4907991.1 CVIN|KAF4918110.1 CVIN|KAF4920574.1 CVYL|A00284 CVYL|A06184 CVYL|A09689 FGRM|XP_011324452.1 FGRM|XP_011325323.1 MGRA|XP_003848785.1 MLAR|XP_007407137.1 MLAR|XP_007410340.1 MLAR|XP_007412734.1 MLAR|XP_007418265.1 MORY|QBZ60264.1 MORY|QBZ62005.1 MORY|QBZ62579.1 SSCL|APA07889.1 SSCL|APA08636.1

>Orthogroup386: BCIN|XP_001555310.2 BCIN|XP_024553408.1 CFRU|XP_031878448.1 CFRU|XP_031882654.1 CFRU|XP_031885894.1 CFRU|XP_031889884.1 CFRU|XP_031893144.1 CGLO|KAF3798337.1 CGLO|KAF3799790.1 CGLO|KAF3801139.1 CHIG|XP_018152438.1 CHIG|XP_018157052.1 CVIN|KAF4912452.1 CVIN|KAF4917792.1 CVYL|A04803 CVYL|A08231 CVYL|A13761 FGRM|XP_011320825.1 FGRM|XP_011321078.1 FGRM|XP_011323110.1 FGRM|XP_011325251.1 MGRA|XP_003851403.1 MGRA|XP_003852542.1 MLAR|XP_007409218.1 MLAR|XP_007412012.1 MLAR|XP_007412529.1 MLAR|XP_007414531.1 MORY|QBZ66407.1 NCRA|XP_958024.2 NCRA|XP_959737.2

>Orthogroup387: BCIN|XP_001545239.2 BGRA|VCU40575.1 CFRU|XP_031877133.1 CFRU|XP_031881267.1 CFRU|XP_031881913.1 CFRU|XP_031892131.1 CGLO|KAF3802553.1 CGLO|KAF3806086.1 CHIG|XP_018150955.1 CHIG|XP_018164588.1 CHIG|XP_018164787.1 CVIN|KAF4909969.1 CVIN|KAF4910739.1 CVIN|KAF4921875.1 CVIN|KAF4925207.1 CVYL|A02143 CVYL|A07426 CVYL|A07741 CVYL|A08811 FGRM|XP_011323747.1 FGRM|XP_011325234.1 FGRM|XP_011328570.1 MGRA|XP_003850224.1 MGRA|XP_003851117.1 MGRA|XP_003852540.1 MLAR|XP_007404033.1 MLAR|XP_007418334.1 MORY|QBZ59153.1 NCRA|XP_962360.2 SSCL|APA13846.1

>Orthogroup388: BGRA|VCU40586.1 BGRA|VCU40589.1 BGRA|VCU40707.1 BGRA|VCU40712.1 BGRA|VCU40766.1 BGRA|VCU40792.1 BGRA|VCU40896.1 BGRA|VCU40905.1 BGRA|VCU40913.1 BGRA|VDB85656.1 BGRA|VDB85658.1 BGRA|VDB85855.1 BGRA|VDB86186.1 BGRA|VDB87692.1 BGRA|VDB87699.1 BGRA|VDB87700.1 BGRA|VDB87709.1 BGRA|VDB87711.1 BGRA|VDB87713.1 BGRA|VDB87715.1 BGRA|VDB87717.1 BGRA|VDB87729.1 BGRA|VDB87735.1 BGRA|VDB87737.1 BGRA|VDB87739.1 BGRA|VDB87743.1 BGRA|VDB87745.1 BGRA|VDB88687.1 BGRA|VDB91355.1 BGRA|VDB91399.1

>Orthogroup389: CFRU|XP_031878724.1 CFRU|XP_031879351.1 CFRU|XP_031880942.1 CFRU|XP_031882078.1 CFRU|XP_031884251.1 CGLO|KAF3798328.1 CGLO|KAF3806020.1 CGLO|KAF3807003.1 CGLO|KAF3808612.1 CGLO|KAF3811071.1 CHIG|XP_018151147.1 CHIG|XP_018151176.1 CHIG|XP_018159412.1 CHIG|XP_018159659.1 CHIG|XP_018161338.1 CHIG|XP_018161904.1 CVIN|KAF4895121.1 CVIN|KAF4915269.1 CVIN|KAF4917824.1 CVIN|KAF4920311.1 CVIN|KAF4926136.1 CVYL|A06298 CVYL|A07492 CVYL|A09040 CVYL|A09723 CVYL|A12344 FGRM|XP_011318488.1 MGRA|XP_003847772.1 MORY|QBZ59830.1 MORY|QBZ64893.1

>Orthogroup390: MLAR|XP_007404744.1 MLAR|XP_007404892.1 MLAR|XP_007404893.1 MLAR|XP_007405582.1 MLAR|XP_007406430.1 MLAR|XP_007407429.1 MLAR|XP_007408279.1 MLAR|XP_007408611.1 MLAR|XP_007408612.1 MLAR|XP_007408790.1 MLAR|XP_007409579.1 MLAR|XP_007410419.1 MLAR|XP_007411573.1 MLAR|XP_007411597.1 MLAR|XP_007412101.1 MLAR|XP_007413018.1 MLAR|XP_007414282.1 MLAR|XP_007414608.1 MLAR|XP_007416182.1 MLAR|XP_007416255.1 MLAR|XP_007417787.1 MLAR|XP_007417884.1 MLAR|XP_007417957.1 MLAR|XP_007418264.1 MLAR|XP_007418863.1 MLAR|XP_007418980.1 MLAR|XP_007419080.1 MLAR|XP_007419637.1 MLAR|XP_007419721.1 MLAR|XP_007419837.1

>Orthogroup391: MLAR|XP_007404639.1 MLAR|XP_007404640.1 MLAR|XP_007404667.1 MLAR|XP_007404951.1 MLAR|XP_007406936.1 MLAR|XP_007408562.1 MLAR|XP_007408563.1 MLAR|XP_007409018.1 MLAR|XP_007409677.1 MLAR|XP_007409678.1 MLAR|XP_007409951.1 MLAR|XP_007411161.1 MLAR|XP_007411423.1 MLAR|XP_007411424.1 MLAR|XP_007411928.1 MLAR|XP_007412010.1 MLAR|XP_007412152.1 MLAR|XP_007413347.1 MLAR|XP_007415715.1 MLAR|XP_007415716.1 MLAR|XP_007415717.1 MLAR|XP_007416617.1 MLAR|XP_007416618.1 MLAR|XP_007417764.1 MLAR|XP_007418318.1 MLAR|XP_007419179.1 MLAR|XP_007419262.1 MLAR|XP_007419340.1 MLAR|XP_007419341.1 MLAR|XP_007419560.1

>Orthogroup392: MLAR|XP_007406404.1 MLAR|XP_007406522.1 MLAR|XP_007407308.1 MLAR|XP_007407319.1 MLAR|XP_007407321.1 MLAR|XP_007407323.1 MLAR|XP_007408055.1 MLAR|XP_007408056.1 MLAR|XP_007408756.1 MLAR|XP_007410192.1 MLAR|XP_007411798.1 MLAR|XP_007412248.1 MLAR|XP_007412249.1 MLAR|XP_007412526.1 MLAR|XP_007412629.1 MLAR|XP_007412746.1 MLAR|XP_007412759.1 MLAR|XP_007412769.1 MLAR|XP_007412834.1 MLAR|XP_007412835.1 MLAR|XP_007413499.1 MLAR|XP_007413620.1 MLAR|XP_007415723.1 MLAR|XP_007415892.1 MLAR|XP_007415894.1 MLAR|XP_007416172.1 MLAR|XP_007417273.1 MLAR|XP_007417392.1 MLAR|XP_007418250.1 MLAR|XP_007418251.1

>Orthogroup393: ANID|CBF69377.1 ANID|CBF81789.1 ANID|CBF87415.1 CFRU|XP_031876279.1 CFRU|XP_031881232.1 CFRU|XP_031881903.1 CFRU|XP_031886064.1 CGLO|KAF3801162.1 CGLO|KAF3805994.1 CGLO|KAF3810001.1 CGLO|KAF3810999.1 CHIG|XP_018154867.1 CHIG|XP_018157731.1 CHIG|XP_018164659.1 CVIN|KAF4911055.1 CVIN|KAF4912999.1 CVIN|KAF4921846.1 CVIN|KAF4925746.1 CVYL|A02155 CVYL|A02602 CVYL|A06226 CVYL|A07521 FGRM|XP_011318383.1 FGRM|XP_011321954.1 FGRM|XP_011322390.1 MGRA|XP_003849209.1 MORY|QBZ56152.1 MORY|QBZ66664.1 NCRA|XP_958752.1

>Orthogroup394: ANID|CBF70172.1 BCIN|XP_001554464.1 CFRU|XP_031875722.1 CFRU|XP_031877524.1 CFRU|XP_031883111.1 CFRU|XP_031890093.1 CFRU|XP_031891937.1 CGLO|KAF3797517.1 CGLO|KAF3798152.1 CGLO|KAF3798259.1 CGLO|KAF3800148.1 CGLO|KAF3808256.1 CHIG|XP_018150803.1 CHIG|XP_018151322.1 CHIG|XP_018153789.1 CHIG|XP_018154046.1 CHIG|XP_018165082.1 CVIN|KAF4900286.1 CVIN|KAF4916931.1 CVIN|KAF4919141.1 CVIN|KAF4921631.1 CVIN|KAF4925863.1 CVYL|A00592 CVYL|A01965 CVYL|A04961 CVYL|A06840 CVYL|A09242 FGRM|XP_011323329.1 FGRM|XP_011325263.1

>Orthogroup395: ANID|CBF70342.1 ANID|CBF85458.1 BCIN|XP_001548318.2 BGRA|VDB95081.1 CFRU|XP_031877978.1 CFRU|XP_031880766.1 CFRU|XP_031886505.1 CFRU|XP_031892580.1 CGLO|KAF3803057.1 CGLO|KAF3806328.1 CGLO|KAF3810890.1 CGLO|KAF3811193.1 CHIG|XP_018153549.1 CHIG|XP_018154923.1 CVIN|KAF4916375.1 CVIN|KAF4919878.1 CVIN|KAF4923934.1 CVIN|KAF4931250.1 CVYL|A00066 CVYL|A02252 CVYL|A04357 CVYL|A11916 FGRM|XP_011316908.1 FGRM|XP_011322706.1 MGRA|XP_003856714.1 MLAR|XP_007408783.1 MORY|QBZ63170.1 NCRA|XP_964169.1 SSCL|APA06374.1

>Orthogroup396: ANID|CBF70354.1 ANID|CBF73614.1 ANID|CBF77326.1 ANID|CBF87974.1 BCIN|XP_001558531.1 BCIN|XP_001559974.1 BCIN|XP_024548815.1 BCIN|XP_024551924.1 BGRA|VCU39960.1 CFRU|XP_031876649.1 CFRU|XP_031890299.1 CGLO|KAF3801908.1 CGLO|KAF3802926.1 CHIG|XP_018158528.1 CVIN|KAF4918711.1 CVIN|KAF4925584.1 CVYL|A02348 CVYL|A05090 FGRM|XP_011320615.1 FGRM|XP_011322236.1 MGRA|XP_003848106.1 MGRA|XP_003855857.1 MGRA|XP_003857793.1 MLAR|XP_007416101.1 MORY|QBZ63209.1 SSCL|APA06362.1 SSCL|APA09628.1 SSCL|APA09787.1 SSCL|APA14671.1

>Orthogroup397: ANID|CBF70690.1 ANID|CBF74338.1 ANID|CBF82877.1 BCIN|XP_001551177.1 BCIN|XP_001552597.1 CFRU|XP_031879671.1 CFRU|XP_031881034.1 CFRU|XP_031891776.1 CGLO|KAF3801398.1 CGLO|KAF3809884.1 CHIG|XP_018154077.1 CHIG|XP_018161470.1 CHIG|XP_018164781.1 CVIN|KAF4919110.1 CVIN|KAF4922326.1 CVIN|KAF4930625.1 CVYL|A01733 CVYL|A04950 CVYL|A07684 FGRM|XP_011322815.1 FGRM|XP_011323525.1 FGRM|XP_011327363.1 MGRA|XP_003850546.1 MGRA|XP_003855264.1 MORY|QBZ63353.1 NCRA|XP_956001.1 NCRA|XP_960171.3 SSCL|APA11448.1 SSCL|APA13207.1

>Orthogroup398: ANID|CBF70950.1 ANID|CBF85886.1 BCIN|XP_001557229.1 BCIN|XP_024553767.1 BGRA|VDB84307.1 BGRA|VDB93140.1 CFRU|XP_031886875.1 CFRU|XP_031887388.1 CGLO|KAF3809291.1 CGLO|KAF3811238.1 CHIG|XP_018156119.1 CHIG|XP_018160461.1 CVIN|KAF4921078.1 CVIN|KAF4931164.1 CVYL|A01486 CVYL|A04427 FGRM|XP_011319355.1 FGRM|XP_011327086.1 MGRA|XP_003849124.1 MGRA|XP_003855988.1 MLAR|XP_007407629.1 MLAR|XP_007412541.1 MLAR|XP_007416800.1 MORY|QBZ59025.1 MORY|QBZ64757.1 NCRA|XP_011394625.1 NCRA|XP_961238.1 SSCL|APA12510.1 SSCL|APA15139.1

>Orthogroup399: ANID|CBF71201.1 ANID|CBF74964.1 ANID|CBF79744.1 ANID|CBF89341.1 CFRU|XP_031876476.1 CFRU|XP_031883944.1 CFRU|XP_031893615.1 CGLO|KAF3798019.1 CGLO|KAF3800819.1 CGLO|KAF3805015.1 CGLO|KAF3811698.1 CHIG|XP_018150480.1 CHIG|XP_018150854.1 CHIG|XP_018152828.1 CVIN|KAF4915604.1 CVIN|KAF4922491.1 CVIN|KAF4927131.1 CVYL|A04563 CVYL|A08338 CVYL|A08730 CVYL|A09999 FGRM|XP_011319559.1 FGRM|XP_011320922.1 FGRM|XP_011320923.1 FGRM|XP_011327349.1 MORY|QBZ53679.1 NCRA|XP_961930.1 NCRA|XP_965031.2 SSCL|APA08444.1

>Orthogroup400: ANID|CBF71211.1 ANID|CBF74011.1 ANID|CBF78356.1 ANID|CBF80599.1 ANID|CBF84289.1 BCIN|XP_024546016.1 BCIN|XP_024552812.1 CFRU|XP_031876387.1 CFRU|XP_031879725.1 CFRU|XP_031884959.1 CFRU|XP_031893558.1 CGLO|KAF3797787.1 CGLO|KAF3798554.1 CGLO|KAF3800982.1 CGLO|KAF3809527.1 CHIG|XP_018152671.1 CHIG|XP_018156483.1 CHIG|XP_018161407.1 CVIN|KAF4913271.1 CVIN|KAF4923160.1 CVIN|KAF4930172.1 CVYL|A03012 CVYL|A05924 CVYL|A08494 FGRM|XP_011318108.1 FGRM|XP_011318356.1 FGRM|XP_011319851.1 MGRA|XP_003855177.1 SSCL|APA16020.1

>Orthogroup401: ANID|CBF71352.1 ANID|CBF82382.1 BCIN|XP_024549994.1 BCIN|XP_024550046.1 BGRA|VDB93480.1 CFRU|XP_031880538.1 CFRU|XP_031882246.1 CFRU|XP_031888855.1 CGLO|KAF3804260.1 CGLO|KAF3804333.1 CGLO|KAF3811573.1 CHIG|XP_018160115.1 CHIG|XP_018160837.1 CVIN|KAF4909067.1 CVIN|KAF4914879.1 CVIN|KAF4920149.1 CVYL|A03420 CVYL|A03941 CVYL|A04026 FGRM|XP_011327925.1 FGRM|XP_011328377.1 MLAR|XP_007414018.1 MORY|QBZ54764.1 MORY|QBZ56619.1 MORY|QBZ59161.1 NCRA|XP_011394144.1 NCRA|XP_011394466.1 SSCL|APA05336.1 SSCL|APA14469.1

>Orthogroup402: ANID|CBF73451.1 ANID|CBF73473.1 ANID|CBF73482.1 ANID|CBF77612.1 ANID|CBF80209.1 ANID|CBF89305.1 BCIN|XP_001553479.1 BCIN|XP_024548368.1 BGRA|VDB93243.1 CFRU|XP_031878582.1 CFRU|XP_031886441.1 CFRU|XP_031892061.1 CFRU|XP_031893332.1 CGLO|KAF3798395.1 CGLO|KAF3798481.1 CGLO|KAF3801296.1 CGLO|KAF3806348.1 CHIG|XP_018155696.1 CHIG|XP_018164847.1 CVIN|KAF4899318.1 CVIN|KAF4921596.1 CVYL|A07790 CVYL|A08211 FGRM|XP_011319335.1 MGRA|XP_003851358.1 MLAR|XP_007415704.1 MORY|QBZ58939.1 NCRA|XP_960678.2 SSCL|APA12346.1

>Orthogroup403: ANID|CBF73711.1 ANID|CBF83723.1 BCIN|XP_001561381.1 BCIN|XP_024547048.1 BGRA|VCU40639.1 BGRA|VDB90701.1 CFRU|XP_031888435.1 CFRU|XP_031888866.1 CGLO|KAF3797644.1 CGLO|KAF3799322.1 CGLO|KAF3803461.1 CHIG|XP_018159918.1 CHIG|XP_018163989.1 CVIN|KAF4924766.1 CVIN|KAF4926353.1 CVYL|A04208 CVYL|A14390 FGRM|XP_011324459.1 FGRM|XP_011326494.1 MGRA|XP_003849946.1 MGRA|XP_003855739.1 MLAR|XP_007408064.1 MLAR|XP_007417615.1 MORY|QBZ59183.1 MORY|QBZ63470.1 NCRA|XP_958421.2 NCRA|XP_961600.1 SSCL|APA06893.1 SSCL|APA14507.1

>Orthogroup404: ANID|CBF73880.1 ANID|CBF86691.1 BCIN|XP_001552825.2 BCIN|XP_001553230.2 CFRU|XP_031875630.1 CFRU|XP_031877436.1 CFRU|XP_031882809.1 CFRU|XP_031888031.1 CGLO|KAF3797030.1 CGLO|KAF3802037.1 CGLO|KAF3805339.1 CGLO|KAF3806642.1 CHIG|XP_018162013.1 CVIN|KAF4898426.1 CVIN|KAF4910506.1 CVIN|KAF4918932.1 CVIN|KAF4931548.1 CVIN|KAF4931570.1 CVYL|A03062 CVYL|A03351 CVYL|A11718 CVYL|A12354 FGRM|XP_011318484.1 FGRM|XP_011320829.1 FGRM|XP_011327530.1 MGRA|XP_003853890.1 MORY|QBZ61666.1 MORY|QBZ61667.1 SSCL|APA12067.1

>Orthogroup405: ANID|CBF73893.1 ANID|CBF78507.1 BCIN|XP_024546426.1 CFRU|XP_031877971.1 CFRU|XP_031878406.1 CFRU|XP_031882688.1 CFRU|XP_031887404.1 CFRU|XP_031892805.1 CGLO|KAF3797062.1 CGLO|KAF3803863.1 CGLO|KAF3809378.1 CGLO|KAF3810743.1 CGLO|KAF3810901.1 CHIG|XP_018154934.1 CHIG|XP_018156878.1 CHIG|XP_018157034.1 CVIN|KAF4894648.1 CVIN|KAF4896771.1 CVIN|KAF4916343.1 CVIN|KAF4932016.1 CVYL|A02244 CVYL|A07208 CVYL|A08937 FGRM|XP_011315836.1 FGRM|XP_011321089.1 FGRM|XP_011323068.1 FGRM|XP_011325485.1 MORY|QBZ53627.1 NCRA|XP_011395059.1

>Orthogroup406: ANID|CBF73906.1 ANID|CBF82620.1 ANID|CBF84249.1 BCIN|XP_001552084.1 CFRU|XP_031880125.1 CFRU|XP_031884479.1 CFRU|XP_031889360.1 CFRU|XP_031890716.1 CFRU|XP_031891850.1 CGLO|KAF3799781.1 CGLO|KAF3801352.1 CGLO|KAF3801574.1 CGLO|KAF3803137.1 CHIG|XP_018155195.1 CHIG|XP_018158218.1 CVIN|KAF4913325.1 CVIN|KAF4918949.1 CVIN|KAF4921086.1 CVIN|KAF4928313.1 CVYL|A01022 CVYL|A07729 CVYL|A11314 CVYL|A12559 FGRM|XP_011323393.1 MGRA|XP_003852951.1 MGRA|XP_003856915.1 MORY|QBZ55904.1 NCRA|XP_964807.1 SSCL|APA09814.1

>Orthogroup407: ANID|CBF74797.1 ANID|CBF78989.1 BCIN|XP_001557273.1 BCIN|XP_001557587.1 BCIN|XP_001558301.1 BGRA|VDB92994.1 CFRU|XP_031879914.1 CFRU|XP_031881317.1 CFRU|XP_031889112.1 CGLO|KAF3799005.1 CGLO|KAF3802453.1 CGLO|KAF3803090.1 CHIG|XP_018151303.1 CHIG|XP_018154751.1 CVIN|KAF4916863.1 CVIN|KAF4923445.1 CVIN|KAF4929557.1 CVYL|A00982 CVYL|A02043 CVYL|A07922 FGRM|XP_011327473.1 MGRA|XP_003852668.1 MGRA|XP_003856964.1 MORY|QBZ62741.1 MORY|QBZ65079.1 NCRA|XP_956851.1 SSCL|APA12319.1 SSCL|APA12491.1 SSCL|APA13686.1

>Orthogroup408: ANID|CBF75342.1 ANID|CBF77396.1 BCIN|XP_001550994.1 BCIN|XP_001560049.1 BGRA|VCU40256.1 BGRA|VDB90676.1 CFRU|XP_031884656.1 CFRU|XP_031890553.1 CGLO|KAF3804920.1 CGLO|KAF3810307.1 CHIG|XP_018158333.1 CHIG|XP_018164418.1 CVIN|KAF4913289.1 CVIN|KAF4919419.1 CVYL|A05797 CVYL|A12136 FGRM|XP_011316491.1 FGRM|XP_011317144.1 MGRA|XP_003855845.1 MLAR|XP_007406841.1 MLAR|XP_007414050.1 MLAR|XP_007414348.1 MLAR|XP_007417667.1 MORY|QBZ56463.1 MORY|QBZ58456.1 NCRA|XP_964367.2 NCRA|XP_964451.2 SSCL|APA07061.1 SSCL|APA09921.1

>Orthogroup409: ANID|CBF75908.1 ANID|CBF76901.1 ANID|CBF85903.1 BCIN|XP_024550814.1 BGRA|VCU39031.1 CFRU|XP_031885415.1 CFRU|XP_031888054.1 CGLO|KAF3799428.1 CGLO|KAF3802267.1 CHIG|XP_018152300.1 CHIG|XP_018154368.1 CVIN|KAF4924433.1 CVIN|KAF4931730.1 CVYL|A10274 CVYL|A13292 FGRM|XP_011319814.1 FGRM|XP_011319817.1 FGRM|XP_011323654.1 MGRA|XP_003853160.1 MGRA|XP_003857027.1 MLAR|XP_007403867.1 MLAR|XP_007415639.1 MLAR|XP_007417104.1 MORY|QBZ64789.1 MORY|QBZ65722.1 NCRA|XP_956370.1 NCRA|XP_964324.1 SSCL|APA07257.1 SSCL|APA11833.1

>Orthogroup410: ANID|CBF76105.1 ANID|CBF76449.1 BCIN|XP_001551135.2 BCIN|XP_001556654.1 BCIN|XP_024546292.1 BCIN|XP_024547216.1 BCIN|XP_024547481.1 BCIN|XP_024551716.1 BCIN|XP_024552334.1 BGRA|VDB92925.1 CFRU|XP_031882303.1 CFRU|XP_031884889.1 CGLO|KAF3804420.1 CHIG|XP_018153246.1 CVIN|KAF4912993.1 CVIN|KAF4930889.1 CVYL|A04124 CVYL|A05909 FGRM|XP_011321602.1 MGRA|XP_003852277.1 MGRA|XP_003854795.1 MLAR|XP_007414648.1 MORY|QBZ57854.1 MORY|QBZ59402.1 NCRA|XP_958013.2 SSCL|APA07656.1 SSCL|APA13186.1 SSCL|APA14784.1 SSCL|APA15053.1

>Orthogroup411: ANID|CBF76406.1 ANID|CBF81299.1 BCIN|XP_001557236.1 BCIN|XP_024551739.1 BGRA|VDB93236.1 CFRU|XP_031884863.1 CFRU|XP_031885356.1 CGLO|KAF3799507.1 CGLO|KAF3801037.1 CHIG|XP_018153294.1 CHIG|XP_018154494.1 CVIN|KAF4908692.1 CVIN|KAF4931867.1 CVYL|A00347 CVYL|A13368 FGRM|XP_011317474.1 FGRM|XP_011321206.1 FGRM|XP_011321578.1 MGRA|XP_003848081.1 MGRA|XP_003854816.1 MLAR|XP_007411372.1 MLAR|XP_007412412.1 MLAR|XP_007417133.1 MORY|QBZ53847.1 MORY|QBZ57280.1 NCRA|XP_956761.2 NCRA|XP_962032.3 SSCL|APA12515.1 SSCL|APA14605.1

>Orthogroup412: ANID|CBF76882.1 ANID|CBF79476.1 BCIN|XP_001545692.1 BCIN|XP_024546048.1 BGRA|VCU39088.1 BGRA|VDB93614.1 CFRU|XP_031876918.1 CFRU|XP_031888021.1 CGLO|KAF3802239.1 CGLO|KAF3810714.1 CHIG|XP_018154346.1 CHIG|XP_018162569.1 CVIN|KAF4908236.1 CVIN|KAF4914844.1 CVYL|A03160 CVYL|A07239 FGRM|XP_011319855.1 FGRM|XP_011323985.1 MGRA|XP_003847656.1 MGRA|XP_003854411.1 MLAR|XP_007404973.1 MLAR|XP_007406025.1 MLAR|XP_007406998.1 MORY|QBZ62842.1 MORY|QBZ66028.1 NCRA|XP_011393921.1 NCRA|XP_964519.3 SSCL|APA07272.1 SSCL|APA14943.1

>Orthogroup413: ANID|CBF79006.1 BCIN|XP_001551952.1 BGRA|VDB94526.1 CFRU|XP_031887701.1 CFRU|XP_031887702.1 CFRU|XP_031889109.1 CGLO|KAF3803220.1 CHIG|XP_018154001.1 CHIG|XP_018155582.1 CHIG|XP_018161072.1 CVIN|KAF4929552.1 CVYL|A00979 CVYL|A03650 FGRM|XP_011323267.1 MGRA|XP_003848294.1 MGRA|XP_003849628.1 MGRA|XP_003851040.1 MGRA|XP_003853386.1 MGRA|XP_003853586.1 MGRA|XP_003854342.1 MGRA|XP_003854533.1 MGRA|XP_003854539.1 MGRA|XP_003855898.1 MLAR|XP_007406838.1 MORY|QBZ53641.1 MORY|QBZ56253.1 MORY|QBZ58290.1 MORY|QBZ60835.1 NCRA|XP_958301.1

>Orthogroup414: ANID|CBF80822.1 ANID|CBF90049.1 BCIN|XP_001546606.1 BGRA|VDB87850.1 BGRA|VDB90628.1 BGRA|VDB92802.1 BGRA|VDB92807.1 CFRU|XP_031891639.1 CFRU|XP_031893029.1 CGLO|KAF3797915.1 CGLO|KAF3800834.1 CHIG|XP_018150873.1 CHIG|XP_018161605.1 CVIN|KAF4907835.1 CVIN|KAF4922460.1 CVYL|A05350 CVYL|A08714 FGRM|XP_011318654.1 FGRM|XP_011319391.1 FGRM|XP_011321720.1 FGRM|XP_011328055.1 MGRA|XP_003849742.1 MLAR|XP_007406244.1 MLAR|XP_007413001.1 MLAR|XP_007413002.1 MLAR|XP_007413124.1 MORY|QBZ64460.1 NCRA|XP_962263.3 SSCL|APA13033.1

>Orthogroup415: ANID|CBF81448.1 CFRU|XP_031890317.1 CFRU|XP_031890853.1 CFRU|XP_031891251.1 CGLO|KAF3804653.1 CGLO|KAF3808337.1 CGLO|KAF3811673.1 CHIG|XP_018151855.1 CHIG|XP_018155494.1 CHIG|XP_018161705.1 CVIN|KAF4904108.1 CVIN|KAF4913897.1 CVIN|KAF4915951.1 CVYL|A05232 CVYL|A09218 CVYL|A12696 FGRM|XP_011322008.1 FGRM|XP_011328394.1 MORY|QBZ53781.1 MORY|QBZ55122.1 MORY|QBZ61249.1 MORY|QBZ61928.1 MORY|QBZ65199.1 MORY|QBZ66310.1 NCRA|XP_011395350.1 NCRA|XP_958969.1 NCRA|XP_959051.1 NCRA|XP_959145.2 NCRA|XP_959657.2

>Orthogroup416: ANID|CBF82519.1 ANID|CBF88940.1 BCIN|XP_024547377.1 BCIN|XP_024551044.1 CFRU|XP_031875855.1 CFRU|XP_031876848.1 CFRU|XP_031877568.1 CFRU|XP_031877598.1 CFRU|XP_031878971.1 CFRU|XP_031880555.1 CFRU|XP_031891756.1 CFRU|XP_031892762.1 CGLO|KAF3797081.1 CGLO|KAF3797990.1 CGLO|KAF3800108.1 CHIG|XP_018151880.1 CHIG|XP_018157029.1 CHIG|XP_018165045.1 CVIN|KAF4911393.1 CVIN|KAF4925853.1 CVYL|A06799 CVYL|A08363 CVYL|A10627 FGRM|XP_011321411.1 FGRM|XP_011325928.1 MGRA|XP_003852735.1 MORY|QBZ53701.1 MORY|QBZ66463.1 SSCL|APA07010.1

>Orthogroup417: ANID|CBF83207.1 ANID|CBF83959.1 ANID|CBF85148.1 BCIN|XP_001554308.2 BCIN|XP_001556149.1 BCIN|XP_024546831.1 CFRU|XP_031888965.1 CFRU|XP_031889342.1 CFRU|XP_031893301.1 CGLO|KAF3803114.1 CGLO|KAF3809667.1 CHIG|XP_018152530.1 CHIG|XP_018155609.1 CVIN|KAF4909311.1 CVIN|KAF4925040.1 CVIN|KAF4928319.1 CVYL|A01002 CVYL|A08383 CVYL|A10110 FGRM|XP_011322476.1 FGRM|XP_011325870.1 MGRA|XP_003847795.1 MGRA|XP_003855471.1 MORY|QBZ58581.1 MORY|QBZ61907.1 MORY|QBZ63298.1 NCRA|XP_963801.1 NCRA|XP_964352.1 SSCL|APA06051.1

>Orthogroup418: ANID|CBF84130.1 BCIN|XP_001559023.1 BGRA|VDB90668.1 CFRU|XP_031877490.1 CFRU|XP_031883624.1 CFRU|XP_031890474.1 CGLO|KAF3798417.1 CGLO|KAF3804855.1 CGLO|KAF3810831.1 CHIG|XP_018155028.1 CHIG|XP_018161238.1 CHIG|XP_018164412.1 CVIN|KAF4911115.1 CVIN|KAF4920028.1 CVIN|KAF4921143.1 CVYL|A01994 CVYL|A11218 CVYL|A12218 FGRM|XP_011316823.1 FGRM|XP_011318046.1 FGRM|XP_011325951.1 MGRA|XP_003852311.1 MLAR|XP_007416306.1 MORY|QBZ59493.1 MORY|QBZ63628.1 MORY|QBZ64590.1 NCRA|XP_957510.3 NCRA|XP_965335.1 SSCL|APA09249.1

>Orthogroup419: ANID|CBF84460.1 CFRU|XP_031880243.1 CFRU|XP_031883417.1 CFRU|XP_031883679.1 CFRU|XP_031884624.1 CFRU|XP_031886353.1 CFRU|XP_031887848.1 CFRU|XP_031890994.1 CGLO|KAF3799066.1 CGLO|KAF3801510.1 CGLO|KAF3801748.1 CGLO|KAF3802191.1 CGLO|KAF3806260.1 CGLO|KAF3808958.1 CGLO|KAF3811918.1 CHIG|XP_018151938.1 CHIG|XP_018155811.1 CVIN|KAF4895631.1 CVIN|KAF4916389.1 CVIN|KAF4918243.1 CVIN|KAF4920727.1 CVIN|KAF4922754.1 CVIN|KAF4931459.1 CVYL|A01128 CVYL|A03202 CVYL|A11849 CVYL|A12621 FGRM|XP_011315749.1 MGRA|XP_003856275.1

>Orthogroup420: ANID|CBF85149.1 BCIN|XP_024552276.1 CFRU|XP_031883805.1 CFRU|XP_031884077.1 CFRU|XP_031884709.1 CFRU|XP_031884710.1 CGLO|KAF3801021.1 CGLO|KAF3805170.1 CHIG|XP_018150593.1 CHIG|XP_018150627.1 CHIG|XP_018150644.1 CHIG|XP_018156649.1 CHIG|XP_018156650.1 CHIG|XP_018163345.1 CVIN|KAF4927476.1 CVYL|A00363 CVYL|A05887 CVYL|A06581 MGRA|XP_003849912.1 MGRA|XP_003851420.1 MLAR|XP_007407531.1 MLAR|XP_007415474.1 MORY|QBZ53453.1 MORY|QBZ53535.1 MORY|QBZ53668.1 NCRA|XP_011393383.1 NCRA|XP_011395091.1 NCRA|XP_956102.2 SSCL|APA13139.1

>Orthogroup421: ANID|CBF88798.1 BCIN|XP_001547687.1 CFRU|XP_031881265.1 CFRU|XP_031886158.1 CFRU|XP_031887715.1 CFRU|XP_031888698.1 CFRU|XP_031893287.1 CGLO|KAF3802556.1 CGLO|KAF3803314.1 CGLO|KAF3804027.1 CGLO|KAF3804301.1 CGLO|KAF3811990.1 CHIG|XP_018151653.1 CHIG|XP_018161166.1 CVIN|KAF4898741.1 CVIN|KAF4920114.1 CVIN|KAF4921858.1 CVIN|KAF4922482.1 CVIN|KAF4930431.1 CVYL|A02146 CVYL|A03749 CVYL|A03983 CVYL|A05517 CVYL|A08777 FGRM|XP_011321364.1 MGRA|XP_003855655.1 MORY|QBZ64582.1 NCRA|XP_001728517.2 SSCL|APA13308.1

>Orthogroup422: BCIN|XP_001546351.1 BCIN|XP_001555596.1 BCIN|XP_001560671.2 BCIN|XP_024551153.1 CFRU|XP_031875991.1 CFRU|XP_031879278.1 CFRU|XP_031883452.1 CFRU|XP_031885920.1 CGLO|KAF3808604.1 CGLO|KAF3810825.1 CHIG|XP_018151565.1 CHIG|XP_018155094.1 CHIG|XP_018161556.1 CVIN|KAF4904634.1 CVIN|KAF4918296.1 CVIN|KAF4926159.1 CVYL|A02002 CVYL|A05631 CVYL|A12378 FGRM|XP_011317400.1 FGRM|XP_011327622.1 MGRA|XP_003848811.1 MORY|QBZ57577.1 MORY|QBZ58424.1 MORY|QBZ60717.1 MORY|QBZ64958.1 NCRA|XP_964773.2 SSCL|APA07161.1 SSCL|APA15015.1

>Orthogroup423: BCIN|XP_001551740.1 BCIN|XP_024546430.1 CFRU|XP_031881586.1 CFRU|XP_031892755.1 CGLO|KAF3797205.1 CGLO|KAF3800077.1 CGLO|KAF3805409.1 CHIG|XP_018157304.1 CHIG|XP_018161372.1 CVIN|KAF4891451.1 CVIN|KAF4917748.1 CVIN|KAF4920811.1 CVYL|A06776 CVYL|A10492 CVYL|A11056 FGRM|XP_011320039.1 FGRM|XP_011322002.1 MGRA|XP_003847705.1 MGRA|XP_003847874.1 MGRA|XP_003853489.1 MLAR|XP_007406355.1 MLAR|XP_007408745.1 MLAR|XP_007408746.1 MLAR|XP_007414971.1 MORY|QBZ53635.1 MORY|QBZ65058.1 MORY|QBZ65979.1 NCRA|XP_964615.1 SSCL|APA12006.1

>Orthogroup424: BCIN|XP_024548528.1 BCIN|XP_024550187.1 BCIN|XP_024552305.1 CFRU|XP_031878773.1 CFRU|XP_031878983.1 CFRU|XP_031886363.1 CGLO|KAF3806401.1 CGLO|KAF3807830.1 CHIG|XP_018152491.1 CHIG|XP_018156499.1 CHIG|XP_018161317.1 CHIG|XP_018162285.1 CHIG|XP_018162980.1 CVIN|KAF4894261.1 CVIN|KAF4911607.1 CVIN|KAF4923276.1 CVYL|A00777 CVYL|A01891 CVYL|A11993 FGRM|XP_011320517.1 MGRA|XP_003848190.1 MORY|QBZ56232.1 NCRA|XP_958149.3 NCRA|XP_960357.2 NCRA|XP_960519.1 NCRA|XP_962620.2 SSCL|APA10866.1 SSCL|APA12100.1 SSCL|APA13268.1

>Orthogroup425: BCIN|XP_001554138.1 BGRA|VCU41289.1 BGRA|VDB87571.1 BGRA|VDB87584.1 BGRA|VDB87588.1 BGRA|VDB87590.1 BGRA|VDB87596.1 BGRA|VDB88027.1 CFRU|XP_031878829.1 CFRU|XP_031878879.1 CFRU|XP_031878979.1 CGLO|KAF3802610.1 CGLO|KAF3802636.1 CGLO|KAF3807887.1 CHIG|XP_018156634.1 CHIG|XP_018161288.1 CHIG|XP_018161304.1 CVIN|KAF4911616.1 CVIN|KAF4921662.1 CVIN|KAF4922602.1 CVYL|A00726 CVYL|A01898 CVYL|A01930 MGRA|XP_003848621.1 MGRA|XP_003849150.1 MGRA|XP_003854832.1 MGRA|XP_003856155.1 NCRA|XP_962439.2 SSCL|APA05526.1

>Orthogroup426: BCIN|XP_001551556.1 BCIN|XP_024552883.1 BGRA|VDB87616.1 CFRU|XP_031878127.1 CFRU|XP_031879406.1 CFRU|XP_031887921.1 CGLO|KAF3799639.1 CGLO|KAF3801804.1 CGLO|KAF3802141.1 CHIG|XP_018154295.1 CHIG|XP_018154648.1 CHIG|XP_018159302.1 CVIN|KAF4912436.1 CVIN|KAF4916973.1 CVIN|KAF4926478.1 CVYL|A03252 CVYL|A12901 FGRM|XP_011320060.1 MGRA|XP_003852726.1 MLAR|XP_007405616.1 MLAR|XP_007412690.1 MLAR|XP_007417952.1 MORY|QBZ61013.1 MORY|QBZ66006.1 MORY|QBZ66499.1 NCRA|XP_958155.2 NCRA|XP_964559.1 SSCL|APA11893.1 SSCL|APA16092.1

>Orthogroup427: BGRA|VCU38847.1 BGRA|VCU38955.1 BGRA|VCU39000.1 BGRA|VCU39194.1 BGRA|VCU39333.1 BGRA|VCU39831.1 BGRA|VCU40790.1 BGRA|VCU40868.1 BGRA|VCU40911.1 BGRA|VCU40926.1 BGRA|VCU41031.1 BGRA|VDB83596.1 BGRA|VDB83602.1 BGRA|VDB83855.1 BGRA|VDB84044.1 BGRA|VDB84225.1 BGRA|VDB84442.1 BGRA|VDB85652.1 BGRA|VDB85785.1 BGRA|VDB87790.1 BGRA|VDB88275.1 BGRA|VDB88347.1 BGRA|VDB88349.1 BGRA|VDB89360.1 BGRA|VDB93133.1 BGRA|VDB94516.1 BGRA|VDB94534.1 BGRA|VDB94775.1 BGRA|VDB96359.1

>Orthogroup428: BGRA|VCU38881.1 BGRA|VCU38884.1 BGRA|VCU38896.1 BGRA|VCU38899.1 BGRA|VCU38905.1 BGRA|VCU38911.1 BGRA|VCU39040.1 BGRA|VCU39230.1 BGRA|VCU39232.1 BGRA|VCU39233.1 BGRA|VDB85767.1 BGRA|VDB85772.1 BGRA|VDB87973.1 BGRA|VDB93030.1 BGRA|VDB93096.1 BGRA|VDB93102.1 BGRA|VDB93103.1 BGRA|VDB94462.1 BGRA|VDB94464.1 BGRA|VDB94468.1 BGRA|VDB94469.1 BGRA|VDB94470.1 BGRA|VDB94474.1 BGRA|VDB94491.1 BGRA|VDB94512.1 BGRA|VDB94518.1 BGRA|VDB96412.1 BGRA|VDB96413.1 BGRA|VDB96415.1

>Orthogroup429: BGRA|VCU39504.1 BGRA|VCU39916.1 BGRA|VCU39919.1 BGRA|VCU40032.1 BGRA|VCU40125.1 BGRA|VCU40678.1 BGRA|VCU40685.1 BGRA|VCU40929.1 BGRA|VCU40958.1 BGRA|VCU40961.1 BGRA|VCU41047.1 BGRA|VCU41057.1 BGRA|VCU41171.1 BGRA|VDB83830.1 BGRA|VDB84054.1 BGRA|VDB84069.1 BGRA|VDB84257.1 BGRA|VDB84383.1 BGRA|VDB87666.1 BGRA|VDB89289.1 BGRA|VDB89385.1 BGRA|VDB90695.1 BGRA|VDB90956.1 BGRA|VDB91108.1 BGRA|VDB92672.1 BGRA|VDB93660.1 BGRA|VDB93681.1 BGRA|VDB94812.1 BGRA|VDB95154.1

>Orthogroup430: CFRU|XP_031877239.1 CFRU|XP_031878122.1 CFRU|XP_031879297.1 CFRU|XP_031880946.1 CFRU|XP_031885223.1 CFRU|XP_031891195.1 CGLO|KAF3800330.1 CGLO|KAF3801806.1 CGLO|KAF3804588.1 CGLO|KAF3806848.1 CGLO|KAF3806858.1 CGLO|KAF3808576.1 CHIG|XP_018156645.1 CHIG|XP_018159545.1 CHIG|XP_018162923.1 CVIN|KAF4893132.1 CVIN|KAF4896546.1 CVIN|KAF4905056.1 CVIN|KAF4923058.1 CVIN|KAF4927780.1 CVYL|A02496 CVYL|A02505 CVYL|A05290 CVYL|A06893 CVYL|A11569 CVYL|A12897 FGRM|XP_011320703.1 FGRM|XP_011325775.1 MGRA|XP_003850267.1

>Orthogroup431: MLAR|XP_007403719.1 MLAR|XP_007405049.1 MLAR|XP_007405789.1 MLAR|XP_007406042.1 MLAR|XP_007406494.1 MLAR|XP_007408407.1 MLAR|XP_007408938.1 MLAR|XP_007409820.1 MLAR|XP_007409863.1 MLAR|XP_007409903.1 MLAR|XP_007410825.1 MLAR|XP_007411094.1 MLAR|XP_007412027.1 MLAR|XP_007413616.1 MLAR|XP_007413840.1 MLAR|XP_007413857.1 MLAR|XP_007414201.1 MLAR|XP_007414215.1 MLAR|XP_007414597.1 MLAR|XP_007415329.1 MLAR|XP_007415966.1 MLAR|XP_007417528.1 MLAR|XP_007418310.1 MLAR|XP_007418464.1 MLAR|XP_007419308.1 MLAR|XP_007419548.1 MLAR|XP_007419607.1 MLAR|XP_007419608.1 MLAR|XP_007419712.1

>Orthogroup432: MLAR|XP_007404393.1 MLAR|XP_007405170.1 MLAR|XP_007405352.1 MLAR|XP_007406118.1 MLAR|XP_007406196.1 MLAR|XP_007406288.1 MLAR|XP_007407018.1 MLAR|XP_007408316.1 MLAR|XP_007409144.1 MLAR|XP_007410689.1 MLAR|XP_007410696.1 MLAR|XP_007411387.1 MLAR|XP_007413120.1 MLAR|XP_007413382.1 MLAR|XP_007413835.1 MLAR|XP_007414265.1 MLAR|XP_007414435.1 MLAR|XP_007415504.1 MLAR|XP_007416082.1 MLAR|XP_007416545.1 MLAR|XP_007416842.1 MLAR|XP_007417769.1 MLAR|XP_007417876.1 MLAR|XP_007418107.1 MLAR|XP_007418957.1 MLAR|XP_007419167.1 MLAR|XP_007419324.1 MLAR|XP_007419645.1 MLAR|XP_007419785.1

>Orthogroup433: MLAR|XP_007405320.1 MLAR|XP_007405532.1 MLAR|XP_007405837.1 MLAR|XP_007406006.1 MLAR|XP_007407280.1 MLAR|XP_007408464.1 MLAR|XP_007408780.1 MLAR|XP_007409307.1 MLAR|XP_007409443.1 MLAR|XP_007410030.1 MLAR|XP_007410273.1 MLAR|XP_007410549.1 MLAR|XP_007410715.1 MLAR|XP_007411916.1 MLAR|XP_007413737.1 MLAR|XP_007414177.1 MLAR|XP_007415611.1 MLAR|XP_007415664.1 MLAR|XP_007416680.1 MLAR|XP_007417160.1 MLAR|XP_007417393.1 MLAR|XP_007418176.1 MLAR|XP_007418435.1 MLAR|XP_007418835.1 MLAR|XP_007418903.1 MLAR|XP_007418981.1 MLAR|XP_007418994.1 MLAR|XP_007419151.1 MLAR|XP_007419599.1

>Orthogroup434: ANID|CBF69490.1 ANID|CBF74922.1 ANID|CBF80489.1 BCIN|XP_001546353.1 BCIN|XP_024549263.1 CFRU|XP_031878812.1 CFRU|XP_031881051.1 CFRU|XP_031884145.1 CFRU|XP_031888075.1 CGLO|KAF3800005.1 CGLO|KAF3802028.1 CGLO|KAF3802613.1 CGLO|KAF3809844.1 CHIG|XP_018153032.1 CHIG|XP_018154146.1 CVIN|KAF4910499.1 CVIN|KAF4911599.1 CVIN|KAF4926860.1 CVIN|KAF4930700.1 CVYL|A01770 CVYL|A01906 CVYL|A03361 CVYL|A10221 FGRM|XP_011325873.1 MGRA|XP_003851362.1 MORY|QBZ59839.1 NCRA|XP_958769.1 SSCL|APA05400.1

>Orthogroup435: ANID|CBF69930.1 ANID|CBF87504.1 BCIN|XP_024546910.1 CFRU|XP_031880983.1 CFRU|XP_031884212.1 CFRU|XP_031888191.1 CFRU|XP_031893328.1 CGLO|KAF3798399.1 CGLO|KAF3800002.1 CGLO|KAF3800453.1 CGLO|KAF3802092.1 CHIG|XP_018152722.1 CHIG|XP_018154241.1 CHIG|XP_018161389.1 CHIG|XP_018163417.1 CVIN|KAF4902811.1 CVIN|KAF4917966.1 CVIN|KAF4922808.1 CVIN|KAF4926858.1 CVYL|A01838 CVYL|A03298 CVYL|A08214 CVYL|A10219 FGRM|XP_011315770.1 MGRA|XP_003850945.1 MORY|QBZ61152.1 MORY|QBZ64912.1 NCRA|XP_959589.1

>Orthogroup436: ANID|CBF70712.1 ANID|CBF83101.1 ANID|CBF84710.1 BCIN|XP_001556692.1 BCIN|XP_024549715.1 BGRA|VDB83944.1 CFRU|XP_031881319.1 CFRU|XP_031885020.1 CFRU|XP_031888501.1 CGLO|KAF3800328.1 CGLO|KAF3802451.1 CGLO|KAF3803541.1 CHIG|XP_018162925.1 CHIG|XP_018164082.1 CHIG|XP_018164083.1 CVIN|KAF4913918.1 CVIN|KAF4922247.1 CVIN|KAF4927787.1 CVYL|A02042 CVYL|A06896 CVYL|A14473 FGRM|XP_011316161.1 MGRA|XP_003857591.1 MGRA|XP_003857608.1 MORY|QBZ56428.1 MORY|QBZ61225.1 SSCL|APA05459.1 SSCL|APA14826.1

>Orthogroup437: ANID|CBF71157.1 ANID|CBF85251.1 BCIN|XP_001556812.2 BCIN|XP_024553562.1 CFRU|XP_031878550.1 CFRU|XP_031887977.1 CFRU|XP_031888971.1 CGLO|KAF3797609.1 CGLO|KAF3798496.1 CGLO|KAF3799210.1 CHIG|XP_018159829.1 CHIG|XP_018161221.1 CHIG|XP_018163471.1 CVIN|KAF4909308.1 CVIN|KAF4914250.1 CVIN|KAF4917482.1 CVYL|A03384 CVYL|A10105 CVYL|A11167 FGRM|XP_011318628.1 FGRM|XP_011323390.1 FGRM|XP_011328822.1 MGRA|XP_003852150.1 MGRA|XP_003854801.1 MORY|QBZ53395.1 MORY|QBZ55137.1 NCRA|XP_956354.1 SSCL|APA14590.1

>Orthogroup438: ANID|CBF71735.1 ANID|CBF80146.1 ANID|CBF82423.1 ANID|CBF90105.1 BCIN|XP_001557620.1 BCIN|XP_024547966.1 BCIN|XP_024551162.1 CFRU|XP_031883004.1 CFRU|XP_031892748.1 CGLO|KAF3808230.1 CGLO|KAF3809073.1 CHIG|XP_018155910.1 CHIG|XP_018156541.1 CHIG|XP_018163155.1 CVIN|KAF4909563.1 CVIN|KAF4919735.1 CVYL|A00571 CVYL|A01638 FGRM|XP_011327507.1 MGRA|XP_003848335.1 MGRA|XP_003849490.1 MGRA|XP_003855429.1 MORY|QBZ53582.1 MORY|QBZ57331.1 NCRA|XP_011395293.1 NCRA|XP_959252.1 SSCL|APA08378.1 SSCL|APA12304.1

>Orthogroup439: ANID|CBF71745.1 ANID|CBF82367.1 ANID|CBF82391.1 ANID|CBF83637.1 ANID|CBF86914.1 BCIN|XP_001549083.2 BCIN|XP_001552524.1 BCIN|XP_024546566.1 BGRA|VDB94898.1 CFRU|XP_031878121.1 CFRU|XP_031888831.1 CGLO|KAF3799353.1 CGLO|KAF3801816.1 CHIG|XP_018159328.1 CHIG|XP_018159938.1 CVIN|KAF4910731.1 CVIN|KAF4917004.1 CVYL|A04530 CVYL|A12891 FGRM|XP_011317569.1 FGRM|XP_011325458.1 MGRA|XP_003855624.1 MORY|QBZ61901.1 NCRA|XP_959563.1 NCRA|XP_959582.3 SSCL|APA06214.1 SSCL|APA06784.1 SSCL|APA10791.1

>Orthogroup440: ANID|CBF73401.1 ANID|CBF87393.1 CFRU|XP_031879141.1 CFRU|XP_031880392.1 CGLO|KAF3807081.1 CGLO|KAF3807262.1 CHIG|XP_018156942.1 CHIG|XP_018159735.1 CHIG|XP_018159749.1 CHIG|XP_018159750.1 CVIN|KAF4919047.1 CVIN|KAF4928188.1 CVYL|A06375 CVYL|A10744 FGRM|XP_011320678.1 FGRM|XP_011320914.1 FGRM|XP_011328643.1 MGRA|XP_003848229.1 MGRA|XP_003850199.1 MGRA|XP_003852967.1 MGRA|XP_003856667.1 MORY|QBZ55125.1 MORY|QBZ60200.1 MORY|QBZ64019.1 MORY|QBZ64104.1 MORY|QBZ65202.1 NCRA|XP_961650.3 NCRA|XP_963903.3

>Orthogroup441: ANID|CBF73423.1 BCIN|XP_001552898.2 BCIN|XP_024552915.1 CFRU|XP_031882404.1 CGLO|KAF3807525.1 CHIG|XP_018156412.1 CVIN|KAF4928936.1 CVYL|A02959 FGRM|XP_011324350.1 MGRA|XP_003853631.1 MGRA|XP_003854186.1 MGRA|XP_003854438.1 MORY|QBZ54782.1 MORY|QBZ54861.1 MORY|QBZ55073.1 MORY|QBZ57876.1 MORY|QBZ60225.1 MORY|QBZ63015.1 MORY|QBZ63321.1 NCRA|XP_001728240.1 NCRA|XP_958257.1 NCRA|XP_959767.2 NCRA|XP_960260.2 NCRA|XP_961674.3 NCRA|XP_962587.3 NCRA|XP_963349.2 SSCL|APA06159.1 SSCL|APA07530.1

>Orthogroup442: ANID|CBF73433.1 ANID|CBF88174.1 BCIN|XP_001548465.2 BCIN|XP_024547848.1 BCIN|XP_024551012.1 BGRA|VCU40920.1 CFRU|XP_031881040.1 CFRU|XP_031883874.1 CFRU|XP_031889414.1 CGLO|KAF3808721.1 CHIG|XP_018154167.1 CHIG|XP_018155549.1 CHIG|XP_018158868.1 CHIG|XP_018161484.1 CHIG|XP_018163420.1 CVIN|KAF4927398.1 CVIN|KAF4929568.1 CVIN|KAF4930710.1 CVYL|A00939 CVYL|A01750 CVYL|A06458 FGRM|XP_011319156.1 MGRA|XP_003849080.1 MLAR|XP_007412504.1 MORY|QBZ63094.1 NCRA|XP_958376.2 SSCL|APA11923.1 SSCL|APA13813.1

>Orthogroup443: ANID|CBF73940.1 ANID|CBF84836.1 BCIN|XP_001549595.1 BCIN|XP_001557811.1 BCIN|XP_024546392.1 BGRA|VDB87924.1 CFRU|XP_031886042.1 CFRU|XP_031890876.1 CGLO|KAF3801469.1 CGLO|KAF3806591.1 CHIG|XP_018151899.1 CHIG|XP_018157631.1 CVIN|KAF4903845.1 CVIN|KAF4920496.1 CVYL|A09277 CVYL|A12655 FGRM|XP_011320577.1 FGRM|XP_011325498.1 FGRM|XP_011327754.1 MGRA|XP_003854045.1 MGRA|XP_003855514.1 MLAR|XP_007404188.1 MLAR|XP_007414142.1 MORY|QBZ61165.1 NCRA|XP_958355.1 SSCL|APA05311.1 SSCL|APA07209.1 SSCL|APA12138.1

>Orthogroup444: ANID|CBF74138.1 ANID|CBF76607.1 ANID|CBF89735.1 BCIN|XP_001554426.1 BCIN|XP_024552363.1 BGRA|VCU39176.1 BGRA|VDB93920.1 CFRU|XP_031881775.1 CFRU|XP_031886710.1 CGLO|KAF3800644.1 CGLO|KAF3804046.1 CHIG|XP_018152037.1 CHIG|XP_018162720.1 CVIN|KAF4922011.1 CVIN|KAF4927636.1 CVYL|A07003 CVYL|A13072 FGRM|XP_011317228.1 FGRM|XP_011320444.1 MGRA|XP_003852209.1 MLAR|XP_007412017.1 MLAR|XP_007414769.1 MORY|QBZ57310.1 MORY|QBZ59310.1 NCRA|XP_956886.1 NCRA|XP_964241.3 SSCL|APA12617.1 SSCL|APA13232.1

>Orthogroup445: ANID|CBF74142.1 ANID|CBF80266.1 ANID|CBF83058.1 ANID|CBF86720.1 BCIN|XP_024547678.1 CFRU|XP_031878020.1 CFRU|XP_031881196.1 CFRU|XP_031881200.1 CFRU|XP_031881277.1 CFRU|XP_031892549.1 CFRU|XP_031892627.1 CGLO|KAF3802455.1 CGLO|KAF3809121.1 CHIG|XP_018154755.1 CVIN|KAF4916884.1 CVIN|KAF4922088.1 CVYL|A01305 CVYL|A02046 CVYL|A04005 FGRM|XP_011319501.1 FGRM|XP_011325299.1 FGRM|XP_011327547.1 MGRA|XP_003852738.1 MGRA|XP_003855494.1 MORY|QBZ55306.1 NCRA|XP_011393052.1 NCRA|XP_958464.3 NCRA|XP_962972.1

>Orthogroup446: ANID|CBF74206.1 ANID|CBF76034.1 ANID|CBF87169.1 BCIN|XP_024549606.1 CFRU|XP_031878266.1 CFRU|XP_031878784.1 CFRU|XP_031884289.1 CGLO|KAF3798029.1 CGLO|KAF3799970.1 CHIG|XP_018153079.1 CHIG|XP_018156565.1 CHIG|XP_018158086.1 CVIN|KAF4911598.1 CVIN|KAF4915609.1 CVIN|KAF4926930.1 CVYL|A01908 CVYL|A08328 CVYL|A10187 FGRM|XP_011317993.1 FGRM|XP_011323852.1 FGRM|XP_011328584.1 MGRA|XP_003851381.1 MGRA|XP_003855003.1 MORY|QBZ57370.1 MORY|QBZ62715.1 MORY|QBZ64354.1 MORY|QBZ65564.1 NCRA|XP_958581.1

>Orthogroup447: ANID|CBF74875.1 BCIN|XP_001550695.1 BGRA|VDB93306.1 CFRU|XP_031882222.1 CFRU|XP_031883711.1 CFRU|XP_031890424.1 CGLO|KAF3804351.1 CGLO|KAF3805273.1 CGLO|KAF3810864.1 CHIG|XP_018154961.1 CHIG|XP_018158799.1 CVIN|KAF4897553.1 CVIN|KAF4901287.1 CVIN|KAF4918358.1 CVYL|A02282 CVYL|A04043 CVYL|A13924 FGRM|XP_011316666.1 FGRM|XP_011320673.1 FGRM|XP_011322656.1 MGRA|XP_003849776.1 MGRA|XP_003855930.1 MLAR|XP_007412073.1 MORY|QBZ56153.1 MORY|QBZ60400.1 MORY|QBZ65348.1 NCRA|XP_964967.3 SSCL|APA09312.1

>Orthogroup448: ANID|CBF75372.1 ANID|CBF89542.1 BCIN|XP_024549171.1 BCIN|XP_024551387.1 BGRA|VCU41132.1 BGRA|VDB92531.1 CFRU|XP_031878429.1 CFRU|XP_031889997.1 CGLO|KAF3802647.1 CGLO|KAF3802914.1 CHIG|XP_018158517.1 CHIG|XP_018162514.1 CVIN|KAF4925567.1 CVIN|KAF4932007.1 CVYL|A05101 CVYL|A07148 FGRM|XP_011316575.1 FGRM|XP_011326968.1 MGRA|XP_003857455.1 MGRA|XP_003857563.1 MLAR|XP_007406566.1 MLAR|XP_007415752.1 MORY|QBZ55675.1 MORY|QBZ63220.1 NCRA|XP_011393180.1 NCRA|XP_956154.2 SSCL|APA08768.1 SSCL|APA10616.1

>Orthogroup449: ANID|CBF75440.1 ANID|CBF82340.1 ANID|CBF82810.1 BCIN|XP_001555678.1 BGRA|VDB92571.1 CFRU|XP_031881370.1 CFRU|XP_031884846.1 CFRU|XP_031892915.1 CGLO|KAF3805823.1 CGLO|KAF3809552.1 CGLO|KAF3812159.1 CHIG|XP_018152626.1 CHIG|XP_018153410.1 CHIG|XP_018163709.1 CVIN|KAF4918547.1 CVIN|KAF4918747.1 CVIN|KAF4920897.1 CVYL|A00232 CVYL|A05999 CVYL|A08470 FGRM|XP_011322857.1 MLAR|XP_007406532.1 MLAR|XP_007411844.1 MLAR|XP_007412521.1 MORY|QBZ54118.1 MORY|QBZ54822.1 MORY|QBZ61587.1 SSCL|APA10104.1

>Orthogroup450: ANID|CBF77158.1 ANID|CBF85192.1 ANID|CBF87418.1 BCIN|XP_001547304.1 BCIN|XP_001549390.2 BCIN|XP_001559720.1 CFRU|XP_031879368.1 CFRU|XP_031884734.1 CFRU|XP_031890293.1 CGLO|KAF3802920.1 CGLO|KAF3808595.1 CGLO|KAF3812137.1 CHIG|XP_018153790.1 CHIG|XP_018158522.1 CHIG|XP_018161833.1 CVIN|KAF4910008.1 CVIN|KAF4925564.1 CVIN|KAF4926202.1 CVYL|A00261 CVYL|A05095 CVYL|A11548 FGRM|XP_011320939.1 FGRM|XP_011321745.1 FGRM|XP_011328373.1 MGRA|XP_003856985.1 MORY|QBZ63211.1 NCRA|XP_959079.1 SSCL|APA09460.1

>Orthogroup451: ANID|CBF77250.1 ANID|CBF81263.1 BCIN|XP_024548302.1 BCIN|XP_024549210.1 BGRA|VCU40721.1 CFRU|XP_031880459.1 CFRU|XP_031882396.1 CGLO|KAF3807270.1 CGLO|KAF3807606.1 CHIG|XP_018156329.1 CHIG|XP_018156944.1 CVIN|KAF4928133.1 CVIN|KAF4928942.1 CVYL|A02876 CVYL|A10753 FGRM|XP_011318996.1 FGRM|XP_011320000.1 MGRA|XP_003847554.1 MGRA|XP_003848161.1 MLAR|XP_007403756.1 MLAR|XP_007405398.1 MLAR|XP_007413259.1 MORY|QBZ56924.1 MORY|QBZ65912.1 NCRA|XP_011392815.1 NCRA|XP_011394897.1 SSCL|APA12550.1 SSCL|APA15741.1

>Orthogroup452: ANID|CBF78782.1 ANID|CBF85258.1 ANID|CBF87082.1 BCIN|XP_024549578.1 BGRA|VDB92479.1 CFRU|XP_031878480.1 CFRU|XP_031883035.1 CFRU|XP_031883903.1 CGLO|KAF3799792.1 CGLO|KAF3805087.1 CGLO|KAF3808179.1 CHIG|XP_018153883.1 CHIG|XP_018163231.1 CHIG|XP_018163426.1 CVIN|KAF4912460.1 CVIN|KAF4924298.1 CVIN|KAF4927446.1 CVYL|A00534 CVYL|A04801 CVYL|A06471 FGRM|XP_011318016.1 FGRM|XP_011318026.1 FGRM|XP_011327639.1 MORY|QBZ65565.1 NCRA|XP_001728196.2 NCRA|XP_958547.1 NCRA|XP_959390.1 SSCL|APA13966.1

>Orthogroup453: ANID|CBF79483.1 BCIN|XP_001551204.1 BCIN|XP_024552393.1 BCIN|XP_024553568.1 BGRA|VCU38790.1 CFRU|XP_031878638.1 CFRU|XP_031883052.1 CFRU|XP_031884559.1 CGLO|KAF3798511.1 CGLO|KAF3800727.1 CGLO|KAF3810356.1 CHIG|XP_018161189.1 CHIG|XP_018163278.1 CVIN|KAF4897445.1 CVIN|KAF4917495.1 CVIN|KAF4919614.1 CVYL|A00477 CVYL|A03814 CVYL|A05754 FGRM|XP_011320073.1 FGRM|XP_011328773.1 MGRA|XP_003853639.1 MLAR|XP_007417675.1 MORY|QBZ56519.1 MORY|QBZ61516.1 NCRA|XP_011394184.1 SSCL|APA13229.1 SSCL|APA13310.1

>Orthogroup454: ANID|CBF79566.1 BCIN|XP_024548564.1 CFRU|XP_031881420.1 CFRU|XP_031881454.1 CFRU|XP_031885111.1 CFRU|XP_031887979.1 CGLO|KAF3800946.1 CGLO|KAF3802201.1 CGLO|KAF3805726.1 CGLO|KAF3807754.1 CHIG|XP_018153211.1 CHIG|XP_018156543.1 CHIG|XP_018163022.1 CVIN|KAF4912788.1 CVIN|KAF4920517.1 CVIN|KAF4926584.1 CVYL|A00844 CVYL|A05959 CVYL|A09817 FGRM|XP_011320694.1 FGRM|XP_011321755.1 MGRA|XP_003848485.1 MGRA|XP_003851106.1 MGRA|XP_003851922.1 MORY|QBZ66372.1 NCRA|XP_956089.2 NCRA|XP_959388.1 SSCL|APA10041.1

>Orthogroup455: ANID|CBF80626.1 CFRU|XP_031878145.1 CFRU|XP_031878711.1 CFRU|XP_031880124.1 CFRU|XP_031884561.1 CFRU|XP_031887970.1 CFRU|XP_031889459.1 CFRU|XP_031890816.1 CFRU|XP_031892172.1 CGLO|KAF3797612.1 CGLO|KAF3799701.1 CGLO|KAF3801320.1 CGLO|KAF3801844.1 CGLO|KAF3808881.1 CHIG|XP_018158309.1 CVIN|KAF4914252.1 CVIN|KAF4914527.1 CVIN|KAF4919984.1 CVIN|KAF4923578.1 CVYL|A01201 CVYL|A03381 CVYL|A05862 CVYL|A08974 CVYL|A12863 FGRM|XP_011321961.1 MGRA|XP_003851327.1 MGRA|XP_003855536.1 MORY|QBZ58847.1

>Orthogroup456: ANID|CBF81283.1 BCIN|XP_001547711.2 BCIN|XP_001548969.1 CFRU|XP_031879900.1 CFRU|XP_031881772.1 CFRU|XP_031885876.1 CFRU|XP_031891839.1 CGLO|KAF3798964.1 CGLO|KAF3801156.1 CGLO|KAF3809985.1 CHIG|XP_018151879.1 CHIG|XP_018152849.1 CHIG|XP_018164874.1 CVIN|KAF4905019.1 CVIN|KAF4911049.1 CVIN|KAF4921975.1 CVIN|KAF4923487.1 CVYL|A02585 CVYL|A05690 CVYL|A06619 CVYL|A07881 FGRM|XP_011322232.1 FGRM|XP_011323536.1 MGRA|XP_003850748.1 MORY|QBZ53347.1 NCRA|XP_959640.1 SSCL|APA07031.1 SSCL|APA13291.1

>Orthogroup457: ANID|CBF81374.1 ANID|CBF89657.1 BCIN|XP_001556507.1 BCIN|XP_024546549.1 BGRA|VDB95042.1 CFRU|XP_031880879.1 CFRU|XP_031888549.1 CGLO|KAF3803553.1 CGLO|KAF3806892.1 CHIG|XP_018159573.1 CHIG|XP_018164138.1 CVIN|KAF4904504.1 CVIN|KAF4922280.1 CVYL|A02535 CVYL|A14486 FGRM|XP_011316151.1 FGRM|XP_011320451.1 MGRA|XP_003850594.1 MGRA|XP_003854950.1 MLAR|XP_007408195.1 MLAR|XP_007411260.1 MLAR|XP_007411398.1 MORY|QBZ61868.1 MORY|QBZ63821.1 NCRA|XP_960510.1 NCRA|XP_963757.2 SSCL|APA06513.1 SSCL|APA06676.1

>Orthogroup458: ANID|CBF82107.1 BCIN|XP_024547086.1 CFRU|XP_031885061.1 CFRU|XP_031885150.1 CFRU|XP_031885163.1 CFRU|XP_031885273.1 CGLO|KAF3800305.1 CHIG|XP_018162794.1 CVIN|KAF4927621.1 CVIN|KAF4927622.1 CVIN|KAF4927623.1 CVIN|KAF4927624.1 CVYL|A06922 CVYL|A06923 CVYL|A06924 CVYL|A06925 FGRM|XP_011320892.1 FGRM|XP_011321604.1 FGRM|XP_011321605.1 FGRM|XP_011321606.1 NCRA|XP_958106.3 NCRA|XP_958110.1 NCRA|XP_958111.1 NCRA|XP_958112.1 NCRA|XP_958231.1 NCRA|XP_958232.2 NCRA|XP_959117.1 SSCL|APA05238.1

>Orthogroup459: ANID|CBF83079.1 ANID|CBF87832.1 BCIN|XP_001547173.1 BCIN|XP_001551018.1 BCIN|XP_001557527.1 CFRU|XP_031880120.1 CFRU|XP_031881201.1 CFRU|XP_031883895.1 CFRU|XP_031889350.1 CGLO|KAF3808806.1 CGLO|KAF3810970.1 CHIG|XP_018152339.1 CHIG|XP_018155709.1 CVIN|KAF4907652.1 CVIN|KAF4908016.1 CVIN|KAF4921839.1 CVYL|A00858 CVYL|A02180 FGRM|XP_011318950.1 FGRM|XP_011319258.1 MGRA|XP_003847998.1 MORY|QBZ53753.1 MORY|QBZ58070.1 NCRA|XP_960810.1 SSCL|APA08914.1 SSCL|APA08915.1 SSCL|APA12366.1 SSCL|APA14296.1

>Orthogroup460: ANID|CBF83091.1 BCIN|XP_024546271.1 BCIN|XP_024549781.1 BCIN|XP_024550674.1 CFRU|XP_031879247.1 CFRU|XP_031880476.1 CFRU|XP_031881678.1 CGLO|KAF3807100.1 CGLO|KAF3807423.1 CGLO|KAF3811565.1 CHIG|XP_018163322.1 CVIN|KAF4906638.1 CVIN|KAF4919040.1 CVIN|KAF4926789.1 CVYL|A04777 CVYL|A10925 FGRM|XP_011317656.1 MGRA|XP_003849057.1 MGRA|XP_003849180.1 MGRA|XP_003850578.1 MLAR|XP_007413403.1 MLAR|XP_007413472.1 MLAR|XP_007417481.1 MORY|QBZ65522.1 NCRA|XP_960040.1 SSCL|APA05730.1 SSCL|APA09444.1 SSCL|APA16004.1

>Orthogroup461: ANID|CBF86075.1 BCIN|XP_001552123.1 BCIN|XP_001556588.1 CFRU|XP_031890538.1 CFRU|XP_031891034.1 CFRU|XP_031892985.1 CGLO|KAF3801598.1 CGLO|KAF3804896.1 CGLO|KAF3808012.1 CHIG|XP_018151350.1 CHIG|XP_018152368.1 CHIG|XP_018164365.1 CVIN|KAF4909777.1 CVIN|KAF4922725.1 CVYL|A12163 CVYL|A12537 FGRM|XP_011317740.1 MGRA|XP_003853858.1 MLAR|XP_007413755.1 MLAR|XP_007413756.1 MLAR|XP_007413757.1 MLAR|XP_007415395.1 MORY|QBZ54210.1 MORY|QBZ56523.1 NCRA|XP_958218.3 NCRA|XP_961276.2 SSCL|APA05277.1 SSCL|APA06261.1

>Orthogroup462: ANID|CBF86432.1 BCIN|XP_024549285.1 BCIN|XP_024549379.1 BGRA|VCU38889.1 CFRU|XP_031875598.1 CFRU|XP_031878781.1 CFRU|XP_031881498.1 CFRU|XP_031890045.1 CGLO|KAF3798253.1 CGLO|KAF3802587.1 CGLO|KAF3804757.1 CGLO|KAF3805786.1 CHIG|XP_018157649.1 CHIG|XP_018163686.1 CVIN|KAF4916890.1 CVIN|KAF4917614.1 CVIN|KAF4926178.1 CVIN|KAF4926570.1 CVYL|A01875 CVYL|A06034 CVYL|A09236 CVYL|A12321 FGRM|XP_011322722.1 MLAR|XP_007403558.1 MLAR|XP_007406664.1 MORY|QBZ56271.1 NCRA|XP_962627.1 SSCL|APA08956.1

>Orthogroup463: ANID|CBF86464.1 BCIN|XP_001559555.2 BCIN|XP_001560503.1 BCIN|XP_024550008.1 BCIN|XP_024552035.1 BGRA|VDB96339.1 CFRU|XP_031879354.1 CFRU|XP_031886312.1 CFRU|XP_031888828.1 CGLO|KAF3799355.1 CGLO|KAF3806201.1 CGLO|KAF3808558.1 CHIG|XP_018161924.1 CHIG|XP_018162190.1 CVIN|KAF4906496.1 CVIN|KAF4910726.1 CVIN|KAF4931518.1 CVYL|A11588 CVYL|A11787 FGRM|XP_011321470.1 FGRM|XP_011325700.1 MGRA|XP_003852687.1 MORY|QBZ55513.1 MORY|QBZ65230.1 NCRA|XP_011394352.1 SSCL|APA05340.1 SSCL|APA07748.1 SSCL|APA12799.1

>Orthogroup464: ANID|CBF86652.1 ANID|CBF87526.1 BCIN|XP_024551320.1 CFRU|XP_031879217.1 CFRU|XP_031879717.1 CFRU|XP_031893503.1 CGLO|KAF3807018.1 CHIG|XP_018152780.1 CHIG|XP_018159684.1 CVIN|KAF4920262.1 CVIN|KAF4920645.1 CVIN|KAF4930166.1 CVYL|A04870 CVYL|A06310 CVYL|A08154 MGRA|XP_003848899.1 MGRA|XP_003853740.1 MLAR|XP_007404780.1 MLAR|XP_007410456.1 MLAR|XP_007411853.1 MLAR|XP_007411922.1 MLAR|XP_007412283.1 MLAR|XP_007415625.1 MLAR|XP_007416193.1 MLAR|XP_007416194.1 MORY|QBZ61670.1 NCRA|XP_960021.3 SSCL|APA10552.1

>Orthogroup465: ANID|CBF87061.1 BCIN|XP_001552507.1 BCIN|XP_024550942.1 CFRU|XP_031876815.1 CFRU|XP_031878701.1 CFRU|XP_031881438.1 CGLO|KAF3797577.1 CGLO|KAF3797978.1 CGLO|KAF3800919.1 CHIG|XP_018153191.1 CVIN|KAF4914947.1 CVIN|KAF4914948.1 CVIN|KAF4920880.1 CVIN|KAF4923586.1 CVYL|A05984 CVYL|A08375 CVYL|A08376 CVYL|A08967 FGRM|XP_011326063.1 MGRA|XP_003849037.1 MGRA|XP_003853287.1 MLAR|XP_007407432.1 MLAR|XP_007407650.1 MLAR|XP_007410875.1 MORY|QBZ58359.1 MORY|QBZ62022.1 NCRA|XP_959465.2 SSCL|APA10771.1

>Orthogroup466: BCIN|XP_001548428.2 BCIN|XP_001551871.1 BCIN|XP_001552940.1 BGRA|VDB88882.1 BGRA|VDB91038.1 CFRU|XP_031876894.1 CFRU|XP_031877858.1 CFRU|XP_031884876.1 CGLO|KAF3799837.1 CGLO|KAF3806616.1 CGLO|KAF3812134.1 CHIG|XP_018150729.1 CHIG|XP_018152659.1 CHIG|XP_018156614.1 CHIG|XP_018156662.1 CHIG|XP_018161326.1 CHIG|XP_018164240.1 CVIN|KAF4909999.1 CVIN|KAF4918895.1 CVYL|A00264 CVYL|A09677 CVYL|A12326 MORY|QBZ55410.1 MORY|QBZ61992.1 NCRA|XP_958517.2 SSCL|APA05427.1 SSCL|APA09286.1 SSCL|APA15224.1

>Orthogroup467: BCIN|XP_001552971.1 CFRU|XP_031875916.1 CFRU|XP_031879042.1 CFRU|XP_031880088.1 CFRU|XP_031889939.1 CGLO|KAF3797740.1 CGLO|KAF3798808.1 CGLO|KAF3802842.1 CGLO|KAF3807815.1 CHIG|XP_018151200.1 CHIG|XP_018158443.1 CHIG|XP_018162981.1 CVIN|KAF4910481.1 CVIN|KAF4917538.1 CVIN|KAF4919590.1 CVIN|KAF4921528.1 CVYL|A00782 CVYL|A05170 CVYL|A09122 CVYL|A11413 FGRM|XP_011322023.1 FGRM|XP_011326002.1 MGRA|XP_003851980.1 MORY|QBZ61917.1 MORY|QBZ64160.1 MORY|QBZ65192.1 NCRA|XP_959049.1 SSCL|APA14000.1

>Orthogroup468: BCIN|XP_001548252.1 BCIN|XP_001548254.1 BCIN|XP_001551086.1 BCIN|XP_024548661.1 BCIN|XP_024548664.1 CFRU|XP_031886142.1 CFRU|XP_031888796.1 CGLO|KAF3798627.1 CGLO|KAF3799380.1 CGLO|KAF3811972.1 CHIG|XP_018151781.1 CHIG|XP_018151846.1 CHIG|XP_018151848.1 CHIG|XP_018153994.1 CHIG|XP_018153999.1 CHIG|XP_018156506.1 CHIG|XP_018156880.1 CHIG|XP_018156882.1 CHIG|XP_018159951.1 CHIG|XP_018164213.1 CVIN|KAF4906801.1 CVIN|KAF4912455.1 CVYL|A00605 CVYL|A04774 CVYL|A04820 MGRA|XP_003851926.1 MORY|QBZ65459.1 SSCL|APA15022.1

>Orthogroup469: BCIN|XP_001560456.1 CFRU|XP_031887293.1 CFRU|XP_031889557.1 CFRU|XP_031891740.1 CGLO|KAF3800160.1 CGLO|KAF3809321.1 CGLO|KAF3810460.1 CHIG|XP_018156159.1 CHIG|XP_018159169.1 CHIG|XP_018165096.1 CVIN|KAF4924176.1 CVIN|KAF4924609.1 CVIN|KAF4925831.1 CVYL|A01521 CVYL|A06857 CVYL|A14034 FGRM|XP_011315577.1 FGRM|XP_011327288.1 MGRA|XP_003854036.1 MLAR|XP_007408693.1 MLAR|XP_007408696.1 MLAR|XP_007413658.1 MLAR|XP_007418362.1 MORY|QBZ59204.1 MORY|QBZ60639.1 MORY|QBZ61859.1 NCRA|XP_958001.1 SSCL|APA15708.1
[truncated: 2,741,771 more chars]
