# Supplementary material for: Histological and transcriptomic insights into the interaction between grapevine and Colletotrichum viniferum
Source: Front Plant Sci. 2024 Aug 16;15:1446288. doi: 10.3389/fpls.2024.1446288 (PMC11362058; doi:10.3389/fpls.2024.1446288)
Supplement: Supplementary file 2 [file DataSheet2.docx]

***Supplementary Material***

**Supplementary 2** 1155 single-copy orthologous families of 14 fungi genome

>Orthogroup2147: ANID|CBF69583.1 BCIN|XP_024552156.1 BGRA|VDB92763.1 CFRU|XP_031877532.1 CGLO|KAF3798432.1 CHIG|XP_018161250.1 CVIN|KAF4920000.1 CVYL|A11205 FGRM|XP_011321438.1 MGRA|XP_003849521.1 MLAR|XP_007411397.1 MORY|QBZ56746.1 NCRA|XP_961965.2 SSCL|APA13153.1

>Orthogroup2148: ANID|CBF69598.1 BCIN|XP_024552426.1 BGRA|VDB93429.1 CFRU|XP_031884335.1 CGLO|KAF3806467.1 CHIG|XP_018162363.1 CVIN|KAF4908638.1 CVYL|A12068 FGRM|XP_011323922.1 MGRA|XP_003854396.1 MLAR|XP_007405558.1 MORY|QBZ55498.1 NCRA|XP_958270.2 SSCL|APA14960.1

>Orthogroup2150: ANID|CBF69629.1 BCIN|XP_001549813.2 BGRA|VDB93586.1 CFRU|XP_031883217.1 CGLO|KAF3800744.1 CHIG|XP_018163293.1 CVIN|KAF4919649.1 CVYL|A00460 FGRM|XP_011326201.1 MGRA|XP_003854082.1 MLAR|XP_007418371.1 MORY|QBZ58246.1 NCRA|XP_962825.2 SSCL|APA14457.1

>Orthogroup2151: ANID|CBF69644.1 BCIN|XP_024552415.1 BGRA|VDB87884.1 CFRU|XP_031886499.1 CGLO|KAF3806217.1 CHIG|XP_018162052.1 CVIN|KAF4931466.1 CVYL|A11806 FGRM|XP_011326624.1 MGRA|XP_003854541.1 MLAR|XP_007408550.1 MORY|QBZ61345.1 NCRA|XP_962863.3 SSCL|APA14949.1

>Orthogroup2152: ANID|CBF69649.1 BCIN|XP_001547514.1 BGRA|VDB93399.1 CFRU|XP_031877206.1 CGLO|KAF3806419.1 CHIG|XP_018162304.1 CVIN|KAF4918069.1 CVYL|A12013 FGRM|XP_011324131.1 MGRA|XP_003854178.1 MLAR|XP_007410545.1 MORY|QBZ55764.1 NCRA|XP_011393824.1 SSCL|APA14963.1

>Orthogroup2153: ANID|CBF69655.1 BCIN|XP_024553716.1 BGRA|VDB93359.1 CFRU|XP_031893014.1 CGLO|KAF3808417.1 CHIG|XP_018152495.1 CVIN|KAF4924991.1 CVYL|A09576 FGRM|XP_011317928.1 MGRA|XP_003854073.1 MLAR|XP_007418346.1 MORY|QBZ54854.1 NCRA|XP_958281.1 SSCL|APA15110.1

>Orthogroup2155: ANID|CBF69674.1 BCIN|XP_024552020.1 BGRA|VDB89684.1 CFRU|XP_031876717.1 CGLO|KAF3798674.1 CHIG|XP_018157480.1 CVIN|KAF4918469.1 CVYL|A09413 FGRM|XP_011317819.1 MGRA|XP_003849071.1 MLAR|XP_007412700.1 MORY|QBZ58956.1 NCRA|XP_011394408.1 SSCL|APA12784.1

>Orthogroup2156: ANID|CBF69680.1 BCIN|XP_024552023.1 BGRA|VDB89690.1 CFRU|XP_031876707.1 CGLO|KAF3798676.1 CHIG|XP_018157479.1 CVIN|KAF4918491.1 CVYL|A09415 FGRM|XP_011327895.1 MGRA|XP_003848872.1 MLAR|XP_007413258.1 MORY|QBZ58958.1 NCRA|XP_956329.1 SSCL|APA12788.1

>Orthogroup2157: ANID|CBF69688.1 BCIN|XP_024549661.1 BGRA|VCU40093.1 CFRU|XP_031885317.1 CGLO|KAF3798653.1 CHIG|XP_018157508.1 CVIN|KAF4919470.1 CVYL|A09390 FGRM|XP_011328137.1 MGRA|XP_003855381.1 MLAR|XP_007410930.1 MORY|QBZ56581.1 NCRA|XP_962286.2 SSCL|APA06184.1

>Orthogroup2159: ANID|CBF69695.1 BCIN|XP_024549783.1 BGRA|VCU40154.1 CFRU|XP_031880611.1 CGLO|KAF3811522.1 CHIG|XP_018160779.1 CVIN|KAF4926735.1 CVYL|A04732 FGRM|XP_011324820.1 MGRA|XP_003848897.1 MLAR|XP_007418591.1 MORY|QBZ56624.1 NCRA|XP_961756.2 SSCL|APA05733.1

>Orthogroup2161: ANID|CBF69713.1 BCIN|XP_001545514.1 BGRA|VCU39244.1 CFRU|XP_031876735.1 CGLO|KAF3798672.1 CHIG|XP_018157482.1 CVIN|KAF4918471.1 CVYL|A09411 FGRM|XP_011317821.1 MGRA|XP_003855029.1 MLAR|XP_007409039.1 MORY|QBZ58988.1 NCRA|XP_956333.2 SSCL|APA13206.1

>Orthogroup2162: ANID|CBF69728.1 BCIN|XP_024549681.1 BGRA|VDB96306.1 CFRU|XP_031876947.1 CGLO|KAF3810702.1 CHIG|XP_018162467.1 CVIN|KAF4909748.1 CVYL|A07252 FGRM|XP_011326994.1 MGRA|XP_003854116.1 MLAR|XP_007408949.1 MORY|QBZ61440.1 NCRA|XP_957361.1 SSCL|APA05250.1

>Orthogroup2163: ANID|CBF69752.1 BCIN|XP_024552863.1 BGRA|VDB93074.1 CFRU|XP_031877992.1 CGLO|KAF3799628.1 CHIG|XP_018154634.1 CVIN|KAF4926432.1 CVYL|A13491 FGRM|XP_011315978.1 MGRA|XP_003852987.1 MLAR|XP_007412291.1 MORY|QBZ61022.1 NCRA|XP_963541.1 SSCL|APA16124.1

>Orthogroup2164: ANID|CBF69773.1 BCIN|XP_001554520.2 BGRA|VCU39583.1 CFRU|XP_031885336.1 CGLO|KAF3799477.1 CHIG|XP_018154462.1 CVIN|KAF4931790.1 CVYL|A13339 FGRM|XP_011316699.1 MGRA|XP_003856587.1 MLAR|XP_007413976.1 MORY|QBZ57266.1 NCRA|XP_963615.1 SSCL|APA11855.1

>Orthogroup2165: ANID|CBF69777.1 BCIN|XP_001554518.1 BGRA|VCU39577.1 CFRU|XP_031883391.1 CGLO|KAF3800533.1 CHIG|XP_018152153.1 CVIN|KAF4919220.1 CVYL|A13182 FGRM|XP_011315948.1 MGRA|XP_003857248.1 MLAR|XP_007406071.1 MORY|QBZ61084.1 NCRA|XP_956517.1 SSCL|APA11853.1

>Orthogroup2166: ANID|CBF69788.1 BCIN|XP_001557588.1 BGRA|VDB88831.1 CFRU|XP_031890975.1 CGLO|KAF3811717.1 CHIG|XP_018151814.1 CVIN|KAF4922980.1 CVYL|A12734 FGRM|XP_011319393.1 MGRA|XP_003857022.1 MLAR|XP_007417848.1 MORY|QBZ61166.1 NCRA|XP_963280.2 SSCL|APA12318.1

>Orthogroup2167: ANID|CBF69809.1 BCIN|XP_024553373.1 BGRA|VDB83655.1 CFRU|XP_031876539.1 CGLO|KAF3811482.1 CHIG|XP_018160735.1 CVIN|KAF4907413.1 CVYL|A04687 FGRM|XP_011317778.1 MGRA|XP_003848888.1 MLAR|XP_007413337.1 MORY|QBZ56558.1 NCRA|XP_962675.2 SSCL|APA13707.1

>Orthogroup2168: ANID|CBF69819.1 BCIN|XP_001549431.1 BGRA|VDB93052.1 CFRU|XP_031885359.1 CGLO|KAF3799511.1 CHIG|XP_018154499.1 CVIN|KAF4931871.1 CVYL|A13372 FGRM|XP_011324927.1 MGRA|XP_003856441.1 MLAR|XP_007407158.1 MORY|QBZ62058.1 NCRA|XP_956880.2 SSCL|APA11817.1

>Orthogroup2169: ANID|CBF69821.1 BCIN|XP_001554581.1 BGRA|VDB93047.1 CFRU|XP_031885328.1 CGLO|KAF3799524.1 CHIG|XP_018154513.1 CVIN|KAF4931768.1 CVYL|A13384 FGRM|XP_011321202.1 MGRA|XP_003857495.1 MLAR|XP_007406901.1 MORY|QBZ62093.1 NCRA|XP_956952.1 SSCL|APA11645.1

>Orthogroup2170: ANID|CBF69823.1 BCIN|XP_001554580.1 BGRA|VDB93046.1 CFRU|XP_031885494.1 CGLO|KAF3799523.1 CHIG|XP_018154512.1 CVIN|KAF4931767.1 CVYL|A13383 FGRM|XP_011321201.1 MGRA|XP_003856440.1 MLAR|XP_007408310.1 MORY|QBZ62077.1 NCRA|XP_956951.1 SSCL|APA11644.1

>Orthogroup2171: ANID|CBF69842.1 BCIN|XP_001546163.1 BGRA|VCU39668.1 CFRU|XP_031885460.1 CGLO|KAF3799536.1 CHIG|XP_018154526.1 CVIN|KAF4931837.1 CVYL|A13396 FGRM|XP_011324916.1 MGRA|XP_003857150.1 MLAR|XP_007410779.1 MORY|QBZ62070.1 NCRA|XP_963511.1 SSCL|APA16278.1

>Orthogroup2172: ANID|CBF69846.1 BCIN|XP_024553070.1 BGRA|VCU39667.1 CFRU|XP_031885454.1 CGLO|KAF3799534.1 CHIG|XP_018154524.1 CVIN|KAF4931819.1 CVYL|A13394 FGRM|XP_011324918.1 MGRA|XP_003856609.1 MLAR|XP_007409482.1 MORY|QBZ62068.1 NCRA|XP_963513.1 SSCL|APA16280.1

>Orthogroup2174: ANID|CBF69852.1 BCIN|XP_001554499.1 BGRA|VDB93033.1 CFRU|XP_031878005.1 CGLO|KAF3799624.1 CHIG|XP_018154628.1 CVIN|KAF4926486.1 CVYL|A13486 FGRM|XP_011317253.1 MGRA|XP_003857078.1 MLAR|XP_007414104.1 MORY|QBZ61006.1 NCRA|XP_956964.1 SSCL|APA11839.1

>Orthogroup2175: ANID|CBF69854.1 BCIN|XP_001546171.1 BGRA|VCU39655.1 CFRU|XP_031883394.1 CGLO|KAF3800529.1 CHIG|XP_018152157.1 CVIN|KAF4919250.1 CVYL|A13186 FGRM|XP_011325511.1 MGRA|XP_003852095.1 MLAR|XP_007409955.1 MORY|QBZ53987.1 NCRA|XP_956482.1 SSCL|APA16286.1

>Orthogroup2176: ANID|CBF69857.1 BCIN|XP_001554496.1 BGRA|VDB93034.1 CFRU|XP_031878060.1 CGLO|KAF3799621.1 CHIG|XP_018154624.1 CVIN|KAF4926493.1 CVYL|A13483 FGRM|XP_011317256.1 MGRA|XP_003857277.1 MLAR|XP_007412724.1 MORY|QBZ61003.1 NCRA|XP_956953.1 SSCL|APA11838.1

>Orthogroup2177: ANID|CBF69861.1 BCIN|XP_001554527.2 BGRA|VDB89078.1 CFRU|XP_031878006.1 CGLO|KAF3799625.1 CHIG|XP_018154629.1 CVIN|KAF4926489.1 CVYL|A13487 FGRM|XP_011315953.1 MGRA|XP_003856399.1 MLAR|XP_007408794.1 MORY|QBZ61007.1 NCRA|XP_956965.1 SSCL|APA11847.1

>Orthogroup2178: ANID|CBF69869.1 BCIN|XP_024552909.1 BGRA|VCU39650.1 CFRU|XP_031883278.1 CGLO|KAF3800527.1 CHIG|XP_018152185.1 CVIN|KAF4919257.1 CVYL|A13188 FGRM|XP_011325507.1 MGRA|XP_003856579.1 MLAR|XP_007416821.1 MORY|QBZ53990.1 NCRA|XP_956484.1 SSCL|APA07524.1

>Orthogroup2180: ANID|CBF69894.1 BCIN|XP_001557622.1 BGRA|VDB93021.1 CFRU|XP_031881295.1 CGLO|KAF3802460.1 CHIG|XP_018154760.1 CVIN|KAF4916862.1 CVYL|A02051 FGRM|XP_011316329.1 MGRA|XP_003853315.1 MLAR|XP_007412049.1 MORY|QBZ60974.1 NCRA|XP_963165.1 SSCL|APA12300.1

>Orthogroup2181: ANID|CBF69898.1 BCIN|XP_024551985.1 BGRA|VCU39273.1 CFRU|XP_031880673.1 CGLO|KAF3809954.1 CHIG|XP_018161537.1 CVIN|KAF4914182.1 CVYL|A01669 FGRM|XP_011318397.1 MGRA|XP_003849106.1 MLAR|XP_007418653.1 MORY|QBZ65251.1 NCRA|XP_962851.2 SSCL|APA12674.1

>Orthogroup2182: ANID|CBF69919.1 BCIN|XP_024549658.1 BGRA|VCU40087.1 CFRU|XP_031880580.1 CGLO|KAF3811558.1 CHIG|XP_018160819.1 CVIN|KAF4926727.1 CVYL|A04770 FGRM|XP_011324840.1 MGRA|XP_003849233.1 MLAR|XP_007406105.1 MORY|QBZ57247.1 NCRA|XP_955827.1 SSCL|APA06187.1

>Orthogroup2185: ANID|CBF69938.1 BCIN|XP_001558780.2 BGRA|VDB96298.1 CFRU|XP_031876949.1 CGLO|KAF3810706.1 CHIG|XP_018162472.1 CVIN|KAF4908239.1 CVYL|A07248 FGRM|XP_011323993.1 MGRA|XP_003853983.1 MLAR|XP_007418043.1 MORY|QBZ61446.1 NCRA|XP_957123.1 SSCL|APA05246.1

>Orthogroup2186: ANID|CBF69944.1 BCIN|XP_001558778.1 BGRA|VDB96288.1 CFRU|XP_031876939.1 CGLO|KAF3810704.1 CHIG|XP_018162470.1 CVIN|KAF4909744.1 CVYL|A07250 FGRM|XP_011326992.1 MGRA|XP_003854292.1 MLAR|XP_007403789.1 MORY|QBZ61442.1 NCRA|XP_957363.1 SSCL|APA05248.1

>Orthogroup2188: ANID|CBF69955.1 BCIN|XP_001547116.2 BGRA|VCU39265.1 CFRU|XP_031876533.1 CGLO|KAF3811474.1 CHIG|XP_018160727.1 CVIN|KAF4907417.1 CVYL|A04678 FGRM|XP_011317770.1 MGRA|XP_003849230.1 MLAR|XP_007411514.1 MORY|QBZ56600.1 NCRA|XP_962682.3 SSCL|APA08400.1

>Orthogroup2189: ANID|CBF69963.1 BCIN|XP_024553020.1 BGRA|VCU39711.1 CFRU|XP_031885380.1 CGLO|KAF3799496.1 CHIG|XP_018154480.1 CVIN|KAF4931775.1 CVYL|A13356 FGRM|XP_011321189.1 MGRA|XP_003856410.1 MLAR|XP_007404549.1 MORY|QBZ57259.1 NCRA|XP_963532.3 SSCL|APA16191.1

>Orthogroup2191: ANID|CBF69975.1 BCIN|XP_001551292.1 BGRA|VCU39700.1 CFRU|XP_031879465.1 CGLO|KAF3799636.1 CHIG|XP_018154645.1 CVIN|KAF4926430.1 CVYL|A13499 FGRM|XP_011324940.1 MGRA|XP_003857029.1 MLAR|XP_007414886.1 MORY|QBZ61037.1 NCRA|XP_963317.2 SSCL|APA07569.1

>Orthogroup2192: ANID|CBF69983.1 BCIN|XP_001550193.2 BGRA|VCU39722.1 CFRU|XP_031881301.1 CGLO|KAF3802468.1 CHIG|XP_018154772.1 CVIN|KAF4916865.1 CVYL|A02059 FGRM|XP_011317364.1 MGRA|XP_003856387.1 MLAR|XP_007408486.1 MORY|QBZ62056.1 NCRA|XP_956881.1 SSCL|APA16321.1

>Orthogroup2193: ANID|CBF69985.1 BCIN|XP_001546172.1 BGRA|VCU39656.1 CFRU|XP_031883393.1 CGLO|KAF3800530.1 CHIG|XP_018152156.1 CVIN|KAF4919252.1 CVYL|A13185 FGRM|XP_011325512.1 MGRA|XP_003857268.1 MLAR|XP_007410861.1 MORY|QBZ61033.1 NCRA|XP_956512.2 SSCL|APA16287.1

>Orthogroup2194: ANID|CBF70021.1 BCIN|XP_024553543.1 BGRA|VCU39935.1 CFRU|XP_031879072.1 CGLO|KAF3798849.1 CHIG|XP_018151182.1 CVIN|KAF4919607.1 CVYL|A09086 FGRM|XP_011324239.1 MGRA|XP_003852576.1 MLAR|XP_007403608.1 MORY|QBZ59438.1 NCRA|XP_957029.1 SSCL|APA13887.1

>Orthogroup2195: ANID|CBF70023.1 BCIN|XP_001554544.1 BGRA|VCU40848.1 CFRU|XP_031882442.1 CGLO|KAF3809409.1 CHIG|XP_018156256.1 CVIN|KAF4916329.1 CVYL|A03075 FGRM|XP_011319123.1 MGRA|XP_003851584.1 MLAR|XP_007411492.1 MORY|QBZ62747.1 NCRA|XP_964540.2 SSCL|APA11866.1

>Orthogroup2196: ANID|CBF70029.1 BCIN|XP_024549730.1 BGRA|VDB90988.1 CFRU|XP_031890662.1 CGLO|KAF3804938.1 CHIG|XP_018164440.1 CVIN|KAF4919428.1 CVYL|A12116 FGRM|XP_011316884.1 MGRA|XP_003851748.1 MLAR|XP_007403663.1 MORY|QBZ54649.1 NCRA|XP_964485.1 SSCL|APA05439.1

>Orthogroup2197: ANID|CBF70033.1 BCIN|XP_001558665.1 BGRA|VDB90984.1 CFRU|XP_031882896.1 CGLO|KAF3812091.1 CHIG|XP_018164162.1 CVIN|KAF4915996.1 CVYL|A14269 FGRM|XP_011316184.1 MGRA|XP_003851589.1 MLAR|XP_007413751.1 MORY|QBZ54652.1 NCRA|XP_964483.1 SSCL|APA05437.1

>Orthogroup2198: ANID|CBF70055.1 BCIN|XP_001550765.1 BGRA|VDB96367.1 CFRU|XP_031885106.1 CGLO|KAF3807749.1 CHIG|XP_018163019.1 CVIN|KAF4920538.1 CVYL|A00849 FGRM|XP_011326202.1 MGRA|XP_003853895.1 MLAR|XP_007417406.1 MORY|QBZ58189.1 NCRA|XP_011393935.1 SSCL|APA15279.1

>Orthogroup2199: ANID|CBF70061.1 BCIN|XP_024553689.1 BGRA|VDB96401.1 CFRU|XP_031878370.1 CGLO|KAF3810754.1 CHIG|XP_018162551.1 CVIN|KAF4932024.1 CVYL|A07201 FGRM|XP_011326322.1 MGRA|XP_003853721.1 MLAR|XP_007414356.1 MORY|QBZ59720.1 NCRA|XP_011393729.1 SSCL|APA15076.1

>Orthogroup2200: ANID|CBF70064.1 BCIN|XP_024553687.1 BGRA|VDB96422.1 CFRU|XP_031878369.1 CGLO|KAF3810756.1 CHIG|XP_018162553.1 CVIN|KAF4932045.1 CVYL|A07199 FGRM|XP_011326320.1 MGRA|XP_003854063.1 MLAR|XP_007404027.1 MORY|QBZ59713.1 NCRA|XP_011393733.1 SSCL|APA15074.1

>Orthogroup2201: ANID|CBF70068.1 BCIN|XP_001550749.1 BGRA|VDB96383.1 CFRU|XP_031892968.1 CGLO|KAF3808029.1 CHIG|XP_018152352.1 CVIN|KAF4909505.1 CVYL|A09455 FGRM|XP_011317746.1 MGRA|XP_003854494.1 MLAR|XP_007414673.1 MORY|QBZ54238.1 NCRA|XP_011394508.1 SSCL|APA15289.1

>Orthogroup2202: ANID|CBF70089.1 BCIN|XP_001550736.1 BGRA|VDB93373.1 CFRU|XP_031883158.1 CGLO|KAF3800737.1 CHIG|XP_018163287.1 CVIN|KAF4919652.1 CVYL|A00467 FGRM|XP_011326140.1 MGRA|XP_003854447.1 MLAR|XP_007409855.1 MORY|QBZ57932.1 NCRA|XP_963033.2 SSCL|APA15303.1

>Orthogroup2203: ANID|CBF70091.1 BCIN|XP_001550737.1 BGRA|VDB90949.1 CFRU|XP_031883157.1 CGLO|KAF3800736.1 CHIG|XP_018163286.1 CVIN|KAF4919617.1 CVYL|A00468 FGRM|XP_011326141.1 MGRA|XP_003853816.1 MLAR|XP_007417058.1 MORY|QBZ57931.1 NCRA|XP_963034.2 SSCL|APA15302.1

>Orthogroup2204: ANID|CBF70100.1 BCIN|XP_001550535.1 BGRA|VDB93419.1 CFRU|XP_031891681.1 CGLO|KAF3800130.1 CHIG|XP_018165064.1 CVIN|KAF4925818.1 CVYL|A06817 FGRM|XP_011323519.1 MGRA|XP_003853781.1 MLAR|XP_007412780.1 MORY|QBZ58691.1 NCRA|XP_962256.1 SSCL|APA15254.1

>Orthogroup2205: ANID|CBF70108.1 BCIN|XP_001549835.1 BGRA|VDB93488.1 CFRU|XP_031883064.1 CGLO|KAF3808248.1 CHIG|XP_018163166.1 CVIN|KAF4909565.1 CVYL|A00584 FGRM|XP_011326198.1 MGRA|XP_003853752.1 MLAR|XP_007412384.1 MORY|QBZ57842.1 NCRA|XP_958364.1 SSCL|APA14443.1

>Orthogroup2206: ANID|CBF70134.1 BCIN|XP_024550059.1 BGRA|VDB93407.1 CFRU|XP_031883230.1 CGLO|KAF3808294.1 CHIG|XP_018163126.1 CVIN|KAF4924044.1 CVYL|A00634 FGRM|XP_011326197.1 MGRA|XP_003853978.1 MLAR|XP_007407224.1 MORY|QBZ58253.1 NCRA|XP_011393963.1 SSCL|APA14453.1

>Orthogroup2207: ANID|CBF70138.1 BCIN|XP_001557147.1 BGRA|VDB93506.1 CFRU|XP_031886305.1 CGLO|KAF3806227.1 CHIG|XP_018162062.1 CVIN|KAF4931527.1 CVYL|A11817 FGRM|XP_011324035.1 MGRA|XP_003853753.1 MLAR|XP_007414677.1 MORY|QBZ61543.1 NCRA|XP_001728497.1 SSCL|APA15029.1

>Orthogroup2208: ANID|CBF70162.1 BCIN|XP_024553693.1 BGRA|VDB96421.1 CFRU|XP_031886342.1 CGLO|KAF3806371.1 CHIG|XP_018162220.1 CVIN|KAF4923264.1 CVYL|A11960 FGRM|XP_011324056.1 MGRA|XP_003853709.1 MLAR|XP_007405514.1 MORY|QBZ55735.1 NCRA|XP_957805.1 SSCL|APA15084.1

>Orthogroup2210: ANID|CBF70170.1 BCIN|XP_024553694.1 BGRA|VDB96420.1 CFRU|XP_031886816.1 CGLO|KAF3801206.1 CHIG|XP_018162490.1 CVIN|KAF4932037.1 CVYL|A07126 FGRM|XP_011324105.1 MGRA|XP_003853892.1 MLAR|XP_007404056.1 MORY|QBZ55571.1 NCRA|XP_957245.3 SSCL|APA15083.1

>Orthogroup2214: ANID|CBF70208.1 BCIN|XP_001552177.1 BGRA|VDB93944.1 CFRU|XP_031878051.1 CGLO|KAF3799562.1 CHIG|XP_018154570.1 CVIN|KAF4931851.1 CVYL|A13421 FGRM|XP_011324894.1 MGRA|XP_003850173.1 MLAR|XP_007415705.1 MORY|QBZ61066.1 NCRA|XP_956500.3 SSCL|APA05596.1

>Orthogroup2215: ANID|CBF70211.1 BCIN|XP_001559569.1 BGRA|VDB83872.1 CFRU|XP_031886628.1 CGLO|KAF3798901.1 CHIG|XP_018158573.1 CVIN|KAF4925598.1 CVYL|A05051 FGRM|XP_011328433.1 MGRA|XP_003848482.1 MLAR|XP_007406633.1 MORY|QBZ60474.1 NCRA|XP_963772.1 SSCL|APA09583.1

>Orthogroup2216: ANID|CBF70213.1 BCIN|XP_024548920.1 BGRA|VDB83866.1 CFRU|XP_031886627.1 CGLO|KAF3798900.1 CHIG|XP_018158572.1 CVIN|KAF4925570.1 CVYL|A05052 FGRM|XP_011328432.1 MGRA|XP_003848493.1 MLAR|XP_007404755.1 MORY|QBZ60470.1 NCRA|XP_964195.1 SSCL|APA09582.1

>Orthogroup2217: ANID|CBF70221.1 BCIN|XP_001551814.1 BGRA|VCU40000.1 CFRU|XP_031892680.1 CGLO|KAF3809124.1 CHIG|XP_018155970.1 CVIN|KAF4922085.1 CVYL|A01308 FGRM|XP_011319196.1 MGRA|XP_003851784.1 MLAR|XP_007409965.1 MORY|QBZ61104.1 NCRA|XP_960786.1 SSCL|APA11950.1

>Orthogroup2218: ANID|CBF70239.1 BCIN|XP_001545753.1 BGRA|VDB91040.1 CFRU|XP_031888408.1 CGLO|KAF3803685.1 CHIG|XP_018163771.1 CVIN|KAF4928533.1 CVYL|A14616 FGRM|XP_011316032.1 MGRA|XP_003851720.1 MLAR|XP_007415367.1 MORY|QBZ54646.1 NCRA|XP_964488.2 SSCL|APA05479.1

>Orthogroup2219: ANID|CBF70241.1 BCIN|XP_001545752.1 BGRA|VDB91039.1 CFRU|XP_031890664.1 CGLO|KAF3804936.1 CHIG|XP_018164438.1 CVIN|KAF4919429.1 CVYL|A12118 FGRM|XP_011316886.1 MGRA|XP_003851582.1 MLAR|XP_007405013.1 MORY|QBZ54647.1 NCRA|XP_964487.1 SSCL|APA05480.1

>Orthogroup2221: ANID|CBF70251.1 BCIN|XP_024553700.1 BGRA|VDB93327.1 CFRU|XP_031877197.1 CGLO|KAF3806427.1 CHIG|XP_018162312.1 CVIN|KAF4918042.1 CVYL|A12021 FGRM|XP_011324125.1 MGRA|XP_003854547.1 MLAR|XP_007417529.1 MORY|QBZ55739.1 NCRA|XP_957816.1 SSCL|APA15077.1

>Orthogroup2222: ANID|CBF70253.1 BCIN|XP_001551174.1 BGRA|VDB93326.1 CFRU|XP_031877209.1 CGLO|KAF3806423.1 CHIG|XP_018162308.1 CVIN|KAF4918035.1 CVYL|A12016 FGRM|XP_011323961.1 MGRA|XP_003853842.1 MLAR|XP_007407792.1 MORY|QBZ55744.1 NCRA|XP_957252.3 SSCL|APA15078.1

>Orthogroup2225: ANID|CBF70279.1 BCIN|XP_001561373.1 BGRA|VDB95099.1 CFRU|XP_031878113.1 CGLO|KAF3801832.1 CHIG|XP_018159309.1 CVIN|KAF4907399.1 CVYL|A12874 FGRM|XP_011320461.1 MGRA|XP_003854755.1 MLAR|XP_007411152.1 MORY|QBZ62874.1 NCRA|XP_960479.1 SSCL|APA06886.1

>Orthogroup2226: ANID|CBF70305.1 BCIN|XP_024547577.1 BGRA|VDB86208.1 CFRU|XP_031877226.1 CGLO|KAF3806457.1 CHIG|XP_018162345.1 CVIN|KAF4897890.1 CVYL|A12056 FGRM|XP_011323936.1 MGRA|XP_003855784.1 MLAR|XP_007409084.1 MORY|QBZ61398.1 NCRA|XP_956406.1 SSCL|APA07839.1

>Orthogroup2227: ANID|CBF70307.1 BCIN|XP_001549722.2 BGRA|VDB93115.1 CFRU|XP_031876563.1 CGLO|KAF3800884.1 CHIG|XP_018150807.1 CVIN|KAF4911855.1 CVYL|A11122 FGRM|XP_011324234.1 MGRA|XP_003852454.1 MLAR|XP_007405355.1 MORY|QBZ64238.1 NCRA|XP_962606.1 SSCL|APA13896.1

>Orthogroup2228: ANID|CBF70313.1 BCIN|XP_024553485.1 BGRA|VCU40122.1 CFRU|XP_031893263.1 CGLO|KAF3800876.1 CHIG|XP_018150816.1 CVIN|KAF4915569.1 CVYL|A11114 FGRM|XP_011324444.1 MGRA|XP_003852404.1 MLAR|XP_007417678.1 MORY|QBZ61309.1 NCRA|XP_962135.1 SSCL|APA13789.1

>Orthogroup2229: ANID|CBF70315.1 BCIN|XP_001546212.1 BGRA|VCU40136.1 CFRU|XP_031876589.1 CGLO|KAF3800900.1 CHIG|XP_018150779.1 CVIN|KAF4907263.1 CVYL|A12439 FGRM|XP_011324476.1 MGRA|XP_003853249.1 MLAR|XP_007411999.1 MORY|QBZ64277.1 NCRA|XP_962599.1 SSCL|APA13795.1

>Orthogroup2231: ANID|CBF70364.1 BCIN|XP_001545853.1 BGRA|VCU40184.1 CFRU|XP_031886020.1 CGLO|KAF3806572.1 CHIG|XP_018157611.1 CVIN|KAF4920454.1 CVYL|A09295 FGRM|XP_011328120.1 MGRA|XP_003852349.1 MLAR|XP_007410381.1 MORY|QBZ56369.1 NCRA|XP_957458.2 SSCL|APA05502.1

>Orthogroup2233: ANID|CBF70374.1 BCIN|XP_001553016.1 BGRA|VDB84414.1 CFRU|XP_031891326.1 CGLO|KAF3797638.1 CHIG|XP_018157430.1 CVIN|KAF4893857.1 CVYL|A11029 FGRM|XP_011323033.1 MGRA|XP_003854586.1 MLAR|XP_007410791.1 MORY|QBZ66384.1 NCRA|XP_965488.1 SSCL|APA11238.1

>Orthogroup2235: ANID|CBF70399.1 BCIN|XP_024547032.1 BGRA|VDB93718.1 CFRU|XP_031888539.1 CGLO|KAF3803753.1 CHIG|XP_018153176.1 CVIN|KAF4912468.1 CVYL|A14133 FGRM|XP_011316827.1 MGRA|XP_003856486.1 MLAR|XP_007415588.1 MORY|QBZ59788.1 NCRA|XP_965644.1 SSCL|APA05917.1

>Orthogroup2236: ANID|CBF70403.1 BCIN|XP_001556462.1 BGRA|VDB92955.1 CFRU|XP_031888395.1 CGLO|KAF3803700.1 CHIG|XP_018163751.1 CVIN|KAF4928576.1 CVYL|A14632 FGRM|XP_011326807.1 MGRA|XP_003851147.1 MLAR|XP_007407009.1 MORY|QBZ54617.1 NCRA|XP_963878.1 SSCL|APA06545.1

>Orthogroup2237: ANID|CBF70405.1 BCIN|XP_001556463.1 BGRA|VDB92957.1 CFRU|XP_031888583.1 CGLO|KAF3803699.1 CHIG|XP_018163752.1 CVIN|KAF4928575.1 CVYL|A14631 FGRM|XP_011326806.1 MGRA|XP_003851262.1 MLAR|XP_007408814.1 MORY|QBZ54615.1 NCRA|XP_963877.1 SSCL|APA06544.1

>Orthogroup2238: ANID|CBF70422.1 BCIN|XP_024546753.1 BGRA|VDB95170.1 CFRU|XP_031879139.1 CGLO|KAF3807079.1 CHIG|XP_018159736.1 CVIN|KAF4919045.1 CVYL|A06373 FGRM|XP_011320682.1 MGRA|XP_003855774.1 MLAR|XP_007415463.1 MORY|QBZ63985.1 NCRA|XP_960570.2 SSCL|APA06396.1

>Orthogroup2239: ANID|CBF70424.1 BCIN|XP_024552044.1 BGRA|VDB92805.1 CFRU|XP_031887695.1 CGLO|KAF3803218.1 CHIG|XP_018161063.1 CVIN|KAF4928718.1 CVYL|A03648 FGRM|XP_011324205.1 MGRA|XP_003853144.1 MLAR|XP_007414698.1 MORY|QBZ64605.1 NCRA|XP_961833.1 SSCL|APA12823.1

>Orthogroup2240: ANID|CBF70428.1 BCIN|XP_001557855.1 BGRA|VDB92803.1 CFRU|XP_031887704.1 CGLO|KAF3803216.1 CHIG|XP_018161061.1 CVIN|KAF4928724.1 CVYL|A03646 FGRM|XP_011324203.1 MGRA|XP_003852316.1 MLAR|XP_007411639.1 MORY|QBZ64607.1 NCRA|XP_961835.1 SSCL|APA12825.1

>Orthogroup2242: ANID|CBF70445.1 BCIN|XP_024553558.1 BGRA|VDB93123.1 CFRU|XP_031893265.1 CGLO|KAF3800874.1 CHIG|XP_018150820.1 CVIN|KAF4915594.1 CVYL|A11112 FGRM|XP_011324445.1 MGRA|XP_003852629.1 MLAR|XP_007408733.1 MORY|QBZ54775.1 NCRA|XP_962477.2 SSCL|APA13899.1

>Orthogroup2243: ANID|CBF70449.1 BCIN|XP_001554935.1 BGRA|VCU39330.1 CFRU|XP_031885342.1 CGLO|KAF3806521.1 CHIG|XP_018157558.1 CVIN|KAF4921331.1 CVYL|A09349 FGRM|XP_011324812.1 MGRA|XP_003852340.1 MLAR|XP_007410260.1 MORY|QBZ58923.1 NCRA|XP_962378.2 SSCL|APA08363.1

>Orthogroup2244: ANID|CBF70472.1 BCIN|XP_024547888.1 BGRA|VDB84454.1 CFRU|XP_031891379.1 CGLO|KAF3804714.1 CHIG|XP_018161801.1 CVIN|KAF4923377.1 CVYL|A05408 FGRM|XP_011321495.1 MGRA|XP_003855745.1 MLAR|XP_007416339.1 MORY|QBZ59672.1 NCRA|XP_964828.1 SSCL|APA08210.1

>Orthogroup2246: ANID|CBF70493.1 BCIN|XP_001555077.2 BGRA|VDB86166.1 CFRU|XP_031878230.1 CGLO|KAF3808543.1 CHIG|XP_018161931.1 CVIN|KAF4917639.1 CVYL|A11599 FGRM|XP_011318509.1 MGRA|XP_003855782.1 MLAR|XP_007407064.1 MORY|QBZ59648.1 NCRA|XP_962883.1 SSCL|APA07830.1

>Orthogroup2247: ANID|CBF70499.1 BCIN|XP_024552137.1 BGRA|VDB92808.1 CFRU|XP_031887543.1 CGLO|KAF3803291.1 CHIG|XP_018161141.1 CVIN|KAF4928693.1 CVYL|A03726 FGRM|XP_011327966.1 MGRA|XP_003852263.1 MLAR|XP_007404083.1 MORY|QBZ64563.1 NCRA|XP_961891.3 SSCL|APA12970.1

>Orthogroup2248: ANID|CBF70501.1 BCIN|XP_024552138.1 BGRA|VDB92822.1 CFRU|XP_031887540.1 CGLO|KAF3803292.1 CHIG|XP_018161142.1 CVIN|KAF4928694.1 CVYL|A03727 FGRM|XP_011327967.1 MGRA|XP_003852546.1 MLAR|XP_007407879.1 MORY|QBZ64564.1 NCRA|XP_961892.2 SSCL|APA12971.1

>Orthogroup2250: ANID|CBF70539.1 BCIN|XP_001553328.1 BGRA|VDB85759.1 CFRU|XP_031877195.1 CGLO|KAF3806439.1 CHIG|XP_018162327.1 CVIN|KAF4918063.1 CVYL|A12036 FGRM|XP_011323957.1 MGRA|XP_003854330.1 MLAR|XP_007410482.1 MORY|QBZ61416.1 NCRA|XP_963131.2 SSCL|APA15173.1

>Orthogroup2251: ANID|CBF70577.1 BCIN|XP_001560887.1 BGRA|VDB84361.1 CFRU|XP_031886528.1 CGLO|KAF3800262.1 CHIG|XP_018162748.1 CVIN|KAF4927565.1 CVYL|A06975 FGRM|XP_011327030.1 MGRA|XP_003853902.1 MLAR|XP_007406808.1 MORY|QBZ61571.1 NCRA|XP_957339.1 SSCL|APA08101.1

>Orthogroup2252: ANID|CBF70579.1 BCIN|XP_001560888.1 BGRA|VDB84360.1 CFRU|XP_031886525.1 CGLO|KAF3800261.1 CHIG|XP_018162747.1 CVIN|KAF4927568.1 CVYL|A06977 FGRM|XP_011327031.1 MGRA|XP_003854364.1 MLAR|XP_007410012.1 MORY|QBZ61570.1 NCRA|XP_957338.1 SSCL|APA08102.1

>Orthogroup2253: ANID|CBF70588.1 BCIN|XP_001560890.1 BGRA|VDB84431.1 CFRU|XP_031886526.1 CGLO|KAF3800260.1 CHIG|XP_018162746.1 CVIN|KAF4927567.1 CVYL|A06978 FGRM|XP_011327032.1 MGRA|XP_003854664.1 MLAR|XP_007410572.1 MORY|QBZ61569.1 NCRA|XP_957337.1 SSCL|APA08103.1

>Orthogroup2256: ANID|CBF70596.1 BCIN|XP_001546783.1 BGRA|VCU40127.1 CFRU|XP_031893383.1 CGLO|KAF3800846.1 CHIG|XP_018150852.1 CVIN|KAF4915590.1 CVYL|A11085 FGRM|XP_011328034.1 MGRA|XP_003852539.1 MLAR|XP_007417297.1 MORY|QBZ56854.1 NCRA|XP_001728535.2 SSCL|APA13864.1

>Orthogroup2257: ANID|CBF70599.1 BCIN|XP_024547974.1 BGRA|VCU39372.1 CFRU|XP_031885345.1 CGLO|KAF3806518.1 CHIG|XP_018157555.1 CVIN|KAF4921334.1 CVYL|A09352 FGRM|XP_011327983.1 MGRA|XP_003852588.1 MLAR|XP_007416801.1 MORY|QBZ58925.1 NCRA|XP_962380.1 SSCL|APA08365.1

>Orthogroup2258: ANID|CBF70605.1 BCIN|XP_001546799.1 BGRA|VCU40114.1 CFRU|XP_031878682.1 CGLO|KAF3803835.1 CHIG|XP_018151095.1 CVIN|KAF4888648.1 CVYL|A08965 FGRM|XP_011323758.1 MGRA|XP_003847973.1 MLAR|XP_007415866.1 MORY|QBZ64230.1 NCRA|XP_011394164.1 SSCL|APA13855.1

>Orthogroup2259: ANID|CBF70609.1 BCIN|XP_024550012.1 BGRA|VDB83693.1 CFRU|XP_031887711.1 CGLO|KAF3811650.1 CHIG|XP_018160963.1 CVIN|KAF4921440.1 CVYL|A03510 FGRM|XP_011323601.1 MGRA|XP_003853244.1 MLAR|XP_007405787.1 MORY|QBZ57184.1 NCRA|XP_962321.1 SSCL|APA05344.1

>Orthogroup2260: ANID|CBF70624.1 BCIN|XP_024549846.1 BGRA|VCU39314.1 CFRU|XP_031887692.1 CGLO|KAF3807144.1 CHIG|XP_018160965.1 CVIN|KAF4920164.1 CVYL|A03544 FGRM|XP_011324877.1 MGRA|XP_003852283.1 MLAR|XP_007410078.1 MORY|QBZ56305.1 NCRA|XP_962530.3 SSCL|APA05495.1

>Orthogroup2261: ANID|CBF70629.1 BCIN|XP_001545547.1 BGRA|VDB83723.1 CFRU|XP_031876730.1 CGLO|KAF3798677.1 CHIG|XP_018157478.1 CVIN|KAF4918497.1 CVYL|A09416 FGRM|XP_011324826.1 MGRA|XP_003852287.1 MLAR|XP_007404072.1 MORY|QBZ56512.1 NCRA|XP_011394090.1 SSCL|APA13612.1

>Orthogroup2262: ANID|CBF70631.1 BCIN|XP_024553237.1 BGRA|VDB94455.1 CFRU|XP_031886201.1 CGLO|KAF3806581.1 CHIG|XP_018157620.1 CVIN|KAF4920464.1 CVYL|A09287 FGRM|XP_011328127.1 MGRA|XP_003852524.1 MLAR|XP_007403548.1 MORY|QBZ56376.1 NCRA|XP_001728541.1 SSCL|APA13611.1

>Orthogroup2263: ANID|CBF70635.1 BCIN|XP_001547723.2 BGRA|VCU40531.1 CFRU|XP_031886019.1 CGLO|KAF3806574.1 CHIG|XP_018157612.1 CVIN|KAF4920462.1 CVYL|A09294 FGRM|XP_011328121.1 MGRA|XP_003852586.1 MLAR|XP_007414759.1 MORY|QBZ56370.1 NCRA|XP_957462.1 SSCL|APA05372.1

>Orthogroup2264: ANID|CBF70640.1 BCIN|XP_001551452.1 BGRA|VCU39534.1 CFRU|XP_031880590.1 CGLO|KAF3811548.1 CHIG|XP_018160808.1 CVIN|KAF4926780.1 CVYL|A04760 FGRM|XP_011324799.1 MGRA|XP_003852464.1 MLAR|XP_007403788.1 MORY|QBZ57226.1 NCRA|XP_962061.1 SSCL|APA06178.1

>Orthogroup2265: ANID|CBF70642.1 BCIN|XP_024550019.1 BGRA|VDB83672.1 CFRU|XP_031885300.1 CGLO|KAF3806496.1 CHIG|XP_018157532.1 CVIN|KAF4919437.1 CVYL|A09373 FGRM|XP_011328139.1 MGRA|XP_003852471.1 MLAR|XP_007408404.1 MORY|QBZ56568.1 NCRA|XP_961729.1 SSCL|APA05353.1

>Orthogroup2266: ANID|CBF70647.1 BCIN|XP_001558323.1 BGRA|VDB88075.1 CFRU|XP_031886205.1 CGLO|KAF3806578.1 CHIG|XP_018157616.1 CVIN|KAF4920467.1 CVYL|A09290 FGRM|XP_011328124.1 MGRA|XP_003852628.1 MLAR|XP_007410614.1 MORY|QBZ56373.1 NCRA|XP_957464.1 SSCL|APA13703.1

>Orthogroup2268: ANID|CBF70657.1 BCIN|XP_001545709.1 BGRA|VDB83643.1 CFRU|XP_031880518.1 CGLO|KAF3811534.1 CHIG|XP_018160793.1 CVIN|KAF4926769.1 CVYL|A04744 FGRM|XP_011324846.1 MGRA|XP_003852221.1 MLAR|XP_007411552.1 MORY|QBZ58763.1 NCRA|XP_962180.1 SSCL|APA05369.1

>Orthogroup2269: ANID|CBF70666.1 BCIN|XP_001547757.1 BGRA|VCU40108.1 CFRU|XP_031880498.1 CGLO|KAF3811553.1 CHIG|XP_018160813.1 CVIN|KAF4926787.1 CVYL|A04766 FGRM|XP_011324792.1 MGRA|XP_003848922.1 MLAR|XP_007417225.1 MORY|QBZ57218.1 NCRA|XP_962066.1 SSCL|APA05351.1

>Orthogroup2270: ANID|CBF70674.1 BCIN|XP_001549744.2 BGRA|VCU40135.1 CFRU|XP_031876552.1 CGLO|KAF3811497.1 CHIG|XP_018160751.1 CVIN|KAF4926749.1 CVYL|A04702 FGRM|XP_011317793.1 MGRA|XP_003849019.1 MLAR|XP_007415673.1 MORY|QBZ56322.1 NCRA|XP_011394345.1 SSCL|APA13874.1

>Orthogroup2271: ANID|CBF70681.1 BCIN|XP_024549665.1 BGRA|VCU40091.1 CFRU|XP_031880566.1 CGLO|KAF3811547.1 CHIG|XP_018160807.1 CVIN|KAF4926779.1 CVYL|A04759 FGRM|XP_011324800.1 MGRA|XP_003852558.1 MLAR|XP_007416180.1 MORY|QBZ57227.1 NCRA|XP_962060.3 SSCL|APA06179.1

>Orthogroup2272: ANID|CBF70700.1 BCIN|XP_001552935.1 BGRA|VDB90990.1 CFRU|XP_031888403.1 CGLO|KAF3803687.1 CHIG|XP_018163766.1 CVIN|KAF4928536.1 CVYL|A14618 FGRM|XP_011316033.1 MGRA|XP_003851792.1 MLAR|XP_007418934.1 MORY|QBZ54645.1 NCRA|XP_964489.1 SSCL|APA05429.1

>Orthogroup2275: ANID|CBF70776.1 BCIN|XP_001557924.1 BGRA|VDB92782.1 CFRU|XP_031889148.1 CGLO|KAF3797678.1 CHIG|XP_018155455.1 CVIN|KAF4919390.1 CVYL|A01092 FGRM|XP_011323895.1 MGRA|XP_003855779.1 MLAR|XP_007417972.1 MORY|QBZ64486.1 NCRA|XP_011395004.1 SSCL|APA12652.1

>Orthogroup2276: ANID|CBF70787.1 BCIN|XP_001545640.2 BGRA|VDB93621.1 CFRU|XP_031886620.1 CGLO|KAF3801230.1 CHIG|XP_018162612.1 CVIN|KAF4932135.1 CVYL|A07100 FGRM|XP_011326638.1 MGRA|XP_003852325.1 MLAR|XP_007409359.1 MORY|QBZ62825.1 NCRA|XP_957230.2 SSCL|APA15091.1

>Orthogroup2277: ANID|CBF70789.1 BCIN|XP_024553701.1 BGRA|VDB93588.1 CFRU|XP_031886619.1 CGLO|KAF3801231.1 CHIG|XP_018162613.1 CVIN|KAF4932134.1 CVYL|A07099 FGRM|XP_011326637.1 MGRA|XP_003852174.1 MLAR|XP_007416245.1 MORY|QBZ62826.1 NCRA|XP_957231.3 SSCL|APA15092.1

>Orthogroup2279: ANID|CBF70817.1 BCIN|XP_001554379.1 BGRA|VDB93340.1 CFRU|XP_031876390.1 CGLO|KAF3797780.1 CHIG|XP_018156451.1 CVIN|KAF4923169.1 CVYL|A03002 FGRM|XP_011318764.1 MGRA|XP_003851390.1 MLAR|XP_007408150.1 MORY|QBZ63505.1 NCRA|XP_956577.2 SSCL|APA12372.1

>Orthogroup2280: ANID|CBF70821.1 BCIN|XP_001550923.1 BGRA|VDB93338.1 CFRU|XP_031876404.1 CGLO|KAF3797777.1 CHIG|XP_018156448.1 CVIN|KAF4918812.1 CVYL|A02999 FGRM|XP_011318767.1 MGRA|XP_003851503.1 MLAR|XP_007409754.1 MORY|QBZ63511.1 NCRA|XP_956574.1 SSCL|APA12378.1

>Orthogroup2281: ANID|CBF70827.1 BCIN|XP_001548141.1 BGRA|VCU40942.1 CFRU|XP_031887448.1 CGLO|KAF3809193.1 CHIG|XP_018156017.1 CVIN|KAF4925299.1 CVYL|A01382 FGRM|XP_011319113.1 MGRA|XP_003851556.1 MLAR|XP_007407013.1 MORY|QBZ62562.1 NCRA|XP_960831.2 SSCL|APA12750.1

>Orthogroup2282: ANID|CBF70845.1 BCIN|XP_024547837.1 BGRA|VCU38809.1 CFRU|XP_031877745.1 CGLO|KAF3796943.1 CHIG|XP_018162390.1 CVIN|KAF4911635.1 CVYL|A07322 FGRM|XP_011318554.1 MGRA|XP_003849904.1 MLAR|XP_007416635.1 MORY|QBZ62837.1 NCRA|XP_957241.2 SSCL|APA08146.1

>Orthogroup2283: ANID|CBF70850.1 BCIN|XP_024554010.1 BGRA|VDB84426.1 CFRU|XP_031877763.1 CGLO|KAF3810674.1 CHIG|XP_018162431.1 CVIN|KAF4916943.1 CVYL|A07285 FGRM|XP_011318442.1 MGRA|XP_003850511.1 MLAR|XP_007410931.1 MORY|QBZ55711.1 NCRA|XP_962931.3 SSCL|APA15371.1

>Orthogroup2284: ANID|CBF70856.1 BCIN|XP_001550836.1 BGRA|VDB84321.1 CFRU|XP_031877777.1 CGLO|KAF3810671.1 CHIG|XP_018162428.1 CVIN|KAF4916954.1 CVYL|A07290 FGRM|XP_011318438.1 MGRA|XP_003850072.1 MLAR|XP_007403819.1 MORY|QBZ55708.1 NCRA|XP_962928.3 SSCL|APA15337.1

>Orthogroup2285: ANID|CBF70867.1 BCIN|XP_024553973.1 BGRA|VDB84437.1 CFRU|XP_031886956.1 CGLO|KAF3811246.1 CHIG|XP_018160471.1 CVIN|KAF4931245.1 CVYL|A04416 FGRM|XP_011327096.1 MGRA|XP_003850487.1 MLAR|XP_007410349.1 MORY|QBZ64765.1 NCRA|XP_961140.1 SSCL|APA15408.1

>Orthogroup2286: ANID|CBF70878.1 BCIN|XP_024553979.1 BGRA|VDB84359.1 CFRU|XP_031880706.1 CGLO|KAF3811204.1 CHIG|XP_018160445.1 CVIN|KAF4931176.1 CVYL|A04370 FGRM|XP_011327079.1 MGRA|XP_003849916.1 MLAR|XP_007410762.1 MORY|QBZ64768.1 NCRA|XP_011394632.1 SSCL|APA15412.1

>Orthogroup2287: ANID|CBF70884.1 BCIN|XP_001549190.1 BGRA|VDB84357.1 CFRU|XP_031887007.1 CGLO|KAF3811233.1 CHIG|XP_018160456.1 CVIN|KAF4931189.1 CVYL|A04433 FGRM|XP_011327081.1 MGRA|XP_003848902.1 MLAR|XP_007416043.1 MORY|QBZ63415.1 NCRA|XP_961545.1 SSCL|APA15415.1

>Orthogroup2288: ANID|CBF70887.1 BCIN|XP_001553356.1 BGRA|VDB84434.1 CFRU|XP_031886880.1 CGLO|KAF3811241.1 CHIG|XP_018160464.1 CVIN|KAF4931171.1 CVYL|A04424 FGRM|XP_011327089.1 MGRA|XP_003855629.1 MLAR|XP_007405277.1 MORY|QBZ64760.1 NCRA|XP_961136.1 SSCL|APA15152.1

>Orthogroup2289: ANID|CBF70889.1 BCIN|XP_001553355.1 BGRA|VDB84433.1 CFRU|XP_031882182.1 CGLO|KAF3804426.1 CHIG|XP_018160248.1 CVIN|KAF4930883.1 CVYL|A04132 FGRM|XP_011326883.1 MGRA|XP_003854875.1 MLAR|XP_007416073.1 MORY|QBZ59056.1 NCRA|XP_960859.1 SSCL|APA15153.1

>Orthogroup2290: ANID|CBF70899.1 BCIN|XP_001560643.1 BGRA|VDB86042.1 CFRU|XP_031887049.1 CGLO|KAF3811262.1 CHIG|XP_018160483.1 CVIN|KAF4931279.1 CVYL|A04401 FGRM|XP_011326926.1 MGRA|XP_003848854.1 MLAR|XP_007411710.1 MORY|QBZ58101.1 NCRA|XP_001728317.2 SSCL|APA07945.1

>Orthogroup2291: ANID|CBF70905.1 BCIN|XP_024547663.1 BGRA|VDB85968.1 CFRU|XP_031891162.1 CGLO|KAF3804492.1 CHIG|XP_018160320.1 CVIN|KAF4930972.1 CVYL|A04206 FGRM|XP_011324752.1 MGRA|XP_003855384.1 MLAR|XP_007407970.1 MORY|QBZ64687.1 NCRA|XP_961204.3 SSCL|APA07736.1

>Orthogroup2292: ANID|CBF70910.1 BCIN|XP_024554059.1 BGRA|VDB93848.1 CFRU|XP_031880751.1 CGLO|KAF3811174.1 CHIG|XP_018159498.1 CVIN|KAF4931285.1 CVYL|A04337 FGRM|XP_011323731.1 MGRA|XP_003848949.1 MLAR|XP_007408458.1 MORY|QBZ64660.1 NCRA|XP_961305.2 SSCL|APA15359.1

>Orthogroup2293: ANID|CBF70912.1 BCIN|XP_024554029.1 BGRA|VDB84261.1 CFRU|XP_031880742.1 CGLO|KAF3811169.1 CHIG|XP_018159490.1 CVIN|KAF4931232.1 CVYL|A04331 FGRM|XP_011323728.1 MGRA|XP_003848951.1 MLAR|XP_007407268.1 MORY|QBZ64667.1 NCRA|XP_955991.1 SSCL|APA15343.1

>Orthogroup2296: ANID|CBF70937.1 BCIN|XP_001560641.1 BGRA|VDB86036.1 CFRU|XP_031886919.1 CGLO|KAF3811274.1 CHIG|XP_018160498.1 CVIN|KAF4927261.1 CVYL|A04460 FGRM|XP_011324717.1 MGRA|XP_003850107.1 MLAR|XP_007409891.1 MORY|QBZ58103.1 NCRA|XP_961386.1 SSCL|APA07943.1

>Orthogroup2297: ANID|CBF70954.1 BCIN|XP_001553370.1 BGRA|VDB84352.1 CFRU|XP_031886876.1 CGLO|KAF3811237.1 CHIG|XP_018160460.1 CVIN|KAF4931163.1 CVYL|A04428 FGRM|XP_011327085.1 MGRA|XP_003848876.1 MLAR|XP_007416664.1 MORY|QBZ64756.1 NCRA|XP_960730.1 SSCL|APA15140.1

>Orthogroup2298: ANID|CBF70956.1 BCIN|XP_001553373.1 BGRA|VDB84301.1 CFRU|XP_031887105.1 CGLO|KAF3811205.1 CHIG|XP_018160444.1 CVIN|KAF4931173.1 CVYL|A04371 FGRM|XP_011327070.1 MGRA|XP_003855476.1 MLAR|XP_007407918.1 MORY|QBZ64770.1 NCRA|XP_961148.2 SSCL|APA15143.1

>Orthogroup2299: ANID|CBF70962.1 BCIN|XP_001553344.2 BGRA|VDB85714.1 CFRU|XP_031880664.1 CGLO|KAF3811181.1 CHIG|XP_018159504.1 CVIN|KAF4931293.1 CVYL|A04344 FGRM|XP_011323733.1 MGRA|XP_003855477.1 MLAR|XP_007409786.1 MORY|QBZ64676.1 NCRA|XP_960892.1 SSCL|APA15161.1

>Orthogroup2300: ANID|CBF70966.1 BCIN|XP_024553846.1 BGRA|VDB84340.1 CFRU|XP_031880733.1 CGLO|KAF3811164.1 CHIG|XP_018159484.1 CVIN|KAF4931226.1 CVYL|A04325 FGRM|XP_011324724.1 MGRA|XP_003848924.1 MLAR|XP_007416614.1 MORY|QBZ64692.1 NCRA|XP_961198.1 SSCL|APA15226.1

>Orthogroup2301: ANID|CBF70994.1 BCIN|XP_001560911.2 BGRA|VDB86204.1 CFRU|XP_031886860.1 CGLO|KAF3811406.1 CHIG|XP_018160644.1 CVIN|KAF4919717.1 CVYL|A04601 FGRM|XP_011327170.1 MGRA|XP_003855313.1 MLAR|XP_007409262.1 MORY|QBZ54276.1 NCRA|XP_961216.2 SSCL|APA08113.1

>Orthogroup2302: ANID|CBF71004.1 BCIN|XP_001555373.1 BGRA|VDB86210.1 CFRU|XP_031886995.1 CGLO|KAF3811410.1 CHIG|XP_018160649.1 CVIN|KAF4919711.1 CVYL|A04606 FGRM|XP_011327175.1 MGRA|XP_003849995.1 MLAR|XP_007404946.1 MORY|QBZ54268.1 NCRA|XP_011394607.1 SSCL|APA08118.1

>Orthogroup2303: ANID|CBF71006.1 BCIN|XP_001555374.1 BGRA|VDB86218.1 CFRU|XP_031886998.1 CGLO|KAF3811411.1 CHIG|XP_018160650.1 CVIN|KAF4919715.1 CVYL|A04607 FGRM|XP_011327176.1 MGRA|XP_003850113.1 MLAR|XP_007404972.1 MORY|QBZ54267.1 NCRA|XP_960596.1 SSCL|APA08120.1

>Orthogroup2304: ANID|CBF71015.1 BCIN|XP_001556976.1 BGRA|VDB87639.1 CFRU|XP_031885075.1 CGLO|KAF3800337.1 CHIG|XP_018162823.1 CVIN|KAF4918873.1 CVYL|A07353 FGRM|XP_011326417.1 MGRA|XP_003850163.1 MLAR|XP_007417563.1 MORY|QBZ63235.1 NCRA|XP_959056.1 SSCL|APA10576.1

>Orthogroup2305: ANID|CBF71017.1 BCIN|XP_001556978.1 BGRA|VDB87641.1 CFRU|XP_031885189.1 CGLO|KAF3800335.1 CHIG|XP_018162821.1 CVIN|KAF4918871.1 CVYL|A07331 FGRM|XP_011326419.1 MGRA|XP_003850023.1 MLAR|XP_007403570.1 MORY|QBZ63233.1 NCRA|XP_959058.1 SSCL|APA10578.1

>Orthogroup2306: ANID|CBF71040.1 BCIN|XP_001551858.1 BGRA|VDB84334.1 CFRU|XP_031880729.1 CGLO|KAF3811161.1 CHIG|XP_018159480.1 CVIN|KAF4931268.1 CVYL|A04321 FGRM|XP_011324726.1 MGRA|XP_003855155.1 MLAR|XP_007405419.1 MORY|QBZ64689.1 NCRA|XP_961196.1 SSCL|APA15229.1

>Orthogroup2308: ANID|CBF71062.1 BCIN|XP_001551897.1 BGRA|VDB83929.1 CFRU|XP_031886924.1 CGLO|KAF3811256.1 CHIG|XP_018160477.1 CVIN|KAF4931276.1 CVYL|A04407 FGRM|XP_011323640.1 MGRA|XP_003850014.1 MLAR|XP_007417975.1 MORY|QBZ63386.1 NCRA|XP_961547.1 SSCL|APA15206.1

>Orthogroup2309: ANID|CBF71064.1 BCIN|XP_024554012.1 BGRA|VDB84438.1 CFRU|XP_031877417.1 CGLO|KAF3805319.1 CHIG|XP_018162032.1 CVIN|KAF4931535.1 CVYL|A11738 FGRM|XP_011324107.1 MGRA|XP_003855223.1 MLAR|XP_007416494.1 MORY|QBZ62966.1 NCRA|XP_957751.1 SSCL|APA15373.1

>Orthogroup2310: ANID|CBF71068.1 BCIN|XP_001550713.1 BGRA|VDB84317.1 CFRU|XP_031886361.1 CGLO|KAF3806397.1 CHIG|XP_018162273.1 CVIN|KAF4923252.1 CVYL|A11987 FGRM|XP_011326569.1 MGRA|XP_003848914.1 MLAR|XP_007406277.1 MORY|QBZ59297.1 NCRA|XP_959021.1 SSCL|APA15319.1

>Orthogroup2312: ANID|CBF71085.1 BCIN|XP_001553377.1 BGRA|VDB85779.1 CFRU|XP_031880661.1 CGLO|KAF3811176.1 CHIG|XP_018159500.1 CVIN|KAF4931291.1 CVYL|A04338 FGRM|XP_011323730.1 MGRA|XP_003848908.1 MLAR|XP_007414031.1 MORY|QBZ64685.1 NCRA|XP_011394560.1 SSCL|APA15146.1

>Orthogroup2313: ANID|CBF71094.1 BCIN|XP_024553963.1 BGRA|VDB84267.1 CFRU|XP_031887140.1 CGLO|KAF3811355.1 CHIG|XP_018160578.1 CVIN|KAF4921044.1 CVYL|A04544 FGRM|XP_011326876.1 MGRA|XP_003855208.1 MLAR|XP_007417162.1 MORY|QBZ62512.1 NCRA|XP_011394572.1 SSCL|APA15426.1

>Orthogroup2314: ANID|CBF71096.1 BCIN|XP_001553326.1 BGRA|VDB85757.1 CFRU|XP_031880757.1 CGLO|KAF3811173.1 CHIG|XP_018159497.1 CVIN|KAF4931286.1 CVYL|A04336 FGRM|XP_011323701.1 MGRA|XP_003849060.1 MLAR|XP_007404405.1 MORY|QBZ64661.1 NCRA|XP_961306.1 SSCL|APA15175.1

>Orthogroup2315: ANID|CBF71100.1 BCIN|XP_024554049.1 BGRA|VDB84315.1 CFRU|XP_031887011.1 CGLO|KAF3811229.1 CHIG|XP_018160414.1 CVIN|KAF4931177.1 CVYL|A04399 FGRM|XP_011323652.1 MGRA|XP_003849173.1 MLAR|XP_007416063.1 MORY|QBZ64664.1 NCRA|XP_961207.1 SSCL|APA15356.1

>Orthogroup2317: ANID|CBF71108.1 BCIN|XP_024554060.1 BGRA|VDB84245.1 CFRU|XP_031880739.1 CGLO|KAF3811172.1 CHIG|XP_018159495.1 CVIN|KAF4931235.1 CVYL|A04334 FGRM|XP_011323699.1 MGRA|XP_003848770.1 MLAR|XP_007415909.1 MORY|QBZ64663.1 NCRA|XP_960741.3 SSCL|APA15361.1

>Orthogroup2318: ANID|CBF71127.1 BCIN|XP_001551695.1 BGRA|VDB88170.1 CFRU|XP_031892496.1 CGLO|KAF3797196.1 CHIG|XP_018157322.1 CVIN|KAF4920801.1 CVYL|A10503 FGRM|XP_011319938.1 MGRA|XP_003853330.1 MLAR|XP_007409512.1 MORY|QBZ66117.1 NCRA|XP_001728457.1 SSCL|APA11605.1

>Orthogroup2319: ANID|CBF71129.1 BCIN|XP_024550988.1 BGRA|VDB88171.1 CFRU|XP_031892760.1 CGLO|KAF3797197.1 CHIG|XP_018157321.1 CVIN|KAF4920826.1 CVYL|A10502 FGRM|XP_011319939.1 MGRA|XP_003853079.1 MLAR|XP_007413760.1 MORY|QBZ66116.1 NCRA|XP_965764.1 SSCL|APA11606.1

>Orthogroup2320: ANID|CBF71131.1 BCIN|XP_024550858.1 BGRA|VDB88160.1 CFRU|XP_031888175.1 CGLO|KAF3802122.1 CHIG|XP_018154269.1 CVIN|KAF4912413.1 CVYL|A03272 FGRM|XP_011320191.1 MGRA|XP_003856377.1 MLAR|XP_007411500.1 MORY|QBZ66123.1 NCRA|XP_965774.1 SSCL|APA11784.1

>Orthogroup2321: ANID|CBF71152.1 BCIN|XP_001550456.1 BGRA|VDB88079.1 CFRU|XP_031877703.1 CGLO|KAF3797372.1 CHIG|XP_018157138.1 CVIN|KAF4929370.1 CVYL|A10317 FGRM|XP_011319861.1 MGRA|XP_003857450.1 MLAR|XP_007406211.1 MORY|QBZ65906.1 NCRA|XP_965820.1 SSCL|APA11668.1

>Orthogroup2322: ANID|CBF71176.1 BCIN|XP_001545837.2 BGRA|VDB89801.1 CFRU|XP_031881715.1 CGLO|KAF3805369.1 CHIG|XP_018156739.1 CVIN|KAF4907237.1 CVYL|A11019 FGRM|XP_011320226.1 MGRA|XP_003852071.1 MLAR|XP_007416316.1 MORY|QBZ66248.1 NCRA|XP_958018.1 SSCL|APA11501.1

>Orthogroup2324: ANID|CBF71232.1 BCIN|XP_024549427.1 BGRA|VDB90972.1 CFRU|XP_031877838.1 CGLO|KAF3806631.1 CHIG|XP_018164230.1 CVIN|KAF4918915.1 CVYL|A12343 FGRM|XP_011326740.1 MGRA|XP_003852242.1 MLAR|XP_007406885.1 MORY|QBZ62979.1 NCRA|XP_964041.3 SSCL|APA09094.1

>Orthogroup2325: ANID|CBF71234.1 BCIN|XP_001559028.1 BGRA|VDB90968.1 CFRU|XP_031877840.1 CGLO|KAF3806629.1 CHIG|XP_018164232.1 CVIN|KAF4918917.1 CVYL|A12341 FGRM|XP_011326742.1 MGRA|XP_003852384.1 MLAR|XP_007412134.1 MORY|QBZ62981.1 NCRA|XP_964043.1 SSCL|APA09096.1

>Orthogroup2326: ANID|CBF71237.1 BCIN|XP_024549423.1 BGRA|VDB90952.1 CFRU|XP_031877872.1 CGLO|KAF3806628.1 CHIG|XP_018164233.1 CVIN|KAF4918911.1 CVYL|A12340 FGRM|XP_011326743.1 MGRA|XP_003853288.1 MLAR|XP_007404416.1 MORY|QBZ62982.1 NCRA|XP_964044.1 SSCL|APA09089.1

>Orthogroup2329: ANID|CBF71262.1 BCIN|XP_024549790.1 BGRA|VDB83665.1 CFRU|XP_031887781.1 CGLO|KAF3803233.1 CHIG|XP_018161081.1 CVIN|KAF4928678.1 CVYL|A03665 FGRM|XP_011323844.1 MGRA|XP_003855486.1 MLAR|XP_007411605.1 MORY|QBZ64592.1 NCRA|XP_962471.3 SSCL|APA05739.1

>Orthogroup2330: ANID|CBF71264.1 BCIN|XP_024549631.1 BGRA|VDB83690.1 CFRU|XP_031880526.1 CGLO|KAF3811505.1 CHIG|XP_018160761.1 CVIN|KAF4926804.1 CVYL|A04712 FGRM|XP_011317803.1 MGRA|XP_003849165.1 MLAR|XP_007413460.1 MORY|QBZ58782.1 NCRA|XP_961757.2 SSCL|APA05752.1

>Orthogroup2331: ANID|CBF71275.1 BCIN|XP_001558329.1 BGRA|VDB83656.1 CFRU|XP_031876556.1 CGLO|KAF3811481.1 CHIG|XP_018160734.1 CVIN|KAF4907412.1 CVYL|A04685 FGRM|XP_011317777.1 MGRA|XP_003849069.1 MLAR|XP_007404168.1 MORY|QBZ56559.1 NCRA|XP_962674.2 SSCL|APA13708.1

>Orthogroup2332: ANID|CBF71277.1 BCIN|XP_024552251.1 BGRA|VDB92683.1 CFRU|XP_031878872.1 CGLO|KAF3802609.1 CHIG|XP_018161305.1 CVIN|KAF4911615.1 CVYL|A01897 FGRM|XP_011321448.1 MGRA|XP_003855466.1 MLAR|XP_007406896.1 MORY|QBZ65041.1 NCRA|XP_962438.1 SSCL|APA13276.1

>Orthogroup2333: ANID|CBF71281.1 BCIN|XP_001558349.1 BGRA|VDB83660.1 CFRU|XP_031876534.1 CGLO|KAF3811485.1 CHIG|XP_018160738.1 CVIN|KAF4926793.1 CVYL|A04690 FGRM|XP_011317781.1 MGRA|XP_003848837.1 MLAR|XP_007410845.1 MORY|QBZ56702.1 NCRA|XP_962691.1 SSCL|APA13726.1

>Orthogroup2335: ANID|CBF71296.1 BCIN|XP_024552343.1 BGRA|VDB92688.1 CFRU|XP_031881053.1 CGLO|KAF3809842.1 CHIG|XP_018161424.1 CVIN|KAF4930605.1 CVYL|A01772 FGRM|XP_011321461.1 MGRA|XP_003848882.1 MLAR|XP_007418555.1 MORY|QBZ65215.1 NCRA|XP_962785.3 SSCL|APA13170.1

>Orthogroup2336: ANID|CBF71323.1 BCIN|XP_024548429.1 BGRA|VDB88819.1 CFRU|XP_031881902.1 CGLO|KAF3800681.1 CHIG|XP_018152001.1 CVIN|KAF4922035.1 CVYL|A13035 FGRM|XP_011321268.1 MGRA|XP_003856148.1 MLAR|XP_007406843.1 MORY|QBZ61269.1 NCRA|XP_958926.1 SSCL|APA12252.1

>Orthogroup2337: ANID|CBF71354.1 BCIN|XP_024549850.1 BGRA|VCU41011.1 CFRU|XP_031876723.1 CGLO|KAF3798690.1 CHIG|XP_018157467.1 CVIN|KAF4918468.1 CVYL|A09428 FGRM|XP_011327910.1 MGRA|XP_003852619.1 MLAR|XP_007407416.1 MORY|QBZ56249.1 NCRA|XP_962389.1 SSCL|APA05516.1

>Orthogroup2341: ANID|CBF71539.1 BCIN|XP_024550144.1 BGRA|VDB94967.1 CFRU|XP_031882157.1 CGLO|KAF3804460.1 CHIG|XP_018160289.1 CVIN|KAF4930944.1 CVYL|A04173 FGRM|XP_011326709.1 MGRA|XP_003855297.1 MLAR|XP_007411433.1 MORY|QBZ62529.1 NCRA|XP_961325.2 SSCL|APA10772.1

>Orthogroup2342: ANID|CBF71541.1 BCIN|XP_001554657.1 BGRA|VDB94755.1 CFRU|XP_031891500.1 CGLO|KAF3804532.1 CHIG|XP_018159459.1 CVIN|KAF4924544.1 CVYL|A04249 FGRM|XP_011328802.1 MGRA|XP_003855073.1 MLAR|XP_007404597.1 MORY|QBZ64726.1 NCRA|XP_955848.1 SSCL|APA11034.1

>Orthogroup2343: ANID|CBF71572.1 BCIN|XP_001554654.1 BGRA|VDB94783.1 CFRU|XP_031891493.1 CGLO|KAF3804535.1 CHIG|XP_018159455.1 CVIN|KAF4924545.1 CVYL|A04252 FGRM|XP_011328800.1 MGRA|XP_003854887.1 MLAR|XP_007414969.1 MORY|QBZ64729.1 NCRA|XP_956750.2 SSCL|APA11036.1

>Orthogroup2345: ANID|CBF71595.1 BCIN|XP_001552517.1 BGRA|VDB94874.1 CFRU|XP_031882195.1 CGLO|KAF3804405.1 CHIG|XP_018160220.1 CVIN|KAF4930897.1 CVYL|A04104 FGRM|XP_011324496.1 MGRA|XP_003855193.1 MLAR|XP_007406301.1 MORY|QBZ62538.1 NCRA|XP_961323.1 SSCL|APA10786.1

>Orthogroup2346: ANID|CBF71599.1 BCIN|XP_024550136.1 BGRA|VDB94965.1 CFRU|XP_031882181.1 CGLO|KAF3804459.1 CHIG|XP_018160288.1 CVIN|KAF4930961.1 CVYL|A04172 FGRM|XP_011326708.1 MGRA|XP_003855270.1 MLAR|XP_007409493.1 MORY|QBZ62539.1 NCRA|XP_961324.3 SSCL|APA10785.1

>Orthogroup2347: ANID|CBF71619.1 BCIN|XP_001552515.2 BGRA|VDB94983.1 CFRU|XP_031891204.1 CGLO|KAF3804524.1 CHIG|XP_018159465.1 CVIN|KAF4924547.1 CVYL|A04244 FGRM|XP_011324720.1 MGRA|XP_003855556.1 MLAR|XP_007411118.1 MORY|QBZ64706.1 NCRA|XP_956745.1 SSCL|APA10784.1

>Orthogroup2348: ANID|CBF71622.1 BCIN|XP_001553879.2 BGRA|VCU39260.1 CFRU|XP_031885866.1 CGLO|KAF3806594.1 CHIG|XP_018157634.1 CVIN|KAF4916895.1 CVYL|A09273 FGRM|XP_011328108.1 MGRA|XP_003849472.1 MLAR|XP_007406326.1 MORY|QBZ56549.1 NCRA|XP_961975.1 SSCL|APA13063.1

>Orthogroup2349: ANID|CBF71624.1 BCIN|XP_024552286.1 BGRA|VCU39261.1 CFRU|XP_031885867.1 CGLO|KAF3806595.1 CHIG|XP_018157635.1 CVIN|KAF4916892.1 CVYL|A09272 FGRM|XP_011328107.1 MGRA|XP_003849642.1 MLAR|XP_007403859.1 MORY|QBZ56550.1 NCRA|XP_961974.2 SSCL|APA13062.1

>Orthogroup2351: ANID|CBF71650.1 BCIN|XP_001555261.1 BGRA|VDB87814.1 CFRU|XP_031883661.1 CGLO|KAF3800251.1 CHIG|XP_018155058.1 CVIN|KAF4921768.1 CVYL|A12392 FGRM|XP_011317542.1 MGRA|XP_003849653.1 MLAR|XP_007414341.1 MORY|QBZ53763.1 NCRA|XP_011393677.1 SSCL|APA08657.1

>Orthogroup2352: ANID|CBF71677.1 BCIN|XP_024550590.1 BGRA|VDB94955.1 CFRU|XP_031877225.1 CGLO|KAF3806459.1 CHIG|XP_018162347.1 CVIN|KAF4897891.1 CVYL|A12058 FGRM|XP_011323934.1 MGRA|XP_003855296.1 MLAR|XP_007415656.1 MORY|QBZ61395.1 NCRA|XP_956404.1 SSCL|APA11396.1

>Orthogroup2353: ANID|CBF71685.1 BCIN|XP_001559329.1 BGRA|VDB94846.1 CFRU|XP_031891174.1 CGLO|KAF3804674.1 CHIG|XP_018161742.1 CVIN|KAF4902798.1 CVYL|A05210 FGRM|XP_011321523.1 MGRA|XP_003854826.1 MLAR|XP_007408984.1 MORY|QBZ59576.1 NCRA|XP_956700.1 SSCL|APA07867.1

>Orthogroup2354: ANID|CBF71690.1 BCIN|XP_001552990.1 BGRA|VDB86345.1 CFRU|XP_031886311.1 CGLO|KAF3806202.1 CHIG|XP_018162191.1 CVIN|KAF4931495.1 CVYL|A11788 FGRM|XP_011326337.1 MGRA|XP_003855291.1 MLAR|XP_007409397.1 MORY|QBZ55786.1 NCRA|XP_959063.1 SSCL|APA11218.1

>Orthogroup2355: ANID|CBF71694.1 BCIN|XP_001552993.1 BGRA|VDB94550.1 CFRU|XP_031886308.1 CGLO|KAF3806205.1 CHIG|XP_018162194.1 CVIN|KAF4931561.1 CVYL|A11792 FGRM|XP_011326341.1 MGRA|XP_003854892.1 MLAR|XP_007417830.1 MORY|QBZ55789.1 NCRA|XP_959060.1 SSCL|APA11221.1

>Orthogroup2356: ANID|CBF71706.1 BCIN|XP_001554662.1 BGRA|VDB94760.1 CFRU|XP_031891276.1 CGLO|KAF3811128.1 CHIG|XP_018160374.1 CVIN|KAF4924505.1 CVYL|A04284 FGRM|XP_011327073.1 MGRA|XP_003854496.1 MLAR|XP_007413128.1 MORY|QBZ63424.1 NCRA|XP_961311.1 SSCL|APA11029.1

>Orthogroup2358: ANID|CBF71716.1 BCIN|XP_024547537.1 BGRA|VDB94894.1 CFRU|XP_031879394.1 CGLO|KAF3808617.1 CHIG|XP_018161899.1 CVIN|KAF4926141.1 CVYL|A12299 FGRM|XP_011318503.1 MGRA|XP_003855617.1 MLAR|XP_007404251.1 MORY|QBZ59639.1 NCRA|XP_962872.3 SSCL|APA07872.1

>Orthogroup2360: ANID|CBF71743.1 BCIN|XP_001554799.1 BGRA|VDB94718.1 CFRU|XP_031878004.1 CGLO|KAF3799591.1 CHIG|XP_018154586.1 CVIN|KAF4926460.1 CVYL|A13452 FGRM|XP_011325566.1 MGRA|XP_003849697.1 MLAR|XP_007410227.1 MORY|QBZ54422.1 NCRA|XP_956473.1 SSCL|APA15516.1

>Orthogroup2363: ANID|CBF73669.1 BCIN|XP_024553343.1 BGRA|VCU39325.1 CFRU|XP_031887118.1 CGLO|KAF3811309.1 CHIG|XP_018160533.1 CVIN|KAF4927200.1 CVYL|A04488 FGRM|XP_011326427.1 MGRA|XP_003850141.1 MLAR|XP_007405037.1 MORY|QBZ63404.1 NCRA|XP_961537.1 SSCL|APA13661.1

>Orthogroup2364: ANID|CBF73674.1 BCIN|XP_024553226.1 BGRA|VDB88685.1 CFRU|XP_031882268.1 CGLO|KAF3804450.1 CHIG|XP_018160276.1 CVIN|KAF4930915.1 CVYL|A04159 FGRM|XP_011324501.1 MGRA|XP_003850306.1 MLAR|XP_007411142.1 MORY|QBZ64717.1 NCRA|XP_011394595.1 SSCL|APA13501.1

>Orthogroup2365: ANID|CBF73680.1 BCIN|XP_024553224.1 BGRA|VCU40503.1 CFRU|XP_031882265.1 CGLO|KAF3804451.1 CHIG|XP_018160277.1 CVIN|KAF4930916.1 CVYL|A04160 FGRM|XP_011324500.1 MGRA|XP_003850102.1 MLAR|XP_007406962.1 MORY|QBZ64716.1 NCRA|XP_961132.2 SSCL|APA13502.1

>Orthogroup2366: ANID|CBF73699.1 BCIN|XP_024553293.1 BGRA|VCU40704.1 CFRU|XP_031880659.1 CGLO|KAF3811147.1 CHIG|XP_018160355.1 CVIN|KAF4931162.1 CVYL|A04306 FGRM|XP_011326476.1 MGRA|XP_003850006.1 MLAR|XP_007416527.1 MORY|QBZ57422.1 NCRA|XP_011394697.1 SSCL|APA13542.1

>Orthogroup2367: ANID|CBF73703.1 BCIN|XP_001558276.1 BGRA|VDB86093.1 CFRU|XP_031887013.1 CGLO|KAF3811313.1 CHIG|XP_018160538.1 CVIN|KAF4927239.1 CVYL|A04493 FGRM|XP_011326434.1 MGRA|XP_003850094.1 MLAR|XP_007405307.1 MORY|QBZ63398.1 NCRA|XP_961541.1 SSCL|APA13670.1

>Orthogroup2368: ANID|CBF73709.1 BCIN|XP_001553777.1 BGRA|VCU40638.1 CFRU|XP_031888652.1 CGLO|KAF3799323.1 CHIG|XP_018159919.1 CVIN|KAF4924763.1 CVYL|A04219 FGRM|XP_011324458.1 MGRA|XP_003850469.1 MLAR|XP_007416538.1 MORY|QBZ59184.1 NCRA|XP_961601.2 SSCL|APA14506.1

>Orthogroup2369: ANID|CBF73720.1 BCIN|XP_024548320.1 BGRA|VCU40555.1 CFRU|XP_031891530.1 CGLO|KAF3811121.1 CHIG|XP_018160382.1 CVIN|KAF4924540.1 CVYL|A04276 FGRM|XP_011326690.1 MGRA|XP_003850333.1 MLAR|XP_007417614.1 MORY|QBZ56912.1 NCRA|XP_961573.1 SSCL|APA12572.1

>Orthogroup2370: ANID|CBF73743.1 BCIN|XP_001558217.1 BGRA|VCU40478.1 CFRU|XP_031886689.1 CGLO|KAF3800271.1 CHIG|XP_018162758.1 CVIN|KAF4927612.1 CVYL|A06966 FGRM|XP_011327025.1 MGRA|XP_003850540.1 MLAR|XP_007413808.1 MORY|QBZ61333.1 NCRA|XP_957332.1 SSCL|APA13639.1

>Orthogroup2371: ANID|CBF73758.1 BCIN|XP_024549707.1 BGRA|VDB86364.1 CFRU|XP_031885923.1 CGLO|KAF3805529.1 CHIG|XP_018155369.1 CVIN|KAF4918286.1 CVYL|A05652 FGRM|XP_011317492.1 MGRA|XP_003849996.1 MLAR|XP_007415322.1 MORY|QBZ58967.1 NCRA|XP_963604.1 SSCL|APA05641.1

>Orthogroup2372: ANID|CBF73760.1 BCIN|XP_024553281.1 BGRA|VDB93963.1 CFRU|XP_031885371.1 CGLO|KAF3799546.1 CHIG|XP_018154537.1 CVIN|KAF4931861.1 CVYL|A13406 FGRM|XP_011324907.1 MGRA|XP_003855663.1 MLAR|XP_007418100.1 MORY|QBZ62096.1 NCRA|XP_956977.1 SSCL|APA13553.1

>Orthogroup2373: ANID|CBF73762.1 BCIN|XP_024553282.1 BGRA|VDB93965.1 CFRU|XP_031883437.1 CGLO|KAF3800599.1 CHIG|XP_018152079.1 CVIN|KAF4927121.1 CVYL|A13117 FGRM|XP_011321290.1 MGRA|XP_003855078.1 MLAR|XP_007413107.1 MORY|QBZ62104.1 NCRA|XP_956974.3 SSCL|APA13552.1

>Orthogroup2374: ANID|CBF73778.1 BCIN|XP_001550729.1 BGRA|VDB93606.1 CFRU|XP_031886341.1 CGLO|KAF3806404.1 CHIG|XP_018162288.1 CVIN|KAF4923228.1 CVYL|A11996 FGRM|XP_011326575.1 MGRA|XP_003849876.1 MLAR|XP_007404206.1 MORY|QBZ63903.1 NCRA|XP_956169.1 SSCL|APA15307.1

>Orthogroup2375: ANID|CBF73780.1 BCIN|XP_001550727.2 BGRA|VDB93608.1 CFRU|XP_031886340.1 CGLO|KAF3806403.1 CHIG|XP_018162287.1 CVIN|KAF4923278.1 CVYL|A11995 FGRM|XP_011326574.1 MGRA|XP_003849879.1 MLAR|XP_007416848.1 MORY|QBZ56510.1 NCRA|XP_958147.2 SSCL|APA15309.1

>Orthogroup2376: ANID|CBF73788.1 BCIN|XP_024546967.1 BGRA|VCU40454.1 CFRU|XP_031882270.1 CGLO|KAF3804453.1 CHIG|XP_018160280.1 CVIN|KAF4930911.1 CVYL|A04163 FGRM|XP_011324497.1 MGRA|XP_003850366.1 MLAR|XP_007415284.1 MORY|QBZ64712.1 NCRA|XP_961181.2 SSCL|APA05971.1

>Orthogroup2377: ANID|CBF73792.1 BCIN|XP_001558216.1 BGRA|VCU40488.1 CFRU|XP_031886687.1 CGLO|KAF3800270.1 CHIG|XP_018162756.1 CVIN|KAF4927610.1 CVYL|A06967 FGRM|XP_011327023.1 MGRA|XP_003849880.1 MLAR|XP_007411127.1 MORY|QBZ61334.1 NCRA|XP_011393917.1 SSCL|APA13638.1

>Orthogroup2378: ANID|CBF73799.1 BCIN|XP_001558235.1 BGRA|VCU40496.1 CFRU|XP_031886530.1 CGLO|KAF3800264.1 CHIG|XP_018162750.1 CVIN|KAF4927563.1 CVYL|A06973 FGRM|XP_011327029.1 MGRA|XP_003850286.1 MLAR|XP_007410303.1 MORY|QBZ61341.1 NCRA|XP_957343.1 SSCL|APA13648.1

>Orthogroup2379: ANID|CBF73806.1 BCIN|XP_024549691.1 BGRA|VDB93974.1 CFRU|XP_031879490.1 CGLO|KAF3799660.1 CHIG|XP_018154668.1 CVIN|KAF4922166.1 CVYL|A13522 FGRM|XP_011316687.1 MGRA|XP_003850091.1 MLAR|XP_007404090.1 MORY|QBZ60975.1 NCRA|XP_963164.1 SSCL|APA05622.1

>Orthogroup2380: ANID|CBF73811.1 BCIN|XP_001555927.1 BGRA|VDB93939.1 CFRU|XP_031875824.1 CGLO|KAF3805549.1 CHIG|XP_018155403.1 CVIN|KAF4890967.1 CVYL|A05672 FGRM|XP_011324646.1 MGRA|XP_003849893.1 MLAR|XP_007408529.1 MORY|QBZ54877.1 NCRA|XP_963397.1 SSCL|APA06037.1

>Orthogroup2381: ANID|CBF73824.1 BCIN|XP_001558717.1 BGRA|VDB93976.1 CFRU|XP_031885925.1 CGLO|KAF3805527.1 CHIG|XP_018155371.1 CVIN|KAF4918291.1 CVYL|A05650 FGRM|XP_011317490.1 MGRA|XP_003849885.1 MLAR|XP_007408266.1 MORY|QBZ58969.1 NCRA|XP_963602.2 SSCL|APA05639.1

>Orthogroup2382: ANID|CBF73828.1 BCIN|XP_001552180.1 BGRA|VDB93940.1 CFRU|XP_031878032.1 CGLO|KAF3799565.1 CHIG|XP_018154567.1 CVIN|KAF4931854.1 CVYL|A13424 FGRM|XP_011324891.1 MGRA|XP_003850535.1 MLAR|XP_007416638.1 MORY|QBZ56059.1 NCRA|XP_965642.2 SSCL|APA05592.1

>Orthogroup2383: ANID|CBF73928.1 BCIN|XP_024546926.1 BGRA|VCU40680.1 CFRU|XP_031887003.1 CGLO|KAF3811226.1 CHIG|XP_018160419.1 CVIN|KAF4931210.1 CVYL|A04394 FGRM|XP_011328292.1 MGRA|XP_003850103.1 MLAR|XP_007412569.1 MORY|QBZ62662.1 NCRA|XP_962034.1 SSCL|APA05813.1

>Orthogroup2386: ANID|CBF74054.1 BCIN|XP_024553317.1 BGRA|VCU40483.1 CFRU|XP_031886887.1 CGLO|KAF3811323.1 CHIG|XP_018160549.1 CVIN|KAF4927251.1 CVYL|A04504 FGRM|XP_011327145.1 MGRA|XP_003849931.1 MLAR|XP_007409296.1 MORY|QBZ58034.1 NCRA|XP_961184.1 SSCL|APA13627.1

>Orthogroup2387: ANID|CBF74056.1 BCIN|XP_024553318.1 BGRA|VCU40482.1 CFRU|XP_031886884.1 CGLO|KAF3811324.1 CHIG|XP_018160550.1 CVIN|KAF4927250.1 CVYL|A04505 FGRM|XP_011327144.1 MGRA|XP_003850483.1 MLAR|XP_007409918.1 MORY|QBZ58033.1 NCRA|XP_961183.1 SSCL|APA13628.1

>Orthogroup2388: ANID|CBF74066.1 BCIN|XP_001558247.1 BGRA|VCU39320.1 CFRU|XP_031880720.1 CGLO|KAF3811142.1 CHIG|XP_018160360.1 CVIN|KAF4931260.1 CVYL|A04301 FGRM|XP_011326450.1 MGRA|XP_003850011.1 MLAR|XP_007415468.1 MORY|QBZ57461.1 NCRA|XP_960994.2 SSCL|APA13658.1

>Orthogroup2389: ANID|CBF74071.1 BCIN|XP_001558262.1 BGRA|VCU39326.1 CFRU|XP_031880716.1 CGLO|KAF3811146.1 CHIG|XP_018160356.1 CVIN|KAF4931192.1 CVYL|A04305 FGRM|XP_011326444.1 MGRA|XP_003850096.1 MLAR|XP_007410693.1 MORY|QBZ57425.1 NCRA|XP_011394694.1 SSCL|APA13662.1

>Orthogroup2391: ANID|CBF74098.1 BCIN|XP_001558214.1 BGRA|VCU40486.1 CFRU|XP_031886691.1 CGLO|KAF3800268.1 CHIG|XP_018162754.1 CVIN|KAF4927608.1 CVYL|A06969 FGRM|XP_011327021.1 MGRA|XP_003850374.1 MLAR|XP_007409091.1 MORY|QBZ61337.1 NCRA|XP_957329.1 SSCL|APA13636.1

>Orthogroup2392: ANID|CBF74121.1 BCIN|XP_024547045.1 BGRA|VCU40629.1 CFRU|XP_031888954.1 CGLO|KAF3799203.1 CHIG|XP_018159805.1 CVIN|KAF4894527.1 CVYL|A10098 FGRM|XP_011328840.1 MGRA|XP_003849884.1 MLAR|XP_007413523.1 MORY|QBZ59130.1 NCRA|XP_958694.1 SSCL|APA05233.1

>Orthogroup2393: ANID|CBF74131.1 BCIN|XP_001558945.1 BGRA|VCU40679.1 CFRU|XP_031886832.1 CGLO|KAF3811225.1 CHIG|XP_018160420.1 CVIN|KAF4931209.1 CVYL|A04393 FGRM|XP_011326832.1 MGRA|XP_003850046.1 MLAR|XP_007409950.1 MORY|QBZ57030.1 NCRA|XP_958762.1 SSCL|APA05815.1

>Orthogroup2394: ANID|CBF74133.1 BCIN|XP_001555871.2 BGRA|VCU40188.1 CFRU|XP_031882135.1 CGLO|KAF3804343.1 CHIG|XP_018160128.1 CVIN|KAF4918342.1 CVYL|A04032 FGRM|XP_011324773.1 MGRA|XP_003850248.1 MLAR|XP_007416254.1 MORY|QBZ58686.1 NCRA|XP_958189.1 SSCL|APA06067.1

>Orthogroup2395: ANID|CBF74149.1 BCIN|XP_001546976.1 BGRA|VCU39408.1 CFRU|XP_031888684.1 CGLO|KAF3799281.1 CHIG|XP_018159878.1 CVIN|KAF4924819.1 CVYL|A03897 FGRM|XP_011324542.1 MGRA|XP_003849921.1 MLAR|XP_007407249.1 MORY|QBZ58665.1 NCRA|XP_958831.1 SSCL|APA12562.1

>Orthogroup2396: ANID|CBF74156.1 BCIN|XP_001553770.1 BGRA|VCU40649.1 CFRU|XP_031888792.1 CGLO|KAF3799385.1 CHIG|XP_018159957.1 CVIN|KAF4906791.1 CVYL|A03463 FGRM|XP_011324466.1 MGRA|XP_003850203.1 MLAR|XP_007417226.1 MORY|QBZ59178.1 NCRA|XP_961712.1 SSCL|APA14515.1

>Orthogroup2397: ANID|CBF74163.1 BCIN|XP_024547001.1 BGRA|VCU41288.1 CFRU|XP_031888725.1 CGLO|KAF3799194.1 CHIG|XP_018159802.1 CVIN|KAF4914912.1 CVYL|A10089 FGRM|XP_011328763.1 MGRA|XP_003850193.1 MLAR|XP_007404084.1 MORY|QBZ63735.1 NCRA|XP_961695.1 SSCL|APA05871.1

>Orthogroup2398: ANID|CBF74165.1 BCIN|XP_001553784.1 BGRA|VCU40628.1 CFRU|XP_031888646.1 CGLO|KAF3799328.1 CHIG|XP_018159925.1 CVIN|KAF4924811.1 CVYL|A04264 FGRM|XP_011328791.1 MGRA|XP_003850256.1 MLAR|XP_007406886.1 MORY|QBZ59173.1 NCRA|XP_958550.1 SSCL|APA05234.1

>Orthogroup2399: ANID|CBF74173.1 BCIN|XP_024546876.1 BGRA|VCU39337.1 CFRU|XP_031888707.1 CGLO|KAF3804180.1 CHIG|XP_018160026.1 CVIN|KAF4918408.1 CVYL|A03855 FGRM|XP_011324767.1 MGRA|XP_003850077.1 MLAR|XP_007406873.1 MORY|QBZ63393.1 NCRA|XP_961158.1 SSCL|APA05930.1

>Orthogroup2400: ANID|CBF74199.1 BCIN|XP_024548337.1 BGRA|VCU39780.1 CFRU|XP_031888669.1 CGLO|KAF3799307.1 CHIG|XP_018159907.1 CVIN|KAF4924796.1 CVYL|A04053 FGRM|XP_011328849.1 MGRA|XP_003850158.1 MLAR|XP_007406455.1 MORY|QBZ58687.1 NCRA|XP_960643.1 SSCL|APA12591.1

>Orthogroup2401: ANID|CBF74201.1 BCIN|XP_001546024.1 BGRA|VCU39779.1 CFRU|XP_031888963.1 CGLO|KAF3799261.1 CHIG|XP_018159896.1 CVIN|KAF4908832.1 CVYL|A10153 FGRM|XP_011324737.1 MGRA|XP_003850245.1 MLAR|XP_007405933.1 MORY|QBZ58673.1 NCRA|XP_958086.1 SSCL|APA12590.1

>Orthogroup2402: ANID|CBF74215.1 BCIN|XP_001553718.1 BGRA|VCU39782.1 CFRU|XP_031889041.1 CGLO|KAF3799189.1 CHIG|XP_018159797.1 CVIN|KAF4914920.1 CVYL|A10084 FGRM|XP_011328761.1 MGRA|XP_003850133.1 MLAR|XP_007408798.1 MORY|QBZ54546.1 NCRA|XP_957976.3 SSCL|APA13498.1

>Orthogroup2403: ANID|CBF74219.1 BCIN|XP_024546832.1 BGRA|VCU40571.1 CFRU|XP_031882260.1 CGLO|KAF3804314.1 CHIG|XP_018160107.1 CVIN|KAF4920098.1 CVYL|A04001 FGRM|XP_011324565.1 MGRA|XP_003850323.1 MLAR|XP_007410350.1 MORY|QBZ54771.1 NCRA|XP_961022.1 SSCL|APA06050.1

>Orthogroup2406: ANID|CBF74230.1 BCIN|XP_001555896.1 BGRA|VCU40598.1 CFRU|XP_031882271.1 CGLO|KAF3804318.1 CHIG|XP_018160110.1 CVIN|KAF4920125.1 CVYL|A04010 FGRM|XP_011324563.1 MGRA|XP_003850322.1 MLAR|XP_007413551.1 MORY|QBZ54769.1 NCRA|XP_961025.2 SSCL|APA06047.1

>Orthogroup2408: ANID|CBF74254.1 BCIN|XP_024546960.1 BGRA|VDB93869.1 CFRU|XP_031881840.1 CGLO|KAF3800617.1 CHIG|XP_018152062.1 CVIN|KAF4918179.1 CVYL|A13100 FGRM|XP_011316337.1 MGRA|XP_003848923.1 MLAR|XP_007408032.1 MORY|QBZ60996.1 NCRA|XP_956123.1 SSCL|APA05989.1

>Orthogroup2409: ANID|CBF74260.1 BCIN|XP_024546969.1 BGRA|VDB93926.1 CFRU|XP_031881777.1 CGLO|KAF3800642.1 CHIG|XP_018152042.1 CVIN|KAF4922013.1 CVYL|A13074 FGRM|XP_011317224.1 MGRA|XP_003855670.1 MLAR|XP_007416293.1 MORY|QBZ57314.1 NCRA|XP_956898.1 SSCL|APA05967.1

>Orthogroup2410: ANID|CBF74269.1 BCIN|XP_024548110.1 BGRA|VDB93922.1 CFRU|XP_031881828.1 CGLO|KAF3800629.1 CHIG|XP_018152055.1 CVIN|KAF4918201.1 CVYL|A13087 FGRM|XP_011317221.1 MGRA|XP_003855481.1 MLAR|XP_007409980.1 MORY|QBZ57306.1 NCRA|XP_956276.2 SSCL|APA12618.1

>Orthogroup2411: ANID|CBF74276.1 BCIN|XP_001559875.1 BGRA|VCU40019.1 CFRU|XP_031887071.1 CGLO|KAF3811389.1 CHIG|XP_018160626.1 CVIN|KAF4919706.1 CVYL|A04581 FGRM|XP_011327183.1 MGRA|XP_003850690.1 MLAR|XP_007414792.1 MORY|QBZ64711.1 NCRA|XP_960733.2 SSCL|APA09729.1

>Orthogroup2412: ANID|CBF74278.1 BCIN|XP_001559911.1 BGRA|VDB89283.1 CFRU|XP_031886932.1 CGLO|KAF3811387.1 CHIG|XP_018160623.1 CVIN|KAF4919675.1 CVYL|A04579 FGRM|XP_011327193.1 MGRA|XP_003851172.1 MLAR|XP_007404399.1 MORY|QBZ58058.1 NCRA|XP_960739.1 SSCL|APA09751.1

>Orthogroup2413: ANID|CBF74279.1 BCIN|XP_024546107.1 BGRA|VDB90709.1 CFRU|XP_031888320.1 CGLO|KAF3803581.1 CHIG|XP_018163890.1 CVIN|KAF4920345.1 CVYL|A14514 FGRM|XP_011316755.1 MGRA|XP_003850704.1 MLAR|XP_007415245.1 MORY|QBZ55312.1 NCRA|XP_964051.2 SSCL|APA06630.1

>Orthogroup2414: ANID|CBF74285.1 BCIN|XP_024546353.1 BGRA|VDB92467.1 CFRU|XP_031890503.1 CGLO|KAF3804828.1 CHIG|XP_018164312.1 CVIN|KAF4929775.1 CVYL|A12246 FGRM|XP_011317018.1 MGRA|XP_003851068.1 MLAR|XP_007409412.1 MORY|QBZ63552.1 NCRA|XP_964475.2 SSCL|APA06871.1

>Orthogroup2415: ANID|CBF74295.1 BCIN|XP_024549364.1 BGRA|VCU38885.1 CFRU|XP_031887519.1 CGLO|KAF3803207.1 CHIG|XP_018161041.1 CVIN|KAF4928660.1 CVYL|A03636 FGRM|XP_011323573.1 MGRA|XP_003854621.1 MLAR|XP_007404856.1 MORY|QBZ58751.1 NCRA|XP_961832.2 SSCL|APA15769.1

>Orthogroup2416: ANID|CBF74317.1 BCIN|XP_024546892.1 BGRA|VDB93915.1 CFRU|XP_031878068.1 CGLO|KAF3799570.1 CHIG|XP_018154562.1 CVIN|KAF4926515.1 CVYL|A13429 FGRM|XP_011323706.1 MGRA|XP_003855014.1 MLAR|XP_007406587.1 MORY|QBZ61080.1 NCRA|XP_957188.3 SSCL|APA05848.1

>Orthogroup2417: ANID|CBF74319.1 BCIN|XP_024553275.1 BGRA|VDB93947.1 CFRU|XP_031883378.1 CGLO|KAF3800594.1 CHIG|XP_018152084.1 CVIN|KAF4927110.1 CVYL|A13121 FGRM|XP_011321295.1 MGRA|XP_003854860.1 MLAR|XP_007404721.1 MORY|QBZ61048.1 NCRA|XP_963444.1 SSCL|APA13562.1

>Orthogroup2418: ANID|CBF74323.1 BCIN|XP_024546885.1 BGRA|VDB93887.1 CFRU|XP_031878065.1 CGLO|KAF3799574.1 CHIG|XP_018154557.1 CVIN|KAF4926469.1 CVYL|A13434 FGRM|XP_011323711.1 MGRA|XP_003855662.1 MLAR|XP_007403959.1 MORY|QBZ61076.1 NCRA|XP_957183.2 SSCL|APA05854.1

>Orthogroup2419: ANID|CBF74326.1 BCIN|XP_024553370.1 BGRA|VDB93729.1 CFRU|XP_031882644.1 CGLO|KAF3799392.1 CHIG|XP_018152260.1 CVIN|KAF4899301.1 CVYL|A13254 FGRM|XP_011324621.1 MGRA|XP_003854852.1 MLAR|XP_007408714.1 MORY|QBZ53997.1 NCRA|XP_956615.2 SSCL|APA13700.1

>Orthogroup2420: ANID|CBF74332.1 BCIN|XP_001558620.1 BGRA|VDB93862.1 CFRU|XP_031879449.1 CGLO|KAF3799649.1 CHIG|XP_018154659.1 CVIN|KAF4922197.1 CVYL|A13512 FGRM|XP_011315956.1 MGRA|XP_003855654.1 MLAR|XP_007412189.1 MORY|QBZ60970.1 NCRA|XP_956119.2 SSCL|APA05681.1

>Orthogroup2421: ANID|CBF74349.1 BCIN|XP_024549490.1 BGRA|VCU39997.1 CFRU|XP_031882248.1 CGLO|KAF3804443.1 CHIG|XP_018160266.1 CVIN|KAF4930968.1 CVYL|A04151 FGRM|XP_011324514.1 MGRA|XP_003856648.1 MLAR|XP_007414682.1 MORY|QBZ54427.1 NCRA|XP_961376.2 SSCL|APA09154.1

>Orthogroup2422: ANID|CBF74360.1 BCIN|XP_024546698.1 BGRA|VDB90767.1 CFRU|XP_031882844.1 CGLO|KAF3803439.1 CHIG|XP_018164112.1 CVIN|KAF4926338.1 CVYL|A14368 FGRM|XP_011326818.1 MGRA|XP_003850722.1 MLAR|XP_007409668.1 MORY|QBZ56469.1 NCRA|XP_963785.3 SSCL|APA06552.1

>Orthogroup2425: ANID|CBF74417.1 BCIN|XP_024551917.1 BGRA|VDB89449.1 CFRU|XP_031882722.1 CGLO|KAF3803390.1 CHIG|XP_018163941.1 CVIN|KAF4918452.1 CVYL|A14320 FGRM|XP_011316143.1 MGRA|XP_003854589.1 MLAR|XP_007410728.1 MORY|QBZ56006.1 NCRA|XP_011393110.1 SSCL|APA06354.1

>Orthogroup2426: ANID|CBF74421.1 BCIN|XP_001546630.1 BGRA|VDB89510.1 CFRU|XP_031883908.1 CGLO|KAF3805062.1 CHIG|XP_018163455.1 CVIN|KAF4902227.1 CVYL|A06498 FGRM|XP_011318757.1 MGRA|XP_003854977.1 MLAR|XP_007411997.1 MORY|QBZ59533.1 NCRA|XP_957491.2 SSCL|APA06344.1

>Orthogroup2427: ANID|CBF74423.1 BCIN|XP_024551280.1 BGRA|VCU39474.1 CFRU|XP_031880725.1 CGLO|KAF3811157.1 CHIG|XP_018160346.1 CVIN|KAF4931272.1 CVYL|A04316 FGRM|XP_011324729.1 MGRA|XP_003854771.1 MLAR|XP_007404835.1 MORY|QBZ58015.1 NCRA|XP_961191.3 SSCL|APA10262.1

>Orthogroup2428: ANID|CBF74424.1 BCIN|XP_001556700.1 BGRA|VDB85821.1 CFRU|XP_031879851.1 CGLO|KAF3803018.1 CHIG|XP_018153567.1 CVIN|KAF4929977.1 CVYL|A00031 FGRM|XP_011316901.1 MGRA|XP_003854990.1 MLAR|XP_007413416.1 MORY|QBZ55446.1 NCRA|XP_963910.1 SSCL|APA06006.1

>Orthogroup2429: ANID|CBF74428.1 BCIN|XP_024551673.1 BGRA|VDB89543.1 CFRU|XP_031879855.1 CGLO|KAF3803022.1 CHIG|XP_018153570.1 CVIN|KAF4929972.1 CVYL|A00036 FGRM|XP_011316900.1 MGRA|XP_003855777.1 MLAR|XP_007409311.1 MORY|QBZ55447.1 NCRA|XP_963909.2 SSCL|APA06004.1

>Orthogroup2430: ANID|CBF74430.1 BCIN|XP_001556825.1 BGRA|VDB89514.1 CFRU|XP_031884021.1 CGLO|KAF3805164.1 CHIG|XP_018163348.1 CVIN|KAF4927489.1 CVYL|A06586 FGRM|XP_011321626.1 MGRA|XP_003854739.1 MLAR|XP_007414377.1 MORY|QBZ63293.1 NCRA|XP_956915.1 SSCL|APA14601.1

>Orthogroup2431: ANID|CBF74440.1 BCIN|XP_001559677.1 BGRA|VDB89323.1 CFRU|XP_031879407.1 CGLO|KAF3799645.1 CHIG|XP_018154655.1 CVIN|KAF4926442.1 CVYL|A13508 FGRM|XP_011317259.1 MGRA|XP_003857550.1 MLAR|XP_007404923.1 MORY|QBZ61017.1 NCRA|XP_963319.2 SSCL|APA09428.1

>Orthogroup2432: ANID|CBF74444.1 BCIN|XP_001559995.1 BGRA|VDB89372.1 CFRU|XP_031879451.1 CGLO|KAF3799647.1 CHIG|XP_018154657.1 CVIN|KAF4926440.1 CVYL|A13510 FGRM|XP_011315954.1 MGRA|XP_003851665.1 MLAR|XP_007404722.1 MORY|QBZ61011.1 NCRA|XP_956507.1 SSCL|APA09986.1

>Orthogroup2435: ANID|CBF74476.1 BCIN|XP_024551370.1 BGRA|VCU41010.1 CFRU|XP_031884214.1 CGLO|KAF3806477.1 CHIG|XP_018162373.1 CVIN|KAF4908630.1 CVYL|A12078 FGRM|XP_011323911.1 MGRA|XP_003853083.1 MLAR|XP_007410952.1 MORY|QBZ61494.1 NCRA|XP_957012.1 SSCL|APA10492.1

>Orthogroup2437: ANID|CBF74493.1 BCIN|XP_001555732.1 BGRA|VCU39814.1 CFRU|XP_031892958.1 CGLO|KAF3808060.1 CHIG|XP_018152764.1 CVIN|KAF4922851.1 CVYL|A08173 FGRM|XP_011317974.1 MGRA|XP_003857387.1 MLAR|XP_007417210.1 MORY|QBZ54986.1 NCRA|XP_011394488.1 SSCL|APA10300.1

>Orthogroup2438: ANID|CBF74496.1 BCIN|XP_001555738.1 BGRA|VCU39817.1 CFRU|XP_031893352.1 CGLO|KAF3808064.1 CHIG|XP_018152759.1 CVIN|KAF4922855.1 CVYL|A08169 FGRM|XP_011317970.1 MGRA|XP_003852664.1 MLAR|XP_007416215.1 MORY|QBZ54982.1 NCRA|XP_961582.3 SSCL|APA10307.1

>Orthogroup2439: ANID|CBF74521.1 BCIN|XP_001549887.2 BGRA|VDB84418.1 CFRU|XP_031885187.1 CGLO|KAF3807643.1 CHIG|XP_018162870.1 CVIN|KAF4927795.1 CVYL|A07808 FGRM|XP_011327039.1 MGRA|XP_003853360.1 MLAR|XP_007414088.1 MORY|QBZ61321.1 NCRA|XP_957310.3 SSCL|APA08228.1

>Orthogroup2440: ANID|CBF74523.1 BCIN|XP_001555748.2 BGRA|VCU39809.1 CFRU|XP_031893337.1 CGLO|KAF3808017.1 CHIG|XP_018152364.1 CVIN|KAF4909499.1 CVYL|A09466 FGRM|XP_011317760.1 MGRA|XP_003857404.1 MLAR|XP_007410967.1 MORY|QBZ58086.1 NCRA|XP_960713.1 SSCL|APA10111.1

>Orthogroup2441: ANID|CBF74525.1 BCIN|XP_001555749.1 BGRA|VCU39810.1 CFRU|XP_031892990.1 CGLO|KAF3808016.1 CHIG|XP_018152365.1 CVIN|KAF4909496.1 CVYL|A09467 FGRM|XP_011317761.1 MGRA|XP_003853094.1 MLAR|XP_007403567.1 MORY|QBZ58085.1 NCRA|XP_960712.1 SSCL|APA10112.1

>Orthogroup2442: ANID|CBF74545.1 BCIN|XP_001559111.1 BGRA|VDB94009.1 CFRU|XP_031887596.1 CGLO|KAF3811636.1 CHIG|XP_018160911.1 CVIN|KAF4921457.1 CVYL|A03497 FGRM|XP_011323580.1 MGRA|XP_003856020.1 MLAR|XP_007412637.1 MORY|QBZ56594.1 NCRA|XP_964434.2 SSCL|APA09049.1

>Orthogroup2446: ANID|CBF74571.1 BCIN|XP_024552501.1 BGRA|VDB84265.1 CFRU|XP_031885699.1 CGLO|KAF3810211.1 CHIG|XP_018157834.1 CVIN|KAF4929144.1 CVYL|A02807 FGRM|XP_011320321.1 MGRA|XP_003853347.1 MLAR|XP_007410333.1 MORY|QBZ62139.1 NCRA|XP_011394892.1 SSCL|APA08500.1

>Orthogroup2447: ANID|CBF74576.1 BCIN|XP_001551314.1 BGRA|VCU39705.1 CFRU|XP_031876381.1 CGLO|KAF3797773.1 CHIG|XP_018156443.1 CVIN|KAF4918830.1 CVYL|A02992 FGRM|XP_011318772.1 MGRA|XP_003852899.1 MLAR|XP_007408997.1 MORY|QBZ63673.1 NCRA|XP_960070.1 SSCL|APA07588.1

>Orthogroup2451: ANID|CBF74736.1 BCIN|XP_001551365.1 BGRA|VCU39505.1 CFRU|XP_031876929.1 CGLO|KAF3810726.1 CHIG|XP_018162585.1 CVIN|KAF4932127.1 CVYL|A07227 FGRM|XP_011323970.1 MGRA|XP_003852753.1 MLAR|XP_007405386.1 MORY|QBZ63897.1 NCRA|XP_956174.2 SSCL|APA10399.1

>Orthogroup2452: ANID|CBF74745.1 BCIN|XP_024551290.1 BGRA|VCU39522.1 CFRU|XP_031893493.1 CGLO|KAF3808001.1 CHIG|XP_018152378.1 CVIN|KAF4895814.1 CVYL|A09481 FGRM|XP_011317731.1 MGRA|XP_003852145.1 MLAR|XP_007413280.1 MORY|QBZ58066.1 NCRA|XP_011394701.1 SSCL|APA10245.1

>Orthogroup2453: ANID|CBF74771.1 BCIN|XP_001548049.1 BGRA|VDB89116.1 CFRU|XP_031886779.1 CGLO|KAF3801238.1 CHIG|XP_018162621.1 CVIN|KAF4932119.1 CVYL|A07091 FGRM|XP_011326657.1 MGRA|XP_003856298.1 MLAR|XP_007412905.1 MORY|QBZ59589.1 NCRA|XP_001728209.1 SSCL|APA10517.1

>Orthogroup2454: ANID|CBF74777.1 BCIN|XP_001554192.2 BGRA|VCU39771.1 CFRU|XP_031886415.1 CGLO|KAF3806356.1 CHIG|XP_018162237.1 CVIN|KAF4923255.1 CVYL|A11945 FGRM|XP_011324046.1 MGRA|XP_003852018.1 MLAR|XP_007410162.1 MORY|QBZ61604.1 NCRA|XP_955951.1 SSCL|APA10171.1

>Orthogroup2455: ANID|CBF74811.1 BCIN|XP_024549794.1 BGRA|VDB83666.1 CFRU|XP_031882531.1 CGLO|KAF3803929.1 CHIG|XP_018151015.1 CVYL|A08872 FGRM|XP_011321766.1 FGRM|XP_011323825.1 MGRA|XP_003848989.1 MLAR|XP_007408868.1 MORY|QBZ58794.1 NCRA|XP_961922.1 SSCL|APA05745.1

>Orthogroup2456: ANID|CBF74819.1 BCIN|XP_001547107.1 BGRA|VDB83575.1 CFRU|XP_031876535.1 CGLO|KAF3811476.1 CHIG|XP_018160729.1 CVIN|KAF4907411.1 CVYL|A04680 FGRM|XP_011317771.1 MGRA|XP_003848889.1 MLAR|XP_007407413.1 MORY|QBZ56599.1 NCRA|XP_962679.1 SSCL|APA08389.1

>Orthogroup2457: ANID|CBF74821.1 BCIN|XP_024549628.1 BGRA|VDB83696.1 CFRU|XP_031885437.1 CGLO|KAF3798656.1 CHIG|XP_018157504.1 CVIN|KAF4919444.1 CVYL|A09394 FGRM|XP_011324838.1 MGRA|XP_003848964.1 MLAR|XP_007409521.1 MORY|QBZ64518.1 NCRA|XP_961771.2 SSCL|APA05755.1

>Orthogroup2458: ANID|CBF74823.1 BCIN|XP_001551503.1 BGRA|VDB83695.1 CFRU|XP_031885383.1 CGLO|KAF3798657.1 CHIG|XP_018157503.1 CVIN|KAF4919458.1 CVYL|A09395 FGRM|XP_011324789.1 MGRA|XP_003848873.1 MLAR|XP_007414864.1 MORY|QBZ64517.1 NCRA|XP_001728322.1 SSCL|APA05754.1

>Orthogroup2461: ANID|CBF74837.1 BCIN|XP_001551510.1 BGRA|VCU40207.1 CFRU|XP_031880598.1 CGLO|KAF3811510.1 CHIG|XP_018160766.1 CVIN|KAF4926762.1 CVYL|A04717 FGRM|XP_011317808.1 MGRA|XP_003848753.1 MLAR|XP_007404518.1 MORY|QBZ58734.1 NCRA|XP_961909.1 SSCL|APA05760.1

>Orthogroup2462: ANID|CBF74847.1 BCIN|XP_024546296.1 BGRA|VDB91088.1 CFRU|XP_031882915.1 CGLO|KAF3812060.1 CHIG|XP_018164183.1 CVIN|KAF4923662.1 CVYL|A14237 FGRM|XP_011326791.1 MGRA|XP_003852647.1 MLAR|XP_007411883.1 MORY|QBZ63787.1 NCRA|XP_959160.2 SSCL|APA06928.1

>Orthogroup2463: ANID|CBF74864.1 BCIN|XP_001548645.1 BGRA|VDB83877.1 CFRU|XP_031884510.1 CGLO|KAF3799681.1 CHIG|XP_018158273.1 CVIN|KAF4919961.1 CVYL|A05845 FGRM|XP_011316517.1 MGRA|XP_003855887.1 MLAR|XP_007412974.1 MORY|QBZ59230.1 NCRA|XP_965581.1 SSCL|APA09633.1

>Orthogroup2464: ANID|CBF74884.1 BCIN|XP_024549623.1 BGRA|VCU40198.1 CFRU|XP_031880525.1 CGLO|KAF3811511.1 CHIG|XP_018160767.1 CVIN|KAF4926761.1 CVYL|A04718 FGRM|XP_011317809.1 MGRA|XP_003848900.1 MLAR|XP_007414193.1 MORY|QBZ58733.1 NCRA|XP_961910.2 SSCL|APA05761.1

>Orthogroup2468: ANID|CBF75018.1 BCIN|XP_001558673.1 BGRA|VDB91004.1 CFRU|XP_031882738.1 CGLO|KAF3803411.1 CHIG|XP_018163923.1 CVIN|KAF4918439.1 CVYL|A14339 FGRM|XP_011316876.1 MGRA|XP_003851730.1 MLAR|XP_007406794.1 MORY|QBZ63193.1 NCRA|XP_964959.2 SSCL|APA05446.1

>Orthogroup2469: ANID|CBF75048.1 BCIN|XP_001558705.2 BGRA|VDB91020.1 CFRU|XP_031888312.1 CGLO|KAF3803546.1 CHIG|XP_018164144.1 CVIN|KAF4922249.1 CVYL|A14479 FGRM|XP_011316155.1 MGRA|XP_003853758.1 MLAR|XP_007407427.1 MORY|QBZ56435.1 NCRA|XP_964696.1 SSCL|APA05468.1

>Orthogroup2470: ANID|CBF75050.1 BCIN|XP_024548000.1 BGRA|VDB91061.1 CFRU|XP_031888540.1 CGLO|KAF3803752.1 CHIG|XP_018153161.1 CVIN|KAF4912469.1 CVYL|A14132 FGRM|XP_011316017.1 MGRA|XP_003851647.1 MLAR|XP_007405618.1 MORY|QBZ59760.1 NCRA|XP_965092.3 SSCL|APA08332.1

>Orthogroup2471: ANID|CBF75060.1 BCIN|XP_001546495.1 BGRA|VDB91167.1 CFRU|XP_031890473.1 CGLO|KAF3804931.1 CHIG|XP_018164433.1 CVIN|KAF4919431.1 CVYL|A12123 FGRM|XP_011324187.1 MGRA|XP_003851498.1 MLAR|XP_007415249.1 MORY|QBZ56394.1 NCRA|XP_964826.1 SSCL|APA08418.1

>Orthogroup2472: ANID|CBF75062.1 BCIN|XP_024547942.1 BGRA|VDB91168.1 CFRU|XP_031890465.1 CGLO|KAF3804930.1 CHIG|XP_018164431.1 CVIN|KAF4919408.1 CVYL|A12124 FGRM|XP_011324190.1 MGRA|XP_003851845.1 MLAR|XP_007414229.1 MORY|QBZ56392.1 NCRA|XP_964824.1 SSCL|APA08417.1

>Orthogroup2473: ANID|CBF75064.1 BCIN|XP_001549097.1 BGRA|VDB90909.1 CFRU|XP_031888357.1 CGLO|KAF3803619.1 CHIG|XP_018163846.1 CVIN|KAF4920357.1 CVYL|A14551 FGRM|XP_011326548.1 MGRA|XP_003851747.1 MLAR|XP_007418509.1 MORY|QBZ63197.1 NCRA|XP_963951.1 SSCL|APA06224.1

>Orthogroup2474: ANID|CBF75090.1 BCIN|XP_001554074.2 BGRA|VDB90888.1 CFRU|XP_031888228.1 CGLO|KAF3803637.1 CHIG|XP_018163822.1 CVIN|KAF4913951.1 CVYL|A14572 FGRM|XP_011317062.1 MGRA|XP_003851703.1 MLAR|XP_007407825.1 MORY|QBZ54694.1 NCRA|XP_011393384.1 SSCL|APA05565.1

>Orthogroup2475: ANID|CBF75092.1 BCIN|XP_001554075.1 BGRA|VDB90884.1 CFRU|XP_031888379.1 CGLO|KAF3803636.1 CHIG|XP_018163823.1 CVIN|KAF4913946.1 CVYL|A14571 FGRM|XP_011317063.1 MGRA|XP_003851920.1 MLAR|XP_007407293.1 MORY|QBZ54693.1 NCRA|XP_011393386.1 SSCL|APA05564.1

>Orthogroup2476: ANID|CBF75121.1 BCIN|XP_024548776.1 BGRA|VDB83900.1 CFRU|XP_031890462.1 CGLO|KAF3805277.1 CHIG|XP_018158597.1 CVIN|KAF4901289.1 CVYL|A13928 FGRM|XP_011328412.1 MGRA|XP_003857142.1 MLAR|XP_007418314.1 MORY|QBZ55949.1 NCRA|XP_965687.2 SSCL|APA09698.1

>Orthogroup2479: ANID|CBF75184.1 BCIN|XP_001553932.1 BGRA|VCU39269.1 CFRU|XP_031878768.1 CGLO|KAF3797564.1 CHIG|XP_018151103.1 CVIN|KAF4923593.1 CVYL|A08982 FGRM|XP_011323493.1 MGRA|XP_003849685.1 MLAR|XP_007404619.1 MORY|QBZ64240.1 NCRA|XP_962569.1 SSCL|APA13110.1

>Orthogroup2480: ANID|CBF75207.1 BCIN|XP_024548759.1 BGRA|VDB89182.1 CFRU|XP_031882295.1 CGLO|KAF3804432.1 CHIG|XP_018160254.1 CVIN|KAF4930899.1 CVYL|A04138 FGRM|XP_011324520.1 MGRA|XP_003850640.1 MLAR|XP_007404546.1 MORY|QBZ54290.1 NCRA|XP_961359.3 SSCL|APA09726.1

>Orthogroup2481: ANID|CBF75211.1 BCIN|XP_001559621.1 BGRA|VDB89192.1 CFRU|XP_031882288.1 CGLO|KAF3804430.1 CHIG|XP_018160252.1 CVIN|KAF4930998.1 CVYL|A04136 FGRM|XP_011324518.1 MGRA|XP_003850598.1 MLAR|XP_007412611.1 MORY|QBZ54292.1 NCRA|XP_961106.1 SSCL|APA09392.1

>Orthogroup2482: ANID|CBF75213.1 BCIN|XP_001559620.1 BGRA|VDB89191.1 CFRU|XP_031882296.1 CGLO|KAF3804431.1 CHIG|XP_018160253.1 CVIN|KAF4930902.1 CVYL|A04137 FGRM|XP_011324519.1 MGRA|XP_003851256.1 MLAR|XP_007405271.1 MORY|QBZ54291.1 NCRA|XP_961358.1 SSCL|APA09391.1

>Orthogroup2483: ANID|CBF75230.1 BCIN|XP_024548770.1 BGRA|VDB89270.1 CFRU|XP_031882183.1 CGLO|KAF3804427.1 CHIG|XP_018160249.1 CVIN|KAF4930903.1 CVYL|A04133 FGRM|XP_011326882.1 MGRA|XP_003851264.1 MLAR|XP_007408493.1 MORY|QBZ53849.1 NCRA|XP_961110.1 SSCL|APA09710.1

>Orthogroup2484: ANID|CBF75261.1 BCIN|XP_024546026.1 BGRA|VCU39824.1 CFRU|XP_031889971.1 CGLO|KAF3802859.1 CHIG|XP_018158459.1 CVIN|KAF4899965.1 CVYL|A05151 FGRM|XP_011316387.1 MGRA|XP_003853305.1 MLAR|XP_007410603.1 MORY|QBZ55914.1 NCRA|XP_964811.1 SSCL|APA07302.1

>Orthogroup2485: ANID|CBF75263.1 BCIN|XP_001555643.1 BGRA|VDB92794.1 CFRU|XP_031889970.1 CGLO|KAF3802858.1 CHIG|XP_018158463.1 CVIN|KAF4923849.1 CVYL|A05150 FGRM|XP_011316388.1 MGRA|XP_003853099.1 MLAR|XP_007417874.1 MORY|QBZ55913.1 NCRA|XP_964812.2 SSCL|APA07303.1

>Orthogroup2486: ANID|CBF75267.1 BCIN|XP_001548531.1 BGRA|VDB92585.1 CFRU|XP_031889596.1 CGLO|KAF3810571.1 CHIG|XP_018159025.1 CVIN|KAF4914224.1 CVYL|A13650 FGRM|XP_011328348.1 MGRA|XP_003852702.1 MLAR|XP_007418651.1 MORY|QBZ57350.1 NCRA|XP_011395353.1 SSCL|APA07227.1

>Orthogroup2487: ANID|CBF75283.1 BCIN|XP_001560460.1 BGRA|VCU39881.1 CFRU|XP_031889545.1 CGLO|KAF3810476.1 CHIG|XP_018159131.1 CVIN|KAF4924633.1 CVYL|A14078 FGRM|XP_011317187.1 MGRA|XP_003852063.1 MLAR|XP_007408190.1 MORY|QBZ56383.1 NCRA|XP_965342.2 SSCL|APA15714.1

>Orthogroup2488: ANID|CBF75287.1 BCIN|XP_024545913.1 BGRA|VCU39840.1 CFRU|XP_031889910.1 CGLO|KAF3810583.1 CHIG|XP_018159019.1 CVIN|KAF4914200.1 CVYL|A13663 FGRM|XP_011315944.1 MGRA|XP_003853570.1 MLAR|XP_007406923.1 MORY|QBZ57344.1 NCRA|XP_011395184.1 SSCL|APA07328.1

>Orthogroup2489: ANID|CBF75298.1 BCIN|XP_001548523.1 BGRA|VCU39826.1 CFRU|XP_031889523.1 CGLO|KAF3810586.1 CHIG|XP_018159015.1 CVIN|KAF4914203.1 CVYL|A13666 FGRM|XP_011316586.1 MGRA|XP_003852124.1 MLAR|XP_007408347.1 MORY|QBZ57347.1 NCRA|XP_959627.2 SSCL|APA07233.1

>Orthogroup2490: ANID|CBF75300.1 BCIN|XP_024546314.1 BGRA|VDB90749.1 CFRU|XP_031888241.1 CGLO|KAF3803477.1 CHIG|XP_018164007.1 CVIN|KAF4926324.1 CVYL|A14406 FGRM|XP_011316867.1 MGRA|XP_003855931.1 MLAR|XP_007414392.1 MORY|QBZ55350.1 NCRA|XP_957081.1 SSCL|APA06909.1

>Orthogroup2491: ANID|CBF75302.1 BCIN|XP_024549021.1 BGRA|VDB93303.1 CFRU|XP_031884661.1 CGLO|KAF3799686.1 CHIG|XP_018158277.1 CVIN|KAF4919963.1 CVYL|A05849 FGRM|XP_011316511.1 MGRA|XP_003857722.1 MLAR|XP_007415993.1 MORY|QBZ54147.1 NCRA|XP_011393215.1 SSCL|APA09294.1

>Orthogroup2492: ANID|CBF75324.1 BCIN|XP_001559963.2 BGRA|VDB93251.1 CFRU|XP_031890608.1 CGLO|KAF3805269.1 CHIG|XP_018158805.1 CVIN|KAF4918679.1 CVYL|A13918 FGRM|XP_011316673.1 MGRA|XP_003857794.1 MLAR|XP_007405168.1 MORY|QBZ60399.1 NCRA|XP_011393083.1 SSCL|APA09781.1

>Orthogroup2493: ANID|CBF75330.1 BCIN|XP_001548399.1 BGRA|VDB92651.1 CFRU|XP_031884666.1 CGLO|KAF3799072.1 CHIG|XP_018158186.1 CVIN|KAF4918246.1 CVYL|A11457 FGRM|XP_011328382.1 MGRA|XP_003856110.1 MLAR|XP_007416817.1 MORY|QBZ54108.1 NCRA|XP_964355.3 SSCL|APA09266.1

>Orthogroup2494: ANID|CBF75334.1 BCIN|XP_024548727.1 BGRA|VDB93252.1 CFRU|XP_031890607.1 CGLO|KAF3805270.1 CHIG|XP_018158804.1 CVIN|KAF4901293.1 CVYL|A13919 FGRM|XP_011316671.1 MGRA|XP_003855869.1 MLAR|XP_007414748.1 MORY|QBZ60398.1 NCRA|XP_964973.1 SSCL|APA09780.1

>Orthogroup2495: ANID|CBF75336.1 BCIN|XP_001559960.1 BGRA|VCU40228.1 CFRU|XP_031884447.1 CGLO|KAF3799760.1 CHIG|XP_018158243.1 CVIN|KAF4918956.1 CVYL|A11335 FGRM|XP_011328583.1 MGRA|XP_003857770.1 MLAR|XP_007408734.1 MORY|QBZ55859.1 NCRA|XP_964547.1 SSCL|APA09778.1

>Orthogroup2496: ANID|CBF75360.1 BCIN|XP_024549118.1 BGRA|VCU39864.1 CFRU|XP_031889845.1 CGLO|KAF3810548.1 CHIG|XP_018159049.1 CVIN|KAF4895561.1 CVYL|A13625 FGRM|XP_011328367.1 MGRA|XP_003853052.1 MLAR|XP_007409780.1 MORY|QBZ54519.1 NCRA|XP_959814.1 SSCL|APA08852.1

>Orthogroup2497: ANID|CBF75362.1 BCIN|XP_001556552.1 BGRA|VDB89199.1 CFRU|XP_031885560.1 CGLO|KAF3810156.1 CHIG|XP_018157990.1 CVIN|KAF4929161.1 CVYL|A02746 FGRM|XP_011320371.1 MGRA|XP_003852691.1 MLAR|XP_007410392.1 MORY|QBZ62636.1 NCRA|XP_960368.3 SSCL|APA06482.1

>Orthogroup2498: ANID|CBF75366.1 BCIN|XP_024546252.1 BGRA|VCU39833.1 CFRU|XP_031889798.1 CGLO|KAF3805880.1 CHIG|XP_018158888.1 CVIN|KAF4920789.1 CVYL|A13833 FGRM|XP_011316682.1 MGRA|XP_003856278.1 MLAR|XP_007406443.1 MORY|QBZ60523.1 NCRA|XP_964108.1 SSCL|APA06974.1

>Orthogroup2499: ANID|CBF75370.1 BCIN|XP_001555541.1 BGRA|VDB92477.1 CFRU|XP_031889765.1 CGLO|KAF3805902.1 CHIG|XP_018158915.1 CVIN|KAF4920794.1 CVYL|A13810 FGRM|XP_011315912.1 MGRA|XP_003856279.1 MLAR|XP_007406510.1 MORY|QBZ60520.1 NCRA|XP_965790.2 SSCL|APA07418.1

>Orthogroup2500: ANID|CBF75390.1 BCIN|XP_024549080.1 BGRA|VDB91361.1 CFRU|XP_031890515.1 CGLO|KAF3806765.1 CHIG|XP_018158782.1 CVIN|KAF4919472.1 CVYL|A04978 FGRM|XP_011316362.1 MGRA|XP_003852101.1 MLAR|XP_007411538.1 MORY|QBZ60361.1 NCRA|XP_965260.3 SSCL|APA15867.1

>Orthogroup2501: ANID|CBF75402.1 BCIN|XP_001553859.1 BGRA|VDB92678.1 CFRU|XP_031878654.1 CGLO|KAF3797556.1 CHIG|XP_018151110.1 CVIN|KAF4923594.1 CVYL|A08991 FGRM|XP_011323496.1 MGRA|XP_003850083.1 MLAR|XP_007406113.1 MORY|QBZ64067.1 NCRA|XP_962572.1 SSCL|APA13077.1

>Orthogroup2502: ANID|CBF75403.1 BCIN|XP_001552196.1 BGRA|VCU39497.1 CFRU|XP_031887645.1 CGLO|KAF3803192.1 CHIG|XP_018161030.1 CVIN|KAF4920204.1 CVYL|A03622 FGRM|XP_011323811.1 MGRA|XP_003849260.1 MLAR|XP_007410406.1 MORY|QBZ58842.1 NCRA|XP_011394325.1 SSCL|APA13010.1

>Orthogroup2503: ANID|CBF75419.1 BCIN|XP_001557471.1 BGRA|VCU41043.1 CFRU|XP_031885218.1 CGLO|KAF3807692.1 CHIG|XP_018162919.1 CVIN|KAF4927831.1 CVYL|A06885 FGRM|XP_011326259.1 MGRA|XP_003849195.1 MLAR|XP_007418974.1 MORY|QBZ59694.1 NCRA|XP_957698.1 SSCL|APA10639.1

>Orthogroup2505: ANID|CBF75454.1 BCIN|XP_001556517.2 BGRA|VDB92425.1 CFRU|XP_031890500.1 CGLO|KAF3804831.1 CHIG|XP_018164307.1 CVIN|KAF4929784.1 CVYL|A12242 FGRM|XP_011324157.1 MGRA|XP_003850703.1 MLAR|XP_007417411.1 MORY|QBZ63542.1 NCRA|XP_964222.1 SSCL|APA06504.1

>Orthogroup2506: ANID|CBF75456.1 BCIN|XP_024546407.1 BGRA|VCU41225.1 CFRU|XP_031882802.1 CGLO|KAF3812045.1 CHIG|XP_018164167.1 CVIN|KAF4923698.1 CVYL|A14224 FGRM|XP_011326777.1 MGRA|XP_003850629.1 MLAR|XP_007412753.1 MORY|QBZ63229.1 NCRA|XP_011393132.1 SSCL|APA07222.1

>Orthogroup2507: ANID|CBF75460.1 BCIN|XP_024546581.1 BGRA|VDB92507.1 CFRU|XP_031879933.1 CGLO|KAF3798983.1 CHIG|XP_018152890.1 CVIN|KAF4923453.1 CVYL|A07904 FGRM|XP_011324193.1 MGRA|XP_003851240.1 MLAR|XP_007411843.1 MORY|QBZ53412.1 NCRA|XP_958140.1 SSCL|APA06491.1

>Orthogroup2508: ANID|CBF75466.1 BCIN|XP_024546408.1 BGRA|VDB92926.1 CFRU|XP_031890486.1 CGLO|KAF3804908.1 CHIG|XP_018164355.1 CVIN|KAF4909781.1 CVYL|A12150 FGRM|XP_011316796.1 MGRA|XP_003851244.1 MLAR|XP_007413885.1 MORY|QBZ56442.1 NCRA|XP_963947.3 SSCL|APA07224.1

>Orthogroup2509: ANID|CBF75476.1 BCIN|XP_001561243.1 BGRA|VDB86371.1 CFRU|XP_031890569.1 CGLO|KAF3804893.1 CHIG|XP_018164369.1 CVIN|KAF4921177.1 CVYL|A12168 FGRM|XP_011317156.1 MGRA|XP_003851212.1 MLAR|XP_007405230.1 MORY|QBZ55301.1 NCRA|XP_964024.3 SSCL|APA07218.1

>Orthogroup2510: ANID|CBF75480.1 BCIN|XP_024549386.1 BGRA|VDB92950.1 CFRU|XP_031888391.1 CGLO|KAF3803704.1 CHIG|XP_018163747.1 CVIN|KAF4928554.1 CVYL|A14636 FGRM|XP_011326812.1 MGRA|XP_003851108.1 MLAR|XP_007415291.1 MORY|QBZ54622.1 NCRA|XP_965056.2 SSCL|APA09037.1

>Orthogroup2511: ANID|CBF75482.1 BCIN|XP_024546682.1 BGRA|VDB92962.1 CFRU|XP_031888394.1 CGLO|KAF3803703.1 CHIG|XP_018163748.1 CVIN|KAF4928531.1 CVYL|A14635 FGRM|XP_011326810.1 MGRA|XP_003850737.1 MLAR|XP_007411530.1 MORY|QBZ54621.1 NCRA|XP_011393310.1 SSCL|APA06530.1

>Orthogroup2512: ANID|CBF75486.1 BCIN|XP_001556484.1 BGRA|VDB92963.1 CFRU|XP_031888396.1 CGLO|KAF3803701.1 CHIG|XP_018163750.1 CVIN|KAF4928553.1 CVYL|A14633 FGRM|XP_011326808.1 MGRA|XP_003851065.1 MLAR|XP_007409736.1 MORY|QBZ54619.1 NCRA|XP_963879.1 SSCL|APA06532.1

>Orthogroup2516: ANID|CBF75527.1 BCIN|XP_024553177.1 BGRA|VDB88961.1 CFRU|XP_031877673.1 CGLO|KAF3801062.1 CHIG|XP_018153708.1 CVIN|KAF4912158.1 CVYL|A00157 FGRM|XP_011327830.1 MGRA|XP_003851868.1 MLAR|XP_007409132.1 MORY|QBZ59014.1 NCRA|XP_959603.2 SSCL|APA13436.1

>Orthogroup2517: ANID|CBF75533.1 BCIN|XP_001546569.1 BGRA|VCU40054.1 CFRU|XP_031892113.1 CGLO|KAF3800064.1 CHIG|XP_018165013.1 CVIN|KAF4919344.1 CVYL|A06763 FGRM|XP_011324991.1 MGRA|XP_003851555.1 MLAR|XP_007404532.1 MORY|QBZ60010.1 NCRA|XP_956336.3 SSCL|APA14127.1

>Orthogroup2518: ANID|CBF75535.1 BCIN|XP_001547614.1 BGRA|VDB89019.1 CFRU|XP_031881963.1 CGLO|KAF3806157.1 CHIG|XP_018164504.1 CVIN|KAF4891768.1 CVYL|A07350 FGRM|XP_011325046.1 MGRA|XP_003851964.1 MLAR|XP_007404260.1 MORY|QBZ59008.1 NCRA|XP_959711.1 SSCL|APA14322.1

>Orthogroup2519: ANID|CBF75541.1 BCIN|XP_024547189.1 BGRA|VCU39589.1 CFRU|XP_031877614.1 CGLO|KAF3801080.1 CHIG|XP_018153731.1 CVIN|KAF4892467.1 CVYL|A00393 FGRM|XP_011327802.1 MGRA|XP_003848331.1 MLAR|XP_007409682.1 MORY|QBZ60609.1 NCRA|XP_959794.3 SSCL|APA14108.1

>Orthogroup2520: ANID|CBF75564.1 BCIN|XP_024547182.1 BGRA|VCU40059.1 CFRU|XP_031877608.1 CGLO|KAF3801074.1 CHIG|XP_018153722.1 CVIN|KAF4902046.1 CVYL|A00035 FGRM|XP_011327794.1 MGRA|XP_003848667.1 MLAR|XP_007415427.1 MORY|QBZ62337.1 NCRA|XP_959223.2 SSCL|APA14116.1

>Orthogroup2522: ANID|CBF75570.1 BCIN|XP_001546159.2 BGRA|VCU39665.1 CFRU|XP_031885534.1 CGLO|KAF3799533.1 CHIG|XP_018154523.1 CVIN|KAF4931834.1 CVYL|A13393 FGRM|XP_011324919.1 MGRA|XP_003853527.1 MLAR|XP_007414834.1 MORY|QBZ62067.1 NCRA|XP_963514.1 SSCL|APA16282.1

>Orthogroup2523: ANID|CBF75578.1 BCIN|XP_001546406.1 BGRA|VDB88789.1 CFRU|XP_031877960.1 CGLO|KAF3810906.1 CHIG|XP_018154939.1 CVIN|KAF4916362.1 CVYL|A02239 FGRM|XP_011324628.1 MGRA|XP_003856400.1 MLAR|XP_007412814.1 MORY|QBZ53816.1 NCRA|XP_963218.1 SSCL|APA12262.1

>Orthogroup2524: ANID|CBF75589.1 BCIN|XP_001560762.2 BGRA|VDB90438.1 CFRU|XP_031883291.1 CGLO|KAF3801696.1 CHIG|XP_018159215.1 CVIN|KAF4915644.1 CVYL|A13002 FGRM|XP_011320278.1 MGRA|XP_003853784.1 MLAR|XP_007415669.1 MORY|QBZ55466.1 NCRA|XP_960232.2 SSCL|APA08017.1

>Orthogroup2525: ANID|CBF75591.1 BCIN|XP_024552908.1 BGRA|VCU39673.1 CFRU|XP_031879412.1 CGLO|KAF3799638.1 CHIG|XP_018154647.1 CVIN|KAF4926479.1 CVYL|A13501 FGRM|XP_011326135.1 MGRA|XP_003852745.1 MLAR|XP_007403901.1 MORY|QBZ61035.1 NCRA|XP_957115.2 SSCL|APA07521.1

>Orthogroup2526: ANID|CBF75596.1 BCIN|XP_024553056.1 BGRA|VCU39714.1 CFRU|XP_031878047.1 CGLO|KAF3799633.1 CHIG|XP_018154640.1 CVIN|KAF4926438.1 CVYL|A13496 FGRM|XP_011315983.1 MGRA|XP_003856406.1 MLAR|XP_007416335.1 MORY|QBZ61030.1 NCRA|XP_011393598.1 SSCL|APA16143.1

>Orthogroup2527: ANID|CBF75602.1 BCIN|XP_024547348.1 BGRA|VDB89032.1 CFRU|XP_031881988.1 CGLO|KAF3806057.1 CHIG|XP_018164624.1 CVIN|KAF4913562.1 CVYL|A07459 FGRM|XP_011326737.1 MGRA|XP_003851624.1 MLAR|XP_007404244.1 MORY|QBZ62351.1 NCRA|XP_959229.2 SSCL|APA14343.1

>Orthogroup2528: ANID|CBF75604.1 BCIN|XP_001554270.1 BGRA|VDB88953.1 CFRU|XP_031881951.1 CGLO|KAF3806010.1 CHIG|XP_018164655.1 CVIN|KAF4925734.1 CVYL|A07502 FGRM|XP_011325019.1 MGRA|XP_003851623.1 MLAR|XP_007413999.1 MORY|QBZ60617.1 NCRA|XP_959596.1 SSCL|APA14365.1

>Orthogroup2529: ANID|CBF75605.1 BCIN|XP_024547366.1 BGRA|VDB88955.1 CFRU|XP_031881948.1 CGLO|KAF3806013.1 CHIG|XP_018164652.1 CVIN|KAF4917819.1 CVYL|A07499 FGRM|XP_011325021.1 MGRA|XP_003851622.1 MLAR|XP_007411241.1 MORY|QBZ60619.1 NCRA|XP_959541.3 SSCL|APA14366.1

>Orthogroup2530: ANID|CBF75613.1 BCIN|XP_024547339.1 BGRA|VDB89014.1 CFRU|XP_031891954.1 CGLO|KAF3805593.1 CHIG|XP_018164723.1 CVIN|KAF4900693.1 CVYL|A07605 FGRM|XP_011324973.1 MGRA|XP_003851629.1 MLAR|XP_007416069.1 MORY|QBZ60938.1 NCRA|XP_959970.2 SSCL|APA14334.1

>Orthogroup2531: ANID|CBF75615.1 BCIN|XP_024551857.1 BGRA|VDB95094.1 CFRU|XP_031883925.1 CGLO|KAF3797450.1 CHIG|XP_018163555.1 CVIN|KAF4905676.1 CVYL|A09884 FGRM|XP_011316961.1 MGRA|XP_003856616.1 MLAR|XP_007409297.1 MORY|QBZ54889.1 NCRA|XP_962149.2 SSCL|APA06304.1

>Orthogroup2534: ANID|CBF75671.1 BCIN|XP_001552408.1 BGRA|VDB89697.1 CFRU|XP_031890754.1 CGLO|KAF3801671.1 CHIG|XP_018151449.1 CVIN|KAF4897552.1 CVYL|A12465 FGRM|XP_011328482.1 MGRA|XP_003857039.1 MLAR|XP_007410597.1 MORY|QBZ62161.1 NCRA|XP_957575.1 SSCL|APA14894.1

>Orthogroup2535: ANID|CBF75673.1 BCIN|XP_024551815.1 BGRA|VDB92642.1 CFRU|XP_031890671.1 CGLO|KAF3804953.1 CHIG|XP_018164460.1 CVIN|KAF4910221.1 CVYL|A12101 FGRM|XP_011317107.1 MGRA|XP_003856780.1 MLAR|XP_007415146.1 MORY|QBZ63816.1 NCRA|XP_964072.1 SSCL|APA14699.1

>Orthogroup2536: ANID|CBF75677.1 BCIN|XP_024551682.1 BGRA|VDB95092.1 CFRU|XP_031879838.1 CGLO|KAF3803005.1 CHIG|XP_018153551.1 CVIN|KAF4929902.1 CVYL|A00017 FGRM|XP_011316956.1 MGRA|XP_003856750.1 MLAR|XP_007418523.1 MORY|QBZ63165.1 NCRA|XP_963787.2 SSCL|APA06017.1

>Orthogroup2537: ANID|CBF75693.1 BCIN|XP_001558437.1 BGRA|VDB90451.1 CFRU|XP_031876502.1 CGLO|KAF3797012.1 CHIG|XP_018153672.1 CVIN|KAF4895820.1 CVYL|A00377 FGRM|XP_011317072.1 MGRA|XP_003856391.1 MLAR|XP_007407844.1 MORY|QBZ63930.1 NCRA|XP_963850.2 SSCL|APA14745.1

>Orthogroup2538: ANID|CBF75703.1 BCIN|XP_001556623.1 BGRA|VDB92667.1 CFRU|XP_031879870.1 CGLO|KAF3802976.1 CHIG|XP_018153637.1 CVIN|KAF4929907.1 CVYL|A00409 FGRM|XP_011316897.1 MGRA|XP_003856924.1 MLAR|XP_007406604.1 MORY|QBZ63572.1 NCRA|XP_963828.1 SSCL|APA14763.1

>Orthogroup2539: ANID|CBF75710.1 BCIN|XP_024546548.1 BGRA|VDB95016.1 CFRU|XP_031889124.1 CGLO|KAF3797712.1 CHIG|XP_018155501.1 CVIN|KAF4928300.1 CVYL|A01063 FGRM|XP_011328462.1 MGRA|XP_003856807.1 MLAR|XP_007404102.1 MORY|QBZ59954.1 NCRA|XP_960113.3 SSCL|APA06681.1

>Orthogroup2540: ANID|CBF75712.1 BCIN|XP_024551650.1 BGRA|VDB92657.1 CFRU|XP_031879784.1 CGLO|KAF3802990.1 CHIG|XP_018153623.1 CVIN|KAF4929921.1 CVYL|A00423 FGRM|XP_011316923.1 MGRA|XP_003856712.1 MLAR|XP_007406137.1 MORY|QBZ55600.1 NCRA|XP_965128.3 SSCL|APA14768.1

>Orthogroup2541: ANID|CBF75718.1 BCIN|XP_001558513.1 BGRA|VDB90602.1 CFRU|XP_031888374.1 CGLO|KAF3803593.1 CHIG|XP_018163872.1 CVIN|KAF4920356.1 CVYL|A14528 FGRM|XP_011316772.1 MGRA|XP_003856713.1 MLAR|XP_007403703.1 MORY|QBZ55378.1 NCRA|XP_965477.1 SSCL|APA14689.1

>Orthogroup2542: ANID|CBF75723.1 BCIN|XP_024551831.1 BGRA|VDB94990.1 CFRU|XP_031892322.1 CGLO|KAF3812276.1 CHIG|XP_018153500.1 CVIN|KAF4919860.1 CVYL|A00107 FGRM|XP_011316089.1 MGRA|XP_003856858.1 MLAR|XP_007409791.1 MORY|QBZ55605.1 NCRA|XP_011393247.1 SSCL|APA14727.1

>Orthogroup2543: ANID|CBF75729.1 BCIN|XP_024551823.1 BGRA|VCU40025.1 CFRU|XP_031883995.1 CGLO|KAF3805145.1 CHIG|XP_018163368.1 CVIN|KAF4927407.1 CVYL|A06411 FGRM|XP_011321616.1 MGRA|XP_003857108.1 MLAR|XP_007405664.1 MORY|QBZ63280.1 NCRA|XP_956919.1 SSCL|APA14716.1

>Orthogroup2544: ANID|CBF75735.1 BCIN|XP_024551850.1 BGRA|VDB95087.1 CFRU|XP_031882718.1 CGLO|KAF3803394.1 CHIG|XP_018163937.1 CVIN|KAF4918410.1 CVYL|A14324 FGRM|XP_011316148.1 MGRA|XP_003856577.1 MLAR|XP_007413442.1 MORY|QBZ56011.1 NCRA|XP_965304.1 SSCL|APA06289.1

>Orthogroup2546: ANID|CBF75745.1 BCIN|XP_024547794.1 BGRA|VDB94820.1 CFRU|XP_031879266.1 CGLO|KAF3808600.1 CHIG|XP_018161891.1 CVIN|KAF4926113.1 CVYL|A12382 FGRM|XP_011318526.1 MGRA|XP_003848800.1 MLAR|XP_007409044.1 MORY|QBZ59633.1 NCRA|XP_962869.1 SSCL|APA08061.1

>Orthogroup2547: ANID|CBF75747.1 BCIN|XP_001545754.2 BGRA|VCU39156.1 CFRU|XP_031882825.1 CGLO|KAF3803384.1 CHIG|XP_018164008.1 CVIN|KAF4918421.1 CVYL|A14311 FGRM|XP_011319678.1 MGRA|XP_003851551.1 MLAR|XP_007404025.1 MORY|QBZ56016.1 NCRA|XP_965294.2 SSCL|APA05473.1

>Orthogroup2548: ANID|CBF75752.1 BCIN|XP_001559608.2 BGRA|VDB84129.1 CFRU|XP_031890409.1 CGLO|KAF3805260.1 CHIG|XP_018158816.1 CVIN|KAF4918652.1 CVYL|A13909 FGRM|XP_011317311.1 MGRA|XP_003856048.1 MLAR|XP_007415276.1 MORY|QBZ60374.1 NCRA|XP_965245.1 SSCL|APA09380.1

>Orthogroup2551: ANID|CBF75796.1 BCIN|XP_024551976.1 BGRA|VCU39293.1 CFRU|XP_031893251.1 CGLO|KAF3800830.1 CHIG|XP_018150868.1 CVIN|KAF4922461.1 CVYL|A08719 FGRM|XP_011328050.1 MGRA|XP_003849306.1 MLAR|XP_007415828.1 MORY|QBZ64101.1 NCRA|XP_962261.1 SSCL|APA12689.1

>Orthogroup2552: ANID|CBF75804.1 BCIN|XP_024550120.1 BGRA|VDB86356.1 CFRU|XP_031892945.1 CGLO|KAF3809656.1 CHIG|XP_018152540.1 CVIN|KAF4925046.1 CVYL|A08395 FGRM|XP_011317917.1 MGRA|XP_003848572.1 MLAR|XP_007405619.1 MORY|QBZ54868.1 NCRA|XP_958705.1 SSCL|APA10590.1

>Orthogroup2553: ANID|CBF75819.1 BCIN|XP_001560997.1 BGRA|VDB90717.1 CFRU|XP_031888482.1 CGLO|KAF3803518.1 CHIG|XP_018164071.1 CVIN|KAF4922267.1 CVYL|A14448 FGRM|XP_011316164.1 MGRA|XP_003853151.1 MLAR|XP_007413029.1 MORY|QBZ56431.1 NCRA|XP_964700.3 SSCL|APA06722.1

>Orthogroup2554: ANID|CBF75827.1 BCIN|XP_024549880.1 BGRA|VDB91119.1 CFRU|XP_031888212.1 CGLO|KAF3803656.1 CHIG|XP_018163804.1 CVIN|KAF4928502.1 CVYL|A14589 FGRM|XP_011317008.1 MGRA|XP_003852643.1 MLAR|XP_007418125.1 MORY|QBZ54678.1 NCRA|XP_963751.3 SSCL|APA05552.1

>Orthogroup2555: ANID|CBF75831.1 BCIN|XP_001554093.1 BGRA|VDB90943.1 CFRU|XP_031888580.1 CGLO|KAF3803691.1 CHIG|XP_018163762.1 CVIN|KAF4928526.1 CVYL|A14622 FGRM|XP_011316037.1 MGRA|XP_003852396.1 MLAR|XP_007403524.1 MORY|QBZ54598.1 NCRA|XP_965667.1 SSCL|APA05555.1

>Orthogroup2556: ANID|CBF75835.1 BCIN|XP_001559941.1 BGRA|VDB93272.1 CFRU|XP_031886644.1 CGLO|KAF3807478.1 CHIG|XP_018158759.1 CVIN|KAF4907604.1 CVYL|A05001 FGRM|XP_011328628.1 MGRA|XP_003852641.1 MLAR|XP_007407470.1 MORY|QBZ60466.1 NCRA|XP_965318.1 SSCL|APA09768.1

>Orthogroup2558: ANID|CBF75851.1 BCIN|XP_024546117.1 BGRA|VDB90929.1 CFRU|XP_031892683.1 CGLO|KAF3803077.1 CHIG|XP_018153519.1 CVIN|KAF4919863.1 CVYL|A00089 FGRM|XP_011316108.1 MGRA|XP_003852532.1 MLAR|XP_007405395.1 MORY|QBZ55584.1 NCRA|XP_011393013.1 SSCL|APA06638.1

>Orthogroup2568: ANID|CBF76261.1 BCIN|XP_001547559.1 BGRA|VCU41182.1 CFRU|XP_031889248.1 CGLO|KAF3808715.1 CHIG|XP_018155576.1 CVIN|KAF4929562.1 CVYL|A00945 FGRM|XP_011319275.1 MGRA|XP_003856891.1 MLAR|XP_007406117.1 MORY|QBZ60124.1 NCRA|XP_957421.1 SSCL|APA07479.1

>Orthogroup2569: ANID|CBF76279.1 BCIN|XP_024552865.1 BGRA|VDB93949.1 CFRU|XP_031889448.1 CGLO|KAF3808815.1 CHIG|XP_018155718.1 CVIN|KAF4908030.1 CVYL|A01263 FGRM|XP_011319267.1 MGRA|XP_003856207.1 MLAR|XP_007417359.1 MORY|QBZ58716.1 NCRA|XP_960812.1 SSCL|APA16122.1

>Orthogroup2570: ANID|CBF76294.1 BCIN|XP_024547371.1 BGRA|VDB88939.1 CFRU|XP_031882072.1 CGLO|KAF3806002.1 CHIG|XP_018164649.1 CVIN|KAF4925705.1 CVYL|A07512 FGRM|XP_011324987.1 MGRA|XP_003851957.1 MLAR|XP_007416313.1 MORY|QBZ62328.1 NCRA|XP_959643.1 SSCL|APA14376.1

>Orthogroup2571: ANID|CBF76314.1 BCIN|XP_001555983.1 BGRA|VDB89002.1 CFRU|XP_031882047.1 CGLO|KAF3806071.1 CHIG|XP_018164619.1 CVIN|KAF4913556.1 CVYL|A07443 FGRM|XP_011316012.1 MGRA|XP_003851601.1 MLAR|XP_007409006.1 MORY|QBZ64928.1 NCRA|XP_959984.3 SSCL|APA13918.1

>Orthogroup2573: ANID|CBF76326.1 BCIN|XP_024547135.1 BGRA|VDB88843.1 CFRU|XP_031881961.1 CGLO|KAF3806164.1 CHIG|XP_018164495.1 CVIN|KAF4911576.1 CVYL|A07343 FGRM|XP_011325037.1 MGRA|XP_003851699.1 MLAR|XP_007412213.1 MORY|QBZ60568.1 NCRA|XP_959598.1 SSCL|APA14039.1

>Orthogroup2574: ANID|CBF76336.1 BCIN|XP_024548169.1 BGRA|VCU40991.1 CFRU|XP_031892864.1 CGLO|KAF3809167.1 CHIG|XP_018155989.1 CVIN|KAF4919817.1 CVYL|A01354 FGRM|XP_011318814.1 MGRA|XP_003857570.1 MLAR|XP_007411795.1 MORY|QBZ57475.1 NCRA|XP_011394478.1 SSCL|APA12421.1

>Orthogroup2575: ANID|CBF76356.1 BCIN|XP_001546921.2 BGRA|VDB95038.1 CFRU|XP_031890710.1 CGLO|KAF3801595.1 CHIG|XP_018151354.1 CVIN|KAF4922713.1 CVYL|A12540 FGRM|XP_011320349.1 MGRA|XP_003855541.1 MLAR|XP_007410630.1 MORY|QBZ55758.1 NCRA|XP_960217.1 SSCL|APA14843.1

>Orthogroup2576: ANID|CBF76372.1 BCIN|XP_001552419.1 BGRA|VDB92455.1 CFRU|XP_031890978.1 CGLO|KAF3801661.1 CHIG|XP_018151435.1 CVIN|KAF4921205.1 CVYL|A12474 FGRM|XP_011328506.1 MGRA|XP_003853668.1 MLAR|XP_007409600.1 MORY|QBZ62152.1 NCRA|XP_957538.3 SSCL|APA14888.1

>Orthogroup2577: ANID|CBF76374.1 BCIN|XP_024551566.1 BGRA|VDB89703.1 CFRU|XP_031890985.1 CGLO|KAF3801662.1 CHIG|XP_018151436.1 CVIN|KAF4921204.1 CVYL|A12473 FGRM|XP_011328505.1 MGRA|XP_003854612.1 MLAR|XP_007417982.1 MORY|QBZ62151.1 NCRA|XP_957540.1 SSCL|APA14889.1

>Orthogroup2578: ANID|CBF76378.1 BCIN|XP_024551805.1 BGRA|VDB95072.1 CFRU|XP_031890844.1 CGLO|KAF3801617.1 CHIG|XP_018151379.1 CVIN|KAF4922720.1 CVYL|A12519 FGRM|XP_011328202.1 MGRA|XP_003854677.1 MLAR|XP_007405391.1 MORY|QBZ53877.1 NCRA|XP_960378.1 SSCL|APA14667.1

>Orthogroup2579: ANID|CBF76388.1 BCIN|XP_024551689.1 BGRA|VDB89426.1 CFRU|XP_031883905.1 CGLO|KAF3805061.1 CHIG|XP_018163456.1 CVIN|KAF4902234.1 CVYL|A06499 FGRM|XP_011318755.1 MGRA|XP_003854721.1 MLAR|XP_007403546.1 MORY|QBZ59523.1 NCRA|XP_957490.2 SSCL|APA06021.1

>Orthogroup2580: ANID|CBF76402.1 BCIN|XP_024551729.1 BGRA|VDB92627.1 CFRU|XP_031884942.1 CGLO|KAF3801026.1 CHIG|XP_018153322.1 CVIN|KAF4889709.1 CVYL|A00358 FGRM|XP_011321550.1 MGRA|XP_003854947.1 MLAR|XP_007415009.1 MORY|QBZ54974.1 NCRA|XP_961765.2 SSCL|APA14584.1

>Orthogroup2581: ANID|CBF76410.1 BCIN|XP_001556673.1 BGRA|VDB92609.1 CFRU|XP_031881347.1 CGLO|KAF3805764.1 CHIG|XP_018163656.1 CVIN|KAF4926567.1 CVYL|A06052 FGRM|XP_011318722.1 MGRA|XP_003854724.1 MLAR|XP_007413627.1 MORY|QBZ59427.1 NCRA|XP_962546.1 SSCL|APA14796.1

>Orthogroup2582: ANID|CBF76417.1 BCIN|XP_001556657.2 BGRA|VCU39115.1 CFRU|XP_031884770.1 CGLO|KAF3800967.1 CHIG|XP_018153227.1 CVIN|KAF4913280.1 CVYL|A05938 FGRM|XP_011321609.1 MGRA|XP_003854812.1 MLAR|XP_007409720.1 MORY|QBZ59413.1 NCRA|XP_962098.1 SSCL|APA14786.1

>Orthogroup2583: ANID|CBF76427.1 BCIN|XP_001558507.1 BGRA|VDB95059.1 CFRU|XP_031890707.1 CGLO|KAF3801590.1 CHIG|XP_018151348.1 CVIN|KAF4922726.1 CVYL|A12545 FGRM|XP_011320344.1 MGRA|XP_003854742.1 MLAR|XP_007409194.1 MORY|QBZ55754.1 NCRA|XP_960221.1 SSCL|APA14695.1

>Orthogroup2584: ANID|CBF76451.1 BCIN|XP_024551659.1 BGRA|VDB92543.1 CFRU|XP_031884778.1 CGLO|KAF3800969.1 CHIG|XP_018153229.1 CVIN|KAF4913261.1 CVYL|A05936 FGRM|XP_011318743.1 MGRA|XP_003855733.1 MLAR|XP_007417184.1 MORY|QBZ59412.1 NCRA|XP_962096.1 SSCL|APA14783.1

>Orthogroup2585: ANID|CBF76480.1 BCIN|XP_001552090.1 BGRA|VDB89339.1 CFRU|XP_031881313.1 CGLO|KAF3802459.1 CHIG|XP_018154759.1 CVIN|KAF4916885.1 CVYL|A02050 FGRM|XP_011316537.1 MGRA|XP_003854791.1 MLAR|XP_007411420.1 MORY|QBZ59084.1 NCRA|XP_956724.1 SSCL|APA09818.1

>Orthogroup2586: ANID|CBF76482.1 BCIN|XP_001559891.2 BGRA|VDB89382.1 CFRU|XP_031881251.1 CGLO|KAF3802458.1 CHIG|XP_018154758.1 CVIN|KAF4916886.1 CVYL|A02049 FGRM|XP_011316538.1 MGRA|XP_003853565.1 MLAR|XP_007413863.1 MORY|QBZ59085.1 NCRA|XP_956725.3 SSCL|APA09738.1

>Orthogroup2587: ANID|CBF76486.1 BCIN|XP_001557401.1 BGRA|VCU41048.1 CFRU|XP_031885225.1 CGLO|KAF3807726.1 CHIG|XP_018162960.1 CVIN|KAF4920514.1 CVYL|A06909 FGRM|XP_011326203.1 MGRA|XP_003853563.1 MLAR|XP_007413294.1 MORY|QBZ63127.1 NCRA|XP_957729.1 SSCL|APA10675.1

>Orthogroup2588: ANID|CBF76488.1 BCIN|XP_024551177.1 BGRA|VCU39772.1 CFRU|XP_031886412.1 CGLO|KAF3806357.1 CHIG|XP_018162236.1 CVIN|KAF4923256.1 CVYL|A11946 FGRM|XP_011324045.1 MGRA|XP_003857299.1 MLAR|XP_007410838.1 MORY|QBZ61605.1 NCRA|XP_955952.1 SSCL|APA10172.1

>Orthogroup2589: ANID|CBF76513.1 BCIN|XP_001549886.1 BGRA|VDB84419.1 CFRU|XP_031885167.1 CGLO|KAF3800372.1 CHIG|XP_018162869.1 CVIN|KAF4927796.1 CVYL|A07797 FGRM|XP_011327041.1 MGRA|XP_003852787.1 MLAR|XP_007412179.1 MORY|QBZ61320.1 NCRA|XP_957309.1 SSCL|APA08229.1

>Orthogroup2590: ANID|CBF76523.1 BCIN|XP_001557574.1 BGRA|VCU40363.1 CFRU|XP_031880320.1 CGLO|KAF3808986.1 CHIG|XP_018155820.1 CVIN|KAF4920846.1 CVYL|A01335 FGRM|XP_011318843.1 MGRA|XP_003848118.1 MLAR|XP_007407838.1 MORY|QBZ62219.1 NCRA|XP_961091.1 SSCL|APA12329.1

>Orthogroup2591: ANID|CBF76540.1 BCIN|XP_001554357.2 BGRA|VDB83843.1 CFRU|XP_031882053.1 CGLO|KAF3806075.1 CHIG|XP_018164599.1 CVIN|KAF4893190.1 CVYL|A07439 FGRM|XP_011315999.1 MGRA|XP_003848575.1 MLAR|XP_007405159.1 MORY|QBZ62267.1 NCRA|XP_011395295.1 SSCL|APA14431.1

>Orthogroup2592: ANID|CBF76559.1 BCIN|XP_001546584.1 BGRA|VDB88986.1 CFRU|XP_031892249.1 CGLO|KAF3805575.1 CHIG|XP_018164726.1 CVIN|KAF4922351.1 CVYL|A07627 FGRM|XP_011324970.1 MGRA|XP_003848305.1 MLAR|XP_007406732.1 MORY|QBZ64919.1 NCRA|XP_958945.3 SSCL|APA14120.1

>Orthogroup2593: ANID|CBF76573.1 BCIN|XP_024547258.1 BGRA|VDB83808.1 CFRU|XP_031878539.1 CGLO|KAF3798605.1 CHIG|XP_018153932.1 CVIN|KAF4930161.1 CVYL|A04841 FGRM|XP_011324698.1 MGRA|XP_003848322.1 MLAR|XP_007412343.1 MORY|QBZ60867.1 NCRA|XP_959460.1 SSCL|APA14214.1

>Orthogroup2595: ANID|CBF76581.1 BCIN|XP_024547196.1 BGRA|VDB83748.1 CFRU|XP_031877358.1 CGLO|KAF3799871.1 CHIG|XP_018153822.1 CVIN|KAF4921712.1 CVYL|A09647 FGRM|XP_011325433.1 MGRA|XP_003851546.1 MLAR|XP_007410422.1 MORY|QBZ59973.1 NCRA|XP_011395193.1 SSCL|APA14123.1

>Orthogroup2596: ANID|CBF76597.1 BCIN|XP_024553255.1 BGRA|VCU39420.1 CFRU|XP_031888410.1 CGLO|KAF3803674.1 CHIG|XP_018163776.1 CVIN|KAF4928511.1 CVYL|A14604 FGRM|XP_011324167.1 MGRA|XP_003857331.1 MLAR|XP_007403549.1 MORY|QBZ54631.1 NCRA|XP_964494.2 SSCL|APA13589.1

>Orthogroup2598: ANID|CBF76666.1 BCIN|XP_001559179.1 BGRA|VCU39126.1 CFRU|XP_031892315.1 CGLO|KAF3797275.1 CHIG|XP_018157277.1 CVIN|KAF4889566.1 CVYL|A10415 FGRM|XP_011319915.1 MGRA|XP_003847765.1 MLAR|XP_007409703.1 MORY|QBZ66045.1 NCRA|XP_964918.2 SSCL|APA15765.1

>Orthogroup2599: ANID|CBF76680.1 BCIN|XP_024549358.1 BGRA|VCU39409.1 CFRU|XP_031892316.1 CGLO|KAF3797276.1 CHIG|XP_018157276.1 CVIN|KAF4889567.1 CVYL|A10414 FGRM|XP_011319917.1 MGRA|XP_003847725.1 MLAR|XP_007414357.1 MORY|QBZ66038.1 NCRA|XP_011393232.1 SSCL|APA15766.1

>Orthogroup2600: ANID|CBF76701.1 BCIN|XP_024549256.1 BGRA|VDB91359.1 CFRU|XP_031876006.1 CGLO|KAF3797310.1 CHIG|XP_018157201.1 CVIN|KAF4899569.1 CVYL|A10386 FGRM|XP_011320198.1 MGRA|XP_003847559.1 MLAR|XP_007408732.1 MORY|QBZ60385.1 NCRA|XP_964295.2 SSCL|APA08861.1

>Orthogroup2603: ANID|CBF76747.1 BCIN|XP_024545957.1 BGRA|VCU38937.1 CFRU|XP_031887890.1 CGLO|KAF3802157.1 CHIG|XP_018154281.1 CVIN|KAF4907206.1 CVYL|A03233 FGRM|XP_011320260.1 MGRA|XP_003847600.1 MLAR|XP_007404531.1 MORY|QBZ65760.1 NCRA|XP_964335.3 SSCL|APA07385.1

>Orthogroup2604: ANID|CBF76749.1 BCIN|XP_001546443.1 BGRA|VCU39035.1 CFRU|XP_031888056.1 CGLO|KAF3802271.1 CHIG|XP_018154364.1 CVIN|KAF4924437.1 CVYL|A10318 FGRM|XP_011319810.1 MGRA|XP_003847713.1 MLAR|XP_007413538.1 MORY|QBZ65758.1 NCRA|XP_964233.1 SSCL|APA07250.1

>Orthogroup2605: ANID|CBF76751.1 BCIN|XP_001546444.1 BGRA|VCU39036.1 CFRU|XP_031888057.1 CGLO|KAF3802270.1 CHIG|XP_018154365.1 CVIN|KAF4924430.1 CVYL|A10307 FGRM|XP_011319811.1 MGRA|XP_003847706.1 MLAR|XP_007413637.1 MORY|QBZ65757.1 NCRA|XP_964232.1 SSCL|APA07249.1

>Orthogroup2606: ANID|CBF76755.1 BCIN|XP_024546040.1 BGRA|VCU39087.1 CFRU|XP_031887964.1 CGLO|KAF3802229.1 CHIG|XP_018154333.1 CVIN|KAF4914860.1 CVYL|A03172 FGRM|XP_011319836.1 MGRA|XP_003847563.1 MLAR|XP_007407242.1 MORY|QBZ66017.1 NCRA|XP_964510.1 SSCL|APA07281.1

>Orthogroup2607: ANID|CBF76757.1 BCIN|XP_024546038.1 BGRA|VCU39079.1 CFRU|XP_031887962.1 CGLO|KAF3802231.1 CHIG|XP_018154335.1 CVIN|KAF4914871.1 CVYL|A03170 FGRM|XP_011319838.1 MGRA|XP_003847692.1 MLAR|XP_007407302.1 MORY|QBZ66019.1 NCRA|XP_011392947.1 SSCL|APA07283.1

>Orthogroup2608: ANID|CBF76763.1 BCIN|XP_024546041.1 BGRA|VDB86333.1 CFRU|XP_031892779.1 CGLO|KAF3797260.1 CHIG|XP_018157256.1 CVIN|KAF4920710.1 CVYL|A10433 FGRM|XP_011320019.1 MGRA|XP_003847632.1 MLAR|XP_007415062.1 MORY|QBZ66014.1 NCRA|XP_964252.1 SSCL|APA07280.1

>Orthogroup2610: ANID|CBF76788.1 BCIN|XP_024549308.1 BGRA|VCU39055.1 CFRU|XP_031881533.1 CGLO|KAF3805383.1 CHIG|XP_018156728.1 CVIN|KAF4886794.1 CVYL|A11030 FGRM|XP_011315795.1 MGRA|XP_003847643.1 MLAR|XP_007415066.1 MORY|QBZ65353.1 NCRA|XP_965735.3 SSCL|APA08933.1

>Orthogroup2611: ANID|CBF76798.1 BCIN|XP_024549198.1 BGRA|VCU39061.1 CFRU|XP_031881648.1 CGLO|KAF3807432.1 CHIG|XP_018156798.1 CVIN|KAF4918520.1 CVYL|A10935 FGRM|XP_011328308.1 MGRA|XP_003847759.1 MLAR|XP_007413015.1 MORY|QBZ66197.1 NCRA|XP_965223.2 SSCL|APA15733.1

>Orthogroup2612: ANID|CBF76802.1 BCIN|XP_001556267.1 BGRA|VDB86107.1 CFRU|XP_031880381.1 CGLO|KAF3807339.1 CHIG|XP_018156902.1 CVIN|KAF4920081.1 CVYL|A10816 FGRM|XP_011325071.1 MGRA|XP_003847709.1 MLAR|XP_007411417.1 MORY|QBZ65864.1 NCRA|XP_965758.1 SSCL|APA15743.1

>Orthogroup2613: ANID|CBF76806.1 BCIN|XP_024549188.1 BGRA|VDB86312.1 CFRU|XP_031881660.1 CGLO|KAF3807429.1 CHIG|XP_018156795.1 CVIN|KAF4918518.1 CVYL|A10933 FGRM|XP_011328305.1 MGRA|XP_003847615.1 MLAR|XP_007407655.1 MORY|QBZ66194.1 NCRA|XP_965423.1 SSCL|APA15720.1

>Orthogroup2614: ANID|CBF76818.1 BCIN|XP_001560224.2 BGRA|VDB86078.1 CFRU|XP_031881556.1 CGLO|KAF3807446.1 CHIG|XP_018156784.1 CVIN|KAF4918530.1 CVYL|A10954 FGRM|XP_011328288.1 MGRA|XP_003847691.1 MLAR|XP_007410830.1 MORY|QBZ66082.1 NCRA|XP_964726.1 SSCL|APA15927.1

>Orthogroup2616: ANID|CBF76839.1 BCIN|XP_001548517.1 BGRA|VCU39092.1 CFRU|XP_031892396.1 CGLO|KAF3807209.1 CHIG|XP_018157015.1 CVIN|KAF4928194.1 CVYL|A10687 FGRM|XP_011320228.1 MGRA|XP_003847606.1 MLAR|XP_007414943.1 MORY|QBZ60441.1 NCRA|XP_964881.1 SSCL|APA07238.1

>Orthogroup2617: ANID|CBF76842.1 BCIN|XP_001548518.1 BGRA|VCU39090.1 CFRU|XP_031888119.1 CGLO|KAF3802303.1 CHIG|XP_018154407.1 CVIN|KAF4896253.1 CVYL|A10718 FGRM|XP_011319760.1 MGRA|XP_003847761.1 MLAR|XP_007415680.1 MORY|QBZ65756.1 NCRA|XP_011392896.1 SSCL|APA07237.1

>Orthogroup2618: ANID|CBF76850.1 BCIN|XP_001545869.1 BGRA|VCU39082.1 CFRU|XP_031888177.1 CGLO|KAF3802233.1 CHIG|XP_018154337.1 CVIN|KAF4914857.1 CVYL|A03168 FGRM|XP_011319843.1 MGRA|XP_003847548.1 MLAR|XP_007413656.1 MORY|QBZ66021.1 NCRA|XP_964516.1 SSCL|APA07285.1

>Orthogroup2619: ANID|CBF76862.1 BCIN|XP_024546045.1 BGRA|VCU38855.1 CFRU|XP_031888024.1 CGLO|KAF3802236.1 CHIG|XP_018154343.1 CVIN|KAF4914848.1 CVYL|A03163 FGRM|XP_011319852.1 MGRA|XP_003847613.1 MLAR|XP_007407061.1 MORY|QBZ66026.1 NCRA|XP_964521.2 SSCL|APA07277.1

>Orthogroup2620: ANID|CBF76872.1 BCIN|XP_001545882.1 BGRA|VCU39089.1 CFRU|XP_031887856.1 CGLO|KAF3802240.1 CHIG|XP_018154347.1 CVIN|KAF4914843.1 CVYL|A03159 FGRM|XP_011319856.1 MGRA|XP_003847525.1 MLAR|XP_007414605.1 MORY|QBZ66029.1 NCRA|XP_964518.1 SSCL|APA07273.1

>Orthogroup2621: ANID|CBF76887.1 BCIN|XP_024546057.1 BGRA|VCU39024.1 CFRU|XP_031888206.1 CGLO|KAF3802263.1 CHIG|XP_018154372.1 CVIN|KAF4924401.1 CVYL|A10230 FGRM|XP_011319821.1 MGRA|XP_003847519.1 MLAR|XP_007408213.1 MORY|QBZ60376.1 NCRA|XP_964339.3 SSCL|APA07262.1

>Orthogroup2622: ANID|CBF76889.1 BCIN|XP_024546055.1 BGRA|VCU39770.1 CFRU|XP_031880335.1 CGLO|KAF3807287.1 CHIG|XP_018156969.1 CVIN|KAF4918076.1 CVYL|A10786 FGRM|XP_011328277.1 MGRA|XP_003847751.1 MLAR|XP_007408871.1 MORY|QBZ65676.1 NCRA|XP_964572.1 SSCL|APA07263.1

>Orthogroup2623: ANID|CBF76895.1 BCIN|XP_001546435.1 BGRA|VCU39022.1 CFRU|XP_031888200.1 CGLO|KAF3802265.1 CHIG|XP_018154370.1 CVIN|KAF4924403.1 CVYL|A10252 FGRM|XP_011319819.1 MGRA|XP_003847735.1 MLAR|XP_007405156.1 MORY|QBZ65717.1 NCRA|XP_011392913.1 SSCL|APA07260.1

>Orthogroup2624: ANID|CBF76904.1 BCIN|XP_024551988.1 BGRA|VCU39285.1 CFRU|XP_031887539.1 CGLO|KAF3803293.1 CHIG|XP_018161143.1 CVIN|KAF4928631.1 CVYL|A03728 FGRM|XP_011327968.1 MGRA|XP_003849543.1 MLAR|XP_007413729.1 MORY|QBZ64568.1 NCRA|XP_961888.1 SSCL|APA12671.1

>Orthogroup2625: ANID|CBF76906.1 BCIN|XP_001557952.1 BGRA|VCU39286.1 CFRU|XP_031887473.1 CGLO|KAF3803294.1 CHIG|XP_018161144.1 CVIN|KAF4928630.1 CVYL|A03730 FGRM|XP_011327969.1 MGRA|XP_003849565.1 MLAR|XP_007416588.1 MORY|QBZ64569.1 NCRA|XP_961887.1 SSCL|APA12670.1

>Orthogroup2626: ANID|CBF76912.1 BCIN|XP_001554621.1 BGRA|VDB85988.1 CFRU|XP_031880421.1 CGLO|KAF3807310.1 CHIG|XP_018156984.1 CVIN|KAF4918089.1 CVYL|A10797 FGRM|XP_011315902.1 MGRA|XP_003854577.1 MLAR|XP_007406315.1 MORY|QBZ65871.1 NCRA|XP_965754.1 SSCL|APA11055.1

>Orthogroup2627: ANID|CBF76920.1 BCIN|XP_001550314.1 BGRA|VDB92832.1 CFRU|XP_031878646.1 CGLO|KAF3798518.1 CHIG|XP_018161195.1 CVIN|KAF4917469.1 CVYL|A03808 FGRM|XP_011328019.1 MGRA|XP_003849261.1 MLAR|XP_007403547.1 MORY|QBZ57192.1 NCRA|XP_962525.2 SSCL|APA12804.1

>Orthogroup2630: ANID|CBF76936.1 BCIN|XP_001553998.2 BGRA|VDB91215.1 CFRU|XP_031881593.1 CGLO|KAF3807386.1 CHIG|XP_018156839.1 CVIN|KAF4929651.1 CVYL|A10879 FGRM|XP_011328315.1 MGRA|XP_003853982.1 MLAR|XP_007404501.1 MORY|QBZ65877.1 NCRA|XP_965749.1 SSCL|APA11100.1

>Orthogroup2631: ANID|CBF76940.1 BCIN|XP_024550366.1 BGRA|VDB84148.1 CFRU|XP_031880439.1 CGLO|KAF3807305.1 CHIG|XP_018156979.1 CVIN|KAF4918074.1 CVYL|A10791 FGRM|XP_011328328.1 MGRA|XP_003853765.1 MLAR|XP_007413818.1 MORY|QBZ65880.1 NCRA|XP_965745.1 SSCL|APA11059.1

>Orthogroup2632: ANID|CBF76955.1 BCIN|XP_001556998.1 BGRA|VDB85942.1 CFRU|XP_031880333.1 CGLO|KAF3807309.1 CHIG|XP_018156983.1 CVIN|KAF4918088.1 CVYL|A10795 FGRM|XP_011315805.1 MGRA|XP_003854159.1 MLAR|XP_007412061.1 MORY|QBZ65922.1 NCRA|XP_965804.1 SSCL|APA10810.1

>Orthogroup2634: ANID|CBF76969.1 BCIN|XP_001560590.1 BGRA|VDB85827.1 CFRU|XP_031888041.1 CGLO|KAF3802062.1 CHIG|XP_018154194.1 CVIN|KAF4917973.1 CVYL|A03323 FGRM|XP_011323005.1 MGRA|XP_003854422.1 MLAR|XP_007409974.1 MORY|QBZ66400.1 NCRA|XP_011393153.1 SSCL|APA07914.1

>Orthogroup2636: ANID|CBF76998.1 BCIN|XP_001552386.1 BGRA|VDB84457.1 CFRU|XP_031877738.1 CGLO|KAF3797333.1 CHIG|XP_018157175.1 CVIN|KAF4917662.1 CVYL|A10360 FGRM|XP_011320251.1 MGRA|XP_003853729.1 MLAR|XP_007415558.1 MORY|QBZ66277.1 NCRA|XP_964285.3 SSCL|APA11202.1

>Orthogroup2637: ANID|CBF77006.1 BCIN|XP_024550324.1 BGRA|VDB85950.1 CFRU|XP_031892471.1 CGLO|KAF3797090.1 CHIG|XP_018157059.1 CVIN|KAF4911395.1 CVYL|A10619 FGRM|XP_011315806.1 MGRA|XP_003854514.1 MLAR|XP_007416370.1 MORY|QBZ65921.1 NCRA|XP_965809.1 SSCL|APA10994.1

>Orthogroup2638: ANID|CBF77008.1 BCIN|XP_024550323.1 BGRA|VDB85949.1 CFRU|XP_031892463.1 CGLO|KAF3797091.1 CHIG|XP_018157060.1 CVIN|KAF4911380.1 CVYL|A10617 FGRM|XP_011315807.1 MGRA|XP_003853750.1 MLAR|XP_007410684.1 MORY|QBZ65920.1 NCRA|XP_965810.3 SSCL|APA10993.1

>Orthogroup2639: ANID|CBF77016.1 BCIN|XP_024550400.1 BGRA|VDB85806.1 CFRU|XP_031888095.1 CGLO|KAF3802284.1 CHIG|XP_018154383.1 CVIN|KAF4924405.1 CVYL|A10485 FGRM|XP_011319787.1 MGRA|XP_003854529.1 MLAR|XP_007414939.1 MORY|QBZ65800.1 NCRA|XP_964120.1 SSCL|APA11095.1

>Orthogroup2640: ANID|CBF77033.1 BCIN|XP_024550571.1 BGRA|VDB85953.1 CFRU|XP_031891298.1 CGLO|KAF3797624.1 CHIG|XP_018157416.1 CVIN|KAF4929428.1 CVYL|A11076 FGRM|XP_011323012.1 MGRA|XP_003854187.1 MLAR|XP_007415959.1 MORY|QBZ66401.1 NCRA|XP_959152.1 SSCL|APA11366.1

>Orthogroup2642: ANID|CBF77056.1 BCIN|XP_001550855.1 BGRA|VDB89598.1 CFRU|XP_031876401.1 CGLO|KAF3797792.1 CHIG|XP_018156469.1 CVIN|KAF4923152.1 CVYL|A03017 FGRM|XP_011318570.1 MGRA|XP_003851666.1 MLAR|XP_007417981.1 MORY|QBZ55897.1 NCRA|XP_960038.1 SSCL|APA12426.1

>Orthogroup2643: ANID|CBF77062.1 BCIN|XP_001547838.1 BGRA|VCU40859.1 CFRU|XP_031875748.1 CGLO|KAF3797764.1 CHIG|XP_018156433.1 CVIN|KAF4918800.1 CVYL|A02980 FGRM|XP_011318782.1 MGRA|XP_003856143.1 MLAR|XP_007410995.1 MORY|QBZ57719.1 NCRA|XP_960073.1 SSCL|APA11552.1

>Orthogroup2644: ANID|CBF77071.1 BCIN|XP_001547832.1 BGRA|VCU40860.1 CFRU|XP_031880313.1 CGLO|KAF3808995.1 CHIG|XP_018155838.1 CVIN|KAF4918027.1 CVYL|A01424 FGRM|XP_011318989.1 MGRA|XP_003857547.1 MLAR|XP_007412338.1 MORY|QBZ61112.1 NCRA|XP_958041.1 SSCL|APA11556.1

>Orthogroup2645: ANID|CBF77090.1 BCIN|XP_024549207.1 BGRA|VCU38839.1 CFRU|XP_031877696.1 CGLO|KAF3797339.1 CHIG|XP_018157172.1 CVIN|KAF4917668.1 CVYL|A10353 FGRM|XP_011320030.1 MGRA|XP_003847609.1 MLAR|XP_007410067.1 MORY|QBZ65861.1 NCRA|XP_965761.2 SSCL|APA15738.1

>Orthogroup2647: ANID|CBF77141.1 BCIN|XP_024549065.1 BGRA|VCU38845.1 CFRU|XP_031877718.1 CGLO|KAF3797352.1 CHIG|XP_018157159.1 CVIN|KAF4889985.1 CVYL|A10338 FGRM|XP_011320098.1 MGRA|XP_003847827.1 MLAR|XP_007404436.1 MORY|QBZ65896.1 NCRA|XP_965832.1 SSCL|APA15846.1

>Orthogroup2648: ANID|CBF77146.1 BCIN|XP_001560203.1 BGRA|VDB86259.1 CFRU|XP_031881560.1 CGLO|KAF3807439.1 CHIG|XP_018156788.1 CVIN|KAF4918540.1 CVYL|A10943 FGRM|XP_011328284.1 MGRA|XP_003847555.1 MLAR|XP_007416928.1 MORY|QBZ65850.1 NCRA|XP_965739.1 SSCL|APA15901.1

>Orthogroup2649: ANID|CBF77152.1 BCIN|XP_001560254.1 BGRA|VDB86323.1 CFRU|XP_031880443.1 CGLO|KAF3807308.1 CHIG|XP_018156982.1 CVIN|KAF4918091.1 CVYL|A10794 FGRM|XP_011315780.1 MGRA|XP_003847744.1 MLAR|XP_007407621.1 MORY|QBZ65669.1 NCRA|XP_964725.1 SSCL|APA15948.1

>Orthogroup2651: ANID|CBF77185.1 BCIN|XP_001545455.1 BGRA|VDB83740.1 CFRU|XP_031878486.1 CGLO|KAF3798636.1 CHIG|XP_018153909.1 CVIN|KAF4912449.1 CVYL|A04814 FGRM|XP_011324038.1 MGRA|XP_003848554.1 MLAR|XP_007404175.1 MORY|QBZ60792.1 NCRA|XP_959239.1 SSCL|APA14199.1

>Orthogroup2652: ANID|CBF77187.1 BCIN|XP_001545454.2 BGRA|VDB83738.1 CFRU|XP_031878515.1 CGLO|KAF3798637.1 CHIG|XP_018153908.1 CVIN|KAF4912443.1 CVYL|A04813 FGRM|XP_011324689.1 MGRA|XP_003848429.1 MLAR|XP_007404429.1 MORY|QBZ60793.1 NCRA|XP_959240.1 SSCL|APA14198.1

>Orthogroup2653: ANID|CBF77195.1 BCIN|XP_001545872.1 BGRA|VCU39078.1 CFRU|XP_031887965.1 CGLO|KAF3802230.1 CHIG|XP_018154334.1 CVIN|KAF4914861.1 CVYL|A03171 FGRM|XP_011319837.1 MGRA|XP_003847785.1 MLAR|XP_007406003.1 MORY|QBZ66018.1 NCRA|XP_961283.1 SSCL|APA07282.1

>Orthogroup2654: ANID|CBF77209.1 BCIN|XP_024550715.1 BGRA|VDB93219.1 CFRU|XP_031887257.1 CGLO|KAF3809269.1 CHIG|XP_018156104.1 CVIN|KAF4907389.1 CVYL|A01462 FGRM|XP_011319319.1 MGRA|XP_003857610.1 MLAR|XP_007405724.1 MORY|QBZ56931.1 NCRA|XP_955863.1 SSCL|APA11560.1

>Orthogroup2656: ANID|CBF77234.1 BCIN|XP_024550708.1 BGRA|VCU40882.1 CFRU|XP_031887232.1 CGLO|KAF3809301.1 CHIG|XP_018156134.1 CVIN|KAF4911371.1 CVYL|A01497 FGRM|XP_011318825.1 MGRA|XP_003857571.1 MLAR|XP_007404730.1 MORY|QBZ62230.1 NCRA|XP_957595.1 SSCL|APA11545.1

>Orthogroup2657: ANID|CBF77238.1 BCIN|XP_001546645.1 BGRA|VCU40888.1 CFRU|XP_031889252.1 CGLO|KAF3808719.1 CHIG|XP_018155547.1 CVIN|KAF4929566.1 CVYL|A00941 FGRM|XP_011319272.1 MGRA|XP_003856041.1 MLAR|XP_007407126.1 MORY|QBZ59253.1 NCRA|XP_958039.1 SSCL|APA11539.1

>Orthogroup2659: ANID|CBF77248.1 BCIN|XP_024549208.1 BGRA|VCU38838.1 CFRU|XP_031877679.1 CGLO|KAF3797340.1 CHIG|XP_018157170.1 CVIN|KAF4917651.1 CVYL|A10351 FGRM|XP_011320031.1 MGRA|XP_003847729.1 MLAR|XP_007407762.1 MORY|QBZ65859.1 NCRA|XP_965762.3 SSCL|APA15739.1

>Orthogroup2660: ANID|CBF77256.1 BCIN|XP_001545585.2 BGRA|VCU41088.1 CFRU|XP_031886717.1 CGLO|KAF3804085.1 CHIG|XP_018162683.1 CVIN|KAF4919553.1 CVYL|A07045 FGRM|XP_011326361.1 MGRA|XP_003852110.1 MLAR|XP_007414647.1 MORY|QBZ55651.1 NCRA|XP_961999.3 SSCL|APA10206.1

>Orthogroup2661: ANID|CBF77258.1 BCIN|XP_001551426.1 BGRA|VDB88945.1 CFRU|XP_031886753.1 CGLO|KAF3804086.1 CHIG|XP_018162682.1 CVIN|KAF4919552.1 CVYL|A07046 FGRM|XP_011326360.1 MGRA|XP_003852708.1 MLAR|XP_007416648.1 MORY|QBZ55650.1 NCRA|XP_956385.1 SSCL|APA10347.1

>Orthogroup2662: ANID|CBF77262.1 BCIN|XP_001559267.1 BGRA|VDB84432.1 CFRU|XP_031886963.1 CGLO|KAF3811304.1 CHIG|XP_018160528.1 CVIN|KAF4927265.1 CVYL|A04482 FGRM|XP_011326470.1 MGRA|XP_003853091.1 MLAR|XP_007410039.1 MORY|QBZ57996.1 NCRA|XP_956756.1 SSCL|APA07845.1

>Orthogroup2664: ANID|CBF77288.1 BCIN|XP_001551361.1 BGRA|VCU39503.1 CFRU|XP_031878400.1 CGLO|KAF3810730.1 CHIG|XP_018162590.1 CVIN|KAF4932126.1 CVYL|A07222 FGRM|XP_011323966.1 MGRA|XP_003852665.1 MLAR|XP_007404497.1 MORY|QBZ63900.1 NCRA|XP_956172.1 SSCL|APA10403.1

>Orthogroup2665: ANID|CBF77290.1 BCIN|XP_024551526.1 BGRA|VCU41096.1 CFRU|XP_031886723.1 CGLO|KAF3804083.1 CHIG|XP_018162687.1 CVIN|KAF4919547.1 CVYL|A07043 FGRM|XP_011326365.1 MGRA|XP_003856282.1 MLAR|XP_007410849.1 MORY|QBZ55653.1 NCRA|XP_956383.3 SSCL|APA10216.1

>Orthogroup2666: ANID|CBF77302.1 BCIN|XP_024551347.1 BGRA|VDB89100.1 CFRU|XP_031886599.1 CGLO|KAF3804112.1 CHIG|XP_018162640.1 CVIN|KAF4932026.1 CVYL|A07078 FGRM|XP_011326649.1 MGRA|XP_003852843.1 MLAR|XP_007418572.1 MORY|QBZ59592.1 NCRA|XP_963100.1 SSCL|APA10515.1

>Orthogroup2667: ANID|CBF77304.1 BCIN|XP_024551349.1 BGRA|VDB89102.1 CFRU|XP_031886766.1 CGLO|KAF3804111.1 CHIG|XP_018162642.1 CVIN|KAF4932025.1 CVYL|A07077 FGRM|XP_011326648.1 MGRA|XP_003852842.1 MLAR|XP_007410968.1 MORY|QBZ59594.1 NCRA|XP_963101.1 SSCL|APA10514.1

>Orthogroup2668: ANID|CBF77310.1 BCIN|XP_001548061.2 BGRA|VDB94931.1 CFRU|XP_031886773.1 CGLO|KAF3804121.1 CHIG|XP_018162625.1 CVIN|KAF4932120.1 CVYL|A07086 FGRM|XP_011326653.1 MGRA|XP_003856457.1 MLAR|XP_007404350.1 MORY|QBZ59591.1 NCRA|XP_011393900.1 SSCL|APA10522.1

>Orthogroup2669: ANID|CBF77316.1 BCIN|XP_024552640.1 BGRA|VDB93078.1 CFRU|XP_031891366.1 CGLO|KAF3797917.1 CHIG|XP_018161603.1 CVIN|KAF4907840.1 CVYL|A05352 FGRM|XP_011318656.1 MGRA|XP_003855896.1 MLAR|XP_007417052.1 MORY|QBZ61997.1 NCRA|XP_959071.1 SSCL|APA15594.1

>Orthogroup2670: ANID|CBF77328.1 BCIN|XP_001554600.1 BGRA|VDB94880.1 CFRU|XP_031891373.1 CGLO|KAF3797902.1 CHIG|XP_018161617.1 CVIN|KAF4905679.1 CVYL|A05339 FGRM|XP_011318667.1 MGRA|XP_003857758.1 MLAR|XP_007408438.1 MORY|QBZ62037.1 NCRA|XP_963051.1 SSCL|APA11069.1

>Orthogroup2671: ANID|CBF77339.1 BCIN|XP_001560061.1 BGRA|VDB83886.1 CFRU|XP_031884513.1 CGLO|KAF3799675.1 CHIG|XP_018158265.1 CVIN|KAF4895369.1 CVYL|A05840 FGRM|XP_011316506.1 MGRA|XP_003855925.1 MLAR|XP_007409626.1 MORY|QBZ56710.1 NCRA|XP_964841.3 SSCL|APA09912.1

>Orthogroup2672: ANID|CBF77343.1 BCIN|XP_001559931.2 BGRA|VDB92865.1 CFRU|XP_031884602.1 CGLO|KAF3799724.1 CHIG|XP_018158252.1 CVIN|KAF4919976.1 CVYL|A05883 FGRM|XP_011328651.1 MGRA|XP_003855924.1 MLAR|XP_007407004.1 MORY|QBZ64461.1 NCRA|XP_964840.1 SSCL|APA09764.1

>Orthogroup2673: ANID|CBF77349.1 BCIN|XP_001554614.1 BGRA|VDB94746.1 CFRU|XP_031891382.1 CGLO|KAF3804720.1 CHIG|XP_018161793.1 CVIN|KAF4923371.1 CVYL|A05401 FGRM|XP_011321478.1 MGRA|XP_003855921.1 MLAR|XP_007411598.1 MORY|QBZ59682.1 NCRA|XP_011393977.1 SSCL|APA11063.1

>Orthogroup2674: ANID|CBF77356.1 BCIN|XP_001560063.1 BGRA|VCU40291.1 CFRU|XP_031890133.1 CGLO|KAF3810385.1 CHIG|XP_018158375.1 CVIN|KAF4909105.1 CVYL|A05727 FGRM|XP_011316528.1 MGRA|XP_003857798.1 MLAR|XP_007417322.1 MORY|QBZ64449.1 NCRA|XP_958022.2 SSCL|APA09911.1

>Orthogroup2676: ANID|CBF77390.1 BCIN|XP_024552927.1 BGRA|VCU41151.1 CFRU|XP_031882478.1 CGLO|KAF3807571.1 CHIG|XP_018156363.1 CVIN|KAF4928925.1 CVYL|A02911 FGRM|XP_011318940.1 MGRA|XP_003857658.1 MLAR|XP_007417844.1 MORY|QBZ57447.1 NCRA|XP_960881.3 SSCL|APA07543.1

>Orthogroup2677: ANID|CBF77394.1 BCIN|XP_001560050.1 BGRA|VCU40271.1 CFRU|XP_031884508.1 CGLO|KAF3799679.1 CHIG|XP_018158271.1 CVIN|KAF4919993.1 CVYL|A05843 FGRM|XP_011316515.1 MGRA|XP_003855882.1 MLAR|XP_007409323.1 MORY|QBZ59227.1 NCRA|XP_965587.1 SSCL|APA09920.1

>Orthogroup2678: ANID|CBF77398.1 BCIN|XP_024548694.1 BGRA|VCU40257.1 CFRU|XP_031884655.1 CGLO|KAF3810308.1 CHIG|XP_018158332.1 CVIN|KAF4913299.1 CVYL|A05796 FGRM|XP_011316492.1 MGRA|XP_003855834.1 MLAR|XP_007417123.1 MORY|QBZ58457.1 NCRA|XP_964366.2 SSCL|APA09922.1

>Orthogroup2679: ANID|CBF77402.1 BCIN|XP_001559750.2 BGRA|VCU40258.1 CFRU|XP_031884473.1 CGLO|KAF3799071.1 CHIG|XP_018158188.1 CVIN|KAF4918244.1 CVYL|A11459 FGRM|XP_011328386.1 MGRA|XP_003855907.1 MLAR|XP_007408808.1 MORY|QBZ64475.1 NCRA|XP_965625.3 SSCL|APA09484.1

>Orthogroup2680: ANID|CBF77416.1 BCIN|XP_001548642.1 BGRA|VDB83876.1 CFRU|XP_031890148.1 CGLO|KAF3802800.1 CHIG|XP_018158422.1 CVIN|KAF4908248.1 CVYL|A11379 FGRM|XP_011316497.1 MGRA|XP_003857754.1 MLAR|XP_007407787.1 MORY|QBZ60274.1 NCRA|XP_011393257.1 SSCL|APA09634.1

>Orthogroup2682: ANID|CBF77432.1 BCIN|XP_024546616.1 BGRA|VDB90642.1 CFRU|XP_031888518.1 CGLO|KAF3803732.1 CHIG|XP_018153181.1 CVIN|KAF4928547.1 CVYL|A14668 FGRM|XP_011316842.1 MGRA|XP_003851135.1 MLAR|XP_007408903.1 MORY|QBZ59791.1 NCRA|XP_965639.1 SSCL|APA06450.1

>Orthogroup2683: ANID|CBF77434.1 BCIN|XP_001553621.1 BGRA|VDB90640.1 CFRU|XP_031888517.1 CGLO|KAF3803733.1 CHIG|XP_018153180.1 CVIN|KAF4928548.1 CVYL|A14115 FGRM|XP_011316841.1 MGRA|XP_003850717.1 MLAR|XP_007406729.1 MORY|QBZ59790.1 NCRA|XP_965640.2 SSCL|APA06449.1

>Orthogroup2684: ANID|CBF77444.1 BCIN|XP_001545413.1 BGRA|VDB90560.1 CFRU|XP_031888541.1 CGLO|KAF3803558.1 CHIG|XP_018164131.1 CVIN|KAF4922293.1 CVYL|A14490 FGRM|XP_011326496.1 MGRA|XP_003850600.1 MLAR|XP_007416668.1 MORY|QBZ56413.1 NCRA|XP_964466.3 SSCL|APA06473.1

>Orthogroup2686: ANID|CBF77453.1 BCIN|XP_001555179.1 BGRA|VCU40011.1 CFRU|XP_031882251.1 CGLO|KAF3804446.1 CHIG|XP_018160272.1 CVIN|KAF4930919.1 CVYL|A04155 FGRM|XP_011324507.1 MGRA|XP_003850673.1 MLAR|XP_007404582.1 MORY|QBZ53829.1 NCRA|XP_011394498.1 SSCL|APA09162.1

>Orthogroup2687: ANID|CBF77455.1 BCIN|XP_001555182.1 BGRA|VCU39566.1 CFRU|XP_031880749.1 CGLO|KAF3811202.1 CHIG|XP_018160447.1 CVIN|KAF4931205.1 CVYL|A04368 FGRM|XP_011326475.1 MGRA|XP_003850687.1 MLAR|XP_007410960.1 MORY|QBZ53850.1 NCRA|XP_011394583.1 SSCL|APA09174.1

>Orthogroup2688: ANID|CBF77467.1 BCIN|XP_024546610.1 BGRA|VDB90654.1 CFRU|XP_031888369.1 CGLO|KAF3803598.1 CHIG|XP_018163867.1 CVIN|KAF4920351.1 CVYL|A14533 FGRM|XP_011316711.1 MGRA|XP_003850639.1 MLAR|XP_007404117.1 MORY|QBZ55364.1 NCRA|XP_964054.1 SSCL|APA06453.1

>Orthogroup2689: ANID|CBF77469.1 BCIN|XP_024546643.1 BGRA|VDB90570.1 CFRU|XP_031884188.1 CGLO|KAF3803765.1 CHIG|XP_018153150.1 CVIN|KAF4917896.1 CVYL|A14145 FGRM|XP_011324181.1 MGRA|XP_003850656.1 MLAR|XP_007414716.1 MORY|QBZ59805.1 NCRA|XP_965641.1 SSCL|APA06476.1

>Orthogroup2690: ANID|CBF77479.1 BCIN|XP_024548549.1 BGRA|VCU39569.1 CFRU|XP_031882164.1 CGLO|KAF3804472.1 CHIG|XP_018160307.1 CVIN|KAF4930990.1 CVYL|A04187 FGRM|XP_011326440.1 MGRA|XP_003851249.1 MLAR|XP_007407977.1 MORY|QBZ57019.1 NCRA|XP_958766.2 SSCL|APA10075.1

>Orthogroup2691: ANID|CBF77490.1 BCIN|XP_001553576.1 BGRA|VDB90720.1 CFRU|XP_031890602.1 CGLO|KAF3804823.1 CHIG|XP_018164317.1 CVIN|KAF4929776.1 CVYL|A12251 FGRM|XP_011317013.1 MGRA|XP_003851215.1 MLAR|XP_007415443.1 MORY|QBZ60574.1 NCRA|XP_964479.1 SSCL|APA06413.1

>Orthogroup2692: ANID|CBF77496.1 BCIN|XP_024546590.1 BGRA|VDB90722.1 CFRU|XP_031890594.1 CGLO|KAF3804821.1 CHIG|XP_018164319.1 CVIN|KAF4929793.1 CVYL|A12253 FGRM|XP_011317011.1 MGRA|XP_003850602.1 MLAR|XP_007405038.1 MORY|QBZ63554.1 NCRA|XP_964481.3 SSCL|APA06415.1

>Orthogroup2694: ANID|CBF77508.1 BCIN|XP_001549321.1 BGRA|VCU40385.1 CFRU|XP_031877818.1 CGLO|KAF3806611.1 CHIG|XP_018164244.1 CVIN|KAF4929824.1 CVYL|A12320 FGRM|XP_011325570.1 MGRA|XP_003848000.1 MLAR|XP_007415965.1 MORY|QBZ54022.1 NCRA|XP_957152.1 SSCL|APA12118.1

>Orthogroup2698: ANID|CBF77573.1 BCIN|XP_024548193.1 BGRA|VCU40729.1 CFRU|XP_031890822.1 CGLO|KAF3811687.1 CHIG|XP_018151825.1 CVIN|KAF4915940.1 CVYL|A12710 FGRM|XP_011319396.1 MGRA|XP_003847843.1 MLAR|XP_007411147.1 MORY|QBZ61890.1 NCRA|XP_959445.3 SSCL|APA12445.1

>Orthogroup2699: ANID|CBF77576.1 BCIN|XP_024548254.1 BGRA|VDB93225.1 CFRU|XP_031885508.1 CGLO|KAF3799505.1 CHIG|XP_018154491.1 CVIN|KAF4931870.1 CVYL|A13366 FGRM|XP_011321246.1 MGRA|XP_003848098.1 MLAR|XP_007416325.1 MORY|QBZ57277.1 NCRA|XP_011393447.1 SSCL|APA12517.1

>Orthogroup2700: ANID|CBF77580.1 BCIN|XP_024548197.1 BGRA|VCU40725.1 CFRU|XP_031883543.1 CGLO|KAF3800189.1 CHIG|XP_018155105.1 CVIN|KAF4897008.1 CVYL|A06099 FGRM|XP_011317481.1 MGRA|XP_003848028.1 MLAR|XP_007415929.1 MORY|QBZ64820.1 NCRA|XP_956298.1 SSCL|APA12450.1

>Orthogroup2702: ANID|CBF77594.1 BCIN|XP_001557568.1 BGRA|VCU40357.1 CFRU|XP_031889210.1 CGLO|KAF3808793.1 CHIG|XP_018155691.1 CVIN|KAF4906811.1 CVYL|A00872 FGRM|XP_011319343.1 MGRA|XP_003847937.1 MLAR|XP_007417700.1 MORY|QBZ58933.1 NCRA|XP_960609.1 SSCL|APA12332.1

>Orthogroup2704: ANID|CBF77602.1 BCIN|XP_001557576.1 BGRA|VCU40361.1 CFRU|XP_031880319.1 CGLO|KAF3808987.1 CHIG|XP_018155818.1 CVIN|KAF4920866.1 CVYL|A01357 FGRM|XP_011318841.1 MGRA|XP_003848200.1 MLAR|XP_007407452.1 MORY|QBZ62221.1 NCRA|XP_960949.1 SSCL|APA12327.1

>Orthogroup2706: ANID|CBF77610.1 BCIN|XP_001550905.1 BGRA|VCU41004.1 CFRU|XP_031889208.1 CGLO|KAF3808787.1 CHIG|XP_018155698.1 CVIN|KAF4906821.1 CVYL|A00878 FGRM|XP_011319331.1 MGRA|XP_003848177.1 MLAR|XP_007403916.1 MORY|QBZ58955.1 NCRA|XP_960802.3 SSCL|APA12390.1

>Orthogroup2708: ANID|CBF77627.1 BCIN|XP_001550911.2 BGRA|VDB93108.1 CFRU|XP_031889424.1 CGLO|KAF3808800.1 CHIG|XP_018155705.1 CVIN|KAF4906817.1 CVYL|A00864 FGRM|XP_011319252.1 MGRA|XP_003848179.1 MLAR|XP_007414619.1 MORY|QBZ58944.1 NCRA|XP_960807.1 SSCL|APA12386.1

>Orthogroup2709: ANID|CBF77645.1 BCIN|XP_001549288.1 BGRA|VCU40425.1 CFRU|XP_031882641.1 CGLO|KAF3800484.1 CHIG|XP_018152277.1 CVIN|KAF4906668.1 CVYL|A13229 FGRM|XP_011323678.1 MGRA|XP_003848109.1 MLAR|XP_007404079.1 MORY|QBZ54410.1 NCRA|XP_956818.1 SSCL|APA12106.1

>Orthogroup2710: ANID|CBF77666.1 BCIN|XP_001557845.2 BGRA|VCU40383.1 CFRU|XP_031890867.1 CGLO|KAF3801449.1 CHIG|XP_018151895.1 CVIN|KAF4912399.1 CVYL|A12677 FGRM|XP_011325497.1 MGRA|XP_003848189.1 MLAR|XP_007408960.1 MORY|QBZ63156.1 NCRA|XP_011393647.1 SSCL|APA12119.1

>Orthogroup2711: ANID|CBF77688.1 BCIN|XP_001557302.1 BGRA|VCU40601.1 CFRU|XP_031885358.1 CGLO|KAF3799513.1 CHIG|XP_018154501.1 CVIN|KAF4931756.1 CVYL|A13374 FGRM|XP_011324934.1 MGRA|XP_003847967.1 MLAR|XP_007406662.1 MORY|QBZ62073.1 NCRA|XP_956870.3 SSCL|APA12472.1

>Orthogroup2712: ANID|CBF77715.1 BCIN|XP_001557203.2 BGRA|VDB93146.1 CFRU|XP_031885387.1 CGLO|KAF3799441.1 CHIG|XP_018152319.1 CVIN|KAF4931809.1 CVYL|A13303 FGRM|XP_011321214.1 MGRA|XP_003848055.1 MLAR|XP_007418039.1 MORY|QBZ57291.1 NCRA|XP_963168.1 SSCL|APA12496.1

>Orthogroup2713: ANID|CBF77747.1 BCIN|XP_001549144.1 BGRA|VCU38753.1 CFRU|XP_031877775.1 CGLO|KAF3810676.1 CHIG|XP_018162433.1 CVIN|KAF4916941.1 CVYL|A07283 FGRM|XP_011318444.1 MGRA|XP_003856481.1 MLAR|XP_007417512.1 MORY|QBZ55712.1 NCRA|XP_962933.1 SSCL|APA15378.1

>Orthogroup2714: ANID|CBF77749.1 BCIN|XP_024550787.1 BGRA|VCU39974.1 CFRU|XP_031887423.1 CGLO|KAF3809174.1 CHIG|XP_018155997.1 CVIN|KAF4919835.1 CVYL|A01362 FGRM|XP_011319100.1 MGRA|XP_003857185.1 MLAR|XP_007415771.1 MORY|QBZ57440.1 NCRA|XP_961173.1 SSCL|APA11869.1

>Orthogroup2716: ANID|CBF77796.1 BCIN|XP_024552892.1 BGRA|VCU39641.1 CFRU|XP_031885405.1 CGLO|KAF3799411.1 CHIG|XP_018152165.1 CVIN|KAF4931815.1 CVYL|A13275 FGRM|XP_011317351.1 MGRA|XP_003847972.1 MLAR|XP_007416407.1 MORY|QBZ53915.1 NCRA|XP_963521.1 SSCL|APA16078.1

>Orthogroup2717: ANID|CBF77798.1 BCIN|XP_024552891.1 BGRA|VCU39640.1 CFRU|XP_031885402.1 CGLO|KAF3799412.1 CHIG|XP_018152166.1 CVIN|KAF4931742.1 CVYL|A13276 FGRM|XP_011317352.1 MGRA|XP_003848138.1 MLAR|XP_007416869.1 MORY|QBZ53916.1 NCRA|XP_963520.1 SSCL|APA16079.1

>Orthogroup2719: ANID|CBF77803.1 BCIN|XP_024548164.1 BGRA|VCU40979.1 CFRU|XP_031880196.1 CGLO|KAF3809028.1 CHIG|XP_018155867.1 CVIN|KAF4923771.1 CVYL|A01611 FGRM|XP_011321018.1 MGRA|XP_003848041.1 MLAR|XP_007408411.1 MORY|QBZ59030.1 NCRA|XP_960860.1 SSCL|APA12413.1

>Orthogroup2720: ANID|CBF77807.1 BCIN|XP_001550883.2 BGRA|VDB94003.1 CFRU|XP_031889370.1 CGLO|KAF3803155.1 CHIG|XP_018155517.1 CVIN|KAF4928288.1 CVYL|A01041 FGRM|XP_011321007.1 MGRA|XP_003848070.1 MLAR|XP_007414320.1 MORY|QBZ62247.1 NCRA|XP_011394653.1 SSCL|APA12408.1

>Orthogroup2721: ANID|CBF77815.1 BCIN|XP_024548468.1 BGRA|VDB93144.1 CFRU|XP_031892381.1 CGLO|KAF3809137.1 CHIG|XP_018155958.1 CVIN|KAF4922113.1 CVYL|A01321 FGRM|XP_011319185.1 MGRA|XP_003848135.1 MLAR|XP_007408774.1 MORY|QBZ56124.1 NCRA|XP_011394661.1 SSCL|APA12193.1

>Orthogroup2722: ANID|CBF77821.1 BCIN|XP_001554449.1 BGRA|VDB93137.1 CFRU|XP_031887358.1 CGLO|KAF3809308.1 CHIG|XP_018156144.1 CVIN|KAF4911362.1 CVYL|A01506 FGRM|XP_011319279.1 MGRA|XP_003848218.1 MLAR|XP_007415401.1 MORY|QBZ62223.1 NCRA|XP_960953.1 SSCL|APA12635.1

>Orthogroup2723: ANID|CBF77829.1 BCIN|XP_001560925.1 BGRA|VDB91100.1 CFRU|XP_031879967.1 CGLO|KAF3798965.1 CHIG|XP_018152848.1 CVIN|KAF4923464.1 CVYL|A07882 FGRM|XP_011323537.1 MGRA|XP_003850691.1 MLAR|XP_007413275.1 MORY|QBZ53346.1 NCRA|XP_959639.1 SSCL|APA06794.1

>Orthogroup2724: ANID|CBF77836.1 BCIN|XP_024547828.1 BGRA|VCU38821.1 CFRU|XP_031886906.1 CGLO|KAF3811464.1 CHIG|XP_018160710.1 CVIN|KAF4917885.1 CVYL|A04668 FGRM|XP_011327248.1 MGRA|XP_003850174.1 MLAR|XP_007412688.1 MORY|QBZ54248.1 NCRA|XP_011394533.1 SSCL|APA08138.1

>Orthogroup2725: ANID|CBF77844.1 BCIN|XP_001555390.1 BGRA|VDB85998.1 CFRU|XP_031886853.1 CGLO|KAF3811459.1 CHIG|XP_018160703.1 CVIN|KAF4917869.1 CVYL|A04661 FGRM|XP_011327241.1 MGRA|XP_003850220.1 MLAR|XP_007405848.1 MORY|QBZ54259.1 NCRA|XP_955999.1 SSCL|APA08134.1

>Orthogroup2726: ANID|CBF77846.1 BCIN|XP_001555385.1 BGRA|VDB86054.1 CFRU|XP_031887000.1 CGLO|KAF3811413.1 CHIG|XP_018160652.1 CVIN|KAF4919714.1 CVYL|A04610 FGRM|XP_011327178.1 MGRA|XP_003849930.1 MLAR|XP_007417480.1 MORY|QBZ54265.1 NCRA|XP_955995.3 SSCL|APA08129.1

>Orthogroup2727: ANID|CBF77869.1 BCIN|XP_001549849.1 BGRA|VDB86175.1 CFRU|XP_031877771.1 CGLO|KAF3810660.1 CHIG|XP_018162415.1 CVIN|KAF4916934.1 CVYL|A07301 FGRM|XP_011318424.1 MGRA|XP_003850458.1 MLAR|XP_007415385.1 MORY|QBZ55663.1 NCRA|XP_963077.1 SSCL|APA08257.1

>Orthogroup2728: ANID|CBF77872.1 BCIN|XP_001549852.1 BGRA|VDB86173.1 CFRU|XP_031877773.1 CGLO|KAF3810664.1 CHIG|XP_018162420.1 CVIN|KAF4916955.1 CVYL|A07296 FGRM|XP_011318430.1 MGRA|XP_003850054.1 MLAR|XP_007406659.1 MORY|QBZ55697.1 NCRA|XP_963082.1 SSCL|APA08259.1

>Orthogroup2729: ANID|CBF77874.1 BCIN|XP_024547609.1 BGRA|VDB86161.1 CFRU|XP_031886460.1 CGLO|KAF3806317.1 CHIG|XP_018162151.1 CVIN|KAF4923974.1 CVYL|A11909 FGRM|XP_011326595.1 MGRA|XP_003850359.1 MLAR|XP_007416309.1 MORY|QBZ61578.1 NCRA|XP_957296.1 SSCL|APA07796.1

>Orthogroup2730: ANID|CBF77885.1 BCIN|XP_024551194.1 BGRA|VCU39443.1 CFRU|XP_031886641.1 CGLO|KAF3801213.1 CHIG|XP_018162596.1 CVIN|KAF4932113.1 CVYL|A07119 FGRM|XP_011324117.1 MGRA|XP_003856464.1 MLAR|XP_007416893.1 MORY|QBZ56495.1 NCRA|XP_959028.1 SSCL|APA10147.1

>Orthogroup2731: ANID|CBF77886.1 BCIN|XP_001555776.1 BGRA|VCU39449.1 CFRU|XP_031886812.1 CGLO|KAF3801202.1 CHIG|XP_018162495.1 CVIN|KAF4932043.1 CVYL|A07132 FGRM|XP_011324109.1 MGRA|XP_003847938.1 MLAR|XP_007409371.1 MORY|QBZ56503.1 NCRA|XP_958006.1 SSCL|APA10133.1

>Orthogroup2732: ANID|CBF77898.1 BCIN|XP_001549846.1 BGRA|VDB86197.1 CFRU|XP_031877768.1 CGLO|KAF3810658.1 CHIG|XP_018162412.1 CVIN|KAF4916937.1 CVYL|A07304 FGRM|XP_011318427.1 MGRA|XP_003850055.1 MLAR|XP_007412648.1 MORY|QBZ55715.1 NCRA|XP_001728214.1 SSCL|APA08254.1

>Orthogroup2733: ANID|CBF77904.1 BCIN|XP_001555069.1 BGRA|VDB85911.1 CFRU|XP_031887075.1 CGLO|KAF3811395.1 CHIG|XP_018160631.1 CVIN|KAF4919704.1 CVYL|A04588 FGRM|XP_011327188.1 MGRA|XP_003855079.1 MLAR|XP_007413652.1 MORY|QBZ64736.1 NCRA|XP_961018.3 SSCL|APA07823.1

>Orthogroup2734: ANID|CBF77911.1 BCIN|XP_024551360.1 BGRA|VCU39947.1 CFRU|XP_031886426.1 CGLO|KAF3806215.1 CHIG|XP_018162051.1 CVIN|KAF4931464.1 CVYL|A11804 FGRM|XP_011326621.1 MGRA|XP_003849953.1 MLAR|XP_007410261.1 MORY|QBZ61347.1 NCRA|XP_957219.1 SSCL|APA10474.1

>Orthogroup2735: ANID|CBF77913.1 BCIN|XP_001556947.1 BGRA|VDB91337.1 CFRU|XP_031893436.1 CGLO|KAF3809566.1 CHIG|XP_018152611.1 CVIN|KAF4912197.1 CVYL|A08460 FGRM|XP_011317946.1 MGRA|XP_003850490.1 MLAR|XP_007407920.1 MORY|QBZ54988.1 NCRA|XP_958192.1 SSCL|APA10555.1

>Orthogroup2736: ANID|CBF77915.1 BCIN|XP_024551319.1 BGRA|VDB91335.1 CFRU|XP_031893563.1 CGLO|KAF3809529.1 CHIG|XP_018152641.1 CVIN|KAF4918551.1 CVYL|A08492 FGRM|XP_011317947.1 MGRA|XP_003849922.1 MLAR|XP_007415677.1 MORY|QBZ54987.1 NCRA|XP_958193.1 SSCL|APA10554.1

>Orthogroup2737: ANID|CBF77917.1 BCIN|XP_024547628.1 BGRA|VCU38832.1 CFRU|XP_031886356.1 CGLO|KAF3806254.1 CHIG|XP_018162086.1 CVIN|KAF4931454.1 CVYL|A11843 FGRM|XP_011326634.1 MGRA|XP_003850119.1 MLAR|XP_007415032.1 MORY|QBZ61515.1 NCRA|XP_957292.1 SSCL|APA07775.1

>Orthogroup2738: ANID|CBF77945.1 BCIN|XP_001555826.2 BGRA|VCU40661.1 CFRU|XP_031891460.1 CGLO|KAF3804514.1 CHIG|XP_018159474.1 CVIN|KAF4924515.1 CVYL|A04229 FGRM|XP_011327135.1 MGRA|XP_003850044.1 MLAR|XP_007405416.1 MORY|QBZ64740.1 NCRA|XP_959198.1 SSCL|APA06099.1

>Orthogroup2739: ANID|CBF77949.1 BCIN|XP_024546908.1 BGRA|VCU40665.1 CFRU|XP_031880723.1 CGLO|KAF3811159.1 CHIG|XP_018160348.1 CVIN|KAF4931270.1 CVYL|A04318 FGRM|XP_011324727.1 MGRA|XP_003850393.1 MLAR|XP_007405845.1 MORY|QBZ58017.1 NCRA|XP_011394597.1 SSCL|APA05833.1

>Orthogroup2740: ANID|CBF77953.1 BCIN|XP_001558968.1 BGRA|VCU40668.1 CFRU|XP_031889062.1 CGLO|KAF3804234.1 CHIG|XP_018160066.1 CVIN|KAF4930128.1 CVYL|A03910 FGRM|XP_011324596.1 MGRA|XP_003849977.1 MLAR|XP_007415579.1 MORY|QBZ57465.1 NCRA|XP_960632.1 SSCL|APA05831.1

>Orthogroup2741: ANID|CBF77960.1 BCIN|XP_024548331.1 BGRA|VCU40538.1 CFRU|XP_031888761.1 CGLO|KAF3799235.1 CHIG|XP_018159840.1 CVIN|KAF4893592.1 CVYL|A10127 FGRM|XP_011328829.1 MGRA|XP_003850307.1 MLAR|XP_007417938.1 MORY|QBZ63740.1 NCRA|XP_960618.2 SSCL|APA12579.1

>Orthogroup2742: ANID|CBF77971.1 BCIN|XP_024553331.1 BGRA|VCU40501.1 CFRU|XP_031886532.1 CGLO|KAF3800266.1 CHIG|XP_018162752.1 CVIN|KAF4927561.1 CVYL|A06971 FGRM|XP_011327027.1 MGRA|XP_003850420.1 MLAR|XP_007412545.1 MORY|QBZ61339.1 NCRA|XP_957341.1 SSCL|APA13646.1

>Orthogroup2744: ANID|CBF77985.1 BCIN|XP_001555894.1 BGRA|VCU40590.1 CFRU|XP_031882257.1 CGLO|KAF3804313.1 CHIG|XP_018160106.1 CVIN|KAF4920099.1 CVYL|A04000 FGRM|XP_011324566.1 MGRA|XP_003850126.1 MLAR|XP_007407025.1 MORY|QBZ54772.1 NCRA|XP_961023.1 SSCL|APA06049.1

>Orthogroup2746: ANID|CBF77996.1 BCIN|XP_001555821.2 BGRA|VCU40645.1 CFRU|XP_031882133.1 CGLO|KAF3804341.1 CHIG|XP_018160130.1 CVIN|KAF4918337.1 CVYL|A04029 FGRM|XP_011324575.1 MGRA|XP_003850494.1 MLAR|XP_007418212.1 MORY|QBZ59168.1 NCRA|XP_960203.3 SSCL|APA05920.1

>Orthogroup2747: ANID|CBF77999.1 BCIN|XP_001555946.1 BGRA|VCU40646.1 CFRU|XP_031889057.1 CGLO|KAF3804230.1 CHIG|XP_018160070.1 CVIN|KAF4930138.1 CVYL|A03914 FGRM|XP_011324593.1 MGRA|XP_003849867.1 MLAR|XP_007410380.1 MORY|QBZ64649.1 NCRA|XP_961349.1 SSCL|APA14518.1

>Orthogroup2748: ANID|CBF78005.1 BCIN|XP_024548329.1 BGRA|VCU40537.1 CFRU|XP_031888825.1 CGLO|KAF3799234.1 CHIG|XP_018159841.1 CVIN|KAF4893587.1 CVYL|A10126 FGRM|XP_011328827.1 MGRA|XP_003850253.1 MLAR|XP_007410099.1 MORY|QBZ63739.1 NCRA|XP_960620.2 SSCL|APA12580.1

>Orthogroup2749: ANID|CBF78014.1 BCIN|XP_001557004.1 BGRA|VCU41100.1 CFRU|XP_031886358.1 CGLO|KAF3806351.1 CHIG|XP_018162243.1 CVIN|KAF4899311.1 CVYL|A11938 FGRM|XP_011324054.1 MGRA|XP_003856292.1 MLAR|XP_007408223.1 MORY|QBZ61533.1 NCRA|XP_001728137.2 SSCL|APA10226.1

>Orthogroup2750: ANID|CBF78020.1 BCIN|XP_024551298.1 BGRA|VCU41065.1 CFRU|XP_031885158.1 CGLO|KAF3807679.1 CHIG|XP_018162901.1 CVIN|KAF4927775.1 CVYL|A06872 FGRM|XP_011326287.1 MGRA|XP_003857293.1 MLAR|XP_007416276.1 MORY|QBZ55624.1 NCRA|XP_011393760.1 SSCL|APA10228.1

>Orthogroup2751: ANID|CBF78029.1 BCIN|XP_024551292.1 BGRA|VCU39509.1 CFRU|XP_031878417.1 CGLO|KAF3810762.1 CHIG|XP_018162559.1 CVIN|KAF4932082.1 CVYL|A07192 FGRM|XP_011326313.1 MGRA|XP_003857382.1 MLAR|XP_007410608.1 MORY|QBZ59703.1 NCRA|XP_956396.2 SSCL|APA10251.1

>Orthogroup2752: ANID|CBF78033.1 BCIN|XP_024548010.1 BGRA|VDB90912.1 CFRU|XP_031888356.1 CGLO|KAF3803620.1 CHIG|XP_018163847.1 CVIN|KAF4920360.1 CVYL|A14552 FGRM|XP_011326549.1 MGRA|XP_003856381.1 MLAR|XP_007417610.1 MORY|QBZ63195.1 NCRA|XP_963950.2 SSCL|APA08321.1

>Orthogroup2754: ANID|CBF78100.1 BCIN|XP_001550468.1 BGRA|VDB88151.1 CFRU|XP_031892539.1 CGLO|KAF3797227.1 CHIG|XP_018157267.1 CVIN|KAF4917702.1 CVYL|A10470 FGRM|XP_011320108.1 MGRA|XP_003852721.1 MLAR|XP_007415307.1 MORY|QBZ66000.1 NCRA|XP_964888.1 SSCL|APA11658.1

>Orthogroup2756: ANID|CBF78163.1 BCIN|XP_024550849.1 BGRA|VDB88143.1 CFRU|XP_031891304.1 CGLO|KAF3797381.1 CHIG|XP_018157129.1 CVIN|KAF4929382.1 CVYL|A10303 FGRM|XP_011320077.1 MGRA|XP_003852069.1 MLAR|XP_007410654.1 MORY|QBZ65939.1 NCRA|XP_964893.2 SSCL|APA11774.1

>Orthogroup2762: ANID|CBF78544.1 BCIN|XP_001551964.1 BGRA|VDB94737.1 CFRU|XP_031891536.1 CGLO|KAF3804705.1 CHIG|XP_018161776.1 CVIN|KAF4923388.1 CVYL|A05418 FGRM|XP_011321514.1 MGRA|XP_003854886.1 MLAR|XP_007407791.1 MORY|QBZ59656.1 NCRA|XP_956785.3 SSCL|APA10956.1

>Orthogroup2763: ANID|CBF78558.1 BCIN|XP_001551718.2 BGRA|VDB86335.1 CFRU|XP_031882439.1 CGLO|KAF3807539.1 CHIG|XP_018156397.1 CVIN|KAF4928923.1 CVYL|A02944 FGRM|XP_011318576.1 MGRA|XP_003854877.1 MLAR|XP_007410000.1 MORY|QBZ56946.1 NCRA|XP_011394865.1 SSCL|APA11633.1

>Orthogroup2765: ANID|CBF78570.1 BCIN|XP_024550260.1 BGRA|VDB94542.1 CFRU|XP_031878195.1 CGLO|KAF3808539.1 CHIG|XP_018161935.1 CVIN|KAF4917622.1 CVYL|A11605 FGRM|XP_011318515.1 MGRA|XP_003854885.1 MLAR|XP_007412145.1 MORY|QBZ59645.1 NCRA|XP_011393851.1 SSCL|APA10935.1

>Orthogroup2767: ANID|CBF78591.1 BCIN|XP_001553034.2 BGRA|VDB94810.1 CFRU|XP_031887024.1 CGLO|KAF3811417.1 CHIG|XP_018160656.1 CVIN|KAF4919691.1 CVYL|A04614 FGRM|XP_011326897.1 MGRA|XP_003855637.1 MLAR|XP_007410169.1 MORY|QBZ58110.1 NCRA|XP_961391.2 SSCL|APA11272.1

>Orthogroup2769: ANID|CBF78614.1 BCIN|XP_024550510.1 BGRA|VDB94855.1 CFRU|XP_031887119.1 CGLO|KAF3811418.1 CHIG|XP_018160657.1 CVIN|KAF4919690.1 CVYL|A04615 FGRM|XP_011326895.1 MGRA|XP_003855627.1 MLAR|XP_007412471.1 MORY|QBZ56894.1 NCRA|XP_961558.1 SSCL|APA11273.1

>Orthogroup2770: ANID|CBF78644.1 BCIN|XP_001550290.1 BGRA|VCU39187.1 CFRU|XP_031887469.1 CGLO|KAF3803318.1 CHIG|XP_018161170.1 CVIN|KAF4898744.1 CVYL|A03755 FGRM|XP_011321361.1 MGRA|XP_003849674.1 MLAR|XP_007414016.1 MORY|QBZ64535.1 NCRA|XP_011394133.1 SSCL|APA12843.1

>Orthogroup2771: ANID|CBF78675.1 BCIN|XP_024552428.1 BGRA|VDB93550.1 CFRU|XP_031886435.1 CGLO|KAF3806349.1 CHIG|XP_018162245.1 CVIN|KAF4899319.1 CVYL|A11936 FGRM|XP_011326627.1 MGRA|XP_003847258.1 MLAR|XP_007418278.1 MORY|QBZ61342.1 NCRA|XP_956688.3 SSCL|APA14981.1

>Orthogroup2772: ANID|CBF78686.1 BCIN|XP_024552442.1 BGRA|VDB93478.1 CFRU|XP_031885033.1 CGLO|KAF3800356.1 CHIG|XP_018162845.1 CVIN|KAF4914542.1 CVYL|A07586 FGRM|XP_011326391.1 MGRA|XP_003854561.1 MLAR|XP_007408342.1 MORY|QBZ59531.1 NCRA|XP_957819.2 SSCL|APA15007.1

>Orthogroup2773: ANID|CBF78691.1 BCIN|XP_024553907.1 BGRA|VDB96362.1 CFRU|XP_031883010.1 CGLO|KAF3800745.1 CHIG|XP_018163294.1 CVIN|KAF4919650.1 CVYL|A00459 FGRM|XP_011326178.1 MGRA|XP_003853805.1 MLAR|XP_007413788.1 MORY|QBZ58245.1 NCRA|XP_962756.2 SSCL|APA15186.1

>Orthogroup2776: ANID|CBF78749.1 BCIN|XP_001552103.1 BGRA|VDB96312.1 CFRU|XP_031886618.1 CGLO|KAF3801228.1 CHIG|XP_018162610.1 CVIN|KAF4932046.1 CVYL|A07102 FGRM|XP_011324004.1 MGRA|XP_003853877.1 MLAR|XP_007408472.1 MORY|QBZ59313.1 NCRA|XP_957808.1 SSCL|APA05287.1

>Orthogroup2777: ANID|CBF78750.1 BCIN|XP_001552544.1 BGRA|VDB94941.1 CFRU|XP_031891443.1 CGLO|KAF3804576.1 CHIG|XP_018161657.1 CVIN|KAF4923070.1 CVYL|A05301 FGRM|XP_011318685.1 MGRA|XP_003855044.1 MLAR|XP_007413608.1 MORY|QBZ61141.1 NCRA|XP_962902.3 SSCL|APA10804.1

>Orthogroup2778: ANID|CBF78760.1 BCIN|XP_001553950.1 BGRA|VDB94852.1 CFRU|XP_031886468.1 CGLO|KAF3806189.1 CHIG|XP_018162174.1 CVIN|KAF4931488.1 CVYL|A11776 FGRM|XP_011324078.1 MGRA|XP_003854896.1 MLAR|XP_007404109.1 MORY|QBZ59558.1 NCRA|XP_011393721.1 SSCL|APA11079.1

>Orthogroup2779: ANID|CBF78768.1 BCIN|XP_024550125.1 BGRA|VDB94909.1 CFRU|XP_031891481.1 CGLO|KAF3797891.1 CHIG|XP_018161631.1 CVIN|KAF4923091.1 CVYL|A05328 FGRM|XP_011318672.1 MGRA|XP_003855043.1 MLAR|XP_007409964.1 MORY|QBZ61140.1 NCRA|XP_011393870.1 SSCL|APA10805.1

>Orthogroup2780: ANID|CBF78870.1 BCIN|XP_001552542.2 BGRA|VDB94945.1 CFRU|XP_031891638.1 CGLO|KAF3797911.1 CHIG|XP_018161609.1 CVIN|KAF4905692.1 CVYL|A05347 FGRM|XP_011318653.1 MGRA|XP_003855120.1 MLAR|XP_007410106.1 MORY|QBZ61996.1 NCRA|XP_958516.1 SSCL|APA10802.1

>Orthogroup2782: ANID|CBF78985.1 BCIN|XP_001553497.1 BGRA|VDB84255.1 CFRU|XP_031891579.1 CGLO|KAF3797635.1 CHIG|XP_018157427.1 CVIN|KAF4890325.1 CVYL|A11062 FGRM|XP_011323030.1 MGRA|XP_003854328.1 MLAR|XP_007407036.1 MORY|QBZ66428.1 NCRA|XP_965393.2 SSCL|APA10869.1

>Orthogroup2783: ANID|CBF78986.1 BCIN|XP_024550188.1 BGRA|VDB84253.1 CFRU|XP_031891578.1 CGLO|KAF3797634.1 CHIG|XP_018157426.1 CVIN|KAF4890322.1 CVYL|A11066 FGRM|XP_011323029.1 MGRA|XP_003854327.1 MLAR|XP_007412444.1 MORY|QBZ66429.1 NCRA|XP_965394.1 SSCL|APA10868.1

>Orthogroup2784: ANID|CBF79000.1 BCIN|XP_001553507.2 BGRA|VDB84239.1 CFRU|XP_031881702.1 CGLO|KAF3807407.1 CHIG|XP_018156819.1 CVIN|KAF4929597.1 CVYL|A10904 FGRM|XP_011320431.1 MGRA|XP_003854602.1 MLAR|XP_007415996.1 MORY|QBZ65578.1 NCRA|XP_965069.3 SSCL|APA10861.1

>Orthogroup2787: ANID|CBF79070.1 BCIN|XP_024547889.1 BGRA|VDB84455.1 CFRU|XP_031891385.1 CGLO|KAF3804715.1 CHIG|XP_018161800.1 CVIN|KAF4923378.1 CVYL|A05407 FGRM|XP_011321516.1 MGRA|XP_003853956.1 MLAR|XP_007407846.1 MORY|QBZ59674.1 NCRA|XP_963104.1 SSCL|APA08211.1

>Orthogroup2788: ANID|CBF79080.1 BCIN|XP_001555085.1 BGRA|VDB86151.1 CFRU|XP_031877223.1 CGLO|KAF3806455.1 CHIG|XP_018162343.1 CVIN|KAF4897894.1 CVYL|A12053 FGRM|XP_011323938.1 MGRA|XP_003855793.1 MLAR|XP_007405703.1 MORY|QBZ61400.1 NCRA|XP_962814.1 SSCL|APA07837.1

>Orthogroup2790: ANID|CBF79184.1 BCIN|XP_001558791.1 BGRA|VDB88254.1 CFRU|XP_031876559.1 CGLO|KAF3811488.1 CHIG|XP_018160741.1 CVIN|KAF4926792.1 CVYL|A04693 FGRM|XP_011317784.1 MGRA|XP_003852314.1 MLAR|XP_007412437.1 MORY|QBZ57152.1 NCRA|XP_962657.1 SSCL|APA05493.1

>Orthogroup2791: ANID|CBF79187.1 BCIN|XP_001558350.2 BGRA|VCU40151.1 CFRU|XP_031876536.1 CGLO|KAF3811491.1 CHIG|XP_018160744.1 CVIN|KAF4926757.1 CVYL|A04695 FGRM|XP_011317787.1 MGRA|XP_003852356.1 MLAR|XP_007405361.1 MORY|QBZ56695.1 NCRA|XP_962669.1 SSCL|APA13727.1

>Orthogroup2792: ANID|CBF79189.1 BCIN|XP_001558793.2 BGRA|VCU40152.1 CFRU|XP_031876537.1 CGLO|KAF3811490.1 CHIG|XP_018160743.1 CVIN|KAF4926755.1 CVYL|A04694 FGRM|XP_011317786.1 MGRA|XP_003852259.1 MLAR|XP_007416033.1 MORY|QBZ56696.1 NCRA|XP_962654.2 SSCL|APA05491.1

>Orthogroup2793: ANID|CBF79193.1 BCIN|XP_001558352.1 BGRA|VCU40147.1 CFRU|XP_031876551.1 CGLO|KAF3811493.1 CHIG|XP_018160746.1 CVIN|KAF4926751.1 CVYL|A04698 FGRM|XP_011317789.1 MGRA|XP_003853275.1 MLAR|XP_007409207.1 MORY|QBZ56316.1 NCRA|XP_011394389.1 SSCL|APA13729.1

>Orthogroup2794: ANID|CBF79199.1 BCIN|XP_001558120.1 BGRA|VDB92700.1 CFRU|XP_031893585.1 CGLO|KAF3800798.1 CHIG|XP_018150904.1 CVIN|KAF4922480.1 CVYL|A08753 FGRM|XP_011323777.1 MGRA|XP_003852482.1 MLAR|XP_007414190.1 MORY|QBZ56545.1 NCRA|XP_961948.2 SSCL|APA12968.1

>Orthogroup2795: ANID|CBF79209.1 BCIN|XP_001558109.1 BGRA|VDB92704.1 CFRU|XP_031893305.1 CGLO|KAF3800790.1 CHIG|XP_018150896.1 CVIN|KAF4922511.1 CVYL|A08761 FGRM|XP_011323774.1 MGRA|XP_003853286.1 MLAR|XP_007417543.1 MORY|QBZ56543.1 NCRA|XP_962337.3 SSCL|APA12960.1

>Orthogroup2796: ANID|CBF79213.1 BCIN|XP_024552189.1 BGRA|VDB92707.1 CFRU|XP_031893587.1 CGLO|KAF3800800.1 CHIG|XP_018150906.1 CVIN|KAF4922506.1 CVYL|A08751 FGRM|XP_011323779.1 MGRA|XP_003852334.1 MLAR|XP_007411098.1 MORY|QBZ56547.1 NCRA|XP_961946.2 SSCL|APA13280.1

>Orthogroup2797: ANID|CBF79220.1 BCIN|XP_024552049.1 BGRA|VDB92809.1 CFRU|XP_031887748.1 CGLO|KAF3803296.1 CHIG|XP_018161146.1 CVIN|KAF4928632.1 CVYL|A03732 FGRM|XP_011318411.1 MGRA|XP_003852547.1 MLAR|XP_007406692.1 MORY|QBZ64571.1 NCRA|XP_962003.3 SSCL|APA12817.1

>Orthogroup2799: ANID|CBF79227.1 BCIN|XP_001558115.1 BGRA|VDB86326.1 CFRU|XP_031893309.1 CGLO|KAF3800794.1 CHIG|XP_018150901.1 CVIN|KAF4922451.1 CVYL|A08757 FGRM|XP_011316820.1 MGRA|XP_003852420.1 MLAR|XP_007405338.1 MORY|QBZ55388.1 NCRA|XP_957941.1 SSCL|APA12964.1

>Orthogroup2801: ANID|CBF79250.1 BCIN|XP_001557854.1 BGRA|VDB92714.1 CFRU|XP_031887524.1 CGLO|KAF3803211.1 CHIG|XP_018161058.1 CVIN|KAF4928657.1 CVYL|A03639 FGRM|XP_011324200.1 MGRA|XP_003852175.1 MLAR|XP_007417966.1 MORY|QBZ58758.1 NCRA|XP_961839.3 SSCL|APA12826.1

>Orthogroup2802: ANID|CBF79253.1 BCIN|XP_001560727.1 BGRA|VCU39383.1 CFRU|XP_031881292.1 CGLO|KAF3802473.1 CHIG|XP_018154763.1 CVIN|KAF4916888.1 CVYL|A02054 FGRM|XP_011316540.1 MGRA|XP_003853672.1 MLAR|XP_007408957.1 MORY|QBZ59087.1 NCRA|XP_956018.1 SSCL|APA07993.1

>Orthogroup2803: ANID|CBF79255.1 BCIN|XP_024548754.1 BGRA|VDB89383.1 CFRU|XP_031881315.1 CGLO|KAF3802457.1 CHIG|XP_018154757.1 CVIN|KAF4916883.1 CVYL|A02048 FGRM|XP_011316539.1 MGRA|XP_003853633.1 MLAR|XP_007408827.1 MORY|QBZ59086.1 NCRA|XP_956726.1 SSCL|APA09737.1

>Orthogroup2804: ANID|CBF79257.1 BCIN|XP_024546564.1 BGRA|VDB95140.1 CFRU|XP_031877249.1 CGLO|KAF3806830.1 CHIG|XP_018159525.1 CVIN|KAF4909282.1 CVYL|A02477 FGRM|XP_011320494.1 MGRA|XP_003854611.1 MLAR|XP_007403887.1 MORY|QBZ62924.1 NCRA|XP_960485.3 SSCL|APA06786.1

>Orthogroup2805: ANID|CBF79260.1 BCIN|XP_001554960.1 BGRA|VDB86230.1 CFRU|XP_031877177.1 CGLO|KAF3806442.1 CHIG|XP_018162330.1 CVIN|KAF4918061.1 CVYL|A12039 FGRM|XP_011323960.1 MGRA|XP_003854347.1 MLAR|XP_007416605.1 MORY|QBZ61419.1 NCRA|XP_963126.1 SSCL|APA07753.1

>Orthogroup2807: ANID|CBF79269.1 BCIN|XP_024547645.1 BGRA|VDB86224.1 CFRU|XP_031886888.1 CGLO|KAF3811299.1 CHIG|XP_018160522.1 CVIN|KAF4927232.1 CVYL|A04478 FGRM|XP_011326467.1 MGRA|XP_003854696.1 MLAR|XP_007412815.1 MORY|QBZ57986.1 NCRA|XP_958772.1 SSCL|APA07750.1

>Orthogroup2808: ANID|CBF79275.1 BCIN|XP_001561085.1 BGRA|VDB95164.1 CFRU|XP_031880909.1 CGLO|KAF3806969.1 CHIG|XP_018159620.1 CVIN|KAF4918776.1 CVYL|A06261 FGRM|XP_011320509.1 MGRA|XP_003854959.1 MLAR|XP_007419529.1 MORY|QBZ62927.1 NCRA|XP_960507.2 SSCL|APA06671.1

>Orthogroup2809: ANID|CBF79279.1 BCIN|XP_001556190.1 BGRA|VDB95152.1 CFRU|XP_031880843.1 CGLO|KAF3806888.1 CHIG|XP_018159570.1 CVIN|KAF4894666.1 CVYL|A02531 FGRM|XP_011320454.1 MGRA|XP_003853939.1 MLAR|XP_007417220.1 MORY|QBZ61865.1 NCRA|XP_960516.1 SSCL|APA09024.1

>Orthogroup2811: ANID|CBF79288.1 BCIN|XP_024546985.1 BGRA|VDB93884.1 CFRU|XP_031885346.1 CGLO|KAF3799456.1 CHIG|XP_018154444.1 CVIN|KAF4931798.1 CVYL|A13317 FGRM|XP_011325521.1 MGRA|XP_003853903.1 MLAR|XP_007415397.1 MORY|QBZ54373.1 NCRA|XP_963149.3 SSCL|APA05949.1

>Orthogroup2812: ANID|CBF79292.1 BCIN|XP_024546124.1 BGRA|VDB89451.1 CFRU|XP_031876411.1 CGLO|KAF3801969.1 CHIG|XP_018159414.1 CVIN|KAF4919799.1 CVYL|A02404 FGRM|XP_011320392.1 MGRA|XP_003854972.1 MLAR|XP_007413232.1 MORY|QBZ63980.1 NCRA|XP_960562.3 SSCL|APA06649.1

>Orthogroup2813: ANID|CBF79306.1 BCIN|XP_001548376.1 BGRA|VDB95217.1 CFRU|XP_031879164.1 CGLO|KAF3807043.1 CHIG|XP_018159704.1 CVIN|KAF4920258.1 CVYL|A06336 FGRM|XP_011320477.1 MGRA|XP_003854980.1 MLAR|XP_007417673.1 MORY|QBZ61820.1 NCRA|XP_960554.3 SSCL|APA06960.1

>Orthogroup2814: ANID|CBF79308.1 BCIN|XP_024546263.1 BGRA|VDB95219.1 CFRU|XP_031879151.1 CGLO|KAF3807044.1 CHIG|XP_018159703.1 CVIN|KAF4920259.1 CVYL|A06338 FGRM|XP_011320478.1 MGRA|XP_003854710.1 MLAR|XP_007415908.1 MORY|QBZ61819.1 NCRA|XP_011394974.1 SSCL|APA06959.1

>Orthogroup2815: ANID|CBF79310.1 BCIN|XP_001561074.1 BGRA|VDB95174.1 CFRU|XP_031878141.1 CGLO|KAF3801827.1 CHIG|XP_018159321.1 CVIN|KAF4895039.1 CVYL|A12880 FGRM|XP_011320638.1 MGRA|XP_003854759.1 MLAR|XP_007406534.1 MORY|QBZ62915.1 NCRA|XP_960476.1 SSCL|APA06766.1

>Orthogroup2816: ANID|CBF79312.1 BCIN|XP_001547053.1 BGRA|VDB93803.1 CFRU|XP_031885976.1 CGLO|KAF3811970.1 CHIG|XP_018155263.1 CVIN|KAF4930328.1 CVYL|A05532 FGRM|XP_011317428.1 MGRA|XP_003853920.1 MLAR|XP_007411602.1 MORY|QBZ56165.1 NCRA|XP_963354.1 SSCL|APA05952.1

>Orthogroup2817: ANID|CBF79317.1 BCIN|XP_024546982.1 BGRA|VDB93809.1 CFRU|XP_031885975.1 CGLO|KAF3811971.1 CHIG|XP_018155262.1 CVIN|KAF4930327.1 CVYL|A05531 FGRM|XP_011317429.1 MGRA|XP_003854352.1 MLAR|XP_007408914.1 MORY|QBZ56166.1 NCRA|XP_963351.1 SSCL|APA05951.1

>Orthogroup2818: ANID|CBF79330.1 BCIN|XP_001558795.2 BGRA|VDB93838.1 CFRU|XP_031883336.1 CGLO|KAF3800573.1 CHIG|XP_018152107.1 CVIN|KAF4927066.1 CVYL|A13142 FGRM|XP_011317233.1 MGRA|XP_003853961.1 MLAR|XP_007410892.1 MORY|QBZ60337.1 NCRA|XP_956908.1 SSCL|APA05955.1

>Orthogroup2820: ANID|CBF79349.1 BCIN|XP_024548174.1 BGRA|VDB89597.1 CFRU|XP_031876394.1 CGLO|KAF3797791.1 CHIG|XP_018156470.1 CVIN|KAF4923153.1 CVYL|A03016 FGRM|XP_011318590.1 MGRA|XP_003851377.1 MLAR|XP_007411936.1 MORY|QBZ57790.1 NCRA|XP_960037.1 SSCL|APA12425.1

>Orthogroup2821: ANID|CBF79356.1 BCIN|XP_001550927.1 BGRA|VDB93324.1 CFRU|XP_031876386.1 CGLO|KAF3797775.1 CHIG|XP_018156444.1 CVIN|KAF4918831.1 CVYL|A02994 FGRM|XP_011318771.1 MGRA|XP_003851636.1 MLAR|XP_007405516.1 MORY|QBZ63530.1 NCRA|XP_956580.1 SSCL|APA12382.1

>Orthogroup2822: ANID|CBF79362.1 BCIN|XP_024550968.1 BGRA|VDB89171.1 CFRU|XP_031887176.1 CGLO|KAF3809396.1 CHIG|XP_018156237.1 CVIN|KAF4916424.1 CVYL|A02974 FGRM|XP_011319173.1 MGRA|XP_003851396.1 MLAR|XP_007416409.1 MORY|QBZ57431.1 NCRA|XP_011394688.1 SSCL|APA11578.1

>Orthogroup2823: ANID|CBF79365.1 BCIN|XP_001550466.1 BGRA|VDB89175.1 CFRU|XP_031887207.1 CGLO|KAF3809344.1 CHIG|XP_018156196.1 CVIN|KAF4924170.1 CVYL|A01549 FGRM|XP_011318896.1 MGRA|XP_003851631.1 MLAR|XP_007406708.1 MORY|QBZ63099.1 NCRA|XP_957381.1 SSCL|APA11660.1

>Orthogroup2824: ANID|CBF79384.1 BCIN|XP_001548600.1 BGRA|VDB93204.1 CFRU|XP_031892560.1 CGLO|KAF3809147.1 CHIG|XP_018155951.1 CVIN|KAF4922135.1 CVYL|A01332 FGRM|XP_011318805.1 MGRA|XP_003851680.1 MLAR|XP_007408722.1 MORY|QBZ57342.1 NCRA|XP_958288.3 SSCL|APA11733.1

>Orthogroup2826: ANID|CBF79397.1 BCIN|XP_024550960.1 BGRA|VDB89183.1 CFRU|XP_031892858.1 CGLO|KAF3809130.1 CHIG|XP_018155964.1 CVIN|KAF4922125.1 CVYL|A01314 FGRM|XP_011319192.1 MGRA|XP_003851965.1 MLAR|XP_007407496.1 MORY|QBZ57327.1 NCRA|XP_011394664.1 SSCL|APA11572.1

>Orthogroup2827: ANID|CBF79408.1 BCIN|XP_024547939.1 BGRA|VDB84444.1 CFRU|XP_031891314.1 CGLO|KAF3804693.1 CHIG|XP_018161764.1 CVIN|KAF4923390.1 CVYL|A05192 FGRM|XP_011321519.1 MGRA|XP_003851511.1 MLAR|XP_007412037.1 MORY|QBZ59630.1 NCRA|XP_956780.2 SSCL|APA08274.1

>Orthogroup2828: ANID|CBF79417.1 BCIN|XP_001554116.1 BGRA|VDB91073.1 CFRU|XP_031877864.1 CGLO|KAF3806625.1 CHIG|XP_018164237.1 CVIN|KAF4918920.1 CVYL|A12337 FGRM|XP_011316215.1 MGRA|XP_003851562.1 MLAR|XP_007406774.1 MORY|QBZ56400.1 NCRA|XP_964127.2 SSCL|APA05538.1

>Orthogroup2829: ANID|CBF79419.1 BCIN|XP_001554114.1 BGRA|VDB91070.1 CFRU|XP_031882730.1 CGLO|KAF3812070.1 CHIG|XP_018164078.1 CVIN|KAF4923707.1 CVYL|A14246 FGRM|XP_011326770.1 MGRA|XP_003851780.1 MLAR|XP_007417829.1 MORY|QBZ56399.1 NCRA|XP_964124.2 SSCL|APA05540.1

>Orthogroup2831: ANID|CBF79440.1 BCIN|XP_001546882.1 BGRA|VDB88135.1 CFRU|XP_031885757.1 CGLO|KAF3810178.1 CHIG|XP_018157797.1 CVIN|KAF4929167.1 CVYL|A02770 FGRM|XP_011320118.1 MGRA|XP_003852818.1 MLAR|XP_007417469.1 MORY|QBZ61284.1 NCRA|XP_957577.2 SSCL|APA08578.1

>Orthogroup2833: ANID|CBF79450.1 BCIN|XP_001546070.1 BGRA|VDB93622.1 CFRU|XP_031886260.1 CGLO|KAF3806276.1 CHIG|XP_018162102.1 CVIN|KAF4923955.1 CVYL|A11867 FGRM|XP_011326632.1 MGRA|XP_003853852.1 MLAR|XP_007407003.1 MORY|QBZ61517.1 NCRA|XP_962915.2 SSCL|APA14994.1

>Orthogroup2835: ANID|CBF79489.1 BCIN|XP_001547702.1 BGRA|VDB89658.1 CFRU|XP_031887721.1 CGLO|KAF3803312.1 CHIG|XP_018161164.1 CVIN|KAF4898736.1 CVYL|A03747 FGRM|XP_011321367.1 MGRA|XP_003849681.1 MLAR|XP_007407647.1 MORY|QBZ64584.1 NCRA|XP_962216.1 SSCL|APA13297.1

>Orthogroup2837: ANID|CBF79503.1 BCIN|XP_001545252.2 BGRA|VDB92837.1 CFRU|XP_031890256.1 CGLO|KAF3798717.1 CHIG|XP_018151273.1 CVIN|KAF4910265.1 CVYL|A09195 FGRM|XP_011327921.1 MGRA|XP_003849809.1 MLAR|XP_007417819.1 MORY|QBZ56565.1 NCRA|XP_961789.1 SSCL|APA12869.1

>Orthogroup2838: ANID|CBF79508.1 BCIN|XP_001552240.2 BGRA|VCU39215.1 CFRU|XP_031887656.1 CGLO|KAF3803329.1 CHIG|XP_018161156.1 CVIN|KAF4919273.1 CVYL|A03767 FGRM|XP_011318405.1 MGRA|XP_003849264.1 MLAR|XP_007415205.1 MORY|QBZ64576.1 NCRA|XP_961885.1 SSCL|APA12908.1

>Orthogroup2839: ANID|CBF79514.1 BCIN|XP_001558150.2 BGRA|VDB92699.1 CFRU|XP_031878771.1 CGLO|KAF3797566.1 CHIG|XP_018151105.1 CVIN|KAF4923592.1 CVYL|A08981 FGRM|XP_011324259.1 MGRA|XP_003849258.1 MLAR|XP_007406529.1 MORY|QBZ63336.1 NCRA|XP_957054.1 SSCL|APA13165.1

>Orthogroup2840: ANID|CBF79563.1 BCIN|XP_024553625.1 BGRA|VDB87864.1 CFRU|XP_031883266.1 CGLO|KAF3801730.1 CHIG|XP_018159245.1 CVIN|KAF4922912.1 CVYL|A12971 FGRM|XP_011320395.1 MGRA|XP_003853358.1 MLAR|XP_007405373.1 MORY|QBZ55469.1 NCRA|XP_960414.1 SSCL|APA08572.1

>Orthogroup2842: ANID|CBF79590.1 BCIN|XP_024553643.1 BGRA|VDB89723.1 CFRU|XP_031885752.1 CGLO|KAF3810183.1 CHIG|XP_018157802.1 CVIN|KAF4929172.1 CVYL|A02775 FGRM|XP_011320113.1 MGRA|XP_003852933.1 MLAR|XP_007410370.1 MORY|QBZ61281.1 NCRA|XP_960327.3 SSCL|APA08561.1

>Orthogroup2843: ANID|CBF79605.1 BCIN|XP_024547012.1 BGRA|VDB93748.1 CFRU|XP_031882863.1 CGLO|KAF3806660.1 CHIG|XP_018164208.1 CVIN|KAF4923680.1 CVYL|A14201 FGRM|XP_011316009.1 MGRA|XP_003857339.1 MLAR|XP_007404238.1 MORY|QBZ62973.1 NCRA|XP_965419.1 SSCL|APA05887.1

>Orthogroup2844: ANID|CBF79613.1 BCIN|XP_024550477.1 BGRA|VDB84195.1 CFRU|XP_031892563.1 CGLO|KAF3797231.1 CHIG|XP_018157228.1 CVIN|KAF4917704.1 CVYL|A10466 FGRM|XP_011320216.1 MGRA|XP_003856489.1 MLAR|XP_007403620.1 MORY|QBZ66126.1 NCRA|XP_965772.1 SSCL|APA11196.1

>Orthogroup2845: ANID|CBF79619.1 BCIN|XP_024549744.1 BGRA|VDB93686.1 CFRU|XP_031892726.1 CGLO|KAF3812259.1 CHIG|XP_018153452.1 CVIN|KAF4919938.1 CVYL|A00128 FGRM|XP_011316944.1 MGRA|XP_003856488.1 MLAR|XP_007412765.1 MORY|QBZ55265.1 NCRA|XP_011393048.1 SSCL|APA05677.1

>Orthogroup2846: ANID|CBF79621.1 BCIN|XP_001558891.1 BGRA|VDB93666.1 CFRU|XP_031879785.1 CGLO|KAF3802995.1 CHIG|XP_018153617.1 CVIN|KAF4929927.1 CVYL|A00006 FGRM|XP_011316277.1 MGRA|XP_003853759.1 MLAR|XP_007405832.1 MORY|QBZ55545.1 NCRA|XP_963722.1 SSCL|APA05785.1

>Orthogroup2847: ANID|CBF79630.1 BCIN|XP_001548490.1 BGRA|VDB86320.1 CFRU|XP_031888409.1 CGLO|KAF3803673.1 CHIG|XP_018163775.1 CVIN|KAF4928564.1 CVYL|A14603 FGRM|XP_011324168.1 MGRA|XP_003857051.1 MLAR|XP_007411508.1 MORY|QBZ54632.1 NCRA|XP_964493.1 SSCL|APA13834.1

>Orthogroup2848: ANID|CBF79641.1 BCIN|XP_001553724.1 BGRA|VDB93536.1 CFRU|XP_031892719.1 CGLO|KAF3812252.1 CHIG|XP_018153456.1 CVIN|KAF4919940.1 CVYL|A00133 FGRM|XP_011316936.1 MGRA|XP_003857141.1 MLAR|XP_007412207.1 MORY|QBZ55260.1 NCRA|XP_965348.1 SSCL|APA13488.1

>Orthogroup2849: ANID|CBF79661.1 BCIN|XP_001547942.1 BGRA|VDB93713.1 CFRU|XP_031882949.1 CGLO|KAF3803450.1 CHIG|XP_018163978.1 CVIN|KAF4926300.1 CVYL|A14378 FGRM|XP_011316977.1 MGRA|XP_003857344.1 MLAR|XP_007404870.1 MORY|QBZ55325.1 NCRA|XP_965462.3 SSCL|APA13608.1

>Orthogroup2850: ANID|CBF79684.1 BCIN|XP_024546943.1 BGRA|VDB86351.1 CFRU|XP_031879780.1 CGLO|KAF3802993.1 CHIG|XP_018153619.1 CVIN|KAF4929925.1 CVYL|A00004 FGRM|XP_011316276.1 MGRA|XP_003856591.1 MLAR|XP_007415659.1 MORY|QBZ62762.1 NCRA|XP_963721.2 SSCL|APA05788.1

>Orthogroup2852: ANID|CBF79722.1 BCIN|XP_024552225.1 BGRA|VCU39492.1 CFRU|XP_031887644.1 CGLO|KAF3803191.1 CHIG|XP_018161029.1 CVIN|KAF4920203.1 CVYL|A03621 FGRM|XP_011323812.1 MGRA|XP_003849636.1 MLAR|XP_007405344.1 MORY|QBZ58841.1 NCRA|XP_964449.1 SSCL|APA13008.1

>Orthogroup2853: ANID|CBF79724.1 BCIN|XP_001548685.1 BGRA|VDB88066.1 CFRU|XP_031883300.1 CGLO|KAF3800508.1 CHIG|XP_018152214.1 CVIN|KAF4919223.1 CVYL|A13206 FGRM|XP_011321322.1 MGRA|XP_003849464.1 MLAR|XP_007411848.1 MORY|QBZ61109.1 NCRA|XP_011393593.1 SSCL|APA15611.1

>Orthogroup2854: ANID|CBF79732.1 BCIN|XP_001555293.1 BGRA|VDB87671.1 CFRU|XP_031879417.1 CGLO|KAF3802420.1 CHIG|XP_018154718.1 CVIN|KAF4918214.1 CVYL|A13563 FGRM|XP_011317177.1 MGRA|XP_003849649.1 MLAR|XP_007417209.1 MORY|QBZ64864.1 NCRA|XP_956525.1 SSCL|APA08639.1

>Orthogroup2855: ANID|CBF79747.1 BCIN|XP_001551622.1 BGRA|VCU39991.1 CFRU|XP_031880272.1 CGLO|KAF3809081.1 CHIG|XP_018155889.1 CVIN|KAF4919721.1 CVYL|A01631 FGRM|XP_011319218.1 MGRA|XP_003849510.1 MLAR|XP_007408918.1 MORY|QBZ62422.1 NCRA|XP_001728228.2 SSCL|APA11778.1

>Orthogroup2856: ANID|CBF79766.1 BCIN|XP_001557372.1 BGRA|VDB91333.1 CFRU|XP_031893201.1 CGLO|KAF3808098.1 CHIG|XP_018152799.1 CVIN|KAF4920634.1 CVYL|A08134 FGRM|XP_011317975.1 MGRA|XP_003850530.1 MLAR|XP_007410388.1 MORY|QBZ55003.1 NCRA|XP_958539.1 SSCL|APA10701.1

>Orthogroup2857: ANID|CBF79770.1 BCIN|XP_024552757.1 BGRA|VCU41238.1 CFRU|XP_031887372.1 CGLO|KAF3809338.1 CHIG|XP_018156167.1 CVIN|KAF4924165.1 CVYL|A01541 FGRM|XP_011318847.1 MGRA|XP_003857183.1 MLAR|XP_007403705.1 MORY|QBZ58953.1 NCRA|XP_960913.1 SSCL|APA07502.1

>Orthogroup2858: ANID|CBF79783.1 BCIN|XP_024552810.1 BGRA|VCU41232.1 CFRU|XP_031889454.1 CGLO|KAF3808819.1 CHIG|XP_018155722.1 CVIN|KAF4908034.1 CVYL|A01259 FGRM|XP_011319147.1 MGRA|XP_003856782.1 MLAR|XP_007412725.1 MORY|QBZ58724.1 NCRA|XP_960697.1 SSCL|APA16023.1

>Orthogroup2864: ANID|CBF79845.1 BCIN|XP_001551325.2 BGRA|VCU41282.1 CFRU|XP_031892834.1 CGLO|KAF3809107.1 CHIG|XP_018155928.1 CVIN|KAF4922097.1 CVYL|A01293 FGRM|XP_011319020.1 MGRA|XP_003857424.1 MLAR|XP_007417488.1 MORY|QBZ57472.1 NCRA|XP_961526.1 SSCL|APA16136.1

>Orthogroup2865: ANID|CBF79881.1 BCIN|XP_024549075.1 BGRA|VCU39548.1 CFRU|XP_031885640.1 CGLO|KAF3810071.1 CHIG|XP_018157779.1 CVIN|KAF4912741.1 CVYL|A02667 FGRM|XP_011326177.1 MGRA|XP_003857785.1 MLAR|XP_007409470.1 MORY|QBZ58143.1 NCRA|XP_962130.3 SSCL|APA15865.1

>Orthogroup2866: ANID|CBF79887.1 BCIN|XP_001553180.1 BGRA|VCU39696.1 CFRU|XP_031887417.1 CGLO|KAF3809281.1 CHIG|XP_018156116.1 CVIN|KAF4921056.1 CVYL|A01475 FGRM|XP_011319358.1 MGRA|XP_003857398.1 MLAR|XP_007405878.1 MORY|QBZ62241.1 NCRA|XP_961083.1 SSCL|APA07531.1

>Orthogroup2867: ANID|CBF79904.1 BCIN|XP_001554553.2 BGRA|VCU40845.1 CFRU|XP_031887403.1 CGLO|KAF3809275.1 CHIG|XP_018156111.1 CVIN|KAF4921062.1 CVYL|A01469 FGRM|XP_011318834.1 MGRA|XP_003856038.1 MLAR|XP_007415649.1 MORY|QBZ62206.1 NCRA|XP_961236.1 SSCL|APA11905.1

>Orthogroup2869: ANID|CBF79908.1 BCIN|XP_001554559.2 BGRA|VCU40837.1 CFRU|XP_031889299.1 CGLO|KAF3808711.1 CHIG|XP_018155572.1 CVIN|KAF4929633.1 CVYL|A00973 FGRM|XP_011319269.1 MGRA|XP_003857577.1 MLAR|XP_007416243.1 MORY|QBZ59035.1 NCRA|XP_960899.1 SSCL|APA11910.1

>Orthogroup2870: ANID|CBF79923.1 BCIN|XP_001549468.1 BGRA|VCU41173.1 CFRU|XP_031892843.1 CGLO|KAF3809149.1 CHIG|XP_018155949.1 CVIN|KAF4922133.1 CVYL|A01334 FGRM|XP_011318803.1 MGRA|XP_003855933.1 MLAR|XP_007406422.1 MORY|QBZ62421.1 NCRA|XP_958665.3 SSCL|APA16234.1

>Orthogroup2871: ANID|CBF79929.1 BCIN|XP_024550493.1 BGRA|VCU38780.1 CFRU|XP_031881542.1 CGLO|KAF3805345.1 CHIG|XP_018156764.1 CVIN|KAF4913619.1 CVYL|A10987 FGRM|XP_011328302.1 MGRA|XP_003853880.1 MLAR|XP_007405317.1 MORY|QBZ65546.1 NCRA|XP_965198.2 SSCL|APA11212.1

>Orthogroup2872: ANID|CBF79934.1 BCIN|XP_024550491.1 BGRA|VCU38783.1 CFRU|XP_031881539.1 CGLO|KAF3805348.1 CHIG|XP_018156767.1 CVIN|KAF4913621.1 CVYL|A10990 FGRM|XP_011328299.1 MGRA|XP_003854198.1 MLAR|XP_007411302.1 MORY|QBZ66207.1 NCRA|XP_965202.1 SSCL|APA11209.1

>Orthogroup2874: ANID|CBF79939.1 BCIN|XP_024550490.1 BGRA|VCU38784.1 CFRU|XP_031881644.1 CGLO|KAF3807469.1 CHIG|XP_018156760.1 CVIN|KAF4913602.1 CVYL|A10980 FGRM|XP_011328298.1 MGRA|XP_003853184.1 MLAR|XP_007406448.1 MORY|QBZ66208.1 NCRA|XP_965201.1 SSCL|APA11208.1

>Orthogroup2875: ANID|CBF79943.1 BCIN|XP_001560598.1 BGRA|VDB85831.1 CFRU|XP_031887818.1 CGLO|KAF3802050.1 CHIG|XP_018154181.1 CVIN|KAF4901930.1 CVYL|A03338 FGRM|XP_011323000.1 MGRA|XP_003854301.1 MLAR|XP_007414670.1 MORY|QBZ66403.1 NCRA|XP_011392841.1 SSCL|APA07907.1

>Orthogroup2876: ANID|CBF79947.1 BCIN|XP_001560866.2 BGRA|VDB84399.1 CFRU|XP_031886690.1 CGLO|KAF3800272.1 CHIG|XP_018162759.1 CVIN|KAF4927611.1 CVYL|A06964 FGRM|XP_011327026.1 MGRA|XP_003849981.1 MLAR|XP_007417909.1 MORY|QBZ61331.1 NCRA|XP_957320.1 SSCL|APA08079.1

>Orthogroup2877: ANID|CBF79950.1 BCIN|XP_024547541.1 BGRA|VCU38819.1 CFRU|XP_031887032.1 CGLO|KAF3811281.1 CHIG|XP_018160505.1 CVIN|KAF4927222.1 CVYL|A04451 FGRM|XP_011323629.1 MGRA|XP_003850531.1 MLAR|XP_007418883.1 MORY|QBZ57015.1 NCRA|XP_961506.1 SSCL|APA07869.1

>Orthogroup2878: ANID|CBF79961.1 BCIN|XP_024545922.1 BGRA|VCU38914.1 CFRU|XP_031887454.1 CGLO|KAF3811598.1 CHIG|XP_018160870.1 CVIN|KAF4914900.1 CVYL|A03456 FGRM|XP_011324859.1 MGRA|XP_003853792.1 MLAR|XP_007418038.1 MORY|QBZ58877.1 NCRA|XP_962224.1 SSCL|APA07344.1

>Orthogroup2879: ANID|CBF79968.1 BCIN|XP_024550449.1 BGRA|VDB84185.1 CFRU|XP_031887841.1 CGLO|KAF3802276.1 CHIG|XP_018154358.1 CVIN|KAF4924438.1 CVYL|A10385 FGRM|XP_011319806.1 MGRA|XP_003854030.1 MLAR|XP_007408175.1 MORY|QBZ65751.1 NCRA|XP_964239.2 SSCL|APA11151.1

>Orthogroup2880: ANID|CBF79970.1 BCIN|XP_024546731.1 BGRA|VCU39101.1 CFRU|XP_031886199.1 CGLO|KAF3806585.1 CHIG|XP_018157624.1 CVIN|KAF4920498.1 CVYL|A09283 FGRM|XP_011324827.1 MGRA|XP_003855781.1 MLAR|XP_007416449.1 MORY|QBZ56235.1 NCRA|XP_962619.2 SSCL|APA06825.1

>Orthogroup2881: ANID|CBF79975.1 BCIN|XP_001555020.1 BGRA|VDB86137.1 CFRU|XP_031886986.1 CGLO|KAF3811255.1 CHIG|XP_018160497.1 CVIN|KAF4931238.1 CVYL|A04409 FGRM|XP_011324718.1 MGRA|XP_003850472.1 MLAR|XP_007410569.1 MORY|QBZ64489.1 NCRA|XP_001728316.1 SSCL|APA07789.1

>Orthogroup2882: ANID|CBF79976.1 BCIN|XP_001555007.1 BGRA|VDB86066.1 CFRU|XP_031891470.1 CGLO|KAF3804496.1 CHIG|XP_018160324.1 CVIN|KAF4930978.1 CVYL|A04211 FGRM|XP_011324747.1 MGRA|XP_003850519.1 MLAR|XP_007413426.1 MORY|QBZ59093.1 NCRA|XP_960927.1 SSCL|APA07781.1

>Orthogroup2883: ANID|CBF79979.1 BCIN|XP_024547788.1 BGRA|VCU38773.1 CFRU|XP_031891680.1 CGLO|KAF3800131.1 CHIG|XP_018165065.1 CVIN|KAF4925817.1 CVYL|A06818 FGRM|XP_011323518.1 MGRA|XP_003849894.1 MLAR|XP_007411133.1 MORY|QBZ58690.1 NCRA|XP_962257.1 SSCL|APA08047.1

>Orthogroup2884: ANID|CBF79990.1 BCIN|XP_024547783.1 BGRA|VCU38774.1 CFRU|XP_031877752.1 CGLO|KAF3796947.1 CHIG|XP_018162394.1 CVIN|KAF4911630.1 CVYL|A07317 FGRM|XP_011318548.1 MGRA|XP_003849944.1 MLAR|XP_007413799.1 MORY|QBZ62831.1 NCRA|XP_957236.2 SSCL|APA08044.1

>Orthogroup2885: ANID|CBF80000.1 BCIN|XP_024551195.1 BGRA|VCU39442.1 CFRU|XP_031886639.1 CGLO|KAF3801214.1 CHIG|XP_018162597.1 CVIN|KAF4932114.1 CVYL|A07118 FGRM|XP_011324118.1 MGRA|XP_003855401.1 MLAR|XP_007413583.1 MORY|QBZ56493.1 NCRA|XP_011393983.1 SSCL|APA10146.1

>Orthogroup2886: ANID|CBF80004.1 BCIN|XP_001553481.1 BGRA|VDB86018.1 CFRU|XP_031891543.1 CGLO|KAF3797436.1 CHIG|XP_018157399.1 CVIN|KAF4929364.1 CVYL|A10240 FGRM|XP_011319867.1 MGRA|XP_003854202.1 MLAR|XP_007414465.1 MORY|QBZ65615.1 NCRA|XP_964506.2 SSCL|APA10879.1

>Orthogroup2887: ANID|CBF80025.1 BCIN|XP_001549995.1 BGRA|VCU39961.1 CFRU|XP_031893215.1 CGLO|KAF3809497.1 CHIG|XP_018152684.1 CVIN|KAF4921254.1 CVYL|A10062 FGRM|XP_011317966.1 MGRA|XP_003850395.1 MLAR|XP_007417697.1 MORY|QBZ55063.1 NCRA|XP_011394828.1 SSCL|APA10468.1

>Orthogroup2888: ANID|CBF80034.1 BCIN|XP_001547476.1 BGRA|VCU38910.1 CFRU|XP_031887687.1 CGLO|KAF3811597.1 CHIG|XP_018160865.1 CVIN|KAF4914892.1 CVYL|A03455 FGRM|XP_011324860.1 MGRA|XP_003854443.1 MLAR|XP_007408287.1 MORY|QBZ58880.1 NCRA|XP_961806.1 SSCL|APA07348.1

>Orthogroup2889: ANID|CBF80037.1 BCIN|XP_024549278.1 BGRA|VCU38860.1 CFRU|XP_031887629.1 CGLO|KAF3803232.1 CHIG|XP_018161079.1 CVIN|KAF4928664.1 CVYL|A03664 FGRM|XP_011323830.1 MGRA|XP_003853695.1 MLAR|XP_007412709.1 MORY|QBZ56328.1 NCRA|XP_962504.1 SSCL|APA08896.1

>Orthogroup2890: ANID|CBF80046.1 BCIN|XP_024550497.1 BGRA|VDB85710.1 CFRU|XP_031887839.1 CGLO|KAF3802274.1 CHIG|XP_018154360.1 CVIN|KAF4924434.1 CVYL|A10363 FGRM|XP_011319801.1 MGRA|XP_003853821.1 MLAR|XP_007404372.1 MORY|QBZ65748.1 NCRA|XP_964237.3 SSCL|APA11215.1

>Orthogroup2891: ANID|CBF80058.1 BCIN|XP_024550576.1 BGRA|VDB84209.1 CFRU|XP_031880456.1 CGLO|KAF3807271.1 CHIG|XP_018156945.1 CVIN|KAF4928134.1 CVYL|A10754 FGRM|XP_011320001.1 MGRA|XP_003854506.1 MLAR|XP_007415379.1 MORY|QBZ65911.1 NCRA|XP_011392812.1 SSCL|APA11374.1

>Orthogroup2895: ANID|CBF80097.1 BCIN|XP_024547671.1 BGRA|VDB85944.1 CFRU|XP_031880391.1 CGLO|KAF3807263.1 CHIG|XP_018156941.1 CVIN|KAF4928183.1 CVYL|A10745 FGRM|XP_011320182.1 MGRA|XP_003854416.1 MLAR|XP_007409099.1 MORY|QBZ66169.1 NCRA|XP_011392943.1 SSCL|APA07734.1

>Orthogroup2904: ANID|CBF80662.1 BCIN|XP_001552102.1 BGRA|VDB96314.1 CFRU|XP_031886617.1 CGLO|KAF3801229.1 CHIG|XP_018162611.1 CVIN|KAF4932136.1 CVYL|A07101 FGRM|XP_011326639.1 MGRA|XP_003854386.1 MLAR|XP_007411201.1 MORY|QBZ62824.1 NCRA|XP_957229.2 SSCL|APA05288.1

>Orthogroup2907: ANID|CBF80860.1 BCIN|XP_024550647.1 BGRA|VCU39558.1 CFRU|XP_031892703.1 CGLO|KAF3809166.1 CHIG|XP_018155988.1 CVIN|KAF4919828.1 CVYL|A01353 FGRM|XP_011318968.1 MGRA|XP_003855390.1 MLAR|XP_007417616.1 MORY|QBZ57474.1 NCRA|XP_961531.1 SSCL|APA11466.1

>Orthogroup2909: ANID|CBF80871.1 BCIN|XP_001548814.1 BGRA|VDB93726.1 CFRU|XP_031881826.1 CGLO|KAF3800635.1 CHIG|XP_018152049.1 CVIN|KAF4918188.1 CVYL|A13081 FGRM|XP_011317344.1 MGRA|XP_003855224.1 MLAR|XP_007407883.1 MORY|QBZ57298.1 NCRA|XP_956269.1 SSCL|APA13343.1

>Orthogroup2910: ANID|CBF80879.1 BCIN|XP_001558766.2 BGRA|VDB93682.1 CFRU|XP_031878067.1 CGLO|KAF3799569.1 CHIG|XP_018154563.1 CVIN|KAF4926516.1 CVYL|A13428 FGRM|XP_011323705.1 MGRA|XP_003855257.1 MLAR|XP_007410662.1 MORY|QBZ61075.1 NCRA|XP_957181.3 SSCL|APA05612.1

>Orthogroup2911: ANID|CBF80886.1 BCIN|XP_024546977.1 BGRA|VDB93693.1 CFRU|XP_031877995.1 CGLO|KAF3799614.1 CHIG|XP_018154614.1 CVIN|KAF4926450.1 CVYL|A13476 FGRM|XP_011316550.1 MGRA|XP_003854856.1 MLAR|XP_007418173.1 MORY|QBZ54303.1 NCRA|XP_963372.2 SSCL|APA05956.1

>Orthogroup2912: ANID|CBF80890.1 BCIN|XP_001558870.1 BGRA|VDB93800.1 CFRU|XP_031883365.1 CGLO|KAF3800552.1 CHIG|XP_018152132.1 CVIN|KAF4923816.1 CVYL|A13165 FGRM|XP_011315966.1 MGRA|XP_003854862.1 MLAR|XP_007409934.1 MORY|QBZ60312.1 NCRA|XP_956027.1 SSCL|APA05773.1

>Orthogroup2913: ANID|CBF80896.1 BCIN|XP_001558873.1 BGRA|VDB93774.1 CFRU|XP_031883358.1 CGLO|KAF3800554.1 CHIG|XP_018152134.1 CVIN|KAF4923818.1 CVYL|A13163 FGRM|XP_011315968.1 MGRA|XP_003855472.1 MLAR|XP_007405602.1 MORY|QBZ60308.1 NCRA|XP_956911.1 SSCL|APA05775.1

>Orthogroup2914: ANID|CBF80900.1 BCIN|XP_001558830.1 BGRA|VDB93792.1 CFRU|XP_031883403.1 CGLO|KAF3800581.1 CHIG|XP_018152099.1 CVIN|KAF4927060.1 CVYL|A13134 FGRM|XP_011325671.1 MGRA|XP_003855092.1 MLAR|XP_007407797.1 MORY|QBZ62123.1 NCRA|XP_956250.1 SSCL|APA05982.1

>Orthogroup2915: ANID|CBF80901.1 BCIN|XP_001558829.1 BGRA|VDB93790.1 CFRU|XP_031883341.1 CGLO|KAF3800580.1 CHIG|XP_018152100.1 CVIN|KAF4927059.1 CVYL|A13135 FGRM|XP_011325672.1 MGRA|XP_003855403.1 MLAR|XP_007407063.1 MORY|QBZ62124.1 NCRA|XP_956253.3 SSCL|APA05981.1

>Orthogroup2916: ANID|CBF80905.1 BCIN|XP_024546956.1 BGRA|VDB93782.1 CFRU|XP_031883254.1 CGLO|KAF3800544.1 CHIG|XP_018152140.1 CVIN|KAF4923798.1 CVYL|A13172 FGRM|XP_011321218.1 MGRA|XP_003855218.1 MLAR|XP_007406623.1 MORY|QBZ60316.1 NCRA|XP_956902.3 SSCL|APA05995.1

>Orthogroup2917: ANID|CBF80920.1 BCIN|XP_001558836.1 BGRA|VDB93801.1 CFRU|XP_031881744.1 CGLO|KAF3800618.1 CHIG|XP_018152061.1 CVIN|KAF4918180.1 CVYL|A13099 FGRM|XP_011316338.1 MGRA|XP_003848926.1 MLAR|XP_007411038.1 MORY|QBZ60997.1 NCRA|XP_956124.1 SSCL|APA05988.1

>Orthogroup2918: ANID|CBF80924.1 BCIN|XP_024553302.1 BGRA|VDB93825.1 CFRU|XP_031883351.1 CGLO|KAF3800558.1 CHIG|XP_018152115.1 CVIN|KAF4923786.1 CVYL|A13159 FGRM|XP_011317237.1 MGRA|XP_003848815.1 MLAR|XP_007411140.1 MORY|QBZ57293.1 NCRA|XP_011393499.1 SSCL|APA13519.1

>Orthogroup2919: ANID|CBF80935.1 BCIN|XP_001558825.1 BGRA|VDB93841.1 CFRU|XP_031885352.1 CGLO|KAF3799455.1 CHIG|XP_018154443.1 CVIN|KAF4931797.1 CVYL|A13316 FGRM|XP_011325518.1 MGRA|XP_003849174.1 MLAR|XP_007415176.1 MORY|QBZ54369.1 NCRA|XP_963155.1 SSCL|APA05978.1

>Orthogroup2920: ANID|CBF80938.1 BCIN|XP_024550318.1 BGRA|VDB84167.1 CFRU|XP_031891305.1 CGLO|KAF3797387.1 CHIG|XP_018157120.1 CVIN|KAF4929390.1 CVYL|A10297 FGRM|XP_011319910.1 MGRA|XP_003850378.1 MLAR|XP_007403971.1 MORY|QBZ66041.1 NCRA|XP_964915.2 SSCL|APA10984.1

>Orthogroup2922: ANID|CBF80959.1 BCIN|XP_001545951.1 BGRA|VDB90808.1 CFRU|XP_031888507.1 CGLO|KAF3803532.1 CHIG|XP_018164056.1 CVIN|KAF4922273.1 CVYL|A14463 FGRM|XP_011326769.1 MGRA|XP_003851318.1 MLAR|XP_007417128.1 MORY|QBZ62815.1 NCRA|XP_958879.1 SSCL|APA07042.1

>Orthogroup2923: ANID|CBF80966.1 BCIN|XP_001549962.1 BGRA|VDB90824.1 CFRU|XP_031890326.1 CGLO|KAF3804873.1 CHIG|XP_018164405.1 CVIN|KAF4921154.1 CVYL|A12196 FGRM|XP_011316821.1 MGRA|XP_003851187.1 MLAR|XP_007413366.1 MORY|QBZ63632.1 NCRA|XP_011393122.1 SSCL|APA06624.1

>Orthogroup2924: ANID|CBF80975.1 BCIN|XP_001557517.1 BGRA|VDB93936.1 CFRU|XP_031883334.1 CGLO|KAF3800571.1 CHIG|XP_018152110.1 CVIN|KAF4927071.1 CVYL|A13144 FGRM|XP_011317250.1 MGRA|XP_003849087.1 MLAR|XP_007416558.1 MORY|QBZ60325.1 NCRA|XP_956039.1 SSCL|APA12603.1

>Orthogroup2925: ANID|CBF80992.1 BCIN|XP_001558781.1 BGRA|VDB96304.1 CFRU|XP_031876958.1 CGLO|KAF3810707.1 CHIG|XP_018162473.1 CVIN|KAF4908241.1 CVYL|A07247 FGRM|XP_011323992.1 MGRA|XP_003853984.1 MLAR|XP_007411150.1 MORY|QBZ61447.1 NCRA|XP_957122.1 SSCL|APA05245.1

>Orthogroup2926: ANID|CBF80996.1 BCIN|XP_001547113.1 BGRA|VDB83626.1 CFRU|XP_031882583.1 CGLO|KAF3803943.1 CHIG|XP_018151003.1 CVIN|KAF4925160.1 CVYL|A08860 FGRM|XP_011323845.1 MGRA|XP_003849170.1 MLAR|XP_007417202.1 MORY|QBZ56336.1 NCRA|XP_962509.2 SSCL|APA08396.1

>Orthogroup2927: ANID|CBF81000.1 BCIN|XP_024552112.1 BGRA|VDB92735.1 CFRU|XP_031880697.1 CGLO|KAF3809966.1 CHIG|XP_018161548.1 CVIN|KAF4914192.1 CVYL|A01657 FGRM|XP_011321386.1 MGRA|XP_003849168.1 MLAR|XP_007411555.1 MORY|QBZ65111.1 NCRA|XP_958709.1 SSCL|APA12945.1

>Orthogroup2930: ANID|CBF81013.1 BCIN|XP_024551010.1 BGRA|VDB89527.1 CFRU|XP_031889187.1 CGLO|KAF3808769.1 CHIG|XP_018155669.1 CVIN|KAF4885136.1 CVYL|A00894 FGRM|XP_011318981.1 MGRA|XP_003851311.1 MLAR|XP_007406379.1 MORY|QBZ54559.1 NCRA|XP_959205.3 SSCL|APA11921.1

>Orthogroup2931: ANID|CBF81026.1 BCIN|XP_001560295.2 BGRA|VDB92567.1 CFRU|XP_031879557.1 CGLO|KAF3802713.1 CHIG|XP_018151495.1 CVIN|KAF4894054.1 CVYL|A08040 FGRM|XP_011327260.1 MGRA|XP_003852881.1 MLAR|XP_007406103.1 MORY|QBZ56965.1 NCRA|XP_960030.1 SSCL|APA08833.1

>Orthogroup2932: ANID|CBF81056.1 BCIN|XP_024549180.1 BGRA|VCU39886.1 CFRU|XP_031889547.1 CGLO|KAF3810475.1 CHIG|XP_018159146.1 CVIN|KAF4924671.1 CVYL|A14076 FGRM|XP_011317184.1 MGRA|XP_003856204.1 MLAR|XP_007416678.1 MORY|QBZ56380.1 NCRA|XP_965675.1 SSCL|APA08749.1

>Orthogroup2933: ANID|CBF81059.1 BCIN|XP_001555615.1 BGRA|VCU40835.1 CFRU|XP_031889804.1 CGLO|KAF3810502.1 CHIG|XP_018159114.1 CVIN|KAF4924677.1 CVYL|A14107 FGRM|XP_011317195.1 MGRA|XP_003852756.1 MLAR|XP_007418623.1 MORY|QBZ56970.1 NCRA|XP_959268.2 SSCL|APA07316.1

>Orthogroup2934: ANID|CBF81112.1 BCIN|XP_001560156.1 BGRA|VDB92555.1 CFRU|XP_031886738.1 CGLO|KAF3806777.1 CHIG|XP_018158770.1 CVIN|KAF4905649.1 CVYL|A04991 FGRM|XP_011316367.1 MGRA|XP_003853041.1 MLAR|XP_007408686.1 MORY|QBZ60421.1 NCRA|XP_964961.1 SSCL|APA15863.1

>Orthogroup2935: ANID|CBF81168.1 BCIN|XP_024546602.1 BGRA|VDB90822.1 CFRU|XP_031892576.1 CGLO|KAF3803061.1 CHIG|XP_018153533.1 CVIN|KAF4919845.1 CVYL|A00071 FGRM|XP_011316127.1 MGRA|XP_003851081.1 MLAR|XP_007412534.1 MORY|QBZ63867.1 NCRA|XP_001727995.2 SSCL|APA06429.1

>Orthogroup2936: ANID|CBF81172.1 BCIN|XP_024549491.1 BGRA|VDB94999.1 CFRU|XP_031886976.1 CGLO|KAF3811373.1 CHIG|XP_018160609.1 CVIN|KAF4921033.1 CVYL|A04565 FGRM|XP_011327212.1 MGRA|XP_003850760.1 MLAR|XP_007417902.1 MORY|QBZ56904.1 NCRA|XP_961567.1 SSCL|APA09151.1

>Orthogroup2937: ANID|CBF81176.1 BCIN|XP_001561262.1 BGRA|VDB90541.1 CFRU|XP_031882771.1 CGLO|KAF3803445.1 CHIG|XP_018163973.1 CVIN|KAF4926303.1 CVYL|A14373 FGRM|XP_011316972.1 MGRA|XP_003851280.1 MLAR|XP_007404548.1 MORY|QBZ63005.1 NCRA|XP_957994.3 SSCL|APA07203.1

>Orthogroup2938: ANID|CBF81178.1 BCIN|XP_001561003.1 BGRA|VDB90772.1 CFRU|XP_031879819.1 CGLO|KAF3802980.1 CHIG|XP_018153633.1 CVIN|KAF4929903.1 CVYL|A00412 FGRM|XP_011316933.1 MGRA|XP_003850632.1 MLAR|XP_007408660.1 MORY|QBZ63614.1 NCRA|XP_963689.1 SSCL|APA06728.1

>Orthogroup2939: ANID|CBF81185.1 BCIN|XP_001561121.1 BGRA|VDB92487.1 CFRU|XP_031884196.1 CGLO|KAF3811023.1 CHIG|XP_018153013.1 CVIN|KAF4918154.1 CVYL|A09756 FGRM|XP_011324315.1 MGRA|XP_003850590.1 MLAR|XP_007412295.1 MORY|QBZ53378.1 NCRA|XP_964931.2 SSCL|APA07125.1

>Orthogroup2940: ANID|CBF81187.1 BCIN|XP_001561122.1 BGRA|VDB92485.1 CFRU|XP_031884141.1 CGLO|KAF3800022.1 CHIG|XP_018153022.1 CVIN|KAF4926909.1 CVYL|A09782 FGRM|XP_011324308.1 MGRA|XP_003851284.1 MLAR|XP_007418273.1 MORY|QBZ64167.1 NCRA|XP_964933.1 SSCL|APA07124.1

>Orthogroup2941: ANID|CBF81189.1 BCIN|XP_001561123.1 BGRA|VDB92483.1 CFRU|XP_031884142.1 CGLO|KAF3800021.1 CHIG|XP_018153021.1 CVIN|KAF4926910.1 CVYL|A09783 FGRM|XP_011324307.1 MGRA|XP_003850593.1 MLAR|XP_007417812.1 MORY|QBZ64166.1 NCRA|XP_964934.1 SSCL|APA07123.1

>Orthogroup2942: ANID|CBF81193.1 BCIN|XP_001548981.2 BGRA|VDB90773.1 CFRU|XP_031879859.1 CGLO|KAF3803035.1 CHIG|XP_018153581.1 CVIN|KAF4929931.1 CVYL|A00047 FGRM|XP_011317134.1 MGRA|XP_003850649.1 MLAR|XP_007412847.1 MORY|QBZ59484.1 NCRA|XP_962088.2 SSCL|APA07022.1

>Orthogroup2943: ANID|CBF81194.1 BCIN|XP_001548980.1 BGRA|VDB90787.1 CFRU|XP_031879826.1 CGLO|KAF3803034.1 CHIG|XP_018153580.1 CVIN|KAF4929932.1 CVYL|A00045 FGRM|XP_011317135.1 MGRA|XP_003850648.1 MLAR|XP_007411709.1 MORY|QBZ59485.1 NCRA|XP_962089.1 SSCL|APA07023.1

>Orthogroup2944: ANID|CBF81198.1 BCIN|XP_001561260.1 BGRA|VDB90567.1 CFRU|XP_031888555.1 CGLO|KAF3803601.1 CHIG|XP_018163864.1 CVIN|KAF4920388.1 CVYL|A14536 FGRM|XP_011316779.1 MGRA|XP_003850762.1 MLAR|XP_007416067.1 MORY|QBZ63003.1 NCRA|XP_957992.2 SSCL|APA07205.1

>Orthogroup2945: ANID|CBF81213.1 BCIN|XP_001561306.1 BGRA|VDB90746.1 CFRU|XP_031890529.1 CGLO|KAF3804864.1 CHIG|XP_018164396.1 CVIN|KAF4921175.1 CVYL|A12207 FGRM|XP_011316300.1 MGRA|XP_003850665.1 MLAR|XP_007409922.1 MORY|QBZ55297.1 NCRA|XP_964276.1 SSCL|APA06856.1

>Orthogroup2947: ANID|CBF81301.1 BCIN|XP_024548309.1 BGRA|VCU40977.1 CFRU|XP_031892559.1 CGLO|KAF3809144.1 CHIG|XP_018155952.1 CVIN|KAF4922108.1 CVYL|A01329 FGRM|XP_011319183.1 MGRA|XP_003848062.1 MLAR|XP_007408709.1 MORY|QBZ56121.1 NCRA|XP_958841.2 SSCL|APA12558.1

>Orthogroup2948: ANID|CBF81316.1 BCIN|XP_001557210.2 BGRA|VCU40434.1 CFRU|XP_031886188.1 CGLO|KAF3801126.1 CHIG|XP_018155236.1 CVIN|KAF4930389.1 CVYL|A05718 FGRM|XP_011317468.1 MGRA|XP_003847980.1 MLAR|XP_007410293.1 MORY|QBZ60913.1 NCRA|XP_963243.1 SSCL|APA12502.1

>Orthogroup2949: ANID|CBF81333.1 BCIN|XP_001557800.1 BGRA|VCU41339.1 CFRU|XP_031878010.1 CGLO|KAF3799595.1 CHIG|XP_018154590.1 CVIN|KAF4926456.1 CVYL|A13456 FGRM|XP_011323713.1 MGRA|XP_003848074.1 MLAR|XP_007408658.1 MORY|QBZ61081.1 NCRA|XP_957190.3 SSCL|APA12144.1

>Orthogroup2950: ANID|CBF81335.1 BCIN|XP_001557801.1 BGRA|VCU41338.1 CFRU|XP_031881321.1 CGLO|KAF3802509.1 CHIG|XP_018154814.1 CVIN|KAF4919021.1 CVYL|A02101 FGRM|XP_011325540.1 MGRA|XP_003848058.1 MLAR|XP_007406572.1 MORY|QBZ53784.1 NCRA|XP_956302.1 SSCL|APA12143.1

>Orthogroup2951: ANID|CBF81341.1 BCIN|XP_024548366.1 BGRA|VDB93231.1 CFRU|XP_031889206.1 CGLO|KAF3808789.1 CHIG|XP_018155695.1 CVIN|KAF4906825.1 CVYL|A00876 FGRM|XP_011319336.1 MGRA|XP_003847966.1 MLAR|XP_007408544.1 MORY|QBZ58938.1 NCRA|XP_960683.2 SSCL|APA12348.1

>Orthogroup2952: ANID|CBF81345.1 BCIN|XP_001551082.2 BGRA|VDB83774.1 CFRU|XP_031892042.1 CGLO|KAF3801419.1 CHIG|XP_018164745.1 CVIN|KAF4922332.1 CVYL|A07666 FGRM|XP_011324963.1 MGRA|XP_003848619.1 MLAR|XP_007412928.1 MORY|QBZ64377.1 NCRA|XP_001727992.2 SSCL|APA14259.1

>Orthogroup2953: ANID|CBF81347.1 BCIN|XP_024547195.1 BGRA|VDB83750.1 CFRU|XP_031877306.1 CGLO|KAF3799869.1 CHIG|XP_018153823.1 CVIN|KAF4921713.1 CVYL|A09648 FGRM|XP_011325432.1 MGRA|XP_003848355.1 MLAR|XP_007416895.1 MORY|QBZ59974.1 NCRA|XP_955943.1 SSCL|APA14122.1

>Orthogroup2954: ANID|CBF81350.1 BCIN|XP_024547263.1 BGRA|VDB83778.1 CFRU|XP_031892073.1 CGLO|KAF3801416.1 CHIG|XP_018164743.1 CVIN|KAF4922328.1 CVYL|A07670 FGRM|XP_011324960.1 MGRA|XP_003848545.1 MLAR|XP_007410851.1 MORY|QBZ60870.1 NCRA|XP_958939.1 SSCL|APA14226.1

>Orthogroup2955: ANID|CBF81354.1 BCIN|XP_001554333.1 BGRA|VCU39343.1 CFRU|XP_031882006.1 CGLO|KAF3806140.1 CHIG|XP_018164521.1 CVIN|KAF4907017.1 CVYL|A07367 FGRM|XP_011325006.1 MGRA|XP_003848463.1 MLAR|XP_007415957.1 MORY|QBZ62363.1 NCRA|XP_959966.1 SSCL|APA14416.1

>Orthogroup2956: ANID|CBF81367.1 BCIN|XP_024547322.1 BGRA|VDB88924.1 CFRU|XP_031882120.1 CGLO|KAF3806103.1 CHIG|XP_018164574.1 CVIN|KAF4902332.1 CVYL|A07410 FGRM|XP_011325003.1 MGRA|XP_003851529.1 MLAR|XP_007407089.1 MORY|QBZ62377.1 NCRA|XP_959553.1 SSCL|APA14289.1

>Orthogroup2957: ANID|CBF81369.1 BCIN|XP_024547323.1 BGRA|VDB88922.1 CFRU|XP_031891842.1 CGLO|KAF3800153.1 CHIG|XP_018165084.1 CVIN|KAF4925859.1 CVYL|A06847 FGRM|XP_011327695.1 MGRA|XP_003851528.1 MLAR|XP_007405433.1 MORY|QBZ60652.1 NCRA|XP_955935.1 SSCL|APA14288.1

>Orthogroup2958: ANID|CBF81376.1 BCIN|XP_001546372.1 BGRA|VDB88198.1 CFRU|XP_031877742.1 CGLO|KAF3797361.1 CHIG|XP_018157149.1 CVIN|KAF4929378.1 CVYL|A10328 FGRM|XP_011320090.1 MGRA|XP_003853359.1 MLAR|XP_007416889.1 MORY|QBZ65947.1 NCRA|XP_964621.1 SSCL|APA12028.1

>Orthogroup2959: ANID|CBF81383.1 BCIN|XP_001549338.2 BGRA|VDB91198.1 CFRU|XP_031879183.1 CGLO|KAF3807077.1 CHIG|XP_018159738.1 CVIN|KAF4919049.1 CVYL|A06371 FGRM|XP_011320690.1 MGRA|XP_003854644.1 MLAR|XP_007411668.1 MORY|QBZ63987.1 NCRA|XP_960568.1 SSCL|APA06398.1

>Orthogroup2961: ANID|CBF81427.1 BCIN|XP_024546637.1 BGRA|VDB90547.1 CFRU|XP_031884284.1 CGLO|KAF3799982.1 CHIG|XP_018152994.1 CVIN|KAF4926876.1 CVYL|A10202 FGRM|XP_011324316.1 MGRA|XP_003851091.1 MLAR|XP_007415361.1 MORY|QBZ64130.1 NCRA|XP_011392830.1 SSCL|APA06470.1

>Orthogroup2962: ANID|CBF81433.1 BCIN|XP_001550622.1 BGRA|VDB88978.1 CFRU|XP_031882070.1 CGLO|KAF3806001.1 CHIG|XP_018164648.1 CVIN|KAF4925704.1 CVYL|A07513 FGRM|XP_011324994.1 MGRA|XP_003848420.1 MLAR|XP_007417262.1 MORY|QBZ62329.1 NCRA|XP_959924.1 SSCL|APA14137.1

>Orthogroup2963: ANID|CBF81435.1 BCIN|XP_024547208.1 BGRA|VDB88980.1 CFRU|XP_031882069.1 CGLO|KAF3806000.1 CHIG|XP_018164647.1 CVIN|KAF4925711.1 CVYL|A07514 FGRM|XP_011324709.1 MGRA|XP_003848265.1 MLAR|XP_007413960.1 MORY|QBZ62330.1 NCRA|XP_959925.2 SSCL|APA14136.1

>Orthogroup2964: ANID|CBF81444.1 BCIN|XP_001554555.1 BGRA|VCU40839.1 CFRU|XP_031889253.1 CGLO|KAF3808718.1 CHIG|XP_018155546.1 CVIN|KAF4929565.1 CVYL|A00942 FGRM|XP_011319273.1 MGRA|XP_003856015.1 MLAR|XP_007404460.1 MORY|QBZ59254.1 NCRA|XP_958040.2 SSCL|APA11907.1

>Orthogroup2966: ANID|CBF81484.1 BCIN|XP_001560604.1 BGRA|VDB94562.1 CFRU|XP_031891033.1 CGLO|KAF3801599.1 CHIG|XP_018151364.1 CVIN|KAF4922681.1 CVYL|A12536 FGRM|XP_011328216.1 MGRA|XP_003854437.1 MLAR|XP_007415113.1 MORY|QBZ59064.1 NCRA|XP_960422.1 SSCL|APA07920.1

>Orthogroup2967: ANID|CBF81503.1 BCIN|XP_001546253.1 BGRA|VDB93333.1 CFRU|XP_031882512.1 CGLO|KAF3809434.1 CHIG|XP_018156284.1 CVIN|KAF4921378.1 CVYL|A03104 FGRM|XP_011319046.1 MGRA|XP_003856066.1 MLAR|XP_007410768.1 MORY|QBZ62547.1 NCRA|XP_960626.1 SSCL|APA12532.1

>Orthogroup2968: ANID|CBF81519.1 BCIN|XP_001549682.1 BGRA|VDB94845.1 CFRU|XP_031885644.1 CGLO|KAF3810269.1 CHIG|XP_018157925.1 CVIN|KAF4918595.1 CVYL|A02871 FGRM|XP_011328519.1 MGRA|XP_003854480.1 MLAR|XP_007410853.1 MORY|QBZ62616.1 NCRA|XP_957659.1 SSCL|APA11392.1

>Orthogroup2969: ANID|CBF81523.1 BCIN|XP_024547761.1 BGRA|VDB90439.1 CFRU|XP_031883303.1 CGLO|KAF3801697.1 CHIG|XP_018159214.1 CVIN|KAF4915643.1 CVYL|A13001 FGRM|XP_011320276.1 MGRA|XP_003853997.1 MLAR|XP_007415804.1 MORY|QBZ55467.1 NCRA|XP_960410.1 SSCL|APA08019.1

>Orthogroup2971: ANID|CBF81540.1 BCIN|XP_001560763.2 BGRA|VDB90445.1 CFRU|XP_031883265.1 CGLO|KAF3801731.1 CHIG|XP_018159246.1 CVIN|KAF4922911.1 CVYL|A12970 FGRM|XP_011320277.1 MGRA|XP_003854010.1 MLAR|XP_007416888.1 MORY|QBZ62851.1 NCRA|XP_960153.2 SSCL|APA08018.1

>Orthogroup2972: ANID|CBF81555.1 BCIN|XP_024548068.1 BGRA|VDB83895.1 CFRU|XP_031892382.1 CGLO|KAF3809136.1 CHIG|XP_018154764.1 CVIN|KAF4922112.1 CVYL|A01320 FGRM|XP_011324661.1 MGRA|XP_003855519.1 MLAR|XP_007418372.1 MORY|QBZ66190.1 NCRA|XP_965396.1 SSCL|APA12760.1

>Orthogroup2973: ANID|CBF81592.1 BCIN|XP_001555455.1 BGRA|VDB94560.1 CFRU|XP_031885575.1 CGLO|KAF3810258.1 CHIG|XP_018157936.1 CVIN|KAF4918604.1 CVYL|A02861 FGRM|XP_011328234.1 MGRA|XP_003853693.1 MLAR|XP_007410980.1 MORY|QBZ56072.1 NCRA|XP_011394986.1 SSCL|APA08173.1

>Orthogroup2974: ANID|CBF81601.1 BCIN|XP_024548590.1 BGRA|VDB89891.1 CFRU|XP_031891092.1 CGLO|KAF3811750.1 CHIG|XP_018151789.1 CVIN|KAF4923015.1 CVYL|A12765 FGRM|XP_011319503.1 MGRA|XP_003853767.1 MLAR|XP_007411369.1 MORY|QBZ61938.1 NCRA|XP_959830.3 SSCL|APA10003.1

>Orthogroup2975: ANID|CBF81608.1 BCIN|XP_024548762.1 BGRA|VDB89564.1 CFRU|XP_031879505.1 CGLO|KAF3802383.1 CHIG|XP_018154697.1 CVIN|KAF4922170.1 CVYL|A13526 FGRM|XP_011317284.1 MGRA|XP_003853996.1 MLAR|XP_007414643.1 MORY|QBZ64825.1 NCRA|XP_956468.3 SSCL|APA09721.1

>Orthogroup2976: ANID|CBF81629.1 BCIN|XP_001552063.2 BGRA|VDB89351.1 CFRU|XP_031880145.1 CGLO|KAF3797744.1 CHIG|XP_018151702.1 CVIN|KAF4917553.1 CVYL|A11409 FGRM|XP_011319524.1 MGRA|XP_003854530.1 MLAR|XP_007410906.1 MORY|QBZ61244.1 NCRA|XP_960097.1 SSCL|APA09996.1

>Orthogroup2977: ANID|CBF81630.1 BCIN|XP_001552056.1 BGRA|VCU40444.1 CFRU|XP_031880092.1 CGLO|KAF3811822.1 CHIG|XP_018151741.1 CVIN|KAF4924892.1 CVYL|A12825 FGRM|XP_011319520.1 MGRA|XP_003854199.1 MLAR|XP_007410773.1 MORY|QBZ63060.1 NCRA|XP_958220.1 SSCL|APA10000.1

>Orthogroup2978: ANID|CBF81638.1 BCIN|XP_024548834.1 BGRA|VDB89575.1 CFRU|XP_031880034.1 CGLO|KAF3811788.1 CHIG|XP_018151713.1 CVIN|KAF4907649.1 CVYL|A12793 FGRM|XP_011321147.1 MGRA|XP_003853770.1 MLAR|XP_007417061.1 MORY|QBZ63139.1 NCRA|XP_959486.3 SSCL|APA09601.1

>Orthogroup2979: ANID|CBF81645.1 BCIN|XP_001559852.1 BGRA|VDB89568.1 CFRU|XP_031885391.1 CGLO|KAF3799437.1 CHIG|XP_018152314.1 CVIN|KAF4931805.1 CVYL|A13299 FGRM|XP_011315945.1 MGRA|XP_003847737.1 MLAR|XP_007415336.1 MORY|QBZ64800.1 NCRA|XP_956052.2 SSCL|APA09713.1

>Orthogroup2980: ANID|CBF81691.1 BCIN|XP_024552569.1 BGRA|VDB87655.1 CFRU|XP_031884231.1 CGLO|KAF3811053.1 CHIG|XP_018153129.1 CVIN|KAF4918139.1 CVYL|A09737 FGRM|XP_011322026.1 MGRA|XP_003856947.1 MLAR|XP_007405892.1 MORY|QBZ62845.1 NCRA|XP_960435.2 SSCL|APA08689.1

>Orthogroup2982: ANID|CBF81719.1 BCIN|XP_001558479.1 BGRA|VDB94986.1 CFRU|XP_031883993.1 CGLO|KAF3805143.1 CHIG|XP_018163370.1 CVIN|KAF4927409.1 CVYL|A06413 FGRM|XP_011321684.1 MGRA|XP_003857440.1 MLAR|XP_007414616.1 MORY|QBZ63278.1 NCRA|XP_956916.1 SSCL|APA14714.1

>Orthogroup2983: ANID|CBF81724.1 BCIN|XP_001560947.1 BGRA|VCU40606.1 CFRU|XP_031889201.1 CGLO|KAF3803168.1 CHIG|XP_018155507.1 CVIN|KAF4928346.1 CVYL|A01055 FGRM|XP_011318125.1 MGRA|XP_003857064.1 MLAR|XP_007416826.1 MORY|QBZ56814.1 NCRA|XP_957647.1 SSCL|APA06679.1

>Orthogroup2984: ANID|CBF81734.1 BCIN|XP_024547217.1 BGRA|VDB89066.1 CFRU|XP_031877628.1 CGLO|KAF3801067.1 CHIG|XP_018153713.1 CVIN|KAF4902052.1 CVYL|A00113 FGRM|XP_011327836.1 MGRA|XP_003851823.1 MLAR|XP_007406357.1 MORY|QBZ60559.1 NCRA|XP_011395169.1 SSCL|APA14141.1

>Orthogroup2985: ANID|CBF81738.1 BCIN|XP_001551017.1 BGRA|VDB88929.1 CFRU|XP_031877622.1 CGLO|KAF3801099.1 CHIG|XP_018153750.1 CVIN|KAF4909550.1 CVYL|A11296 FGRM|XP_011327809.1 MGRA|XP_003851474.1 MLAR|XP_007411906.1 MORY|QBZ60575.1 NCRA|XP_959773.1 SSCL|APA14294.1

>Orthogroup2986: ANID|CBF81739.1 BCIN|XP_024547185.1 BGRA|VCU40056.1 CFRU|XP_031877611.1 CGLO|KAF3801077.1 CHIG|XP_018153725.1 CVIN|KAF4892468.1 CVYL|A00002 FGRM|XP_011327797.1 MGRA|XP_003848275.1 MLAR|XP_007406581.1 MORY|QBZ60614.1 NCRA|XP_959785.1 SSCL|APA14113.1

>Orthogroup2988: ANID|CBF81796.1 BCIN|XP_001552345.1 BGRA|VDB94000.1 CFRU|XP_031885846.1 CGLO|KAF3810165.1 CHIG|XP_018157784.1 CVIN|KAF4929155.1 CVYL|A02757 FGRM|XP_011320316.1 MGRA|XP_003854290.1 MLAR|XP_007414765.1 MORY|QBZ62132.1 NCRA|XP_958636.1 SSCL|APA11176.1

>Orthogroup2989: ANID|CBF81800.1 BCIN|XP_024548900.1 BGRA|VDB84121.1 CFRU|XP_031890414.1 CGLO|KAF3805257.1 CHIG|XP_018158819.1 CVIN|KAF4918682.1 CVYL|A13906 FGRM|XP_011317314.1 MGRA|XP_003852797.1 MLAR|XP_007411049.1 MORY|QBZ60371.1 NCRA|XP_011393191.1 SSCL|APA09383.1

>Orthogroup2991: ANID|CBF81819.1 BCIN|XP_024548901.1 BGRA|VDB84127.1 CFRU|XP_031890415.1 CGLO|KAF3805258.1 CHIG|XP_018158818.1 CVIN|KAF4918681.1 CVYL|A13907 FGRM|XP_011317313.1 MGRA|XP_003848017.1 MLAR|XP_007406442.1 MORY|QBZ60372.1 NCRA|XP_965247.1 SSCL|APA09382.1

>Orthogroup2992: ANID|CBF81826.1 BCIN|XP_024546370.1 BGRA|VDB90860.1 CFRU|XP_031890455.1 CGLO|KAF3804947.1 CHIG|XP_018164453.1 CVIN|KAF4910227.1 CVYL|A12106 FGRM|XP_011316862.1 MGRA|XP_003856113.1 MLAR|XP_007408741.1 MORY|QBZ54732.1 NCRA|XP_011393267.1 SSCL|APA06843.1

>Orthogroup2993: ANID|CBF81828.1 BCIN|XP_001561296.1 BGRA|VDB90837.1 CFRU|XP_031890596.1 CGLO|KAF3804819.1 CHIG|XP_018164321.1 CVIN|KAF4929830.1 CVYL|A12256 FGRM|XP_011317046.1 MGRA|XP_003853503.1 MLAR|XP_007405119.1 MORY|QBZ54734.1 NCRA|XP_964259.2 SSCL|APA06841.1

>Orthogroup2994: ANID|CBF81874.1 BCIN|XP_024546545.1 BGRA|VDB90813.1 CFRU|XP_031890598.1 CGLO|KAF3804817.1 CHIG|XP_018164323.1 CVIN|KAF4929821.1 CVYL|A12258 FGRM|XP_011317048.1 MGRA|XP_003857523.1 MLAR|XP_007403498.1 MORY|QBZ54736.1 NCRA|XP_964262.1 SSCL|APA06716.1

>Orthogroup2995: ANID|CBF81885.1 BCIN|XP_024551351.1 BGRA|VDB89122.1 CFRU|XP_031878378.1 CGLO|KAF3810772.1 CHIG|XP_018162543.1 CVIN|KAF4932004.1 CVYL|A07181 FGRM|XP_011326983.1 MGRA|XP_003855262.1 MLAR|XP_007414305.1 MORY|QBZ61353.1 NCRA|XP_011393827.1 SSCL|APA10509.1

>Orthogroup2996: ANID|CBF81901.1 BCIN|XP_001557448.1 BGRA|VCU41027.1 CFRU|XP_031877175.1 CGLO|KAF3806412.1 CHIG|XP_018162295.1 CVIN|KAF4923262.1 CVYL|A12005 FGRM|XP_011326559.1 MGRA|XP_003855665.1 MLAR|XP_007411725.1 MORY|QBZ63911.1 NCRA|XP_956795.1 SSCL|APA10656.1

>Orthogroup2997: ANID|CBF81904.1 BCIN|XP_001557449.1 BGRA|VCU41030.1 CFRU|XP_031877172.1 CGLO|KAF3806413.1 CHIG|XP_018162296.1 CVIN|KAF4923261.1 CVYL|A12006 FGRM|XP_011326558.1 MGRA|XP_003855088.1 MLAR|XP_007414719.1 MORY|QBZ63912.1 NCRA|XP_956794.2 SSCL|APA10655.1

>Orthogroup2999: ANID|CBF82045.1 BCIN|XP_024549516.1 BGRA|VDB86321.1 CFRU|XP_031890085.1 CGLO|KAF3798734.1 CHIG|XP_018151259.1 CVIN|KAF4903212.1 CVYL|A09176 FGRM|XP_011328075.1 MGRA|XP_003851900.1 MLAR|XP_007408854.1 MORY|QBZ55233.1 NCRA|XP_962117.1 SSCL|APA09125.1

>Orthogroup3000: ANID|CBF82067.1 BCIN|XP_001559113.1 BGRA|VDB94011.1 CFRU|XP_031887594.1 CGLO|KAF3811638.1 CHIG|XP_018160913.1 CVIN|KAF4921459.1 CVYL|A03499 FGRM|XP_011323582.1 MGRA|XP_003856181.1 MLAR|XP_007410755.1 MORY|QBZ56596.1 NCRA|XP_964432.2 SSCL|APA09047.1

>Orthogroup3001: ANID|CBF82072.1 BCIN|XP_001547369.1 BGRA|VDB83578.1 CFRU|XP_031893388.1 CGLO|KAF3800850.1 CHIG|XP_018150844.1 CVIN|KAF4915585.1 CVYL|A11090 FGRM|XP_011328042.1 MGRA|XP_003857651.1 MLAR|XP_007412980.1 MORY|QBZ57982.1 NCRA|XP_962489.1 SSCL|APA09255.1

>Orthogroup3005: ANID|CBF82425.1 BCIN|XP_024552309.1 BGRA|VDB92845.1 CFRU|XP_031878863.1 CGLO|KAF3802632.1 CHIG|XP_018161290.1 CVIN|KAF4921640.1 CVYL|A01926 FGRM|XP_011318493.1 MGRA|XP_003849162.1 MLAR|XP_007406014.1 MORY|QBZ65086.1 NCRA|XP_962442.1 SSCL|APA13264.1

>Orthogroup3006: ANID|CBF82451.1 BCIN|XP_024546171.1 BGRA|VDB90680.1 CFRU|XP_031890638.1 CGLO|KAF3804922.1 CHIG|XP_018164420.1 CVIN|KAF4919425.1 CVYL|A12134 FGRM|XP_011316200.1 MGRA|XP_003855885.1 MLAR|XP_007412848.1 MORY|QBZ56461.1 NCRA|XP_964453.2 SSCL|APA07058.1

>Orthogroup3009: ANID|CBF82499.1 BCIN|XP_001559585.1 BGRA|VDB93928.1 CFRU|XP_031884406.1 CGLO|KAF3810341.1 CHIG|XP_018158353.1 CVIN|KAF4895353.1 CVYL|A05765 FGRM|XP_011316489.1 MGRA|XP_003855833.1 MLAR|XP_007417579.1 MORY|QBZ55184.1 NCRA|XP_964380.1 SSCL|APA09569.1

>Orthogroup3010: ANID|CBF82508.1 BCIN|XP_024552845.1 BGRA|VCU39734.1 CFRU|XP_031882400.1 CGLO|KAF3807521.1 CHIG|XP_018156418.1 CVIN|KAF4928929.1 CVYL|A02964 FGRM|XP_011318610.1 MGRA|XP_003853498.1 MLAR|XP_007407978.1 MORY|QBZ63667.1 NCRA|XP_957106.3 SSCL|APA07602.1

>Orthogroup3011: ANID|CBF82510.1 BCIN|XP_024552936.1 BGRA|VCU41276.1 CFRU|XP_031882343.1 CGLO|KAF3807518.1 CHIG|XP_018156421.1 CVIN|KAF4928930.1 CVYL|A02967 FGRM|XP_011318612.1 MGRA|XP_003849635.1 MLAR|XP_007412328.1 MORY|QBZ63670.1 NCRA|XP_957103.1 SSCL|APA07552.1

>Orthogroup3012: ANID|CBF82541.1 BCIN|XP_001551266.1 BGRA|VCU39742.1 CFRU|XP_031882481.1 CGLO|KAF3807573.1 CHIG|XP_018156361.1 CVIN|KAF4928957.1 CVYL|A02909 FGRM|XP_011318945.1 MGRA|XP_003856002.1 MLAR|XP_007411647.1 MORY|QBZ63102.1 NCRA|XP_960789.2 SSCL|APA07546.1

>Orthogroup3014: ANID|CBF82545.1 BCIN|XP_001552848.2 BGRA|VCU41373.1 CFRU|XP_031888442.1 CGLO|KAF3797800.1 CHIG|XP_018156475.1 CVIN|KAF4923157.1 CVYL|A03028 FGRM|XP_011318354.1 MGRA|XP_003853517.1 MLAR|XP_007403709.1 MORY|QBZ57797.1 NCRA|XP_959841.3 SSCL|APA07621.1

>Orthogroup3015: ANID|CBF82549.1 BCIN|XP_001551280.1 BGRA|VCU41372.1 CFRU|XP_031887331.1 CGLO|KAF3809393.1 CHIG|XP_018156234.1 CVIN|KAF4916414.1 CVYL|A02941 FGRM|XP_011319128.1 MGRA|XP_003853511.1 MLAR|XP_007410945.1 MORY|QBZ57429.1 NCRA|XP_960821.1 SSCL|APA07559.1

>Orthogroup3016: ANID|CBF82558.1 BCIN|XP_001551271.1 BGRA|VCU41296.1 CFRU|XP_031889080.1 CGLO|KAF3808697.1 CHIG|XP_018155561.1 CVIN|KAF4929577.1 CVYL|A00959 FGRM|XP_011321121.1 MGRA|XP_003854193.1 MLAR|XP_007415877.1 MORY|QBZ60103.1 NCRA|XP_957407.1 SSCL|APA07549.1

>Orthogroup3017: ANID|CBF82562.1 BCIN|XP_024552932.1 BGRA|VCU41279.1 CFRU|XP_031889445.1 CGLO|KAF3808694.1 CHIG|XP_018155557.1 CVIN|KAF4929622.1 CVYL|A00957 FGRM|XP_011321118.1 MGRA|XP_003853548.1 MLAR|XP_007405712.1 MORY|QBZ60102.1 NCRA|XP_957404.2 SSCL|APA07547.1

>Orthogroup3018: ANID|CBF82564.1 BCIN|XP_001550341.1 BGRA|VDB84096.1 CFRU|XP_031890436.1 CGLO|KAF3798140.1 CHIG|XP_018158630.1 CVIN|KAF4926034.1 CVYL|A13965 FGRM|XP_011316599.1 MGRA|XP_003857666.1 MLAR|XP_007416903.1 MORY|QBZ56089.1 NCRA|XP_964103.1 SSCL|APA10043.1

>Orthogroup3021: ANID|CBF82585.1 BCIN|XP_001550336.1 BGRA|VDB84058.1 CFRU|XP_031890621.1 CGLO|KAF3805305.1 CHIG|XP_018158624.1 CVIN|KAF4925977.1 CVYL|A13958 FGRM|XP_011316429.1 MGRA|XP_003855837.1 MLAR|XP_007412641.1 MORY|QBZ62604.1 NCRA|XP_963727.3 SSCL|APA10027.1

>Orthogroup3022: ANID|CBF82593.1 BCIN|XP_001561287.1 BGRA|VDB90512.1 CFRU|XP_031882836.1 CGLO|KAF3803429.1 CHIG|XP_018164116.1 CVIN|KAF4926287.1 CVYL|A14358 FGRM|XP_011326825.1 MGRA|XP_003853519.1 MLAR|XP_007417293.1 MORY|QBZ56420.1 NCRA|XP_963693.1 SSCL|APA06586.1

>Orthogroup3023: ANID|CBF82595.1 BCIN|XP_024549440.1 BGRA|VDB90922.1 CFRU|XP_031880003.1 CGLO|KAF3798996.1 CHIG|XP_018152906.1 CVIN|KAF4923491.1 CVYL|A07915 FGRM|XP_011324333.1 MGRA|XP_003853010.1 MLAR|XP_007410295.1 MORY|QBZ53523.1 NCRA|XP_958105.1 SSCL|APA09214.1

>Orthogroup3026: ANID|CBF82618.1 BCIN|XP_001548040.1 BGRA|VDB89120.1 CFRU|XP_031878379.1 CGLO|KAF3810773.1 CHIG|XP_018162542.1 CVIN|KAF4932059.1 CVYL|A07180 FGRM|XP_011326982.1 MGRA|XP_003855198.1 MLAR|XP_007410120.1 MORY|QBZ61352.1 NCRA|XP_957281.3 SSCL|APA10510.1

>Orthogroup3027: ANID|CBF82624.1 BCIN|XP_001551776.2 BGRA|VDB89840.1 CFRU|XP_031877680.1 CGLO|KAF3797341.1 CHIG|XP_018157169.1 CVIN|KAF4917649.1 CVYL|A10350 FGRM|XP_011319871.1 MGRA|XP_003854915.1 MLAR|XP_007403910.1 MORY|QBZ65965.1 NCRA|XP_965816.2 SSCL|APA11963.1

>Orthogroup3028: ANID|CBF82627.1 BCIN|XP_024547243.1 BGRA|VDB93070.1 CFRU|XP_031877667.1 CGLO|KAF3796985.1 CHIG|XP_018153701.1 CVIN|KAF4898629.1 CVYL|A00235 FGRM|XP_011327823.1 MGRA|XP_003848628.1 MLAR|XP_007414728.1 MORY|QBZ60540.1 NCRA|XP_011395182.1 SSCL|APA14186.1

>Orthogroup3029: ANID|CBF82633.1 BCIN|XP_001560084.2 BGRA|VDB93279.1 CFRU|XP_031890229.1 CGLO|KAF3798878.1 CHIG|XP_018158547.1 CVIN|KAF4925553.1 CVYL|A05075 FGRM|XP_011316626.1 MGRA|XP_003854785.1 MLAR|XP_007407755.1 MORY|QBZ55970.1 NCRA|XP_965007.1 SSCL|APA09900.1

>Orthogroup3030: ANID|CBF82635.1 BCIN|XP_001560085.1 BGRA|VDB93298.1 CFRU|XP_031890032.1 CGLO|KAF3798879.1 CHIG|XP_018158548.1 CVIN|KAF4925573.1 CVYL|A05074 FGRM|XP_011316625.1 MGRA|XP_003855742.1 MLAR|XP_007403890.1 MORY|QBZ55971.1 NCRA|XP_965008.1 SSCL|APA09899.1

>Orthogroup3031: ANID|CBF82655.1 BCIN|XP_001550570.1 BGRA|VDB89068.1 CFRU|XP_031877634.1 CGLO|KAF3801065.1 CHIG|XP_018153711.1 CVIN|KAF4902053.1 CVYL|A00135 FGRM|XP_011327834.1 MGRA|XP_003848656.1 MLAR|XP_007412816.1 MORY|QBZ60563.1 NCRA|XP_959606.1 SSCL|APA14166.1

>Orthogroup3033: ANID|CBF82659.1 BCIN|XP_024547351.1 BGRA|VDB88912.1 CFRU|XP_031891838.1 CGLO|KAF3800157.1 CHIG|XP_018165089.1 CVIN|KAF4925834.1 CVYL|A06851 FGRM|XP_011327706.1 MGRA|XP_003848600.1 MLAR|XP_007411404.1 MORY|QBZ60658.1 NCRA|XP_956064.3 SSCL|APA14349.1

>Orthogroup3034: ANID|CBF82660.1 BCIN|XP_001555974.1 BGRA|VDB88898.1 CFRU|XP_031879699.1 CGLO|KAF3798221.1 CHIG|XP_018153938.1 CVIN|KAF4916823.1 CVYL|A04900 FGRM|XP_011325611.1 MGRA|XP_003848580.1 MLAR|XP_007404630.1 MORY|QBZ60772.1 NCRA|XP_960011.3 SSCL|APA13912.1

>Orthogroup3036: ANID|CBF82674.1 BCIN|XP_024547237.1 BGRA|VDB89087.1 CFRU|XP_031881992.1 CGLO|KAF3806148.1 CHIG|XP_018164514.1 CVIN|KAF4907015.1 CVYL|A07359 FGRM|XP_011325023.1 MGRA|XP_003848586.1 MLAR|XP_007411640.1 MORY|QBZ60621.1 NCRA|XP_001728360.2 SSCL|APA14182.1

>Orthogroup3037: ANID|CBF82677.1 BCIN|XP_024547200.1 BGRA|VCU40073.1 CFRU|XP_031882025.1 CGLO|KAF3806037.1 CHIG|XP_018164631.1 CVIN|KAF4917848.1 CVYL|A07470 FGRM|XP_011326733.1 MGRA|XP_003848436.1 MLAR|XP_007413800.1 MORY|QBZ62361.1 NCRA|XP_011395314.1 SSCL|APA14129.1

>Orthogroup3038: ANID|CBF82683.1 BCIN|XP_024547255.1 BGRA|VDB94020.1 CFRU|XP_031877662.1 CGLO|KAF3801091.1 CHIG|XP_018153742.1 CVIN|KAF4909537.1 CVYL|A11304 FGRM|XP_011327811.1 MGRA|XP_003848430.1 MLAR|XP_007406997.1 MORY|QBZ60600.1 NCRA|XP_011395147.1 SSCL|APA14205.1

>Orthogroup3040: ANID|CBF82694.1 BCIN|XP_001552794.1 BGRA|VDB88845.1 CFRU|XP_031892021.1 CGLO|KAF3800140.1 CHIG|XP_018165075.1 CVIN|KAF4925841.1 CVYL|A06830 FGRM|XP_011327684.1 MGRA|XP_003851908.1 MLAR|XP_007407819.1 MORY|QBZ54030.1 NCRA|XP_961195.2 SSCL|APA14035.1

>Orthogroup3041: ANID|CBF82698.1 BCIN|XP_001547853.2 BGRA|VCU40884.1 CFRU|XP_031887231.1 CGLO|KAF3809303.1 CHIG|XP_018156136.1 CVIN|KAF4911372.1 CVYL|A01499 FGRM|XP_011318823.1 MGRA|XP_003857513.1 MLAR|XP_007410964.1 MORY|QBZ62228.1 NCRA|XP_957594.3 SSCL|APA11543.1

>Orthogroup3042: ANID|CBF82707.1 BCIN|XP_001554359.1 BGRA|VDB83760.1 CFRU|XP_031881931.1 CGLO|KAF3806143.1 CHIG|XP_018164518.1 CVIN|KAF4907022.1 CVYL|A07363 FGRM|XP_011325001.1 MGRA|XP_003848691.1 MLAR|XP_007411344.1 MORY|QBZ62354.1 NCRA|XP_959963.1 SSCL|APA14433.1

>Orthogroup3043: ANID|CBF82711.1 BCIN|XP_024547282.1 BGRA|VCU40078.1 CFRU|XP_031891781.1 CGLO|KAF3801394.1 CHIG|XP_018164776.1 CVIN|KAF4907812.1 CVYL|A07690 FGRM|XP_011326674.1 MGRA|XP_003848529.1 MLAR|XP_007407204.1 MORY|QBZ62265.1 NCRA|XP_959469.1 SSCL|APA14242.1

>Orthogroup3044: ANID|CBF82714.1 BCIN|XP_024547290.1 BGRA|VCU40203.1 CFRU|XP_031877638.1 CGLO|KAF3801088.1 CHIG|XP_018153739.1 CVIN|KAF4890683.1 CVYL|A11307 FGRM|XP_011327783.1 MGRA|XP_003848281.1 MLAR|XP_007404547.1 MORY|QBZ60594.1 NCRA|XP_011395151.1 SSCL|APA14249.1

>Orthogroup3045: ANID|CBF82722.1 BCIN|XP_024551048.1 BGRA|VDB88094.1 CFRU|XP_031881591.1 CGLO|KAF3807384.1 CHIG|XP_018156841.1 CVIN|KAF4929605.1 CVYL|A10877 FGRM|XP_011328165.1 MGRA|XP_003848514.1 MLAR|XP_007415825.1 MORY|QBZ65658.1 NCRA|XP_011393056.1 SSCL|APA11979.1

>Orthogroup3046: ANID|CBF82731.1 BCIN|XP_024550689.1 BGRA|VDB93214.1 CFRU|XP_031880241.1 CGLO|KAF3808960.1 CHIG|XP_018155813.1 CVIN|KAF4920725.1 CVYL|A01125 FGRM|XP_011319096.1 MGRA|XP_003856180.1 MLAR|XP_007405611.1 MORY|QBZ62200.1 NCRA|XP_961063.1 SSCL|APA11514.1

>Orthogroup3047: ANID|CBF82740.1 BCIN|XP_001557711.1 BGRA|VCU40938.1 CFRU|XP_031892572.1 CGLO|KAF3809156.1 CHIG|XP_018155976.1 CVIN|KAF4919820.1 CVYL|A01342 FGRM|XP_011318810.1 MGRA|XP_003856060.1 MLAR|XP_007417057.1 MORY|QBZ62424.1 NCRA|XP_958854.1 SSCL|APA12219.1

>Orthogroup3050: ANID|CBF82760.1 BCIN|XP_001560976.1 BGRA|VCU39925.1 CFRU|XP_031889606.1 CGLO|KAF3805214.1 CHIG|XP_018158855.1 CVIN|KAF4907187.1 CVYL|A13862 FGRM|XP_011316438.1 MGRA|XP_003852059.1 MLAR|XP_007411600.1 MORY|QBZ62490.1 NCRA|XP_963854.2 SSCL|APA06706.1

>Orthogroup3051: ANID|CBF82844.1 BCIN|XP_024546537.1 BGRA|VCU39927.1 CFRU|XP_031889847.1 CGLO|KAF3810546.1 CHIG|XP_018159050.1 CVIN|KAF4895565.1 CVYL|A13622 FGRM|XP_011328360.1 MGRA|XP_003852161.1 MLAR|XP_007415455.1 MORY|QBZ54528.1 NCRA|XP_959820.1 SSCL|APA06712.1

>Orthogroup3052: ANID|CBF82862.1 BCIN|XP_024545982.1 BGRA|VCU39846.1 CFRU|XP_031890185.1 CGLO|KAF3802868.1 CHIG|XP_018158466.1 CVIN|KAF4923830.1 CVYL|A05145 FGRM|XP_011316411.1 MGRA|XP_003857449.1 MLAR|XP_007407545.1 MORY|QBZ55902.1 NCRA|XP_964754.2 SSCL|APA07428.1

>Orthogroup3053: ANID|CBF82976.1 BCIN|XP_001554582.2 BGRA|VDB90747.1 CFRU|XP_031892313.1 CGLO|KAF3809129.1 CHIG|XP_018155965.1 CVIN|KAF4922124.1 CVYL|A01312 FGRM|XP_011323532.1 MGRA|XP_003851714.1 MLAR|XP_007411929.1 MORY|QBZ63101.1 NCRA|XP_960790.1 SSCL|APA11646.1

>Orthogroup3057: ANID|CBF83232.1 BCIN|XP_024553066.1 BGRA|VCU41379.1 CFRU|XP_031882374.1 CGLO|KAF3807566.1 CHIG|XP_018156368.1 CVIN|KAF4928972.1 CVYL|A02916 FGRM|XP_011318930.1 MGRA|XP_003856311.1 MLAR|XP_007405375.1 MORY|QBZ57111.1 NCRA|XP_961616.1 SSCL|APA16270.1

>Orthogroup3058: ANID|CBF83255.1 BCIN|XP_001559658.1 BGRA|VDB89376.1 CFRU|XP_031885884.1 CGLO|KAF3805443.1 CHIG|XP_018155312.1 CVIN|KAF4930396.1 CVYL|A05573 FGRM|XP_011325484.1 MGRA|XP_003856031.1 MLAR|XP_007409892.1 MORY|QBZ53796.1 NCRA|XP_956827.1 SSCL|APA09420.1

>Orthogroup3059: ANID|CBF83264.1 BCIN|XP_001559648.1 BGRA|VDB89381.1 CFRU|XP_031885940.1 CGLO|KAF3805455.1 CHIG|XP_018155317.1 CVIN|KAF4930451.1 CVYL|A05585 FGRM|XP_011325475.1 MGRA|XP_003856080.1 MLAR|XP_007405926.1 MORY|QBZ54417.1 NCRA|XP_956840.1 SSCL|APA09412.1

>Orthogroup3060: ANID|CBF83268.1 BCIN|XP_001559672.1 BGRA|VDB89402.1 CFRU|XP_031878016.1 CGLO|KAF3799579.1 CHIG|XP_018154552.1 CVIN|KAF4926505.1 CVYL|A13439 FGRM|XP_011325556.1 MGRA|XP_003856176.1 MLAR|XP_007406614.1 MORY|QBZ53980.1 NCRA|XP_011393507.1 SSCL|APA09425.1

>Orthogroup3061: ANID|CBF83271.1 BCIN|XP_001559650.1 BGRA|VDB89379.1 CFRU|XP_031885942.1 CGLO|KAF3805457.1 CHIG|XP_018155319.1 CVIN|KAF4930452.1 CVYL|A05587 FGRM|XP_011325473.1 MGRA|XP_003857631.1 MLAR|XP_007404053.1 MORY|QBZ54419.1 NCRA|XP_956842.1 SSCL|APA09414.1

>Orthogroup3063: ANID|CBF83293.1 BCIN|XP_001547239.1 BGRA|VCU38932.1 CFRU|XP_031887516.1 CGLO|KAF3811605.1 CHIG|XP_018160877.1 CVIN|KAF4921450.1 CVYL|A03462 FGRM|XP_011324865.1 MGRA|XP_003848775.1 MLAR|XP_007404530.1 MORY|QBZ58884.1 NCRA|XP_961905.1 SSCL|APA07375.1

>Orthogroup3064: ANID|CBF83297.1 BCIN|XP_001547564.1 BGRA|VDB91217.1 CFRU|XP_031889166.1 CGLO|KAF3803140.1 CHIG|XP_018155535.1 CVIN|KAF4928268.1 CVYL|A01027 FGRM|XP_011319354.1 MGRA|XP_003857416.1 MLAR|XP_007417740.1 MORY|QBZ60175.1 NCRA|XP_955821.1 SSCL|APA07474.1

>Orthogroup3065: ANID|CBF83299.1 BCIN|XP_001547587.1 BGRA|VCU41309.1 CFRU|XP_031889110.1 CGLO|KAF3803086.1 CHIG|XP_018155583.1 CVIN|KAF4929553.1 CVYL|A00978 FGRM|XP_011321130.1 MGRA|XP_003856266.1 MLAR|XP_007417758.1 MORY|QBZ59251.1 NCRA|XP_957431.2 SSCL|APA07456.1

>Orthogroup3066: ANID|CBF83315.1 BCIN|XP_024552001.1 BGRA|VDB89694.1 CFRU|XP_031880629.1 CGLO|KAF3809946.1 CHIG|XP_018161526.1 CVIN|KAF4930663.1 CVYL|A01677 FGRM|XP_011318384.1 MGRA|XP_003848945.1 MLAR|XP_007408775.1 MORY|QBZ65121.1 NCRA|XP_958407.1 SSCL|APA12734.1

>Orthogroup3067: ANID|CBF83342.1 BCIN|XP_024548716.1 BGRA|VDB89325.1 CFRU|XP_031877784.1 CGLO|KAF3799403.1 CHIG|XP_018152263.1 CVIN|KAF4931829.1 CVYL|A13267 FGRM|XP_011324600.1 MGRA|XP_003852809.1 MLAR|XP_007409035.1 MORY|QBZ53936.1 NCRA|XP_956627.2 SSCL|APA09990.1

>Orthogroup3069: ANID|CBF83347.1 BCIN|XP_024551625.1 BGRA|VDB89562.1 CFRU|XP_031884765.1 CGLO|KAF3812102.1 CHIG|XP_018153340.1 CVIN|KAF4918127.1 CVYL|A00295 FGRM|XP_011318736.1 MGRA|XP_003855786.1 MLAR|XP_007413072.1 MORY|QBZ55245.1 NCRA|XP_965331.1 SSCL|APA06269.1

>Orthogroup3070: ANID|CBF83359.1 BCIN|XP_001548304.2 BGRA|VCU39981.1 CFRU|XP_031883846.1 CGLO|KAF3805157.1 CHIG|XP_018163356.1 CVIN|KAF4927418.1 CVYL|A06593 FGRM|XP_011321658.1 MGRA|XP_003854714.1 MLAR|XP_007405035.1 MORY|QBZ63266.1 NCRA|XP_956930.1 SSCL|APA06379.1

>Orthogroup3072: ANID|CBF83367.1 BCIN|XP_024551922.1 BGRA|VCU39978.1 CFRU|XP_031883896.1 CGLO|KAF3805136.1 CHIG|XP_018163378.1 CVIN|KAF4927460.1 CVYL|A06420 FGRM|XP_011321623.1 MGRA|XP_003855748.1 MLAR|XP_007406278.1 MORY|QBZ63274.1 NCRA|XP_956922.1 SSCL|APA06358.1

>Orthogroup3074: ANID|CBF83378.1 BCIN|XP_001556701.1 BGRA|VDB85886.1 CFRU|XP_031883963.1 CGLO|KAF3805160.1 CHIG|XP_018163352.1 CVIN|KAF4927493.1 CVYL|A06590 FGRM|XP_011321652.1 MGRA|XP_003855540.1 MLAR|XP_007415490.1 MORY|QBZ63262.1 NCRA|XP_956934.1 SSCL|APA06005.1

>Orthogroup3076: ANID|CBF83412.1 BCIN|XP_024548877.1 BGRA|VDB89395.1 CFRU|XP_031885937.1 CGLO|KAF3805453.1 CHIG|XP_018155301.1 CVIN|KAF4930376.1 CVYL|A05583 FGRM|XP_011325477.1 MGRA|XP_003851691.1 MLAR|XP_007407435.1 MORY|QBZ53797.1 NCRA|XP_956834.1 SSCL|APA09424.1

>Orthogroup3077: ANID|CBF83414.1 BCIN|XP_024548878.1 BGRA|VDB89394.1 CFRU|XP_031885936.1 CGLO|KAF3805452.1 CHIG|XP_018155302.1 CVIN|KAF4930377.1 CVYL|A05582 FGRM|XP_011325478.1 MGRA|XP_003851874.1 MLAR|XP_007413785.1 MORY|QBZ53790.1 NCRA|XP_956833.3 SSCL|APA09423.1

>Orthogroup3078: ANID|CBF83421.1 BCIN|XP_001552033.1 BGRA|VDB89407.1 CFRU|XP_031882702.1 CGLO|KAF3799399.1 CHIG|XP_018152249.1 CVIN|KAF4931826.1 CVYL|A13263 FGRM|XP_011324610.1 MGRA|XP_003851400.1 MLAR|XP_007406269.1 MORY|QBZ54010.1 NCRA|XP_956495.1 SSCL|APA10015.1

>Orthogroup3079: ANID|CBF83426.1 BCIN|XP_024552855.1 BGRA|VCU39674.1 CFRU|XP_031892510.1 CGLO|KAF3809094.1 CHIG|XP_018155894.1 CVIN|KAF4900562.1 CVYL|A01281 FGRM|XP_011318900.1 MGRA|XP_003856727.1 MLAR|XP_007407434.1 MORY|QBZ64501.1 NCRA|XP_011394709.1 SSCL|APA16130.1

>Orthogroup3081: ANID|CBF83444.1 BCIN|XP_024547128.1 BGRA|VDB92978.1 CFRU|XP_031879650.1 CGLO|KAF3798235.1 CHIG|XP_018154013.1 CVIN|KAF4930123.1 CVYL|A04888 FGRM|XP_011325601.1 MGRA|XP_003851654.1 MLAR|XP_007415593.1 MORY|QBZ61205.1 NCRA|XP_958077.1 SSCL|APA14027.1

>Orthogroup3082: ANID|CBF83464.1 BCIN|XP_024547354.1 BGRA|VDB88972.1 CFRU|XP_031877308.1 CGLO|KAF3799874.1 CHIG|XP_018153819.1 CVIN|KAF4921727.1 CVYL|A09644 FGRM|XP_011325435.1 MGRA|XP_003848426.1 MLAR|XP_007408019.1 MORY|QBZ59956.1 NCRA|XP_958049.2 SSCL|APA14356.1

>Orthogroup3083: ANID|CBF83469.1 BCIN|XP_001551077.2 BGRA|VCU40157.1 CFRU|XP_031891815.1 CGLO|KAF3805564.1 CHIG|XP_018164737.1 CVIN|KAF4922383.1 CVYL|A07639 FGRM|XP_011324687.1 MGRA|XP_003848653.1 MLAR|XP_007415863.1 MORY|QBZ64923.1 NCRA|XP_955939.3 SSCL|APA14263.1

>Orthogroup3085: ANID|CBF83482.1 BCIN|XP_024547352.1 BGRA|VDB88968.1 CFRU|XP_031877310.1 CGLO|KAF3799876.1 CHIG|XP_018153817.1 CVIN|KAF4921729.1 CVYL|A09642 FGRM|XP_011325409.1 MGRA|XP_003848276.1 MLAR|XP_007418583.1 MORY|QBZ59977.1 NCRA|XP_958047.1 SSCL|APA14354.1

>Orthogroup3086: ANID|CBF83517.1 BCIN|XP_024553187.1 BGRA|VDB89036.1 CFRU|XP_031882108.1 CGLO|KAF3806125.1 CHIG|XP_018164538.1 CVIN|KAF4911039.1 CVYL|A07384 FGRM|XP_011325639.1 MGRA|XP_003851798.1 MLAR|XP_007410958.1 MORY|QBZ62313.1 NCRA|XP_959500.2 SSCL|APA13450.1

>Orthogroup3087: ANID|CBF83537.1 BCIN|XP_024547337.1 BGRA|VDB89013.1 CFRU|XP_031892259.1 CGLO|KAF3805577.1 CHIG|XP_018164725.1 CVIN|KAF4922353.1 CVYL|A07625 FGRM|XP_011324971.1 MGRA|XP_003851846.1 MLAR|XP_007411348.1 MORY|QBZ60857.1 NCRA|XP_958960.1 SSCL|APA14336.1

>Orthogroup3089: ANID|CBF83562.1 BCIN|XP_024547340.1 BGRA|VDB89015.1 CFRU|XP_031891662.1 CGLO|KAF3805594.1 CHIG|XP_018164722.1 CVIN|KAF4900694.1 CVYL|A07604 FGRM|XP_011324974.1 MGRA|XP_003848499.1 MLAR|XP_007417932.1 MORY|QBZ60937.1 NCRA|XP_959546.2 SSCL|APA14333.1

>Orthogroup3090: ANID|CBF83564.1 BCIN|XP_001546309.1 BGRA|VDB89026.1 CFRU|XP_031891668.1 CGLO|KAF3805595.1 CHIG|XP_018164720.1 CVIN|KAF4900689.1 CVYL|A07602 FGRM|XP_011324976.1 MGRA|XP_003848320.1 MLAR|XP_007411188.1 MORY|QBZ60934.1 NCRA|XP_958590.1 SSCL|APA14318.1

>Orthogroup3091: ANID|CBF83566.1 BCIN|XP_024547332.1 BGRA|VDB89027.1 CFRU|XP_031891671.1 CGLO|KAF3805597.1 CHIG|XP_018164717.1 CVIN|KAF4889191.1 CVYL|A07599 FGRM|XP_011324980.1 MGRA|XP_003848325.1 MLAR|XP_007403627.1 MORY|QBZ62324.1 NCRA|XP_959646.1 SSCL|APA14319.1

>Orthogroup3092: ANID|CBF83571.1 BCIN|XP_001554275.1 BGRA|VDB88910.1 CFRU|XP_031882073.1 CGLO|KAF3806003.1 CHIG|XP_018164650.1 CVIN|KAF4925706.1 CVYL|A07511 FGRM|XP_011324995.1 MGRA|XP_003848513.1 MLAR|XP_007413871.1 MORY|QBZ62327.1 NCRA|XP_959644.1 SSCL|APA14375.1

>Orthogroup3093: ANID|CBF83584.1 BCIN|XP_001556000.1 BGRA|VDB83784.1 CFRU|XP_031877312.1 CGLO|KAF3799878.1 CHIG|XP_018153815.1 CVIN|KAF4921731.1 CVYL|A09640 FGRM|XP_011325411.1 MGRA|XP_003848536.1 MLAR|XP_007417873.1 MORY|QBZ59978.1 NCRA|XP_958045.3 SSCL|APA13929.1

>Orthogroup3094: ANID|CBF83593.1 BCIN|XP_024550688.1 BGRA|VDB93216.1 CFRU|XP_031887411.1 CGLO|KAF3809265.1 CHIG|XP_018156100.1 CVIN|KAF4907385.1 CVYL|A01458 FGRM|XP_011319313.1 MGRA|XP_003856037.1 MLAR|XP_007403525.1 MORY|QBZ57395.1 NCRA|XP_011394873.1 SSCL|APA11512.1

>Orthogroup3095: ANID|CBF83595.1 BCIN|XP_001557201.2 BGRA|VCU40943.1 CFRU|XP_031882360.1 CGLO|KAF3807582.1 CHIG|XP_018156352.1 CVIN|KAF4928900.1 CVYL|A02900 FGRM|XP_011319220.1 MGRA|XP_003857701.1 MLAR|XP_007405720.1 MORY|QBZ62166.1 NCRA|XP_011394878.1 SSCL|APA12494.1

>Orthogroup3096: ANID|CBF83598.1 BCIN|XP_001552589.1 BGRA|VCU39549.1 CFRU|XP_031887281.1 CGLO|KAF3809202.1 CHIG|XP_018156036.1 CVIN|KAF4925345.1 CVYL|A01392 FGRM|XP_011319121.1 MGRA|XP_003856138.1 MLAR|XP_007408301.1 MORY|QBZ57072.1 NCRA|XP_960690.1 SSCL|APA11441.1

>Orthogroup3098: ANID|CBF83607.1 BCIN|XP_024552988.1 BGRA|VCU41179.1 CFRU|XP_031892556.1 CGLO|KAF3809141.1 CHIG|XP_018155954.1 CVIN|KAF4922105.1 CVYL|A01326 FGRM|XP_011318878.1 MGRA|XP_003856120.1 MLAR|XP_007408568.1 MORY|QBZ62438.1 NCRA|XP_011394818.1 SSCL|APA16218.1

>Orthogroup3099: ANID|CBF83612.1 BCIN|XP_024549245.1 BGRA|VDB90736.1 CFRU|XP_031892688.1 CGLO|KAF3803074.1 CHIG|XP_018153523.1 CVIN|KAF4919844.1 CVYL|A00086 FGRM|XP_011316112.1 MGRA|XP_003853258.1 MLAR|XP_007404177.1 MORY|QBZ55587.1 NCRA|XP_965122.2 SSCL|APA09017.1

>Orthogroup3100: ANID|CBF83616.1 BCIN|XP_024549244.1 BGRA|VDB90735.1 CFRU|XP_031892687.1 CGLO|KAF3803073.1 CHIG|XP_018153524.1 CVIN|KAF4919873.1 CVYL|A00085 FGRM|XP_011316113.1 MGRA|XP_003853142.1 MLAR|XP_007412286.1 MORY|QBZ55588.1 NCRA|XP_011393017.1 SSCL|APA09018.1

>Orthogroup3101: ANID|CBF83623.1 BCIN|XP_024549883.1 BGRA|VDB91116.1 CFRU|XP_031890540.1 CGLO|KAF3804892.1 CHIG|XP_018164370.1 CVIN|KAF4921178.1 CVYL|A12169 FGRM|XP_011316799.1 MGRA|XP_003850504.1 MLAR|XP_007405626.1 MORY|QBZ55317.1 NCRA|XP_964025.3 SSCL|APA05560.1

>Orthogroup3102: ANID|CBF83633.1 BCIN|XP_001556189.1 BGRA|VDB91153.1 CFRU|XP_031888229.1 CGLO|KAF3803645.1 CHIG|XP_018163809.1 CVIN|KAF4913950.1 CVYL|A14579 FGRM|XP_011317004.1 MGRA|XP_003853175.1 MLAR|XP_007406668.1 MORY|QBZ54671.1 NCRA|XP_963917.1 SSCL|APA09025.1

>Orthogroup3103: ANID|CBF83644.1 BCIN|XP_024551160.1 BGRA|VDB89158.1 CFRU|XP_031886748.1 CGLO|KAF3804091.1 CHIG|XP_018162677.1 CVIN|KAF4919520.1 CVYL|A07051 FGRM|XP_011326355.1 MGRA|XP_003856195.1 MLAR|XP_007406410.1 MORY|QBZ55634.1 NCRA|XP_957212.1 SSCL|APA10345.1

>Orthogroup3104: ANID|CBF83646.1 BCIN|XP_024546310.1 BGRA|VDB90758.1 CFRU|XP_031888455.1 CGLO|KAF3803487.1 CHIG|XP_018164017.1 CVIN|KAF4926308.1 CVYL|A14416 FGRM|XP_011316982.1 MGRA|XP_003853207.1 MLAR|XP_007406890.1 MORY|QBZ55337.1 NCRA|XP_965460.1 SSCL|APA06916.1

>Orthogroup3106: ANID|CBF83655.1 BCIN|XP_024551501.1 BGRA|VCU39799.1 CFRU|XP_031893336.1 CGLO|KAF3808024.1 CHIG|XP_018152356.1 CVIN|KAF4909489.1 CVYL|A09459 FGRM|XP_011317757.1 MGRA|XP_003856194.1 MLAR|XP_007404506.1 MORY|QBZ54217.1 NCRA|XP_960747.2 SSCL|APA10349.1

>Orthogroup3108: ANID|CBF83689.1 BCIN|XP_024553460.1 BGRA|VDB93522.1 CFRU|XP_031888530.1 CGLO|KAF3803575.1 CHIG|XP_018163919.1 CVIN|KAF4922226.1 CVYL|A14509 FGRM|XP_011316746.1 MGRA|XP_003857340.1 MLAR|XP_007404184.1 MORY|QBZ56027.1 NCRA|XP_963864.3 SSCL|APA13830.1

>Orthogroup3109: ANID|CBF83695.1 BCIN|XP_001547950.1 BGRA|VDB93716.1 CFRU|XP_031884318.1 CGLO|KAF3803764.1 CHIG|XP_018153160.1 CVIN|KAF4917893.1 CVYL|A14144 FGRM|XP_011324183.1 MGRA|XP_003856592.1 MLAR|XP_007415555.1 MORY|QBZ59780.1 NCRA|XP_965646.2 SSCL|APA13602.1

>Orthogroup3110: ANID|CBF83699.1 BCIN|XP_024547019.1 BGRA|VDB93747.1 CFRU|XP_031877851.1 CGLO|KAF3804774.1 CHIG|XP_018164252.1 CVIN|KAF4929801.1 CVYL|A12303 FGRM|XP_011317033.1 MGRA|XP_003857178.1 MLAR|XP_007415822.1 MORY|QBZ63568.1 NCRA|XP_963953.2 SSCL|APA05894.1

>Orthogroup3111: ANID|CBF83701.1 BCIN|XP_001548808.1 BGRA|VDB93641.1 CFRU|XP_031879866.1 CGLO|KAF3802972.1 CHIG|XP_018153641.1 CVIN|KAF4929910.1 CVYL|A00405 FGRM|XP_011316892.1 MGRA|XP_003856320.1 MLAR|XP_007412385.1 MORY|QBZ63538.1 NCRA|XP_963824.1 SSCL|APA13338.1

>Orthogroup3112: ANID|CBF83711.1 BCIN|XP_001559942.1 BGRA|VDB93271.1 CFRU|XP_031886645.1 CGLO|KAF3807477.1 CHIG|XP_018158760.1 CVIN|KAF4907605.1 CVYL|A05000 FGRM|XP_011328627.1 MGRA|XP_003852324.1 MLAR|XP_007416506.1 MORY|QBZ60417.1 NCRA|XP_965317.1 SSCL|APA09767.1

>Orthogroup3113: ANID|CBF83718.1 BCIN|XP_001561383.1 BGRA|VDB90699.1 CFRU|XP_031882920.1 CGLO|KAF3803403.1 CHIG|XP_018163929.1 CVIN|KAF4918430.1 CVYL|A14333 FGRM|XP_011316857.1 MGRA|XP_003852218.1 MLAR|XP_007412762.1 MORY|QBZ62749.1 NCRA|XP_964215.2 SSCL|APA06895.1

>Orthogroup3114: ANID|CBF83720.1 BCIN|XP_024546322.1 BGRA|VDB90731.1 CFRU|XP_031882909.1 CGLO|KAF3812076.1 CHIG|XP_018164088.1 CVIN|KAF4923675.1 CVYL|A14252 FGRM|XP_011326756.1 MGRA|XP_003853128.1 MLAR|XP_007404385.1 MORY|QBZ62745.1 NCRA|XP_011393134.1 SSCL|APA06901.1

>Orthogroup3115: ANID|CBF83738.1 BCIN|XP_024547043.1 BGRA|VDB93821.1 CFRU|XP_031890679.1 CGLO|KAF3804833.1 CHIG|XP_018164330.1 CVIN|KAF4929786.1 CVYL|A12240 FGRM|XP_011324155.1 MGRA|XP_003857330.1 MLAR|XP_007411355.1 MORY|QBZ62769.1 NCRA|XP_965276.1 SSCL|APA06112.1

>Orthogroup3120: ANID|CBF83765.1 BCIN|XP_024550682.1 BGRA|VDB93207.1 CFRU|XP_031887146.1 CGLO|KAF3809208.1 CHIG|XP_018156031.1 CVIN|KAF4925320.1 CVYL|A01397 FGRM|XP_011319081.1 MGRA|XP_003856071.1 MLAR|XP_007409853.1 MORY|QBZ57086.1 NCRA|XP_011394513.1 SSCL|APA11504.1

>Orthogroup3121: ANID|CBF83769.1 BCIN|XP_001552593.1 BGRA|VCU39552.1 CFRU|XP_031887212.1 CGLO|KAF3809349.1 CHIG|XP_018156201.1 CVIN|KAF4924188.1 CVYL|A01554 FGRM|XP_011318797.1 MGRA|XP_003853017.1 MLAR|XP_007411786.1 MORY|QBZ59037.1 NCRA|XP_960897.1 SSCL|APA11445.1

>Orthogroup3122: ANID|CBF83776.1 BCIN|XP_024547342.1 BGRA|VDB89004.1 CFRU|XP_031878490.1 CGLO|KAF3798601.1 CHIG|XP_018153928.1 CVIN|KAF4930153.1 CVYL|A04845 FGRM|XP_011324694.1 MGRA|XP_003848347.1 MLAR|XP_007410940.1 MORY|QBZ60860.1 NCRA|XP_959882.1 SSCL|APA14327.1

>Orthogroup3123: ANID|CBF83778.1 BCIN|XP_024547369.1 BGRA|VDB88904.1 CFRU|XP_031891666.1 CGLO|KAF3805600.1 CHIG|XP_018164707.1 CVIN|KAF4890023.1 CVYL|A07594 FGRM|XP_011325634.1 MGRA|XP_003848327.1 MLAR|XP_007416315.1 MORY|QBZ62291.1 NCRA|XP_959904.1 SSCL|APA14371.1

>Orthogroup3124: ANID|CBF83780.1 BCIN|XP_001554261.1 BGRA|VDB93995.1 CFRU|XP_031882096.1 CGLO|KAF3806135.1 CHIG|XP_018164528.1 CVIN|KAF4911022.1 CVYL|A07372 FGRM|XP_011325620.1 MGRA|XP_003851813.1 MLAR|XP_007407559.1 MORY|QBZ62275.1 NCRA|XP_959917.1 SSCL|APA14361.1

>Orthogroup3126: ANID|CBF83808.1 BCIN|XP_024551855.1 BGRA|VDB95084.1 CFRU|XP_031884910.1 CGLO|KAF3812200.1 CHIG|XP_018153427.1 CVIN|KAF4920952.1 CVYL|A00185 FGRM|XP_011316044.1 MGRA|XP_003854722.1 MLAR|XP_007415817.1 MORY|QBZ63514.1 NCRA|XP_957916.2 SSCL|APA06297.1

>Orthogroup3127: ANID|CBF83817.1 BCIN|XP_001552410.1 BGRA|VDB89696.1 CFRU|XP_031890962.1 CGLO|KAF3801677.1 CHIG|XP_018151455.1 CVIN|KAF4897547.1 CVYL|A12460 FGRM|XP_011328481.1 MGRA|XP_003854954.1 MLAR|XP_007404217.1 MORY|QBZ62160.1 NCRA|XP_957574.1 SSCL|APA14896.1

>Orthogroup3129: ANID|CBF83826.1 BCIN|XP_001556817.1 BGRA|VDB89265.1 CFRU|XP_031884901.1 CGLO|KAF3812105.1 CHIG|XP_018153342.1 CVIN|KAF4918132.1 CVYL|A00292 FGRM|XP_011318738.1 MGRA|XP_003854712.1 MLAR|XP_007411408.1 MORY|QBZ63848.1 NCRA|XP_957875.2 SSCL|APA14598.1

>Orthogroup3130: ANID|CBF83829.1 BCIN|XP_001556818.1 BGRA|VDB89263.1 CFRU|XP_031883263.1 CGLO|KAF3801733.1 CHIG|XP_018159248.1 CVIN|KAF4922913.1 CVYL|A12968 FGRM|XP_011318737.1 MGRA|XP_003855623.1 MLAR|XP_007403991.1 MORY|QBZ63129.1 NCRA|XP_011394729.1 SSCL|APA14597.1

>Orthogroup3131: ANID|CBF83833.1 BCIN|XP_001559795.1 BGRA|VCU40892.1 CFRU|XP_031890431.1 CGLO|KAF3798137.1 CHIG|XP_018158633.1 CVIN|KAF4926029.1 CVYL|A13969 FGRM|XP_011325453.1 MGRA|XP_003851121.1 MLAR|XP_007415346.1 MORY|QBZ56136.1 NCRA|XP_964977.1 SSCL|APA09668.1

>Orthogroup3132: ANID|CBF83844.1 BCIN|XP_024551701.1 BGRA|VDB89505.1 CFRU|XP_031884714.1 CGLO|KAF3812205.1 CHIG|XP_018153433.1 CVIN|KAF4920947.1 CVYL|A00180 FGRM|XP_011316050.1 MGRA|XP_003853613.1 MLAR|XP_007417005.1 MORY|QBZ63526.1 NCRA|XP_011393370.1 SSCL|APA14537.1

>Orthogroup3133: ANID|CBF83845.1 BCIN|XP_001556741.2 BGRA|VDB89506.1 CFRU|XP_031884920.1 CGLO|KAF3812204.1 CHIG|XP_018153432.1 CVIN|KAF4920948.1 CVYL|A00181 FGRM|XP_011316049.1 MGRA|XP_003855794.1 MLAR|XP_007406329.1 MORY|QBZ63525.1 NCRA|XP_963704.1 SSCL|APA14536.1

>Orthogroup3135: ANID|CBF83856.1 BCIN|XP_001557400.1 BGRA|VCU41046.1 CFRU|XP_031883200.1 CGLO|KAF3808152.1 CHIG|XP_018163254.1 CVIN|KAF4924305.1 CVYL|A00503 FGRM|XP_011326234.1 MGRA|XP_003852151.1 MLAR|XP_007403631.1 MORY|QBZ63090.1 NCRA|XP_958369.1 SSCL|APA10676.1

>Orthogroup3136: ANID|CBF83867.1 BCIN|XP_024551519.1 BGRA|VCU41092.1 CFRU|XP_031886585.1 CGLO|KAF3804098.1 CHIG|XP_018162660.1 CVIN|KAF4894847.1 CVYL|A07061 FGRM|XP_011326350.1 MGRA|XP_003852828.1 MLAR|XP_007413744.1 MORY|QBZ55635.1 NCRA|XP_957214.2 SSCL|APA10213.1

>Orthogroup3137: ANID|CBF83881.1 BCIN|XP_024552015.1 BGRA|VDB83861.1 CFRU|XP_031878623.1 CGLO|KAF3798521.1 CHIG|XP_018161198.1 CVIN|KAF4917494.1 CVYL|A03804 FGRM|XP_011326679.1 MGRA|XP_003849846.1 MLAR|XP_007404844.1 MORY|QBZ57197.1 NCRA|XP_011395277.1 SSCL|APA12778.1

>Orthogroup3138: ANID|CBF84043.1 BCIN|XP_024547304.1 BGRA|VCU40104.1 CFRU|XP_031878532.1 CGLO|KAF3798604.1 CHIG|XP_018153931.1 CVIN|KAF4930160.1 CVYL|A04842 FGRM|XP_011324697.1 MGRA|XP_003848469.1 MLAR|XP_007414000.1 MORY|QBZ60866.1 NCRA|XP_959459.2 SSCL|APA14270.1

>Orthogroup3139: ANID|CBF84055.1 BCIN|XP_001552739.2 BGRA|VDB92904.1 CFRU|XP_031879686.1 CGLO|KAF3798187.1 CHIG|XP_018154063.1 CVIN|KAF4919128.1 CVYL|A04936 FGRM|XP_011325442.1 MGRA|XP_003851745.1 MLAR|XP_007405141.1 MORY|QBZ60827.1 NCRA|XP_959949.1 SSCL|APA14080.1

>Orthogroup3140: ANID|CBF84062.1 BCIN|XP_024547112.1 BGRA|VDB92898.1 CFRU|XP_031879767.1 CGLO|KAF3798205.1 CHIG|XP_018153948.1 CVIN|KAF4916811.1 CVYL|A04914 FGRM|XP_011325613.1 MGRA|XP_003851575.1 MLAR|XP_007414721.1 MORY|QBZ60733.1 NCRA|XP_001728355.2 SSCL|APA14010.1

>Orthogroup3141: ANID|CBF84070.1 BCIN|XP_024547169.1 BGRA|VDB92909.1 CFRU|XP_031877653.1 CGLO|KAF3796981.1 CHIG|XP_018153705.1 CVIN|KAF4912160.1 CVYL|A00190 FGRM|XP_011327827.1 MGRA|XP_003848358.1 MLAR|XP_007414203.1 MORY|QBZ60533.1 NCRA|XP_011395179.1 SSCL|APA14068.1

>Orthogroup3142: ANID|CBF84079.1 BCIN|XP_024546061.1 BGRA|VCU39030.1 CFRU|XP_031887822.1 CGLO|KAF3802269.1 CHIG|XP_018154366.1 CVIN|KAF4924431.1 CVYL|A10296 FGRM|XP_011319812.1 MGRA|XP_003847543.1 MLAR|XP_007409650.1 MORY|QBZ65761.1 NCRA|XP_964240.1 SSCL|APA07254.1

>Orthogroup3143: ANID|CBF84085.1 BCIN|XP_001552395.1 BGRA|VCU38782.1 CFRU|XP_031881540.1 CGLO|KAF3805347.1 CHIG|XP_018156766.1 CVIN|KAF4913608.1 CVYL|A10989 FGRM|XP_011328300.1 MGRA|XP_003853848.1 MLAR|XP_007408574.1 MORY|QBZ65569.1 NCRA|XP_965197.3 SSCL|APA11210.1

>Orthogroup3144: ANID|CBF84088.1 BCIN|XP_024551977.1 BGRA|VCU39276.1 CFRU|XP_031893256.1 CGLO|KAF3800825.1 CHIG|XP_018150884.1 CVIN|KAF4922496.1 CVYL|A08724 FGRM|XP_011328045.1 MGRA|XP_003849785.1 MLAR|XP_007406782.1 MORY|QBZ64181.1 NCRA|XP_962248.1 SSCL|APA12685.1

>Orthogroup3145: ANID|CBF84090.1 BCIN|XP_024551979.1 BGRA|VCU39277.1 CFRU|XP_031893253.1 CGLO|KAF3800824.1 CHIG|XP_018150885.1 CVIN|KAF4922499.1 CVYL|A08725 FGRM|XP_011328044.1 MGRA|XP_003849324.1 MLAR|XP_007414754.1 MORY|QBZ64179.1 NCRA|XP_962247.1 SSCL|APA12684.1

>Orthogroup3146: ANID|CBF84101.1 BCIN|XP_001555765.1 BGRA|VDB86359.1 CFRU|XP_031893333.1 CGLO|KAF3808021.1 CHIG|XP_018152360.1 CVIN|KAF4909486.1 CVYL|A09462 FGRM|XP_011317755.1 MGRA|XP_003852825.1 MLAR|XP_007405954.1 MORY|QBZ58091.1 NCRA|XP_960754.1 SSCL|APA10125.1

>Orthogroup3147: ANID|CBF84103.1 BCIN|XP_001555764.1 BGRA|VDB86358.1 CFRU|XP_031893334.1 CGLO|KAF3808022.1 CHIG|XP_018152359.1 CVIN|KAF4909487.1 CVYL|A09461 FGRM|XP_011317756.1 MGRA|XP_003857291.1 MLAR|XP_007408337.1 MORY|QBZ58092.1 NCRA|XP_960755.2 SSCL|APA10124.1

>Orthogroup3148: ANID|CBF84107.1 BCIN|XP_024551380.1 BGRA|VCU41134.1 CFRU|XP_031878426.1 CGLO|KAF3802645.1 CHIG|XP_018162516.1 CVIN|KAF4932009.1 CVYL|A07150 FGRM|XP_011326970.1 MGRA|XP_003857206.1 MLAR|XP_007405452.1 MORY|QBZ55673.1 NCRA|XP_956152.2 SSCL|APA10605.1

>Orthogroup3149: ANID|CBF84116.1 BCIN|XP_024549431.1 BGRA|VDB90666.1 CFRU|XP_031888401.1 CGLO|KAF3803686.1 CHIG|XP_018163772.1 CVIN|KAF4928532.1 CVYL|A14617 FGRM|XP_011316031.1 MGRA|XP_003852573.1 MLAR|XP_007409114.1 MORY|QBZ56000.1 NCRA|XP_964815.1 SSCL|APA09246.1

>Orthogroup3150: ANID|CBF84117.1 BCIN|XP_001549197.1 BGRA|VDB90662.1 CFRU|XP_031890413.1 CGLO|KAF3804793.1 CHIG|XP_018164287.1 CVIN|KAF4929809.1 CVYL|A12280 FGRM|XP_011316228.1 MGRA|XP_003852646.1 MLAR|XP_007418630.1 MORY|QBZ55357.1 NCRA|XP_964021.1 SSCL|APA09247.1

>Orthogroup3151: ANID|CBF84119.1 BCIN|XP_024548699.1 BGRA|VCU40232.1 CFRU|XP_031889794.1 CGLO|KAF3805876.1 CHIG|XP_018158885.1 CVIN|KAF4920784.1 CVYL|A13837 FGRM|XP_011316597.1 MGRA|XP_003852630.1 MLAR|XP_007404149.1 MORY|QBZ56101.1 NCRA|XP_965410.1 SSCL|APA09870.1

>Orthogroup3152: ANID|CBF84123.1 BCIN|XP_001549250.2 BGRA|VDB91068.1 CFRU|XP_031888434.1 CGLO|KAF3803465.1 CHIG|XP_018163995.1 CVIN|KAF4926292.1 CVYL|A14395 FGRM|XP_011326481.1 MGRA|XP_003852372.1 MLAR|XP_007403595.1 MORY|QBZ63492.1 NCRA|XP_011393165.1 SSCL|APA09204.1

>Orthogroup3153: ANID|CBF84124.1 BCIN|XP_024549452.1 BGRA|VDB91030.1 CFRU|XP_031890655.1 CGLO|KAF3804912.1 CHIG|XP_018164351.1 CVIN|KAF4919398.1 CVYL|A12146 FGRM|XP_011317152.1 MGRA|XP_003853228.1 MLAR|XP_007416802.1 MORY|QBZ56450.1 NCRA|XP_964266.3 SSCL|APA09207.1

>Orthogroup3154: ANID|CBF84128.1 BCIN|XP_024549450.1 BGRA|VDB91063.1 CFRU|XP_031888432.1 CGLO|KAF3803463.1 CHIG|XP_018163993.1 CVIN|KAF4926293.1 CVYL|A14393 FGRM|XP_011326479.1 MGRA|XP_003852373.1 MLAR|XP_007410710.1 MORY|QBZ63487.1 NCRA|XP_958415.1 SSCL|APA09209.1

>Orthogroup3157: ANID|CBF84739.1 BCIN|XP_001548812.1 BGRA|VDB93644.1 CFRU|XP_031879869.1 CGLO|KAF3802968.1 CHIG|XP_018153645.1 CVIN|KAF4929995.1 CVYL|A00400 FGRM|XP_011316888.1 MGRA|XP_003857334.1 MLAR|XP_007412872.1 MORY|QBZ63534.1 NCRA|XP_965370.1 SSCL|APA13341.1

>Orthogroup3159: ANID|CBF84745.1 BCIN|XP_001558936.1 BGRA|VDB93512.1 CFRU|XP_031888342.1 CGLO|KAF3803565.1 CHIG|XP_018164122.1 CVIN|KAF4922253.1 CVYL|A14498 FGRM|XP_011316786.1 MGRA|XP_003857133.1 MLAR|XP_007411499.1 MORY|QBZ56405.1 NCRA|XP_964458.1 SSCL|APA05809.1

>Orthogroup3160: ANID|CBF84749.1 BCIN|XP_001553699.2 BGRA|VDB93898.1 CFRU|XP_031890399.1 CGLO|KAF3804804.1 CHIG|XP_018164299.1 CVIN|KAF4929757.1 CVYL|A12268 FGRM|XP_011316306.1 MGRA|XP_003857346.1 MLAR|XP_007415567.1 MORY|QBZ62794.1 NCRA|XP_965442.1 SSCL|APA13483.1

>Orthogroup3161: ANID|CBF84754.1 BCIN|XP_001547960.2 BGRA|VDB93629.1 CFRU|XP_031888256.1 CGLO|KAF3803495.1 CHIG|XP_018164025.1 CVIN|KAF4926278.1 CVYL|A14424 FGRM|XP_011326516.1 MGRA|XP_003857170.1 MLAR|XP_007408419.1 MORY|QBZ54717.1 NCRA|XP_011393126.1 SSCL|APA13594.1

>Orthogroup3162: ANID|CBF84756.1 BCIN|XP_001559004.1 BGRA|VDB93830.1 CFRU|XP_031877876.1 CGLO|KAF3804768.1 CHIG|XP_018164257.1 CVIN|KAF4929769.1 CVYL|A12312 FGRM|XP_011316712.1 MGRA|XP_003857348.1 MLAR|XP_007416563.1 MORY|QBZ55531.1 NCRA|XP_964321.1 SSCL|APA05855.1

>Orthogroup3164: ANID|CBF84773.1 BCIN|XP_024548723.1 BGRA|VDB84066.1 CFRU|XP_031890634.1 CGLO|KAF3805282.1 CHIG|XP_018158602.1 CVIN|KAF4926006.1 CVYL|A13933 FGRM|XP_011316641.1 MGRA|XP_003852188.1 MLAR|XP_007409403.1 MORY|QBZ60502.1 NCRA|XP_965697.3 SSCL|APA09792.1

>Orthogroup3165: ANID|CBF84779.1 BCIN|XP_001550630.2 BGRA|VDB84075.1 CFRU|XP_031884591.1 CGLO|KAF3799049.1 CHIG|XP_018158205.1 CVIN|KAF4918250.1 CVYL|A11481 FGRM|XP_011316463.1 MGRA|XP_003853259.1 MLAR|XP_007410529.1 MORY|QBZ55127.1 NCRA|XP_958645.2 SSCL|APA09370.1

>Orthogroup3166: ANID|CBF84782.1 BCIN|XP_001550377.1 BGRA|VCU40219.1 CFRU|XP_031890359.1 CGLO|KAF3806687.1 CHIG|XP_018158656.1 CVIN|KAF4925992.1 CVYL|A13991 FGRM|XP_011323379.1 MGRA|XP_003852216.1 MLAR|XP_007404315.1 MORY|QBZ62568.1 NCRA|XP_964606.3 SSCL|APA10067.1

>Orthogroup3167: ANID|CBF84797.1 BCIN|XP_024548986.1 BGRA|VDB84100.1 CFRU|XP_031886579.1 CGLO|KAF3798907.1 CHIG|XP_018158588.1 CVIN|KAF4925538.1 CVYL|A05045 FGRM|XP_011328436.1 MGRA|XP_003852190.1 MLAR|XP_007405064.1 MORY|QBZ60490.1 NCRA|XP_965703.3 SSCL|APA09364.1

>Orthogroup3170: ANID|CBF84862.1 BCIN|XP_024551315.1 BGRA|VCU41079.1 CFRU|XP_031886702.1 CGLO|KAF3804063.1 CHIG|XP_018162708.1 CVIN|KAF4919560.1 CVYL|A07025 FGRM|XP_011326953.1 MGRA|XP_003852858.1 MLAR|XP_007404898.1 MORY|QBZ61437.1 NCRA|XP_957124.1 SSCL|APA10561.1

>Orthogroup3172: ANID|CBF84871.1 BCIN|XP_001553815.1 BGRA|VCU39415.1 CFRU|XP_031882947.1 CGLO|KAF3803378.1 CHIG|XP_018163952.1 CVIN|KAF4887104.1 CVYL|A14305 FGRM|XP_011317146.1 MGRA|XP_003857603.1 MLAR|XP_007418320.1 MORY|QBZ62772.1 NCRA|XP_965149.2 SSCL|APA05911.1

>Orthogroup3173: ANID|CBF84890.1 BCIN|XP_001558877.1 BGRA|VDB93687.1 CFRU|XP_031892391.1 CGLO|KAF3812283.1 CHIG|XP_018153509.1 CVIN|KAF4919886.1 CVYL|A00099 FGRM|XP_011316096.1 MGRA|XP_003857162.1 MLAR|XP_007415459.1 MORY|QBZ55567.1 NCRA|XP_958915.1 SSCL|APA05778.1

>Orthogroup3174: ANID|CBF84893.1 BCIN|XP_001558878.1 BGRA|VDB93700.1 CFRU|XP_031892392.1 CGLO|KAF3812284.1 CHIG|XP_018153510.1 CVIN|KAF4919885.1 CVYL|A00098 FGRM|XP_011316097.1 MGRA|XP_003856327.1 MLAR|XP_007404683.1 MORY|QBZ55568.1 NCRA|XP_001728408.1 SSCL|APA05779.1

>Orthogroup3175: ANID|CBF84903.1 BCIN|XP_024546940.1 BGRA|VDB93715.1 CFRU|XP_031890418.1 CGLO|KAF3804799.1 CHIG|XP_018164293.1 CVIN|KAF4929753.1 CVYL|A12272 FGRM|XP_011316298.1 MGRA|XP_003856646.1 MLAR|XP_007404455.1 MORY|QBZ55352.1 NCRA|XP_963926.2 SSCL|APA05800.1

>Orthogroup3177: ANID|CBF84906.1 BCIN|XP_024548847.1 BGRA|VDB89405.1 CFRU|XP_031885771.1 CGLO|KAF3810056.1 CHIG|XP_018157771.1 CVIN|KAF4898735.1 CVYL|A02653 FGRM|XP_011321149.1 MGRA|XP_003856547.1 MLAR|XP_007416691.1 MORY|QBZ53619.1 NCRA|XP_963415.1 SSCL|APA09473.1

>Orthogroup3178: ANID|CBF84932.1 BCIN|XP_001561401.1 BGRA|VDB90756.1 CFRU|XP_031888233.1 CGLO|KAF3803475.1 CHIG|XP_018164005.1 CVIN|KAF4926345.1 CVYL|A14405 FGRM|XP_011316865.1 MGRA|XP_003852649.1 MLAR|XP_007419223.1 MORY|QBZ55348.1 NCRA|XP_965448.1 SSCL|APA06912.1

>Orthogroup3179: ANID|CBF84937.1 BCIN|XP_001561379.1 BGRA|VDB90703.1 CFRU|XP_031888240.1 CGLO|KAF3803469.1 CHIG|XP_018163999.1 CVIN|KAF4926318.1 CVYL|A14399 FGRM|XP_011326486.1 MGRA|XP_003852606.1 MLAR|XP_007408730.1 MORY|QBZ63488.1 NCRA|XP_958483.1 SSCL|APA06891.1

>Orthogroup3180: ANID|CBF84947.1 BCIN|XP_001561403.1 BGRA|VDB90751.1 CFRU|XP_031888451.1 CGLO|KAF3803485.1 CHIG|XP_018164015.1 CVIN|KAF4926314.1 CVYL|A14414 FGRM|XP_011316984.1 MGRA|XP_003853127.1 MLAR|XP_007405247.1 MORY|QBZ55339.1 NCRA|XP_965458.1 SSCL|APA06914.1

>Orthogroup3181: ANID|CBF84964.1 BCIN|XP_024548861.1 BGRA|VCU40250.1 CFRU|XP_031890605.1 CGLO|KAF3805264.1 CHIG|XP_018158811.1 CVIN|KAF4918689.1 CVYL|A13913 FGRM|XP_011317299.1 MGRA|XP_003852651.1 MLAR|XP_007412559.1 MORY|QBZ60365.1 NCRA|XP_965271.1 SSCL|APA09438.1

>Orthogroup3182: ANID|CBF84968.1 BCIN|XP_024551184.1 BGRA|VCU39517.1 CFRU|XP_031893495.1 CGLO|KAF3808000.1 CHIG|XP_018152379.1 CVIN|KAF4895815.1 CVYL|A09482 FGRM|XP_011317730.1 MGRA|XP_003848393.1 MLAR|XP_007416022.1 MORY|QBZ54477.1 NCRA|XP_959176.1 SSCL|APA10159.1

>Orthogroup3183: ANID|CBF84996.1 BCIN|XP_001554078.1 BGRA|VDB88829.1 CFRU|XP_031888380.1 CGLO|KAF3803635.1 CHIG|XP_018163824.1 CVIN|KAF4913952.1 CVYL|A14570 FGRM|XP_011317064.1 MGRA|XP_003851857.1 MLAR|XP_007412308.1 MORY|QBZ54692.1 NCRA|XP_011393313.1 SSCL|APA05563.1

>Orthogroup3184: ANID|CBF85017.1 BCIN|XP_001548628.2 BGRA|VCU40245.1 CFRU|XP_031890633.1 CGLO|KAF3805283.1 CHIG|XP_018158603.1 CVIN|KAF4926002.1 CVYL|A13935 FGRM|XP_011316640.1 MGRA|XP_003852376.1 MLAR|XP_007412382.1 MORY|QBZ60503.1 NCRA|XP_965696.2 SSCL|APA09646.1

>Orthogroup3185: ANID|CBF85019.1 BCIN|XP_001548629.1 BGRA|VCU40246.1 CFRU|XP_031890632.1 CGLO|KAF3805284.1 CHIG|XP_018158604.1 CVIN|KAF4926001.1 CVYL|A13936 FGRM|XP_011316638.1 MGRA|XP_003852435.1 MLAR|XP_007409243.1 MORY|QBZ60504.1 NCRA|XP_965695.1 SSCL|APA09645.1

>Orthogroup3186: ANID|CBF85028.1 BCIN|XP_001553722.1 BGRA|VDB93691.1 CFRU|XP_031892725.1 CGLO|KAF3812253.1 CHIG|XP_018153455.1 CVIN|KAF4919939.1 CVYL|A00132 FGRM|XP_011316945.1 MGRA|XP_003857125.1 MLAR|XP_007405363.1 MORY|QBZ55261.1 NCRA|XP_965347.3 SSCL|APA13493.1

>Orthogroup3187: ANID|CBF85037.1 BCIN|XP_024553259.1 BGRA|VDB93723.1 CFRU|XP_031882841.1 CGLO|KAF3803425.1 CHIG|XP_018163909.1 CVIN|KAF4926327.1 CVYL|A14355 FGRM|XP_011316994.1 MGRA|XP_003857138.1 MLAR|XP_007415560.1 MORY|QBZ62791.1 NCRA|XP_965434.3 SSCL|APA13580.1

>Orthogroup3188: ANID|CBF85039.1 BCIN|XP_024553258.1 BGRA|VDB93711.1 CFRU|XP_031882840.1 CGLO|KAF3803424.1 CHIG|XP_018163908.1 CVIN|KAF4926330.1 CVYL|A14354 FGRM|XP_011316993.1 MGRA|XP_003857037.1 MLAR|XP_007411986.1 MORY|QBZ62789.1 NCRA|XP_965433.3 SSCL|APA13581.1

>Orthogroup3189: ANID|CBF85046.1 BCIN|XP_001547946.1 BGRA|VDB93662.1 CFRU|XP_031892649.1 CGLO|KAF3812267.1 CHIG|XP_018153490.1 CVIN|KAF4919911.1 CVYL|A00118 FGRM|XP_011324160.1 MGRA|XP_003856612.1 MLAR|XP_007406582.1 MORY|QBZ55271.1 NCRA|XP_965159.1 SSCL|APA13605.1

>Orthogroup3190: ANID|CBF85065.1 BCIN|XP_024547062.1 BGRA|VDB93673.1 CFRU|XP_031882894.1 CGLO|KAF3812089.1 CHIG|XP_018164160.1 CVIN|KAF4915994.1 CVYL|A14267 FGRM|XP_011316186.1 MGRA|XP_003857153.1 MLAR|XP_007416109.1 MORY|QBZ55557.1 NCRA|XP_011393063.1 SSCL|APA06133.1

>Orthogroup3191: ANID|CBF85067.1 BCIN|XP_001553748.1 BGRA|VDB93674.1 CFRU|XP_031890661.1 CGLO|KAF3804939.1 CHIG|XP_018164441.1 CVIN|KAF4919435.1 CVYL|A12115 FGRM|XP_011316883.1 MGRA|XP_003856740.1 MLAR|XP_007409768.1 MORY|QBZ55558.1 NCRA|XP_963893.2 SSCL|APA06132.1

>Orthogroup3192: ANID|CBF85069.1 BCIN|XP_001553764.1 BGRA|VDB93677.1 CFRU|XP_031888559.1 CGLO|KAF3803605.1 CHIG|XP_018163858.1 CVIN|KAF4920380.1 CVYL|A14540 FGRM|XP_011316314.1 MGRA|XP_003856983.1 MLAR|XP_007407594.1 MORY|QBZ62998.1 NCRA|XP_958134.1 SSCL|APA06117.1

>Orthogroup3193: ANID|CBF85098.1 BCIN|XP_024546182.1 BGRA|VDB90500.1 CFRU|XP_031890397.1 CGLO|KAF3804808.1 CHIG|XP_018164305.1 CVIN|KAF4929817.1 CVYL|A12263 FGRM|XP_011317053.1 MGRA|XP_003852609.1 MLAR|XP_007415359.1 MORY|QBZ54743.1 NCRA|XP_011393273.1 SSCL|APA07046.1

>Orthogroup3195: ANID|CBF85111.1 BCIN|XP_024546542.1 BGRA|VDB90819.1 CFRU|XP_031890599.1 CGLO|KAF3804814.1 CHIG|XP_018164326.1 CVIN|KAF4929819.1 CVYL|A12260 FGRM|XP_011317051.1 MGRA|XP_003852488.1 MLAR|XP_007404249.1 MORY|QBZ54740.1 NCRA|XP_964693.2 SSCL|APA06720.1

>Orthogroup3196: ANID|CBF85122.1 BCIN|XP_001550344.1 BGRA|VDB84104.1 CFRU|XP_031876039.1 CGLO|KAF3805239.1 CHIG|XP_018158835.1 CVIN|KAF4918672.1 CVYL|A13886 FGRM|XP_011317209.1 MGRA|XP_003852656.1 MLAR|XP_007416885.1 MORY|QBZ62495.1 NCRA|XP_963855.1 SSCL|APA10047.1

>Orthogroup3197: ANID|CBF85252.1 BCIN|XP_024550946.1 BGRA|VCU40931.1 CFRU|XP_031880209.1 CGLO|KAF3809079.1 CHIG|XP_018155919.1 CVIN|KAF4919740.1 CVYL|A01633 FGRM|XP_011318894.1 MGRA|XP_003851179.1 MLAR|XP_007403950.1 MORY|QBZ57365.1 NCRA|XP_961057.1 SSCL|APA11714.1

>Orthogroup3198: ANID|CBF85271.1 BCIN|XP_024546283.1 BGRA|VDB90828.1 CFRU|XP_031890641.1 CGLO|KAF3804924.1 CHIG|XP_018164423.1 CVIN|KAF4919405.1 CVYL|A12130 FGRM|XP_011320448.1 MGRA|XP_003851181.1 MLAR|XP_007404988.1 MORY|QBZ63764.1 NCRA|XP_959172.2 SSCL|APA06946.1

>Orthogroup3199: ANID|CBF85301.1 BCIN|XP_024549584.1 BGRA|VDB89366.1 CFRU|XP_031891471.1 CGLO|KAF3804495.1 CHIG|XP_018160323.1 CVIN|KAF4930979.1 CVYL|A04210 FGRM|XP_011324748.1 MGRA|XP_003851254.1 MLAR|XP_007416851.1 MORY|QBZ59105.1 NCRA|XP_961117.3 SSCL|APA13971.1

>Orthogroup3200: ANID|CBF85312.1 BCIN|XP_024546429.1 BGRA|VCU40689.1 CFRU|XP_031876041.1 CGLO|KAF3805241.1 CHIG|XP_018158833.1 CVIN|KAF4918660.1 CVYL|A13888 FGRM|XP_011317294.1 MGRA|XP_003853443.1 MLAR|XP_007417851.1 MORY|QBZ60345.1 NCRA|XP_011393028.1 SSCL|APA07109.1

>Orthogroup3201: ANID|CBF85319.1 BCIN|XP_024549377.1 BGRA|VCU40688.1 CFRU|XP_031876052.1 CGLO|KAF3805243.1 CHIG|XP_018158831.1 CVIN|KAF4918653.1 CVYL|A13891 FGRM|XP_011317320.1 MGRA|XP_003852965.1 MLAR|XP_007416372.1 MORY|QBZ60348.1 NCRA|XP_963944.1 SSCL|APA08950.1

>Orthogroup3202: ANID|CBF85327.1 BCIN|XP_024549123.1 BGRA|VCU39883.1 CFRU|XP_031889555.1 CGLO|KAF3810440.1 CHIG|XP_018159177.1 CVIN|KAF4924619.1 CVYL|A13845 FGRM|XP_011327296.1 MGRA|XP_003857309.1 MLAR|XP_007418400.1 MORY|QBZ62546.1 NCRA|XP_011395274.1 SSCL|APA08842.1

>Orthogroup3203: ANID|CBF85385.1 BCIN|XP_001556540.1 BGRA|VCU40815.1 CFRU|XP_031889529.1 CGLO|KAF3810448.1 CHIG|XP_018151472.1 CVIN|KAF4924618.1 CVYL|A13900 FGRM|XP_011327301.1 MGRA|XP_003852993.1 MLAR|XP_007405008.1 MORY|QBZ56887.1 NCRA|XP_959629.1 SSCL|APA06802.1

>Orthogroup3204: ANID|CBF85389.1 BCIN|XP_001556410.2 BGRA|VCU40874.1 CFRU|XP_031879646.1 CGLO|KAF3802693.1 CHIG|XP_018151480.1 CVIN|KAF4899840.1 CVYL|A08018 FGRM|XP_011327274.1 MGRA|XP_003852139.1 MLAR|XP_007403550.1 MORY|QBZ58700.1 NCRA|XP_958035.1 SSCL|APA06836.1

>Orthogroup3205: ANID|CBF85391.1 BCIN|XP_024546649.1 BGRA|VCU40828.1 CFRU|XP_031889786.1 CGLO|KAF3810446.1 CHIG|XP_018151474.1 CVIN|KAF4924624.1 CVYL|A13889 FGRM|XP_011327300.1 MGRA|XP_003852108.1 MLAR|XP_007406441.1 MORY|QBZ56888.1 NCRA|XP_959276.3 SSCL|APA06801.1

>Orthogroup3206: ANID|CBF85394.1 BCIN|XP_024546718.1 BGRA|VCU40875.1 CFRU|XP_031879581.1 CGLO|KAF3802691.1 CHIG|XP_018151479.1 CVIN|KAF4889149.1 CVYL|A08016 FGRM|XP_011327275.1 MGRA|XP_003852686.1 MLAR|XP_007415679.1 MORY|QBZ58701.1 NCRA|XP_958036.1 SSCL|APA06835.1

>Orthogroup3207: ANID|CBF85409.1 BCIN|XP_024546240.1 BGRA|VDB92561.1 CFRU|XP_031889559.1 CGLO|KAF3810462.1 CHIG|XP_018159195.1 CVIN|KAF4924612.1 CVYL|A14056 FGRM|XP_011327328.1 MGRA|XP_003856303.1 MLAR|XP_007404838.1 MORY|QBZ62541.1 NCRA|XP_959678.3 SSCL|APA06987.1

>Orthogroup3209: ANID|CBF85414.1 BCIN|XP_001557972.1 BGRA|VDB92741.1 CFRU|XP_031887765.1 CGLO|KAF3803273.1 CHIG|XP_018161122.1 CVIN|KAF4928638.1 CVYL|A03708 FGRM|XP_011327960.1 MGRA|XP_003849542.1 MLAR|XP_007404581.1 MORY|QBZ58368.1 NCRA|XP_961964.1 SSCL|APA12678.1

>Orthogroup3210: ANID|CBF85416.1 BCIN|XP_024546657.1 BGRA|VCU40827.1 CFRU|XP_031879629.1 CGLO|KAF3802679.1 CHIG|XP_018159160.1 CVIN|KAF4920400.1 CVYL|A08070 FGRM|XP_011327319.1 MGRA|XP_003853003.1 MLAR|XP_007403598.1 MORY|QBZ57771.1 NCRA|XP_958751.2 SSCL|APA06808.1

>Orthogroup3211: ANID|CBF85442.1 BCIN|XP_024552490.1 BGRA|VDB87775.1 CFRU|XP_031885588.1 CGLO|KAF3810203.1 CHIG|XP_018157824.1 CVIN|KAF4929177.1 CVYL|A02798 FGRM|XP_011328533.1 MGRA|XP_003853069.1 MLAR|XP_007405097.1 MORY|QBZ64935.1 NCRA|XP_960181.2 SSCL|APA08517.1

>Orthogroup3212: ANID|CBF85447.1 BCIN|XP_024552483.1 BGRA|VDB87808.1 CFRU|XP_031885629.1 CGLO|KAF3810145.1 CHIG|XP_018157975.1 CVIN|KAF4929153.1 CVYL|A02734 FGRM|XP_011328541.1 MGRA|XP_003853334.1 MLAR|XP_007408784.1 MORY|QBZ56103.1 NCRA|XP_957689.1 SSCL|APA08529.1

>Orthogroup3213: ANID|CBF85449.1 BCIN|XP_001555328.1 BGRA|VDB87896.1 CFRU|XP_031883375.1 CGLO|KAF3801723.1 CHIG|XP_018159238.1 CVIN|KAF4922944.1 CVYL|A12978 FGRM|XP_011320401.1 MGRA|XP_003853451.1 MLAR|XP_007407971.1 MORY|QBZ62852.1 NCRA|XP_960152.1 SSCL|APA08614.1

>Orthogroup3215: ANID|CBF85480.1 BCIN|XP_024552491.1 BGRA|VDB87773.1 CFRU|XP_031885592.1 CGLO|KAF3810206.1 CHIG|XP_018157828.1 CVIN|KAF4929183.1 CVYL|A02802 FGRM|XP_011328535.1 MGRA|XP_003853338.1 MLAR|XP_007410671.1 MORY|QBZ64937.1 NCRA|XP_960179.1 SSCL|APA08516.1

>Orthogroup3216: ANID|CBF85486.1 BCIN|XP_024552486.1 BGRA|VDB87696.1 CFRU|XP_031885590.1 CGLO|KAF3810202.1 CHIG|XP_018157823.1 CVIN|KAF4929178.1 CVYL|A02797 FGRM|XP_011328532.1 MGRA|XP_003856467.1 MLAR|XP_007410557.1 MORY|QBZ64933.1 NCRA|XP_011395015.1 SSCL|APA08523.1

>Orthogroup3217: ANID|CBF85494.1 BCIN|XP_024552625.1 BGRA|VDB87902.1 CFRU|XP_031878331.1 CGLO|KAF3798048.1 CHIG|XP_018158095.1 CVIN|KAF4928011.1 CVYL|A08308 FGRM|XP_011328461.1 MGRA|XP_003852974.1 MLAR|XP_007418766.1 MORY|QBZ62732.1 NCRA|XP_957655.1 SSCL|APA08714.1

>Orthogroup3218: ANID|CBF85496.1 BCIN|XP_001556307.1 BGRA|VDB87880.1 CFRU|XP_031885795.1 CGLO|KAF3810115.1 CHIG|XP_018157867.1 CVIN|KAF4910239.1 CVYL|A02707 FGRM|XP_011320304.1 MGRA|XP_003857311.1 MLAR|XP_007409825.1 MORY|QBZ62264.1 NCRA|XP_957692.1 SSCL|APA15693.1

>Orthogroup3220: ANID|CBF85505.1 BCIN|XP_024550609.1 BGRA|VCU40781.1 CFRU|XP_031889085.1 CGLO|KAF3797670.1 CHIG|XP_018155443.1 CVIN|KAF4919364.1 CVYL|A01100 FGRM|XP_011323889.1 MGRA|XP_003853584.1 MLAR|XP_007410677.1 MORY|QBZ57696.1 NCRA|XP_958786.1 SSCL|APA11423.1

>Orthogroup3221: ANID|CBF85511.1 BCIN|XP_024553634.1 BGRA|VDB87608.1 CFRU|XP_031890806.1 CGLO|KAF3801635.1 CHIG|XP_018151400.1 CVIN|KAF4921221.1 CVYL|A12502 FGRM|XP_011328449.1 MGRA|XP_003853485.1 MLAR|XP_007416406.1 MORY|QBZ62686.1 NCRA|XP_957956.3 SSCL|APA08566.1

>Orthogroup3224: ANID|CBF85530.1 BCIN|XP_001557034.1 BGRA|VDB87659.1 CFRU|XP_031891011.1 CGLO|KAF3801640.1 CHIG|XP_018151407.1 CVIN|KAF4921238.1 CVYL|A12496 FGRM|XP_011328454.1 MGRA|XP_003852869.1 MLAR|XP_007417277.1 MORY|QBZ64401.1 NCRA|XP_957682.1 SSCL|APA08493.1

>Orthogroup3225: ANID|CBF85539.1 BCIN|XP_024550616.1 BGRA|VCU40774.1 CFRU|XP_031889215.1 CGLO|KAF3808737.1 CHIG|XP_018155638.1 CVIN|KAF4929586.1 CVYL|A00925 FGRM|XP_011318078.1 MGRA|XP_003856187.1 MLAR|XP_007413338.1 MORY|QBZ65001.1 NCRA|XP_960205.1 SSCL|APA11433.1

>Orthogroup3226: ANID|CBF85545.1 BCIN|XP_001546899.2 BGRA|VDB92917.1 CFRU|XP_031890805.1 CGLO|KAF3801634.1 CHIG|XP_018151399.1 CVIN|KAF4921222.1 CVYL|A12503 FGRM|XP_011328448.1 MGRA|XP_003852934.1 MLAR|XP_007405994.1 MORY|QBZ62685.1 NCRA|XP_957955.2 SSCL|APA08567.1

>Orthogroup3227: ANID|CBF85548.1 BCIN|XP_024553636.1 BGRA|VDB89724.1 CFRU|XP_031890809.1 CGLO|KAF3801638.1 CHIG|XP_018151403.1 CVIN|KAF4921226.1 CVYL|A12499 FGRM|XP_011328452.1 MGRA|XP_003852725.1 MLAR|XP_007419189.1 MORY|QBZ62688.1 NCRA|XP_957892.2 SSCL|APA08565.1

>Orthogroup3232: ANID|CBF85675.1 BCIN|XP_001545954.1 BGRA|VDB90804.1 CFRU|XP_031888269.1 CGLO|KAF3803507.1 CHIG|XP_018164038.1 CVIN|KAF4908042.1 CVYL|A14437 FGRM|XP_011316787.1 MGRA|XP_003850710.1 MLAR|XP_007407487.1 MORY|QBZ62809.1 NCRA|XP_958487.1 SSCL|APA07038.1

>Orthogroup3233: ANID|CBF85686.1 BCIN|XP_024553730.1 BGRA|VDB93448.1 CFRU|XP_031885209.1 CGLO|KAF3800347.1 CHIG|XP_018162836.1 CVIN|KAF4914536.1 CVYL|A07486 FGRM|XP_011326408.1 MGRA|XP_003854311.1 MLAR|XP_007412312.1 MORY|QBZ63238.1 NCRA|XP_958612.1 SSCL|APA15119.1

>Orthogroup3235: ANID|CBF85733.1 BCIN|XP_024552667.1 BGRA|VDB87788.1 CFRU|XP_031885666.1 CGLO|KAF3810236.1 CHIG|XP_018157880.1 CVIN|KAF4929089.1 CVYL|A02836 FGRM|XP_011325092.1 MGRA|XP_003853071.1 MLAR|XP_007418590.1 MORY|QBZ53907.1 NCRA|XP_957639.1 SSCL|APA15550.1

>Orthogroup3236: ANID|CBF85735.1 BCIN|XP_001547036.1 BGRA|VDB87689.1 CFRU|XP_031885669.1 CGLO|KAF3810237.1 CHIG|XP_018157878.1 CVIN|KAF4929088.1 CVYL|A02837 FGRM|XP_011325093.1 MGRA|XP_003853436.1 MLAR|XP_007416454.1 MORY|QBZ53906.1 NCRA|XP_957637.1 SSCL|APA15551.1

>Orthogroup3237: ANID|CBF85741.1 BCIN|XP_024553611.1 BGRA|VDB83814.1 CFRU|XP_031885822.1 CGLO|KAF3810133.1 CHIG|XP_018157965.1 CVIN|KAF4912132.1 CVYL|A02723 FGRM|XP_011328245.1 MGRA|XP_003853530.1 MLAR|XP_007412985.1 MORY|QBZ59271.1 NCRA|XP_957567.3 SSCL|APA08457.1

>Orthogroup3239: ANID|CBF85744.1 BCIN|XP_024553574.1 BGRA|VDB87677.1 CFRU|XP_031889429.1 CGLO|KAF3808741.1 CHIG|XP_018155634.1 CVIN|KAF4929582.1 CVYL|A00921 FGRM|XP_011318142.1 MGRA|XP_003853540.1 MLAR|XP_007417358.1 MORY|QBZ64467.1 NCRA|XP_960166.3 SSCL|APA08431.1

>Orthogroup3242: ANID|CBF85808.1 BCIN|XP_001554781.1 BGRA|VDB87751.1 CFRU|XP_031889197.1 CGLO|KAF3803172.1 CHIG|XP_018155504.1 CVIN|KAF4928344.1 CVYL|A01060 FGRM|XP_011323867.1 MGRA|XP_003853415.1 MLAR|XP_007405314.1 MORY|QBZ58642.1 NCRA|XP_958781.1 SSCL|APA15506.1

>Orthogroup3243: ANID|CBF85812.1 BCIN|XP_001555093.1 BGRA|VCU40793.1 CFRU|XP_031885791.1 CGLO|KAF3810111.1 CHIG|XP_018157863.1 CVIN|KAF4910242.1 CVYL|A02704 FGRM|XP_011319965.1 MGRA|XP_003853440.1 MLAR|XP_007407573.1 MORY|QBZ59276.1 NCRA|XP_957635.1 SSCL|APA08463.1

>Orthogroup3244: ANID|CBF85815.1 BCIN|XP_024553588.1 BGRA|VDB87888.1 CFRU|XP_031885800.1 CGLO|KAF3810110.1 CHIG|XP_018157862.1 CVIN|KAF4910236.1 CVYL|A02703 FGRM|XP_011319966.1 MGRA|XP_003857212.1 MLAR|XP_007413869.1 MORY|QBZ59280.1 NCRA|XP_960443.1 SSCL|APA08441.1

>Orthogroup3245: ANID|CBF85824.1 BCIN|XP_001546896.2 BGRA|VDB89735.1 CFRU|XP_031883409.1 CGLO|KAF3801713.1 CHIG|XP_018159229.1 CVIN|KAF4915638.1 CVYL|A12986 FGRM|XP_011320148.1 MGRA|XP_003852732.1 MLAR|XP_007409264.1 MORY|QBZ62860.1 NCRA|XP_960155.2 SSCL|APA08569.1

>Orthogroup3246: ANID|CBF85832.1 BCIN|XP_001557047.1 BGRA|VDB87796.1 CFRU|XP_031885854.1 CGLO|KAF3810222.1 CHIG|XP_018157847.1 CVIN|KAF4929188.1 CVYL|A02819 FGRM|XP_011320324.1 MGRA|XP_003857295.1 MLAR|XP_007404358.1 MORY|QBZ61294.1 NCRA|XP_957611.1 SSCL|APA08502.1

>Orthogroup3247: ANID|CBF85850.1 BCIN|XP_001556393.2 BGRA|VDB88204.1 CFRU|XP_031885694.1 CGLO|KAF3810102.1 CHIG|XP_018157853.1 CVIN|KAF4910251.1 CVYL|A02695 FGRM|XP_011328219.1 MGRA|XP_003853539.1 MLAR|XP_007412398.1 MORY|QBZ53890.1 NCRA|XP_957530.2 SSCL|APA08697.1

>Orthogroup3248: ANID|CBF85851.1 BCIN|XP_024552866.1 BGRA|VDB93029.1 CFRU|XP_031882380.1 CGLO|KAF3807619.1 CHIG|XP_018156313.1 CVIN|KAF4921412.1 CVYL|A03134 FGRM|XP_011319092.1 MGRA|XP_003853016.1 MLAR|XP_007411097.1 MORY|QBZ54549.1 NCRA|XP_001728226.1 SSCL|APA16118.1

>Orthogroup3249: ANID|CBF85857.1 BCIN|XP_001548203.1 BGRA|VDB88241.1 CFRU|XP_031886294.1 CGLO|KAF3806178.1 CHIG|XP_018162165.1 CVIN|KAF4931550.1 CVYL|A11764 FGRM|XP_011326722.1 MGRA|XP_003853394.1 MLAR|XP_007411141.1 MORY|QBZ63146.1 NCRA|XP_957749.2 SSCL|APA15581.1

>Orthogroup3251: ANID|CBF85914.1 BCIN|XP_001557645.2 BGRA|VDB88686.1 CFRU|XP_031881322.1 CGLO|KAF3802506.1 CHIG|XP_018154811.1 CVIN|KAF4919023.1 CVYL|A02098 FGRM|XP_011316320.1 MGRA|XP_003857079.1 MLAR|XP_007410386.1 MORY|QBZ60967.1 NCRA|XP_956297.1 SSCL|APA12282.1

>Orthogroup3252: ANID|CBF85920.1 BCIN|XP_001554492.1 BGRA|VDB93040.1 CFRU|XP_031885408.1 CGLO|KAF3799429.1 CHIG|XP_018152302.1 CVIN|KAF4931737.1 CVYL|A13293 FGRM|XP_011323653.1 MGRA|XP_003857503.1 MLAR|XP_007410268.1 MORY|QBZ64790.1 NCRA|XP_955769.1 SSCL|APA11834.1

>Orthogroup3253: ANID|CBF85923.1 BCIN|XP_024553096.1 BGRA|VCU39720.1 CFRU|XP_031881299.1 CGLO|KAF3802467.1 CHIG|XP_018154771.1 CVIN|KAF4916869.1 CVYL|A02060 FGRM|XP_011325542.1 MGRA|XP_003857498.1 MLAR|XP_007405076.1 MORY|QBZ62055.1 NCRA|XP_958573.1 SSCL|APA16309.1

>Orthogroup3254: ANID|CBF85929.1 BCIN|XP_024553123.1 BGRA|VCU41250.1 CFRU|XP_031891132.1 CGLO|KAF3801499.1 CHIG|XP_018151929.1 CVIN|KAF4916399.1 CVYL|A12633 FGRM|XP_011325494.1 MGRA|XP_003856160.1 MLAR|XP_007413283.1 MORY|QBZ63148.1 NCRA|XP_963569.2 SSCL|APA16350.1

>Orthogroup3255: ANID|CBF85940.1 BCIN|XP_024548420.1 BGRA|VDB92992.1 CFRU|XP_031881246.1 CGLO|KAF3802496.1 CHIG|XP_018154802.1 CVIN|KAF4919008.1 CVYL|A02088 FGRM|XP_011316323.1 MGRA|XP_003856412.1 MLAR|XP_007411763.1 MORY|QBZ60964.1 NCRA|XP_963428.2 SSCL|APA12275.1

>Orthogroup3256: ANID|CBF85945.1 BCIN|XP_001550209.1 BGRA|VCU41248.1 CFRU|XP_031890889.1 CGLO|KAF3801490.1 CHIG|XP_018151921.1 CVIN|KAF4916384.1 CVYL|A12639 FGRM|XP_011321180.1 MGRA|XP_003856404.1 MLAR|XP_007410486.1 MORY|QBZ55806.1 NCRA|XP_963181.1 SSCL|APA16332.1

>Orthogroup3257: ANID|CBF85954.1 BCIN|XP_001546404.1 BGRA|VDB88781.1 CFRU|XP_031877963.1 CGLO|KAF3810903.1 CHIG|XP_018154936.1 CVIN|KAF4916341.1 CVYL|A02242 FGRM|XP_011324625.1 MGRA|XP_003853313.1 MLAR|XP_007416557.1 MORY|QBZ53819.1 NCRA|XP_958194.1 SSCL|APA12259.1

>Orthogroup3258: ANID|CBF85960.1 BCIN|XP_024553013.1 BGRA|VCU39710.1 CFRU|XP_031885478.1 CGLO|KAF3799486.1 CHIG|XP_018154471.1 CVIN|KAF4931822.1 CVYL|A13348 FGRM|XP_011321193.1 MGRA|XP_003856608.1 MLAR|XP_007418585.1 MORY|QBZ57260.1 NCRA|XP_963533.1 SSCL|APA16196.1

>Orthogroup3259: ANID|CBF85970.1 BCIN|XP_024548293.1 BGRA|VCU40375.1 CFRU|XP_031881789.1 CGLO|KAF3800696.1 CHIG|XP_018157660.1 CVIN|KAF4900986.1 CVYL|A13023 FGRM|XP_011325662.1 MGRA|XP_003857221.1 MLAR|XP_007407231.1 MORY|QBZ61259.1 NCRA|XP_011393605.1 SSCL|APA12542.1

>Orthogroup3261: ANID|CBF85983.1 BCIN|XP_024550465.1 BGRA|VCU39400.1 CFRU|XP_031885844.1 CGLO|KAF3810164.1 CHIG|XP_018157783.1 CVIN|KAF4929158.1 CVYL|A02756 FGRM|XP_011320315.1 MGRA|XP_003854563.1 MLAR|XP_007409967.1 MORY|QBZ62133.1 NCRA|XP_958635.3 SSCL|APA11175.1

>Orthogroup3262: ANID|CBF85990.1 BCIN|XP_024547839.1 BGRA|VDB90405.1 CFRU|XP_031885820.1 CGLO|KAF3810244.1 CHIG|XP_018157952.1 CVIN|KAF4910794.1 CVYL|A02845 FGRM|XP_011319952.1 MGRA|XP_003853700.1 MLAR|XP_007405490.1 MORY|QBZ56051.1 NCRA|XP_958807.2 SSCL|APA08155.1

>Orthogroup3263: ANID|CBF85997.1 BCIN|XP_024552997.1 BGRA|VCU41235.1 CFRU|XP_031885509.1 CGLO|KAF3799503.1 CHIG|XP_018154488.1 CVIN|KAF4931866.1 CVYL|A13364 FGRM|XP_011321244.1 MGRA|XP_003857222.1 MLAR|XP_007415426.1 MORY|QBZ57276.1 NCRA|XP_011393451.1 SSCL|APA16207.1

>Orthogroup3264: ANID|CBF86025.1 BCIN|XP_024553712.1 BGRA|VDB93381.1 CFRU|XP_031886229.1 CGLO|KAF3806237.1 CHIG|XP_018162072.1 CVIN|KAF4931580.1 CVYL|A11827 FGRM|XP_011324016.1 MGRA|XP_003853868.1 MLAR|XP_007403500.1 MORY|QBZ61559.1 NCRA|XP_957221.3 SSCL|APA15106.1

>Orthogroup3266: ANID|CBF86080.1 BCIN|XP_024553657.1 BGRA|VDB93440.1 CFRU|XP_031893490.1 CGLO|KAF3808008.1 CHIG|XP_018152372.1 CVIN|KAF4895809.1 CVYL|A09475 FGRM|XP_011317736.1 MGRA|XP_003854124.1 MLAR|XP_007406363.1 MORY|QBZ54201.1 NCRA|XP_961271.1 SSCL|APA15037.1

>Orthogroup3267: ANID|CBF86082.1 BCIN|XP_001551107.1 BGRA|VDB93442.1 CFRU|XP_031886558.1 CGLO|KAF3804042.1 CHIG|XP_018162714.1 CVIN|KAF4927634.1 CVYL|A06996 FGRM|XP_011326369.1 MGRA|XP_003853734.1 MLAR|XP_007417977.1 MORY|QBZ59306.1 NCRA|XP_956791.1 SSCL|APA15036.1

>Orthogroup3268: ANID|CBF86086.1 BCIN|XP_001552127.1 BGRA|VDB92850.1 CFRU|XP_031886534.1 CGLO|KAF3804028.1 CHIG|XP_018162742.1 CVIN|KAF4927569.1 CVYL|A06980 FGRM|XP_011326378.1 MGRA|XP_003854460.1 MLAR|XP_007410679.1 MORY|QBZ59548.1 NCRA|XP_957827.2 SSCL|APA05274.1

>Orthogroup3269: ANID|CBF86108.1 BCIN|XP_024553648.1 BGRA|VDB89740.1 CFRU|XP_031883402.1 CGLO|KAF3801727.1 CHIG|XP_018159240.1 CVIN|KAF4922940.1 CVYL|A12974 FGRM|XP_011320387.1 MGRA|XP_003852731.1 MLAR|XP_007412767.1 MORY|QBZ62846.1 NCRA|XP_001728299.2 SSCL|APA08560.1

>Orthogroup3270: ANID|CBF86115.1 BCIN|XP_024552628.1 BGRA|VDB88200.1 CFRU|XP_031885811.1 CGLO|KAF3810099.1 CHIG|XP_018157893.1 CVIN|KAF4910244.1 CVYL|A02692 FGRM|XP_011328231.1 MGRA|XP_003852962.1 MLAR|XP_007406012.1 MORY|QBZ61304.1 NCRA|XP_011394885.1 SSCL|APA08703.1

>Orthogroup3271: ANID|CBF86120.1 BCIN|XP_024552454.1 BGRA|VCU40775.1 CFRU|XP_031885779.1 CGLO|KAF3810092.1 CHIG|XP_018157901.1 CVIN|KAF4891969.1 CVYL|A02686 FGRM|XP_011320210.1 MGRA|XP_003853449.1 MLAR|XP_007418201.1 MORY|QBZ59269.1 NCRA|XP_957630.3 SSCL|APA08555.1

>Orthogroup3272: ANID|CBF86122.1 BCIN|XP_024552455.1 BGRA|VCU40776.1 CFRU|XP_031885780.1 CGLO|KAF3810093.1 CHIG|XP_018157900.1 CVIN|KAF4892591.1 CVYL|A02687 FGRM|XP_011320211.1 MGRA|XP_003852960.1 MLAR|XP_007404740.1 MORY|QBZ59268.1 NCRA|XP_957629.2 SSCL|APA08554.1

>Orthogroup3273: ANID|CBF86124.1 BCIN|XP_001547330.1 BGRA|VDB87661.1 CFRU|XP_031883275.1 CGLO|KAF3801720.1 CHIG|XP_018159235.1 CVIN|KAF4922945.1 CVYL|A12981 FGRM|XP_011320272.1 MGRA|XP_003853056.1 MLAR|XP_007414961.1 MORY|QBZ62855.1 NCRA|XP_960397.2 SSCL|APA15638.1

>Orthogroup3274: ANID|CBF86140.1 BCIN|XP_001547323.1 BGRA|VDB89751.1 CFRU|XP_031885750.1 CGLO|KAF3810185.1 CHIG|XP_018157804.1 CVIN|KAF4929122.1 CVYL|A02777 FGRM|XP_011319983.1 MGRA|XP_003853031.1 MLAR|XP_007412857.1 MORY|QBZ61278.1 NCRA|XP_957545.2 SSCL|APA15630.1

>Orthogroup3275: ANID|CBF86148.1 BCIN|XP_001549241.1 BGRA|VDB91066.1 CFRU|XP_031888405.1 CGLO|KAF3803683.1 CHIG|XP_018163768.1 CVIN|KAF4928497.1 CVYL|A14614 FGRM|XP_011316027.1 MGRA|XP_003848293.1 MLAR|XP_007406424.1 MORY|QBZ55415.1 NCRA|XP_964037.1 SSCL|APA09224.1

>Orthogroup3276: ANID|CBF86155.1 BCIN|XP_024549429.1 BGRA|VDB90892.1 CFRU|XP_031882881.1 CGLO|KAF3803371.1 CHIG|XP_018163965.1 CVIN|KAF4915988.1 CVYL|A14290 FGRM|XP_011316812.1 MGRA|XP_003848394.1 MLAR|XP_007416246.1 MORY|QBZ62783.1 NCRA|XP_963814.2 SSCL|APA09243.1

>Orthogroup3277: ANID|CBF86164.1 BCIN|XP_024549414.1 BGRA|VDB91092.1 CFRU|XP_031882812.1 CGLO|KAF3812056.1 CHIG|XP_018164187.1 CVIN|KAF4923702.1 CVYL|A14241 FGRM|XP_011326784.1 MGRA|XP_003848346.1 MLAR|XP_007414770.1 MORY|QBZ63791.1 NCRA|XP_959164.3 SSCL|APA09086.1

>Orthogroup3278: ANID|CBF86166.1 BCIN|XP_024549416.1 BGRA|VDB91091.1 CFRU|XP_031882821.1 CGLO|KAF3812057.1 CHIG|XP_018164186.1 CVIN|KAF4923661.1 CVYL|A14240 FGRM|XP_011326785.1 MGRA|XP_003848633.1 MLAR|XP_007405239.1 MORY|QBZ63790.1 NCRA|XP_959163.2 SSCL|APA09087.1

>Orthogroup3280: ANID|CBF86191.1 BCIN|XP_001558357.1 BGRA|VDB90895.1 CFRU|XP_031882778.1 CGLO|KAF3803364.1 CHIG|XP_018163958.1 CVIN|KAF4915971.1 CVYL|A14297 FGRM|XP_011317150.1 MGRA|XP_003848655.1 MLAR|XP_007415039.1 MORY|QBZ62775.1 NCRA|XP_964676.1 SSCL|APA13762.1

>Orthogroup3282: ANID|CBF86215.1 BCIN|XP_024552489.1 BGRA|VDB87695.1 CFRU|XP_031885709.1 CGLO|KAF3810155.1 CHIG|XP_018157987.1 CVIN|KAF4929105.1 CVYL|A02745 FGRM|XP_011328515.1 MGRA|XP_003853058.1 MLAR|XP_007407788.1 MORY|QBZ62403.1 NCRA|XP_956086.2 SSCL|APA08521.1

>Orthogroup3283: ANID|CBF86216.1 BCIN|XP_001555311.2 BGRA|VDB89727.1 CFRU|XP_031889114.1 CGLO|KAF3803159.1 CHIG|XP_018155523.1 CVIN|KAF4928292.1 CVYL|A01045 FGRM|XP_011318128.1 MGRA|XP_003853062.1 MLAR|XP_007410567.1 MORY|QBZ58473.1 NCRA|XP_960211.3 SSCL|APA08626.1

>Orthogroup3284: ANID|CBF86237.1 BCIN|XP_024550291.1 BGRA|VDB94733.1 CFRU|XP_031878205.1 CGLO|KAF3808532.1 CHIG|XP_018161937.1 CVIN|KAF4917625.1 CVYL|A11614 FGRM|XP_011318525.1 MGRA|XP_003854884.1 MLAR|XP_007410237.1 MORY|QBZ59655.1 NCRA|XP_956786.1 SSCL|APA10957.1

>Orthogroup3285: ANID|CBF86250.1 BCIN|XP_024550543.1 BGRA|VDB94681.1 CFRU|XP_031887066.1 CGLO|KAF3811432.1 CHIG|XP_018160674.1 CVIN|KAF4917854.1 CVYL|A04631 FGRM|XP_011326847.1 MGRA|XP_003855363.1 MLAR|XP_007412407.1 MORY|QBZ54564.1 NCRA|XP_960648.1 SSCL|APA11331.1

>Orthogroup3286: ANID|CBF86259.1 BCIN|XP_001549142.1 BGRA|VCU38808.1 CFRU|XP_031887023.1 CGLO|KAF3811425.1 CHIG|XP_018160664.1 CVIN|KAF4919698.1 CVYL|A04623 FGRM|XP_011327181.1 MGRA|XP_003855271.1 MLAR|XP_007404065.1 MORY|QBZ57323.1 NCRA|XP_961053.1 SSCL|APA15376.1

>Orthogroup3288: ANID|CBF86262.1 BCIN|XP_024550526.1 BGRA|VDB94627.1 CFRU|XP_031887087.1 CGLO|KAF3811436.1 CHIG|XP_018160678.1 CVIN|KAF4917850.1 CVYL|A04635 FGRM|XP_011326851.1 MGRA|XP_003855112.1 MLAR|XP_007406517.1 MORY|QBZ57134.1 NCRA|XP_960652.1 SSCL|APA11253.1

>Orthogroup3289: ANID|CBF86271.1 BCIN|XP_001554039.2 BGRA|VDB94573.1 CFRU|XP_031886942.1 CGLO|KAF3811344.1 CHIG|XP_018160594.1 CVIN|KAF4921001.1 CVYL|A04526 FGRM|XP_011324777.1 MGRA|XP_003855437.1 MLAR|XP_007412537.1 MORY|QBZ56979.1 NCRA|XP_961444.3 SSCL|APA11127.1

>Orthogroup3290: ANID|CBF86275.1 BCIN|XP_024552270.1 BGRA|VCU39300.1 CFRU|XP_031877157.1 CGLO|KAF3804007.1 CHIG|XP_018150942.1 CVIN|KAF4925136.1 CVYL|A08794 FGRM|XP_011323760.1 MGRA|XP_003849755.1 MLAR|XP_007405505.1 MORY|QBZ64295.1 NCRA|XP_955907.2 SSCL|APA13126.1

>Orthogroup3291: ANID|CBF86282.1 BCIN|XP_001552263.1 BGRA|VDB92811.1 CFRU|XP_031890269.1 CGLO|KAF3798718.1 CHIG|XP_018151274.1 CVIN|KAF4910264.1 CVYL|A09194 FGRM|XP_011327920.1 MGRA|XP_003849671.1 MLAR|XP_007404371.1 MORY|QBZ58896.1 NCRA|XP_962036.1 SSCL|APA13044.1

>Orthogroup3292: ANID|CBF86297.1 BCIN|XP_024552360.1 BGRA|VCU39211.1 CFRU|XP_031892934.1 CGLO|KAF3800785.1 CHIG|XP_018150889.1 CVIN|KAF4922490.1 CVYL|A08766 FGRM|XP_011323785.1 MGRA|XP_003849769.1 MLAR|XP_007410925.1 MORY|QBZ56532.1 NCRA|XP_961937.1 SSCL|APA13241.1

>Orthogroup3293: ANID|CBF86299.1 BCIN|XP_024552361.1 BGRA|VCU38883.1 CFRU|XP_031893593.1 CGLO|KAF3800805.1 CHIG|XP_018150911.1 CVIN|KAF4922501.1 CVYL|A08746 FGRM|XP_011323787.1 MGRA|XP_003849348.1 MLAR|XP_007405066.1 MORY|QBZ56531.1 NCRA|XP_961938.1 SSCL|APA13242.1

>Orthogroup3294: ANID|CBF86313.1 BCIN|XP_024552458.1 BGRA|VDB88058.1 CFRU|XP_031881898.1 CGLO|KAF3800673.1 CHIG|XP_018152009.1 CVIN|KAF4922045.1 CVYL|A13044 FGRM|XP_011321252.1 MGRA|XP_003849488.1 MLAR|XP_007416647.1 MORY|QBZ62111.1 NCRA|XP_011393463.1 SSCL|APA08551.1

>Orthogroup3295: ANID|CBF86317.1 BCIN|XP_024552047.1 BGRA|VDB92712.1 CFRU|XP_031876715.1 CGLO|KAF3798671.1 CHIG|XP_018157483.1 CVIN|KAF4918476.1 CVYL|A09409 FGRM|XP_011328016.1 MGRA|XP_003849815.1 MLAR|XP_007407222.1 MORY|QBZ56202.1 NCRA|XP_956143.2 SSCL|APA12814.1

>Orthogroup3296: ANID|CBF86343.1 BCIN|XP_001551161.1 BGRA|VDB96418.1 CFRU|XP_031878395.1 CGLO|KAF3810757.1 CHIG|XP_018162554.1 CVIN|KAF4932086.1 CVYL|A07197 FGRM|XP_011326319.1 MGRA|XP_003853713.1 MLAR|XP_007411551.1 MORY|QBZ59712.1 NCRA|XP_957017.1 SSCL|APA15073.1

>Orthogroup3298: ANID|CBF86395.1 BCIN|XP_001557348.1 BGRA|VDB89560.1 CFRU|XP_031893511.1 CGLO|KAF3809491.1 CHIG|XP_018152678.1 CVIN|KAF4921299.1 CVYL|A10058 FGRM|XP_011317900.1 MGRA|XP_003849901.1 MLAR|XP_007414749.1 MORY|QBZ55015.1 NCRA|XP_961590.2 SSCL|APA10451.1

>Orthogroup3299: ANID|CBF86416.1 BCIN|XP_001556175.1 BGRA|VCU39007.1 CFRU|XP_031886200.1 CGLO|KAF3806586.1 CHIG|XP_018157625.1 CVIN|KAF4920499.1 CVYL|A09282 FGRM|XP_011328110.1 MGRA|XP_003854573.1 MLAR|XP_007407311.1 MORY|QBZ56231.1 NCRA|XP_962159.3 SSCL|APA15749.1

>Orthogroup3300: ANID|CBF86430.1 BCIN|XP_001561198.1 BGRA|VCU39104.1 CFRU|XP_031887752.1 CGLO|KAF3811627.1 CHIG|XP_018160900.1 CVIN|KAF4921427.1 CVYL|A03487 FGRM|XP_011328161.1 MGRA|XP_003854281.1 MLAR|XP_007412103.1 MORY|QBZ56700.1 NCRA|XP_961981.1 SSCL|APA07084.1

>Orthogroup3301: ANID|CBF86443.1 BCIN|XP_024549148.1 BGRA|VCU39111.1 CFRU|XP_031887572.1 CGLO|KAF3811621.1 CHIG|XP_018160893.1 CVIN|KAF4921469.1 CVYL|A03479 FGRM|XP_011328156.1 MGRA|XP_003854377.1 MLAR|XP_007413667.1 MORY|QBZ56586.1 NCRA|XP_964429.2 SSCL|APA08804.1

>Orthogroup3302: ANID|CBF86449.1 BCIN|XP_001561199.1 BGRA|VCU39105.1 CFRU|XP_031887751.1 CGLO|KAF3811626.1 CHIG|XP_018160899.1 CVIN|KAF4921428.1 CVYL|A03486 FGRM|XP_011328160.1 MGRA|XP_003853853.1 MLAR|XP_007406170.1 MORY|QBZ56582.1 NCRA|XP_011394270.1 SSCL|APA07085.1

>Orthogroup3303: ANID|CBF86458.1 BCIN|XP_024548050.1 BGRA|VDB86188.1 CFRU|XP_031886858.1 CGLO|KAF3811402.1 CHIG|XP_018160638.1 CVIN|KAF4919682.1 CVYL|A04595 FGRM|XP_011327162.1 MGRA|XP_003848853.1 MLAR|XP_007413439.1 MORY|QBZ54282.1 NCRA|XP_955993.1 SSCL|APA08295.1

>Orthogroup3304: ANID|CBF86460.1 BCIN|XP_024547580.1 BGRA|VDB85921.1 CFRU|XP_031886864.1 CGLO|KAF3811400.1 CHIG|XP_018160636.1 CVIN|KAF4919684.1 CVYL|A04593 FGRM|XP_011327160.1 MGRA|XP_003849144.1 MLAR|XP_007416001.1 MORY|QBZ54285.1 NCRA|XP_960597.1 SSCL|APA07834.1

>Orthogroup3305: ANID|CBF86487.1 BCIN|XP_024547614.1 BGRA|VDB86142.1 CFRU|XP_031891209.1 CGLO|KAF3804521.1 CHIG|XP_018159468.1 CVIN|KAF4924497.1 CVYL|A04240 FGRM|XP_011328797.1 MGRA|XP_003850298.1 MLAR|XP_007414135.1 MORY|QBZ58116.1 NCRA|XP_011394470.1 SSCL|APA07790.1

>Orthogroup3306: ANID|CBF86489.1 BCIN|XP_024547613.1 BGRA|VDB86141.1 CFRU|XP_031891206.1 CGLO|KAF3804522.1 CHIG|XP_018159467.1 CVIN|KAF4924523.1 CVYL|A04241 FGRM|XP_011328798.1 MGRA|XP_003850110.1 MLAR|XP_007406679.1 MORY|QBZ58117.1 NCRA|XP_961048.2 SSCL|APA07791.1

>Orthogroup3307: ANID|CBF86491.1 BCIN|XP_001551895.1 BGRA|VDB85933.1 CFRU|XP_031887051.1 CGLO|KAF3811264.1 CHIG|XP_018160485.1 CVIN|KAF4927228.1 CVYL|A04474 FGRM|XP_011326920.1 MGRA|XP_003850409.1 MLAR|XP_007407759.1 MORY|QBZ58099.1 NCRA|XP_960723.1 SSCL|APA15208.1

>Orthogroup3308: ANID|CBF86501.1 BCIN|XP_001547777.1 BGRA|VDB84353.1 CFRU|XP_031886877.1 CGLO|KAF3811236.1 CHIG|XP_018160459.1 CVIN|KAF4931185.1 CVYL|A04429 FGRM|XP_011327084.1 MGRA|XP_003850084.1 MLAR|XP_007406513.1 MORY|QBZ64755.1 NCRA|XP_960729.1 SSCL|APA15420.1

>Orthogroup3310: ANID|CBF86515.1 BCIN|XP_001555047.2 BGRA|VDB85927.1 CFRU|XP_031886451.1 CGLO|KAF3806314.1 CHIG|XP_018162148.1 CVIN|KAF4923936.1 CVYL|A11906 FGRM|XP_011326599.1 MGRA|XP_003850388.1 MLAR|XP_007416799.1 MORY|QBZ61575.1 NCRA|XP_957299.2 SSCL|APA07808.1

>Orthogroup3311: ANID|CBF86520.1 BCIN|XP_001550040.1 BGRA|VCU39954.1 CFRU|XP_031886543.1 CGLO|KAF3804056.1 CHIG|XP_018162695.1 CVIN|KAF4919528.1 CVYL|A07017 FGRM|XP_011327052.1 MGRA|XP_003850303.1 MLAR|XP_007403753.1 MORY|QBZ61427.1 NCRA|XP_957128.2 SSCL|APA10473.1

>Orthogroup3312: ANID|CBF86522.1 BCIN|XP_001555070.1 BGRA|VDB85913.1 CFRU|XP_031887074.1 CGLO|KAF3811396.1 CHIG|XP_018160632.1 CVIN|KAF4919705.1 CVYL|A04589 FGRM|XP_011327189.1 MGRA|XP_003849947.1 MLAR|XP_007403743.1 MORY|QBZ64737.1 NCRA|XP_961017.1 SSCL|APA07824.1

>Orthogroup3313: ANID|CBF86525.1 BCIN|XP_024547593.1 BGRA|VDB85904.1 CFRU|XP_031886433.1 CGLO|KAF3806342.1 CHIG|XP_018162132.1 CVIN|KAF4908674.1 CVYL|A11930 FGRM|XP_011326612.1 MGRA|XP_003850259.1 MLAR|XP_007414896.1 MORY|QBZ61386.1 NCRA|XP_962987.1 SSCL|APA07813.1

>Orthogroup3315: ANID|CBF86533.1 BCIN|XP_024551233.1 BGRA|VDB89557.1 CFRU|XP_031893079.1 CGLO|KAF3809578.1 CHIG|XP_018152604.1 CVIN|KAF4896062.1 CVYL|A08453 FGRM|XP_011317898.1 MGRA|XP_003849911.1 MLAR|XP_007412819.1 MORY|QBZ54095.1 NCRA|XP_011394745.1 SSCL|APA10106.1

>Orthogroup3316: ANID|CBF86539.1 BCIN|XP_024551442.1 BGRA|VDB87646.1 CFRU|XP_031886314.1 CGLO|KAF3806283.1 CHIG|XP_018162111.1 CVIN|KAF4923921.1 CVYL|A11874 FGRM|XP_011324058.1 MGRA|XP_003850315.1 MLAR|XP_007413524.1 MORY|QBZ61376.1 NCRA|XP_956772.2 SSCL|APA10698.1

>Orthogroup3317: ANID|CBF86548.1 BCIN|XP_024551433.1 BGRA|VDB87638.1 CFRU|XP_031886549.1 CGLO|KAF3804054.1 CHIG|XP_018162726.1 CVIN|KAF4919527.1 CVYL|A07015 FGRM|XP_011327061.1 MGRA|XP_003850159.1 MLAR|XP_007417134.1 MORY|QBZ59319.1 NCRA|XP_011393770.1 SSCL|APA10689.1

>Orthogroup3318: ANID|CBF86549.1 BCIN|XP_024549228.1 BGRA|VDB90494.1 CFRU|XP_031890341.1 CGLO|KAF3804881.1 CHIG|XP_018164388.1 CVIN|KAF4921157.1 CVYL|A12182 FGRM|XP_011316263.1 MGRA|XP_003850172.1 MLAR|XP_007410248.1 MORY|QBZ63921.1 NCRA|XP_964034.1 SSCL|APA08985.1

>Orthogroup3319: ANID|CBF86559.1 BCIN|XP_024551217.1 BGRA|VCU39450.1 CFRU|XP_031886808.1 CGLO|KAF3801200.1 CHIG|XP_018162497.1 CVIN|KAF4932104.1 CVYL|A07134 FGRM|XP_011324112.1 MGRA|XP_003850423.1 MLAR|XP_007412069.1 MORY|QBZ56499.1 NCRA|XP_011393987.1 SSCL|APA10132.1

>Orthogroup3320: ANID|CBF86560.1 BCIN|XP_001557361.2 BGRA|VDB87606.1 CFRU|XP_031893538.1 CGLO|KAF3808112.1 CHIG|XP_018152811.1 CVIN|KAF4920609.1 CVYL|A08122 FGRM|XP_011317876.1 MGRA|XP_003849951.1 MLAR|XP_007414676.1 MORY|QBZ54839.1 NCRA|XP_958190.1 SSCL|APA10454.1

>Orthogroup3321: ANID|CBF86581.1 BCIN|XP_001551181.2 BGRA|VCU38788.1 CFRU|XP_031885295.1 CGLO|KAF3806502.1 CHIG|XP_018157539.1 CVIN|KAF4921326.1 CVYL|A09367 FGRM|XP_011328069.1 MGRA|XP_003851230.1 MLAR|XP_007412052.1 MORY|QBZ56352.1 NCRA|XP_961897.2 SSCL|APA13209.1

>Orthogroup3322: ANID|CBF86583.1 BCIN|XP_024552265.1 BGRA|VCU39493.1 CFRU|XP_031887762.1 CGLO|KAF3803269.1 CHIG|XP_018161127.1 CVIN|KAF4928702.1 CVYL|A03703 FGRM|XP_011327955.1 MGRA|XP_003849508.1 MLAR|XP_007416836.1 MORY|QBZ58361.1 NCRA|XP_011394314.1 SSCL|APA13116.1

>Orthogroup3324: ANID|CBF86588.1 BCIN|XP_001557986.2 BGRA|VCU39292.1 CFRU|XP_031893252.1 CGLO|KAF3800829.1 CHIG|XP_018150880.1 CVIN|KAF4922462.1 CVYL|A08720 FGRM|XP_011328049.1 MGRA|XP_003849807.1 MLAR|XP_007410559.1 MORY|QBZ64185.1 NCRA|XP_962253.1 SSCL|APA12688.1

>Orthogroup3325: ANID|CBF86590.1 BCIN|XP_024553280.1 BGRA|VDB93957.1 CFRU|XP_031883325.1 CGLO|KAF3800610.1 CHIG|XP_018152071.1 CVIN|KAF4918172.1 CVYL|A13108 FGRM|XP_011321302.1 MGRA|XP_003855404.1 MLAR|XP_007412218.1 MORY|QBZ61043.1 NCRA|XP_011393580.1 SSCL|APA13556.1

>Orthogroup3326: ANID|CBF86593.1 BCIN|XP_001549546.1 BGRA|VDB93964.1 CFRU|XP_031883324.1 CGLO|KAF3800611.1 CHIG|XP_018152072.1 CVIN|KAF4918173.1 CVYL|A13107 FGRM|XP_011321300.1 MGRA|XP_003848865.1 MLAR|XP_007415150.1 MORY|QBZ61041.1 NCRA|XP_963448.1 SSCL|APA13554.1

>Orthogroup3327: ANID|CBF86614.1 BCIN|XP_024546689.1 BGRA|VDB92959.1 CFRU|XP_031884316.1 CGLO|KAF3799937.1 CHIG|XP_018153066.1 CVIN|KAF4906994.1 CVYL|A14185 FGRM|XP_011323510.1 MGRA|XP_003851549.1 MLAR|XP_007415248.1 MORY|QBZ64026.1 NCRA|XP_964389.1 SSCL|APA06540.1

>Orthogroup3328: ANID|CBF86624.1 BCIN|XP_001548350.1 BGRA|VDB92938.1 CFRU|XP_031882944.1 CGLO|KAF3803381.1 CHIG|XP_018163862.1 CVIN|KAF4918425.1 CVYL|A14308 FGRM|XP_011324170.1 MGRA|XP_003850753.1 MLAR|XP_007412483.1 MORY|QBZ63200.1 NCRA|XP_011392866.1 SSCL|APA06948.1

>Orthogroup3329: ANID|CBF86787.1 BCIN|XP_024554006.1 BGRA|VDB84269.1 CFRU|XP_031880683.1 CGLO|KAF3811190.1 CHIG|XP_018156381.1 CVIN|KAF4931216.1 CVYL|A04354 FGRM|XP_011323732.1 MGRA|XP_003855009.1 MLAR|XP_007414680.1 MORY|QBZ64670.1 NCRA|XP_955989.1 SSCL|APA15389.1

>Orthogroup3331: ANID|CBF86802.1 BCIN|XP_001559069.1 BGRA|VDB90901.1 CFRU|XP_031888578.1 CGLO|KAF3803696.1 CHIG|XP_018163756.1 CVIN|KAF4928572.1 CVYL|A14628 FGRM|XP_011316024.1 MGRA|XP_003852657.1 MLAR|XP_007416168.1 MORY|QBZ54594.1 NCRA|XP_965664.1 SSCL|APA09074.1

>Orthogroup3332: ANID|CBF86804.1 BCIN|XP_024549406.1 BGRA|VDB90902.1 CFRU|XP_031888576.1 CGLO|KAF3803695.1 CHIG|XP_018163757.1 CVIN|KAF4928571.1 CVYL|A14627 FGRM|XP_011316023.1 MGRA|XP_003852441.1 MLAR|XP_007417255.1 MORY|QBZ54593.1 NCRA|XP_011392960.1 SSCL|APA09075.1

>Orthogroup3333: ANID|CBF86805.1 BCIN|XP_024549407.1 BGRA|VDB90904.1 CFRU|XP_031888498.1 CGLO|KAF3803694.1 CHIG|XP_018163758.1 CVIN|KAF4928570.1 CVYL|A14626 FGRM|XP_011316022.1 MGRA|XP_003852370.1 MLAR|XP_007409390.1 MORY|QBZ54591.1 NCRA|XP_965662.2 SSCL|APA09076.1

>Orthogroup3334: ANID|CBF86809.1 BCIN|XP_001558358.1 BGRA|VDB90896.1 CFRU|XP_031882777.1 CGLO|KAF3803363.1 CHIG|XP_018163957.1 CVIN|KAF4915970.1 CVYL|A14298 FGRM|XP_011317149.1 MGRA|XP_003853289.1 MLAR|XP_007410043.1 MORY|QBZ62773.1 NCRA|XP_964678.2 SSCL|APA13761.1

>Orthogroup3335: ANID|CBF86839.1 BCIN|XP_001558154.2 BGRA|VCU39483.1 CFRU|XP_031878723.1 CGLO|KAF3798329.1 CHIG|XP_018151146.1 CVIN|KAF4920312.1 CVYL|A09039 FGRM|XP_011323767.1 MGRA|XP_003849386.1 MLAR|XP_007416236.1 MORY|QBZ56199.1 NCRA|XP_962353.3 SSCL|APA13161.1

>Orthogroup3336: ANID|CBF86847.1 BCIN|XP_001558167.1 BGRA|VDB92771.1 CFRU|XP_031881039.1 CGLO|KAF3800401.1 CHIG|XP_018161404.1 CVIN|KAF4930599.1 CVYL|A01796 FGRM|XP_011321415.1 MGRA|XP_003849599.1 MLAR|XP_007412841.1 MORY|QBZ65290.1 NCRA|XP_962984.2 SSCL|APA13152.1

>Orthogroup3337: ANID|CBF86876.1 BCIN|XP_001557041.1 BGRA|VDB88236.1 CFRU|XP_031879461.1 CGLO|KAF3802397.1 CHIG|XP_018154673.1 CVIN|KAF4922200.1 CVYL|A13540 FGRM|XP_011317289.1 MGRA|XP_003849758.1 MLAR|XP_007410308.1 MORY|QBZ60980.1 NCRA|XP_963439.2 SSCL|APA08498.1

>Orthogroup3338: ANID|CBF86885.1 BCIN|XP_024552512.1 BGRA|VDB92867.1 CFRU|XP_031879482.1 CGLO|KAF3799662.1 CHIG|XP_018154670.1 CVIN|KAF4922168.1 CVYL|A13524 FGRM|XP_011316681.1 MGRA|XP_003849300.1 MLAR|XP_007403978.1 MORY|QBZ60979.1 NCRA|XP_001728061.1 SSCL|APA08483.1

>Orthogroup3339: ANID|CBF86899.1 BCIN|XP_024552622.1 BGRA|VDB95257.1 CFRU|XP_031886003.1 CGLO|KAF3812009.1 CHIG|XP_018155240.1 CVIN|KAF4930437.1 CVYL|A05500 FGRM|XP_011317470.1 MGRA|XP_003849532.1 MLAR|XP_007407389.1 MORY|QBZ53767.1 NCRA|XP_011393554.1 SSCL|APA08718.1

>Orthogroup3341: ANID|CBF86951.1 BCIN|XP_001547457.1 BGRA|VCU38920.1 CFRU|XP_031880589.1 CGLO|KAF3811589.1 CHIG|XP_018160854.1 CVIN|KAF4914875.1 CVYL|A03443 FGRM|XP_011325052.1 MGRA|XP_003854783.1 MLAR|XP_007407212.1 MORY|QBZ58867.1 NCRA|XP_962233.2 SSCL|APA07359.1

>Orthogroup3342: ANID|CBF86960.1 BCIN|XP_024552726.1 BGRA|VDB86340.1 CFRU|XP_031889079.1 CGLO|KAF3808698.1 CHIG|XP_018155562.1 CVIN|KAF4929576.1 CVYL|A00960 FGRM|XP_011321111.1 MGRA|XP_003857428.1 MLAR|XP_007406509.1 MORY|QBZ60108.1 NCRA|XP_011394713.1 SSCL|APA07460.1

>Orthogroup3343: ANID|CBF86964.1 BCIN|XP_024550903.1 BGRA|VDB89787.1 CFRU|XP_031880378.1 CGLO|KAF3807278.1 CHIG|XP_018156954.1 CVIN|KAF4928162.1 CVYL|A10761 FGRM|XP_011320005.1 MGRA|XP_003856239.1 MLAR|XP_007413293.1 MORY|QBZ65969.1 NCRA|XP_965827.2 SSCL|APA11876.1

>Orthogroup3345: ANID|CBF86981.1 BCIN|XP_024551584.1 BGRA|VDB89229.1 CFRU|XP_031890708.1 CGLO|KAF3801588.1 CHIG|XP_018151345.1 CVIN|KAF4922728.1 CVYL|A12547 FGRM|XP_011320341.1 MGRA|XP_003857392.1 MLAR|XP_007406731.1 MORY|QBZ62603.1 NCRA|XP_960224.2 SSCL|APA14873.1

>Orthogroup3346: ANID|CBF86995.1 BCIN|XP_024552937.1 BGRA|VCU41223.1 CFRU|XP_031892709.1 CGLO|KAF3809161.1 CHIG|XP_018155983.1 CVIN|KAF4919814.1 CVYL|A01348 FGRM|XP_011318977.1 MGRA|XP_003856792.1 MLAR|XP_007405083.1 MORY|QBZ62468.1 NCRA|XP_959179.1 SSCL|APA07558.1

>Orthogroup3347: ANID|CBF87000.1 BCIN|XP_001557544.1 BGRA|VCU40755.1 CFRU|XP_031876395.1 CGLO|KAF3797790.1 CHIG|XP_018156471.1 CVIN|KAF4923132.1 CVYL|A03015 FGRM|XP_011318589.1 MGRA|XP_003857575.1 MLAR|XP_007414533.1 MORY|QBZ57791.1 NCRA|XP_960041.3 SSCL|APA12354.1

>Orthogroup3348: ANID|CBF87005.1 BCIN|XP_024552878.1 BGRA|VDB93058.1 CFRU|XP_031882492.1 CGLO|KAF3807631.1 CHIG|XP_018156298.1 CVIN|KAF4921402.1 CVYL|A03119 FGRM|XP_011318964.1 MGRA|XP_003857444.1 MLAR|XP_007410840.1 MORY|QBZ56115.1 NCRA|XP_957863.3 SSCL|APA16105.1

>Orthogroup3349: ANID|CBF87010.1 BCIN|XP_024552938.1 BGRA|VCU41227.1 CFRU|XP_031892708.1 CGLO|KAF3809160.1 CHIG|XP_018155982.1 CVIN|KAF4919813.1 CVYL|A01347 FGRM|XP_011318976.1 MGRA|XP_003856887.1 MLAR|XP_007411717.1 MORY|QBZ62467.1 NCRA|XP_959180.2 SSCL|APA07557.1

>Orthogroup3350: ANID|CBF87030.1 BCIN|XP_001557193.1 BGRA|VCU40915.1 CFRU|XP_031882411.1 CGLO|KAF3809426.1 CHIG|XP_018156267.1 CVIN|KAF4921396.1 CVYL|A03097 FGRM|XP_011319056.1 MGRA|XP_003857641.1 MLAR|XP_007411002.1 MORY|QBZ56659.1 NCRA|XP_961335.1 SSCL|APA12525.1

>Orthogroup3356: ANID|CBF87476.1 BCIN|XP_001550971.1 BGRA|VDB90786.1 CFRU|XP_031888255.1 CGLO|KAF3803494.1 CHIG|XP_018164024.1 CVIN|KAF4926279.1 CVYL|A14423 FGRM|XP_011326517.1 MGRA|XP_003855832.1 MLAR|XP_007406195.1 MORY|QBZ54721.1 NCRA|XP_011393123.1 SSCL|APA07071.1

>Orthogroup3358: ANID|CBF87537.1 BCIN|XP_024549094.1 BGRA|VCU38964.1 CFRU|XP_031887694.1 CGLO|KAF3807142.1 CHIG|XP_018160930.1 CVIN|KAF4920169.1 CVYL|A03540 FGRM|XP_011323740.1 MGRA|XP_003855438.1 MLAR|XP_007409725.1 MORY|QBZ56314.1 NCRA|XP_962536.3 SSCL|APA15911.1

>Orthogroup3359: ANID|CBF87538.1 BCIN|XP_001547274.1 BGRA|VCU38901.1 CFRU|XP_031887746.1 CGLO|KAF3811609.1 CHIG|XP_018160882.1 CVIN|KAF4921447.1 CVYL|A03468 FGRM|XP_011328147.1 MGRA|XP_003855049.1 MLAR|XP_007417003.1 MORY|QBZ56224.1 NCRA|XP_962633.1 SSCL|APA06990.1

>Orthogroup3360: ANID|CBF87544.1 BCIN|XP_001552306.1 BGRA|VDB84179.1 CFRU|XP_031892651.1 CGLO|KAF3797157.1 CHIG|XP_018157106.1 CVIN|KAF4918842.1 CVYL|A10542 FGRM|XP_011315900.1 MGRA|XP_003854679.1 MLAR|XP_007404339.1 MORY|QBZ65923.1 NCRA|XP_965805.1 SSCL|APA11142.1

>Orthogroup3361: ANID|CBF87545.1 BCIN|XP_001552316.1 BGRA|VDB84193.1 CFRU|XP_031891327.1 CGLO|KAF3797637.1 CHIG|XP_018157429.1 CVIN|KAF4893853.1 CVYL|A11040 FGRM|XP_011323032.1 MGRA|XP_003854594.1 MLAR|XP_007412989.1 MORY|QBZ66426.1 NCRA|XP_965390.1 SSCL|APA11152.1

>Orthogroup3362: ANID|CBF87551.1 BCIN|XP_001555575.1 BGRA|VDB83939.1 CFRU|XP_031886011.1 CGLO|KAF3806546.1 CHIG|XP_018157587.1 CVIN|KAF4920484.1 CVYL|A09323 FGRM|XP_011328000.1 MGRA|XP_003853659.1 MLAR|XP_007418328.1 MORY|QBZ56248.1 NCRA|XP_962370.2 SSCL|APA07438.1

>Orthogroup3363: ANID|CBF87556.1 BCIN|XP_024546441.1 BGRA|VCU38849.1 CFRU|XP_031887693.1 CGLO|KAF3807141.1 CHIG|XP_018160931.1 CVIN|KAF4920168.1 CVYL|A03539 FGRM|XP_011323741.1 MGRA|XP_003855524.1 MLAR|XP_007403555.1 MORY|QBZ56315.1 NCRA|XP_962537.1 SSCL|APA07093.1

>Orthogroup3364: ANID|CBF87570.1 BCIN|XP_024552794.1 BGRA|VCU39688.1 CFRU|XP_031883284.1 CGLO|KAF3800523.1 CHIG|XP_018152181.1 CVIN|KAF4919240.1 CVYL|A13192 FGRM|XP_011325486.1 MGRA|XP_003848020.1 MLAR|XP_007409935.1 MORY|QBZ53931.1 NCRA|XP_955917.2 SSCL|APA16050.1

>Orthogroup3365: ANID|CBF87584.1 BCIN|XP_001557748.1 BGRA|VDB93151.1 CFRU|XP_031886141.1 CGLO|KAF3811960.1 CHIG|XP_018155275.1 CVIN|KAF4930336.1 CVYL|A05544 FGRM|XP_011324653.1 MGRA|XP_003848047.1 MLAR|XP_007416476.1 MORY|QBZ54035.1 NCRA|XP_955871.1 SSCL|APA12186.1

>Orthogroup3366: ANID|CBF87590.1 BCIN|XP_001557802.1 BGRA|VCU41337.1 CFRU|XP_031881316.1 CGLO|KAF3802510.1 CHIG|XP_018154815.1 CVIN|KAF4919018.1 CVYL|A02102 FGRM|XP_011325535.1 MGRA|XP_003848113.1 MLAR|XP_007415988.1 MORY|QBZ53785.1 NCRA|XP_956303.1 SSCL|APA12142.1

>Orthogroup3367: ANID|CBF87602.1 BCIN|XP_001550886.2 BGRA|VCU40973.1 CFRU|XP_031880188.1 CGLO|KAF3809042.1 CHIG|XP_018155870.1 CVIN|KAF4923778.1 CVYL|A01616 FGRM|XP_011319084.1 MGRA|XP_003848214.1 MLAR|XP_007409629.1 MORY|QBZ62458.1 NCRA|XP_960962.3 SSCL|APA12406.1

>Orthogroup3368: ANID|CBF87604.1 BCIN|XP_024548162.1 BGRA|VDB84032.1 CFRU|XP_031880187.1 CGLO|KAF3809043.1 CHIG|XP_018155869.1 CVIN|KAF4923777.1 CVYL|A01618 FGRM|XP_011319085.1 MGRA|XP_003848211.1 MLAR|XP_007409613.1 MORY|QBZ62459.1 NCRA|XP_960963.1 SSCL|APA12405.1

>Orthogroup3370: ANID|CBF87610.1 BCIN|XP_001557549.1 BGRA|VDB93233.1 CFRU|XP_031883081.1 CGLO|KAF3800704.1 CHIG|XP_018163258.1 CVIN|KAF4924279.1 CVYL|A00500 FGRM|XP_011320133.1 MGRA|XP_003848219.1 MLAR|XP_007418174.1 MORY|QBZ62136.1 NCRA|XP_964638.1 SSCL|APA12350.1

>Orthogroup3371: ANID|CBF87612.1 BCIN|XP_001550885.1 BGRA|VCU40975.1 CFRU|XP_031889371.1 CGLO|KAF3803154.1 CHIG|XP_018155518.1 CVIN|KAF4928342.1 CVYL|A01040 FGRM|XP_011319236.1 MGRA|XP_003848127.1 MLAR|XP_007405467.1 MORY|QBZ62245.1 NCRA|XP_961078.1 SSCL|APA12407.1

>Orthogroup3372: ANID|CBF87621.1 BCIN|XP_024551576.1 BGRA|VDB92463.1 CFRU|XP_031885647.1 CGLO|KAF3810125.1 CHIG|XP_018157958.1 CVIN|KAF4912146.1 CVYL|A02716 FGRM|XP_011319979.1 MGRA|XP_003856871.1 MLAR|XP_007410014.1 MORY|QBZ62145.1 NCRA|XP_960283.1 SSCL|APA14900.1

>Orthogroup3373: ANID|CBF87625.1 BCIN|XP_001556679.1 BGRA|VDB92666.1 CFRU|XP_031892581.1 CGLO|KAF3803058.1 CHIG|XP_018153550.1 CVIN|KAF4919853.1 CVYL|A00067 FGRM|XP_011316909.1 MGRA|XP_003856516.1 MLAR|XP_007403902.1 MORY|QBZ55422.1 NCRA|XP_964172.3 SSCL|APA14799.1

>Orthogroup3374: ANID|CBF87637.1 BCIN|XP_001558526.1 BGRA|VDB90588.1 CFRU|XP_031882728.1 CGLO|KAF3812068.1 CHIG|XP_018164080.1 CVIN|KAF4923705.1 CVYL|A14244 FGRM|XP_011318759.1 MGRA|XP_003856806.1 MLAR|XP_007409790.1 MORY|QBZ54634.1 NCRA|XP_964498.1 SSCL|APA14678.1

>Orthogroup3375: ANID|CBF87652.1 BCIN|XP_024548087.1 BGRA|VDB93165.1 CFRU|XP_031892860.1 CGLO|KAF3809134.1 CHIG|XP_018155960.1 CVIN|KAF4922121.1 CVYL|A01318 FGRM|XP_011319188.1 MGRA|XP_003847897.1 MLAR|XP_007417942.1 MORY|QBZ56126.1 NCRA|XP_011394659.1 SSCL|APA12747.1

>Orthogroup3376: ANID|CBF87661.1 BCIN|XP_001557734.1 BGRA|VDB93095.1 CFRU|XP_031882464.1 CGLO|KAF3807596.1 CHIG|XP_018156338.1 CVIN|KAF4928887.1 CVYL|A02885 FGRM|XP_011319007.1 MGRA|XP_003848149.1 MLAR|XP_007408679.1 MORY|QBZ54750.1 NCRA|XP_961633.1 SSCL|APA12199.1

>Orthogroup3377: ANID|CBF87671.1 BCIN|XP_001557732.1 BGRA|VDB93098.1 CFRU|XP_031882466.1 CGLO|KAF3807594.1 CHIG|XP_018156340.1 CVIN|KAF4928956.1 CVYL|A02888 FGRM|XP_011319009.1 MGRA|XP_003848034.1 MLAR|XP_007404938.1 MORY|QBZ54748.1 NCRA|XP_961635.2 SSCL|APA12201.1

>Orthogroup3378: ANID|CBF87680.1 BCIN|XP_024548168.1 BGRA|VCU40997.1 CFRU|XP_031889353.1 CGLO|KAF3808810.1 CHIG|XP_018155713.1 CVIN|KAF4908026.1 CVYL|A01267 FGRM|XP_011319262.1 MGRA|XP_003847872.1 MLAR|XP_007408148.1 MORY|QBZ58712.1 NCRA|XP_011394755.1 SSCL|APA12417.1

>Orthogroup3379: ANID|CBF87681.1 BCIN|XP_024552795.1 BGRA|VCU39689.1 CFRU|XP_031883283.1 CGLO|KAF3800522.1 CHIG|XP_018152179.1 CVIN|KAF4919239.1 CVYL|A13193 FGRM|XP_011325487.1 MGRA|XP_003848006.1 MLAR|XP_007414123.1 MORY|QBZ53930.1 NCRA|XP_955918.1 SSCL|APA16049.1

>Orthogroup3380: ANID|CBF87683.1 BCIN|XP_024552782.1 BGRA|VCU39681.1 CFRU|XP_031883282.1 CGLO|KAF3800521.1 CHIG|XP_018152178.1 CVIN|KAF4919234.1 CVYL|A13194 FGRM|XP_011317363.1 MGRA|XP_003848085.1 MLAR|XP_007416957.1 MORY|QBZ53929.1 NCRA|XP_955919.1 SSCL|APA07513.1

>Orthogroup3382: ANID|CBF87697.1 BCIN|XP_001545305.1 BGRA|VDB93155.1 CFRU|XP_031887317.1 CGLO|KAF3809254.1 CHIG|XP_018156087.1 CVIN|KAF4925301.1 CVYL|A01445 FGRM|XP_011318861.1 MGRA|XP_003847959.1 MLAR|XP_007410992.1 MORY|QBZ62176.1 NCRA|XP_956556.1 SSCL|APA12649.1

>Orthogroup3383: ANID|CBF87712.1 BCIN|XP_024548438.1 BGRA|VDB95211.1 CFRU|XP_031887252.1 CGLO|KAF3809284.1 CHIG|XP_018156128.1 CVIN|KAF4921069.1 CVYL|A01478 FGRM|XP_011319143.1 MGRA|XP_003847851.1 MLAR|XP_007404019.1 MORY|QBZ57415.1 NCRA|XP_961069.3 SSCL|APA12269.1

>Orthogroup3385: ANID|CBF87773.1 BCIN|XP_001558447.1 BGRA|VDB95003.1 CFRU|XP_031883838.1 CGLO|KAF3805066.1 CHIG|XP_018163451.1 CVIN|KAF4902236.1 CVYL|A06494 FGRM|XP_011318753.1 MGRA|XP_003857094.1 MLAR|XP_007403782.1 MORY|QBZ54171.1 NCRA|XP_957486.1 SSCL|APA14735.1

>Orthogroup3386: ANID|CBF87798.1 BCIN|XP_001558433.1 BGRA|VDB90456.1 CFRU|XP_031876488.1 CGLO|KAF3797016.1 CHIG|XP_018153666.1 CVIN|KAF4890190.1 CVYL|A00381 FGRM|XP_011317069.1 MGRA|XP_003856618.1 MLAR|XP_007411246.1 MORY|QBZ63869.1 NCRA|XP_001728407.2 SSCL|APA14748.1

>Orthogroup3387: ANID|CBF87801.1 BCIN|XP_001556757.1 BGRA|VDB89484.1 CFRU|XP_031884888.1 CGLO|KAF3800990.1 CHIG|XP_018153239.1 CVIN|KAF4913267.1 CVYL|A05920 FGRM|XP_011318727.1 MGRA|XP_003857006.1 MLAR|XP_007408512.1 MORY|QBZ55249.1 NCRA|XP_965329.1 SSCL|APA14541.1

>Orthogroup3389: ANID|CBF87838.1 BCIN|XP_001556646.2 BGRA|VDB92653.1 CFRU|XP_031879874.1 CGLO|KAF3802957.1 CHIG|XP_018153658.1 CVIN|KAF4929988.1 CVYL|A00389 FGRM|XP_011317084.1 MGRA|XP_003856666.1 MLAR|XP_007417974.1 MORY|QBZ63846.1 NCRA|XP_965101.1 SSCL|APA14777.1

>Orthogroup3390: ANID|CBF87839.1 BCIN|XP_001556647.1 BGRA|VDB92652.1 CFRU|XP_031879875.1 CGLO|KAF3802958.1 CHIG|XP_018153657.1 CVIN|KAF4929942.1 CVYL|A00390 FGRM|XP_011317085.1 MGRA|XP_003857016.1 MLAR|XP_007409666.1 MORY|QBZ63845.1 NCRA|XP_965102.1 SSCL|APA14778.1

>Orthogroup3391: ANID|CBF87853.1 BCIN|XP_001556637.1 BGRA|VDB92655.1 CFRU|XP_031876515.1 CGLO|KAF3802956.1 CHIG|XP_018153659.1 CVIN|KAF4929943.1 CVYL|A00388 FGRM|XP_011317082.1 MGRA|XP_003856212.1 MLAR|XP_007409000.1 MORY|QBZ63842.1 NCRA|XP_965486.1 SSCL|APA14775.1

>Orthogroup3392: ANID|CBF87861.1 BCIN|XP_001548321.1 BGRA|VDB95031.1 CFRU|XP_031884000.1 CGLO|KAF3805140.1 CHIG|XP_018163373.1 CVIN|KAF4927405.1 CVYL|A06416 FGRM|XP_011321666.1 MGRA|XP_003856822.1 MLAR|XP_007414272.1 MORY|QBZ59391.1 NCRA|XP_962430.1 SSCL|APA06367.1

>Orthogroup3393: ANID|CBF87876.1 BCIN|XP_001556627.2 BGRA|VDB92658.1 CFRU|XP_031879786.1 CGLO|KAF3802989.1 CHIG|XP_018153624.1 CVIN|KAF4929922.1 CVYL|A00422 FGRM|XP_011316924.1 MGRA|XP_003856828.1 MLAR|XP_007411945.1 MORY|QBZ55601.1 NCRA|XP_965127.1 SSCL|APA14767.1

>Orthogroup3394: ANID|CBF87892.1 BCIN|XP_001558512.1 BGRA|VDB90604.1 CFRU|XP_031888373.1 CGLO|KAF3803594.1 CHIG|XP_018163871.1 CVIN|KAF4920355.1 CVYL|A14529 FGRM|XP_011316773.1 MGRA|XP_003856927.1 MLAR|XP_007409717.1 MORY|QBZ55383.1 NCRA|XP_965478.1 SSCL|APA14690.1

>Orthogroup3395: ANID|CBF87895.1 BCIN|XP_001556843.1 BGRA|VDB90608.1 CFRU|XP_031888371.1 CGLO|KAF3803596.1 CHIG|XP_018163869.1 CVIN|KAF4920353.1 CVYL|A14531 FGRM|XP_011316775.1 MGRA|XP_003856753.1 MLAR|XP_007412568.1 MORY|QBZ55385.1 NCRA|XP_001728409.1 SSCL|APA14808.1

>Orthogroup3398: ANID|CBF87904.1 BCIN|XP_001556887.1 BGRA|VDB89302.1 CFRU|XP_031892327.1 CGLO|KAF3812273.1 CHIG|XP_018153496.1 CVIN|KAF4919928.1 CVYL|A00110 FGRM|XP_011316086.1 MGRA|XP_003851515.1 MLAR|XP_007409872.1 MORY|QBZ62940.1 NCRA|XP_965431.2 SSCL|APA14619.1

>Orthogroup3399: ANID|CBF87906.1 BCIN|XP_001556886.1 BGRA|VDB89301.1 CFRU|XP_031892324.1 CGLO|KAF3812274.1 CHIG|XP_018153497.1 CVIN|KAF4919930.1 CVYL|A00109 FGRM|XP_011316087.1 MGRA|XP_003851413.1 MLAR|XP_007415452.1 MORY|QBZ62939.1 NCRA|XP_011392996.1 SSCL|APA14618.1

>Orthogroup3400: ANID|CBF87952.1 BCIN|XP_001558431.1 BGRA|VDB90475.1 CFRU|XP_031879862.1 CGLO|KAF3802997.1 CHIG|XP_018153615.1 CVIN|KAF4929929.1 CVYL|A00008 FGRM|XP_011316919.1 MGRA|XP_003856638.1 MLAR|XP_007418521.1 MORY|QBZ55596.1 NCRA|XP_965112.2 SSCL|APA14750.1

>Orthogroup3401: ANID|CBF87957.1 BCIN|XP_024551555.1 BGRA|VDB92633.1 CFRU|XP_031890450.1 CGLO|KAF3804960.1 CHIG|XP_018164466.1 CVIN|KAF4910218.1 CVYL|A12094 FGRM|XP_011317115.1 MGRA|XP_003857040.1 MLAR|XP_007406558.1 MORY|QBZ61475.1 NCRA|XP_958622.2 SSCL|APA14922.1

>Orthogroup3403: ANID|CBF87987.1 BCIN|XP_024551768.1 BGRA|VDB89296.1 CFRU|XP_031879834.1 CGLO|KAF3802998.1 CHIG|XP_018153614.1 CVIN|KAF4929978.1 CVYL|A00009 FGRM|XP_011316074.1 MGRA|XP_003856959.1 MLAR|XP_007410166.1 MORY|QBZ63178.1 NCRA|XP_964164.1 SSCL|APA14621.1

>Orthogroup3404: ANID|CBF87989.1 BCIN|XP_001550410.1 BGRA|VDB89424.1 CFRU|XP_031884076.1 CGLO|KAF3805069.1 CHIG|XP_018163448.1 CVIN|KAF4902237.1 CVYL|A06491 FGRM|XP_011318750.1 MGRA|XP_003857054.1 MLAR|XP_007406844.1 MORY|QBZ54174.1 NCRA|XP_957483.3 SSCL|APA06022.1

>Orthogroup3405: ANID|CBF87991.1 BCIN|XP_024551693.1 BGRA|VDB89422.1 CFRU|XP_031883839.1 CGLO|KAF3805070.1 CHIG|XP_018163447.1 CVIN|KAF4927437.1 CVYL|A06490 FGRM|XP_011318749.1 MGRA|XP_003857053.1 MLAR|XP_007406858.1 MORY|QBZ54176.1 NCRA|XP_957482.2 SSCL|APA06023.1

>Orthogroup3406: ANID|CBF87992.1 BCIN|XP_024551694.1 BGRA|VDB89420.1 CFRU|XP_031883920.1 CGLO|KAF3805071.1 CHIG|XP_018163446.1 CVIN|KAF4927436.1 CVYL|A06489 FGRM|XP_011318748.1 MGRA|XP_003856626.1 MLAR|XP_007413048.1 MORY|QBZ54177.1 NCRA|XP_957481.1 SSCL|APA06024.1

>Orthogroup3407: ANID|CBF87996.1 BCIN|XP_001550415.1 BGRA|VDB89413.1 CFRU|XP_031883936.1 CGLO|KAF3805118.1 CHIG|XP_018163399.1 CVIN|KAF4927449.1 CVYL|A06439 FGRM|XP_011321614.1 MGRA|XP_003856744.1 MLAR|XP_007412489.1 MORY|QBZ59495.1 NCRA|XP_962458.1 SSCL|APA06026.1

>Orthogroup3408: ANID|CBF88003.1 BCIN|XP_001559090.2 BGRA|VDB89201.1 CFRU|XP_031890958.1 CGLO|KAF3801680.1 CHIG|XP_018151459.1 CVIN|KAF4896996.1 CVYL|A12456 FGRM|XP_011328493.1 MGRA|XP_003856773.1 MLAR|XP_007408107.1 MORY|QBZ56044.1 NCRA|XP_960280.3 SSCL|APA09063.1

>Orthogroup3409: ANID|CBF88013.1 BCIN|XP_001549136.1 BGRA|VDB95022.1 CFRU|XP_031888575.1 CGLO|KAF3803590.1 CHIG|XP_018163875.1 CVIN|KAF4920375.1 CVYL|A14525 FGRM|XP_011316769.1 MGRA|XP_003857135.1 MLAR|XP_007415387.1 MORY|QBZ55373.1 NCRA|XP_965480.2 SSCL|APA06330.1

>Orthogroup3410: ANID|CBF88017.1 BCIN|XP_024547652.1 BGRA|VCU38786.1 CFRU|XP_031877415.1 CGLO|KAF3805320.1 CHIG|XP_018162031.1 CVIN|KAF4931510.1 CVYL|A11737 FGRM|XP_011324106.1 MGRA|XP_003856475.1 MLAR|XP_007408941.1 MORY|QBZ62965.1 NCRA|XP_957772.1 SSCL|APA07745.1

>Orthogroup3411: ANID|CBF88019.1 BCIN|XP_001558489.2 BGRA|VDB90582.1 CFRU|XP_031888574.1 CGLO|KAF3803589.1 CHIG|XP_018163876.1 CVIN|KAF4920374.1 CVYL|A14524 FGRM|XP_011316768.1 MGRA|XP_003856498.1 MLAR|XP_007405903.1 MORY|QBZ55366.1 NCRA|XP_964055.1 SSCL|APA14704.1

>Orthogroup3412: ANID|CBF88028.1 BCIN|XP_024553948.1 BGRA|VCU38805.1 CFRU|XP_031891528.1 CGLO|KAF3804550.1 CHIG|XP_018160386.1 CVIN|KAF4924534.1 CVYL|A04268 FGRM|XP_011326696.1 MGRA|XP_003856443.1 MLAR|XP_007406327.1 MORY|QBZ56908.1 NCRA|XP_961563.2 SSCL|APA15448.1

>Orthogroup3413: ANID|CBF88036.1 BCIN|XP_024551830.1 BGRA|VDB92451.1 CFRU|XP_031888571.1 CGLO|KAF3803587.1 CHIG|XP_018163878.1 CVIN|KAF4920379.1 CVYL|A14522 FGRM|XP_011316766.1 MGRA|XP_003857112.1 MLAR|XP_007414390.1 MORY|QBZ55368.1 NCRA|XP_965242.2 SSCL|APA14722.1

>Orthogroup3414: ANID|CBF88046.1 BCIN|XP_001557196.1 BGRA|VDB88849.1 CFRU|XP_031890736.1 CGLO|KAF3801541.1 CHIG|XP_018151954.1 CVIN|KAF4921101.1 CVYL|A12595 FGRM|XP_011319373.1 MGRA|XP_003857496.1 MLAR|XP_007409223.1 MORY|QBZ53669.1 NCRA|XP_963288.1 SSCL|APA12713.1

>Orthogroup3416: ANID|CBF88106.1 BCIN|XP_024551808.1 BGRA|VDB90590.1 CFRU|XP_031877877.1 CGLO|KAF3806626.1 CHIG|XP_018164238.1 CVIN|KAF4918921.1 CVYL|A12338 FGRM|XP_011316217.1 MGRA|XP_003856879.1 MLAR|XP_007414185.1 MORY|QBZ54629.1 NCRA|XP_964496.1 SSCL|APA14681.1

>Orthogroup3419: ANID|CBF88153.1 BCIN|XP_001554001.2 BGRA|VDB94930.1 CFRU|XP_031891168.1 CGLO|KAF3804569.1 CHIG|XP_018161647.1 CVIN|KAF4923056.1 CVYL|A05308 FGRM|XP_011318691.1 MGRA|XP_003855639.1 MLAR|XP_007403881.1 MORY|QBZ59235.1 NCRA|XP_962912.2 SSCL|APA11101.1

>Orthogroup3420: ANID|CBF88155.1 BCIN|XP_001552546.1 BGRA|VDB94908.1 CFRU|XP_031891482.1 CGLO|KAF3797892.1 CHIG|XP_018161630.1 CVIN|KAF4923068.1 CVYL|A05329 FGRM|XP_011318671.1 MGRA|XP_003854874.1 MLAR|XP_007404979.1 MORY|QBZ61139.1 NCRA|XP_962900.1 SSCL|APA10806.1

>Orthogroup3421: ANID|CBF88157.1 BCIN|XP_001553952.1 BGRA|VDB94851.1 CFRU|XP_031891568.1 CGLO|KAF3804684.1 CHIG|XP_018161758.1 CVIN|KAF4898357.1 CVYL|A05199 FGRM|XP_011321506.1 MGRA|XP_003855640.1 MLAR|XP_007410304.1 MORY|QBZ61567.1 NCRA|XP_959008.1 SSCL|APA11078.1

>Orthogroup3422: ANID|CBF88161.1 BCIN|XP_024550534.1 BGRA|VDB94585.1 CFRU|XP_031887043.1 CGLO|KAF3811445.1 CHIG|XP_018160688.1 CVIN|KAF4917873.1 CVYL|A04646 FGRM|XP_011324778.1 MGRA|XP_003855415.1 MLAR|XP_007403960.1 MORY|QBZ54567.1 NCRA|XP_960773.3 SSCL|APA11316.1

>Orthogroup3423: ANID|CBF88164.1 BCIN|XP_024550303.1 BGRA|VDB94750.1 CFRU|XP_031877191.1 CGLO|KAF3806435.1 CHIG|XP_018162321.1 CVIN|KAF4918053.1 CVYL|A12031 FGRM|XP_011323952.1 MGRA|XP_003855373.1 MLAR|XP_007403733.1 MORY|QBZ61412.1 NCRA|XP_962723.2 SSCL|APA10966.1

>Orthogroup3426: ANID|CBF88179.1 BCIN|XP_024547913.1 BGRA|VDB94818.1 CFRU|XP_031887091.1 CGLO|KAF3811336.1 CHIG|XP_018160562.1 CVIN|KAF4927215.1 CVYL|A04517 FGRM|XP_011326904.1 MGRA|XP_003855114.1 MLAR|XP_007415648.1 MORY|QBZ57006.1 NCRA|XP_961513.1 SSCL|APA08233.1

>Orthogroup3427: ANID|CBF88182.1 BCIN|XP_001555479.2 BGRA|VDB94868.1 CFRU|XP_031886947.1 CGLO|KAF3811349.1 CHIG|XP_018160589.1 CVIN|KAF4921002.1 CVYL|A04532 FGRM|XP_011323643.1 MGRA|XP_003848784.1 MLAR|XP_007404051.1 MORY|QBZ56984.1 NCRA|XP_961489.1 SSCL|APA08188.1

>Orthogroup3428: ANID|CBF88215.1 BCIN|XP_024550406.1 BGRA|VDB94925.1 CFRU|XP_031891171.1 CGLO|KAF3804567.1 CHIG|XP_018161645.1 CVIN|KAF4923102.1 CVYL|A05310 FGRM|XP_011318690.1 MGRA|XP_003855123.1 MLAR|XP_007414874.1 MORY|QBZ61120.1 NCRA|XP_962906.3 SSCL|APA11103.1

>Orthogroup3430: ANID|CBF88229.1 BCIN|XP_001553502.1 BGRA|VDB84445.1 CFRU|XP_031881613.1 CGLO|KAF3807405.1 CHIG|XP_018156817.1 CVIN|KAF4929599.1 CVYL|A10906 FGRM|XP_011328169.1 MGRA|XP_003848856.1 MLAR|XP_007408797.1 MORY|QBZ65599.1 NCRA|XP_965066.1 SSCL|APA10865.1

>Orthogroup3431: ANID|CBF88254.1 BCIN|XP_001558859.1 BGRA|VDB93694.1 CFRU|XP_031885354.1 CGLO|KAF3799451.1 CHIG|XP_018154438.1 CVIN|KAF4931902.1 CVYL|A13313 FGRM|XP_011316564.1 MGRA|XP_003849067.1 MLAR|XP_007413077.1 MORY|QBZ54388.1 NCRA|XP_957194.2 SSCL|APA06245.1

>Orthogroup3432: ANID|CBF88257.1 BCIN|XP_024546890.1 BGRA|VDB93917.1 CFRU|XP_031885928.1 CGLO|KAF3805523.1 CHIG|XP_018155376.1 CVIN|KAF4918310.1 CVYL|A05645 FGRM|XP_011317527.1 MGRA|XP_003855483.1 MLAR|XP_007417281.1 MORY|QBZ53805.1 NCRA|XP_963623.1 SSCL|APA05846.1

>Orthogroup3433: ANID|CBF88281.1 BCIN|XP_001558315.2 BGRA|VDB93913.1 CFRU|XP_031883238.1 CGLO|KAF3800568.1 CHIG|XP_018152114.1 CVIN|KAF4927070.1 CVYL|A13147 FGRM|XP_011317248.1 MGRA|XP_003855343.1 MLAR|XP_007413409.1 MORY|QBZ60322.1 NCRA|XP_011393490.1 SSCL|APA13695.1

>Orthogroup3434: ANID|CBF88312.1 BCIN|XP_001551485.1 BGRA|VDB83701.1 CFRU|XP_031885421.1 CGLO|KAF3806489.1 CHIG|XP_018157524.1 CVIN|KAF4919465.1 CVYL|A09380 FGRM|XP_011324834.1 MGRA|XP_003848891.1 MLAR|XP_007414919.1 MORY|QBZ64521.1 NCRA|XP_011394257.1 SSCL|APA06198.1

>Orthogroup3435: ANID|CBF88318.1 BCIN|XP_001547729.1 BGRA|VDB83646.1 CFRU|XP_031880513.1 CGLO|KAF3811531.1 CHIG|XP_018160789.1 CVIN|KAF4926776.1 CVYL|A04740 FGRM|XP_011323559.1 MGRA|XP_003855024.1 MLAR|XP_007412138.1 MORY|QBZ58766.1 NCRA|XP_962201.1 SSCL|APA05376.1

>Orthogroup3437: ANID|CBF88325.1 BCIN|XP_024553378.1 BGRA|VDB83652.1 CFRU|XP_031880544.1 CGLO|KAF3811569.1 CHIG|XP_018160826.1 CVIN|KAF4898061.1 CVYL|A04781 FGRM|XP_011328093.1 MGRA|XP_003848933.1 MLAR|XP_007403984.1 MORY|QBZ56678.1 NCRA|XP_011394219.1 SSCL|APA13720.1

>Orthogroup3442: ANID|CBF88354.1 BCIN|XP_001546484.1 BGRA|VDB83628.1 CFRU|XP_031880609.1 CGLO|KAF3811519.1 CHIG|XP_018160776.1 CVIN|KAF4926738.1 CVYL|A04728 FGRM|XP_011327898.1 MGRA|XP_003849004.1 MLAR|XP_007417931.1 MORY|QBZ57237.1 NCRA|XP_961918.2 SSCL|APA08406.1

>Orthogroup3443: ANID|CBF88356.1 BCIN|XP_001554148.1 BGRA|VDB94482.1 CFRU|XP_031892983.1 CGLO|KAF3808355.1 CHIG|XP_018157514.1 CVIN|KAF4909495.1 CVYL|A09470 FGRM|XP_011328131.1 MGRA|XP_003848880.1 MLAR|XP_007412224.1 MORY|QBZ56551.1 NCRA|XP_962283.1 SSCL|APA05511.1

>Orthogroup3445: ANID|CBF88387.1 BCIN|XP_024551401.1 BGRA|VCU41044.1 CFRU|XP_031885219.1 CGLO|KAF3807691.1 CHIG|XP_018162917.1 CVIN|KAF4927832.1 CVYL|A06884 FGRM|XP_011326261.1 MGRA|XP_003855592.1 MLAR|XP_007411198.1 MORY|QBZ59696.1 NCRA|XP_957701.3 SSCL|APA10638.1

>Orthogroup3447: ANID|CBF88408.1 BCIN|XP_001560702.1 BGRA|VDB90346.1 CFRU|XP_031886443.1 CGLO|KAF3811888.1 CHIG|XP_018155172.1 CVIN|KAF4920583.1 CVYL|A06181 FGRM|XP_011317391.1 MGRA|XP_003850668.1 MLAR|XP_007418796.1 MORY|QBZ53761.1 NCRA|XP_955772.2 SSCL|APA07985.1

>Orthogroup3448: ANID|CBF88446.1 BCIN|XP_024551317.1 BGRA|VCU39458.1 CFRU|XP_031886696.1 CGLO|KAF3804059.1 CHIG|XP_018162702.1 CVIN|KAF4919557.1 CVYL|A07021 FGRM|XP_011326945.1 MGRA|XP_003849208.1 MLAR|XP_007415781.1 MORY|QBZ55692.1 NCRA|XP_956375.3 SSCL|APA10558.1

>Orthogroup3449: ANID|CBF88452.1 BCIN|XP_024547475.1 BGRA|VDB90356.1 CFRU|XP_031881283.1 CGLO|KAF3802529.1 CHIG|XP_018154833.1 CVIN|KAF4921849.1 CVYL|A02129 FGRM|XP_011324643.1 MGRA|XP_003855648.1 MLAR|XP_007404843.1 MORY|QBZ53953.1 NCRA|XP_963204.1 SSCL|APA07646.1

>Orthogroup3450: ANID|CBF88470.1 BCIN|XP_001559810.1 BGRA|VDB93283.1 CFRU|XP_031890334.1 CGLO|KAF3806704.1 CHIG|XP_018158678.1 CVIN|KAF4925994.1 CVYL|A14010 FGRM|XP_011315940.1 MGRA|XP_003852949.1 MLAR|XP_007411800.1 MORY|QBZ55745.1 NCRA|XP_964593.1 SSCL|APA09687.1

>Orthogroup3452: ANID|CBF88477.1 BCIN|XP_001559522.1 BGRA|VDB83926.1 CFRU|XP_031890241.1 CGLO|KAF3802879.1 CHIG|XP_018158479.1 CVIN|KAF4923846.1 CVYL|A05134 FGRM|XP_011316604.1 MGRA|XP_003857556.1 MLAR|XP_007412068.1 MORY|QBZ55408.1 NCRA|XP_964745.1 SSCL|APA09523.1

>Orthogroup3453: ANID|CBF88504.1 BCIN|XP_001553218.1 BGRA|VDB89711.1 CFRU|XP_031881680.1 CGLO|KAF3805376.1 CHIG|XP_018156733.1 CVIN|KAF4890837.1 CVYL|A11024 FGRM|XP_011320048.1 MGRA|XP_003848847.1 MLAR|XP_007414603.1 MORY|QBZ65426.1 NCRA|XP_965729.1 SSCL|APA12073.1

>Orthogroup3456: ANID|CBF88511.1 BCIN|XP_024551363.1 BGRA|VDB89096.1 CFRU|XP_031893096.1 CGLO|KAF3808358.1 CHIG|XP_018152420.1 CVIN|KAF4918625.1 CVYL|A09516 FGRM|XP_011317700.1 MGRA|XP_003848943.1 MLAR|XP_007407166.1 MORY|QBZ54482.1 NCRA|XP_961466.1 SSCL|APA10482.1

>Orthogroup3457: ANID|CBF88513.1 BCIN|XP_024552874.1 BGRA|VCU39621.1 CFRU|XP_031889471.1 CGLO|KAF3808838.1 CHIG|XP_018155737.1 CVIN|KAF4909983.1 CVYL|A01243 FGRM|XP_011318921.1 MGRA|XP_003855161.1 MLAR|XP_007403888.1 MORY|QBZ53858.1 NCRA|XP_961416.1 SSCL|APA16110.1

>Orthogroup3458: ANID|CBF88528.1 BCIN|XP_024547710.1 BGRA|VDB94646.1 CFRU|XP_031883327.1 CGLO|KAF3800514.1 CHIG|XP_018152288.1 CVIN|KAF4919228.1 CVYL|A13200 FGRM|XP_011323668.1 MGRA|XP_003855280.1 MLAR|XP_007418381.1 MORY|QBZ64834.1 NCRA|XP_955879.1 SSCL|APA07935.1

>Orthogroup3463: ANID|CBF88666.1 BCIN|XP_001548883.1 BGRA|VDB90353.1 CFRU|XP_031881152.1 CGLO|KAF3802516.1 CHIG|XP_018154821.1 CVIN|KAF4918993.1 CVYL|A02108 FGRM|XP_011325576.1 MGRA|XP_003855039.1 MLAR|XP_007413520.1 MORY|QBZ53947.1 NCRA|XP_963209.1 SSCL|APA15992.1

>Orthogroup3464: ANID|CBF88672.1 BCIN|XP_024547463.1 BGRA|VDB94789.1 CFRU|XP_031886211.1 CGLO|KAF3801150.1 CHIG|XP_018155214.1 CVIN|KAF4904484.1 CVYL|A05695 FGRM|XP_011317410.1 MGRA|XP_003855643.1 MLAR|XP_007414455.1 MORY|QBZ60896.1 NCRA|XP_963212.2 SSCL|APA07633.1

>Orthogroup3465: ANID|CBF88674.1 BCIN|XP_001559487.1 BGRA|VDB90354.1 CFRU|XP_031881151.1 CGLO|KAF3802512.1 CHIG|XP_018154817.1 CVIN|KAF4919016.1 CVYL|A02104 FGRM|XP_011324638.1 MGRA|XP_003855038.1 MLAR|XP_007410876.1 MORY|QBZ53958.1 NCRA|XP_963230.2 SSCL|APA07632.1

>Orthogroup3466: ANID|CBF88694.1 BCIN|XP_001557493.1 BGRA|VCU39524.1 CFRU|XP_031885259.1 CGLO|KAF3807686.1 CHIG|XP_018162912.1 CVIN|KAF4927839.1 CVYL|A06879 FGRM|XP_011326268.1 MGRA|XP_003855162.1 MLAR|XP_007417741.1 MORY|QBZ59708.1 NCRA|XP_956682.1 SSCL|APA10620.1

>Orthogroup3467: ANID|CBF88699.1 BCIN|XP_001554185.1 BGRA|VCU41023.1 CFRU|XP_031893447.1 CGLO|KAF3808354.1 CHIG|XP_018152413.1 CVIN|KAF4918640.1 CVYL|A09514 FGRM|XP_011317698.1 MGRA|XP_003848944.1 MLAR|XP_007414547.1 MORY|QBZ54485.1 NCRA|XP_961454.2 SSCL|APA10177.1

>Orthogroup3468: ANID|CBF88706.1 BCIN|XP_001548085.1 BGRA|VDB91347.1 CFRU|XP_031889958.1 CGLO|KAF3802899.1 CHIG|XP_018158490.1 CVIN|KAF4907432.1 CVYL|A05114 FGRM|XP_011316403.1 MGRA|XP_003852952.1 MLAR|XP_007412948.1 MORY|QBZ55933.1 NCRA|XP_011392849.1 SSCL|APA09515.1

>Orthogroup3470: ANID|CBF88723.1 BCIN|XP_001560094.1 BGRA|VDB93293.1 CFRU|XP_031889993.1 CGLO|KAF3802915.1 CHIG|XP_018158518.1 CVIN|KAF4925560.1 CVYL|A05102 FGRM|XP_011316360.1 MGRA|XP_003857517.1 MLAR|XP_007411447.1 MORY|QBZ55947.1 NCRA|XP_965022.1 SSCL|APA09891.1

>Orthogroup3472: ANID|CBF88730.1 BCIN|XP_001552059.1 BGRA|VDB83917.1 CFRU|XP_031889986.1 CGLO|KAF3802934.1 CHIG|XP_018158537.1 CVIN|KAF4925591.1 CVYL|A05084 FGRM|XP_011316353.1 MGRA|XP_003852913.1 MLAR|XP_007410756.1 MORY|QBZ55988.1 NCRA|XP_965019.2 SSCL|APA09998.1

>Orthogroup3473: ANID|CBF88741.1 BCIN|XP_001549037.1 BGRA|VDB84028.1 CFRU|XP_031890544.1 CGLO|KAF3806693.1 CHIG|XP_018158662.1 CVIN|KAF4925986.1 CVYL|A13996 FGRM|XP_011316651.1 MGRA|XP_003852940.1 MLAR|XP_007418175.1 MORY|QBZ55480.1 NCRA|XP_964097.2 SSCL|APA09846.1

>Orthogroup3474: ANID|CBF88754.1 BCIN|XP_001548614.2 BGRA|VDB93290.1 CFRU|XP_031890438.1 CGLO|KAF3805311.1 CHIG|XP_018158629.1 CVIN|KAF4926033.1 CVYL|A13964 FGRM|XP_011316593.1 MGRA|XP_003852942.1 MLAR|XP_007411956.1 MORY|QBZ56079.1 NCRA|XP_964186.3 SSCL|APA09659.1

>Orthogroup3475: ANID|CBF88763.1 BCIN|XP_001559723.1 BGRA|VDB83972.1 CFRU|XP_031890617.1 CGLO|KAF3805309.1 CHIG|XP_018158627.1 CVIN|KAF4926009.1 CVYL|A13962 FGRM|XP_011316592.1 MGRA|XP_003853594.1 MLAR|XP_007412818.1 MORY|QBZ56077.1 NCRA|XP_964188.1 SSCL|APA09464.1

>Orthogroup3476: ANID|CBF88765.1 BCIN|XP_001549045.1 BGRA|VDB84091.1 CFRU|XP_031890430.1 CGLO|KAF3798134.1 CHIG|XP_018158637.1 CVIN|KAF4925981.1 CVYL|A13972 FGRM|XP_011323400.1 MGRA|XP_003854497.1 MLAR|XP_007419060.1 MORY|QBZ56084.1 NCRA|XP_964182.1 SSCL|APA09852.1

>Orthogroup3477: ANID|CBF88768.1 BCIN|XP_001559724.1 BGRA|VDB83968.1 CFRU|XP_031890437.1 CGLO|KAF3805310.1 CHIG|XP_018158628.1 CVIN|KAF4926010.1 CVYL|A13963 FGRM|XP_011316591.1 MGRA|XP_003854498.1 MLAR|XP_007404351.1 MORY|QBZ56078.1 NCRA|XP_964187.1 SSCL|APA09465.1

>Orthogroup3479: ANID|CBF88804.1 BCIN|XP_024551136.1 BGRA|VDB89542.1 CFRU|XP_031887867.1 CGLO|KAF3802296.1 CHIG|XP_018154398.1 CVIN|KAF4924414.1 CVYL|A10629 FGRM|XP_011319770.1 MGRA|XP_003855605.1 MLAR|XP_007418512.1 MORY|QBZ65773.1 NCRA|XP_011392901.1 SSCL|APA12082.1

>Orthogroup3480: ANID|CBF88834.1 BCIN|XP_001550035.1 BGRA|VCU41021.1 CFRU|XP_031893140.1 CGLO|KAF3797483.1 CHIG|XP_018152384.1 CVIN|KAF4898429.1 CVYL|A09486 FGRM|XP_011317724.1 MGRA|XP_003854940.1 MLAR|XP_007411353.1 MORY|QBZ54048.1 NCRA|XP_961281.3 SSCL|APA10477.1

>Orthogroup3481: ANID|CBF88852.1 BCIN|XP_024549520.1 BGRA|VCU40083.1 CFRU|XP_031887725.1 CGLO|KAF3807153.1 CHIG|XP_018160976.1 CVIN|KAF4920184.1 CVYL|A03555 FGRM|XP_011327945.1 MGRA|XP_003851903.1 MLAR|XP_007410686.1 MORY|QBZ58809.1 NCRA|XP_962075.1 SSCL|APA09116.1

>Orthogroup3482: ANID|CBF88896.1 BCIN|XP_024547083.1 BGRA|VDB83612.1 CFRU|XP_031877148.1 CGLO|KAF3803973.1 CHIG|XP_018150970.1 CVIN|KAF4925149.1 CVYL|A08828 FGRM|XP_011323754.1 MGRA|XP_003851876.1 MLAR|XP_007417511.1 MORY|QBZ61676.1 NCRA|XP_955912.1 SSCL|APA06165.1

>Orthogroup3483: ANID|CBF88900.1 BCIN|XP_024552644.1 BGRA|VDB88073.1 CFRU|XP_031882562.1 CGLO|KAF3800476.1 CHIG|XP_018152219.1 CVIN|KAF4906664.1 CVYL|A13235 FGRM|XP_011321325.1 MGRA|XP_003851676.1 MLAR|XP_007408168.1 MORY|QBZ62011.1 NCRA|XP_956005.1 SSCL|APA15591.1

>Orthogroup3484: ANID|CBF88902.1 BCIN|XP_024552645.1 BGRA|VDB88074.1 CFRU|XP_031883387.1 CGLO|KAF3800509.1 CHIG|XP_018152215.1 CVIN|KAF4919222.1 CVYL|A13205 FGRM|XP_011321321.1 MGRA|XP_003851687.1 MLAR|XP_007411841.1 MORY|QBZ61108.1 NCRA|XP_956536.3 SSCL|APA15590.1

>Orthogroup3485: ANID|CBF88905.1 BCIN|XP_001545705.1 BGRA|VDB95213.1 CFRU|XP_031887592.1 CGLO|KAF3811640.1 CHIG|XP_018160915.1 CVIN|KAF4921461.1 CVYL|A03501 FGRM|XP_011323584.1 MGRA|XP_003851585.1 MLAR|XP_007409510.1 MORY|QBZ56598.1 NCRA|XP_962663.2 SSCL|APA06206.1

>Orthogroup3486: ANID|CBF88927.1 BCIN|XP_024553539.1 BGRA|VDB83687.1 CFRU|XP_031877108.1 CGLO|KAF3803962.1 CHIG|XP_018150982.1 CVIN|KAF4925159.1 CVYL|A08840 FGRM|XP_011323416.1 MGRA|XP_003857546.1 MLAR|XP_007407443.1 MORY|QBZ59212.1 NCRA|XP_957506.1 SSCL|APA13717.1

>Orthogroup3487: ANID|CBF88931.1 BCIN|XP_001546341.1 BGRA|VCU39349.1 CFRU|XP_031879031.1 CGLO|KAF3798778.1 CHIG|XP_018151237.1 CVIN|KAF4912770.1 CVYL|A09145 FGRM|XP_011328079.1 MGRA|XP_003856052.1 MLAR|XP_007414223.1 MORY|QBZ55238.1 NCRA|XP_962119.1 SSCL|APA05406.1

>Orthogroup3488: ANID|CBF88948.1 BCIN|XP_024552651.1 BGRA|VDB88343.1 CFRU|XP_031882665.1 CGLO|KAF3796939.1 CHIG|XP_018152225.1 CVIN|KAF4901179.1 CVYL|A13242 FGRM|XP_011325696.1 MGRA|XP_003856050.1 MLAR|XP_007410154.1 MORY|QBZ62048.1 NCRA|XP_956266.1 SSCL|APA15579.1

>Orthogroup3489: ANID|CBF88968.1 BCIN|XP_001545578.1 BGRA|VDB89086.1 CFRU|XP_031889355.1 CGLO|KAF3808812.1 CHIG|XP_018155715.1 CVIN|KAF4908017.1 CVYL|A01266 FGRM|XP_011319264.1 MGRA|XP_003856415.1 MLAR|XP_007406436.1 MORY|QBZ58710.1 NCRA|XP_960675.1 SSCL|APA16299.1

>Orthogroup3490: ANID|CBF88984.1 BCIN|XP_001545580.1 BGRA|VDB89084.1 CFRU|XP_031889455.1 CGLO|KAF3808814.1 CHIG|XP_018155717.1 CVIN|KAF4908029.1 CVYL|A01264 FGRM|XP_011319266.1 MGRA|XP_003857393.1 MLAR|XP_007403726.1 MORY|QBZ58715.1 NCRA|XP_960813.3 SSCL|APA16301.1

>Orthogroup3491: ANID|CBF89002.1 BCIN|XP_001556217.1 BGRA|VDB90878.1 CFRU|XP_031890457.1 CGLO|KAF3804943.1 CHIG|XP_018164448.1 CVIN|KAF4919410.1 CVYL|A12110 FGRM|XP_011316854.1 MGRA|XP_003856205.1 MLAR|XP_007413588.1 MORY|QBZ54708.1 NCRA|XP_964422.1 SSCL|APA08981.1

>Orthogroup3492: ANID|CBF89004.1 BCIN|XP_001559538.1 BGRA|VDB89213.1 CFRU|XP_031891158.1 CGLO|KAF3804493.1 CHIG|XP_018160321.1 CVIN|KAF4930981.1 CVYL|A04207 FGRM|XP_011324751.1 MGRA|XP_003856340.1 MLAR|XP_007408125.1 MORY|QBZ59108.1 NCRA|XP_961120.1 SSCL|APA09535.1

>Orthogroup3493: ANID|CBF89025.1 BCIN|XP_001547579.1 BGRA|VCU41301.1 CFRU|XP_031889159.1 CGLO|KAF3808692.1 CHIG|XP_018155555.1 CVIN|KAF4929647.1 CVYL|A00955 FGRM|XP_011321114.1 MGRA|XP_003857009.1 MLAR|XP_007406487.1 MORY|QBZ60106.1 NCRA|XP_957390.1 SSCL|APA07465.1

>Orthogroup3494: ANID|CBF89031.1 BCIN|XP_024548712.1 BGRA|VDB89319.1 CFRU|XP_031882148.1 CGLO|KAF3804447.1 CHIG|XP_018160271.1 CVIN|KAF4930920.1 CVYL|A04156 FGRM|XP_011324505.1 MGRA|XP_003856342.1 MLAR|XP_007416709.1 MORY|QBZ53831.1 NCRA|XP_960744.3 SSCL|APA09981.1

>Orthogroup3495: ANID|CBF89063.1 BCIN|XP_001559530.1 BGRA|VDB89287.1 CFRU|XP_031886478.1 CGLO|KAF3806170.1 CHIG|XP_018162155.1 CVIN|KAF4931574.1 CVYL|A11756 FGRM|XP_011324089.1 MGRA|XP_003856234.1 MLAR|XP_007412217.1 MORY|QBZ61385.1 NCRA|XP_011393782.1 SSCL|APA09530.1

>Orthogroup3496: ANID|CBF89073.1 BCIN|XP_001560698.1 BGRA|VDB90347.1 CFRU|XP_031885413.1 CGLO|KAF3799426.1 CHIG|XP_018152298.1 CVIN|KAF4931732.1 CVYL|A13290 FGRM|XP_011323656.1 MGRA|XP_003855593.1 MLAR|XP_007412063.1 MORY|QBZ64787.1 NCRA|XP_955777.1 SSCL|APA07987.1

>Orthogroup3497: ANID|CBF89079.1 BCIN|XP_001557459.1 BGRA|VCU41034.1 CFRU|XP_031886535.1 CGLO|KAF3800278.1 CHIG|XP_018162765.1 CVIN|KAF4927560.1 CVYL|A06958 FGRM|XP_011318456.1 MGRA|XP_003854934.1 MLAR|XP_007406768.1 MORY|QBZ61595.1 NCRA|XP_957356.3 SSCL|APA10642.1

>Orthogroup3498: ANID|CBF89085.1 BCIN|XP_001548863.1 BGRA|VDB90350.1 CFRU|XP_031881164.1 CGLO|KAF3802521.1 CHIG|XP_018154840.1 CVIN|KAF4918996.1 CVYL|A02113 FGRM|XP_011316703.1 MGRA|XP_003855412.1 MLAR|XP_007403641.1 MORY|QBZ60994.1 NCRA|XP_958196.1 SSCL|APA15982.1

>Orthogroup3499: ANID|CBF89096.1 BCIN|XP_001546275.1 BGRA|VCU39785.1 CFRU|XP_031886539.1 CGLO|KAF3800286.1 CHIG|XP_018162774.1 CVIN|KAF4927558.1 CVYL|A06948 FGRM|XP_011318469.1 MGRA|XP_003855669.1 MLAR|XP_007413938.1 MORY|QBZ63887.1 NCRA|XP_956671.2 SSCL|APA10244.1

>Orthogroup3500: ANID|CBF89108.1 BCIN|XP_001545584.1 BGRA|VCU39472.1 CFRU|XP_031885115.1 CGLO|KAF3800301.1 CHIG|XP_018162790.1 CVIN|KAF4927578.1 CVYL|A06928 FGRM|XP_011326426.1 MGRA|XP_003855691.1 MLAR|XP_007412021.1 MORY|QBZ63844.1 NCRA|XP_957779.1 SSCL|APA10205.1

>Orthogroup3502: ANID|CBF89158.1 BCIN|XP_024552204.1 BGRA|VCU39480.1 CFRU|XP_031877117.1 CGLO|KAF3804000.1 CHIG|XP_018150935.1 CVIN|KAF4925205.1 CVYL|A08802 FGRM|XP_011323763.1 MGRA|XP_003849303.1 MLAR|XP_007409100.1 MORY|QBZ59433.1 NCRA|XP_962347.1 SSCL|APA12893.1

>Orthogroup3503: ANID|CBF89160.1 BCIN|XP_001558194.1 BGRA|VDB89687.1 CFRU|XP_031878546.1 CGLO|KAF3798523.1 CHIG|XP_018161201.1 CVIN|KAF4919294.1 CVYL|A03802 FGRM|XP_011327904.1 MGRA|XP_003849625.1 MLAR|XP_007409251.1 MORY|QBZ57200.1 NCRA|XP_964443.1 SSCL|APA13084.1

>Orthogroup3505: ANID|CBF89169.1 BCIN|XP_024552106.1 BGRA|VDB92755.1 CFRU|XP_031878625.1 CGLO|KAF3798502.1 CHIG|XP_018161219.1 CVIN|KAF4917474.1 CVYL|A03822 FGRM|XP_011327929.1 MGRA|XP_003849357.1 MLAR|XP_007411537.1 MORY|QBZ64528.1 NCRA|XP_011394267.1 SSCL|APA12936.1

>Orthogroup3506: ANID|CBF89188.1 BCIN|XP_024552077.1 BGRA|VDB92746.1 CFRU|XP_031878624.1 CGLO|KAF3798503.1 CHIG|XP_018161220.1 CVIN|KAF4917475.1 CVYL|A03821 FGRM|XP_011327930.1 MGRA|XP_003849711.1 MLAR|XP_007404055.1 MORY|QBZ64529.1 NCRA|XP_962298.1 SSCL|APA12858.1

>Orthogroup3507: ANID|CBF89196.1 BCIN|XP_001550272.1 BGRA|VDB92748.1 CFRU|XP_031878544.1 CGLO|KAF3798528.1 CHIG|XP_018161206.1 CVIN|KAF4919295.1 CVYL|A03797 FGRM|XP_011327980.1 MGRA|XP_003849415.1 MLAR|XP_007406630.1 MORY|QBZ57213.1 NCRA|XP_961969.1 SSCL|APA12855.1

>Orthogroup3509: ANID|CBF89203.1 BCIN|XP_001556347.1 BGRA|VDB88250.1 CFRU|XP_031882591.1 CGLO|KAF3800487.1 CHIG|XP_018152280.1 CVIN|KAF4906660.1 CVYL|A13226 FGRM|XP_011323684.1 MGRA|XP_003849544.1 MLAR|XP_007410123.1 MORY|QBZ53771.1 NCRA|XP_011393532.1 SSCL|APA08728.1

>Orthogroup3510: ANID|CBF89206.1 BCIN|XP_001556314.2 BGRA|VDB88288.1 CFRU|XP_031886001.1 CGLO|KAF3812010.1 CHIG|XP_018155241.1 CVIN|KAF4930438.1 CVYL|A05499 FGRM|XP_011317469.1 MGRA|XP_003849740.1 MLAR|XP_007417759.1 MORY|QBZ60963.1 NCRA|XP_955894.1 SSCL|APA15702.1

>Orthogroup3511: ANID|CBF89210.1 BCIN|XP_001550302.1 BGRA|VDB92826.1 CFRU|XP_031878607.1 CGLO|KAF3798513.1 CHIG|XP_018161191.1 CVIN|KAF4917492.1 CVYL|A03812 FGRM|XP_011328780.1 MGRA|XP_003849605.1 MLAR|XP_007414340.1 MORY|QBZ56517.1 NCRA|XP_962404.1 SSCL|APA12834.1

>Orthogroup3512: ANID|CBF89212.1 BCIN|XP_001556324.1 BGRA|VDB87872.1 CFRU|XP_031881207.1 CGLO|KAF3802464.1 CHIG|XP_018154769.1 CVIN|KAF4916864.1 CVYL|A02063 FGRM|XP_011325547.1 MGRA|XP_003849829.1 MLAR|XP_007416019.1 MORY|QBZ64782.1 NCRA|XP_956128.1 SSCL|APA08739.1

>Orthogroup3513: ANID|CBF89232.1 BCIN|XP_024552218.1 BGRA|VDB92830.1 CFRU|XP_031887613.1 CGLO|KAF3803178.1 CHIG|XP_018161018.1 CVIN|KAF4920228.1 CVYL|A03608 FGRM|XP_011327870.1 MGRA|XP_003849694.1 MLAR|XP_007410632.1 MORY|QBZ58828.1 NCRA|XP_961819.3 SSCL|APA12992.1

>Orthogroup3514: ANID|CBF89237.1 BCIN|XP_001553860.2 BGRA|VCU39227.1 CFRU|XP_031887579.1 CGLO|KAF3811617.1 CHIG|XP_018160891.1 CVIN|KAF4921473.1 CVYL|A03475 FGRM|XP_011328153.1 MGRA|XP_003849416.1 MLAR|XP_007417507.1 MORY|QBZ56589.1 NCRA|XP_962666.1 SSCL|APA13076.1

>Orthogroup3516: ANID|CBF89348.1 BCIN|XP_001554818.1 BGRA|VDB91205.1 CFRU|XP_031879439.1 CGLO|KAF3802404.1 CHIG|XP_018154704.1 CVIN|KAF4922175.1 CVYL|A13548 FGRM|XP_011317269.1 MGRA|XP_003849587.1 MLAR|XP_007412681.1 MORY|QBZ64844.1 NCRA|XP_956721.2 SSCL|APA15524.1

>Orthogroup3517: ANID|CBF89350.1 BCIN|XP_001554817.1 BGRA|VDB91203.1 CFRU|XP_031879438.1 CGLO|KAF3802403.1 CHIG|XP_018154703.1 CVIN|KAF4922174.1 CVYL|A13547 FGRM|XP_011317270.1 MGRA|XP_003849588.1 MLAR|XP_007414990.1 MORY|QBZ64845.1 NCRA|XP_956722.1 SSCL|APA15523.1

>Orthogroup3518: ANID|CBF89380.1 BCIN|XP_024549977.1 BGRA|VDB96346.1 CFRU|XP_031878492.1 CGLO|KAF3798602.1 CHIG|XP_018153926.1 CVIN|KAF4930154.1 CVYL|A04844 FGRM|XP_011327729.1 MGRA|XP_003853811.1 MLAR|XP_007410197.1 MORY|QBZ58120.1 NCRA|XP_957732.2 SSCL|APA05307.1

>Orthogroup3519: ANID|CBF89414.1 BCIN|XP_001560795.1 BGRA|VDB90431.1 CFRU|XP_031885662.1 CGLO|KAF3810109.1 CHIG|XP_018157861.1 CVIN|KAF4910253.1 CVYL|A02702 FGRM|XP_011319967.1 MGRA|XP_003854580.1 MLAR|XP_007416014.1 MORY|QBZ62184.1 NCRA|XP_960147.1 SSCL|APA08038.1

>Orthogroup3520: ANID|CBF89423.1 BCIN|XP_001545158.2 BGRA|VDB94795.1 CFRU|XP_031891030.1 CGLO|KAF3801602.1 CHIG|XP_018151361.1 CVIN|KAF4922686.1 CVYL|A12534 FGRM|XP_011320357.1 MGRA|XP_003854502.1 MLAR|XP_007415660.1 MORY|QBZ59069.1 NCRA|XP_960432.1 SSCL|APA08004.1

>Orthogroup3521: ANID|CBF89435.1 BCIN|XP_024548416.1 BGRA|VDB88739.1 CFRU|XP_031877928.1 CGLO|KAF3810951.1 CHIG|XP_018154903.1 CVIN|KAF4928525.1 CVYL|A02197 FGRM|XP_011324635.1 MGRA|XP_003856607.1 MLAR|XP_007418172.1 MORY|QBZ60922.1 NCRA|XP_011393644.1 SSCL|APA12284.1

>Orthogroup3522: ANID|CBF89438.1 BCIN|XP_001560753.2 BGRA|VDB90375.1 CFRU|XP_031885670.1 CGLO|KAF3810240.1 CHIG|XP_018157876.1 CVIN|KAF4910805.1 CVYL|A02840 FGRM|XP_011325088.1 MGRA|XP_003851062.1 MLAR|XP_007411243.1 MORY|QBZ62663.1 NCRA|XP_957662.2 SSCL|APA08010.1

>Orthogroup3523: ANID|CBF89461.1 BCIN|XP_001555450.1 BGRA|VDB90777.1 CFRU|XP_031883293.1 CGLO|KAF3801693.1 CHIG|XP_018159218.1 CVIN|KAF4915647.1 CVYL|A13004 FGRM|XP_011320285.1 MGRA|XP_003854520.1 MLAR|XP_007411467.1 MORY|QBZ55463.1 NCRA|XP_960229.1 SSCL|APA08169.1

>Orthogroup3524: ANID|CBF89467.1 BCIN|XP_001546179.1 BGRA|VCU39627.1 CFRU|XP_031883251.1 CGLO|KAF3800538.1 CHIG|XP_018152145.1 CVIN|KAF4923823.1 CVYL|A13177 FGRM|XP_011316346.1 MGRA|XP_003857287.1 MLAR|XP_007404913.1 MORY|QBZ54398.1 NCRA|XP_957202.1 SSCL|APA16292.1

>Orthogroup3525: ANID|CBF89470.1 BCIN|XP_024553080.1 BGRA|VCU39623.1 CFRU|XP_031883255.1 CGLO|KAF3800541.1 CHIG|XP_018152142.1 CVIN|KAF4923795.1 CVYL|A13175 FGRM|XP_011316565.1 MGRA|XP_003856171.1 MLAR|XP_007414800.1 MORY|QBZ54392.1 NCRA|XP_957199.1 SSCL|APA16294.1

>Orthogroup3526: ANID|CBF89472.1 BCIN|XP_024553081.1 BGRA|VCU39670.1 CFRU|XP_031885433.1 CGLO|KAF3799529.1 CHIG|XP_018154518.1 CVIN|KAF4931773.1 CVYL|A13389 FGRM|XP_011324922.1 MGRA|XP_003856172.1 MLAR|XP_007404899.1 MORY|QBZ62064.1 NCRA|XP_011393586.1 SSCL|APA16295.1

>Orthogroup3527: ANID|CBF89487.1 BCIN|XP_001548121.1 BGRA|VDB93264.1 CFRU|XP_031890211.1 CGLO|KAF3802909.1 CHIG|XP_018158513.1 CVIN|KAF4907423.1 CVYL|A05104 FGRM|XP_011316421.1 MGRA|XP_003853499.1 MLAR|XP_007417054.1 MORY|QBZ55400.1 NCRA|XP_964814.2 SSCL|APA09548.1

>Orthogroup3530: ANID|CBF89502.1 BCIN|XP_001548118.1 BGRA|VDB93262.1 CFRU|XP_031890208.1 CGLO|KAF3802907.1 CHIG|XP_018158511.1 CVIN|KAF4907422.1 CVYL|A05106 FGRM|XP_011316423.1 MGRA|XP_003856126.1 MLAR|XP_007414921.1 MORY|QBZ63205.1 NCRA|XP_011392858.1 SSCL|APA09550.1

>Orthogroup3531: ANID|CBF89509.1 BCIN|XP_001549061.1 BGRA|VDB84085.1 CFRU|XP_031890579.1 CGLO|KAF3806728.1 CHIG|XP_018158704.1 CVIN|KAF4919494.1 CVYL|A14038 FGRM|XP_011317336.1 MGRA|XP_003855985.1 MLAR|XP_007416596.1 MORY|QBZ60457.1 NCRA|XP_963929.1 SSCL|APA09864.1

>Orthogroup3532: ANID|CBF89511.1 BCIN|XP_024548636.1 BGRA|VDB84084.1 CFRU|XP_031890582.1 CGLO|KAF3806729.1 CHIG|XP_018158706.1 CVIN|KAF4919493.1 CVYL|A14039 FGRM|XP_011317337.1 MGRA|XP_003854544.1 MLAR|XP_007415302.1 MORY|QBZ60458.1 NCRA|XP_011393202.1 SSCL|APA09865.1

>Orthogroup3533: ANID|CBF89513.1 BCIN|XP_024548630.1 BGRA|VDB84024.1 CFRU|XP_031890391.1 CGLO|KAF3806724.1 CHIG|XP_018158700.1 CVIN|KAF4919478.1 CVYL|A14032 FGRM|XP_011317331.1 MGRA|XP_003854555.1 MLAR|XP_007417753.1 MORY|QBZ60454.1 NCRA|XP_963932.2 SSCL|APA09859.1

>Orthogroup3534: ANID|CBF89518.1 BCIN|XP_024548806.1 BGRA|VCU40329.1 CFRU|XP_031890036.1 CGLO|KAF3798887.1 CHIG|XP_018158556.1 CVIN|KAF4925579.1 CVYL|A05066 FGRM|XP_011316615.1 MGRA|XP_003852904.1 MLAR|XP_007418050.1 MORY|QBZ55965.1 NCRA|XP_965015.2 SSCL|APA09642.1

>Orthogroup3536: ANID|CBF89544.1 BCIN|XP_024551377.1 BGRA|VCU41061.1 CFRU|XP_031886606.1 CGLO|KAF3802651.1 CHIG|XP_018162512.1 CVIN|KAF4932012.1 CVYL|A07144 FGRM|XP_011326965.1 MGRA|XP_003857454.1 MLAR|XP_007406209.1 MORY|QBZ55681.1 NCRA|XP_011393735.1 SSCL|APA10603.1

>Orthogroup3537: ANID|CBF89596.1 BCIN|XP_024546095.1 BGRA|VDB92886.1 CFRU|XP_031879878.1 CGLO|KAF3798944.1 CHIG|XP_018152870.1 CVIN|KAF4889612.1 CVYL|A07860 FGRM|XP_011324367.1 MGRA|XP_003851095.1 MLAR|XP_007410300.1 MORY|QBZ53358.1 NCRA|XP_959526.2 SSCL|APA06615.1

>Orthogroup3538: ANID|CBF89598.1 BCIN|XP_024546670.1 BGRA|VDB92417.1 CFRU|XP_031888543.1 CGLO|KAF3803555.1 CHIG|XP_018164140.1 CVIN|KAF4922282.1 CVYL|A14487 FGRM|XP_011316153.1 MGRA|XP_003850555.1 MLAR|XP_007417908.1 MORY|QBZ63825.1 NCRA|XP_963759.1 SSCL|APA06508.1

>Orthogroup3539: ANID|CBF89621.1 BCIN|XP_001556472.1 BGRA|VDB92960.1 CFRU|XP_031884315.1 CGLO|KAF3799936.1 CHIG|XP_018153067.1 CVIN|KAF4907000.1 CVYL|A14184 FGRM|XP_011323511.1 MGRA|XP_003851191.1 MLAR|XP_007414642.1 MORY|QBZ64027.1 NCRA|XP_964390.1 SSCL|APA06541.1

>Orthogroup3540: ANID|CBF89638.1 BCIN|XP_024546101.1 BGRA|VDB92941.1 CFRU|XP_031890494.1 CGLO|KAF3804910.1 CHIG|XP_018164353.1 CVIN|KAF4909780.1 CVYL|A12148 FGRM|XP_011317154.1 MGRA|XP_003850574.1 MLAR|XP_007405158.1 MORY|QBZ56447.1 NCRA|XP_964270.2 SSCL|APA06620.1

>Orthogroup3541: ANID|CBF89641.1 BCIN|XP_001548355.1 BGRA|VDB92587.1 CFRU|XP_031877051.1 CGLO|KAF3805200.1 CHIG|XP_018152951.1 CVIN|KAF4909095.1 CVYL|A07956 FGRM|XP_011324274.1 MGRA|XP_003851252.1 MLAR|XP_007407527.1 MORY|QBZ64163.1 NCRA|XP_964950.1 SSCL|APA06951.1

>Orthogroup3542: ANID|CBF89643.1 BCIN|XP_024546094.1 BGRA|VDB92889.1 CFRU|XP_031879888.1 CGLO|KAF3798950.1 CHIG|XP_018152864.1 CVIN|KAF4923499.1 CVYL|A07866 FGRM|XP_011324363.1 MGRA|XP_003851176.1 MLAR|XP_007407822.1 MORY|QBZ53369.1 NCRA|XP_959522.1 SSCL|APA06612.1

>Orthogroup3543: ANID|CBF89661.1 BCIN|XP_024546573.1 BGRA|VDB92421.1 CFRU|XP_031877036.1 CGLO|KAF3811110.1 CHIG|XP_018152939.1 CVIN|KAF4911067.1 CVYL|A07974 FGRM|XP_011328006.1 MGRA|XP_003850633.1 MLAR|XP_007413683.1 MORY|QBZ64145.1 NCRA|XP_964945.1 SSCL|APA06497.1

>Orthogroup3545: ANID|CBF89674.1 BCIN|XP_024546065.1 BGRA|VCU39094.1 CFRU|XP_031888117.1 CGLO|KAF3802301.1 CHIG|XP_018154403.1 CVIN|KAF4924422.1 CVYL|A10685 FGRM|XP_011319764.1 MGRA|XP_003847813.1 MLAR|XP_007405963.1 MORY|QBZ65728.1 NCRA|XP_965547.1 SSCL|APA07245.1

>Orthogroup3546: ANID|CBF89710.1 BCIN|XP_024549903.1 BGRA|VDB93951.1 CFRU|XP_031885368.1 CGLO|KAF3799551.1 CHIG|XP_018154543.1 CVIN|KAF4931752.1 CVYL|A13411 FGRM|XP_011324903.1 MGRA|XP_003850449.1 MLAR|XP_007406825.1 MORY|QBZ61055.1 NCRA|XP_963330.1 SSCL|APA05582.1

>Orthogroup3547: ANID|CBF89718.1 BCIN|XP_024547824.1 BGRA|VDB88261.1 CFRU|XP_031887106.1 CGLO|KAF3811468.1 CHIG|XP_018160715.1 CVIN|KAF4895033.1 CVYL|A04671 FGRM|XP_011327254.1 MGRA|XP_003850289.1 MLAR|XP_007407471.1 MORY|QBZ54255.1 NCRA|XP_955842.2 SSCL|APA08131.1

>Orthogroup3549: ANID|CBF89731.1 BCIN|XP_024553272.1 BGRA|VDB93969.1 CFRU|XP_031883305.1 CGLO|KAF3800593.1 CHIG|XP_018152085.1 CVIN|KAF4927052.1 CVYL|A13122 FGRM|XP_011321296.1 MGRA|XP_003848919.1 MLAR|XP_007412722.1 MORY|QBZ61045.1 NCRA|XP_963256.2 SSCL|APA13565.1

>Orthogroup3552: ANID|CBF89780.1 BCIN|XP_001559975.1 BGRA|VDB84040.1 CFRU|XP_031890279.1 CGLO|KAF3798892.1 CHIG|XP_018158563.1 CVIN|KAF4925548.1 CVYL|A05061 FGRM|XP_011316610.1 MGRA|XP_003852631.1 MLAR|XP_007414446.1 MORY|QBZ55991.1 NCRA|XP_965684.1 SSCL|APA09788.1

>Orthogroup3553: ANID|CBF89782.1 BCIN|XP_024546318.1 BGRA|VDB90693.1 CFRU|XP_031888449.1 CGLO|KAF3803479.1 CHIG|XP_018164010.1 CVIN|KAF4926322.1 CVYL|A14408 FGRM|XP_011316990.1 MGRA|XP_003852268.1 MLAR|XP_007405633.1 MORY|QBZ55344.1 NCRA|XP_011392999.1 SSCL|APA06903.1

>Orthogroup3554: ANID|CBF89788.1 BCIN|XP_024551172.1 BGRA|VCU39774.1 CFRU|XP_031893425.1 CGLO|KAF3808369.1 CHIG|XP_018152431.1 CVIN|KAF4916836.1 CVYL|A09528 FGRM|XP_011317878.1 MGRA|XP_003848508.1 MLAR|XP_007411453.1 MORY|QBZ54054.1 NCRA|XP_961292.3 SSCL|APA10185.1

>Orthogroup3555: ANID|CBF89791.1 BCIN|XP_024549873.1 BGRA|VDB90936.1 CFRU|XP_031888421.1 CGLO|KAF3803649.1 CHIG|XP_018163812.1 CVIN|KAF4928517.1 CVYL|A14582 FGRM|XP_011317001.1 MGRA|XP_003853192.1 MLAR|XP_007419090.1 MORY|QBZ54666.1 NCRA|XP_963750.2 SSCL|APA05546.1

>Orthogroup3556: ANID|CBF89801.1 BCIN|XP_001556929.1 BGRA|VDB89154.1 CFRU|XP_031886565.1 CGLO|KAF3804073.1 CHIG|XP_018162692.1 CVIN|KAF4919537.1 CVYL|A07036 FGRM|XP_011326940.1 MGRA|XP_003857205.1 MLAR|XP_007412490.1 MORY|QBZ55656.1 NCRA|XP_956377.2 SSCL|APA10541.1

>Orthogroup3557: ANID|CBF89820.1 BCIN|XP_001559021.1 BGRA|VDB90652.1 CFRU|XP_031890646.1 CGLO|KAF3804856.1 CHIG|XP_018164413.1 CVIN|KAF4921144.1 CVYL|A12217 FGRM|XP_011316822.1 MGRA|XP_003852312.1 MLAR|XP_007417612.1 MORY|QBZ63629.1 NCRA|XP_965337.1 SSCL|APA09248.1

>Orthogroup3559: ANID|CBF89848.1 BCIN|XP_001557017.2 BGRA|VCU39510.1 CFRU|XP_031878416.1 CGLO|KAF3810763.1 CHIG|XP_018162560.1 CVIN|KAF4932083.1 CVYL|A07191 FGRM|XP_011326312.1 MGRA|XP_003857375.1 MLAR|XP_007404254.1 MORY|QBZ59704.1 NCRA|XP_956395.3 SSCL|APA10252.1

>Orthogroup3560: ANID|CBF89864.1 BCIN|XP_001559945.1 BGRA|VDB93277.1 CFRU|XP_031886650.1 CGLO|KAF3807476.1 CHIG|XP_018158761.1 CVIN|KAF4907601.1 CVYL|A04999 FGRM|XP_011328621.1 MGRA|XP_003852493.1 MLAR|XP_007403623.1 MORY|QBZ60418.1 NCRA|XP_965316.1 SSCL|APA09771.1

>Orthogroup3561: ANID|CBF89871.1 BCIN|XP_024549039.1 BGRA|VDB89691.1 CFRU|XP_031884442.1 CGLO|KAF3799074.1 CHIG|XP_018158184.1 CVIN|KAF4918248.1 CVYL|A11455 FGRM|XP_011328384.1 MGRA|XP_003853124.1 MLAR|XP_007412732.1 MORY|QBZ54110.1 NCRA|XP_964357.2 SSCL|APA09268.1

>Orthogroup3562: ANID|CBF89873.1 BCIN|XP_001554103.1 BGRA|VDB90935.1 CFRU|XP_031888420.1 CGLO|KAF3803647.1 CHIG|XP_018163811.1 CVIN|KAF4913940.1 CVYL|A14581 FGRM|XP_011317002.1 MGRA|XP_003852185.1 MLAR|XP_007416693.1 MORY|QBZ54665.1 NCRA|XP_963919.1 SSCL|APA05547.1

>Orthogroup3563: ANID|CBF89874.1 BCIN|XP_001554094.1 BGRA|VDB90940.1 CFRU|XP_031888388.1 CGLO|KAF3803631.1 CHIG|XP_018163828.1 CVIN|KAF4913956.1 CVYL|A14566 FGRM|XP_011326532.1 MGRA|XP_003852512.1 MLAR|XP_007409406.1 MORY|QBZ54686.1 NCRA|XP_011393241.1 SSCL|APA05554.1

>Orthogroup3564: ANID|CBF89876.1 BCIN|XP_001554096.1 BGRA|VDB90939.1 CFRU|XP_031888381.1 CGLO|KAF3803630.1 CHIG|XP_018163829.1 CVIN|KAF4913960.1 CVYL|A14565 FGRM|XP_011326533.1 MGRA|XP_003852292.1 MLAR|XP_007409568.1 MORY|QBZ54685.1 NCRA|XP_958891.1 SSCL|APA05553.1

>Orthogroup3565: ANID|CBF89895.1 BCIN|XP_024547667.1 BGRA|VDB85799.1 CFRU|XP_031880393.1 CGLO|KAF3807265.1 CHIG|XP_018156938.1 CVIN|KAF4928129.1 CVYL|A10747 FGRM|XP_011320010.1 MGRA|XP_003854579.1 MLAR|XP_007417056.1 MORY|QBZ66171.1 NCRA|XP_964292.1 SSCL|APA07732.1

>Orthogroup3566: ANID|CBF89912.1 BCIN|XP_001549976.1 BGRA|VDB92885.1 CFRU|XP_031879884.1 CGLO|KAF3798939.1 CHIG|XP_018152865.1 CVIN|KAF4898131.1 CVYL|A07856 FGRM|XP_011324364.1 MGRA|XP_003851122.1 MLAR|XP_007403848.1 MORY|QBZ53382.1 NCRA|XP_959521.1 SSCL|APA06614.1

>Orthogroup3567: ANID|CBF89933.1 BCIN|XP_001555013.1 BGRA|VDB86146.1 CFRU|XP_031882308.1 CGLO|KAF3804414.1 CHIG|XP_018160228.1 CVIN|KAF4930958.1 CVYL|A04114 FGRM|XP_011328785.1 MGRA|XP_003850299.1 MLAR|XP_007412128.1 MORY|QBZ59150.1 NCRA|XP_960635.1 SSCL|APA07785.1

>Orthogroup3568: ANID|CBF89978.1 BCIN|XP_001558171.2 BGRA|VCU39496.1 CFRU|XP_031877497.1 CGLO|KAF3798435.1 CHIG|XP_018161253.1 CVIN|KAF4920003.1 CVYL|A11202 FGRM|XP_011321441.1 MGRA|XP_003849765.1 MLAR|XP_007409021.1 MORY|QBZ56760.1 NCRA|XP_961962.2 SSCL|APA13142.1

>Orthogroup3569: ANID|CBF89980.1 BCIN|XP_001558170.1 BGRA|VCU39489.1 CFRU|XP_031887640.1 CGLO|KAF3803266.1 CHIG|XP_018161132.1 CVIN|KAF4928698.1 CVYL|A03701 FGRM|XP_011327954.1 MGRA|XP_003849352.1 MLAR|XP_007404585.1 MORY|QBZ58360.1 NCRA|XP_961777.1 SSCL|APA13141.1

>Orthogroup3570: ANID|CBF89982.1 BCIN|XP_024552200.1 BGRA|VDB92768.1 CFRU|XP_031887759.1 CGLO|KAF3803275.1 CHIG|XP_018161120.1 CVIN|KAF4928636.1 CVYL|A03710 FGRM|XP_011321436.1 MGRA|XP_003849360.1 MLAR|XP_007411116.1 MORY|QBZ56751.1 NCRA|XP_001728327.1 SSCL|APA12889.1

>Orthogroup3572: ANID|CBF89989.1 BCIN|XP_001558140.1 BGRA|VCU39207.1 CFRU|XP_031876694.1 CGLO|KAF3798699.1 CHIG|XP_018157455.1 CVIN|KAF4918501.1 CVYL|A09436 FGRM|XP_011321347.1 MGRA|XP_003849427.1 MLAR|XP_007416012.1 MORY|QBZ57156.1 NCRA|XP_962698.1 SSCL|APA12983.1

>Orthogroup3574: ANID|CBF90009.1 BCIN|XP_001558034.2 BGRA|VDB92766.1 CFRU|XP_031877500.1 CGLO|KAF3798437.1 CHIG|XP_018161255.1 CVIN|KAF4920006.1 CVYL|A11200 FGRM|XP_011321443.1 MGRA|XP_003851258.1 MLAR|XP_007410571.1 MORY|QBZ56758.1 NCRA|XP_961961.1 SSCL|APA12877.1

>Orthogroup3576: ANID|CBF90056.1 BCIN|XP_024552088.1 BGRA|VDB89540.1 CFRU|XP_031887700.1 CGLO|KAF3803222.1 CHIG|XP_018161074.1 CVIN|KAF4928715.1 CVYL|A03653 FGRM|XP_011324212.1 MGRA|XP_003849515.1 MLAR|XP_007414580.1 MORY|QBZ58801.1 NCRA|XP_956325.1 SSCL|APA12876.1

>Orthogroup3577: ANID|CBF90063.1 BCIN|XP_024552233.1 BGRA|VDB92777.1 CFRU|XP_031876569.1 CGLO|KAF3800894.1 CHIG|XP_018150788.1 CVIN|KAF4911866.1 CVYL|A11136 FGRM|XP_011323793.1 MGRA|XP_003849743.1 MLAR|XP_007412593.1 MORY|QBZ56526.1 NCRA|XP_962268.2 SSCL|APA13016.1

>Orthogroup3579: ANID|CBF90085.1 BCIN|XP_001555228.1 BGRA|VDB88130.1 CFRU|XP_031885921.1 CGLO|KAF3805532.1 CHIG|XP_018155366.1 CVIN|KAF4918287.1 CVYL|A05655 FGRM|XP_011317448.1 MGRA|XP_003849446.1 MLAR|XP_007406407.1 MORY|QBZ58992.1 NCRA|XP_963590.1 SSCL|APA15621.1

>Orthogroup3580: ANID|CBF90114.1 BCIN|XP_001555223.2 BGRA|VDB88118.1 CFRU|XP_031878009.1 CGLO|KAF3799594.1 CHIG|XP_018154589.1 CVIN|KAF4926455.1 CVYL|A13455 FGRM|XP_011321224.1 MGRA|XP_003849669.1 MLAR|XP_007407368.1 MORY|QBZ54424.1 NCRA|XP_956475.1 SSCL|APA15626.1

>Orthogroup3581: ANID|CBF90118.1 BCIN|XP_001554754.1 BGRA|VDB89743.1 CFRU|XP_031878002.1 CGLO|KAF3799599.1 CHIG|XP_018154594.1 CVIN|KAF4926500.1 CVYL|A13460 FGRM|XP_011316341.1 MGRA|XP_003849772.1 MLAR|XP_007404608.1 MORY|QBZ54403.1 NCRA|XP_956478.1 SSCL|APA15486.1

>Orthogroup3582: ANID|CBF90121.1 BCIN|XP_001554752.1 BGRA|VDB87890.1 CFRU|XP_031885331.1 CGLO|KAF3799473.1 CHIG|XP_018154458.1 CVIN|KAF4931792.1 CVYL|A13335 FGRM|XP_011325533.1 MGRA|XP_003849593.1 MLAR|XP_007410316.1 MORY|QBZ64882.1 NCRA|XP_957179.2 SSCL|APA15488.1

>Orthogroup3583: ANID|CBF90125.1 BCIN|XP_001554750.1 BGRA|VDB87878.1 CFRU|XP_031885333.1 CGLO|KAF3799471.1 CHIG|XP_018154456.1 CVIN|KAF4931888.1 CVYL|A13333 FGRM|XP_011325527.1 MGRA|XP_003849527.1 MLAR|XP_007415046.1 MORY|QBZ64884.1 NCRA|XP_956217.1 SSCL|APA15490.1

>Orthogroup3584: ANID|CBF90132.1 BCIN|XP_024552542.1 BGRA|VDB87670.1 CFRU|XP_031879416.1 CGLO|KAF3802419.1 CHIG|XP_018154717.1 CVIN|KAF4918213.1 CVYL|A13562 FGRM|XP_011325649.1 MGRA|XP_003849378.1 MLAR|XP_007410299.1 MORY|QBZ64865.1 NCRA|XP_011393469.1 SSCL|APA08638.1

>Orthogroup3585: ANID|CBF90137.1 BCIN|XP_001556350.1 BGRA|VDB88252.1 CFRU|XP_031879511.1 CGLO|KAF3802392.1 CHIG|XP_018154684.1 CVIN|KAF4922184.1 CVYL|A13535 FGRM|XP_011317272.1 MGRA|XP_003849763.1 MLAR|XP_007405107.1 MORY|QBZ64807.1 NCRA|XP_955761.1 SSCL|APA08726.1

>Orthogroup3586: ANID|CBF90143.1 BCIN|XP_001548678.2 BGRA|VDB88069.1 CFRU|XP_031883269.1 CGLO|KAF3800505.1 CHIG|XP_018152211.1 CVIN|KAF4919226.1 CVYL|A13209 FGRM|XP_011321318.1 MGRA|XP_003849744.1 MLAR|XP_007405241.1 MORY|QBZ61099.1 NCRA|XP_011393570.1 SSCL|APA15606.1

>Orthogroup3587: ANID|CBF90145.1 BCIN|XP_024552638.1 BGRA|VDB88070.1 CFRU|XP_031883326.1 CGLO|KAF3800506.1 CHIG|XP_018152212.1 CVIN|KAF4919225.1 CVYL|A13208 FGRM|XP_011321319.1 MGRA|XP_003849370.1 MLAR|XP_007416894.1 MORY|QBZ61100.1 NCRA|XP_956727.2 SSCL|APA15605.1

>Orthogroup3588: ANID|CBF90150.1 BCIN|XP_001558190.1 BGRA|VDB89683.1 CFRU|XP_031878634.1 CGLO|KAF3798526.1 CHIG|XP_018161204.1 CVIN|KAF4919297.1 CVYL|A03799 FGRM|XP_011327907.1 MGRA|XP_003849618.1 MLAR|XP_007415933.1 MORY|QBZ57203.1 NCRA|XP_961971.2 SSCL|APA13087.1

>Orthogroup3589: ANID|CBF90159.1 BCIN|XP_024552372.1 BGRA|VCU39153.1 CFRU|XP_031885298.1 CGLO|KAF3806498.1 CHIG|XP_018157534.1 CVIN|KAF4886795.1 CVYL|A09371 FGRM|XP_011327888.1 MGRA|XP_003849498.1 MLAR|XP_007418245.1 MORY|QBZ56357.1 NCRA|XP_961896.1 SSCL|APA13216.1

>Orthogroup3590: ANID|CBF90161.1 BCIN|XP_024552373.1 BGRA|VCU39149.1 CFRU|XP_031885297.1 CGLO|KAF3806497.1 CHIG|XP_018157533.1 CVIN|KAF4919439.1 CVYL|A09372 FGRM|XP_011328775.1 MGRA|XP_003849619.1 MLAR|XP_007417933.1 MORY|QBZ56358.1 NCRA|XP_961898.1 SSCL|APA13215.1

>Orthogroup3591: ANID|CBF90171.1 BCIN|XP_001548672.1 BGRA|VDB88040.1 CFRU|XP_031881862.1 CGLO|KAF3800677.1 CHIG|XP_018152005.1 CVIN|KAF4922057.1 CVYL|A13039 FGRM|XP_011321258.1 MGRA|XP_003849610.1 MLAR|XP_007413762.1 MORY|QBZ62130.1 NCRA|XP_956205.1 SSCL|APA15601.1

>Orthogroup3592: ANID|CBF90181.1 BCIN|XP_024552295.1 BGRA|VCU39225.1 CFRU|XP_031893296.1 CGLO|KAF3799102.1 CHIG|XP_018157445.1 CVIN|KAF4908268.1 CVYL|A09446 FGRM|XP_011321332.1 MGRA|XP_003849430.1 MLAR|XP_007414107.1 MORY|QBZ57167.1 NCRA|XP_962705.3 SSCL|APA13072.1

>Orthogroup3594: ANID|CBF90192.1 BCIN|XP_024552271.1 BGRA|VCU39302.1 CFRU|XP_031893027.1 CGLO|KAF3800833.1 CHIG|XP_018150871.1 CVIN|KAF4922458.1 CVYL|A08716 FGRM|XP_011328053.1 MGRA|XP_003849603.1 MLAR|XP_007407396.1 MORY|QBZ56825.1 NCRA|XP_962259.1 SSCL|APA13132.1

>Orthogroup3595: ANID|CBF90222.1 BCIN|XP_001554031.1 BGRA|VDB94567.1 CFRU|XP_031886941.1 CGLO|KAF3811347.1 CHIG|XP_018160591.1 CVIN|KAF4921004.1 CVYL|A04529 FGRM|XP_011323641.1 MGRA|XP_003854495.1 MLAR|XP_007415301.1 MORY|QBZ56982.1 NCRA|XP_961487.1 SSCL|APA11119.1

>Orthogroup3596: ANID|CBF90225.1 BCIN|XP_001551941.2 BGRA|VDB94779.1 CFRU|XP_031877178.1 CGLO|KAF3806444.1 CHIG|XP_018162332.1 CVIN|KAF4918059.1 CVYL|A12041 FGRM|XP_011323949.1 MGRA|XP_003855375.1 MLAR|XP_007411498.1 MORY|QBZ61409.1 NCRA|XP_962809.1 SSCL|APA10943.1

>Orthogroup3597: ANID|CBF90234.1 BCIN|XP_001548704.2 BGRA|VDB94947.1 CFRU|XP_031891178.1 CGLO|KAF3804678.1 CHIG|XP_018161749.1 CVIN|KAF4902796.1 CVYL|A05205 FGRM|XP_011321531.1 MGRA|XP_003855367.1 MLAR|XP_007409384.1 MORY|QBZ59568.1 NCRA|XP_956705.3 SSCL|APA10858.1

>Orthogroup3598: ANID|CBF90245.1 BCIN|XP_024550531.1 BGRA|VDB94611.1 CFRU|XP_031887039.1 CGLO|KAF3811449.1 CHIG|XP_018160693.1 CVIN|KAF4917879.1 CVYL|A04650 FGRM|XP_011327228.1 MGRA|XP_003854889.1 MLAR|XP_007404128.1 MORY|QBZ53841.1 NCRA|XP_960655.1 SSCL|APA11308.1

>Orthogroup3599: ANID|CBF90250.1 BCIN|XP_024550277.1 BGRA|VDB94743.1 CFRU|XP_031887099.1 CGLO|KAF3811333.1 CHIG|XP_018160560.1 CVIN|KAF4927214.1 CVYL|A04515 FGRM|XP_011326902.1 MGRA|XP_003849201.1 MLAR|XP_007414751.1 MORY|QBZ57010.1 NCRA|XP_961515.1 SSCL|APA10947.1

>Orthogroup3600: ANID|CBF90252.1 BCIN|XP_024550532.1 BGRA|VDB94591.1 CFRU|XP_031887042.1 CGLO|KAF3811450.1 CHIG|XP_018160694.1 CVIN|KAF4917878.1 CVYL|A04651 FGRM|XP_011327229.1 MGRA|XP_003853710.1 MLAR|XP_007413037.1 MORY|QBZ53840.1 NCRA|XP_960774.1 SSCL|APA11312.1

>Orthogroup3601: ANID|CBF90266.1 BCIN|XP_001548712.1 BGRA|VDB94836.1 CFRU|XP_031877202.1 CGLO|KAF3806450.1 CHIG|XP_018162338.1 CVIN|KAF4918071.1 CVYL|A12048 FGRM|XP_011323940.1 MGRA|XP_003849185.1 MLAR|XP_007412146.1 MORY|QBZ61404.1 NCRA|XP_962820.1 SSCL|APA10852.1

>Orthogroup3602: ANID|CBF90267.1 BCIN|XP_024550456.1 BGRA|VDB94911.1 CFRU|XP_031877187.1 CGLO|KAF3806447.1 CHIG|XP_018162335.1 CVIN|KAF4918055.1 CVYL|A12045 FGRM|XP_011323946.1 MGRA|XP_003855352.1 MLAR|XP_007405152.1 MORY|QBZ61407.1 NCRA|XP_962817.1 SSCL|APA11167.1

>Orthogroup3603: ANID|CBF90269.1 BCIN|XP_024550457.1 BGRA|VDB94910.1 CFRU|XP_031877186.1 CGLO|KAF3806448.1 CHIG|XP_018162336.1 CVIN|KAF4918057.1 CVYL|A12046 FGRM|XP_011323945.1 MGRA|XP_003855130.1 MLAR|XP_007412794.1 MORY|QBZ61406.1 NCRA|XP_962818.1 SSCL|APA11168.1

>Orthogroup3604: ANID|CBF90277.1 BCIN|XP_024550175.1 BGRA|VDB94833.1 CFRU|XP_031877184.1 CGLO|KAF3806451.1 CHIG|XP_018162339.1 CVIN|KAF4897885.1 CVYL|A12049 FGRM|XP_011323941.1 MGRA|XP_003855138.1 MLAR|XP_007417923.1 MORY|QBZ61403.1 NCRA|XP_962821.2 SSCL|APA10851.1

>Orthogroup3605: ANID|CBF90310.1 BCIN|XP_024549870.1 BGRA|VDB90947.1 CFRU|XP_031888591.1 CGLO|KAF3803627.1 CHIG|XP_018163835.1 CVIN|KAF4913961.1 CVYL|A14560 FGRM|XP_011326540.1 MGRA|XP_003853260.1 MLAR|XP_007413169.1 MORY|QBZ60369.1 NCRA|XP_959261.2 SSCL|APA05543.1

>Orthogroup3606: ANID|CBF90313.1 BCIN|XP_001554616.1 BGRA|VDB94769.1 CFRU|XP_031891440.1 CGLO|KAF3804578.1 CHIG|XP_018161660.1 CVIN|KAF4923088.1 CVYL|A05299 FGRM|XP_011318682.1 MGRA|XP_003855135.1 MLAR|XP_007418797.1 MORY|QBZ59357.1 NCRA|XP_958511.2 SSCL|APA11061.1

>Orthogroup3607: ANID|CBF90315.1 BCIN|XP_024550371.1 BGRA|VDB94768.1 CFRU|XP_031891441.1 CGLO|KAF3804577.1 CHIG|XP_018161659.1 CVIN|KAF4923089.1 CVYL|A05300 FGRM|XP_011318683.1 MGRA|XP_003855345.1 MLAR|XP_007405092.1 MORY|QBZ59356.1 NCRA|XP_958512.3 SSCL|APA11062.1

>Orthogroup3609: ANID|CBF90326.1 BCIN|XP_024550362.1 BGRA|VDB94597.1 CFRU|XP_031886306.1 CGLO|KAF3806207.1 CHIG|XP_018162196.1 CVIN|KAF4931563.1 CVYL|A11793 FGRM|XP_011324066.1 MGRA|XP_003854929.1 MLAR|XP_007408474.1 MORY|QBZ55791.1 NCRA|XP_957745.3 SSCL|APA11053.1
