# Supplementary material for: Histological and transcriptomic insights into the interaction between grapevine and Colletotrichum viniferum
Source: Front Plant Sci. 2024 Aug 16;15:1446288. doi: 10.3389/fpls.2024.1446288 (PMC11362058; doi:10.3389/fpls.2024.1446288)
Supplement: Supplementary file 3 [file DataSheet3.docx]

***Supplementary Material***

**Histological and transcriptome analyses provide insight into the interaction of grape and *Colletotrichum viniferum***

Mengru Dou^1,2,3^, Yuhang Li^1,2,3^, Yu Hao^1,2,3^, Kangzhuang Zhang^1,2,3^, Xiao Yin^1,2,3^, Zinuo Feng^1,2,3^, Xi Xu^1,2,3^, Qi Zhang^1,2,3^, Wenwu Bao^1,2^, Xi Chen^1,2,3^, Guotian Liu^1,2,3^, Yuejin Wang^1,2,3^, Ling Tian^4,^*, Yan Xu^1,2,3,^*

^1^State Key Laboratory of Crop Stress Biology in Arid Areas, Northwest A&F University, Yangling, Shaanxi, P.R. China

^2^College of Horticulture, Northwest A&F University, Yangling, Shaanxi, P.R. China

^3^Key Laboratory of Horticultural Plant Biology and Germplasm Innovation in Northwest China, Ministry of Agriculture, Yangling, Shaanxi, P.R. China

^4^School of Management, Shenzhen Polytechnic University, Shenzhen, Guangdong, P.R. China

*** Correspondence:**Corresponding Author:

Ling Tian ([tianling@szpu.edu.cn](mailto:tianling@szpu.edu.cn))

Yan Xu ([yan.xu@nwsuaf.edu.cn](mailto:yan.xu@nwsuaf.edu.cn))

**
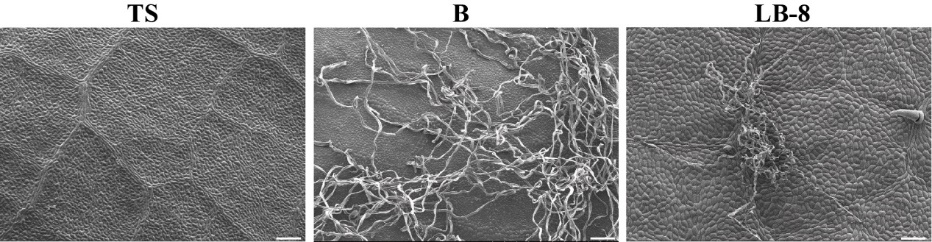
Figures**

**Figure S1**. The adaxial surface of leaves from different germplasms of grapevine was observed by SEM. Scale bar = 100 µm.


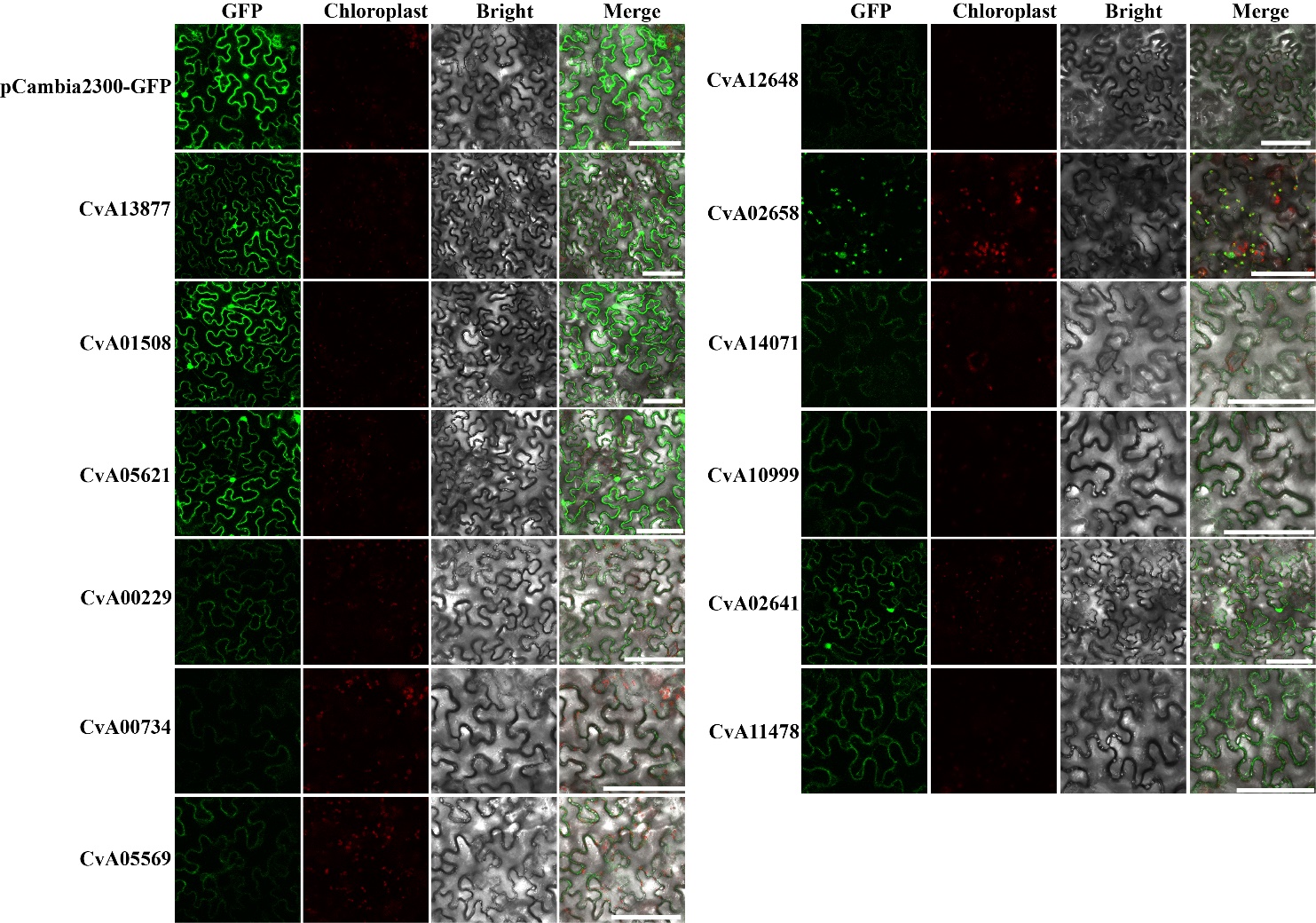


**Figure S2**. Subcellular localization of three effectors in *N. benthamiana* leaves: plasma membrane and the nucleus (CvA13877, CvA01508, CvA05621 and CvA02641), and plasma membrane (CvA00229, CvA00734, CvA05569, CvA12648 CvA14071, CvA10999 and CvA11478), and chloroplast (CvA02658). Effectors-GFP fusion proteins and GFP were transiently expressed in *N. benthamiana* following *Agrobacterium* transformation method. Photographs were taken 24 h after transformation. Scale bars = 100 µm.
